# Combined RNA- and DNA-targeting CRISPR ribonucleoproteins enhance suppression of HBV transcripts

**DOI:** 10.3389/fgeed.2026.1824895

**Published:** 2026-09-23

**Authors:** Puja Banik, Addison C. Hill, Madison B. Schank, Yi Zhang, Juan Zhao, Jaeden S. Pyburn, Holly Orfield, Yuezu Wu, Mohammad J. Hajipour, Mohamed El Gazzar, Shunbin Ning, Jonathan P. Moorman, Zhi Q. Yao, Ling Wang

**Affiliations:** 1 Center of Excellence in Inflammation, Infectious Disease and Immunity, James H. Quillen College of Medicine, East Tennessee State University, Johnson, TN, United States; 2 Department of Internal Medicine, Division of Infectious, Inflammatory and Immunologic Diseases, Quillen College of Medicine, ETSU, Johnson, TN, United States; 3 Hepatitis (HCV/HBV/HIV) Program, James H. Quillen VA Medical Center, Department of Veterans Affairs, Johnson, TN, United States

**Keywords:** Cas12a, Cas13d, Cas9, gRNA, HBV transcripts, inhibition, ribonucleoprotein

## Abstract

**Introduction:**

Chronic hepatitis B virus (HBV) infection remains a major global health problem and there is no curative treatment currently available. RNA-targeting CRISPR systems offer a potential alternative to DNA-editing nucleases by enabling direct degradation of viral transcripts without introducing genomic breaks. In this report, we evaluated the antiviral activity of synthetic guide RNA (gRNA)/Cas13d ribonucleoproteins (RNPs) targeting HBV RNA and a combination use with DNA-targeting RNPs.

**Methods:**

We designed 10 gRNAs targeting conserved regions across the HBV transcriptome followed by screening for best candidate gRNA for Cas13d. To enhance antiviral efficacy, we evaluated multiplex RNA targeting using combined gRNA6+gRNA7/Cas13d RNPs, and combined RNA-targeting gRNA7/Cas13d with previously validated HBV DNA-targeting gRNA9/Cas9 and gRNA4/Cas12a RNPs for HBV treatment.

**Results:**

Amongst these 10 gRNAs, gRNA7/Cas13d exhibited selective knockdown of its specific target RNA transcript without inducing a broad suppression of total HBV DNA, mRNAs, and proteins. The antiviral effect of gRNA7/Cas13d RNP was transient, diminishing by Day 3 post the treatment. The combined gRNA6+gRNA7/Cas13d treatment also did not further induce global HBV suppression compared with single gRNAs, although gRNA7/Cas13d still showed a robust target-specific RNA cleavage during the combined treatment in HBV cellular models. While gRNA9/Cas9 and gRNA4/Cas12a exhibited the dominant antiviral effects, either alone or in combination, leading to substantial reductions in HBV replication and expression, the addition of gRNA7/Cas13d further enhanced the suppression of the gRNA7-specific HBV RNA transcript, achieving up to an 80% reduction in the target RNA level.

**Discussion:**

Collectively, these findings demonstrate that gRNA7/Cas13d can mediate target-specific HBV RNA cleavage but has limited overall antiviral activity when used alone as a single-agent; however, the combination of DNA- and RNA-targeting CRISPR-RNPs may provide a complementary approach for enhancing target-specific HBV RNA suppression.

## Introduction

Chronic hepatitis B virus (HBV) infection remains a major global healthcare challenge, affecting more than 250 million people worldwide and contributing to significant morbidity and mortality from cirrhosis and hepatocellular carcinoma (HCC) (Schweitzer et al., 2015; Ott et al., 2017). Current antiviral therapies, including nucleos(t)ide analogs (NAs) and interferon-α, effectively suppress viral replication but rarely achieve complete viral eradication, primarily due to the persistence of covalently closed circular DNA (cccDNA) in infected hepatocytes (Gish et al., 2015; Koumbi, 2015; Ye and Chen, 2021). The cccDNA exists as an episomal minichromosome in the nucleus of infected cells and serves as the transcriptional template for all viral RNAs, including the pregenomic RNA (pgRNA), which functions as the template for viral reverse transcription and HBV production (Guo and Guo, 2015; Nassal, 2015; Zhu et al., 2019). Therefore, developing novel strategies that can permanently eliminate or silence HBV genetic materials (including both DNA and RNA components) in infected cells remains an unmet medical need for HBV eradication.

Recent advances in RNA-guided RNA-targeting CRISPR systems have opened novel strategies for disrupting viral genomes. While the widely used DNA-targeting Cas9 and Cas12 systems have been explored for cleavage of integrated viral DNA or host genomic sequences, their mechanism of creating double-strand breaks in DNA raises significant safety concerns about off-target mutagenesis, framework deletions, chromosomal rearrangements, and genomic instability (Kosicki et al., 2018; Park et al., 2022; Hwang et al., 2025). In contrast, the RNA-targeting CRISPR effector Cas13 d (also known as RfxCas13 d or CasRx) offers a programmable, RNA-cleaving approach that degrades viral RNA transcripts without directly modifying host DNA (Nguyen et al., 2021; Gupta et al., 2022; Zeng et al., 2022; Hussein et al., 2024).

Cas13 d is a compact Type VI-D CRISPR-Cas effector (∼930 aa) that uses a single guide RNA (gRNA) to directly bind to and cleave single-stranded RNA (ssRNA); it does not require a protospacer adjacent motif (PAM) or protospacer flanking sequence (PFS) for target recognition, and can therefore be delivered in RNA, mRNA, viral vectors and other vehicles for therapeutic applications (Konermann et al., 2018; Zhang et al., 2018; Kuo et al., 2024). Previous studies have shown that Cas13 d directed at conserved sequences of the SARS-CoV-2 genome achieved >99% reduction of viral titers and maintained activity against multiple variants in human airway-epithelium cultures (Zeng et al., 2022). Another study explored the influence of RNA secondary structure on Cas13 d cleavage efficiency and affirmed its robustness in cleaving even highly structured viral target RNAs (Kuo et al., 2024). Additionally, in a mouse model of Huntington’s disease, Cas13 d was used to selectively reduce mutant Huntington (HTT) mRNA and protein, yielding durable phenotypic improvements and showing minimal off-target effects on the cell transcriptome (Morelli et al., 2023). Although there are fewer published studies specifically using Cas13 d against the HBV genome, related CRISPR systems directed against viral RNA have been applied. A recent study showed that reprogrammed CRISPR-PspCas13 b can efficiently suppress HBV RNAs, resulting in decreased HBV DNA replication and viral protein expression in cell culture models. Furthermore, hydrodynamic injection of HBV, PspCas13b, and its corresponding crRNA plasmids into mice reduced serum HBsAg levels by approximately 47% (McCoullough et al., 2024). These studies provide a proof-of-concept that RNA-guided degradation of HBV transcripts is feasible, and suggests that Cas13d, given its compact size, high specificity, and efficient RNA-cleaving activity, could be adapted as an additional anti-HBV therapeutic.

We have previously reported the use of synthetic gRNA/Cas9 and gRNA/Cas12a RNPs to cleave HBV DNA for viral suppression (Zhang et al., 2023; Hill et al., 2026a; Hill et al., 2026b). In this study, we designed 10 Cas13 d gRNAs targeting different regions of the HBV transcriptome and evaluated their antiviral potential. Amongst these candidates, gRNA6, gRNA7, and gRNA8 significantly reduced their targeted RNA transcripts, respectively. Notably, gRNA7 exhibited the highest knockdown efficiency of its target RNA transcript. Despite being effective at target RNA degradation, this targeted RNA knockdown did not translate into measurable decreases in total HBV DNA, mRNA, and proteins. Importantly, in the combination treatment, gRNA9/Cas9 and gRNA4/Cas12a remained the main contributors to overall antiviral activity, while the addition of gRNA7/Cas13 d further enhanced suppression of the targeted HBV RNA transcript. These findings suggest that while Cas13 d can selectively degrade HBV RNA transcripts, further optimization of gRNA design, delivery efficiency, and combinatorial targeting strategies or treatment protocols are necessary to achieve a broader antiviral efficacy.

## Materials and methods

### Cell culture

The HepG2.2.15 Human Hepatoblastoma cell line (Millipore Sigma, Burlington, MA, Cat# SCC249), a traditional HBV cellular model that can constitutively express HBV DNA, mRNAs, and proteins, was cultured in DMEM/F-12 media, supplemented with 10% heat-inactivated Fetal bovine serum (Fisher Scientific, Waltham, MA, Cat # A5256801), 100 IU/mL penicillin-streptomycin (Fisher Scientific, Cat # 15070063), 2 mM L-glutamine (Fisher Scientific, Cat # 25030081), and 400 μg/mL G418 Sulfate (Corning, Tewksbury, MA, Cat # 61–234-RG). The HepDE19 cell line is a robust HBV cell model that supports the expression of all HBV life-cycle transcripts. Cells were maintained in complete DMEM/F-12 culture medium supplemented with 1 μg/mL tetracycline (Tet) and 400 μg/mL G418. To induce HBV replication, HepDE19 cells were cultured in Tet-free medium for 3 days prior to all gRNA/Cas13 d RNP or combined gRNA/Cas9 and gRNA/Cas12a RNP treatment. Cells were maintained on collagen-coated plates in a 37 °C incubator with a 5% CO_2_ atmosphere.

### Design and synthesis of gRNAs targeting the HBV genome

Using the online CRISPR design tool CHOPCHOP (v3.7) to analyze the HBV ayw strain (GenBank accession no. NC_003977.2), we identified 10 unique gRNAs that target one or more highly conserved regions in the HBV genome, including polymerase (P), capsid (C), surface (S), and X genes (Table 1). The selected 10 gRNAs and a non-targeting scramble gRNA control (gRNAc) were synthesized by Integrated DNA Technologies (IDT, Coralville, IA) with RNA-stabilizing modifications (5′ and 3′ Alt-R).

**TABLE 1:** The features of Cas13d gRNAs targeting HBV genomes.

gRNA #	Target	Genomic location (nt)	Target sequence	GC content (%)	Self-complementarity	MM0	MM1	MM2	MM3
1	P/X	1596	CCTCTGCACGTCGCATGGAGACCACCGT	63	0	0	0	0	0
2	S/P	366	TGGTTATCGCTGGATGTGTCTGCGGCGT	59	0	0	0	0	0
3	Pre-C	1848	TTGTTCATGTCCTACTGTTCAAGCCTC	44	0	0	0	0	0
4	S/P	248	GTCTAGACTCGTGGTGGACTTCTCTCA	52	0	0	0	0	0
5	X	1756	TAGGTTAAAGGTCTTTGTACTAGGAGG	41	0	0	0	0	0
6	S/P	538	GCTCAAGGAACCTCTATGTATCCCTCC	52	0	0	0	0	0
7	X/Pre-C	1813	ACCATGCAACTTTTTCACCTCTGCCTA	44	0	0	0	0	0
8	C	2276	GGATTCGCACTCCTCCAGCTTATAGAC	52	0	0	0	0	0
9	X	1725	TTAAAGACTGGGAGGAGTTGGGGGAGG	56	0	0	0	0	0
10	PreS1/P	2994	GCTGGAGCATTCGGGCTGGGTTTCACC	63	0	0	0	0	0

### Formation of gRNA/Cas13 d RNP complexes and cell nucleofection

To assemble the gRNA/Cas13 d RNP complex, 1,000 pmol of gRNA or gRNAc was mixed with 174 pmol of Cas13 d protein (MCLAB, South San Francisco, CA, Cat # CAS13d-200) along with Cas13 d reaction buffer (MCLAB, Cat # CAS13BUF-200-D; 20 mM HEPES, 5 mM MgCl2, 100 mM NaCl, 0.1 mM EDTA, pH 6.5) and incubated at 37 °C for 20 min. For the combined gRNA^6^ and gRNA7 treatment, 500 pmol each of gRNA6 and gRNA7 were mixed with 174 pmol of Cas13 d protein under the same reaction conditions. The assembled gRNA/Cas13 d RNP complexes were subsequently kept on ice to preserve protein activity. For gRNA/Cas13 d treatment, 1 × 106 HepG2.2.15 or HepDE19 cells were nucleofected with the gRNA/Cas13 d RNPs using the SF Cell Line 4D-Nucleofector X Kit (Lonza, Basel, Switzerland, Cat# V4XC-2024) and a 4D nucleofector core unit with the manufacturer’s program optimized for HepG2 cells. After various time points post-nucleofection, the cell culture supernatants and cell pellets were collected for ELISA and qPCR analysis, respectively. Cell numbers and viabilities were determined using 0.4% Trypan Blue Stain (Thermo Fisher Scientific, Waltham, MA, Cat #15250–061) with an Invitrogen Countess 3 cell counter.

### Combination treatment with gRNA9/Cas9, gRNA4/Cas12a, and gRNA7/Cas13 d RNPs

gRNA9/Cas9 and gRNA4/Cas12a RNP complexes were freshly prepared before each experiment as described previously (Hill et al., 2026b). For gRNA9/Cas9 RNP assembly, 125 pmol of gRNA9 (IDT) or gRNAc (Horizon Discovery, Cambridge, UK, Cat # U-007501–01–20) was mixed with 125 pmol of tracrRNA (Horizon Discovery, Cat # U-002005–200) and incubated at 37 °C for 30 min, followed by incubation with 100 pmol Cas9 protein (MacroLAB, Berkeley, CA) at 37 °C for 15 min. For gRNA4/Cas12a RNP assembly, 1,000 pmol of gRNA4 or gRNAc (IDT) was incubated with 670 pmol of Cas12a protein (IDT, Cat # 10007924) at room temperature for 20 min. The gRNA9/Cas9 and gRNA4/Cas12a RNPs were mixed at a 1:1 ratio before cell nucleofection. The total amount of RNP complexes introduced per nucleofection was kept constant across all treatment groups. Three (for HepG2.2.15 cells) or two (for HepDE19 cells) days after nucleofection with gRNA9/Cas9 and/or gRNA4/Cas12a, the cells were nucleofected again with gRNA7/Cas13 d or gRNAc/Cas13 d RNPs. After an additional 24 h, the cell culture supernatants and cell pellets were collected for ELISA and qPCR analyses, respectively. Cell numbers and viabilities were determined using 0.4% Trypan Blue with an Invitrogen Countess 3 cell counter.

### Real-time qPCR

Total RNA was extracted from 5 x10^5^ treated cells using the Qiagen RNeasy Mini Kit (QIAGEN, Hilden, Germany, Cat # 74106). Complementary DNA (cDNA) was synthesized from extracted RNA using the High-Capacity Reverse Transcription Kit (Applied Biosystems, Waltham, MA, Cat # 4368813). Real-time quantitative PCR (qPCR) was performed to amplify HBV mRNA, pgRNA, and specific mRNA transcripts containing the HBV gRNA/Cas13 d target sites, using the iTaq Universal SYBR Green Master Mix (Bio-Rad, Hercules, CA; Cat# 1725124). All PCR primers were synthesized by IDT, and their sequences are listed in Table 2. The PCR cycling conditions were as follows: one cycle of enzyme activation at 95 °C for 3 min, 40 cycles of denaturing at 95 °C for 10 s, and annealing and extension at 60 °C for 1 min. Gene expression levels were determined by the 2^−ΔΔCt^ threshold method, normalized first to β−2−microglobulin levels and then to the non-targeting control (gRNAc). The results were presented as fold change relative to the control.

**TABLE 2:** The sequences of PCR primers.

Target	Forward (5’→3’)	Reverse (5’→3’)	Internal Probe (5’→3’)
β-2-microglobulin	TGCTGTCTCCATGTTTGATGTATCT	TCTCTGCTCCCCACCTCTAAGT	n/a
HBV mRNA	GAGTGCTGTATGGTGAGGTG	TTTGGGGCATGGACATTGAC	n/a
HBV pgRNA	TGGATTCGCACTCCTCCAGCTT	GGGACCTGCCTCGTCGTCTA	n/a
HBV mRNA/gRNA1	ACTTCGCTTCACCTCTGCAC	TGCTGAGAGTCCAAGAGTCC	n/a
HBV mRNA/gRNA2	AAATTCGCAGTCCCCAACCT	AGAGGACAAACGGGCAACAT	n/a
HBV mRNA/gRNA3	CATGCAACTTTTTCACCTCTGCCT	GCACAGCTTGGAGGCTTGAAC	n/a
HBV mRNA/gRNA4	GGCGGGGTTTTTCTTGTTGA	AAGACACACGGTAGTTCCCC	n/a
HBV mRNA/gRNA5, 9	CTCAGCAATGTCAACGACCG	GCGCAGACCAATTTATGCCT	n/a
HBV mRNA/gRNA6	ATGTTGCCCGTTTGTCCTCT	GGTGCAATTTCCGTCCGAAG	n/a
HBV mRNA/gRNA7	CGACCGACCTTGAGGCATAC	AAAAGTTGCATGGTGCTGGT	n/a
HBV mRNA/gRNA8	CTTTCGGAGTGTGGATTCGC	TGCCTCGTCGTCTAACAACA	n/a
HBV mRNA/gRNA10	CAGACGCCAACAAGGTAGGA	CTCAAAGGTGGAGACAGCGG	n/a
HBV DNA	CCGTCTGTGCCTTCTCATCTG	AGTCCAAGAGTYCTCTTATGYAAGACCTT	/56-FAM/CCGTGTGCACTTCGCTTCACCTCTGC/36-TAMSp/
HBV cccDNA	TCATCTGCCGGACCGTGTGC	TCCCGATACAGAGCTGAGGCGG	/56-FAM/TTCAAGCCTCCAAGCTGTGCCTTGGGTGGC/36-TAMSp/

Total DNA was isolated from 1 × 106 treated cells using the PureLink™ Genomic DNA Mini Kit (ThermoFisher, Waltham, MA, Cat #K182002). The total HBV DNA was quantified using PrimeTime™ Gene Expression Master Mix (IDT, Cat # 1055772), following the manufacturer’s protocol for HBV primers and probe. PCR primers and probes were synthesized by IDT, and their sequences are shown in Table 2. The PCR conditions were: 95 °C for 10min, followed by 45 cycles of 95 °C for 15 s and 60 °C for 1 min. The DNA levels were determined by the 2^−ΔΔCT^ threshold method, and values were normalized to gRNAc.

### HBeAg and HBsAg ELISA

The levels of HBV antigens in the culture supernatants of treated cells were determined using a Human HBeAg ELISA Kit (Novus Biologicals, Centennial, CO, Cat# NBP2-60029–1) and a Human HBsAg ELISA Kit (Novus Biologicals, Cat# KA0286) according to the manufacturer’s instructions. Absorbance was read at 450 nm using a BioTek Synergy H1 Microplate Reader (BioTek, Winooski, VT), and OD450 values were normalized to gRNAc.

### Statistical analysis

Data were analyzed using the GraphPad Prism 10.6.0 software and are presented as mean ± SEM. Comparisons among multiple groups were performed using one-way or two-way ANOVA, as appropriate. Comparisons between treated and control groups were performed using paired t-tests. P-values <0.05 (*) were considered statistically significant, and p-values <0.01 (**), <0.001 (***), or <0.0001 (****) were considered very significant.

In Silico–Predicted CRISPR Off-Target Analysis. Potential off-target sites for Cas9 HBV gRNA9 and Cas12a HBV gRNA4 were identified by *in silico* analysis and evaluated using targeted rhAmpSeq™ sequencing. Candidate sites were selected based on predicted off-target potential and genomic location, and custom primer panels were designed for targeted amplification and Illumina sequencing. Detailed procedures for off-target site selection, rhAmpSeq panel design, library preparation, sequencing, and data analysis are provided in the Supplemental Methods.

## Results

### Designing Cas13 d-specific gRNAs targeting the HBV transcriptome

Designing Cas13 d-specific gRNAs that effectively target critical HBV transcripts involved in viral replication while minimizing off-target effects is a key step in developing CRISPR-based antiviral therapeutics. Using the online CRISPR design tool CHOPCHOP (Labun et al., 2019) to analyze the HBV ayw strain sequence (GenBank accession no: NC_003977.2), we identified 10 unique gRNAs directed against highly conserved regions within the HBV genome, including the polymerase (P), core (C), surface (S), and X genes (Figure 1A). Candidate gRNAs were further screened against the human transcriptome to ensure minimal off-target complementarity, a critical consideration for RNA-targeting approaches.

**FIGURE 1:**
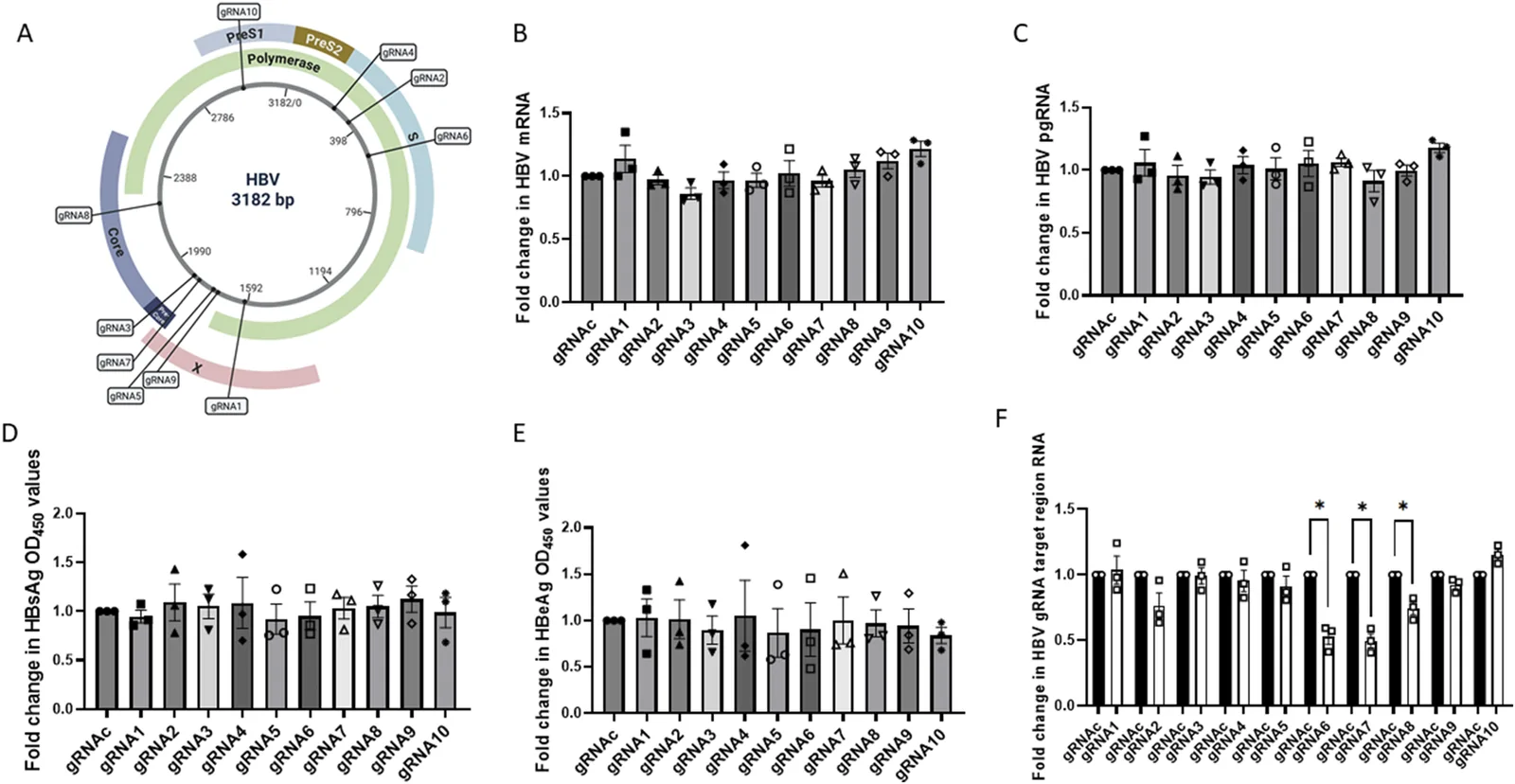
Evaluation of antiviral activity of gRNA/Cas13d candidates in HepG2.2.15 cells. **(A)** Schematic representation of the 10 Cas13 d gRNAs targeting different regions of the HBV genome. Created in BioRender. Wang, L (2026) https://BioRender.com/dyh93fu. **(B,C)** Quantification of total HBV mRNA and pgRNA levels by RT-qPCR in HepG2.2.15 cells at 24 h after nucleofection with individual gRNA/Cas13 d RNPs. **(D,E)** ELISA measurement of HBsAg and HBeAg levels in the supernatants of treated cells. **(F)** RT-qPCR analysis using primers specific for each gRNA target site to detect the corresponding HBV RNA. Data are presented as mean ± SEM from three independent experiments. Statistical significance was determined relative to gRNAc (*P < 0.05), using one-way ANOVA for panels B–E and paired t-test for **(F)**.

Each selected gRNA consisted of a conserved direct repeat (DR, CCC​CTA​CCA​ACT​GGT​CGG​GGT​TT) sequence at the 5′end that forms the stem-loop handle necessary for Cas13 d binding and function, followed by a 27–28 nt HBV-specific spacer sequence complementary to the target RNA. The design criteria and features of these Cas13 d gRNAs - including target gene(s), genomic location of the corresponding transcript, GC content, self-complementarity, predicted on-target efficiency, and potential off-target hits with 0–3 mismatches - are presented in Table 1.

For comparison and validation, Cas13 d gRNA1 and gRNA2 were designed to target the same HBV regions as our previous published Cas9 gRNA9 and gRNA5, respectively (Zhang et al., 2023); Cas13 d gRNA3 and gRNA4 correspond to our Cas9 gRNA10 and gRNA11 (Hill et al., 2026a); and Cas13 d gRNA5–gRNA10 were matched with the target sites of our Cas12a gRNA1–gRNA6 (Hill et al., 2026b). These correspondences enabled direct comparison of RNA-targeting Cas13 d activity with our DNA-targeting CRISPR approaches.

Testing the antiviral activity of gRNA/Cas13 d RNP candidates in HepG2.2.15 cells. The HepG2.2.15 hepatoma cell line, which constitutively replicates and expresses HBV, is a well-established model for evaluating the efficacy and cytotoxicity of antiviral candidates (Sells et al., 1987). We therefore assessed the antiviral activity of our synthetic gRNA/Cas13 d RNPs in this persistently HBV-producing cell model. The 24-h time point was selected to capture the early activity of Cas13 d RNP following nucleofection, consistent with previous studies showing peak RNP-mediated knockdown at this time (Méndez-Mancilla et al., 2022). Thus, HepG2.2.15 cells were harvested at 24 h post-nucleofection with RNPs, and levels of HBV mRNA, pgRNA, and DNA in treated cells were quantified by RT-qPCR or qPCR, and HBV proteins (HBsAg and HBeAg) in the culture supernatants were measured by ELISA.

Analysis of total HBV transcripts revealed that no significant differences in HBV mRNA and pgRNA levels were observed following treatment with any HBV-targeting gRNA/Cas13 d RNPs compared with gRNAc/Cas13 d control (Figures 1B,C). In parallel, no significant differences were detected in HBsAg or HBeAg levels among all groups (Figures 1D,E).

To assess target-specific RNA cleavage, RT-qPCR analysis was performed using primers flanking the individual gRNA targeting sites. As shown in Figure 1F, gRNA6, gRNA7, and gRNA8 each produced a significant reduction in mRNA levels corresponding to their respective target sites, indicating effective and sequence-specific Cas13 d-mediated HBV RNA cleavage. Amongst the 10 gRNAs tested, gRNA7 was selected for subsequent experiments due to its higher suppression efficiency compared with gRNA8 and greater stability relative to gRNA6.

These results indicate that although Cas13 d can be directed to cleave specific HBV transcripts, the resulting RNA degradation was limited in scope and did not lead to a broader suppression of other viral RNAs or antigen expression after 24 h of treatment. These findings highlight the complexity of developing RNA-targeted antiviral agents using this strategy and emphasize the need for further optimization of gRNA design and/or Cas13 d delivery efficiency.

Evaluation of the antiviral activity of gRNA7/Cas13 d RNP in HepG2.2.15 cells over time. To further assess the time-based effects of the most active gRNA7/Cas13 d RNP, we monitored HBV markers at Day 1, Day 3, and Day 6 post-treatment in HepG2.2.15 cells. As shown in Figures 2A–E, the treatment with gRNA7/Cas13 d did not change HBV mRNA, pgRNA, HBV DNA, or secreted HBsAg and HBeAg levels at any of the time-points tested, compared with gRNAc/Cas13 d control. When specific primers were used to quantify RNA transcripts corresponding to the gRNA7 target region (Figure 2F), a significant reduction in the targeted mRNA was observed at Day 1 post-treatment, indicating a transient Cas13 d-mediated RNA cleavage at its target site. However, this reduction gradually diminished over time, and by Day 3 and Day 6, no significant differences were detected between gRNA7/Cas13 d-treated cells and controls.

**FIGURE 2:**
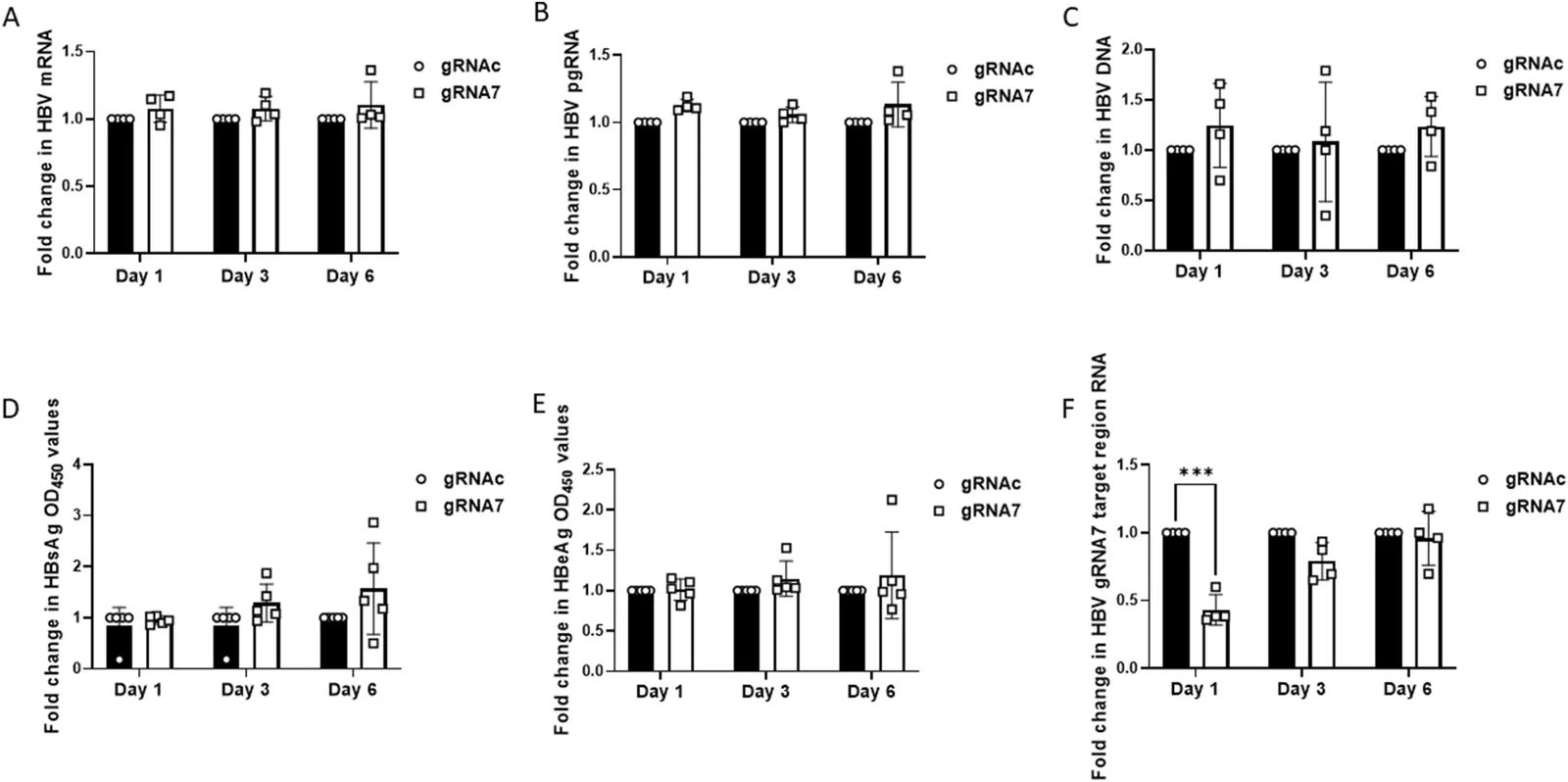
Analysis of gRNA7/Cas13d RNP antiviral activity in HepG2.2.15 cells over-time. HepG2.2.15 cells were treated with gRNA7/Cas13 d or gRNAc/Cas13 d for 1, 3 or 6 days. **(A–C)** Quantification of intracellular HBV total mRNA and pgRNA by RT-qPCR, and HBV DNA by qPCR. **(D,E)** ELISA measurement of HBsAg and HBeAg levels in the supernatants. **(F)** RT-qPCR analysis using primers specific to the gRNA7 target region to detect the corresponding HBV RNA levels. Data are presented as mean ± SEM from four independent replicates. Statistical significance was determined by a paired t-test (***P < 0.001).

These findings suggest that the antiviral activity of gRNA7/Cas13 d was both target-specific and short-lived, insufficient to induce sustained suppression of viral RNA replication or antigen production in this model. Further optimization of gRNA stability, RNP delivery, repeated dosing, and combination with the DNA-targeting approach may be necessary to achieve an effective and prolonged total RNA degradation and HBV suppression.

### Assessment of dual-gRNA Cas13 d-mediated HBV RNA targeting in persistent HBV cell models

To further evaluate whether multiplex RNA targeting could enhance antiviral efficacy, combined gRNA6/Cas13 d plus gRNA7/Cas13 d treatment was examined in both HepG2.2.15 and DE19 cells at Day 1 post treatment (Figure 3). In both HepG2.2.15 and HepDE19 cells, neither single nor combined gRNA6/Cas13 d and gRNA7/Cas13 d treatments significantly reduced total HBV mRNA, pgRNA, HBsAg or HBeAg levels compared with the gRNAc/Cas13 d control (Figures 3A–D,G-J).

**FIGURE 3:**
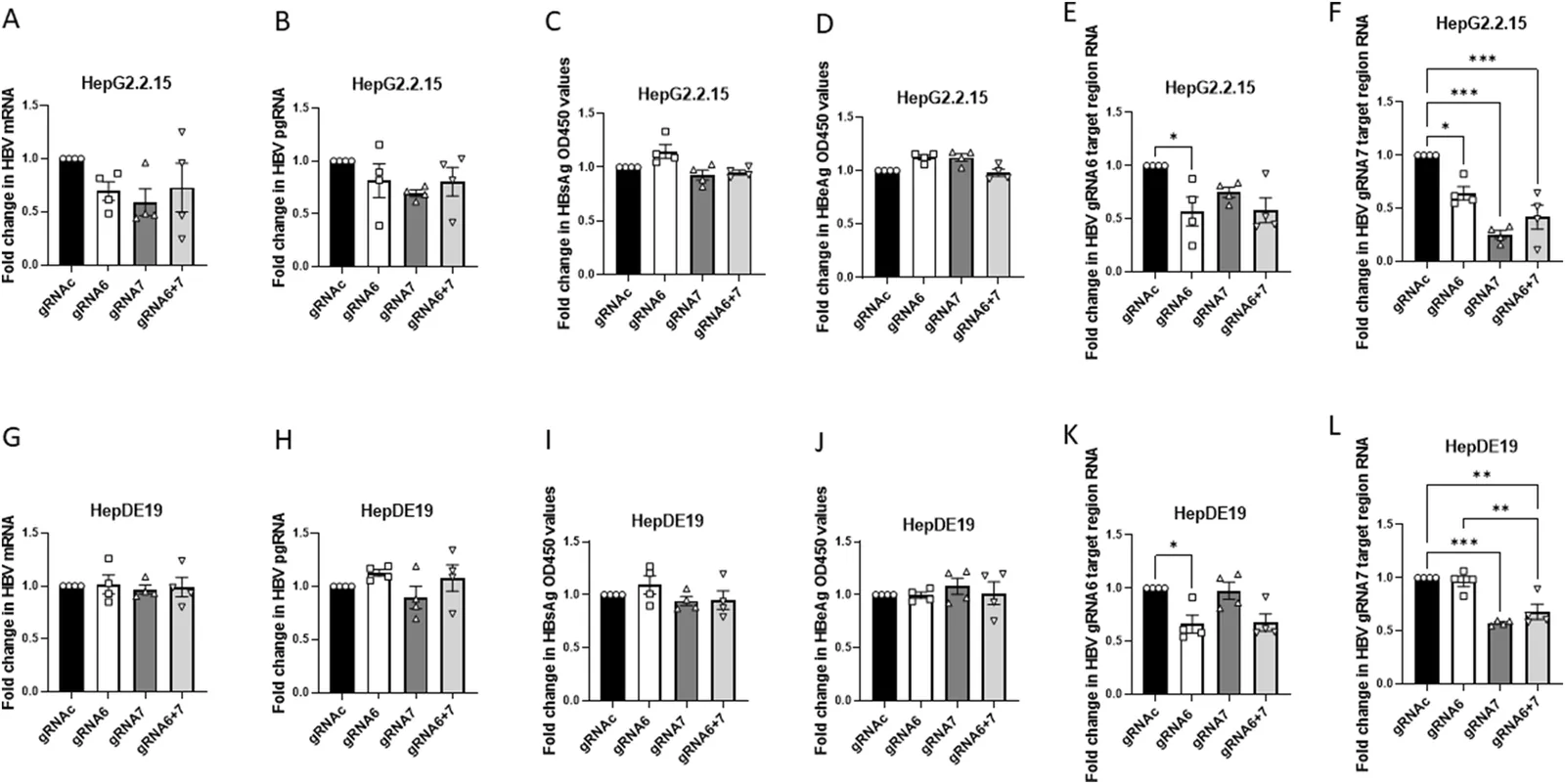
Assessment of gRNA6/Cas13d plus gRNA7/Cas13 d-mediated HBV RNA targeting in HepG2.2.15 and HepDE19 cells. HepG2.2.15 and HepDE19 cells were treated with gRNA6/Cas13d, gRNA7/Cas13d, gRNA6/Cas13 d plus gRNA7/Cas13 d or gRNAc/Cas13 d for 1 day. **(A,B)** Quantification of intracellular HBV total mRNA and pgRNA by RT-qPCR in treated HepG2.2.15 cells. **(C,D)** ELISA measurement of HBsAg and HBeAg levels in the supernatants from treated HepG2.2.15 cells. **(E,F)** RT-qPCR analysis using primers specific to the gRNA6 or gRNA7 target region to detect the corresponding HBV RNA levels in treated HepG2.2.15 cells. **(G,H)** Quantification of intracellular HBV total mRNA and pgRNA by RT-qPCR in treated HepDE19 cells. **(I,J)** ELISA measurement of HBsAg and HBeAg levels in the supernatants from treated HepDE19 cells. **(K,L)** RT-qPCR analysis using primers specific to the gRNA6 or gRNA7 target region to detect the corresponding HBV RNA levels in treated HepDE19 cells. Data are presented as mean ± SEM from four independent replicates. Statistical significance was determined by two-way ANOVA (*P < 0.05; **P < 0.01; ****P < 0.0001).

Target-region analysis, however, revealed distinct gRNA-dependent RNA cleavage patterns. Single gRNA6/Cas13 d treatment significantly reduced RNA levels corresponding to the gRNA6 target region, whereas this effect was not maintained in the combined gRNA6/Cas13 d plus gRNA7/Cas13 d treatment (Figures 3E,K). In contrast, both single gRNA7/Cas13 d treatment and the combined treatment significantly reduced RNA levels at the gRNA7 target region (Figures 3F,L). Interestingly, in HepG2.2.15 cells, but not in HepDE19 cells, treatment with gRNA6/Cas13 d alone also significantly reduced RNA levels measured at the gRNA7 target region, although the reduction was less pronounced than that observed with gRNA7/Cas13 d alone or the combined treatment (Figure 3F). Nevertheless, no significant differences were observed between single and combined treatments, possibly because half doses of each gRNA were used in the combined-treatment group to maintain equal total gRNA amounts across all samples. Importantly, even at half the dose, gRNA7/Cas13 d in the combined treatment with gRNA6/Cas13 d significantly reduced RNA levels at its target region. These results further indicate that gRNA7 has stronger and more consistent antiviral activity than gRNA6, supporting its selection for subsequent experiments.

Taken together, these findings indicate that multiplex Cas13 d-mediated targeting using gRNA6 and gRNA7 did not provide additional antiviral effects on global HBV RNA transcription or antigen production under the experimental conditions tested, despite maintaining sequence-specific cleavage activity at the targeted RNA transcripts.

Enhancing antiviral activity by triple combination treatment with gRNA9/Cas9, gRNA4/Cas12a, and gRNA7/Cas13 d RNPs in persistent HBV cell models. Based on our previous findings that combined gRNA9/Cas9 and gRNA4/Cas12a treatment produced an enhanced antiviral activity (Hill et al., 2026b), we next examined whether adding an RNA-targeting CRISPR system could further improve antiviral efficacy following DNA-targeting intervention. To this end, HepG2.2.15 and HepDE19 cells were first treated with gRNA4/Cas12a (targeting the HBV C gene) and gRNA9/Cas9 (targeting the HBV X/P genes) for 2 days (HepDE19) or 3 days (HepG2.2.15), followed by nucleofection with gRNA7/Cas13 d (targeting HBV X/Pre-C transcripts) for an additional day. HBV DNA, RNA, and protein levels were then quantified by real-time qPCR, RT-PCR and ELISA. In HepG2.2.15 cells (Figures 4A–E), treatment with either gRNA9/Cas9, or gRNA4/Cas12a alone, or their combination significantly reduced HBV mRNA, pgRNA, HBV DNA, HBeAg, and HBsAg levels compared with the control group (gRNAc/Cas9 + gRNAc/Cas12a + gRNAc/Cas13 d). In contrast, gRNA7/Cas13 d alone (white bar) did not significantly alter these HBV markers relative to its corresponding controls (black bar). However, when examining the gRNA7-specific target region (Figure 4F), a marked reduction in HBV RNA was observed following gRNA7/Cas13 d treatment in cells pre-treated with gRNA9/Cas9 and/or gRNA4/Cas12a (comparison of black and white bars). Notably, the triple combination treatment (gRNA9/Cas9 + gRNA4/Cas12a + gRNA7/Cas13 d) achieved the strongest suppression, resulting in approximately 80% reduction of the gRNA7-targeted HBV RNA transcript.

**FIGURE 4:**
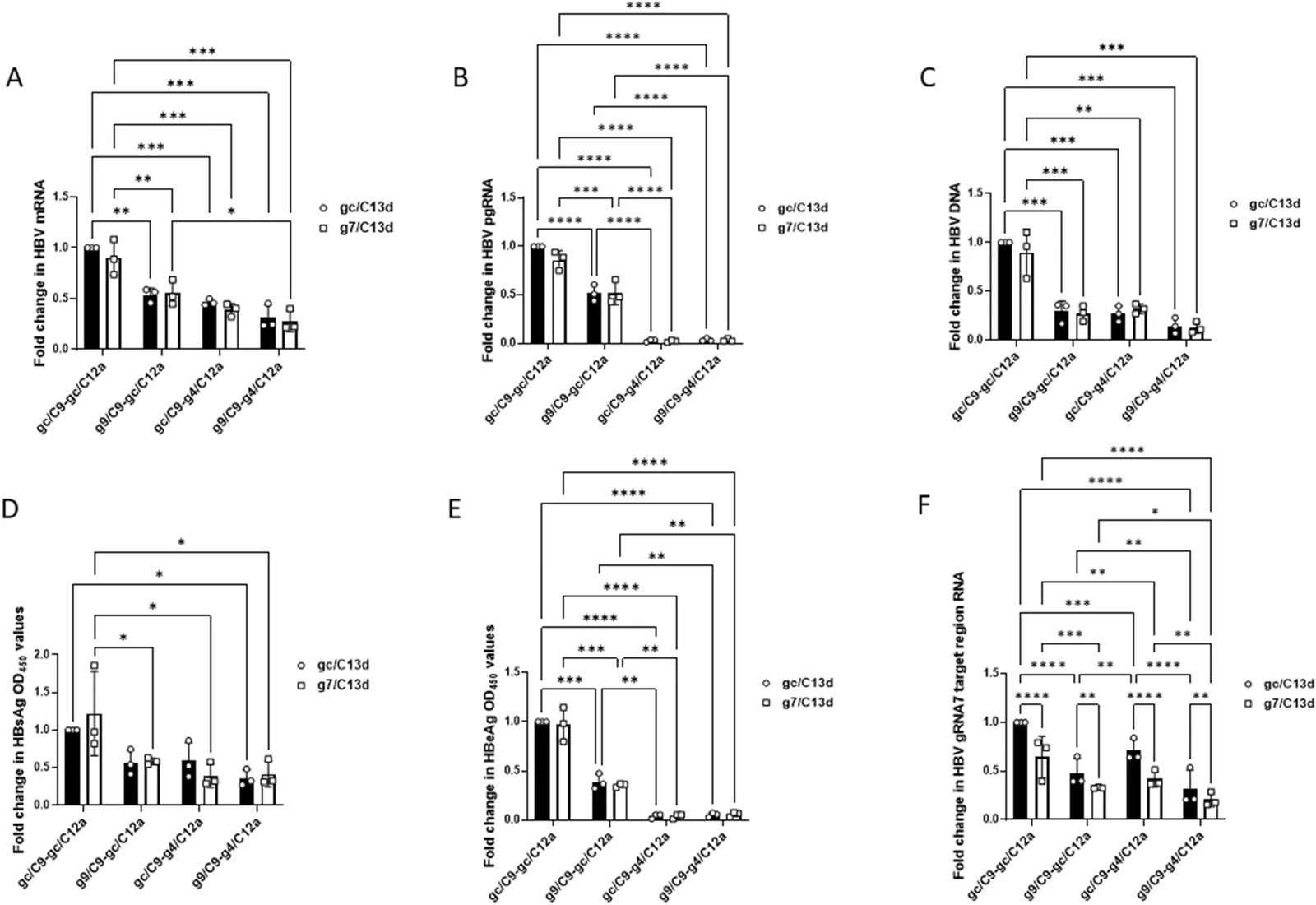
HBV suppression by combined treatment with gRNA9/Cas9, gRNA4/Cas12a, and gRNA7/Cas13 d RNPs in HepG2.2.15 cells. HepG2.2.15 cells were treated with gRNA9/Cas9 and gRNA4/Cas12a for 3 days, followed by nucleofection with gRNA7/Cas13 d for one additional day. HBV total mRNA **(A)**, pgRNA **(B)**, HBV DNA **(C)**, HBsAg **(D)**, and HBeAg **(E)** levels were measured by qPCR and ELISA **(F)** RT-qPCR analysis using primers specific to the gRNA7 target region to detect the corresponding HBV RNA. Data are presented as mean ± SEM from three independent replicates. Statistical significance was determined by two-way ANOVA (*P < 0.05; **P < 0.01; ***P < 0.001; ****P < 0.0001). Abbreviations: gRNAc, non-targeting control guide RNA; g9, gRNA9; g4, gRNA4; C9, Cas9; C12a, Cas12a; C13d, Cas13d.

Comparable results were observed in HepDE19 cells, where HBV replication and gene expression are induced under tetracycline-free culture conditions and driven by cccDNA, providing a more physiologically relevant model. As shown in Figures 5A–C, treatment with either gRNA9/Cas9, or gRNA4/Cas12a alone, or their combination significantly reduced HBV mRNA, pgRNA, and HBV DNA levels compared with control. For HBsAg, gRNA9/Cas9 treatment alone significantly increased HBsAg levels in the absence of gRNA7/Cas13 d (black bars), whereas this increase was not observed in cells receiving subsequent gRNA7/Cas13 d treatment (white bars) (Figure 5D). Notably, the increase in HBsAg was not accompanied by corresponding increases in HBV DNA, total HBV mRNA, or pgRNA levels (Figures 5A–C). In contrast, HBeAg levels were significantly reduced by gRNA4/Cas12a alone and by the combination treatment, but not by gRNA9/Cas9 alone (Figure 5E). Consistent with the findings in HepG2.2.15 cells, gRNA7/Cas13 d alone did not significantly affect overall HBV markers, yet it significantly reduced RNA levels at the gRNA7 target site in gRNA9/Cas9 and/or gRNA4/Cas12a pre-treated cells (Figure 5F). The triple combination treatment again produced the most pronounced effect, achieving approximately 92% reduction in gRNA7-targeted HBV RNA transcript.

**FIGURE 5:**
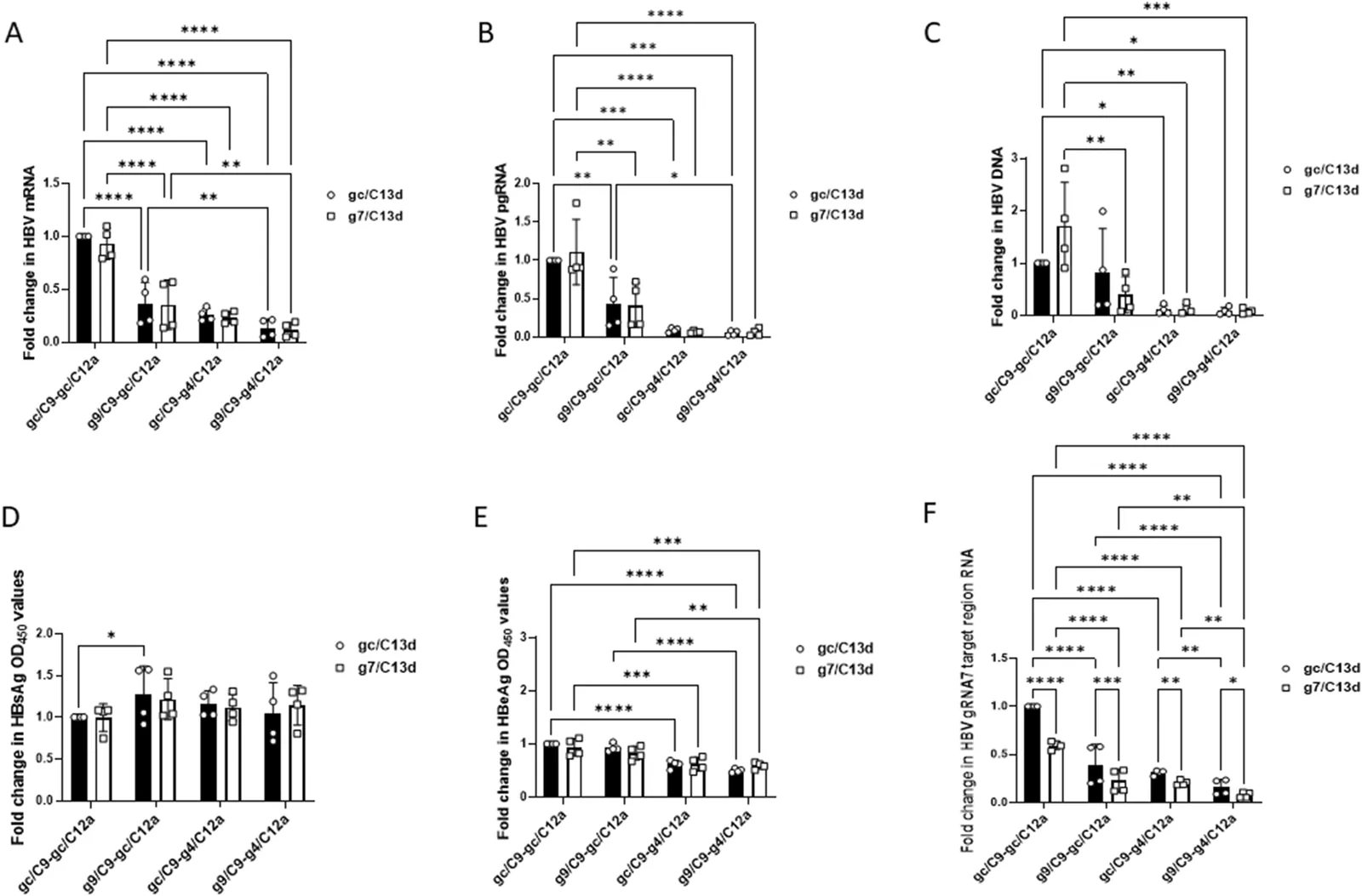
HBV suppression by combined treatment with gRNA9/Cas9, gRNA4/Cas12a, and gRNA7/Cas13 d RNPs in HepDE19 cells. HepDE19 cells were treated with gRNA9/Cas9 and gRNA4/Cas12a for 2 days, followed by nucleofection with gRNA7/Cas13 d for one additional day. HBV total mRNA **(A)**, pgRNA **(B)**, HBV DNA **(C)**, HBsAg **(D)**, and HBeAg **(E)** levels were measured by qPCR and ELISA respectively. **(F)** RT-qPCR analysis using primers specific to the gRNA7 target region to detect the corresponding HBV RNA. Data are presented as mean ± SEM from three independent replicates. Statistical significance was determined by two-way ANOVA (*P < 0.05; **P < 0.01; ***P < 0.001; ****P < 0.0001). Abbreviations: gRNAc, non-targeting control guide RNA; g9, gRNA9; g4, gRNA4; C9, Cas9; C12a, Cas12a; C13d, Cas13 d.

Collectively, these results suggest that combining DNA- and RNA-targeting CRISPR systems may yield enhanced antiviral effects compared with individual treatments. Although gRNA7/Cas13 d alone showed limited impact on global HBV markers, it substantially enhanced targeted HBV RNA suppression when applied following DNA-targeting CRISPR treatments, highlighting the potential of multiplexed CRISPR strategies for achieving a more robust and durable HBV suppression.

Limited Off-Target Editing. To evaluate the specificity of the CRISPR-Cas systems, we performed targeted rhAmpSeq sequencing of predicted off-target sites for the Cas9 and Cas12a HBV-targeting gRNAs. The majority of analyzed sites exhibited >95% unedited reads across all tested samples, indicating minimal detectable sequence alteration at most loci. However, editing was detected at a small subset of the selected sites, with similar editing patterns and frequencies observed across all samples, including those treated with control gRNAs. This finding raised the possibility that the detected sequence variations may reflect pre-existing genomic differences in the cells rather than CRISPR-Cas–mediated off-target activity. Because the initial off-target analysis was based on the HG38 reference genome, sequence differences between the genome of the experimental cells and the reference genome may contribute to the observed editing signals. Overall, these findings indicate limited editing at most evaluated sites and support a relatively favorable specificity profile for the gRNAs used in this study. The complete site-specific sequencing results, including genomic coordinates, predicted variant influence scores, and editing frequencies, are provided in the Supplementary Material.

## Discussion

Developing an effective HBV gene therapy is an unmet medical need and is urgently required for curing chronic HBV infection. This study demonstrates the feasibility of employing the RNA-targeting CRISPR-Cas13 d system to specifically degrade HBV RNA transcripts and explores its integration with DNA-targeting nucleases to improve target-specific antiviral effects. Our results showing gRNA7/Cas13 d-mediated cleavage of its target RNA transcript indicate that sequence context and local RNA structure strongly influence Cas13 d activity. gRNA6/Cas13 d also reduced RNA levels measured at the gRNA7 target region in HepG2.2.15 cells, but not in HepDE19 cells. The mechanism underlying this cell-specific observation remains unclear and warrants further investigation. However, the observed RNA degradation was transient and insufficient to significantly affect overall HBV DNA, RNA, and protein levels, highlighting current limitations in delivery efficiency, intracellular stability of Cas13 d RNPs, and target accessibility within the HBV transcriptome. Because nucleofection efficiency, intracellular persistence of Cas13d/gRNA, and Cas13 d activity against a positive-control transcript were not directly evaluated in this study, the mechanisms underlying the transient antiviral effect remain to be fully elucidated.

Our findings are in contrast with previous studies showing robust RNA suppression by Cas13 d against highly expressed viral targets such as SARS-CoV-2 (Zeng et al., 2022) and other RNA viruses, suggesting that the HBV’s compact and overlapping transcriptional genome may constrain effective target selection. Additionally, the high abundance and stability of HBV transcripts derived from cccDNA may overwhelm transient RNA cleavage activity. Because HBV intermediates are continuously produced by viral DNA in HepG2.2.15 cells, this may explain why one-time treatment with gRNA7/Cas13 d could not lead to a sustained reduction of HBV transcripts. These findings highlight the need for more durable and sustained antiviral strategies. To directly address the long-term efficacy of combinatorial CRISPR targeting, we are planning future studies using fluorescence (GFP/RFP)-labeled Cas proteins (GFP-Cas9 and RFP-Cas12a) to enable enrichment of dual-targeted cells by cell sorting, followed by long-term culture and monitoring of viral suppression *versus* rebound. These studies will help determine whether more complete targeting and/or repeated treatments can achieve more effective and sustained HBV suppression.

Notably, previous studies using Cas13 b have shown broader suppression of HBV RNA, DNA, and protein expression (McCoullough et al., 2024). In contrast, our study showed that Cas13 d only reduced the target site-specific HBV RNA. This discrepancy may reflect variations in Cas13 ortholog activity and delivery approaches, as Cas13 b in reported study was expressed from plasmids enabling sustained activity, whereas Cas13 d was delivered as transient RNPs in this study. Although a direct side-by-side comparison between Cas13 b and Cas13 d was beyond the scope of the present study, direct comparisons under matched experimental conditions may help further clarify these differences and their relative antiviral potential. Optimization of gRNA design, increased RNP dosage, and repeated combination treatments with both DNA- and RNA-targeting gRNA/Cas RNPs may help sustain target RNA degradation and achieve more complete viral suppression.

Importantly, the enhanced antiviral efficacy observed with the triple combination treatment supports a multilayered CRISPR targeting model and strategy to suppress the HBV life cycle (Figure 6). In HBV-replicating hepatoma cells, cccDNA in the nucleus serves as the transcriptional template for HBV RNAs, including pgRNA and subgenomic transcripts. The RNA transcripts are translated in the cytoplasm, where pgRNA is reverse transcribed into relaxed circular DNA (rcDNA) inside cytoplasmic viral capsids, and mature virions are assembled with envelop proteins and secreted. Within this framework, gRNA9/Cas9 and gRNA4/Cas12a disrupt HBV DNA in the nucleus, thereby reducing transcription from cccDNA as well as integrated HBV DNA and limiting replenishment of viral RNA intermediates. The two DNA-targeting CRISPR modalities generate distinct cleavage patterns, with Cas9 producing blunt-end breaks and Cas12a generating staggered DNA breaks, respectively, which may further impair accurate viral DNA repair and re-amplification. The addition of gRNA7/Cas13 d introduces a complementary cytoplasmic layer of intervention by directly degrading specific HBV RNA transcripts. Together, DNA disruption and RNA cleavage target both the viral template and its transcriptional products, contributing to additional reduction of specific viral RNA transcript levels. Thus, this combined approach represents a complementary strategy for targeting multiple stages of the HBV life cycle and provides a conceptual framework for reducing both replicative templates and transcriptional intermediates to limit overall viral output.

**FIGURE 6:**
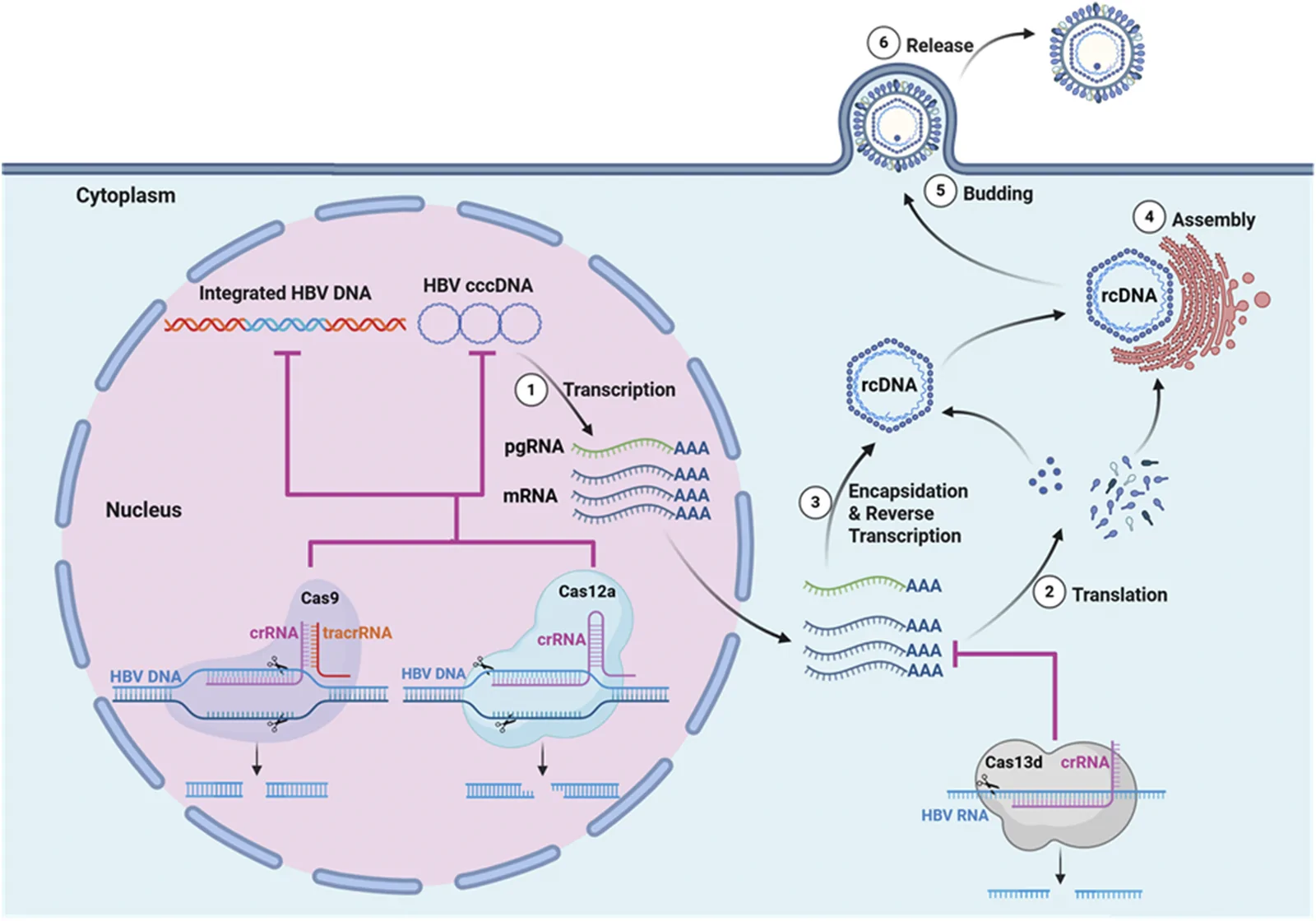
Conceptual model of CRISPR-Cas–mediated suppression of HBV replication in persistent HBV cell models based on experimental trends observed in this study. HBV life cycle (replication, transcription, translation, and viron production) in HepG2.2.15 and HepDE19 cells proceed as following: (1) viral RNAs (including pgRNA) are transcribed from cccDNA and HBV integrated DNA in the nucleus; (2) viral mRNAs are translated into viral proteins in the cytoplasm; (3) pgRNA is encapsidated into nucleocapsids, where reverse transcription generates rcDNA; (4) newly formed nucleocapsids acquire envelope proteins at the interface between the endoplasmic reticulum and the Golgi apparatus (5–6) mature virions bud into the secretory pathway and are subsequently released from the cell. CRISPR-based antiviral strategies target multiple stages of HBV replication and transcription: gRNA9/Cas9 and gRNA4/Cas12a mediate cleavage of HBV DNA in the nucleus, whereas gRNA7/Cas13 d degrades specific HBV RNA transcript in the cytoplasm. This schematic model is created in BioRender. Wang, L (2026) https://BioRender.com/8mcdj58.

Nevertheless, several challenges remain before such a CRISPR combinatorial strategy can be clinically translated. Delivery of multiple large RNPs into hepatocytes, potential immunogenicity, and off-target cleavage of host genomic loci or RNA transcripts must be rigorously addressed. To address safety concerns, we performed initial cell viability assays following RNP nucleofection, with results showing no significant cytotoxicity under the tested conditions (Supplementary Figures S1, S2). Targeted rhAmpSeq analysis of selected candidate sites indicated an overall favorable specificity profile. However, the similar sequence changes observed across treatment groups, including control gRNA-treated samples, raise the possibility that some variants may reflect pre-existing genomic differences rather than CRISPR-Cas–mediated activity. Thus, sample-matched controls and further validation will be important for accurately assessing off-target effects. In future studies, pairing optimized Cas9, Cas12a, and Cas13 d constructs with efficient delivery systems such as lipid nanoparticles (LNPs) or Adeno-Associated Virus (AAV) vectors, and applying multiple treatments with combinatorial or sequential targeting schemes, may achieve more pronounced and durable HBV suppression.

In summary, our results demonstrate that Cas13 d enables sequence-specific HBV RNA cleavage but shows limited antiviral activity when used alone. In the combination setting, the dominant antiviral effects were mediated by the DNA-targeting systems (Cas9 and Cas12a RNPs), whereas Cas13 d contributed mainly to additional targeted RNA knockdown without substantially enhancing overall antiviral efficacy. These findings suggest that multiplexed CRISPR-RNP approaches may contribute to future efforts toward a functional and sustained HBV cure.

## Data Availability

The original contributions presented in the study are included in the article/Supplementary Material, further inquiries can be directed to the corresponding authors.
