# Supplementary material for: Combined RNA- and DNA-targeting CRISPR ribonucleoproteins enhance suppression of HBV transcripts
Source: Front Genome Ed. 2026 Sep 23;8:1824895. doi: 10.3389/fgeed.2026.1824895 (PMC13642003; doi:10.3389/fgeed.2026.1824895)

Analysis mode: CRISPAltRations 1_2_1
Start date: 2026/08/21 15:25
Targets: HBV_Cas9_EditR__ACTR3B, HBV_Cas9_EditR__CCDC60, HBV_Cas9_EditR__CLYBL, HBV_Cas9_EditR__FLYWCH1, HBV_Cas9_EditR__GALNT16, HBV_Cas9_EditR__JAM2, HBV_Cas9_EditR__LHPP, HBV_Cas9_EditR__LINC00301, HBV_Cas9_EditR__LOC105372647, HBV_Cas9_EditR__PRSS55, HBV_Cas9_EditR__RPS19BP1, HBV_Cas9_EditR__SUGP1, HBV_Cas9_EditR__TBCD, HBV_Cas9_EditR__none24, HBV_Cas9_gRNA9__DTNA, HBV_Cas9_gRNA9__LOC101929268, HBV_Cas9_gRNA9__MIR4663, HBV_Cas9_gRNA9__PARP11-AS1, HBV_Cas9_gRNA9__PDE1C, HBV_Cas9_gRNA9__PRRC2B, HBV_Cas9_gRNA9__SHISAL1, HBV_Cas9_gRNA9__none11, HBV_Cas9_gRNA9__none12, HBV_Cas9_gRNA9__none13, HBV_Cas9_gRNA9__none14, HBV_Cas9_gRNA9__none15, HBV_Cas9_gRNA9__none16, HBV_Cas9_gRNA9__none17, HBV_Cas9_gRNA9__none18, HBV_Cas9_gRNA9__none19, HBV_Cas9_gRNA9__none20, HBV_Cas9_gRNA9__none21, HBV_Cas9_gRNA9__none22

Metrics

	Group ID	Treated	Total Reads	Percent Edited	Percent Unedited	Percent NHEJ	Percent Perfect HDR	Percent Imperfect HDR	Percent HDR	Percent Other	Percent In Frame	Percent Frameshift	Percent Indels	Percent Insertions	Percent Templated Insertion	Percent GC-Insertion	Percent Deletions
**HBV_Cas9_EditR__ACTR3B**	HBV_Fig3	TRUE	8262	2.43	97.57	0.05	0.0	0.0	0.0	2.38	99.95	0.05	0.05	0.0	0.0	0.0	0.05
**HBV_Cas9_EditR__CCDC60**	HBV_Fig3	TRUE	3522	1.76	98.24	0.03	0.0	0.0	0.0	1.73	100.0	0.0	0.03	0.03	0.0	0.0	0.0
**HBV_Cas9_EditR__CLYBL**	HBV_Fig3	TRUE	22229	2.35	97.65	0.03	0.0	0.0	0.0	2.32	99.97	0.03	0.03	0.0	0.0	0.0	0.03
**HBV_Cas9_EditR__FLYWCH1**	HBV_Fig3	TRUE	14277	2.25	97.75	0.09	0.0	0.0	0.0	2.16	99.91	0.09	0.09	0.0	0.0	0.0	0.09
**HBV_Cas9_EditR__GALNT16**	HBV_Fig3	TRUE	95404	2.23	97.77	0.22	0.0	0.0	0.0	2.01	99.78	0.22	0.22	0.02	100.0	0.0	0.2
**HBV_Cas9_EditR__JAM2**	HBV_Fig3	TRUE	67979	2.82	97.18	0.05	0.0	0.0	0.0	2.77	99.95	0.05	0.05	0.0	100.0	0.0	0.05
**HBV_Cas9_EditR__LHPP**	HBV_Fig3	TRUE	16237	1.48	98.52	0.01	0.0	0.0	0.0	1.47	99.99	0.01	0.01	0.0	0.0	0.0	0.01
**HBV_Cas9_EditR__LINC00301**	HBV_Fig3	TRUE	43180	99.99	0.01	0.38	0.0	0.0	0.0	99.61	99.94	0.06	0.38	0.02	37.5	0.0	0.36
**HBV_Cas9_EditR__LOC105372647**	HBV_Fig3	TRUE	1148361	89.69	10.31	0.72	0.0	0.0	0.0	88.97	99.31	0.69	0.72	0.42	3.38	0.0	0.32
**HBV_Cas9_EditR__PRSS55**	HBV_Fig3	TRUE	80948	99.9	0.1	0.08	0.0	0.0	0.0	99.82	99.92	0.08	0.08	0.0	100.0	0.0	0.08
**HBV_Cas9_EditR__RPS19BP1**	HBV_Fig3	TRUE	16114	2.09	97.91	0.08	0.0	0.0	0.0	2.01	99.92	0.08	0.08	0.0	0.0	0.0	0.08
**HBV_Cas9_EditR__SUGP1**	HBV_Fig3	TRUE	14709	2.55	97.45	0.15	0.0	0.0	0.0	2.4	99.85	0.15	0.15	0.0	0.0	0.0	0.15
**HBV_Cas9_EditR__TBCD**	HBV_Fig3	TRUE	10018	2.68	97.32	0.04	0.0	0.0	0.0	2.64	99.96	0.04	0.04	0.0	0.0	0.0	0.04
**HBV_Cas9_EditR__none24**	HBV_Fig3	TRUE	34703	2.98	97.02	0.03	0.0	0.0	0.0	2.95	99.97	0.03	0.03	0.0	0.0	0.0	0.03
**HBV_Cas9_gRNA9__DTNA**	HBV_Fig3	TRUE	26516	67.9	32.1	0.01	0.0	0.0	0.0	67.89	99.99	0.01	0.01	0.0	0.0	0.0	0.0
**HBV_Cas9_gRNA9__LOC101929268**	HBV_Fig3	TRUE	52509	3.13	96.87	0.03	0.0	0.0	0.0	3.1	99.97	0.03	0.03	0.0	100.0	0.0	0.03
**HBV_Cas9_gRNA9__MIR4663**	HBV_Fig3	TRUE	63070	3.19	96.81	0.1	0.0	0.0	0.0	3.09	99.9	0.1	0.1	0.1	0.0	0.0	0.1
**HBV_Cas9_gRNA9__PARP11-AS1**	HBV_Fig3	TRUE	76456	2.61	97.39	0.01	0.0	0.0	0.0	2.6	99.99	0.01	0.01	0.0	0.0	0.0	0.01
**HBV_Cas9_gRNA9__PDE1C**	HBV_Fig3	TRUE	48506	2.26	97.74	0.14	0.0	0.0	0.0	2.12	99.86	0.14	0.14	0.02	88.89	0.0	0.13
**HBV_Cas9_gRNA9__PRRC2B**	HBV_Fig3	TRUE	41908	2.59	97.41	0.18	0.0	0.0	0.0	2.41	99.82	0.18	0.18	0.03	100.0	0.0	0.15
**HBV_Cas9_gRNA9__SHISAL1**	HBV_Fig3	TRUE	58584	2.78	97.22	0.14	0.0	0.0	0.0	2.64	99.86	0.14	0.14	0.03	86.67	0.0	0.11
**HBV_Cas9_gRNA9__none11**	HBV_Fig3	TRUE	3408	2.61	97.39	0.0	0.0	0.0	0.0	2.61	100.0	0.0	0.0	0.0	0.0	0.0	0.0
**HBV_Cas9_gRNA9__none12**	HBV_Fig3	TRUE	18052	2.37	97.63	0.08	0.0	0.0	0.0	2.29	99.98	0.02	0.08	0.0	0.0	0.0	0.08
**HBV_Cas9_gRNA9__none13**	HBV_Fig3	TRUE	1254	58.61	41.39	55.98	0.0	0.0	0.0	2.63	44.02	55.98	55.98	0.0	0.0	0.0	55.98
**HBV_Cas9_gRNA9__none14**	HBV_Fig3	TRUE	60558	2.94	97.06	0.18	0.0	0.0	0.0	2.76	99.82	0.18	0.18	0.04	95.83	0.0	0.14
**HBV_Cas9_gRNA9__none15**	HBV_Fig3	TRUE	18015	2.21	97.79	0.0	0.0	0.0	0.0	2.21	100.0	0.0	0.0	0.0	0.0	0.0	0.0
**HBV_Cas9_gRNA9__none16**	HBV_Fig3	TRUE	56	1.79	98.21	0.0	0.0	0.0	0.0	1.79	100.0	0.0	0.0	0.0	0.0	0.0	0.0
**HBV_Cas9_gRNA9__none17**	HBV_Fig3	TRUE	75854	2.7	97.3	0.01	0.0	0.0	0.0	2.69	99.99	0.01	0.01	0.0	0.0	0.0	0.01
**HBV_Cas9_gRNA9__none18**	HBV_Fig3	TRUE	131239	5.8	94.2	0.91	0.0	0.0	0.0	4.89	99.1	0.9	0.91	0.02	0.0	0.0	0.89
**HBV_Cas9_gRNA9__none19**	HBV_Fig3	TRUE	42995	2.59	97.41	0.03	0.0	0.0	0.0	2.56	99.97	0.03	0.03	0.0	0.0	0.0	0.03

**HBV_Cas9_gRNA9__none20**	HBV_Fig3	TRUE	56836	29.28	70.72	0.02	0.0	0.0	0.0	29.26	99.99	0.01	0.02	0.01	0.0	0.0	0.01
**HBV_Cas9_gRNA9__none21**	HBV_Fig3	TRUE	44751	2.74	97.26	0.36	0.0	0.0	0.0	2.38	99.64	0.36	0.36	0.05	95.83	0.0	0.31
**HBV_Cas9_gRNA9__none22**	HBV_Fig3	TRUE	44410	2.65	97.35	0.05	0.0	0.0	0.0	2.6	99.96	0.04	0.05	0.02	0.0	0.0	0.03

Percentages

	% Edited	*% Repair Pathways*	*% Frameshift*
HBV_Cas9_EditR__ACTR3B	97.57%	97.57%	99.95%
HBV_Cas9_EditR__CCDC60	98.24%	98.24%	100.0%
HBV_Cas9_EditR__CLYBL	97.65%	97.65%	99.97%
HBV_Cas9_EditR__FLYWCH1	97.75%	97.75%	99.91%
HBV_Cas9_EditR__GALNT16	97.77%	97.77%	99.78%
HBV_Cas9_EditR__JAM2	97.18%	97.18%	99.95%
HBV_Cas9_EditR__LHPP	98.52%	98.52%	99.99%
HBV_Cas9_EditR__LINC00301	99.99%	99.61%	99.94%
HBV_Cas9_EditR__LOC105372647	.31 89.69%	.31 88.97%	99.31%
HBV_Cas9_EditR__PRSS55	99.9%	99.82%	99.92%
HBV_Cas9_EditR__RPS19BP1	97.91%	97.91%	99.92%
HBV_Cas9_EditR__SUGP1	97.45%	97.45%	99.85%
HBV_Cas9_EditR__TBCD	97.32%	97.32%	99.96%
HBV_Cas9_EditR__none24	97.02%	97.02%	99.97%
HBV_Cas9_gRNA9__DTNA	32.1% 67.9%	32.1% 67.89%	99.99%
HBV_Cas9_gRNA9__LOC101929268	96.87%	96.87%	99.97%
HBV_Cas9_gRNA9__MIR4663	96.81%	96.81%	99.9%
HBV_Cas9_gRNA9__PARP11-AS1	97.39%	97.39%	99.99%
HBV_Cas9_gRNA9__PDE1C	97.74%	97.74%	99.86%
HBV_Cas9_gRNA9__PRRC2B	97.41%	97.41%	99.82%
HBV_Cas9_gRNA9__SHISAL1	97.22%	97.22%	99.86%
HBV_Cas9_gRNA9__none11	97.39%	97.39%	100.0%
HBV_Cas9_gRNA9__none12	97.63%	97.63%	99.98%
HBV_Cas9_gRNA9__none13	41.39% 58.61%	41.39% 55.98%	44.02% 55.98%
HBV_Cas9_gRNA9__none14	97.06%	97.06%	99.82%

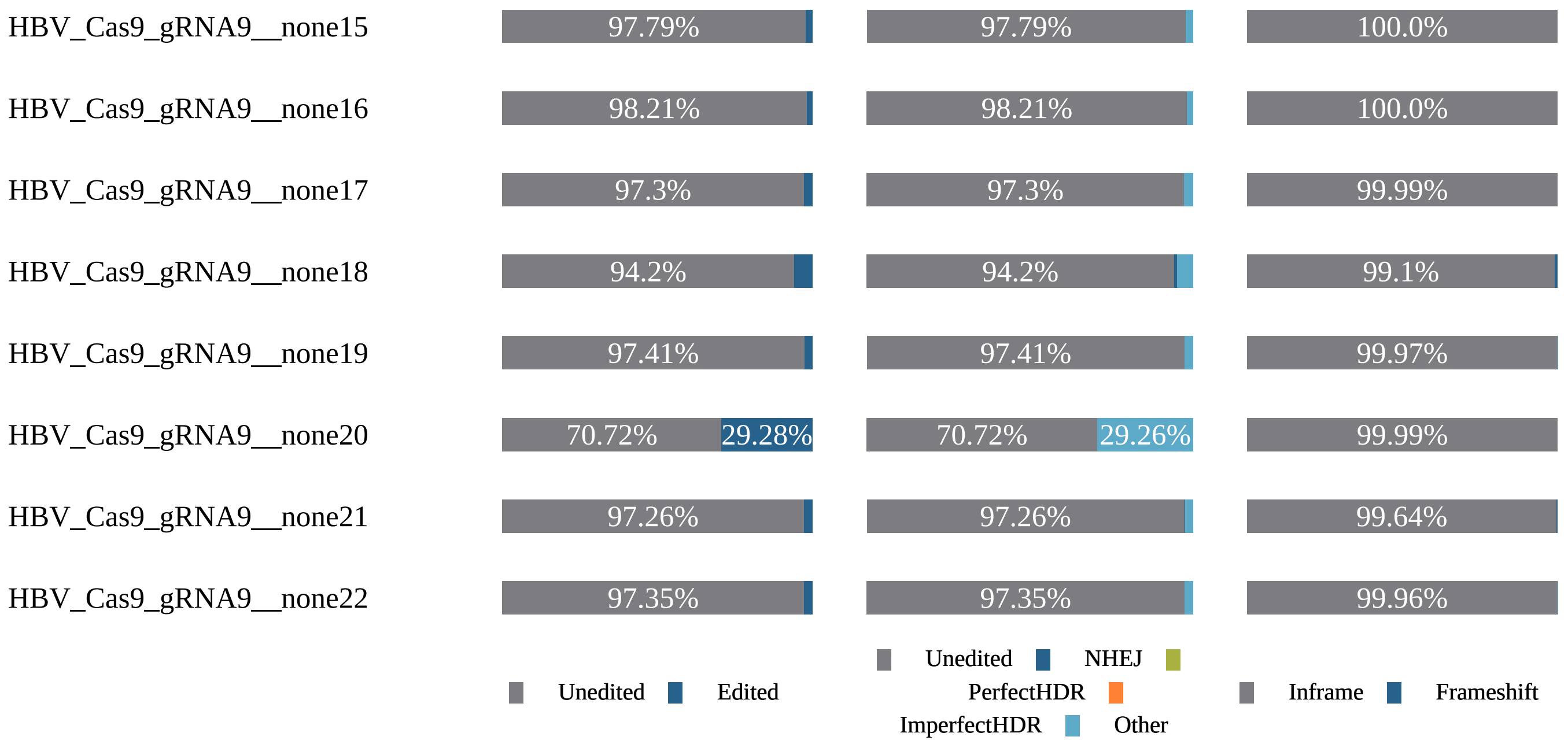

Insertions

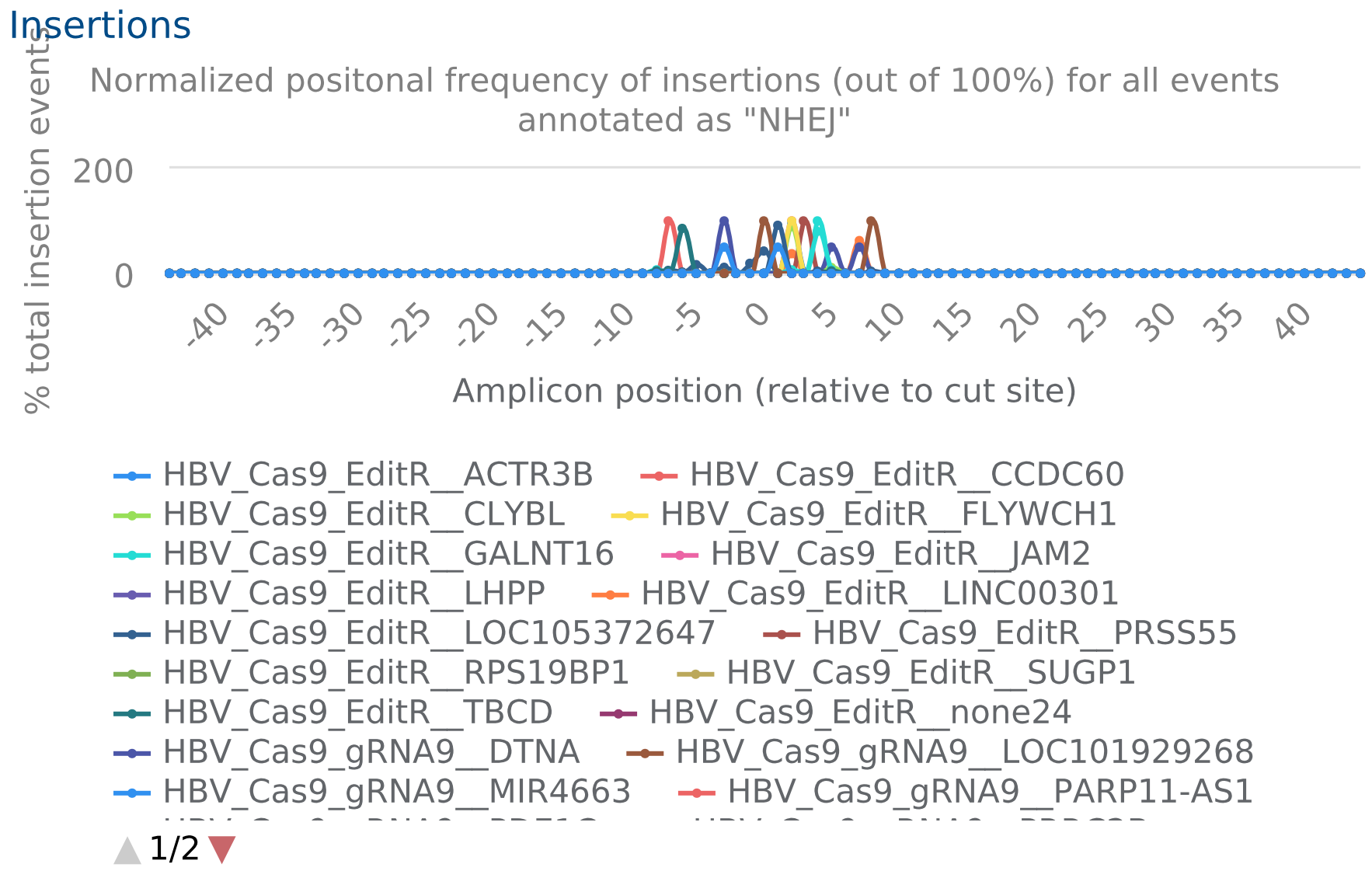

Deletions

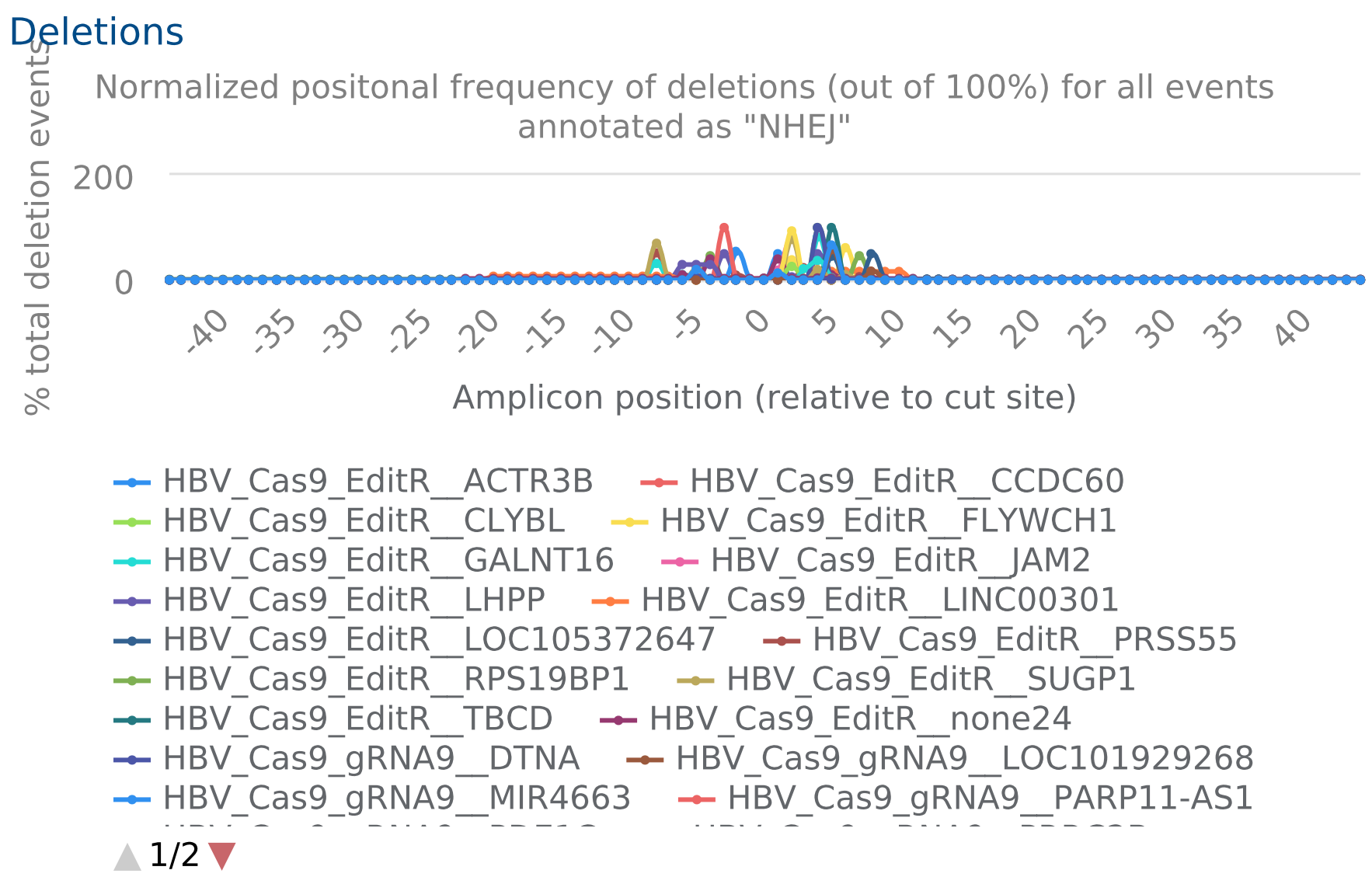

Indel Frequencies

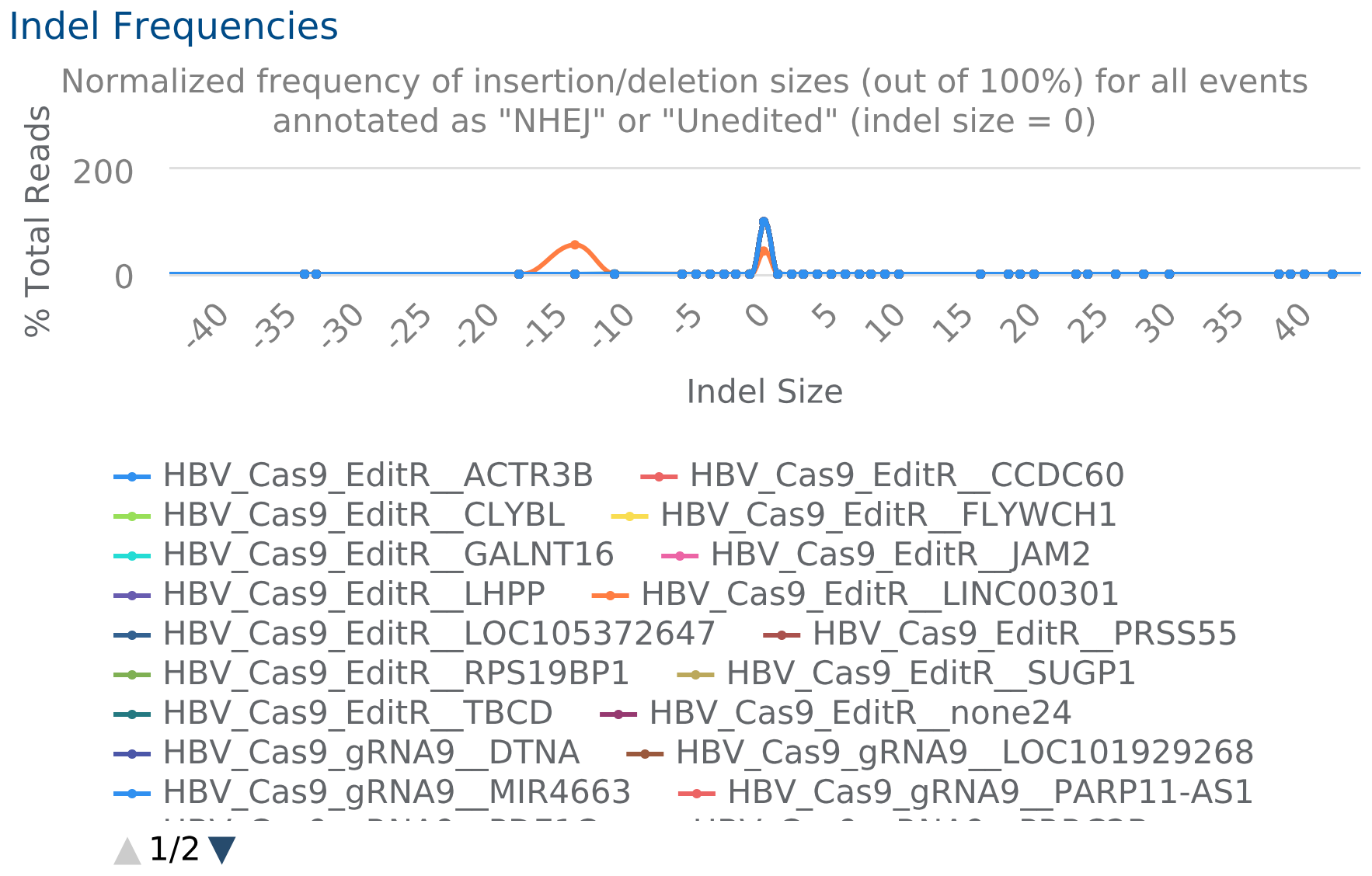

SNP Graph

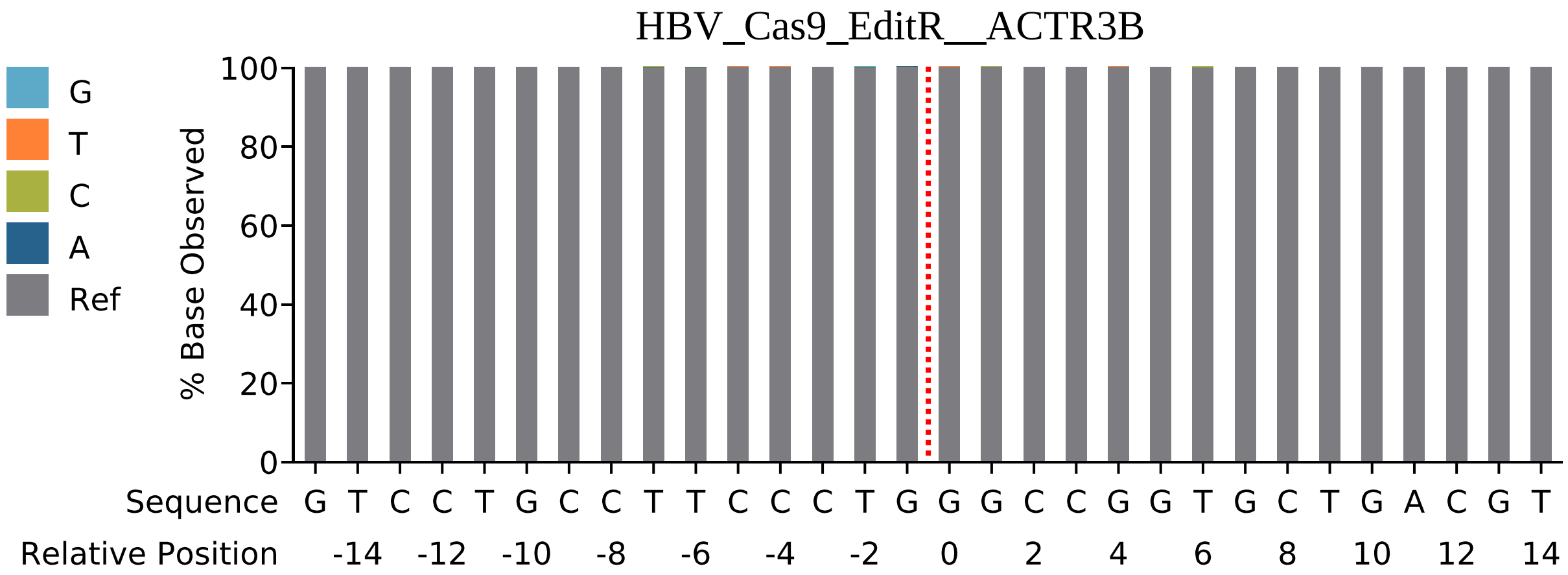

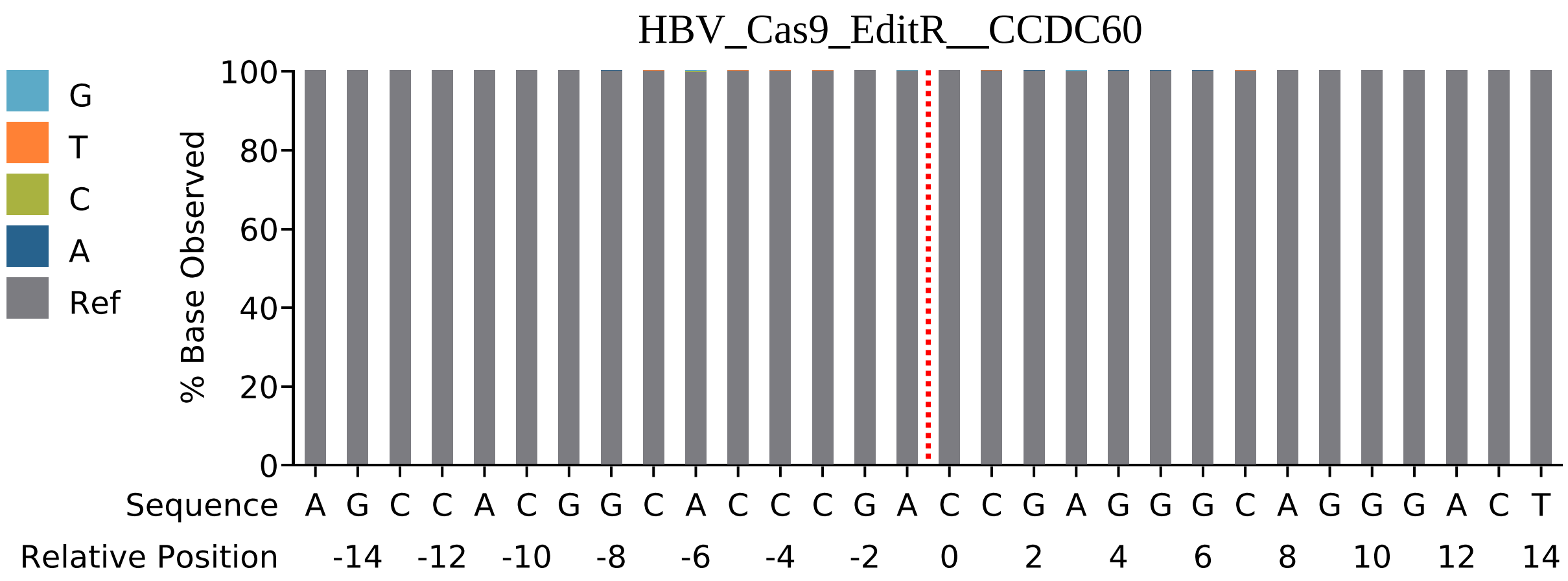

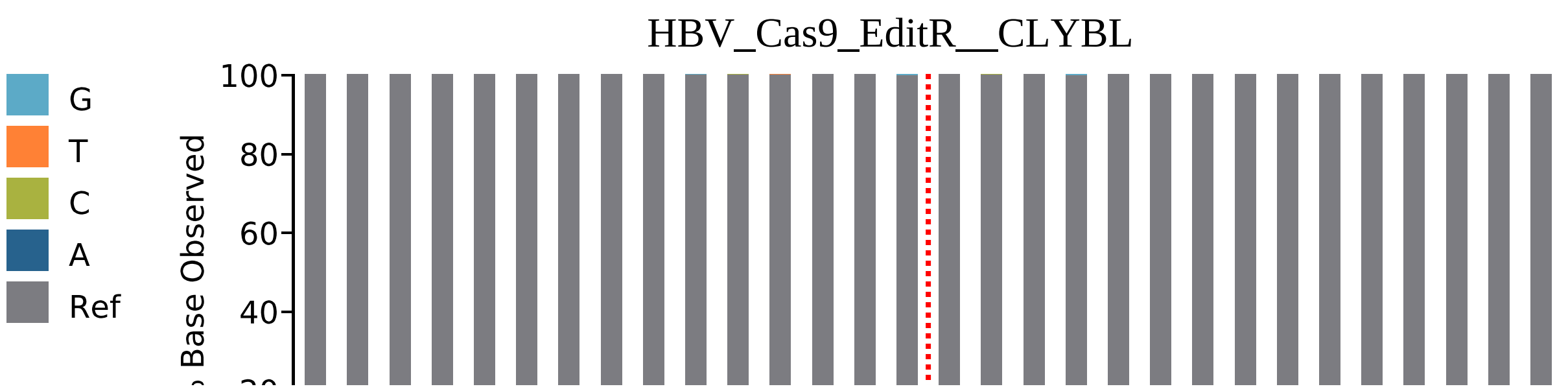

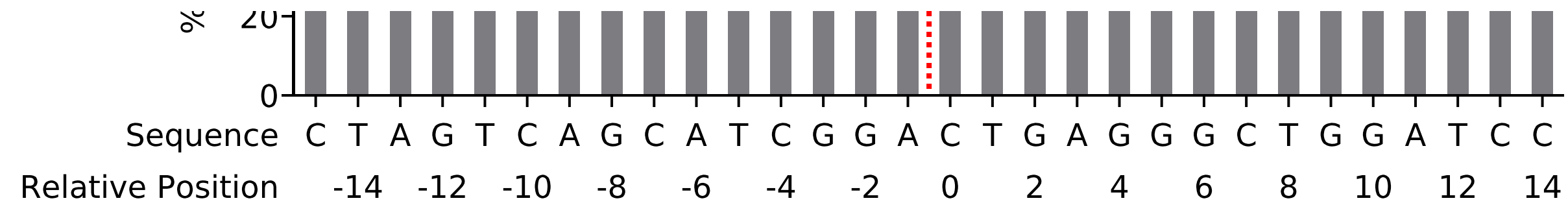

HBV_Cas9_EditR__FLYWCH1

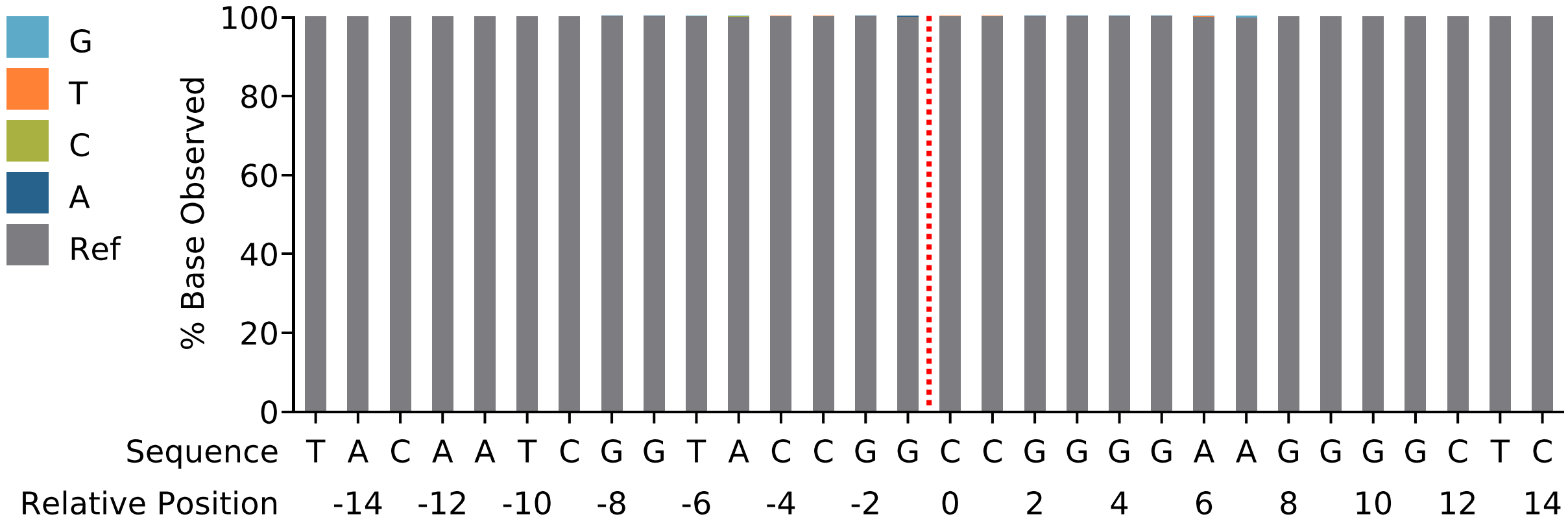

HBV_Cas9_EditR__GALNT16

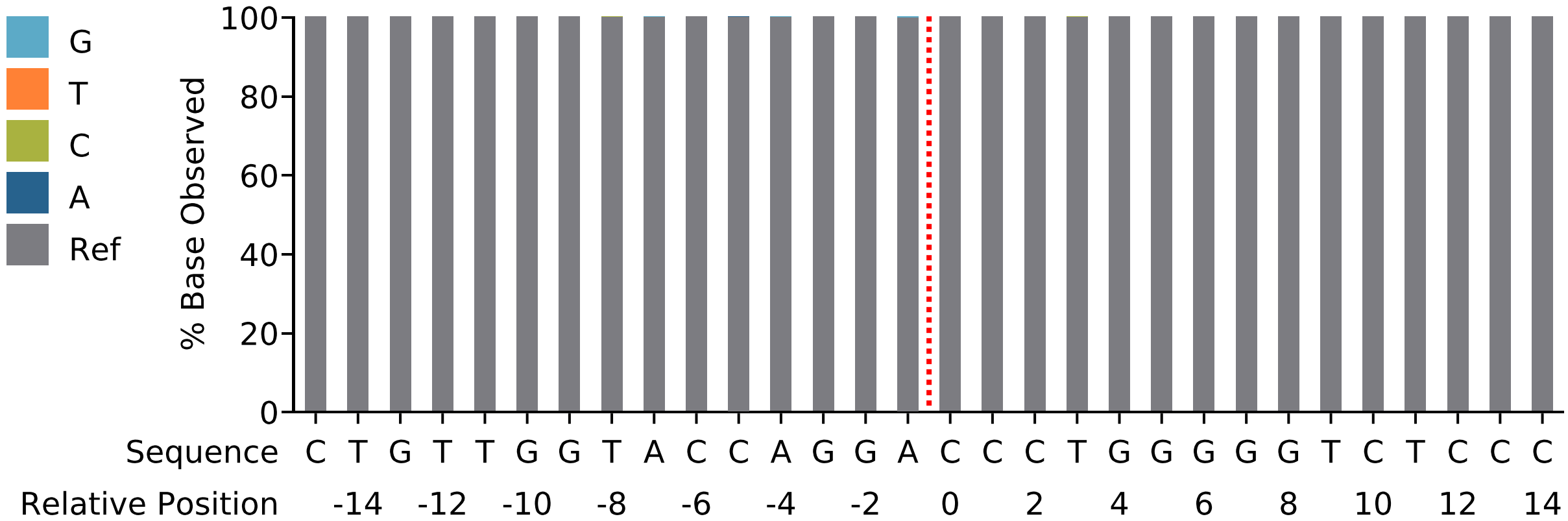

HBV_Cas9_EditR__JAM2

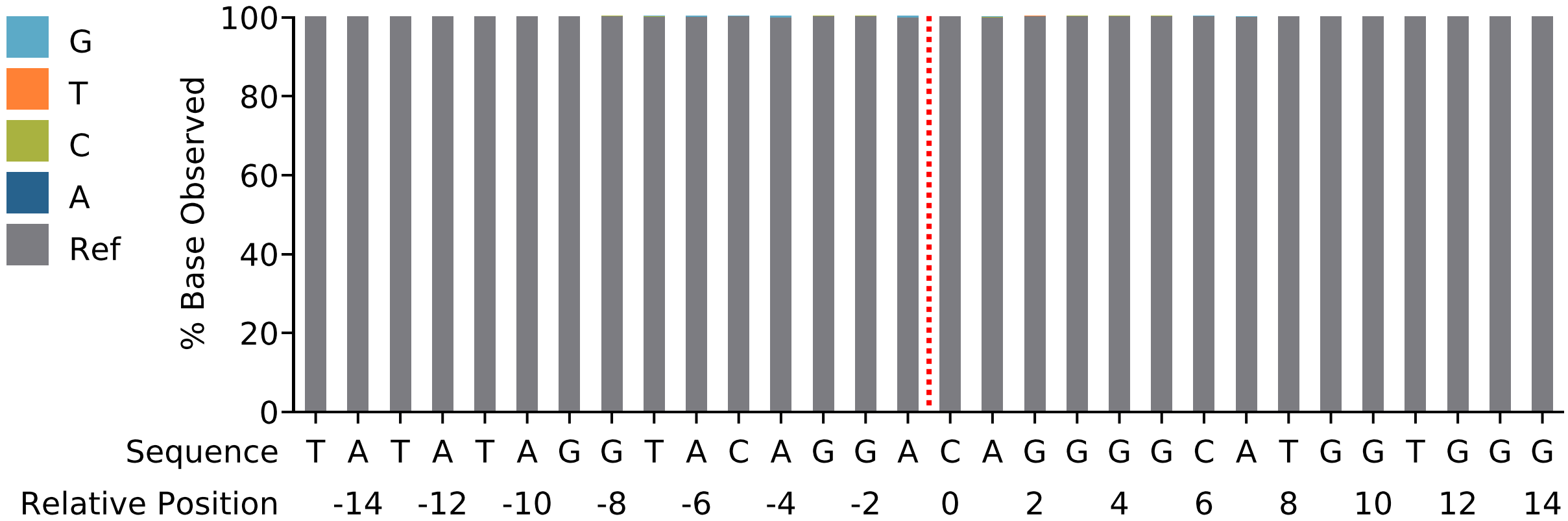

HBV_Cas9_EditR__LHPP

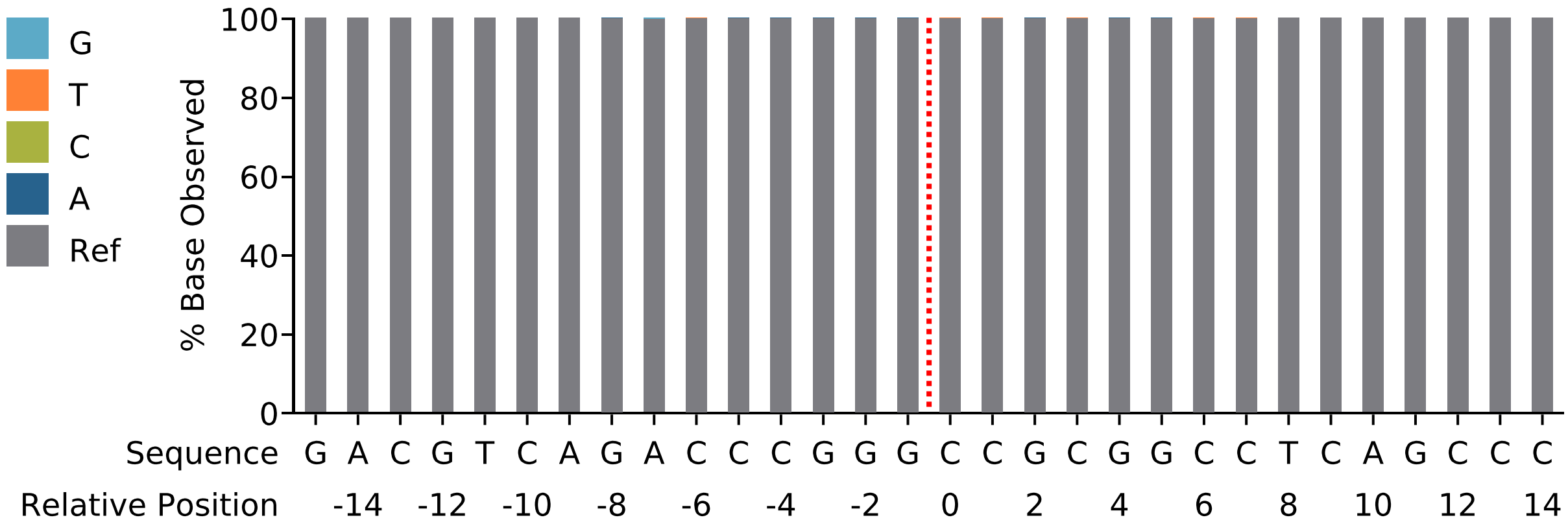

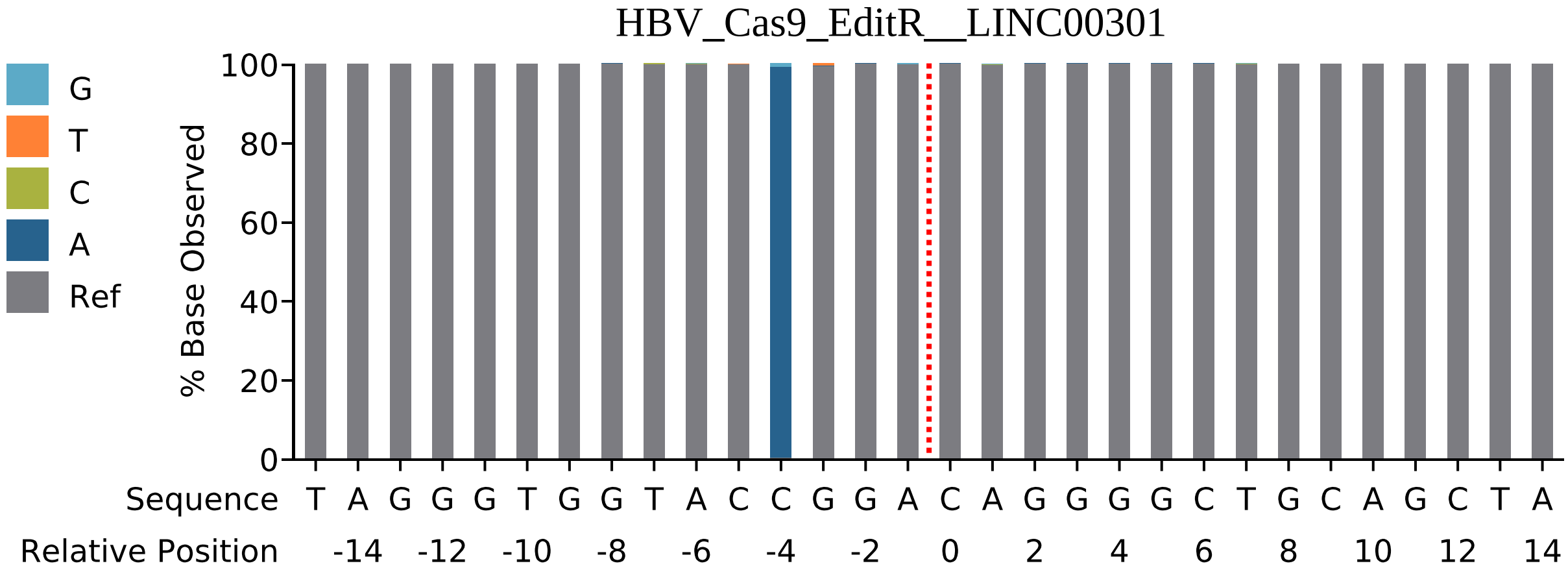
HBV_Cas9_EditR__LINC00301
G
T
C
A
Ref
% Base Observed
100 80 60 40 20 0
Sequence T A G G G T G G T A C C G G A C A G G G G C T G C A G C T A
Relative Position -14 -12 -10 -8 -6 -4 -2 0 2 4 6 8 10 12 14

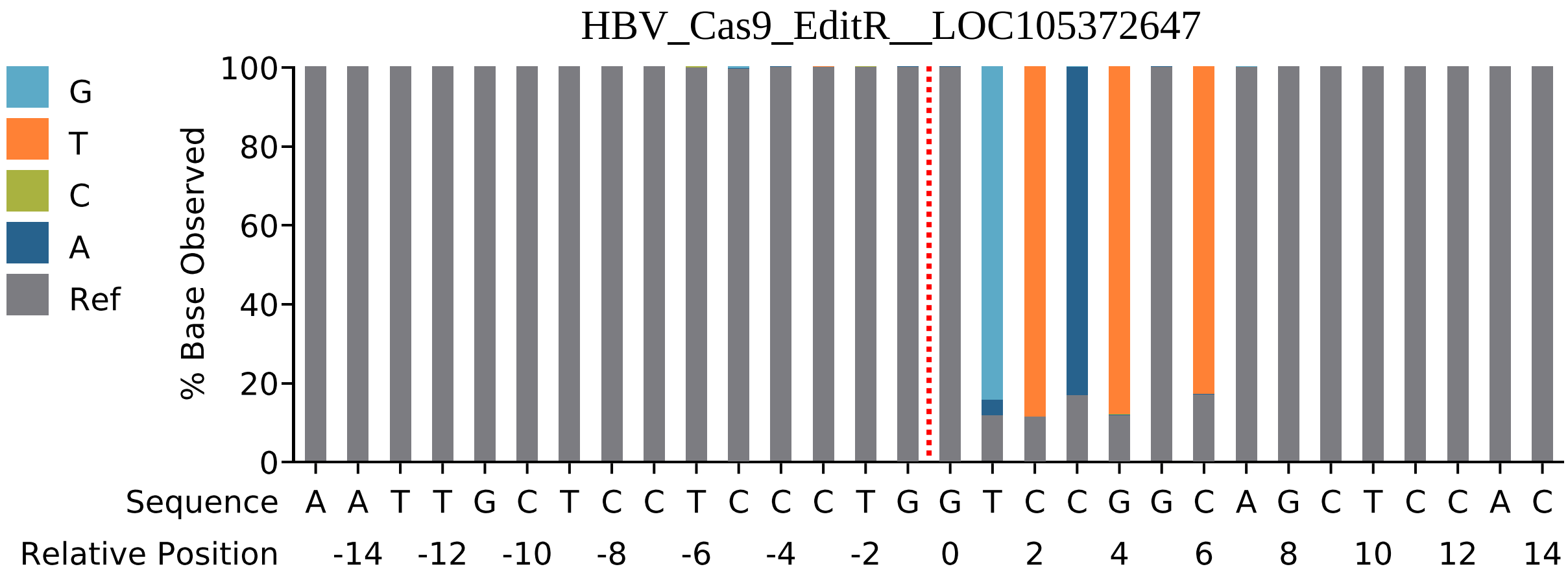
HBV_Cas9_EditR__LOC105372647
G
T
C
A
Ref
% Base Observed
100 80 60 40 20 0
Sequence A A T T G C T C C T C C C T G G T C C G G C A G C T C C A C
Relative Position -14 -12 -10 -8 -6 -4 -2 0 2 4 6 8 10 12 14

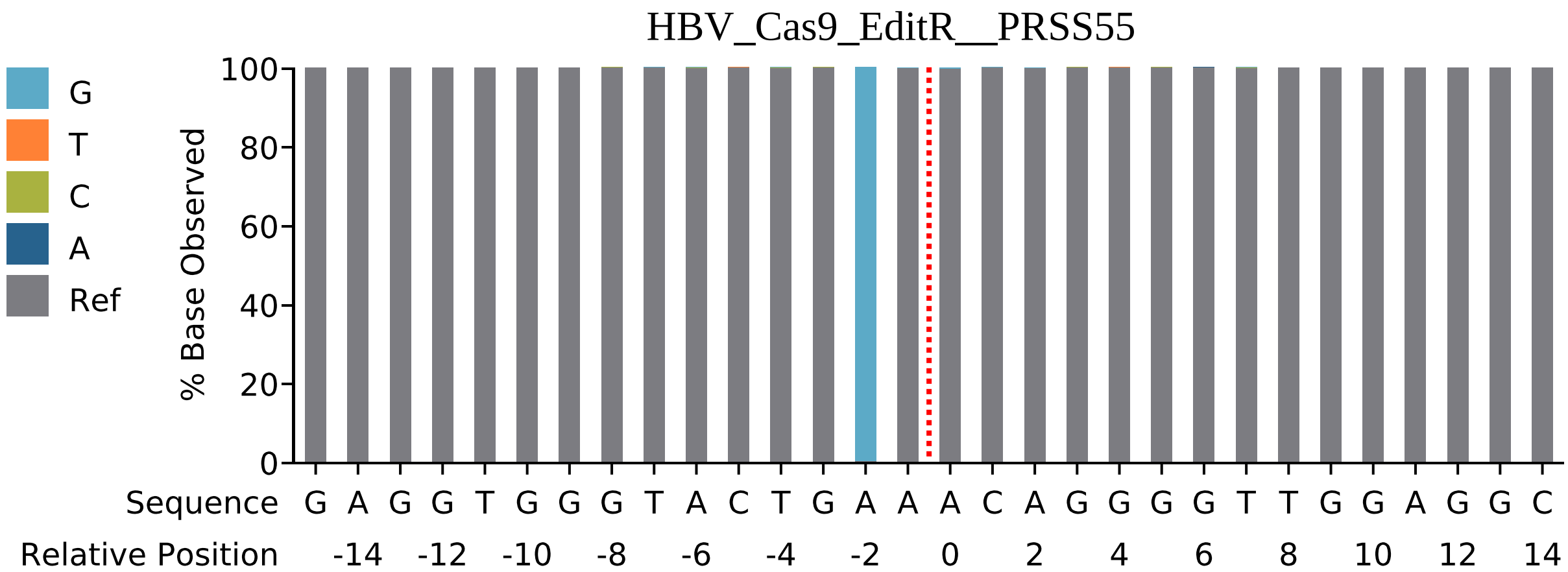
HBV_Cas9_EditR__PRSS55
G
T
C
A
Ref
% Base Observed
100 80 60 40 20 0
Sequence G A G G T G G G T A C T G A A A C A G G G G T T G G A G G C
Relative Position -14 -12 -10 -8 -6 -4 -2 0 2 4 6 8 10 12 14

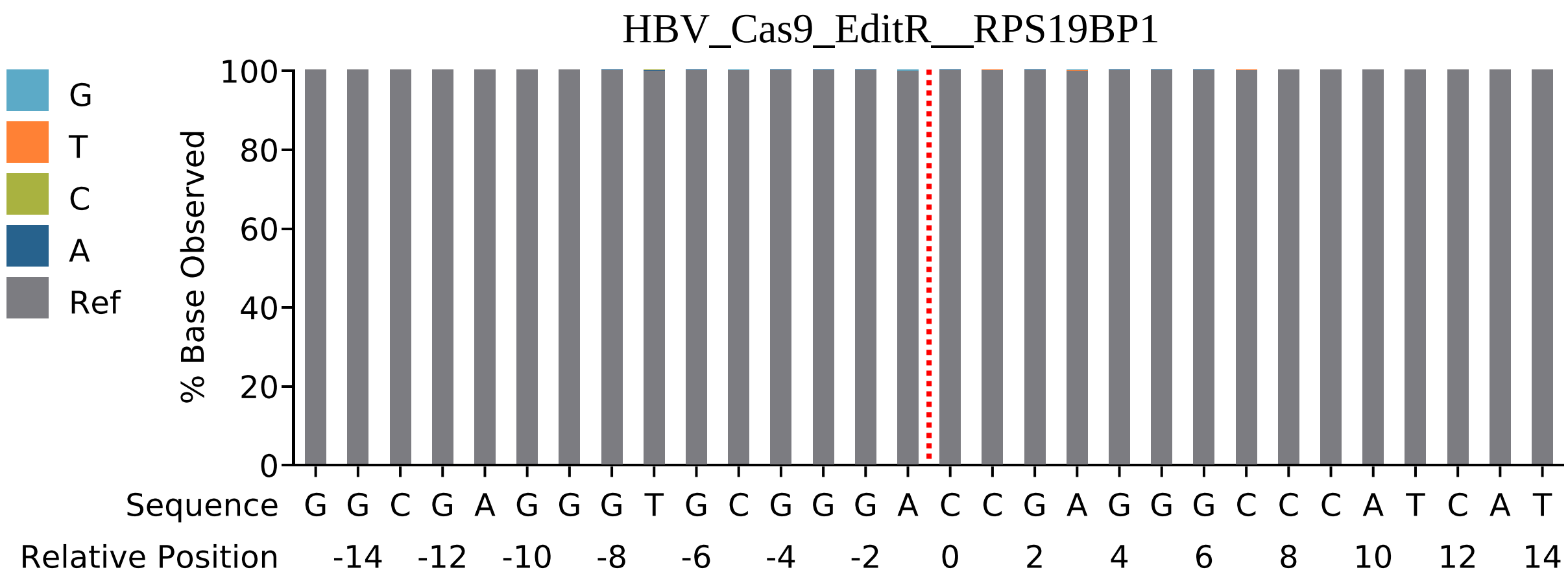
HBV_Cas9_EditR__RPS19BP1
G
T
C
A
Ref
% Base Observed
100 80 60 40 20 0
Sequence G G C G A G G G T G C G G G A C C G A G G G C C C A T C A T
Relative Position -14 -12 -10 -8 -6 -4 -2 0 2 4 6 8 10 12 14

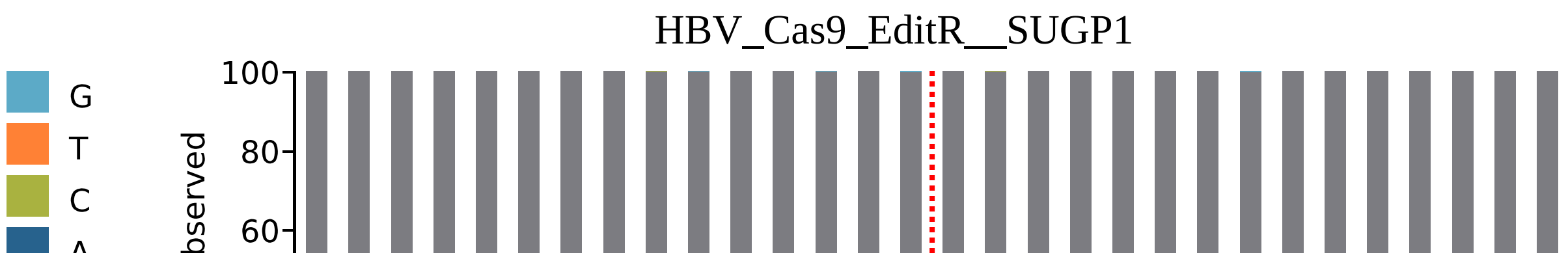
HBV_Cas9_EditR__SUGP1
G
T
C
100 80 60

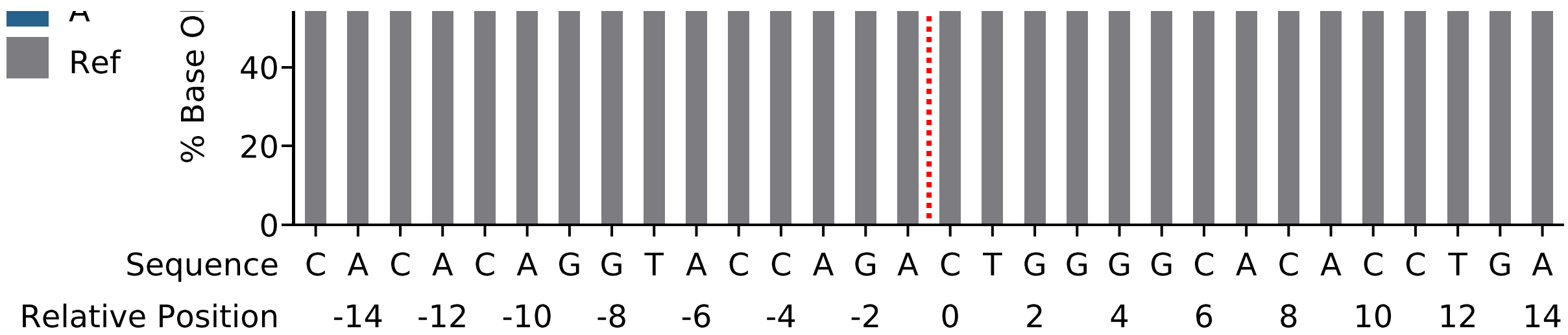

HBV_Cas9_EditR__TBCD

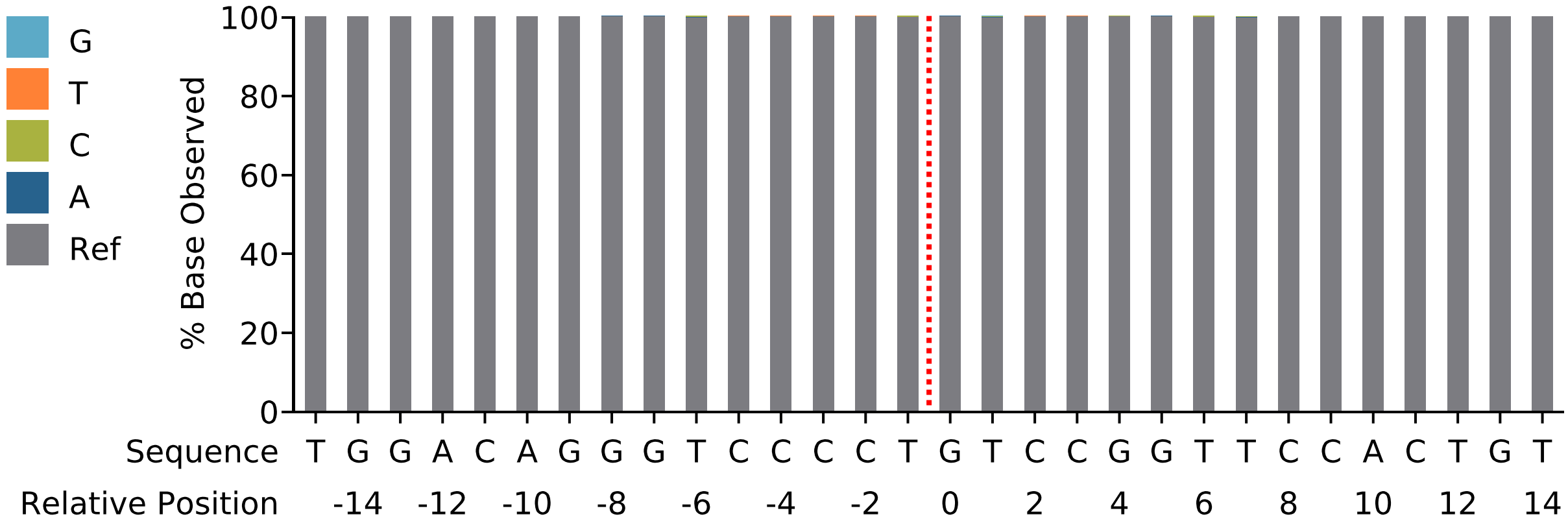

HBV_Cas9_EditR__none24

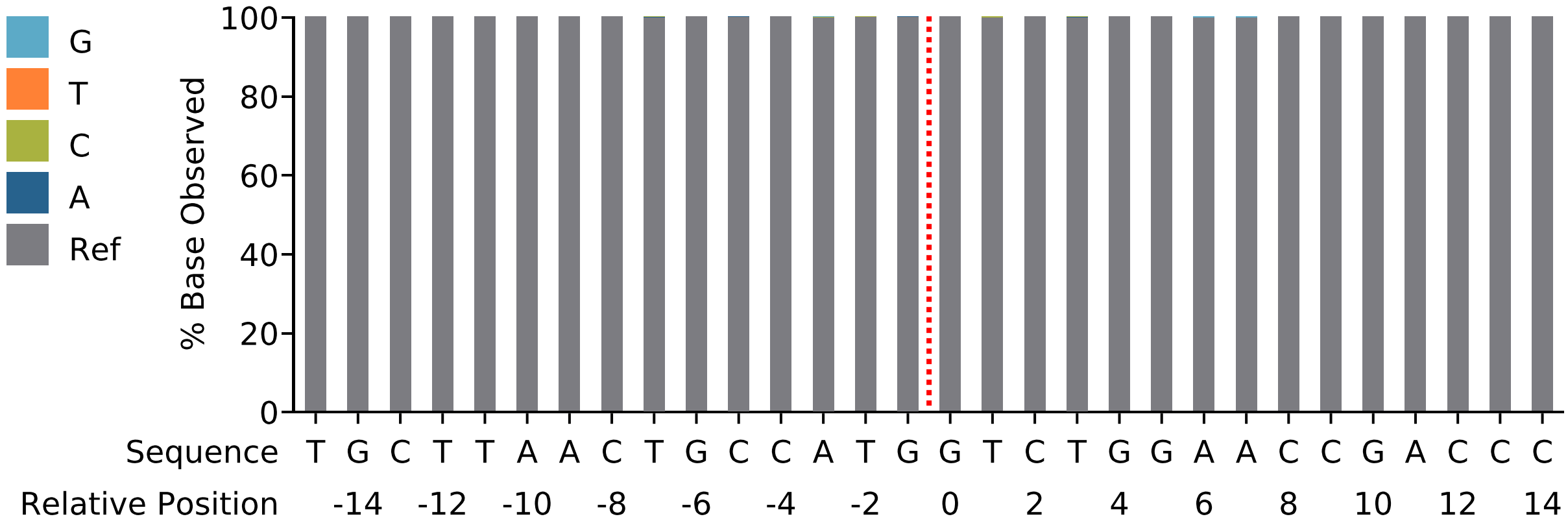

HBV_Cas9_gRNA9__DTNA

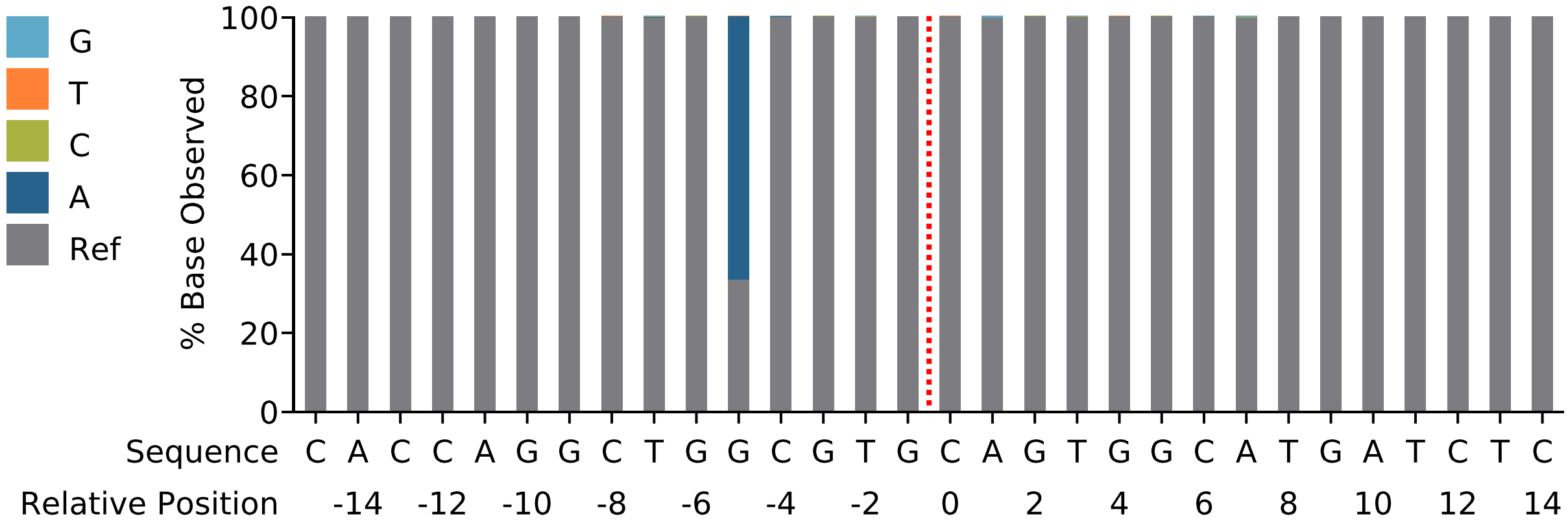

HBV_Cas9_gRNA9__LOC101929268

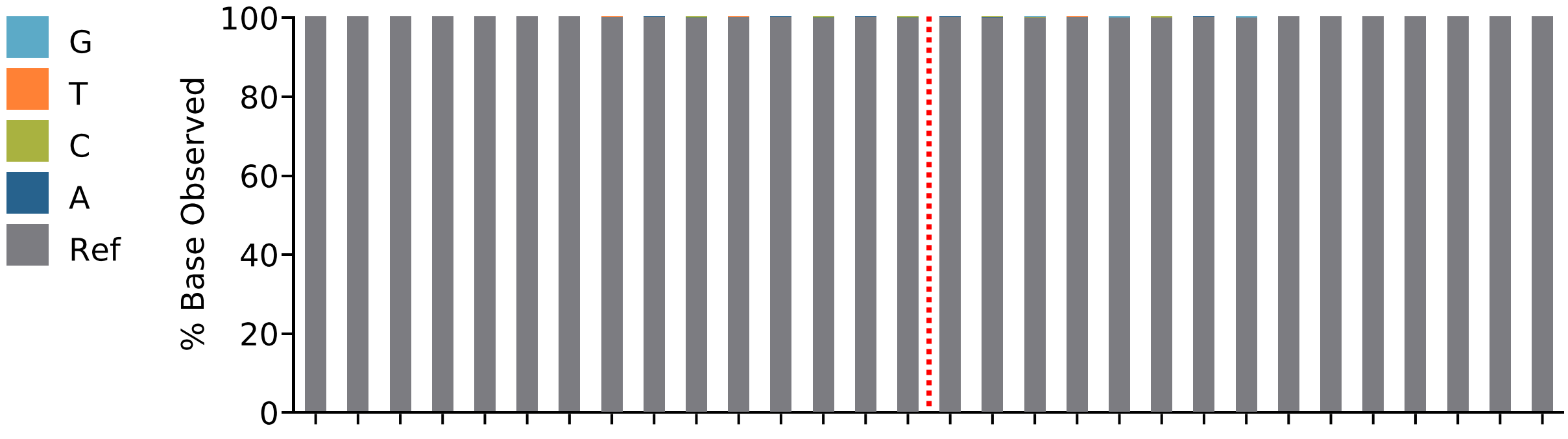

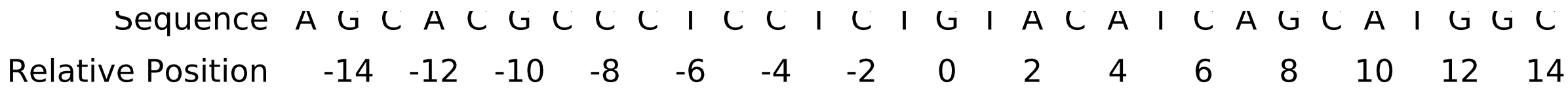

HBV_Cas9_gRNA9__MIR4663

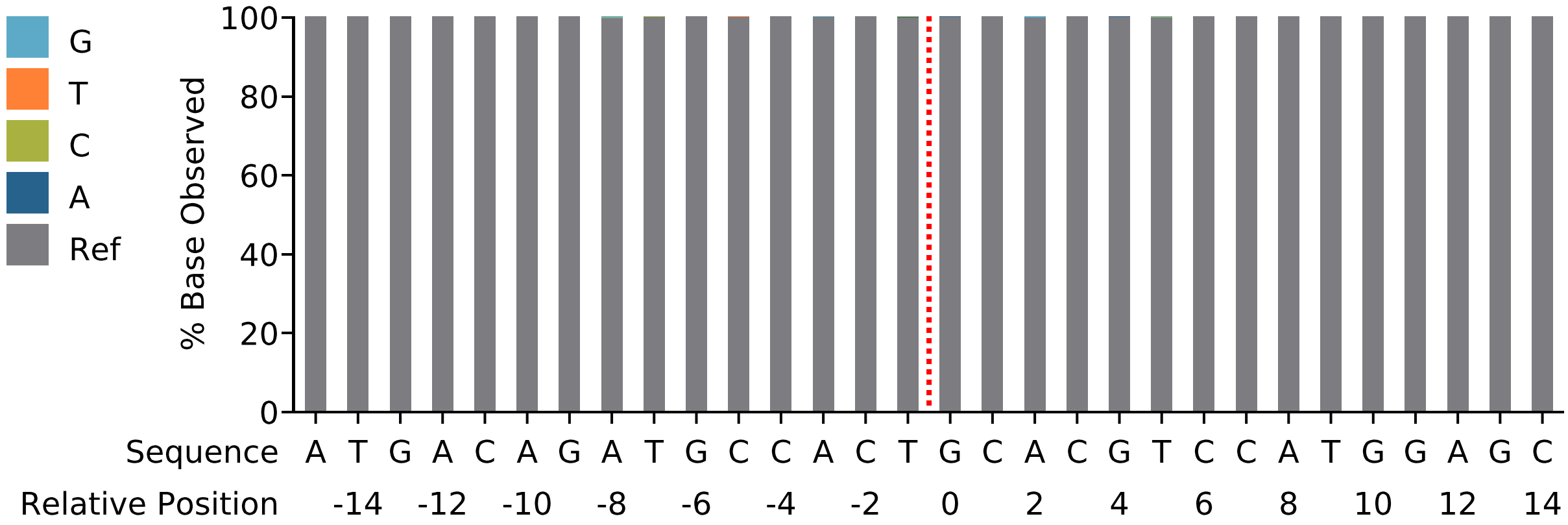

HBV_Cas9_gRNA9__PARP11-AS1

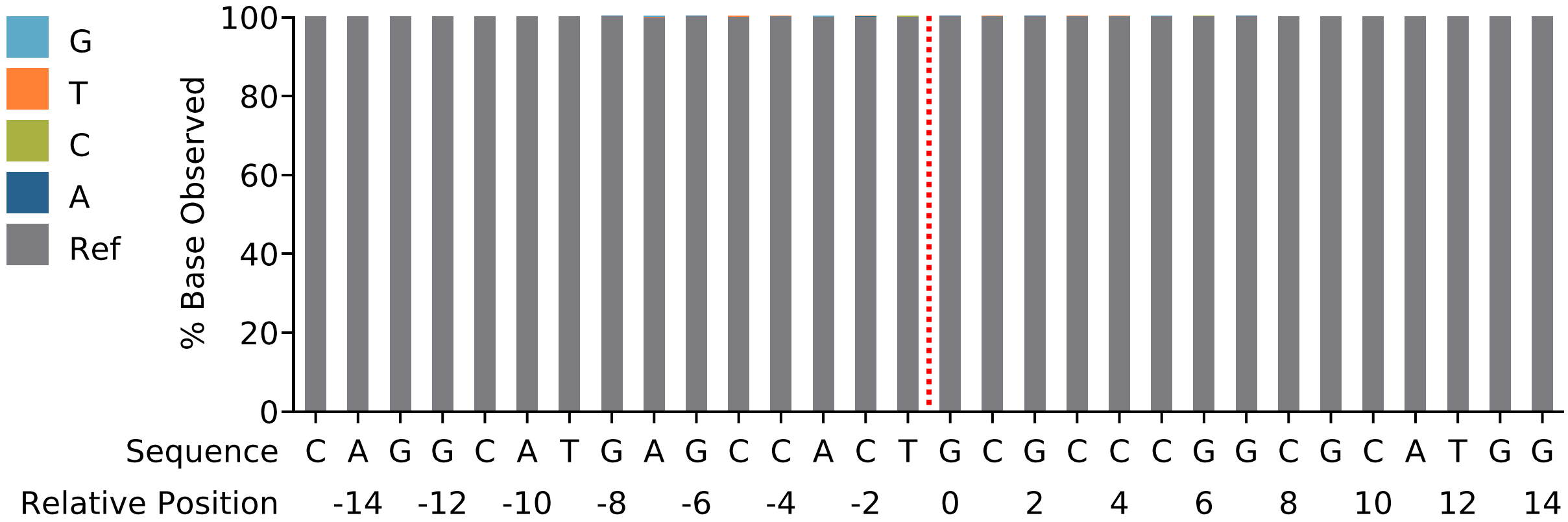

HBV_Cas9_gRNA9__PDE1C

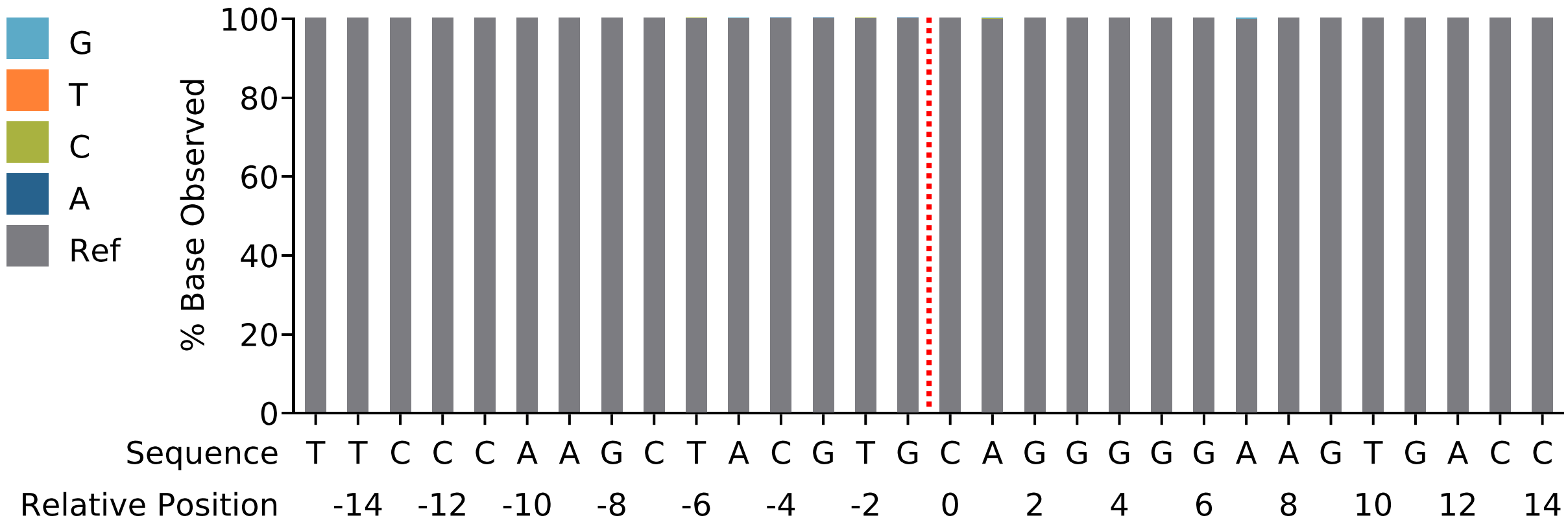

HBV_Cas9_gRNA9__PRRC2B

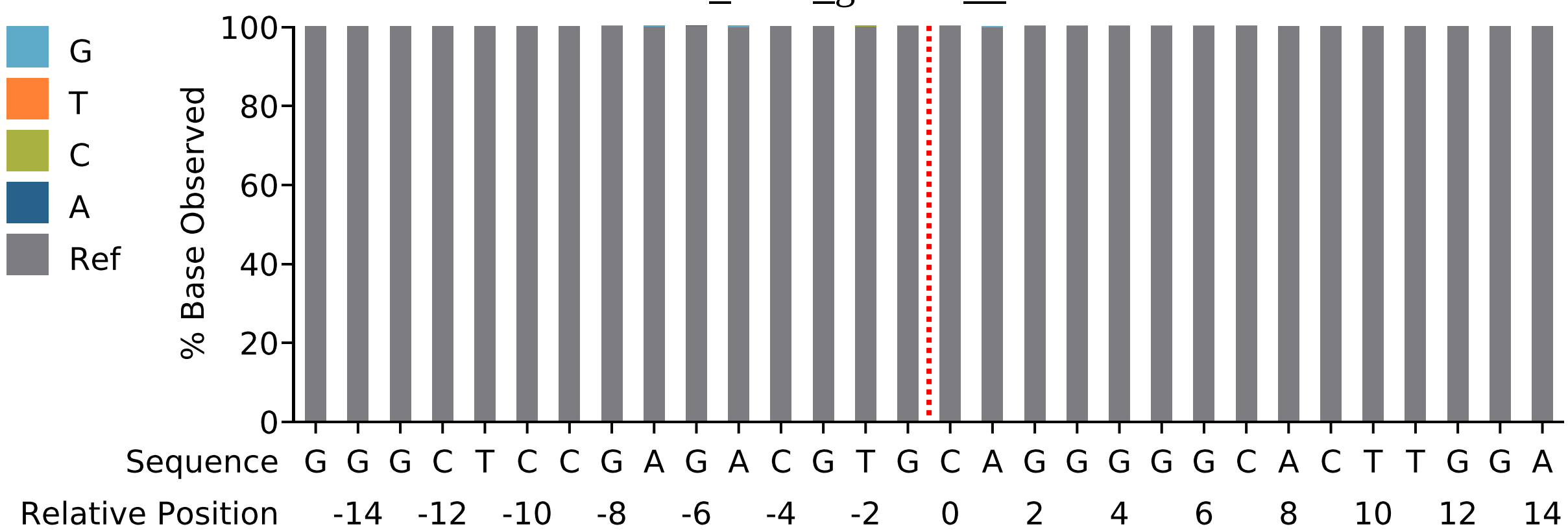

HBV_Cas9_gRNA9__SHISAL1

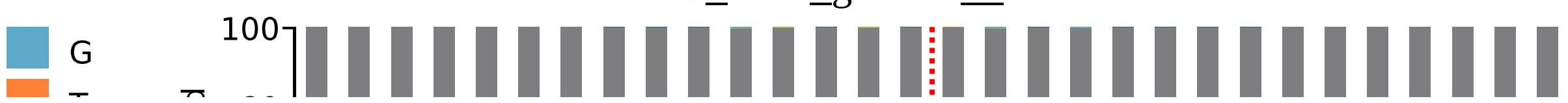

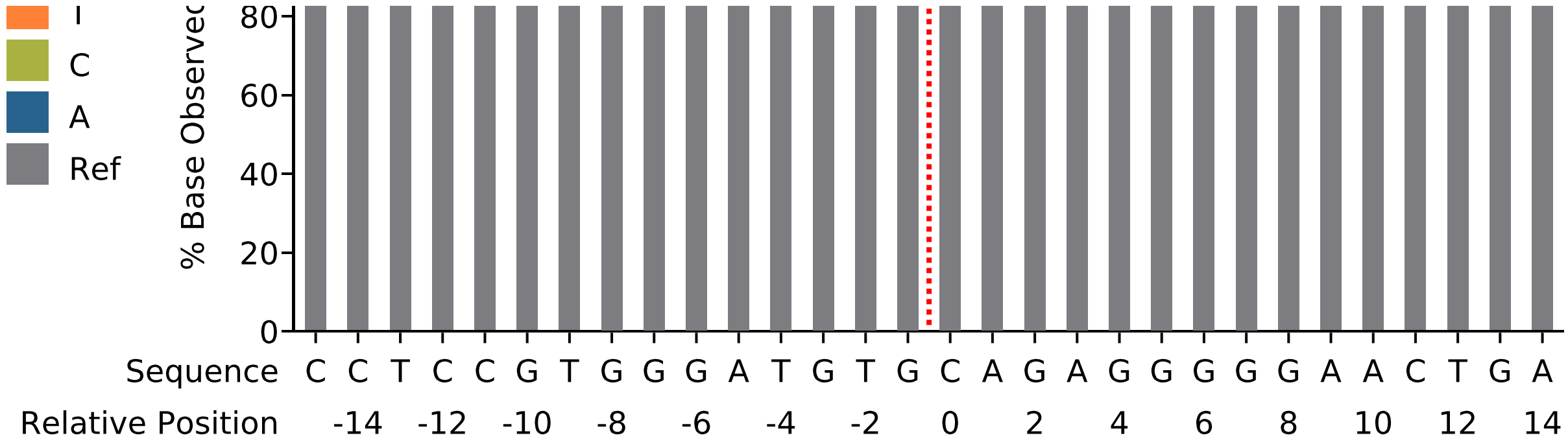

HBV_Cas9_gRNA9__none11

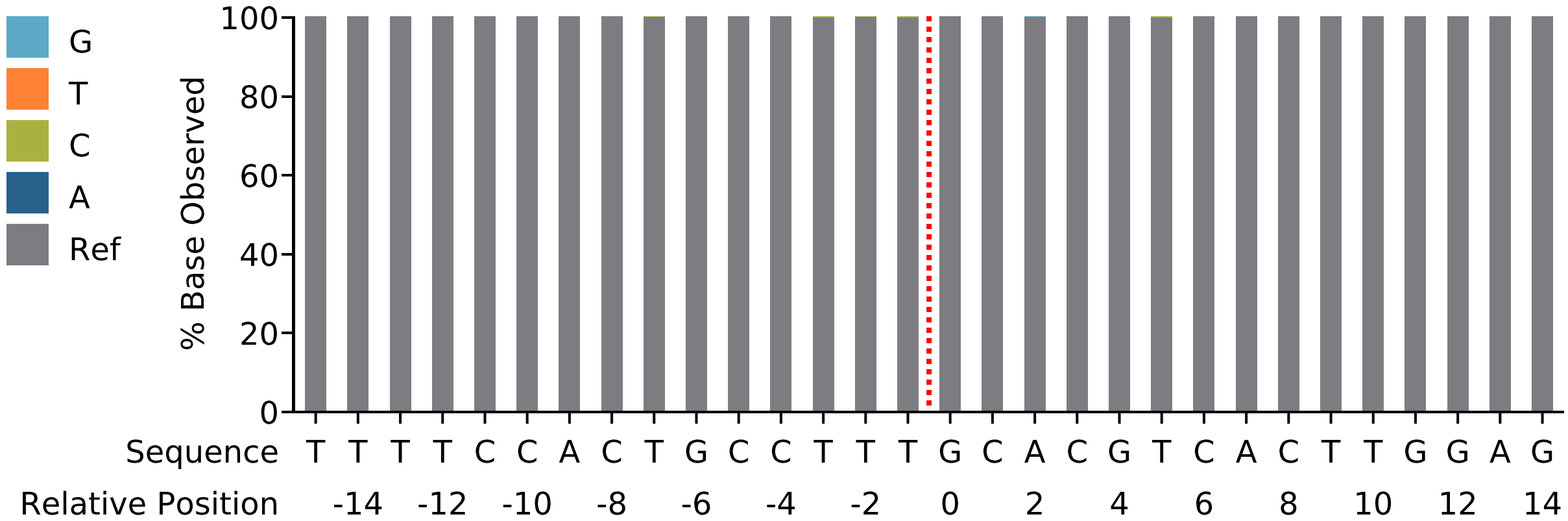

HBV_Cas9_gRNA9__none12

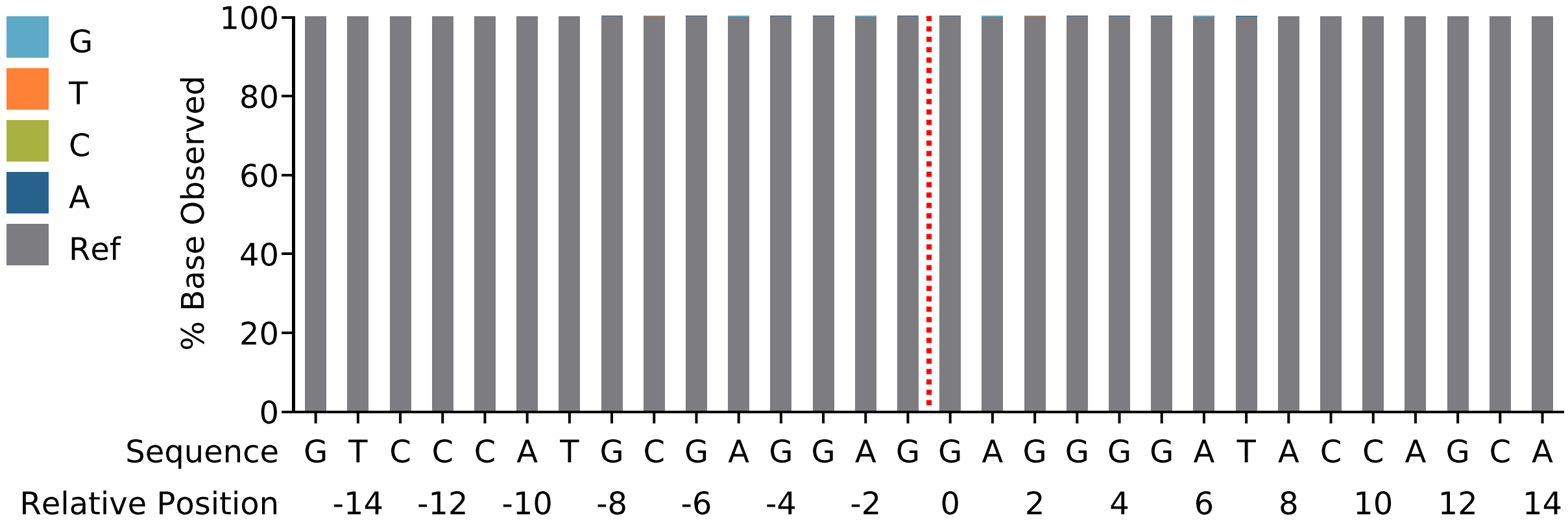

HBV_Cas9_gRNA9__none13

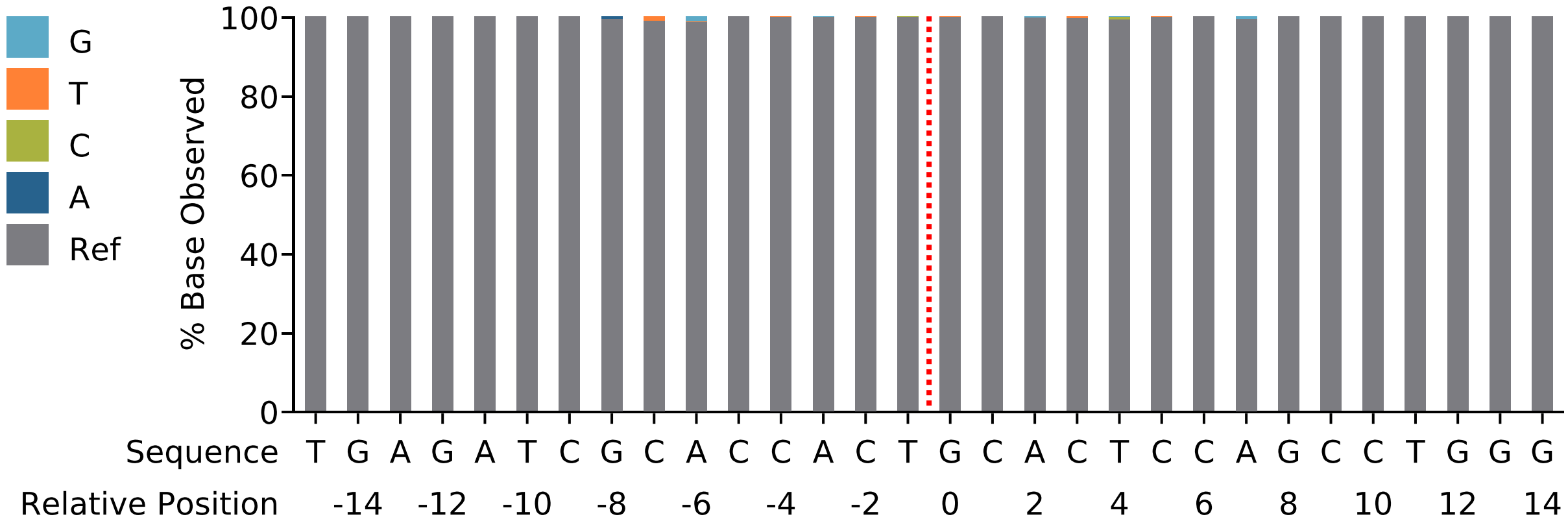

HBV_Cas9_gRNA9__none14

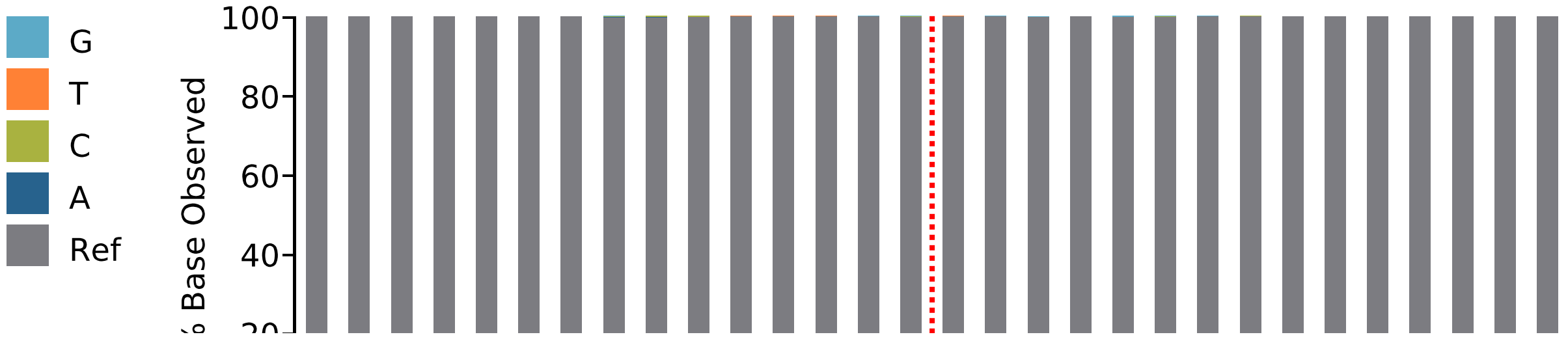

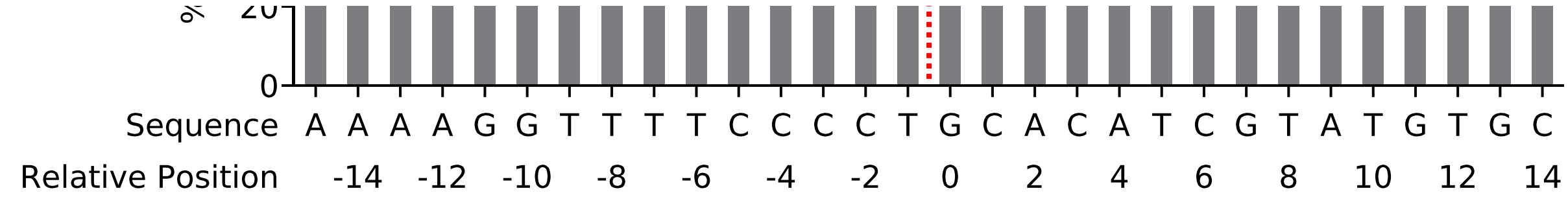

HBV_Cas9_gRNA9__none15

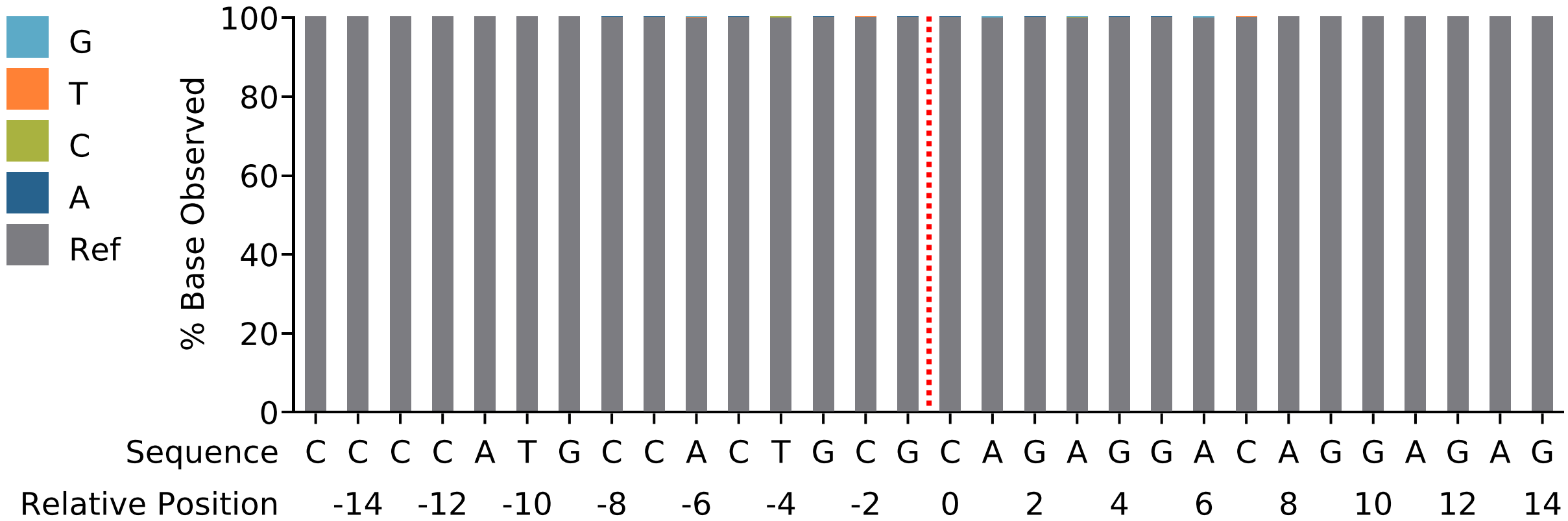

HBV_Cas9_gRNA9__none16

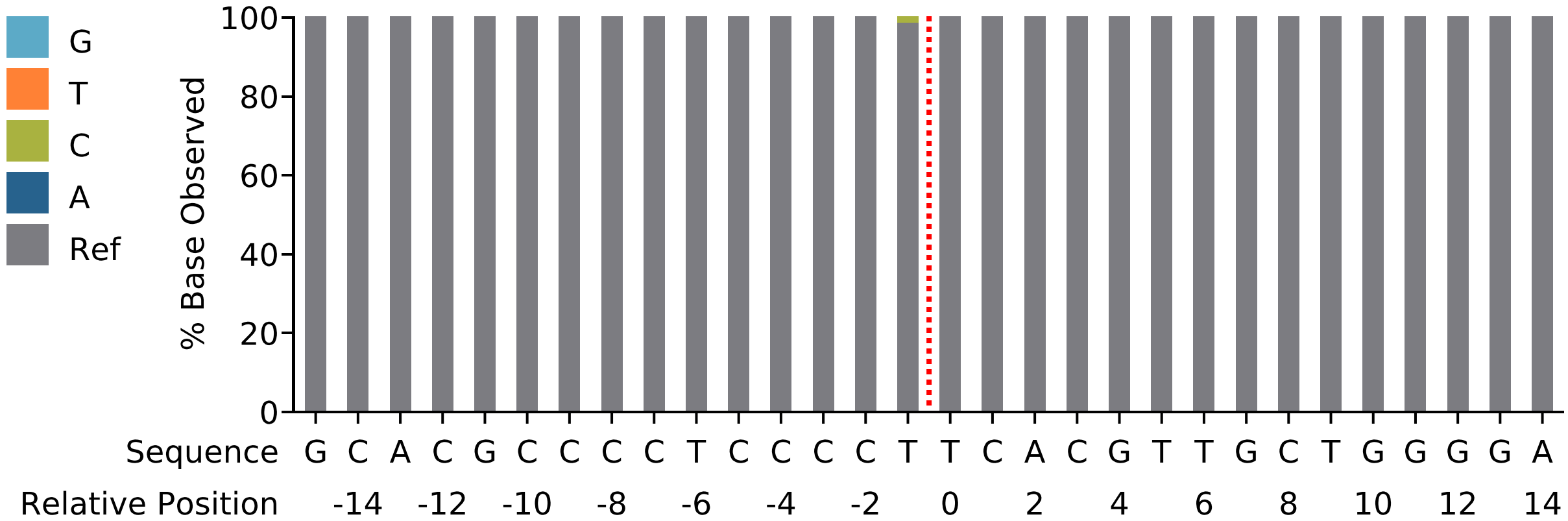

HBV_Cas9_gRNA9__none17

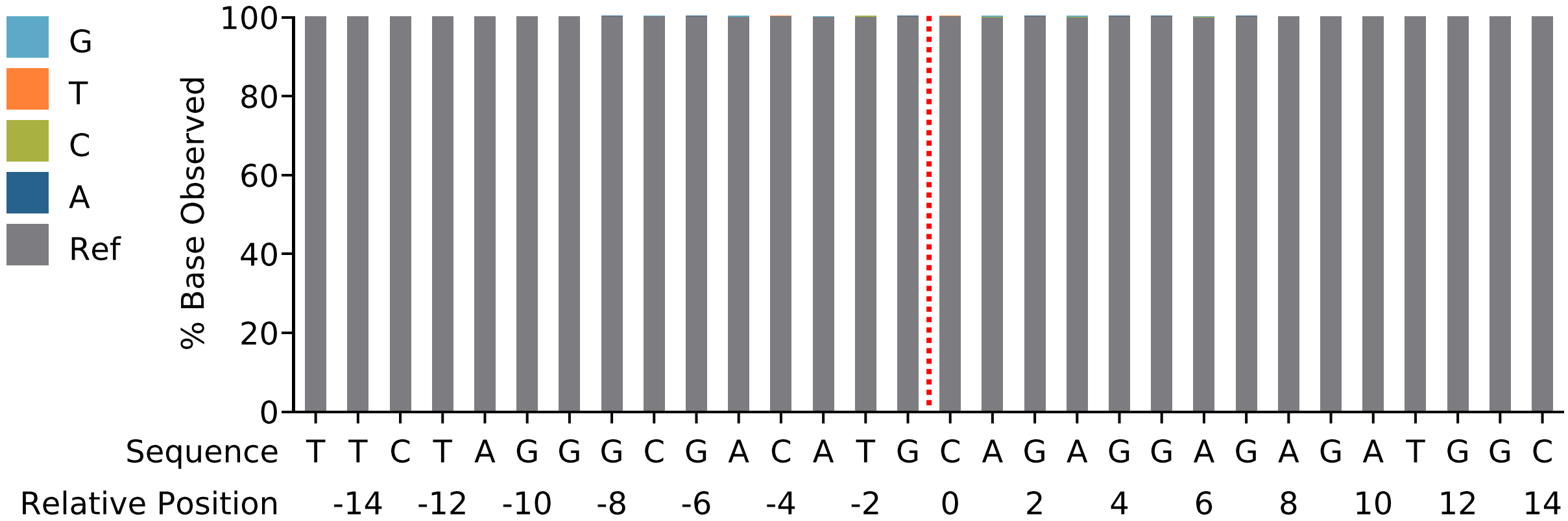

HBV_Cas9_gRNA9__none18

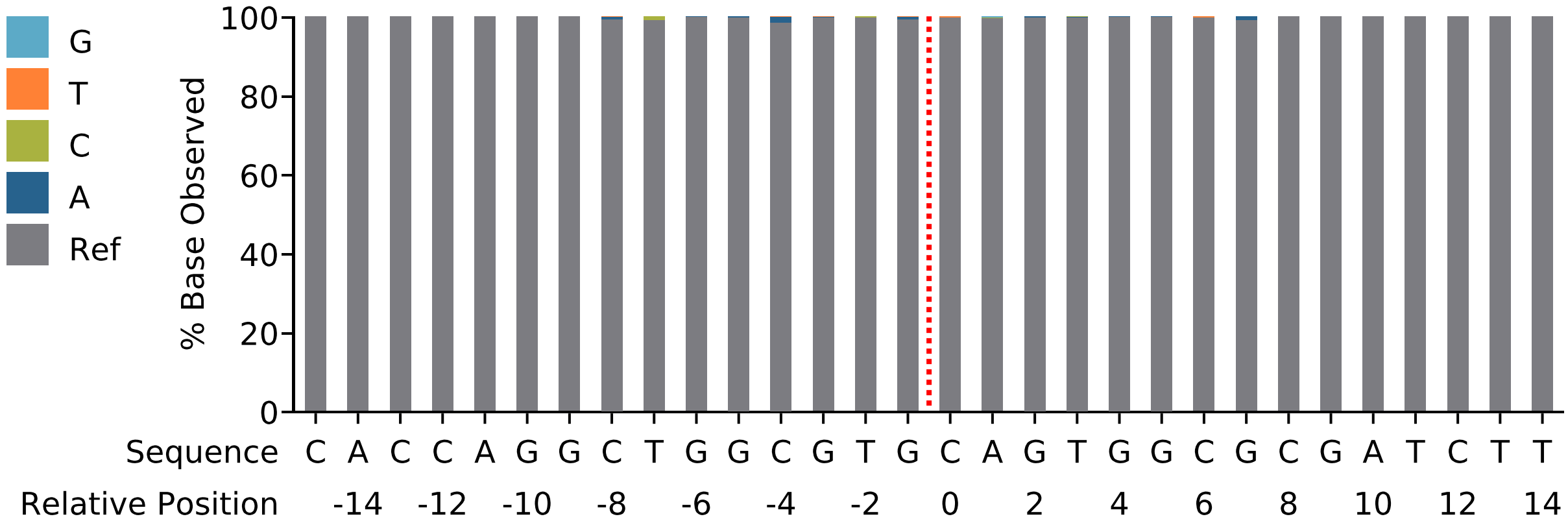

HBV_Cas9_gRNA9__none19

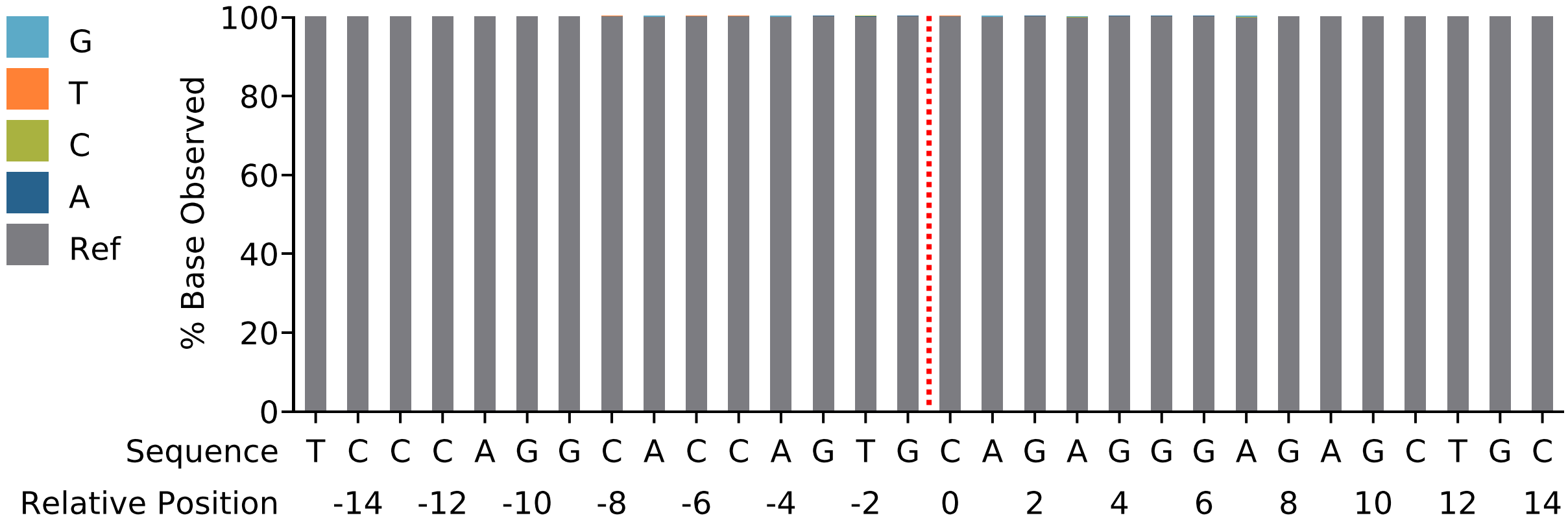

HBV_Cas9_gRNA9__none20

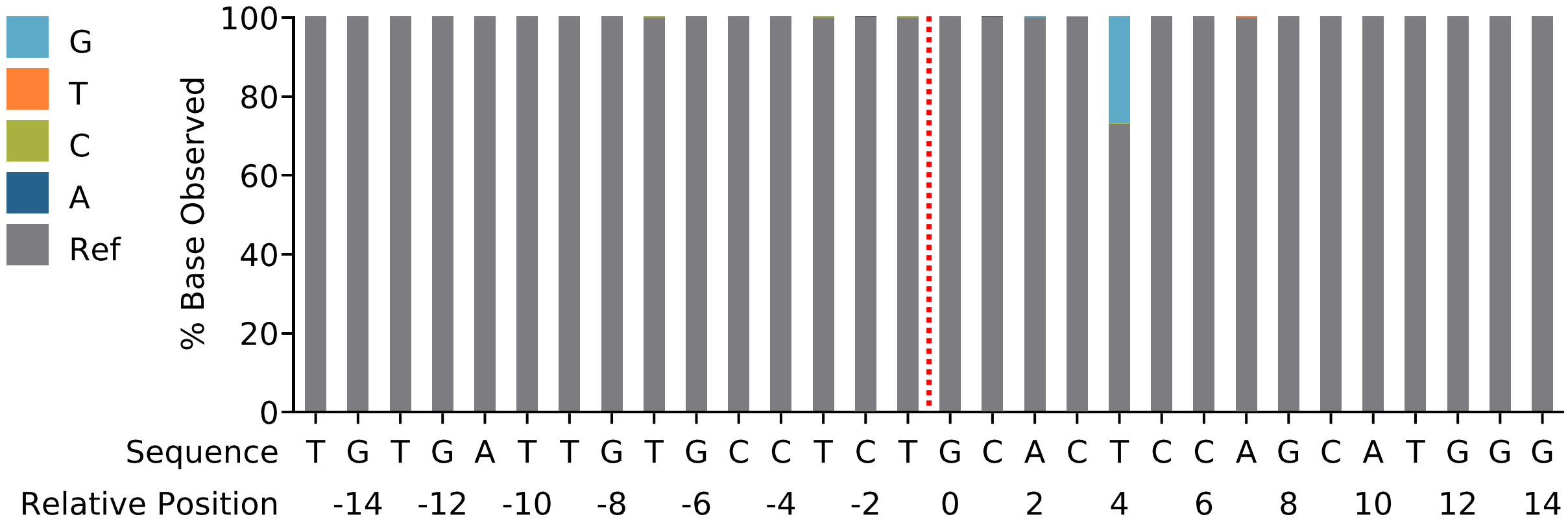

HBV_Cas9_gRNA9__none21

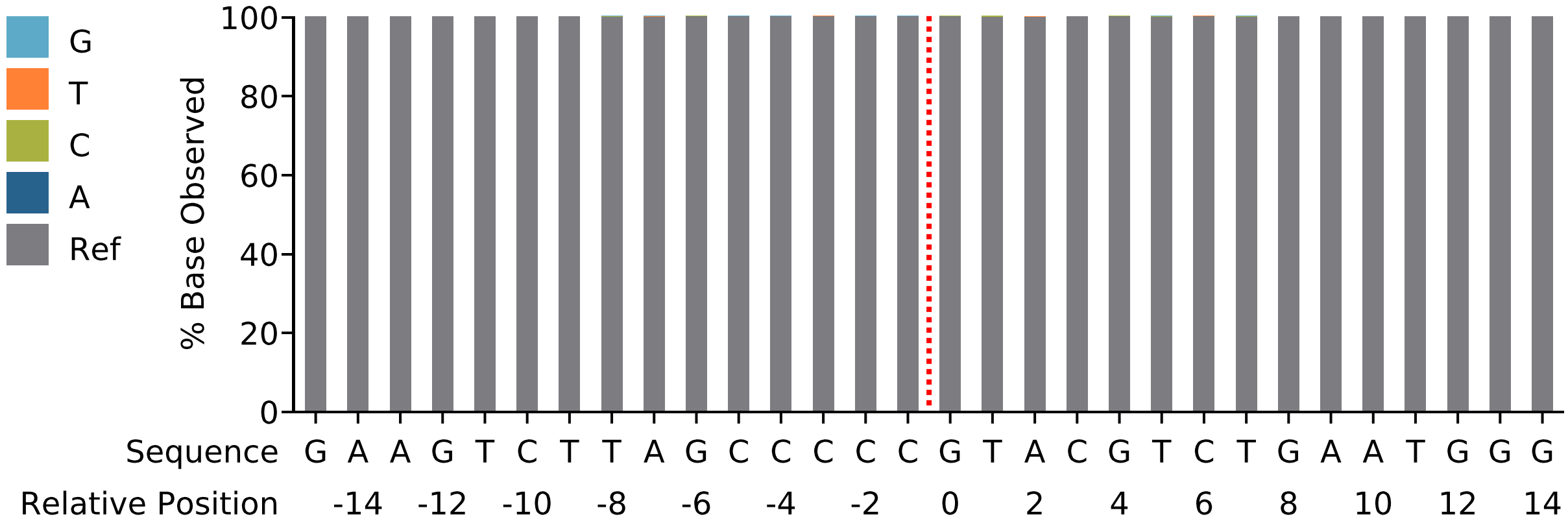

HBV_Cas9_gRNA9__none22

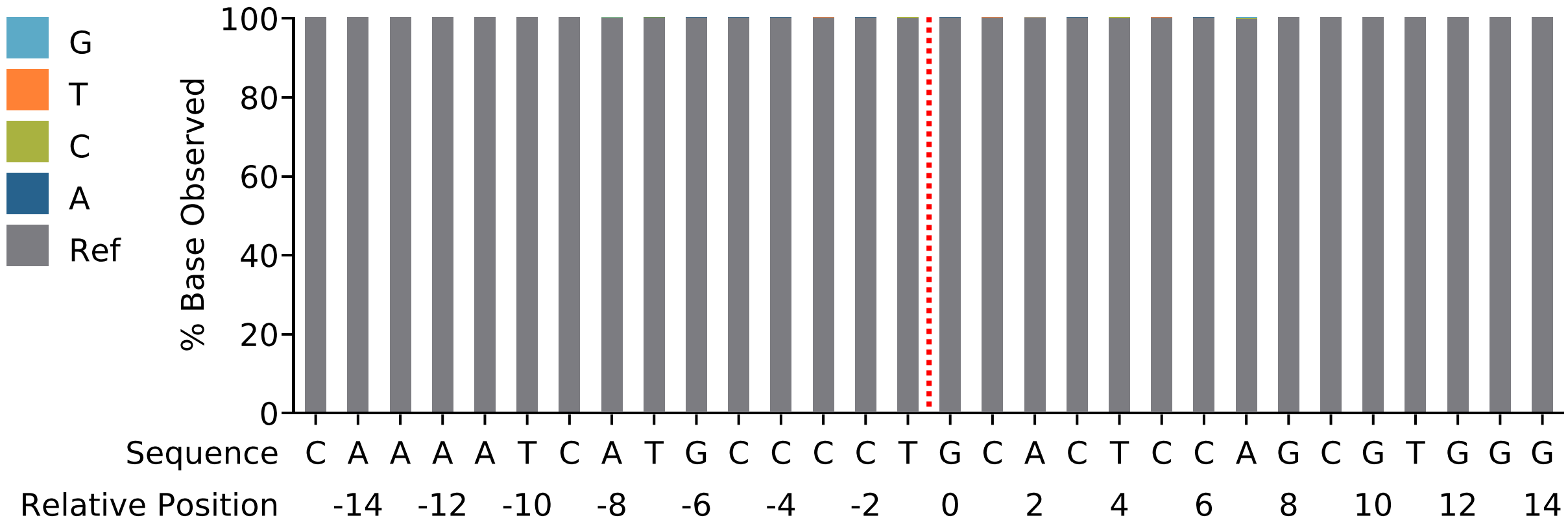

Supplement: Supplementary file 1 [file DataSheet1.pdf]
