# Supplementary material for: Combined RNA- and DNA-targeting CRISPR ribonucleoproteins enhance suppression of HBV transcripts
Source: Front Genome Ed. 2026 Sep 23;8:1824895. doi: 10.3389/fgeed.2026.1824895 (PMC13642003; doi:10.3389/fgeed.2026.1824895)

Analysis mode: CRISPAltRations 1_2_1
Start date: 2026/08/21 15:27
Targets: HBV_Cas12_gRNA4__CD300E, HBV_Cas12_gRNA4__CHKB-DT, HBV_Cas12_gRNA4__GNA12, HBV_Cas12_gRNA4__LINC00989, HBV_Cas12_gRNA4__LOC107986930, HBV_Cas12_gRNA4__MRGBP, HBV_Cas12_gRNA4__PCNX2, HBV_Cas12_gRNA4__SHROOM2, HBV_Cas12_gRNA4__SYNJ2BP, HBV_Cas12_gRNA4__TANK, HBV_Cas12_gRNA4__TCF12, HBV_Cas12_gRNA4__TEKT5, HBV_Cas12_gRNA4__VTI1A, HBV_Cas12_gRNA4__ZBTB44-DT, HBV_Cas12_gRNA4__ZNF292, HBV_Cas12_gRNA4__none52, HBV_Cas12_gRNA4__none54, HBV_Cas12_gRNA4__none55, HBV_Cas12_gRNA4__none56, HBV_Cas12a_Control__CNTN451, HBV_Cas12a_Control__POGLUT3, HBV_Cas12a_Control__ROR1, HBV_Cas12a_Control__SHISA6, HBV_Cas12a_Control__TMC4, HBV_Cas12a_Control__none44, HBV_Cas12a_Control__none46, HBV_Cas12a_Control__none47, HBV_Cas12a_Control__none48, HBV_Cas12a_Control__none49

Metrics

	Group ID	Treated	Total Reads	Percent Edited	Percent Unedited	Percent NHEJ	Percent Perfect HDR	Percent Imperfect HDR	Percent HDR	Percent Other	Percent In Frame	Percent Frameshift	Percent Indels	Percent Insertions	Percent Templated Insertion	Percent GC-Insertion	Percent Deletions
**HBV_Cas12_gRNA4__CD300E**	HBV_Fig4	FALSE	461	98.7	1.3	0.0	0.0	0.0	0.0	98.7	100.0	0.0	0.0	0.0	0.0	0.0	0.0
**HBV_Cas12_gRNA4__CHKB-DT**	HBV_Fig4	FALSE	6617	2.87	97.13	0.03	0.0	0.0	0.0	2.84	99.97	0.03	0.03	0.0	0.0	0.0	0.03
**HBV_Cas12_gRNA4__GNA12**	HBV_Fig4	FALSE	32106	3.26	96.74	0.11	0.0	0.0	0.0	3.15	99.9	0.1	0.11	0.0	0.0	0.0	0.11
**HBV_Cas12_gRNA4__LINC00989**	HBV_Fig4	FALSE	65488	3.19	96.81	0.09	0.0	0.0	0.0	3.1	99.91	0.09	0.09	0.0	0.0	0.0	0.09
**HBV_Cas12_gRNA4__LOC107986930**	HBV_Fig4	FALSE	35770	4.23	95.77	0.16	0.0	0.0	0.0	4.07	99.84	0.16	0.16	0.0	0.0	0.0	0.16
**HBV_Cas12_gRNA4__MRGBP**	HBV_Fig4	FALSE	14379	44.71	55.29	0.08	0.0	0.0	0.0	44.63	99.94	0.06	0.08	0.0	0.0	0.0	0.08
**HBV_Cas12_gRNA4__PCNX2**	HBV_Fig4	FALSE	172718	3.49	96.51	0.09	0.0	0.0	0.0	3.4	99.91	0.09	0.09	0.0	0.0	0.0	0.09
**HBV_Cas12_gRNA4__SHROOM2**	HBV_Fig4	FALSE	36849	3.22	96.78	0.44	0.0	0.0	0.0	2.78	99.56	0.44	0.44	0.06	86.96	0.0	0.38
**HBV_Cas12_gRNA4__SYNJ2BP**	HBV_Fig4	FALSE	44876	3.4	96.6	0.07	0.0	0.0	0.0	3.33	99.93	0.07	0.07	0.0	0.0	0.0	0.07
**HBV_Cas12_gRNA4__TANK**	HBV_Fig4	FALSE	79276	3.59	96.41	0.19	0.0	0.0	0.0	3.4	99.81	0.19	0.19	0.0	0.0	0.0	0.19
**HBV_Cas12_gRNA4__TCF12**	HBV_Fig4	FALSE	19943	99.96	0.04	99.85	0.0	0.0	0.0	0.11	99.97	0.03	99.85	99.85	0.0	0.27	0.01
**HBV_Cas12_gRNA4__TEKT5**	HBV_Fig4	FALSE	1437862	4.37	95.63	0.08	0.0	0.0	0.0	4.29	99.93	0.07	0.08	0.0	0.0	0.0	0.08
**HBV_Cas12_gRNA4__VTI1A**	HBV_Fig4	FALSE	26004	3.81	96.19	0.01	0.0	0.0	0.0	3.8	99.99	0.01	0.01	0.0	0.0	0.0	0.01
**HBV_Cas12_gRNA4__ZBTB44-DT**	HBV_Fig4	FALSE	3405	3.11	96.89	0.03	0.0	0.0	0.0	3.08	99.97	0.03	0.03	0.0	0.0	0.0	0.03
**HBV_Cas12_gRNA4__ZNF292**	HBV_Fig4	FALSE	44081	4.24	95.76	0.1	0.0	0.0	0.0	4.14	99.9	0.1	0.1	0.0	0.0	0.0	0.1
**HBV_Cas12_gRNA4__none52**	HBV_Fig4	FALSE	72941	3.68	96.32	0.03	0.0	0.0	0.0	3.65	99.97	0.03	0.03	0.0	0.0	0.0	0.03
**HBV_Cas12_gRNA4__none54**	HBV_Fig4	FALSE	4976	3.42	96.58	0.08	0.0	0.0	0.0	3.34	99.92	0.08	0.08	0.0	0.0	0.0	0.08
**HBV_Cas12_gRNA4__none55**	HBV_Fig4	FALSE	46773	99.92	0.08	0.1	0.0	0.0	0.0	99.82	99.92	0.08	0.1	0.0	0.0	0.0	0.1
**HBV_Cas12_gRNA4__none56**	HBV_Fig4	FALSE	65221	3.76	96.24	0.12	0.0	0.0	0.0	3.64	99.88	0.12	0.12	0.0	0.0	0.0	0.12
**HBV_Cas12a_Control__CNTN451**	HBV_Fig4	FALSE	52847	3.9	96.1	0.06	0.0	0.0	0.0	3.84	99.94	0.06	0.06	0.0	0.0	0.0	0.06
**HBV_Cas12a_Control__POGLUT3**	HBV_Fig4	FALSE	148407	36.75	63.25	0.26	0.0	0.0	0.0	36.49	99.74	0.26	0.26	0.02	100.0	0.0	0.24
**HBV_Cas12a_Control__ROR1**	HBV_Fig4	FALSE	8558	9.22	90.78	3.31	0.0	0.0	0.0	5.91	97.15	2.85	3.31	0.89	0.0	0.0	2.43
**HBV_Cas12a_Control__SHISA6**	HBV_Fig4	FALSE	68512	2.97	97.03	0.25	0.0	0.0	0.0	2.72	99.76	0.24	0.25	0.01	0.0	20.0	0.23
**HBV_Cas12a_Control__TMC4**	HBV_Fig4	FALSE	7380	100.0	0.0	100.0	0.0	0.0	0.0	0.0	97.02	2.98	100.0	100.0	0.0	0.0	0.0
**HBV_Cas12a_Control__none44**	HBV_Fig4	FALSE	472	93.86	6.14	83.47	0.0	0.0	0.0	10.39	38.14	61.86	83.47	18.64	0.0	25.0	64.83
**HBV_Cas12a_Control__none46**	HBV_Fig4	FALSE	1059802	54.86	45.14	0.07	0.0	0.0	0.0	54.79	99.93	0.07	0.07	0.01	92.31	2.56	0.07
**HBV_Cas12a_Control__none47**	HBV_Fig4	FALSE	21581	11.14	88.86	0.13	0.0	0.0	0.0	11.01	99.96	0.04	0.13	0.0	0.0	0.0	0.13
**HBV_Cas12a_Control__none48**	HBV_Fig4	FALSE	452450	4.06	95.94	0.07	0.0	0.0	0.0	3.99	99.93	0.07	0.07	0.0	0.0	0.0	0.07
**HBV_Cas12a_Control__none49**	HBV_Fig4	FALSE	201570	100.0	0.0	97.73	0.0	0.0	0.0	2.27	19.29	80.71	97.73	46.18	0.0	21.38	51.55

Percentages

	% Edited	% Repair Pathways	% Frameshift
HBV_Cas12_gRNA4__CD300E	98.7%	98.7%	100.0%
HBV_Cas12_gRNA4__CHKB-DT	97.13%	97.13%	99.97%
HBV_Cas12_gRNA4__GNA12	96.74%	96.74%	99.9%
HBV_Cas12_gRNA4__LINC00989	96.81%	96.81%	99.91%
HBV_Cas12_gRNA4__LOC107986930	95.77%	95.77%	99.84%
HBV_Cas12_gRNA4__MRGBP	55.29% 44.71%	55.29% 44.63%	99.94%
HBV_Cas12_gRNA4__PCNX2	96.51%	96.51%	99.91%
HBV_Cas12_gRNA4__SHROOM2	96.78%	96.78%	99.56%
HBV_Cas12_gRNA4__SYNJ2BP	96.6%	96.6%	99.93%
HBV_Cas12_gRNA4__TANK	96.41%	96.41%	99.81%
HBV_Cas12_gRNA4__TCF12	99.96%	99.85%	99.97%
HBV_Cas12_gRNA4__TEKT5	95.63%	95.63%	99.93%
HBV_Cas12_gRNA4__VTI1A	96.19%	96.19%	99.99%
HBV_Cas12_gRNA4__ZBTB44-DT	96.89%	96.89%	99.97%
HBV_Cas12_gRNA4__ZNF292	95.76%	95.76%	99.9%
HBV_Cas12_gRNA4__none52	96.32%	96.32%	99.97%
HBV_Cas12_gRNA4__none54	96.58%	96.58%	99.92%
HBV_Cas12_gRNA4__none55	99.92%	99.82%	99.92%
HBV_Cas12_gRNA4__none56	96.24%	96.24%	99.88%
HBV_Cas12a_Control__CNTN451	96.1%	96.1%	99.94%
HBV_Cas12a_Control__POGLUT3	63.25% 36.75%	63.25% 36.49%	99.74%
HBV_Cas12a_Control__ROR1	90.78%	90.78%	97.15%
HBV_Cas12a_Control__SHISA6	97.03%	97.03%	99.76%
HBV_Cas12a_Control__TMC4	100.0%	100.0%	97.02%
HBV_Cas12a_Control__none44	93.86%	83.47% 10.39	38.14% 61.86%
HBV_Cas12a_Control__none46	45.14% 54.86%	45.14% 54.79%	99.93%
HBV_Cas12a_Control__none47	88.86% 11.14	88.86% 11.01	99.96%
HBV_Cas12a_Control__none48	95.94%	95.94%	99.93%

HBV_Cas12a_Control__none49

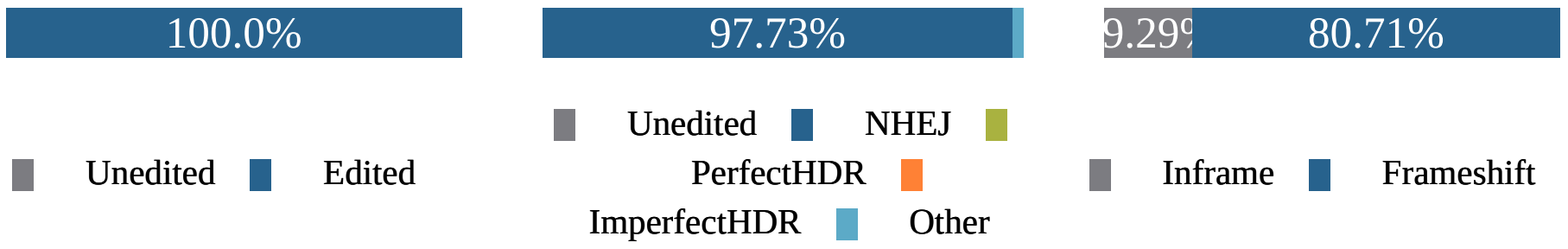

Insertions

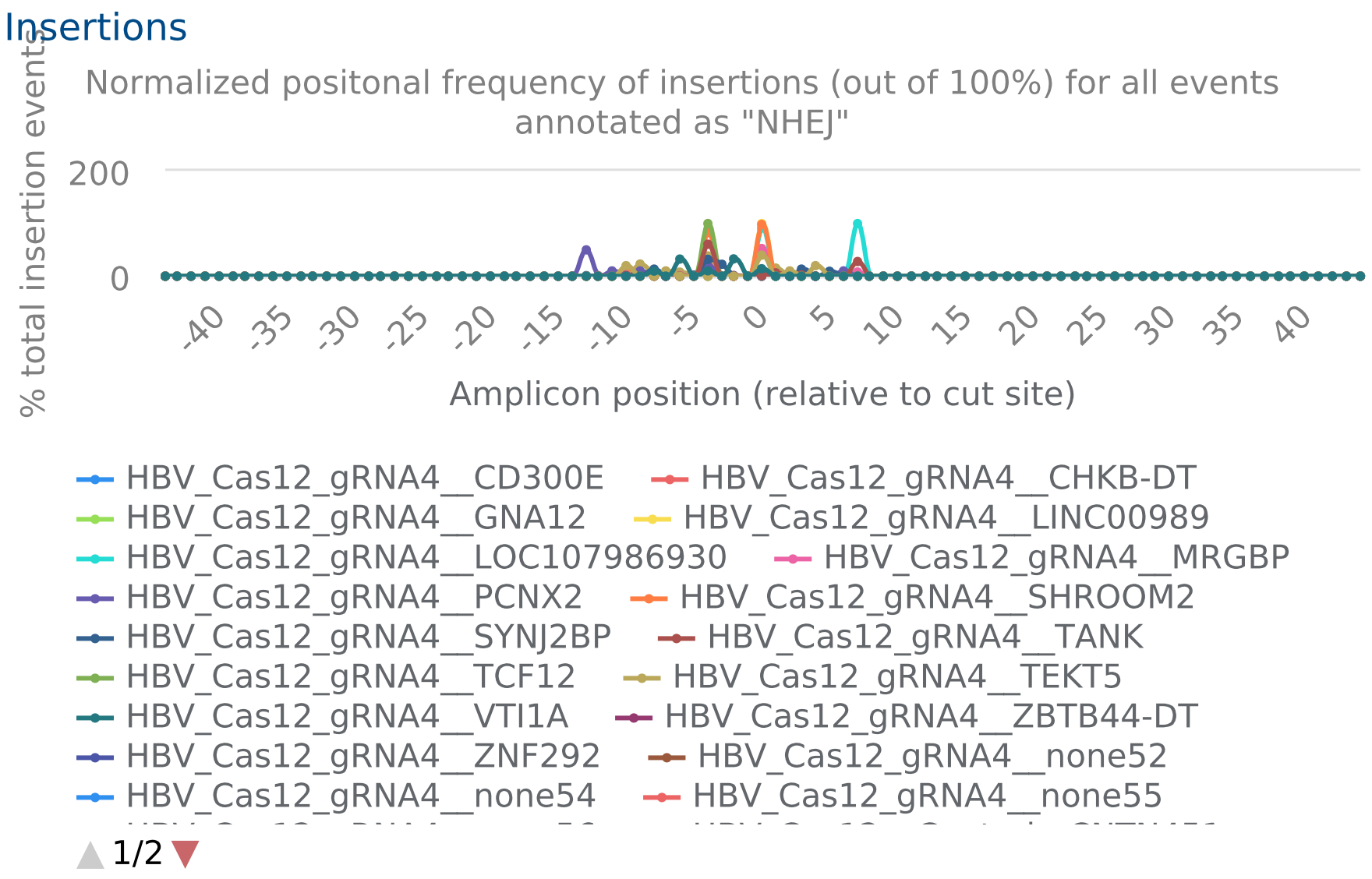

Deletions

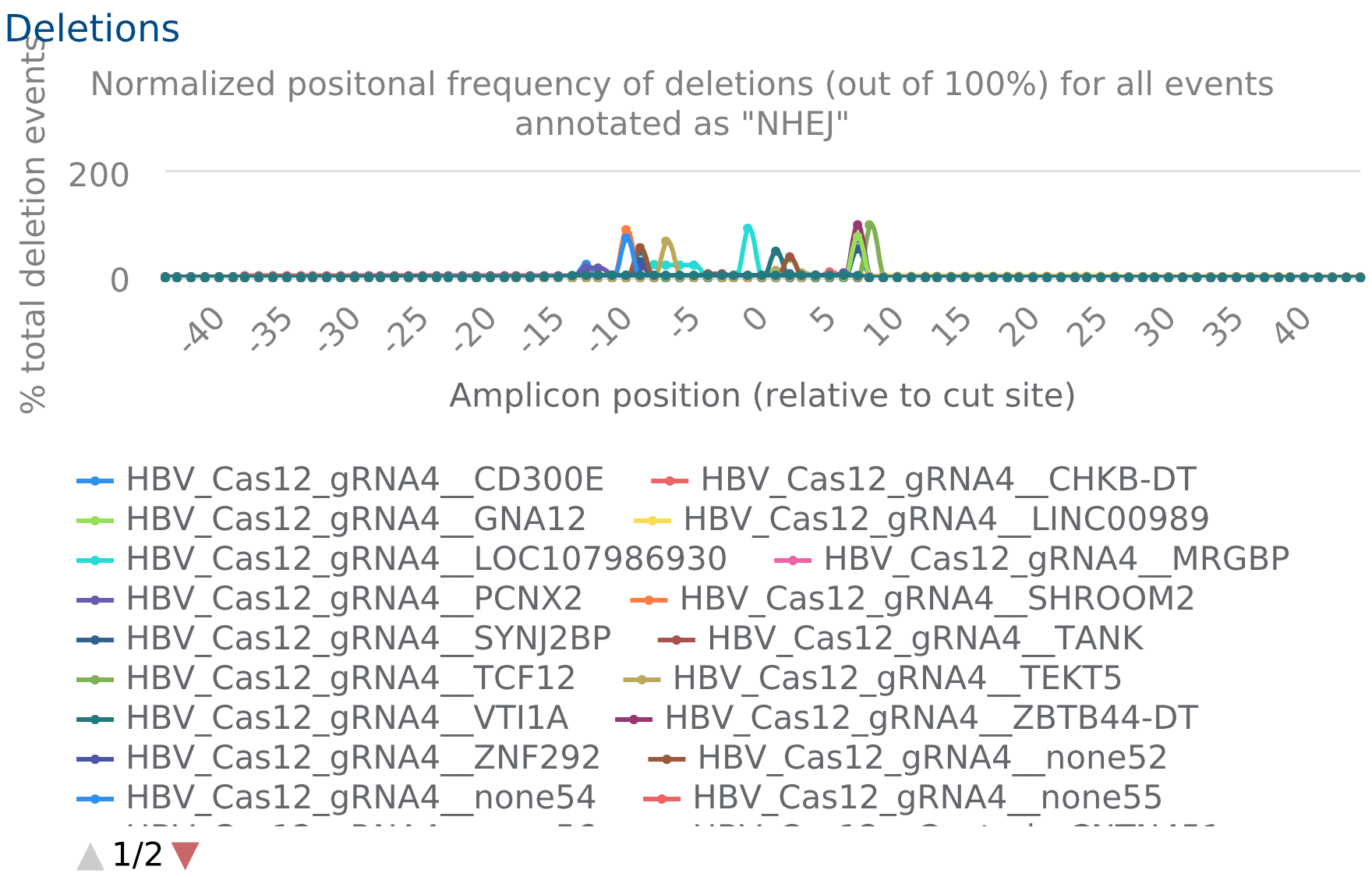

Indel Frequencies

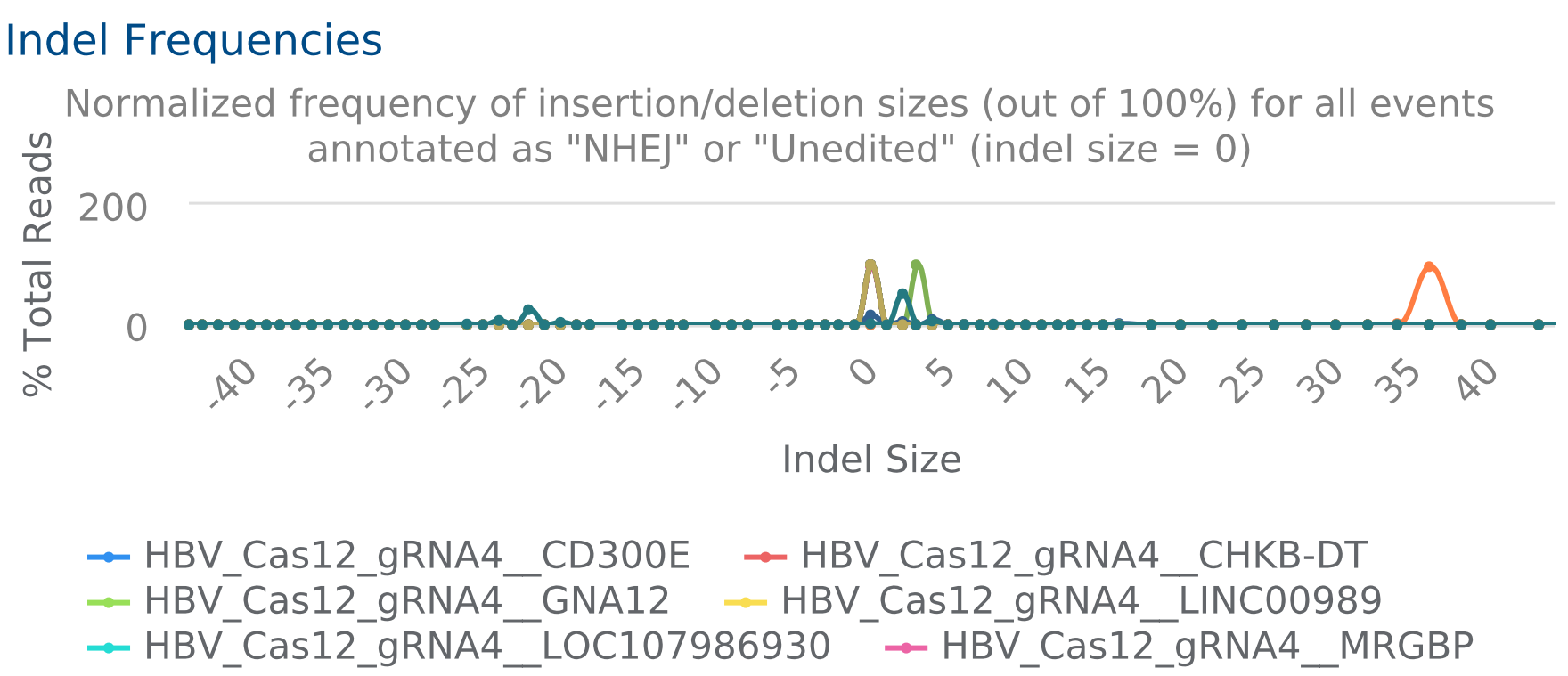

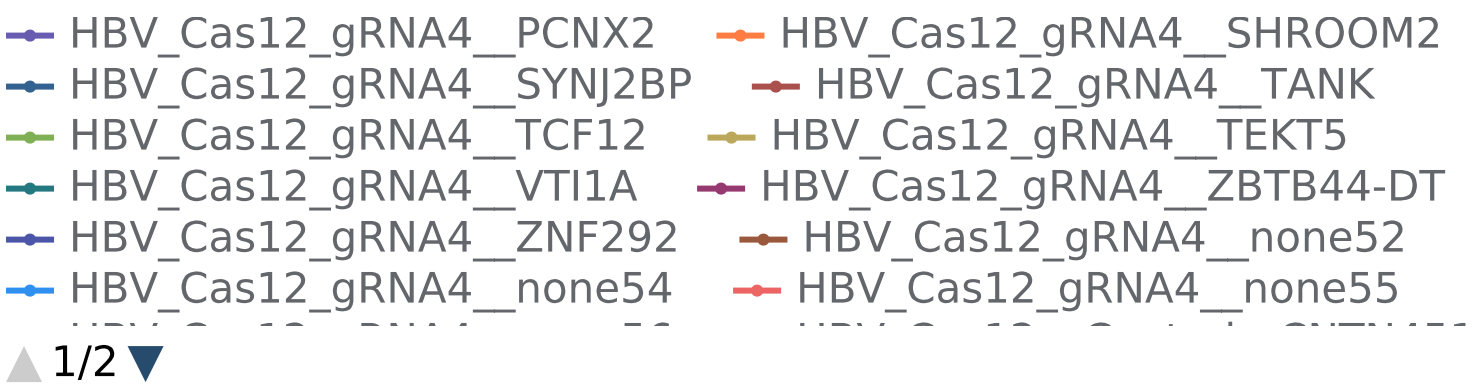
HBV_Cas12_gRNA4__PCNX2
HBV_Cas12_gRNA4__SHROOM2
HBV_Cas12_gRNA4__SYNJ2BP
HBV_Cas12_gRNA4__TANK
HBV_Cas12_gRNA4__TCF12
HBV_Cas12_gRNA4__TEKT5
HBV_Cas12_gRNA4__VTI1A
HBV_Cas12_gRNA4__ZBTB44-DT
HBV_Cas12_gRNA4__ZNF292
HBV_Cas12_gRNA4__none52
HBV_Cas12_gRNA4__none54
HBV_Cas12_gRNA4__none55
1/2

SNP Graph

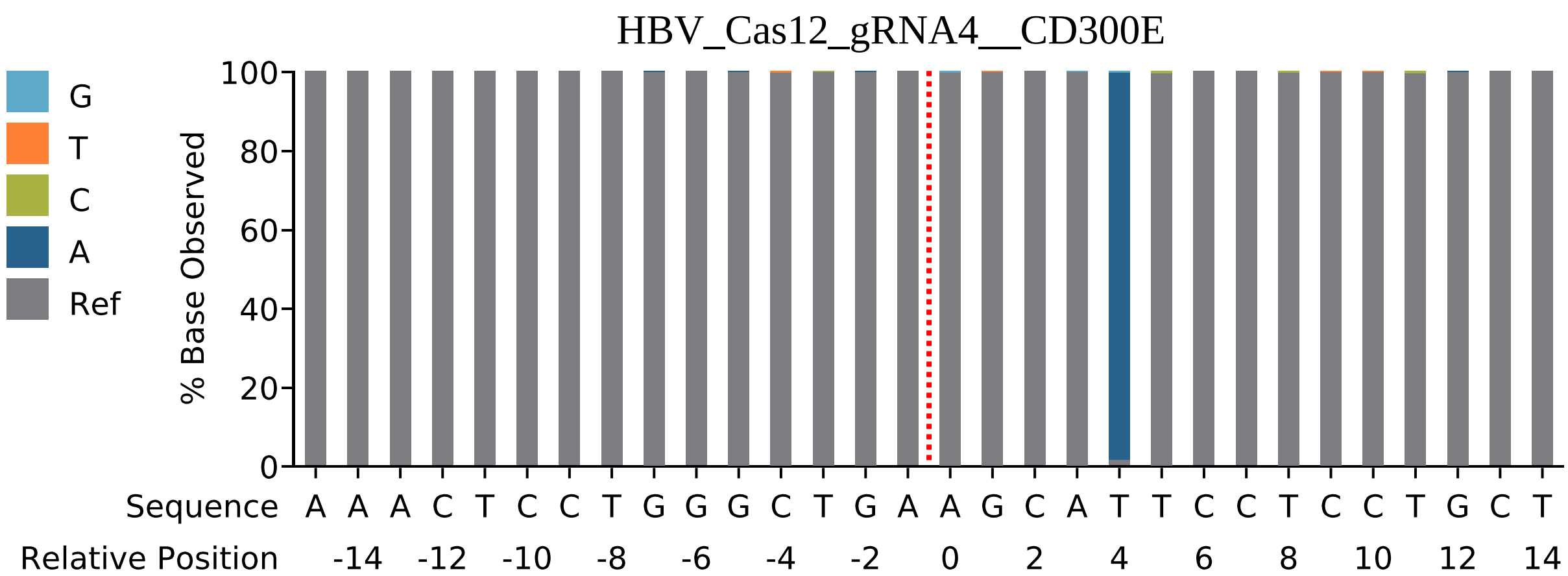
HBV_Cas12_gRNA4__CD300E
G
T
C
A
Ref
% Base Observed
0
20
40
60
80
100
Sequence A A A C T C C T G G G C T G A A G C A T T C C T C C T G C T
Relative Position -14 -12 -10 -8 -6 -4 -2 0 2 4 6 8 10 12 14

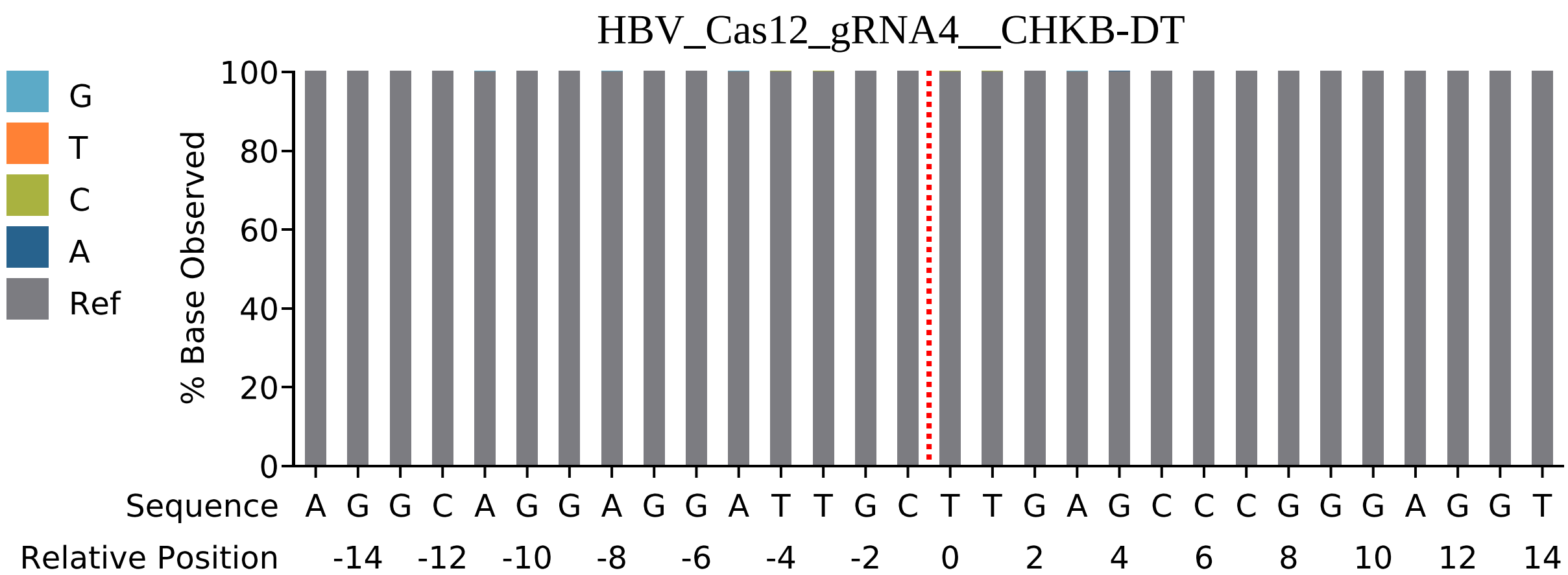
HBV_Cas12_gRNA4__CHKB-DT
G
T
C
A
Ref
% Base Observed
0
20
40
60
80
100
Sequence A G G C A G G A G G A T T G C T T G A G C C C G G G A G G T
Relative Position -14 -12 -10 -8 -6 -4 -2 0 2 4 6 8 10 12 14

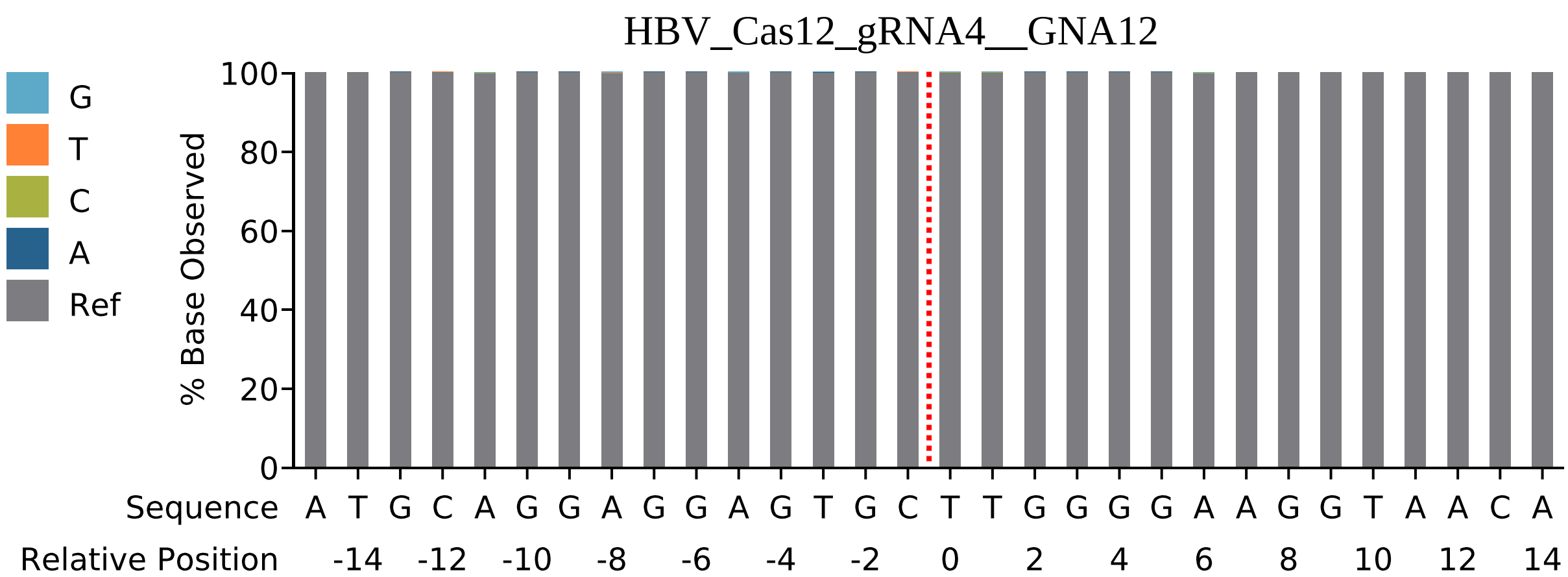
HBV_Cas12_gRNA4__GNA12
G
T
C
A
Ref
% Base Observed
0
20
40
60
80
100
Sequence A T G C A G G A G G A G T G C T T G G G G A A G G T A A C A
Relative Position -14 -12 -10 -8 -6 -4 -2 0 2 4 6 8 10 12 14

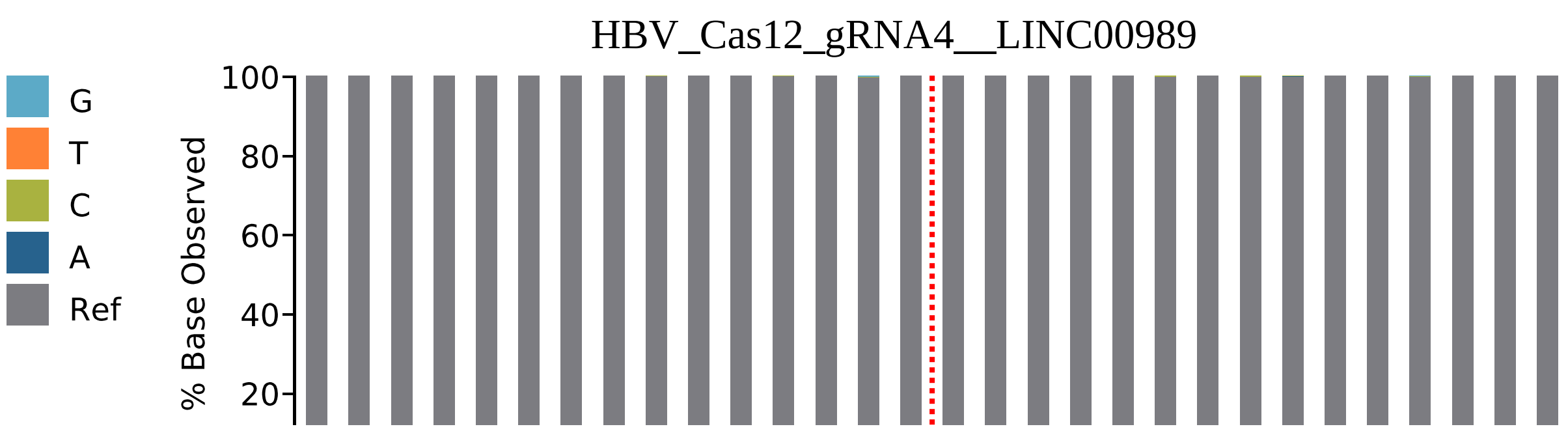
HBV_Cas12_gRNA4__LINC00989
G
T
C
A
Ref
% Base Observed
20
40
60
80
100

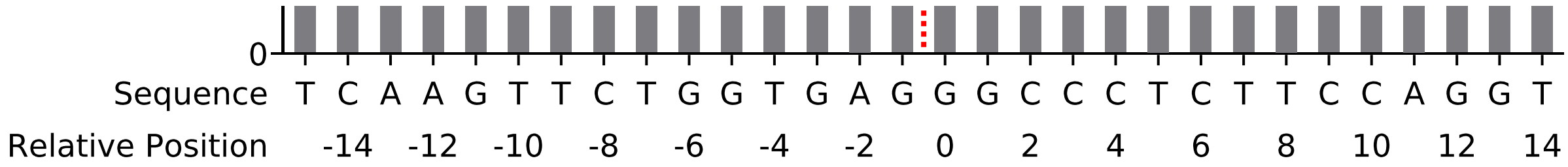

HBV_Cas12_gRNA4__LOC107986930

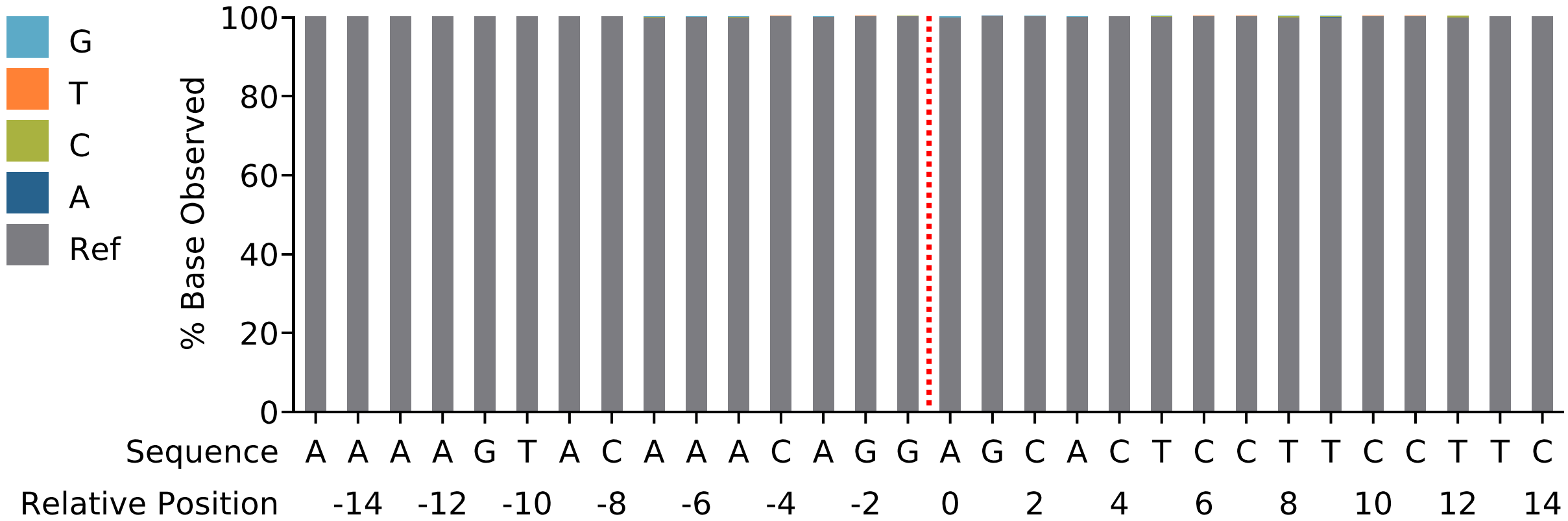

HBV_Cas12_gRNA4__MRGBP

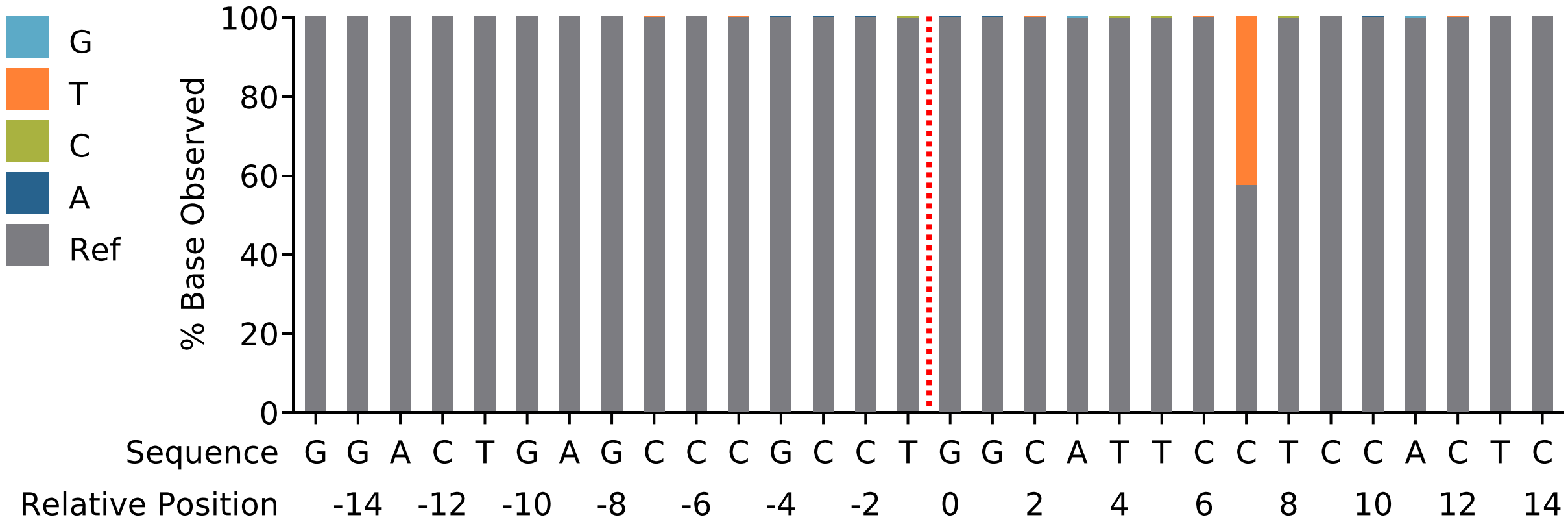

HBV_Cas12_gRNA4__PCNX2

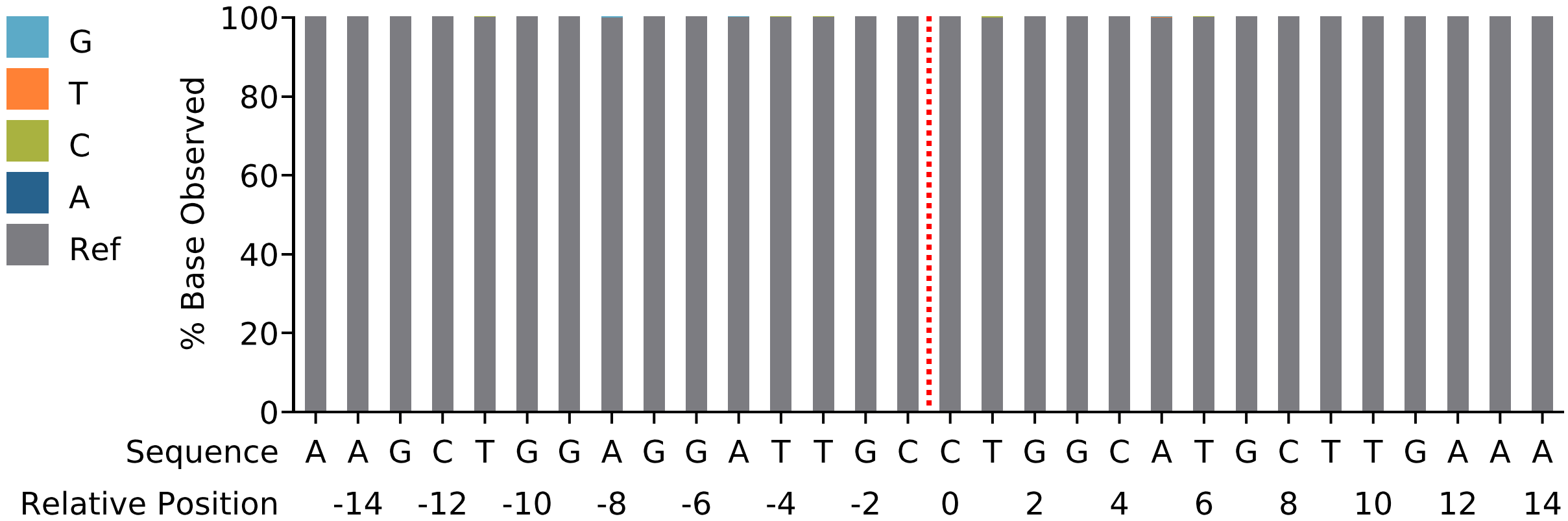

HBV_Cas12_gRNA4__SHROOM2

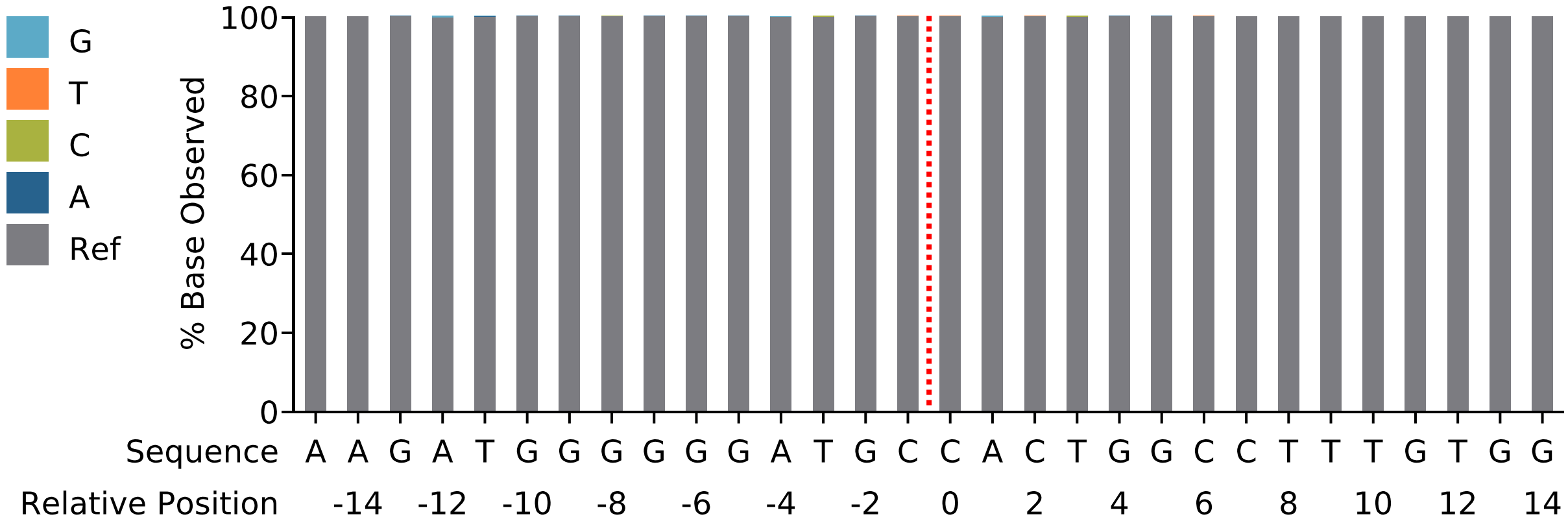

HBV_Cas12_gRNA4__SYNJ2BP

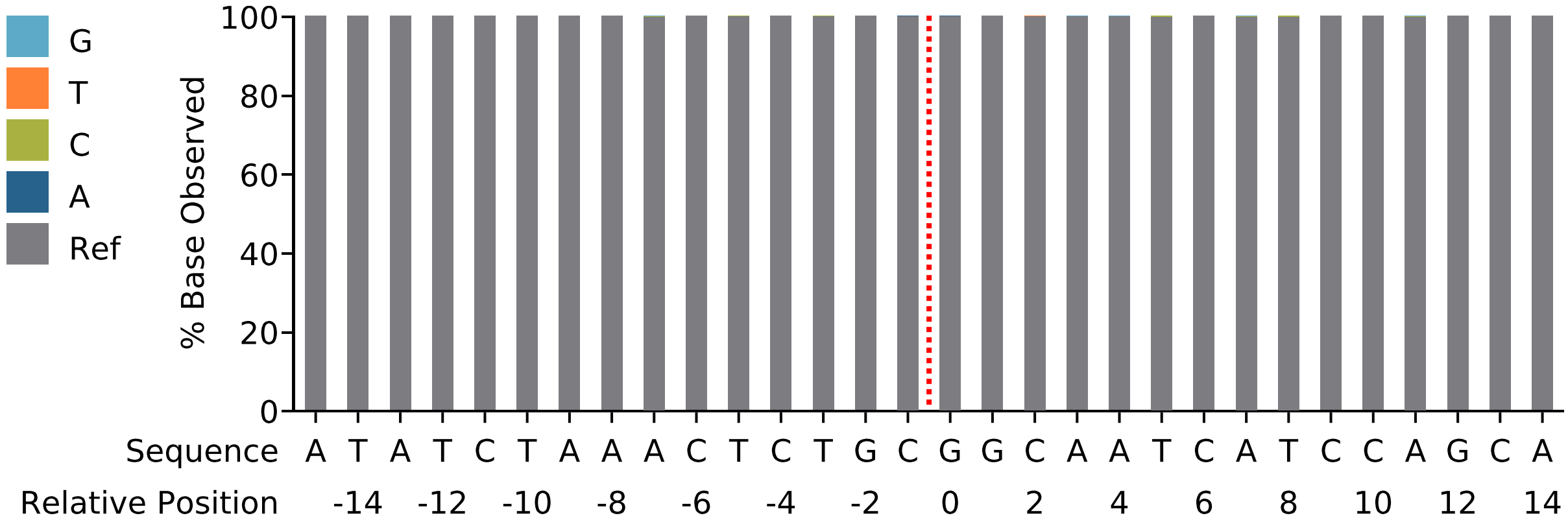

HBV_Cas12_gRNA4__TANK

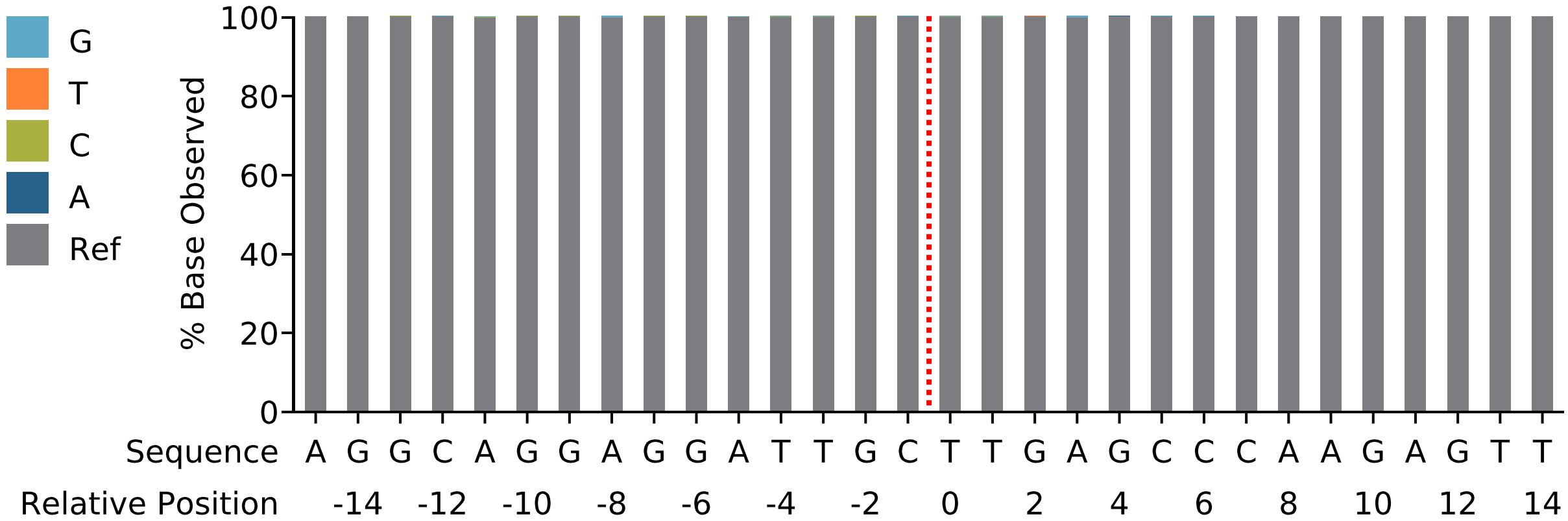

HBV_Cas12_gRNA4__TCF12

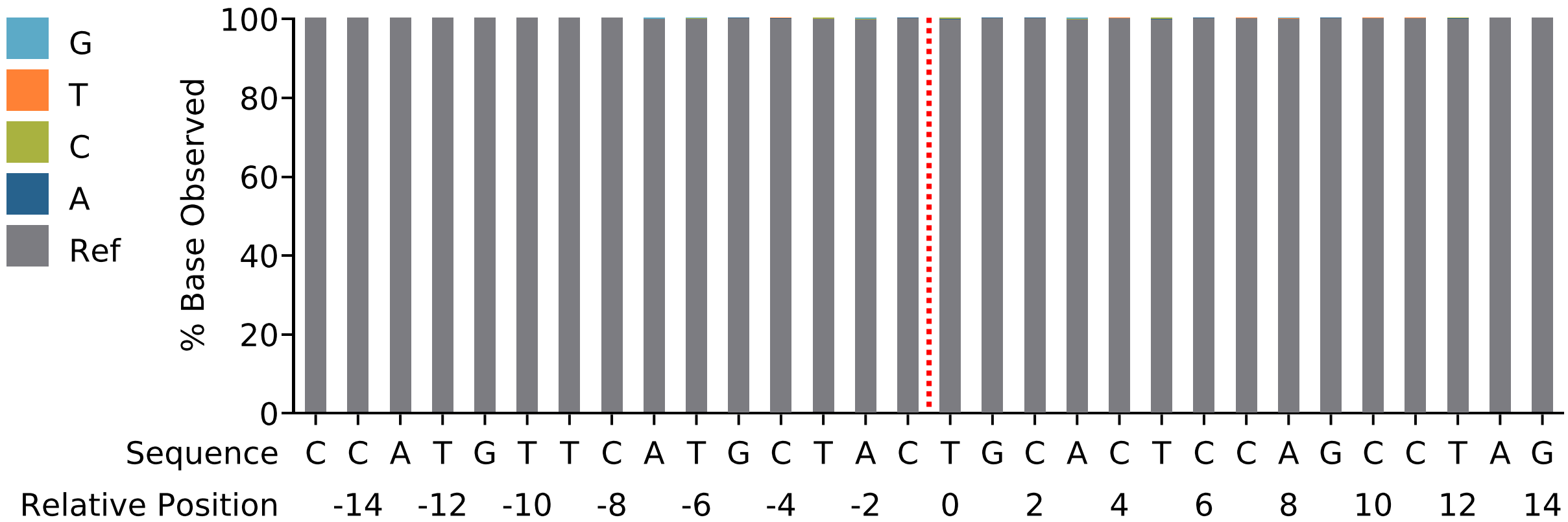

HBV_Cas12_gRNA4__TEKT5

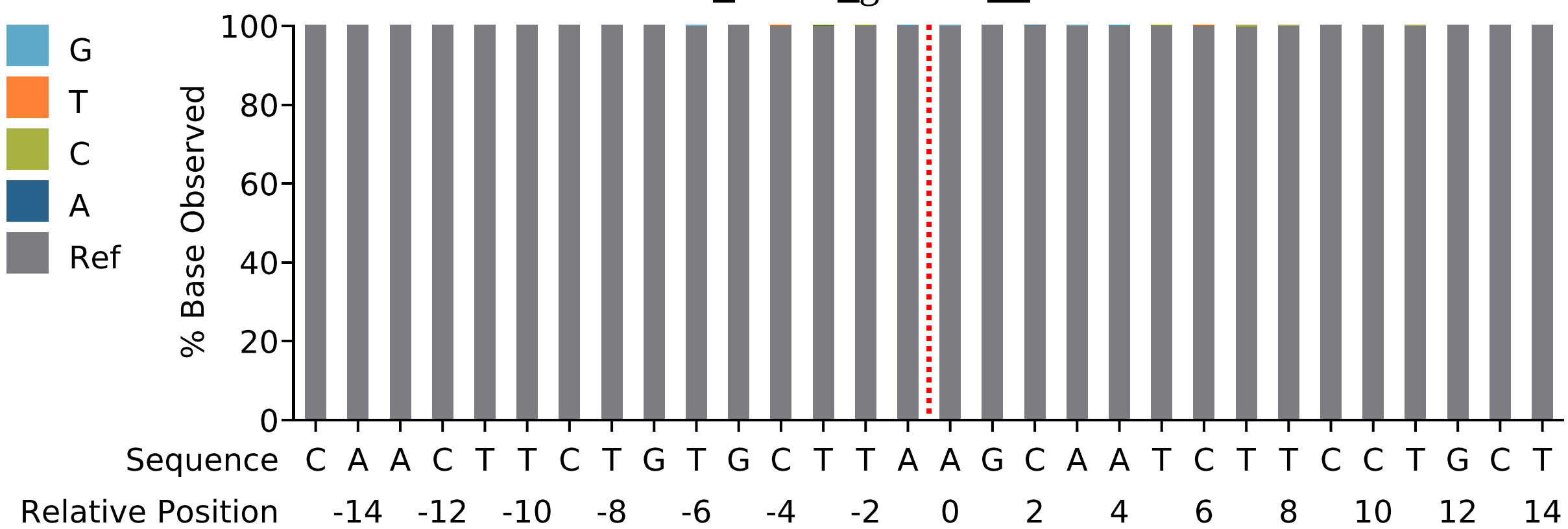

HBV_Cas12_gRNA4__VTI1A

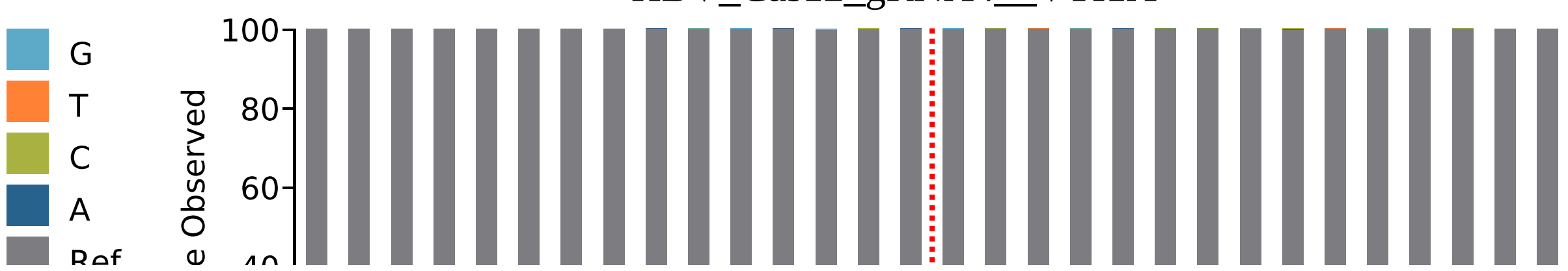

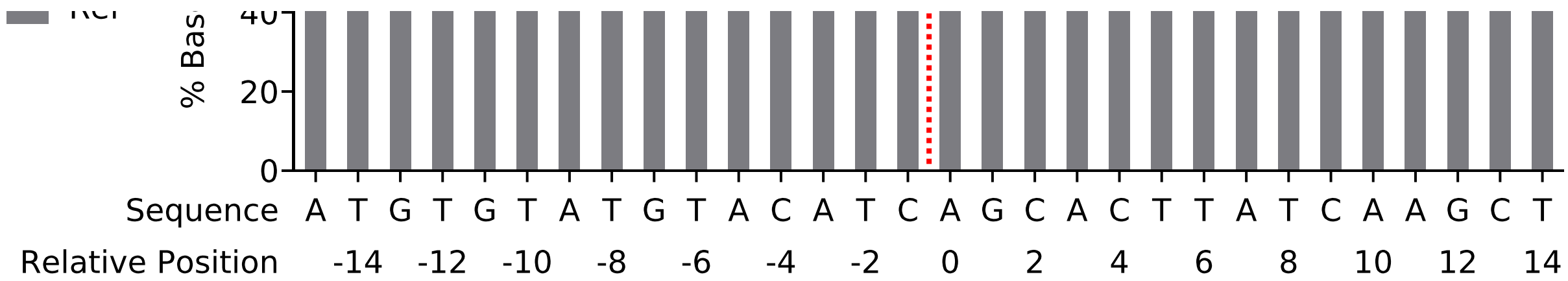

HBV_Cas12_gRNA4__ZBTB44-DT

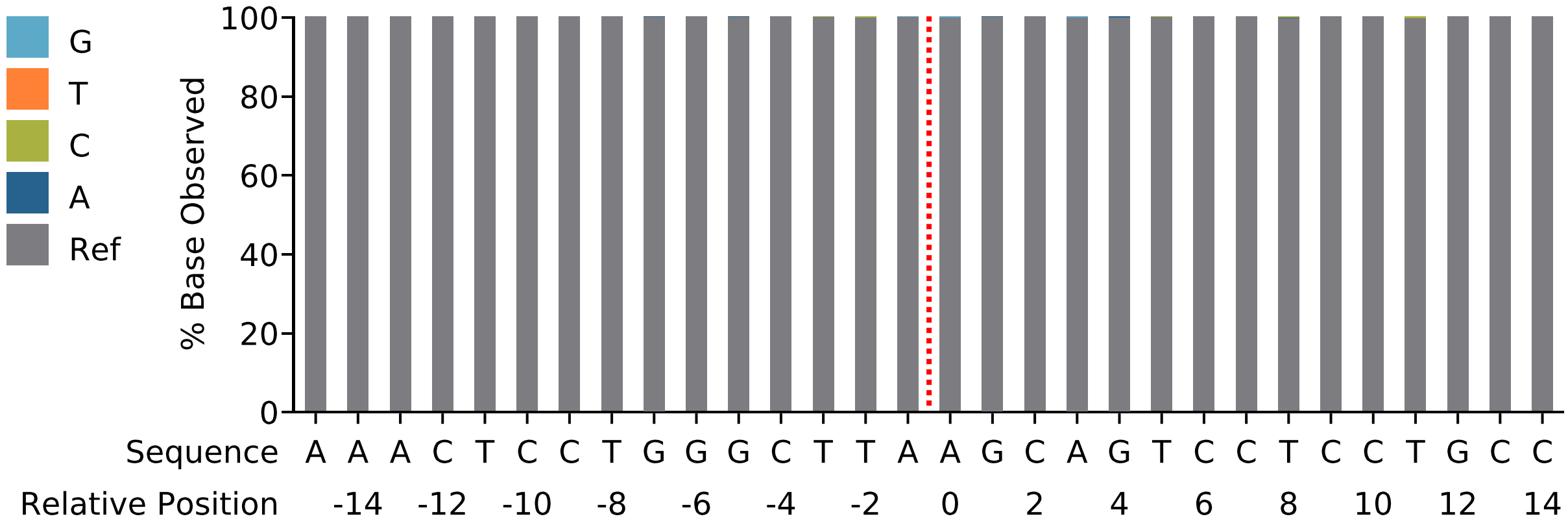

HBV_Cas12_gRNA4__ZNF292

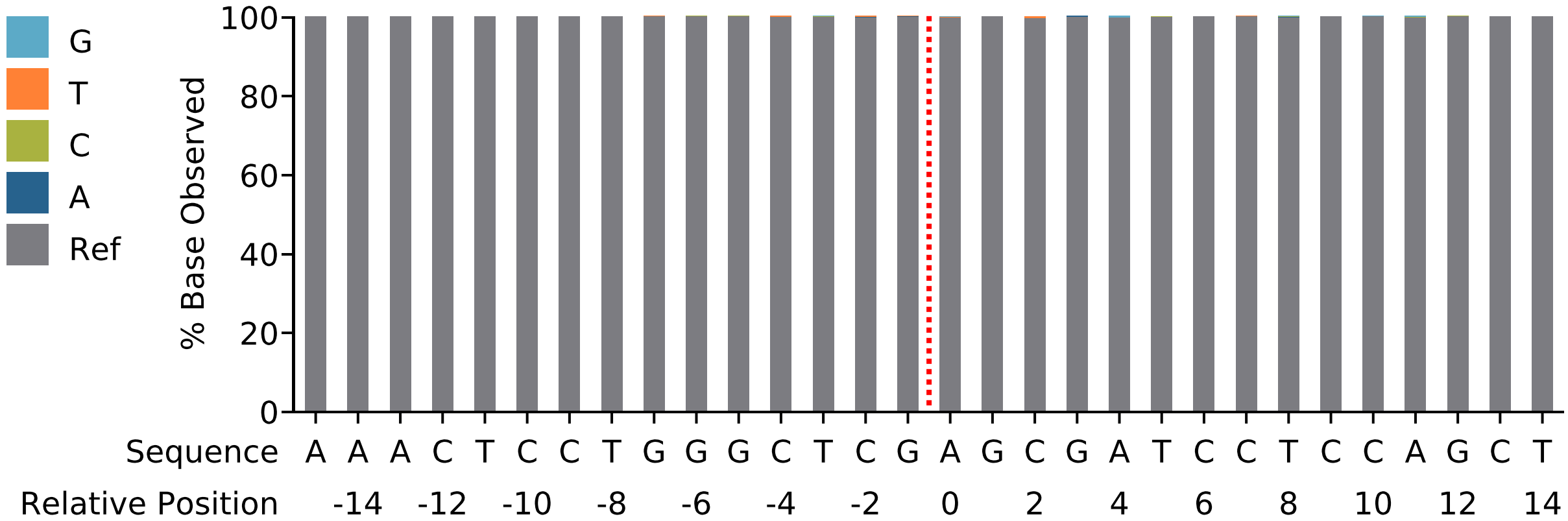

HBV_Cas12_gRNA4__none52

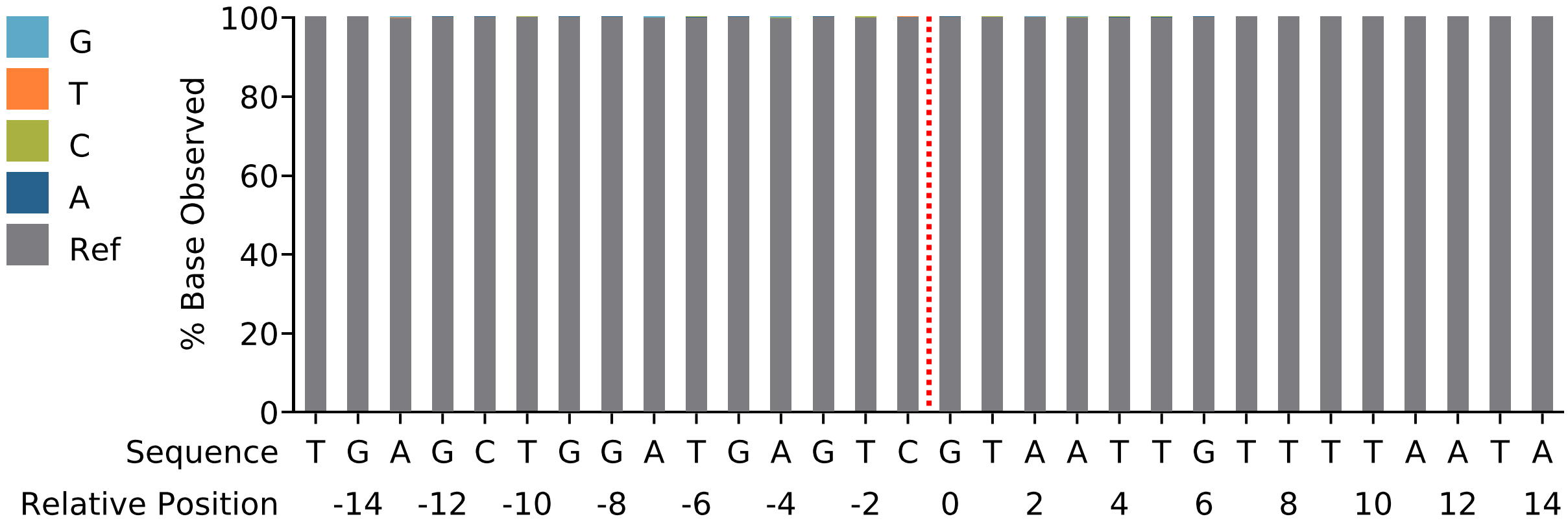

HBV_Cas12_gRNA4__none54

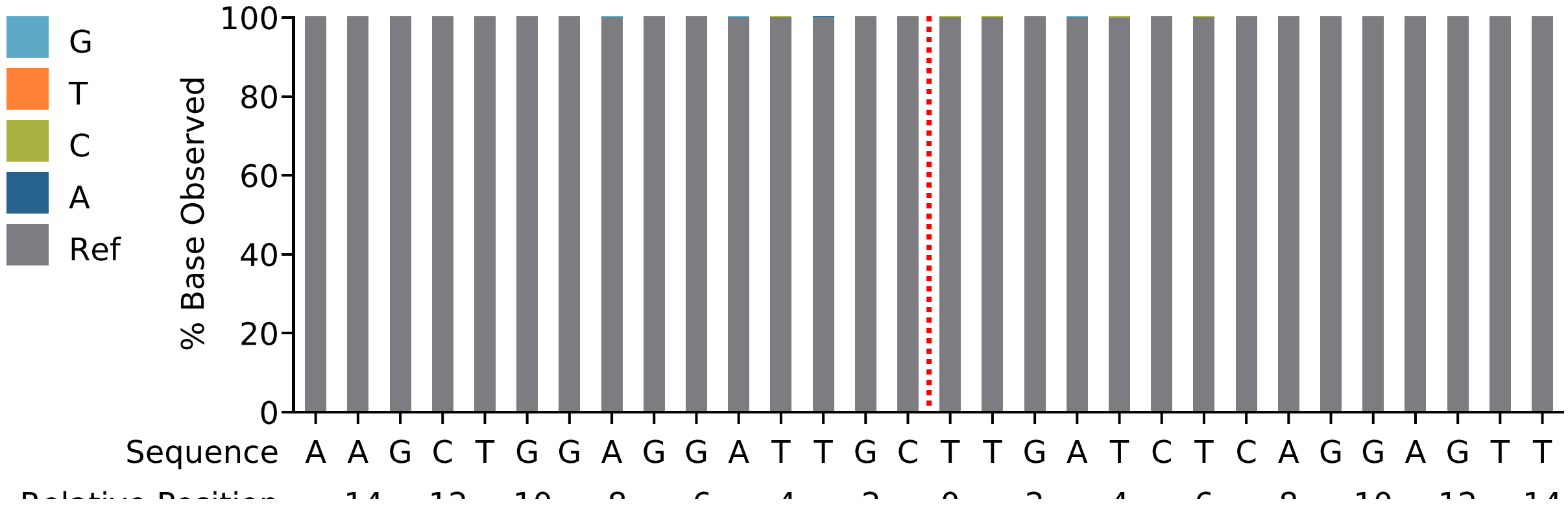

HBV_Cas12_gRNA4__none55

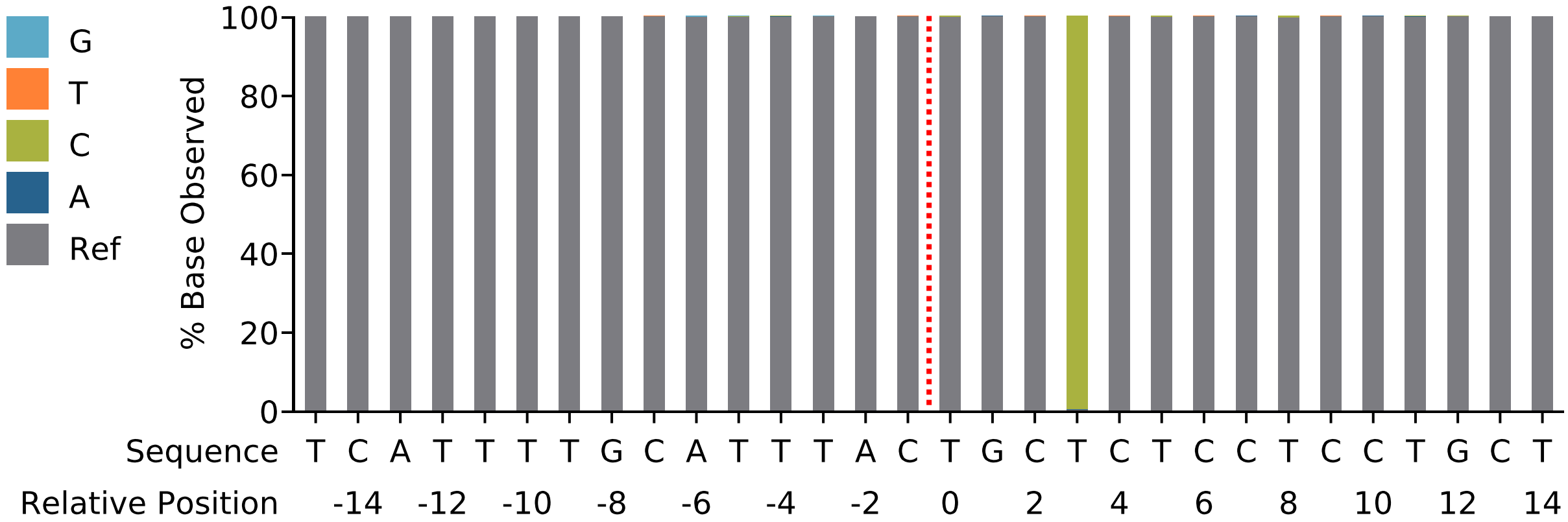

HBV_Cas12_gRNA4__none56

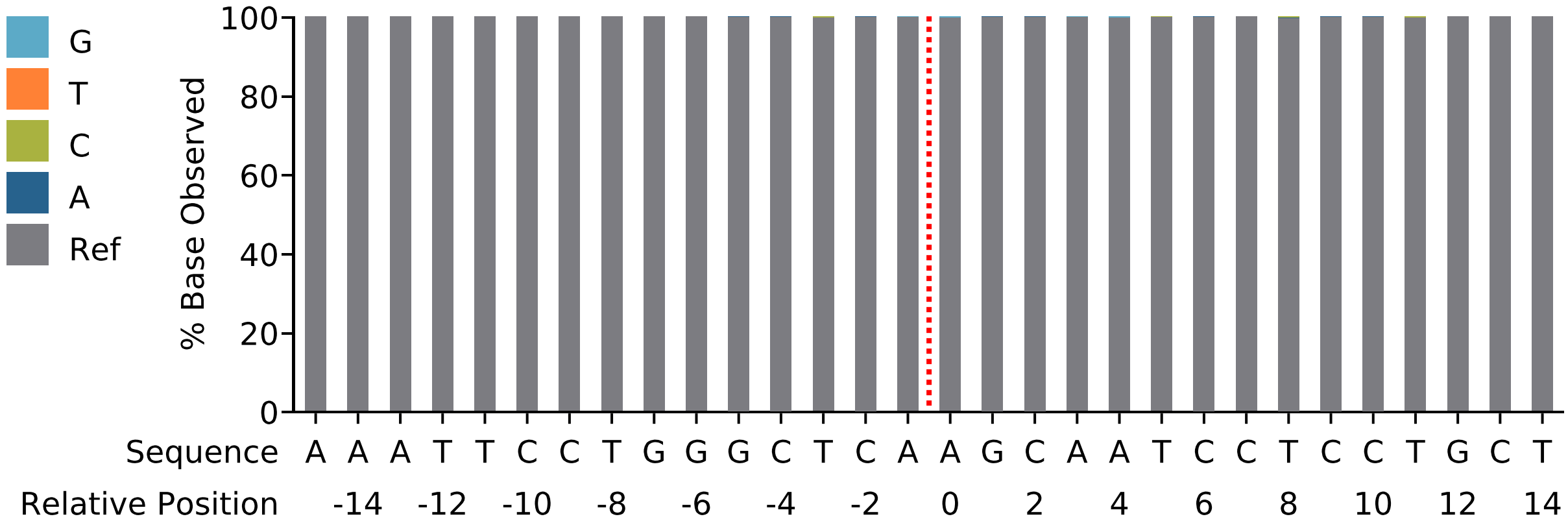

HBV_Cas12a_Control__CNTN451

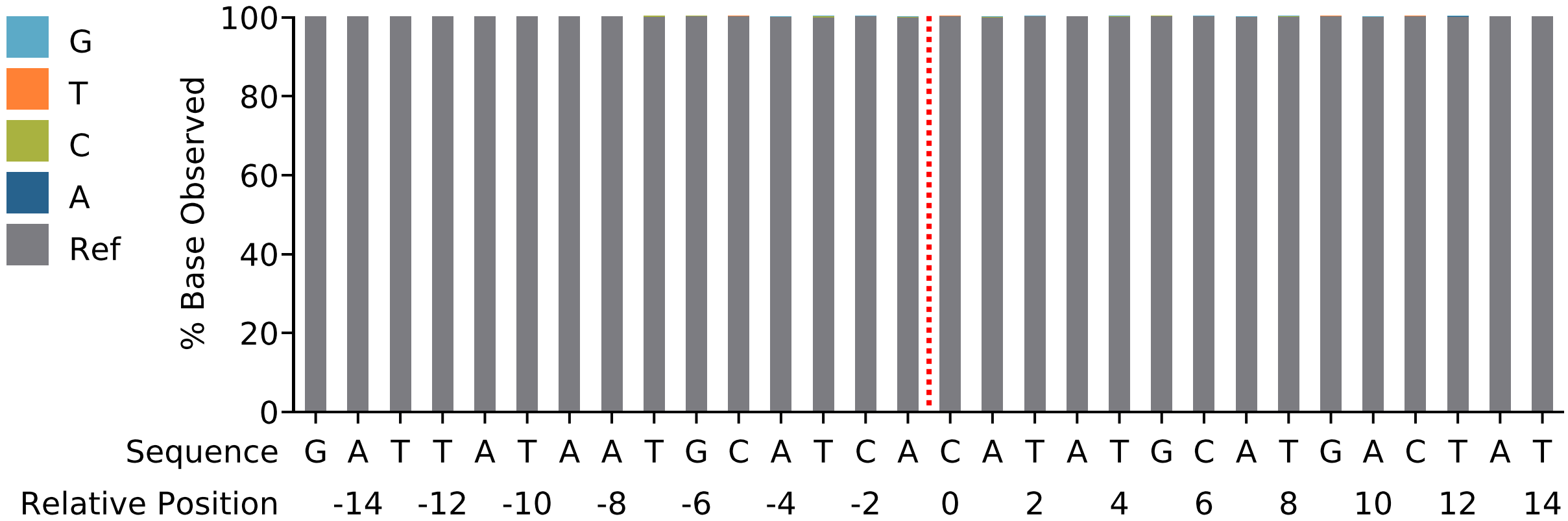

HBV_Cas12a_Control__POGLUT3

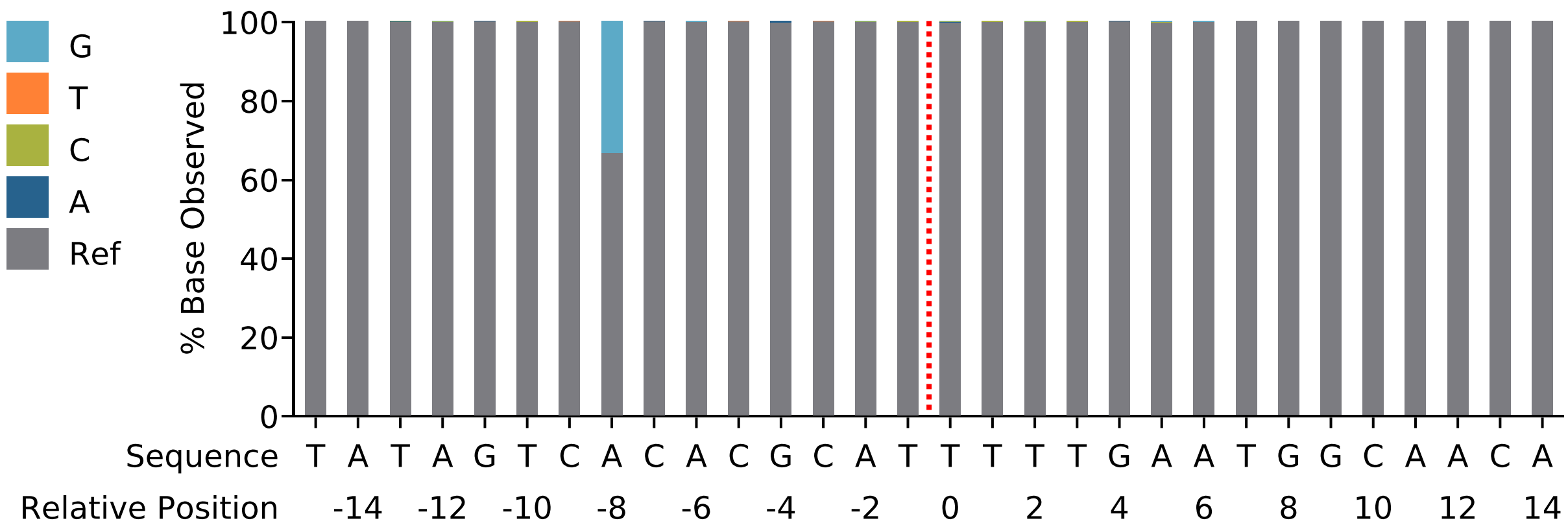

HBV_Cas12a_Control__ROR1

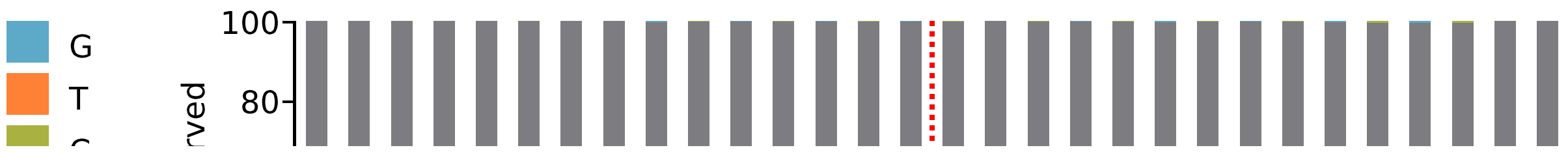

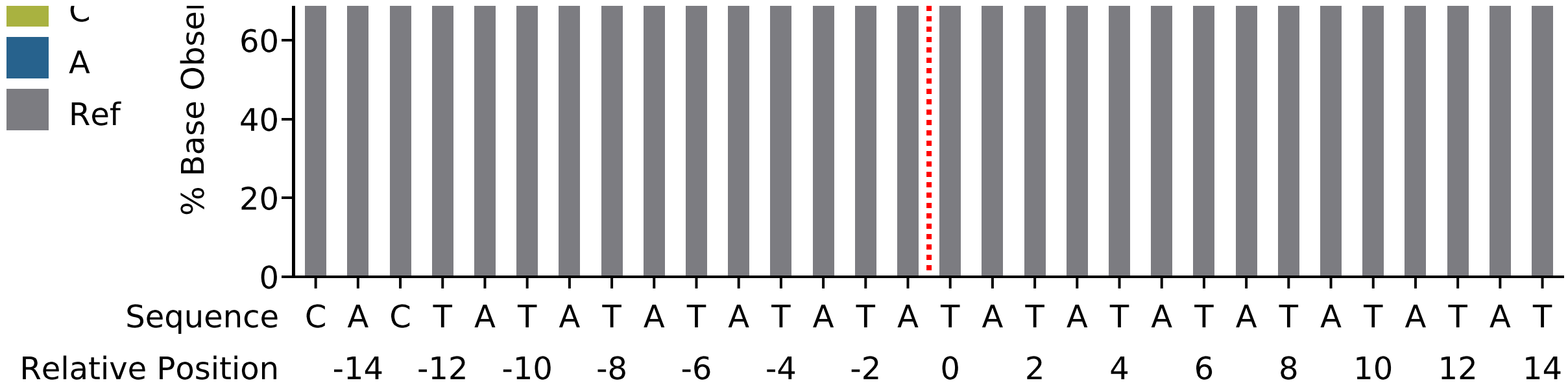

HBV_Cas12a_Control__SHISA6

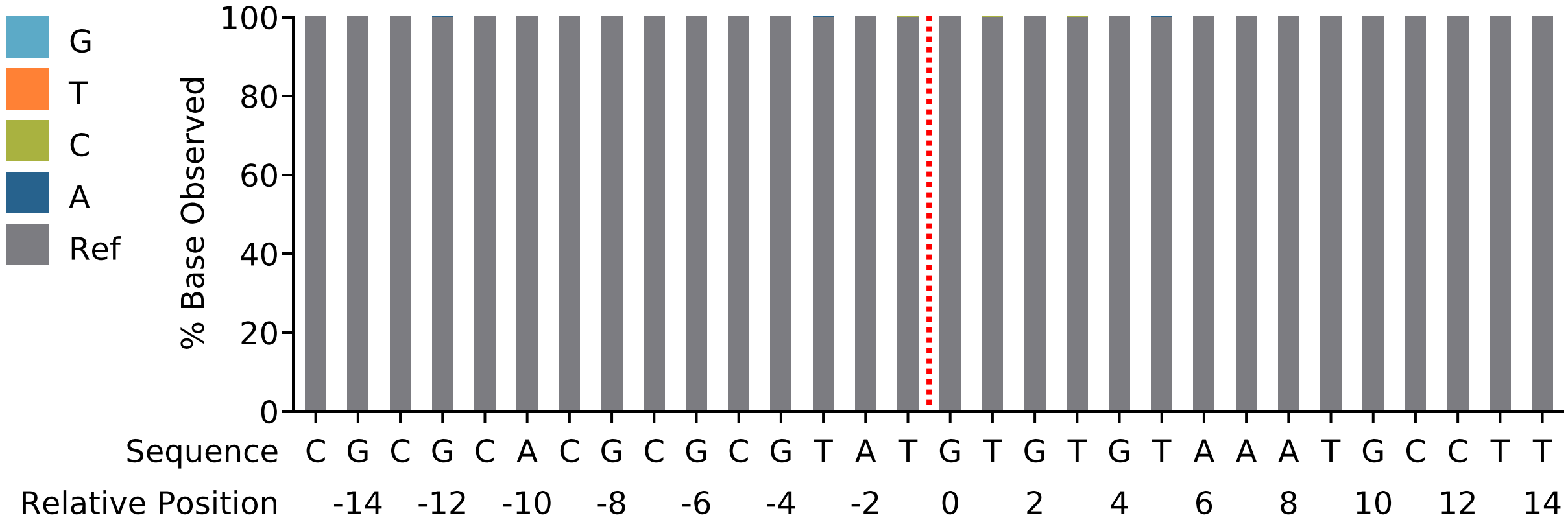

HBV_Cas12a_Control__TMC4

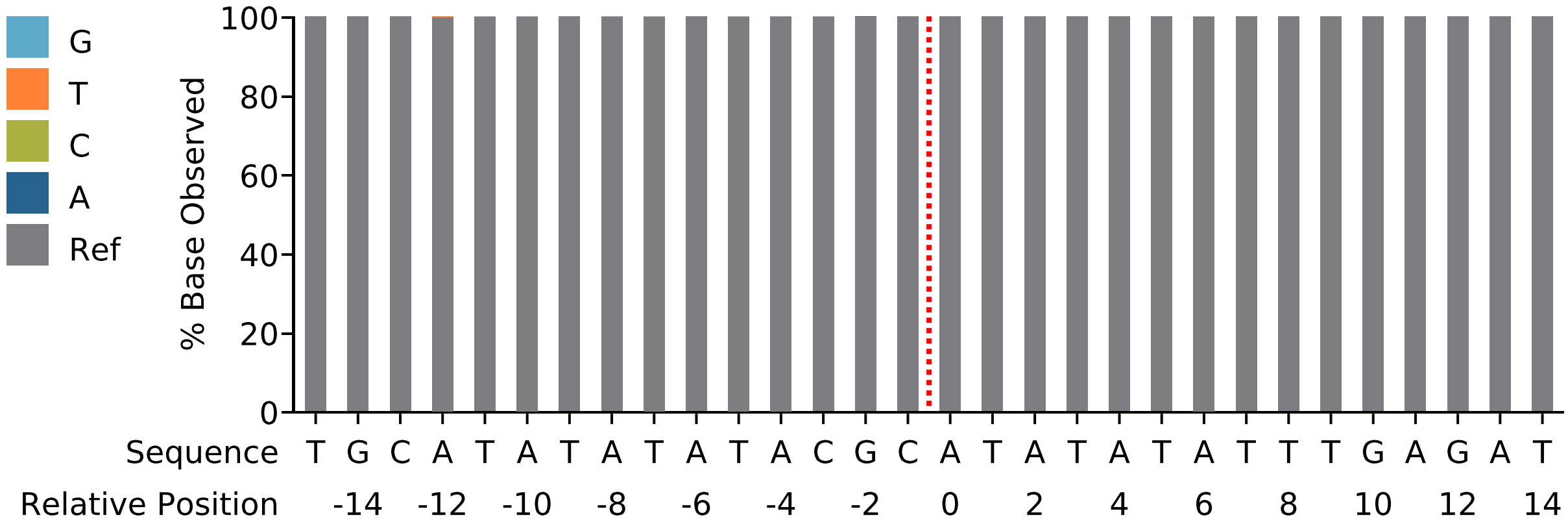

HBV_Cas12a_Control__none44

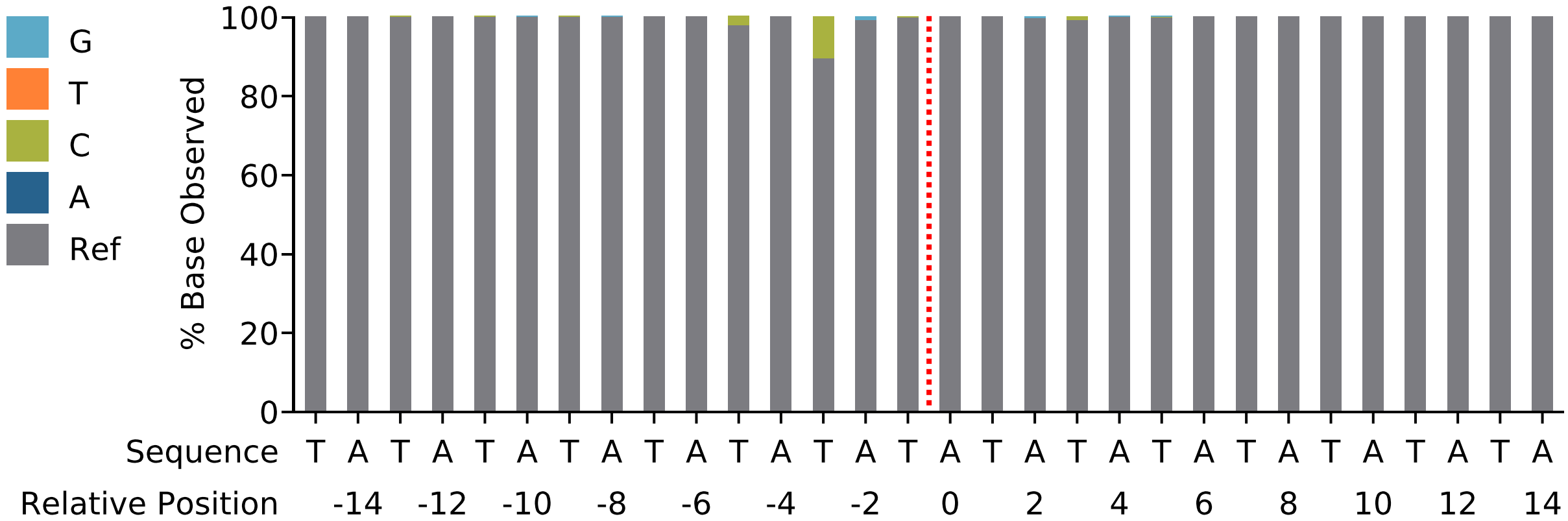

HBV_Cas12a_Control__none46

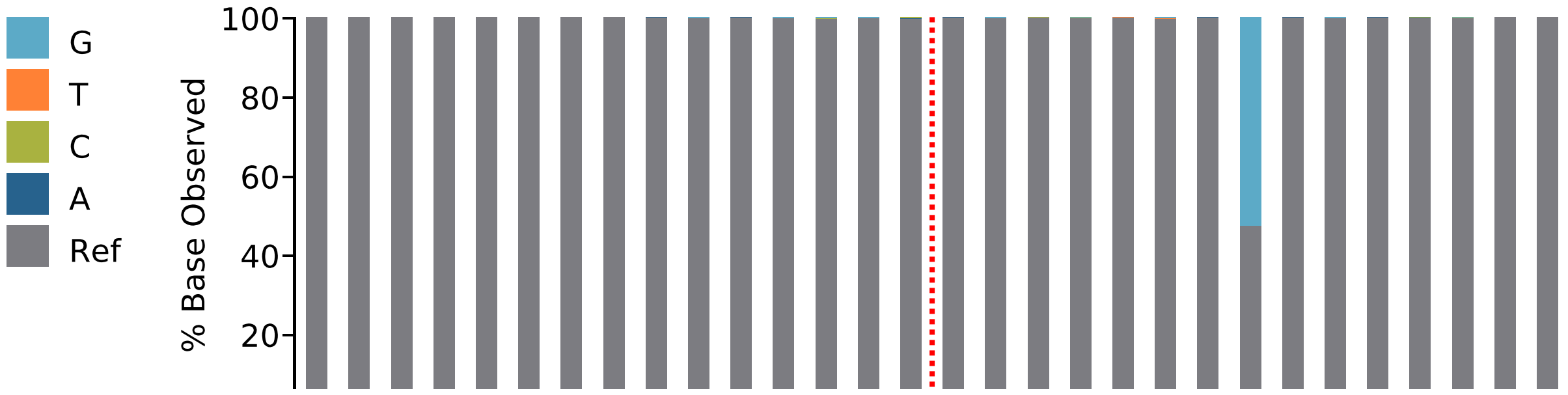

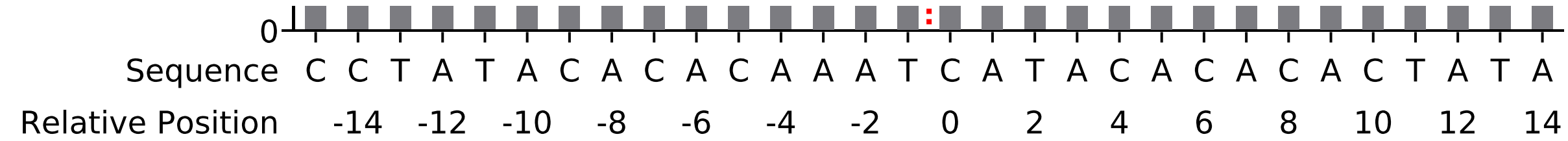

HBV_Cas12a_Control__none47

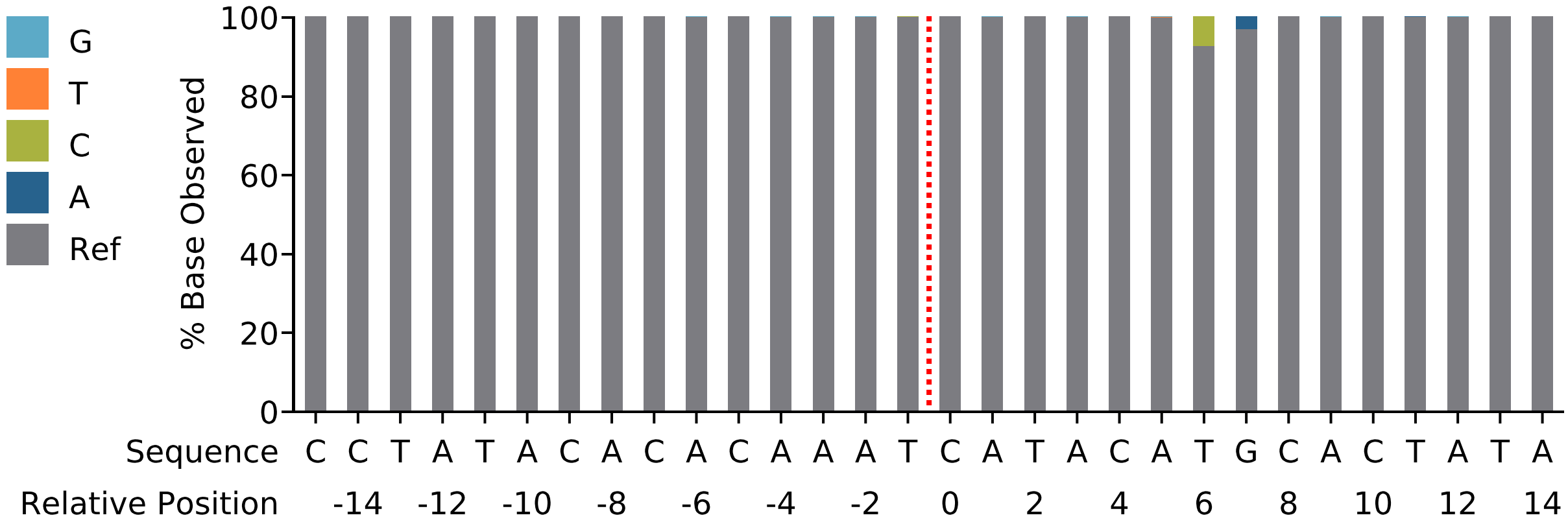

HBV_Cas12a_Control__none48

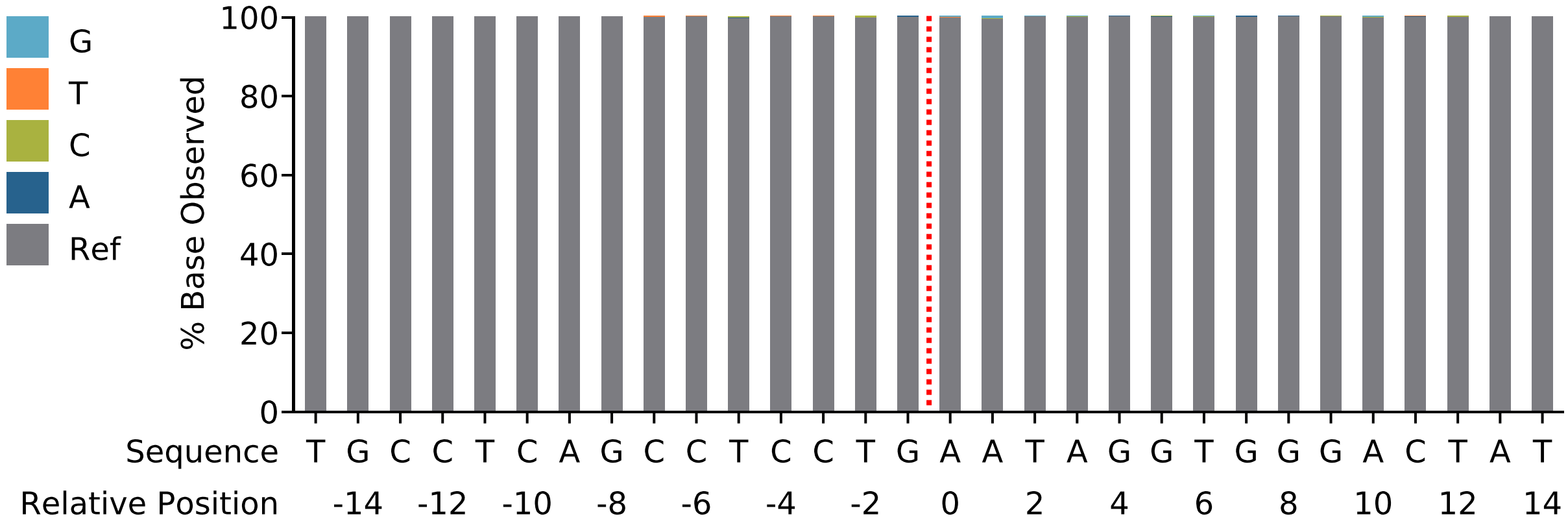

HBV_Cas12a_Control__none49

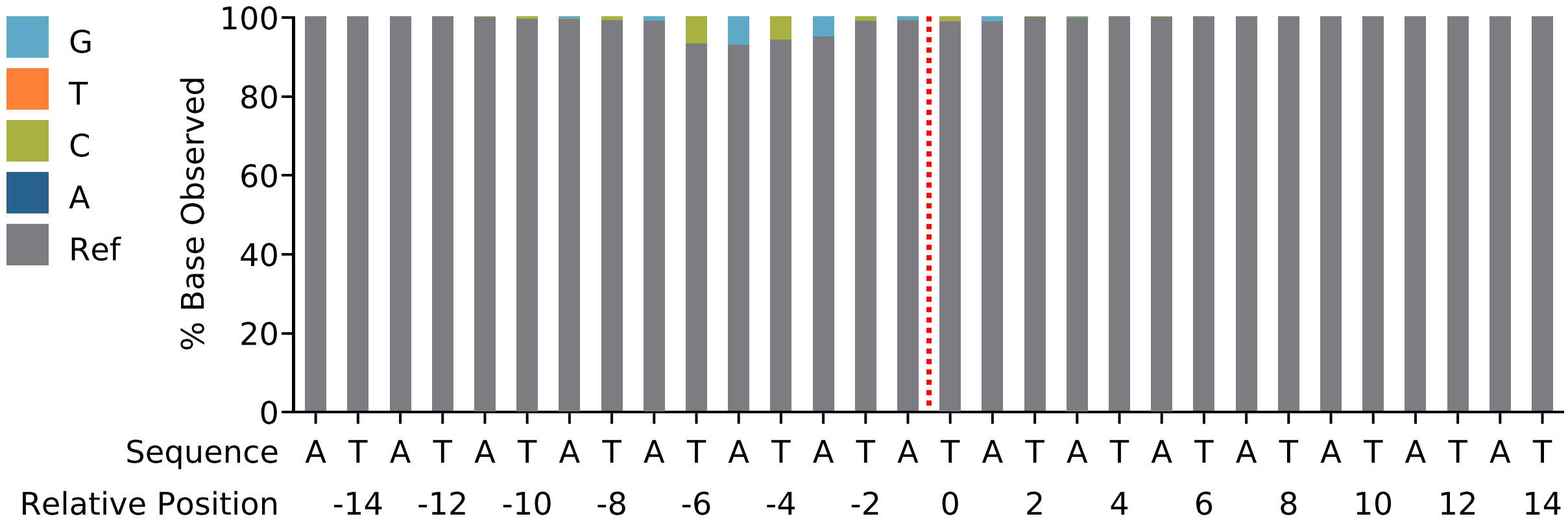

Supplement: Supplementary file 2 [file DataSheet2.pdf]
