# Supplementary material for: Combined RNA- and DNA-targeting CRISPR ribonucleoproteins enhance suppression of HBV transcripts
Source: Front Genome Ed. 2026 Sep 23;8:1824895. doi: 10.3389/fgeed.2026.1824895 (PMC13642003; doi:10.3389/fgeed.2026.1824895)

Analysis mode: CRISPAltRations 1_2_1
Start date: 2026/08/21 15:25
Targets: HBV_Cas9_EditR__ACTR3B, HBV_Cas9_EditR__CCDC60, HBV_Cas9_EditR__CLYBL, HBV_Cas9_EditR__FLYWCH1, HBV_Cas9_EditR__GALNT16, HBV_Cas9_EditR__JAM2, HBV_Cas9_EditR__LHPP, HBV_Cas9_EditR__LINC00301, HBV_Cas9_EditR__LOC105372647, HBV_Cas9_EditR__PRSS55, HBV_Cas9_EditR__RPS19BP1, HBV_Cas9_EditR__SUGP1, HBV_Cas9_EditR__TBCD, HBV_Cas9_EditR__none24, HBV_Cas9_gRNA9__DTNA, HBV_Cas9_gRNA9__LOC101929268, HBV_Cas9_gRNA9__MIR4663, HBV_Cas9_gRNA9__PARP11-AS1, HBV_Cas9_gRNA9__PDE1C, HBV_Cas9_gRNA9__PRRC2B, HBV_Cas9_gRNA9__SHISAL1, HBV_Cas9_gRNA9__none11, HBV_Cas9_gRNA9__none12, HBV_Cas9_gRNA9__none13, HBV_Cas9_gRNA9__none14, HBV_Cas9_gRNA9__none15, HBV_Cas9_gRNA9__none16, HBV_Cas9_gRNA9__none17, HBV_Cas9_gRNA9__none18, HBV_Cas9_gRNA9__none19, HBV_Cas9_gRNA9__none20, HBV_Cas9_gRNA9__none21, HBV_Cas9_gRNA9__none22

Metrics

	Group ID	Treated	Total Reads	Percent Edited	Percent Unedited	Percent NHEJ	Percent Perfect HDR	Percent Imperfect HDR	Percent HDR	Percent Other	Percent In Frame	Percent Frameshift	Percent Indels	Percent Insertions	Percent Templated Insertion	Percent GC-Insertion	Percent Deletions
**HBV_Cas9_EditR__ACTR3B**	HBV_Fig3	TRUE	7158	2.44	97.56	0.08	0.0	0.0	0.0	2.36	99.92	0.08	0.08	0.0	0.0	0.0	0.08
**HBV_Cas9_EditR__CCDC60**	HBV_Fig3	TRUE	2652	1.7	98.3	0.0	0.0	0.0	0.0	1.7	100.0	0.0	0.0	0.0	0.0	0.0	0.0
**HBV_Cas9_EditR__CLYBL**	HBV_Fig3	TRUE	17142	2.11	97.89	0.0	0.0	0.0	0.0	2.11	100.0	0.0	0.0	0.0	0.0	0.0	0.0
**HBV_Cas9_EditR__FLYWCH1**	HBV_Fig3	TRUE	11873	2.4	97.6	0.12	0.0	0.0	0.0	2.28	99.88	0.12	0.12	0.01	100.0	0.0	0.11
**HBV_Cas9_EditR__GALNT16**	HBV_Fig3	TRUE	69421	2.21	97.79	0.18	0.0	0.0	0.0	2.03	99.82	0.18	0.18	0.02	93.75	0.0	0.16
**HBV_Cas9_EditR__JAM2**	HBV_Fig3	TRUE	50714	2.82	97.18	0.06	0.0	0.0	0.0	2.76	99.95	0.05	0.06	0.01	100.0	0.0	0.05
**HBV_Cas9_EditR__LHPP**	HBV_Fig3	TRUE	12495	1.55	98.45	0.0	0.0	0.0	0.0	1.55	100.0	0.0	0.0	0.0	0.0	0.0	0.0
**HBV_Cas9_EditR__LINC00301**	HBV_Fig3	TRUE	33845	100.0	0.0	0.39	0.0	0.0	0.0	99.61	99.97	0.03	0.39	0.0	0.0	0.0	0.39
**HBV_Cas9_EditR__LOC105372647**	HBV_Fig3	TRUE	865936	88.7	11.3	0.72	0.0	0.0	0.0	87.98	99.32	0.68	0.72	0.42	3.22	0.0	0.32
**HBV_Cas9_EditR__PRSS55**	HBV_Fig3	TRUE	62415	99.94	0.06	0.07	0.0	0.0	0.0	99.87	99.93	0.07	0.07	0.0	100.0	0.0	0.07
**HBV_Cas9_EditR__RPS19BP1**	HBV_Fig3	TRUE	13047	2.21	97.79	0.05	0.0	0.0	0.0	2.16	99.95	0.05	0.05	0.0	0.0	0.0	0.05
**HBV_Cas9_EditR__SUGP1**	HBV_Fig3	TRUE	13505	2.68	97.32	0.16	0.0	0.0	0.0	2.52	99.84	0.16	0.16	0.01	100.0	0.0	0.15
**HBV_Cas9_EditR__TBCD**	HBV_Fig3	TRUE	8555	2.55	97.45	0.07	0.0	0.0	0.0	2.48	99.93	0.07	0.07	0.0	0.0	0.0	0.07
**HBV_Cas9_EditR__none24**	HBV_Fig3	TRUE	25676	2.93	97.07	0.03	0.0	0.0	0.0	2.9	99.97	0.03	0.03	0.0	0.0	0.0	0.03
**HBV_Cas9_gRNA9__DTNA**	HBV_Fig3	TRUE	20613	68.53	31.47	0.02	0.0	0.0	0.0	68.51	99.98	0.02	0.02	0.02	0.0	0.0	0.0
**HBV_Cas9_gRNA9__LOC101929268**	HBV_Fig3	TRUE	44100	2.98	97.02	0.02	0.0	0.0	0.0	2.96	99.98	0.02	0.02	0.0	0.0	0.0	0.02
**HBV_Cas9_gRNA9__MIR4663**	HBV_Fig3	TRUE	46849	3.17	96.83	0.16	0.0	0.0	0.0	3.01	99.84	0.16	0.16	0.12	0.0	0.0	0.16
**HBV_Cas9_gRNA9__PARP11-AS1**	HBV_Fig3	TRUE	59327	2.46	97.54	0.02	0.0	0.0	0.0	2.44	99.98	0.02	0.02	0.0	0.0	0.0	0.02
**HBV_Cas9_gRNA9__PDE1C**	HBV_Fig3	TRUE	37893	2.51	97.49	0.14	0.0	0.0	0.0	2.37	99.86	0.14	0.14	0.02	100.0	0.0	0.12
**HBV_Cas9_gRNA9__PRRC2B**	HBV_Fig3	TRUE	34423	2.31	97.69	0.22	0.0	0.0	0.0	2.09	99.78	0.22	0.22	0.06	95.45	0.0	0.16
**HBV_Cas9_gRNA9__SHISAL1**	HBV_Fig3	TRUE	44948	2.69	97.31	0.14	0.0	0.0	0.0	2.55	99.86	0.14	0.14	0.04	81.25	0.0	0.1
**HBV_Cas9_gRNA9__none11**	HBV_Fig3	TRUE	3440	2.67	97.33	0.03	0.0	0.0	0.0	2.64	99.97	0.03	0.03	0.0	0.0	0.0	0.03
**HBV_Cas9_gRNA9__none12**	HBV_Fig3	TRUE	13832	2.43	97.57	0.08	0.0	0.0	0.0	2.35	99.96	0.04	0.08	0.0	0.0	0.0	0.08
**HBV_Cas9_gRNA9__none13**	HBV_Fig3	TRUE	985	55.63	44.37	52.39	0.0	0.0	0.0	3.24	47.72	52.28	52.39	0.0	0.0	0.0	52.39
**HBV_Cas9_gRNA9__none14**	HBV_Fig3	TRUE	46886	3.01	96.99	0.17	0.0	0.0	0.0	2.84	99.83	0.17	0.17	0.04	95.24	0.0	0.13
**HBV_Cas9_gRNA9__none15**	HBV_Fig3	TRUE	14721	2.25	97.75	0.0	0.0	0.0	0.0	2.25	100.0	0.0	0.0	0.0	0.0	0.0	0.0
**HBV_Cas9_gRNA9__none16**	HBV_Fig3	TRUE	46	0.0	100.0	0.0	0.0	0.0	0.0	0.0	100.0	0.0	0.0	0.0	0.0	0.0	0.0
**HBV_Cas9_gRNA9__none17**	HBV_Fig3	TRUE	58778	2.63	97.37	0.01	0.0	0.0	0.0	2.62	99.99	0.01	0.01	0.0	100.0	0.0	0.01
**HBV_Cas9_gRNA9__none18**	HBV_Fig3	TRUE	157803	5.86	94.14	0.87	0.0	0.0	0.0	4.99	99.13	0.87	0.87	0.01	0.0	0.0	0.86
**HBV_Cas9_gRNA9__none19**	HBV_Fig3	TRUE	32830	2.54	97.46	0.04	0.0	0.0	0.0	2.5	99.96	0.04	0.04	0.0	0.0	0.0	0.04
**HBV_Cas9_gRNA9__none20**	HBV_Fig3	TRUE	42561	28.48	71.52	0.03	0.0	0.0	0.0	28.45	99.98	0.02	0.03	0.01	0.0	0.0	0.02

**HBV_Cas9_gRNA9__none21**	HBV_Fig3	TRUE	35196	2.7	97.3	0.3	0.0	0.0	0.0	2.4	99.7	0.3	0.3	0.04	100.0	0.0	0.26	[illegible]
**HBV_Cas9_gRNA9__none22**	HBV_Fig3	TRUE	34843	2.7	97.3	0.06	0.0	0.0	0.0	2.64	99.95	0.05	0.06	0.01	0.0	0.0	0.05	[illegible]

Percentages

	% Edited	*% Repair Pathways*	*% Frameshift*
HBV_Cas9_EditR__ACTR3B	97.56%	97.56%	99.92%
HBV_Cas9_EditR__CCDC60	98.3%	98.3%	100.0%
HBV_Cas9_EditR__CLYBL	97.89%	97.89%	100.0%
HBV_Cas9_EditR__FLYWCH1	97.6%	97.6%	99.88%
HBV_Cas9_EditR__GALNT16	97.79%	97.79%	99.82%
HBV_Cas9_EditR__JAM2	97.18%	97.18%	99.95%
HBV_Cas9_EditR__LHPP	98.45%	98.45%	100.0%
HBV_Cas9_EditR__LINC00301	100.0%	99.61%	99.97%
HBV_Cas9_EditR__LOC105372647	1.3[illegible] 88.7%	1.3[illegible] 87.98%	99.32%
HBV_Cas9_EditR__PRSS55	99.94%	99.87%	99.93%
HBV_Cas9_EditR__RPS19BP1	97.79%	97.79%	99.95%
HBV_Cas9_EditR__SUGP1	97.32%	97.32%	99.84%
HBV_Cas9_EditR__TBCD	97.45%	97.45%	99.93%
HBV_Cas9_EditR__none24	97.07%	97.07%	99.97%
HBV_Cas9_gRNA9__DTNA	31.47% 68.53%	31.47% 68.51%	99.98%
HBV_Cas9_gRNA9__LOC101929268	97.02%	97.02%	99.98%
HBV_Cas9_gRNA9__MIR4663	96.83%	96.83%	99.84%
HBV_Cas9_gRNA9__PARP11-AS1	97.54%	97.54%	99.98%
HBV_Cas9_gRNA9__PDE1C	97.49%	97.49%	99.86%
HBV_Cas9_gRNA9__PRRC2B	97.69%	97.69%	99.78%
HBV_Cas9_gRNA9__SHISAL1	97.31%	97.31%	99.86%
HBV_Cas9_gRNA9__none11	97.33%	97.33%	99.97%
HBV_Cas9_gRNA9__none12	97.57%	97.57%	99.96%
HBV_Cas9_gRNA9__none13	44.37% 55.63%	44.37% 52.39%	47.72% 52.28%
HBV_Cas9_gRNA9__none14	96.99%	96.99%	99.83%
HBV_Cas9_gRNA9__none15	97.75%	97.75%	100.0%

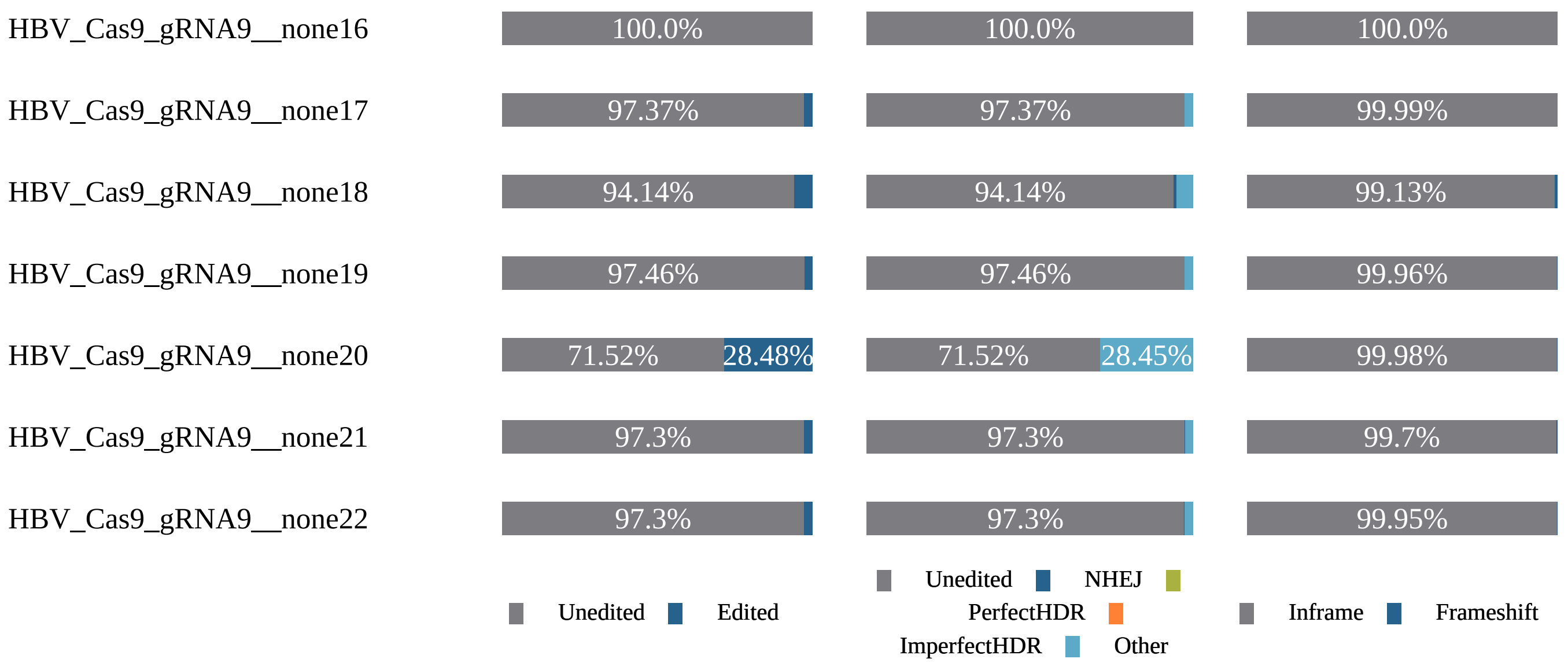

Insertions

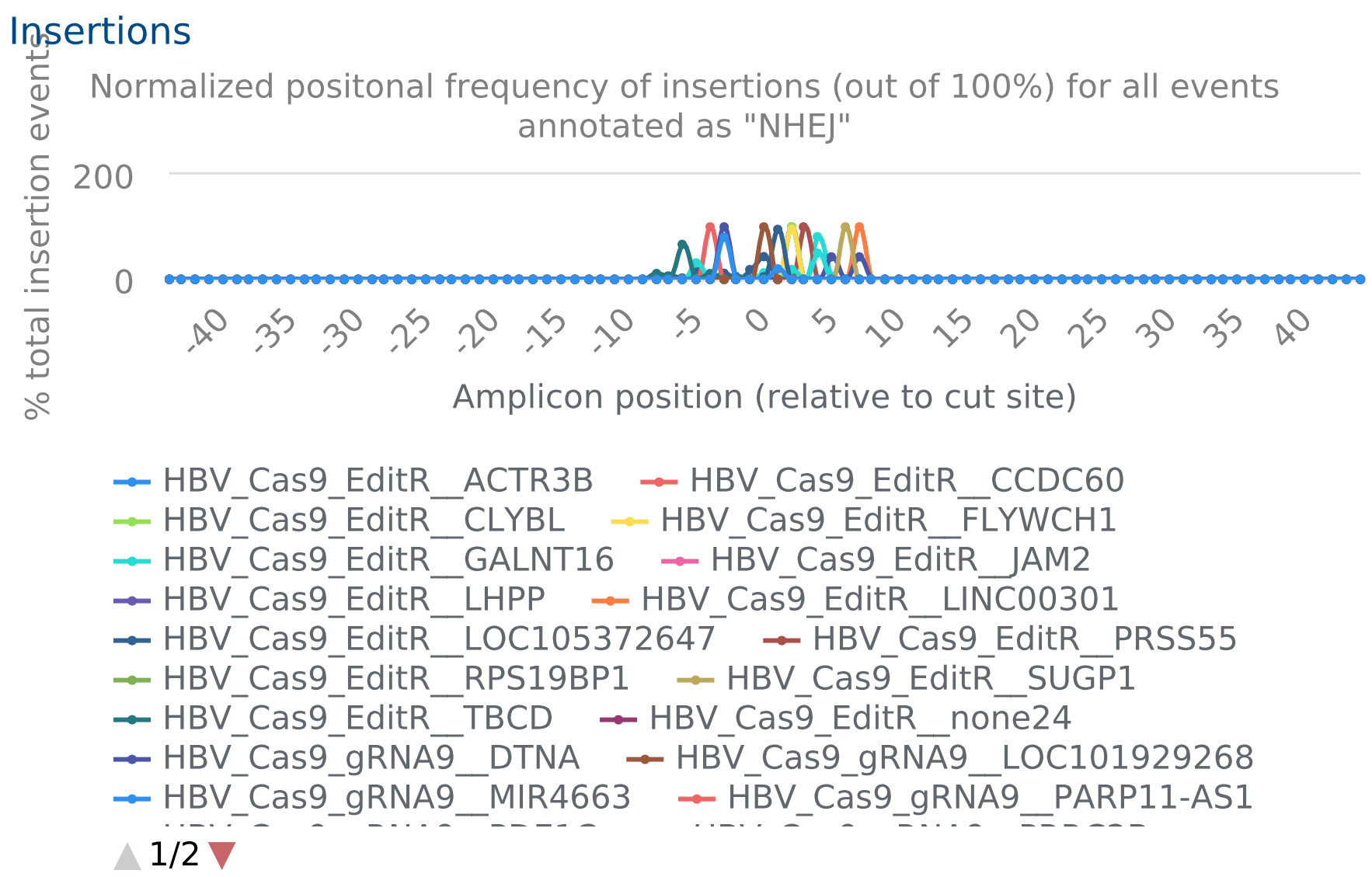

Deletions

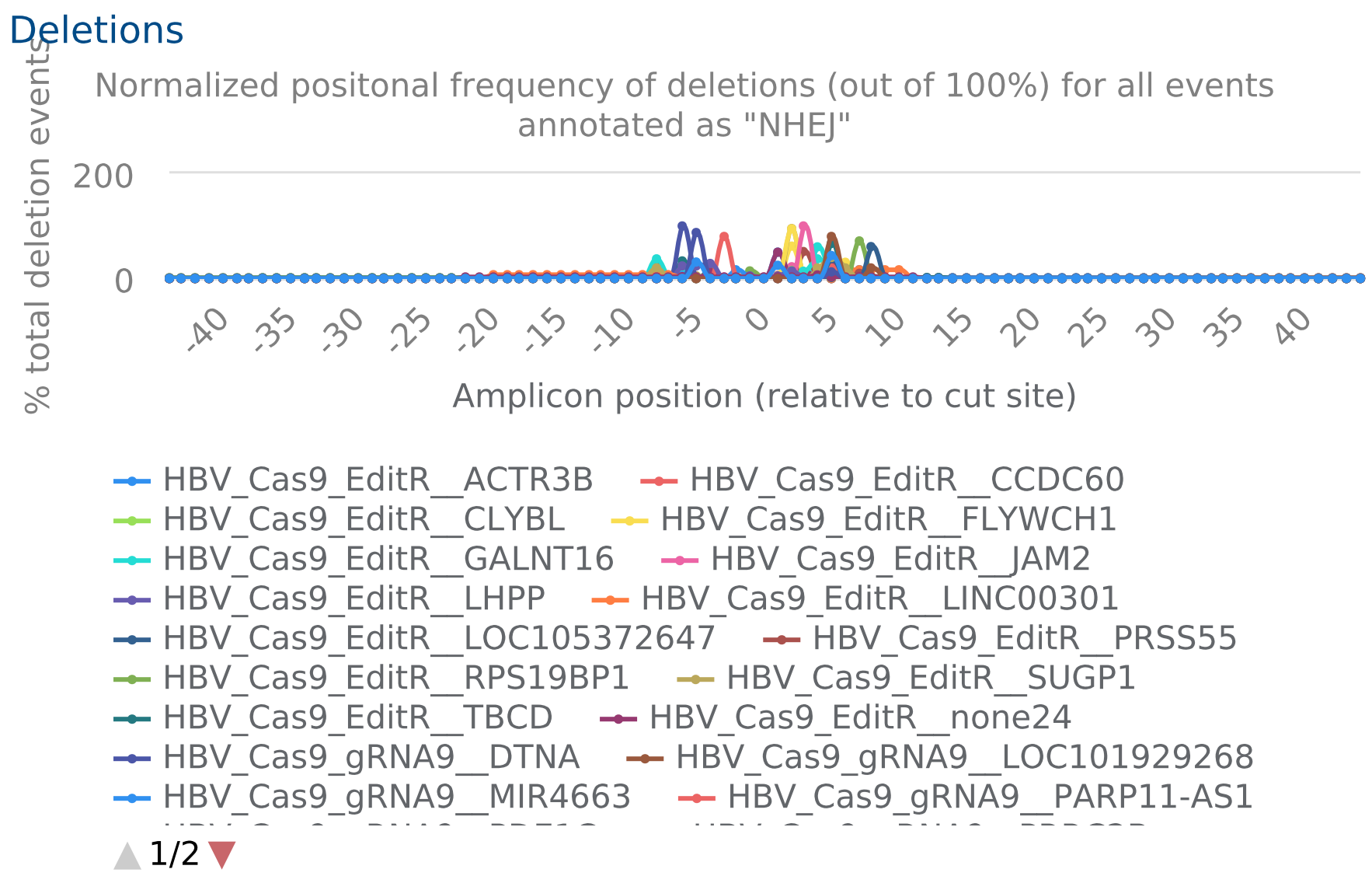

Indel Frequencies

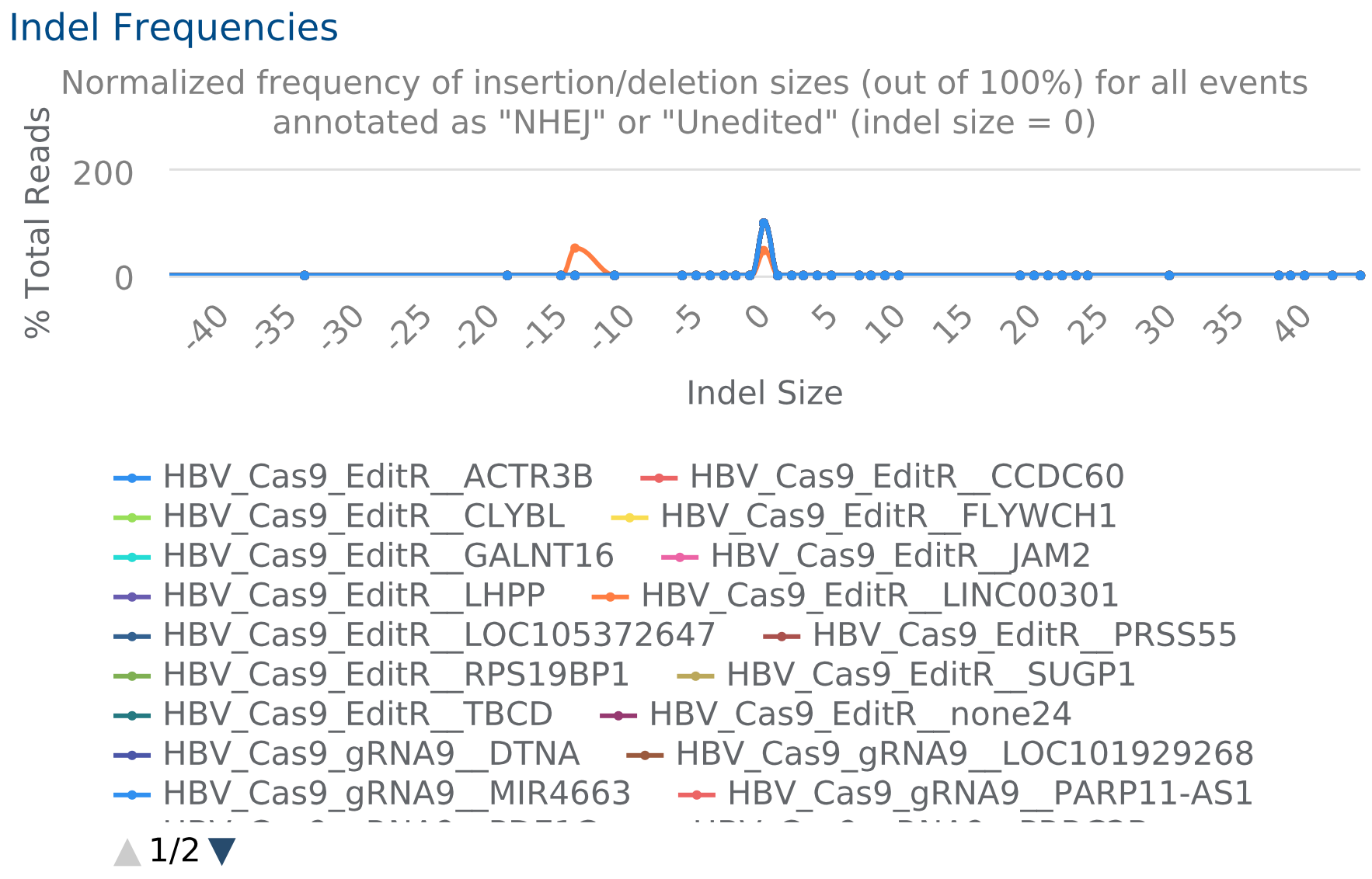

SNP Graph

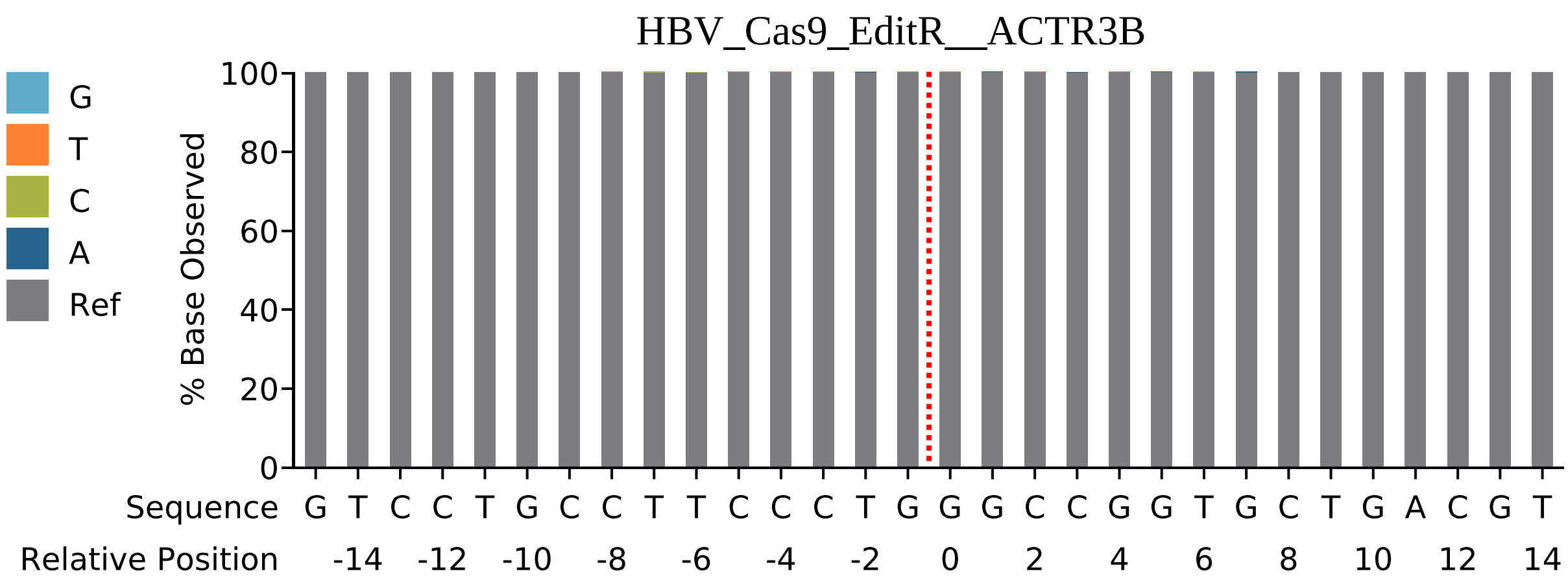

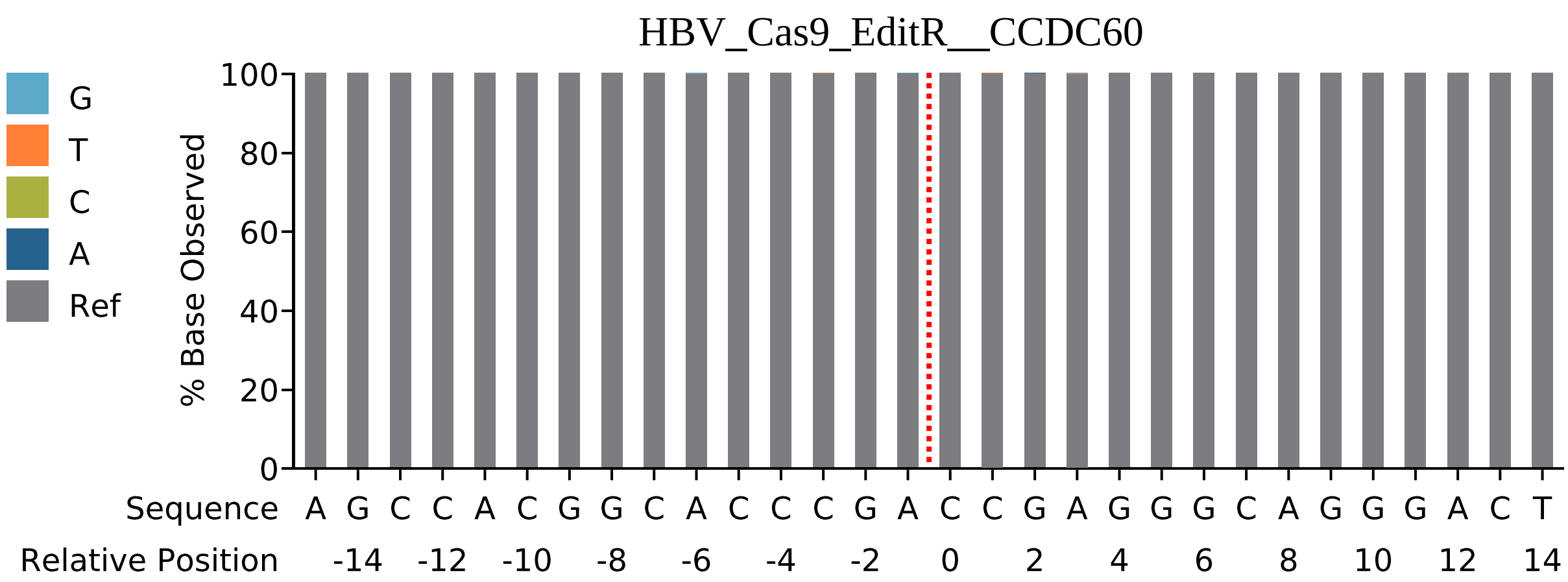

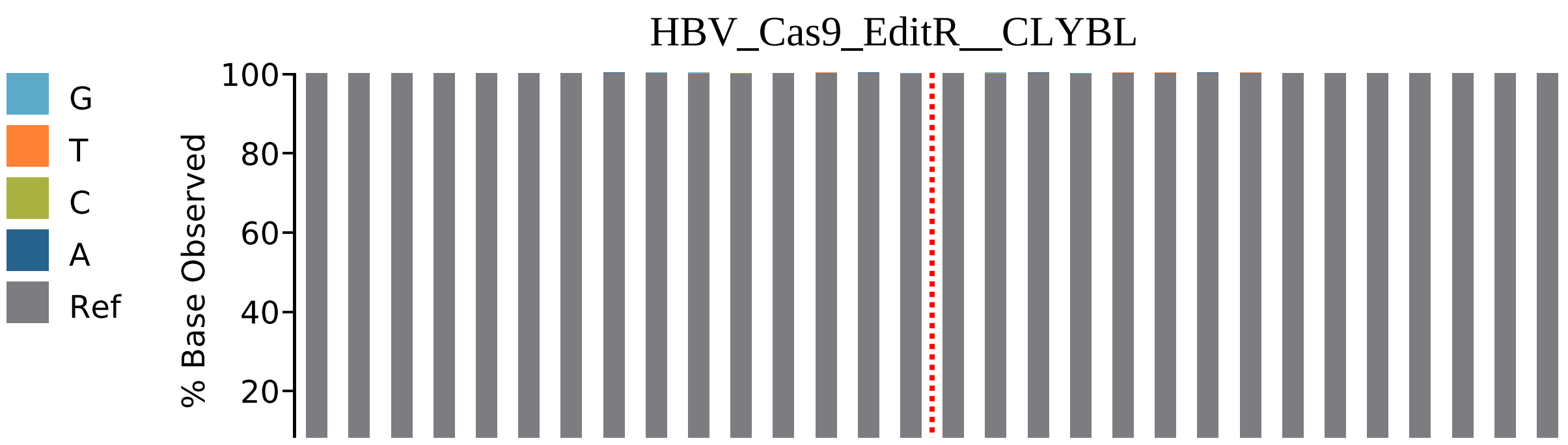

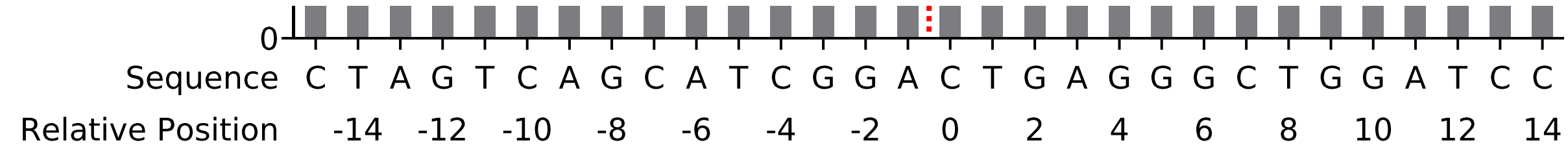

HBV_Cas9_EditR__FLYWCH1

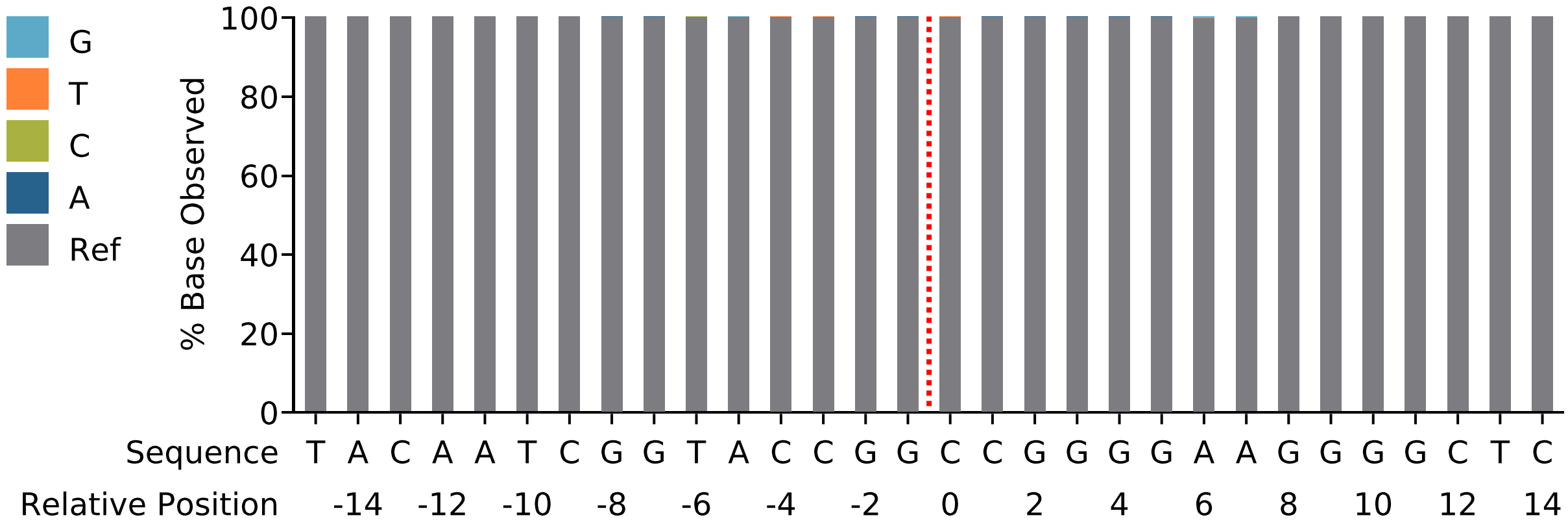

HBV_Cas9_EditR__GALNT16

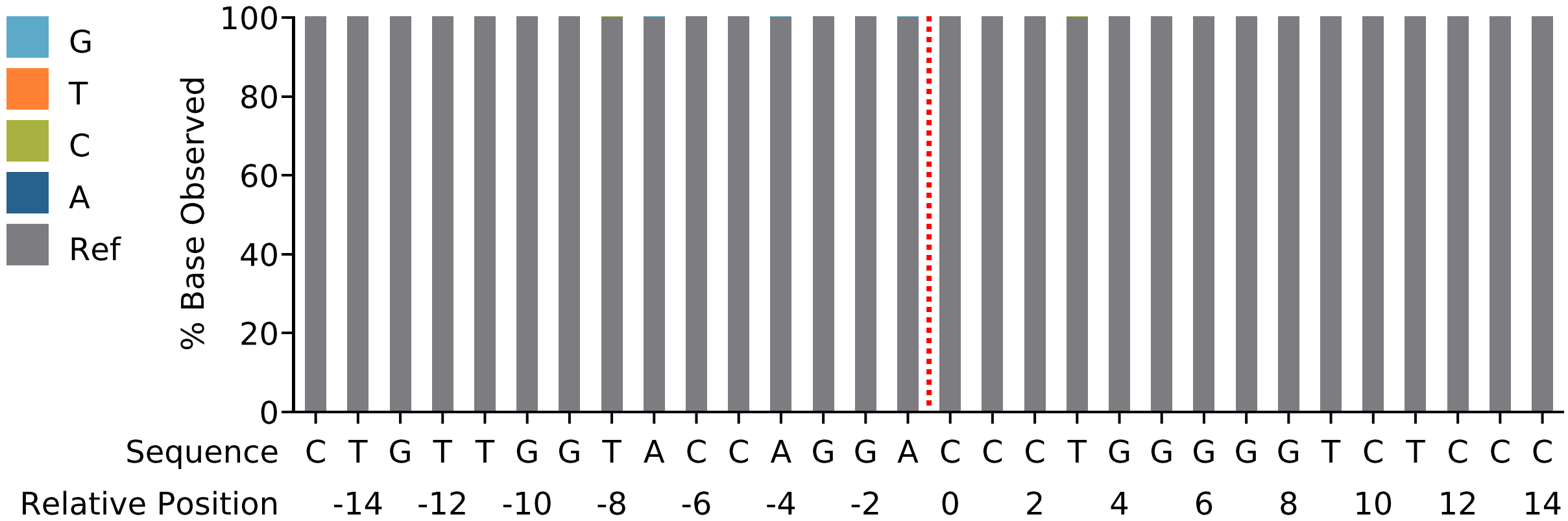

HBV_Cas9_EditR__JAM2

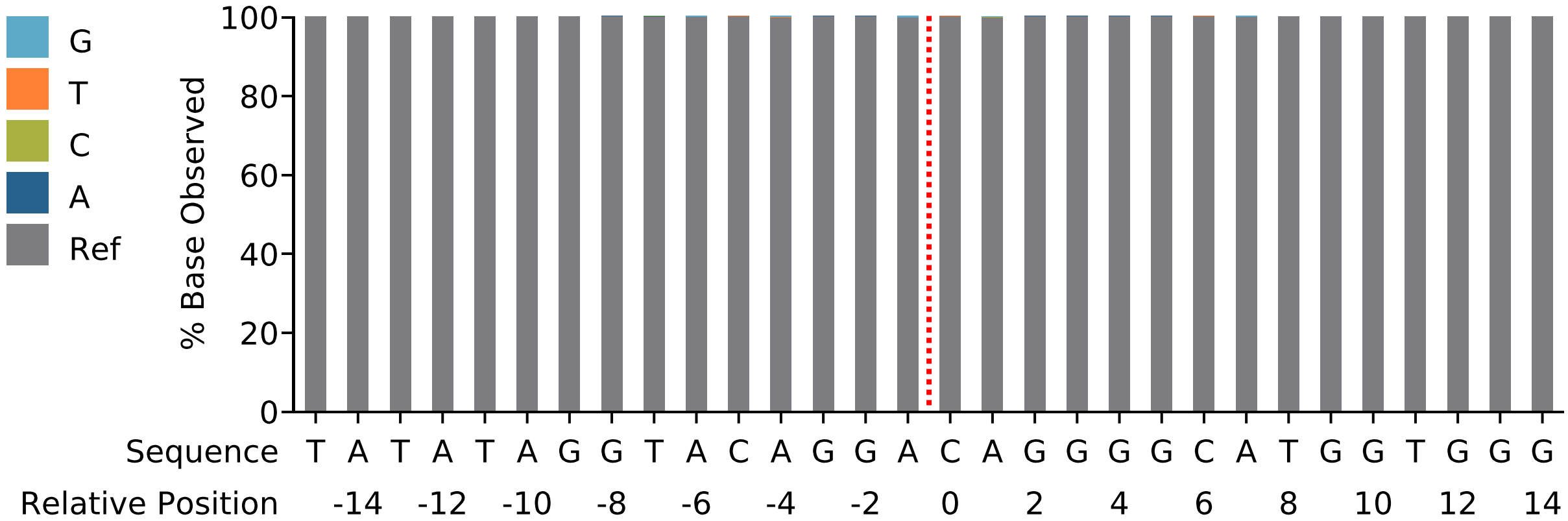

HBV_Cas9_EditR__LHPP

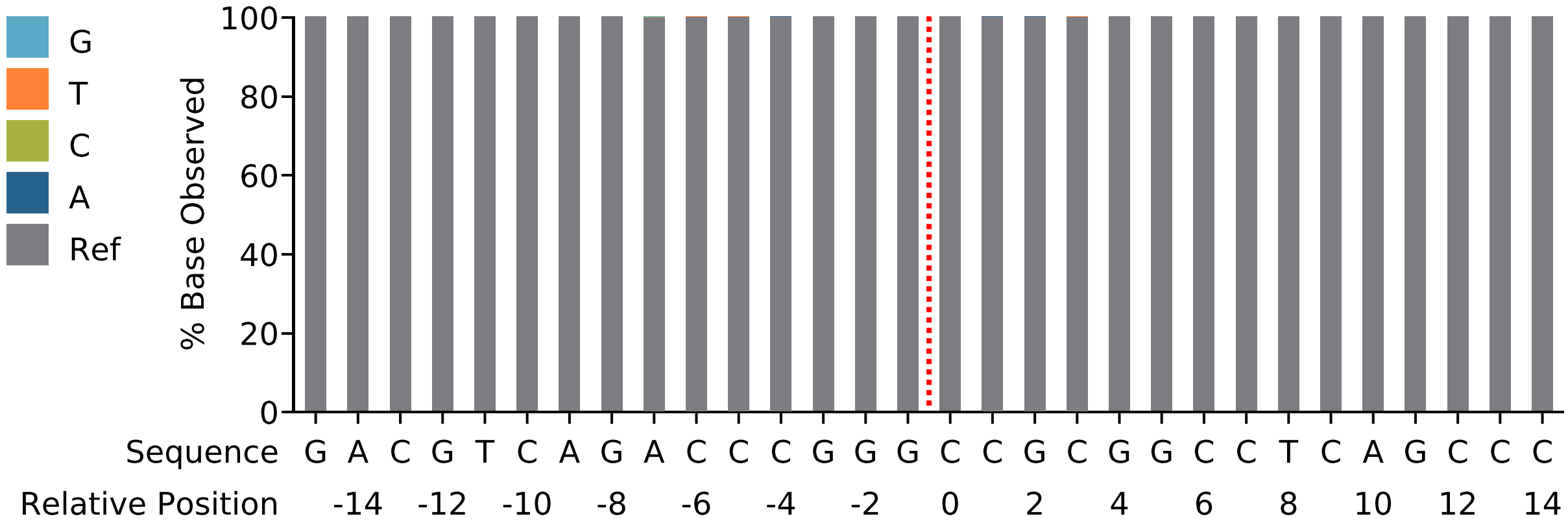

HBV_Cas9_EditR__LINC00301

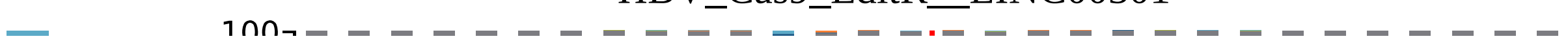

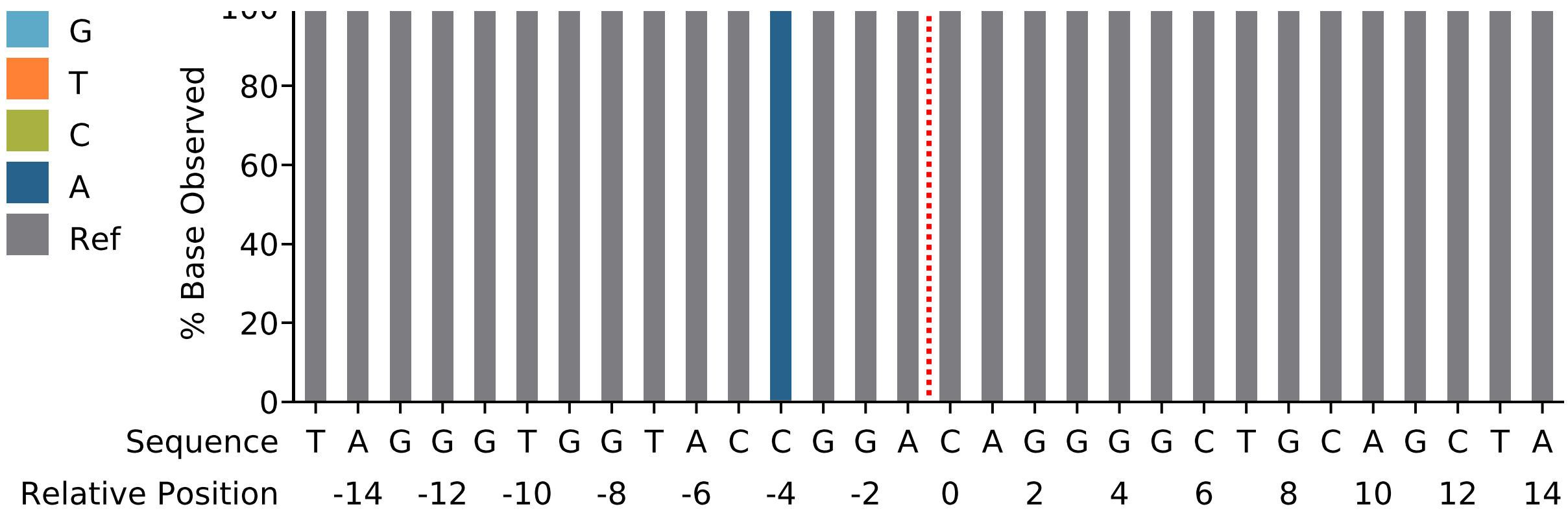

HBV_Cas9_EditR__LOC105372647

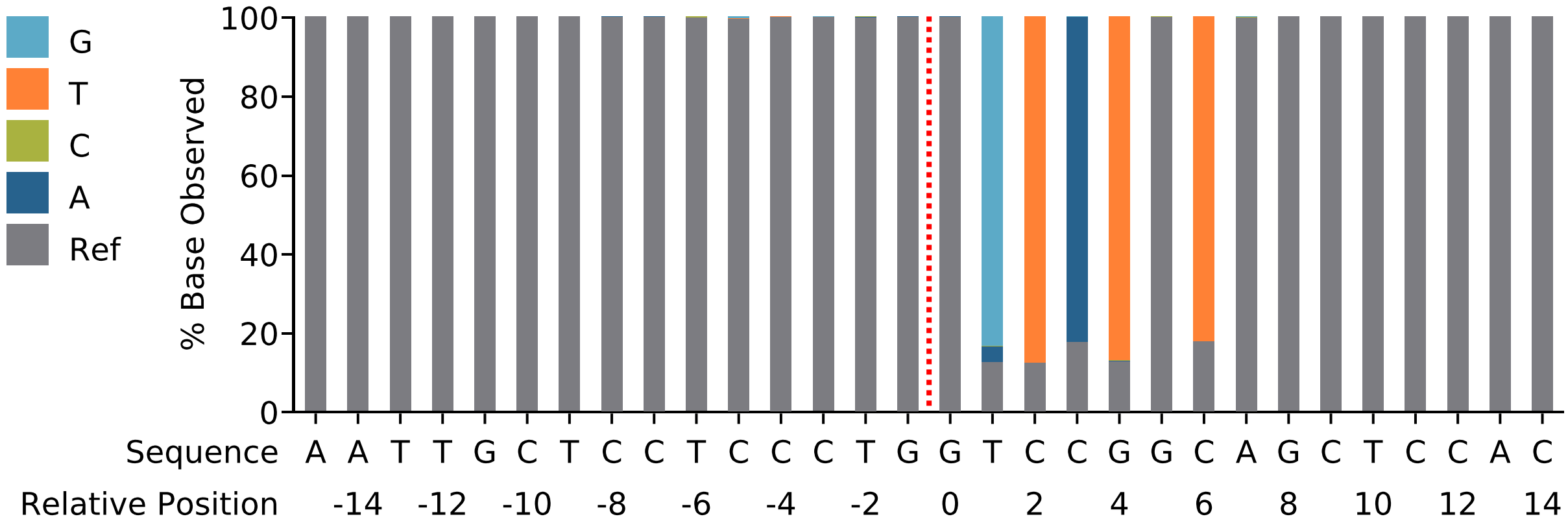

HBV_Cas9_EditR__PRSS55

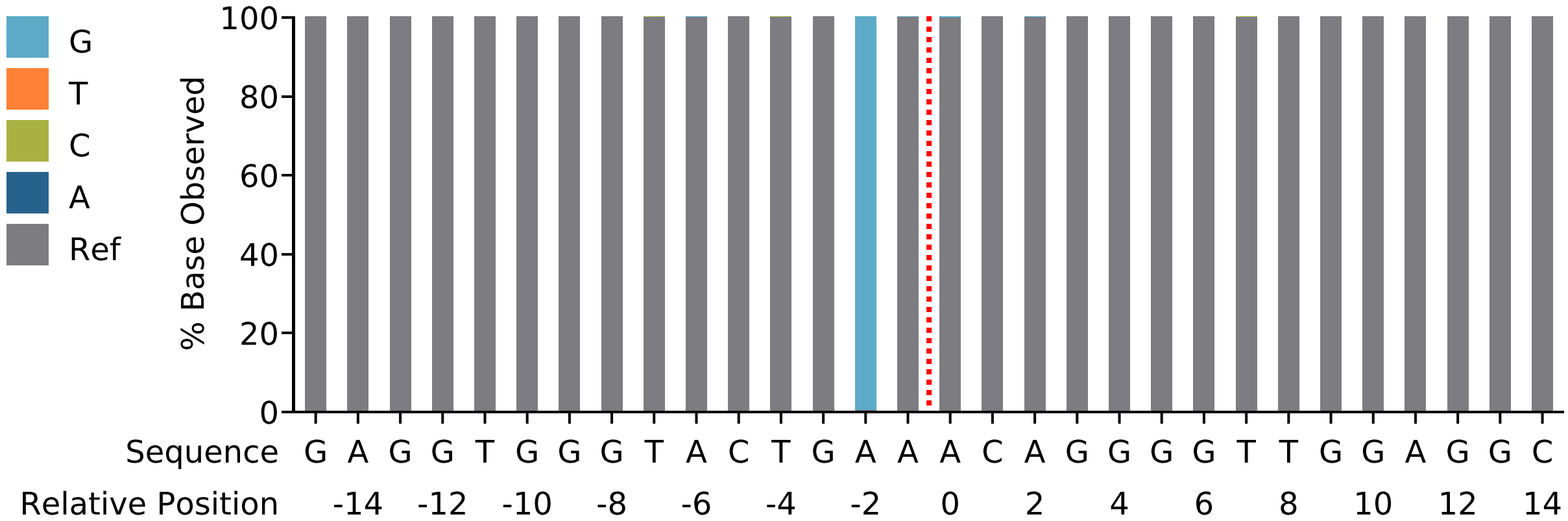

HBV_Cas9_EditR__RPS19BP1

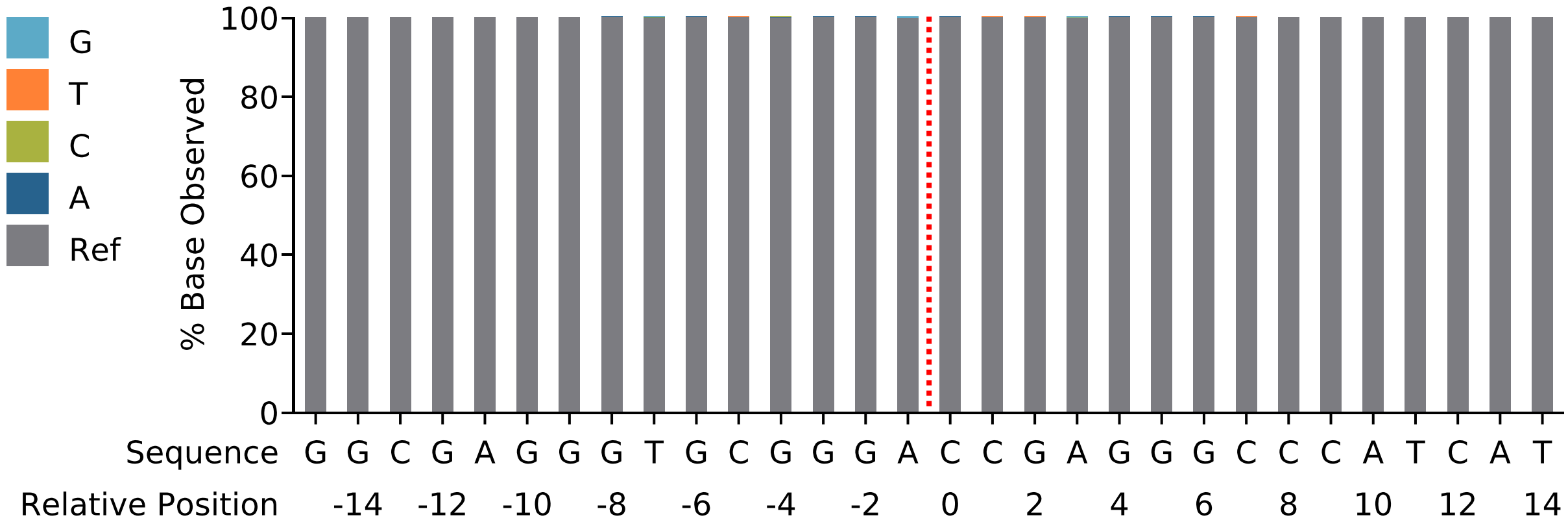

HBV_Cas9_EditR__SUGP1

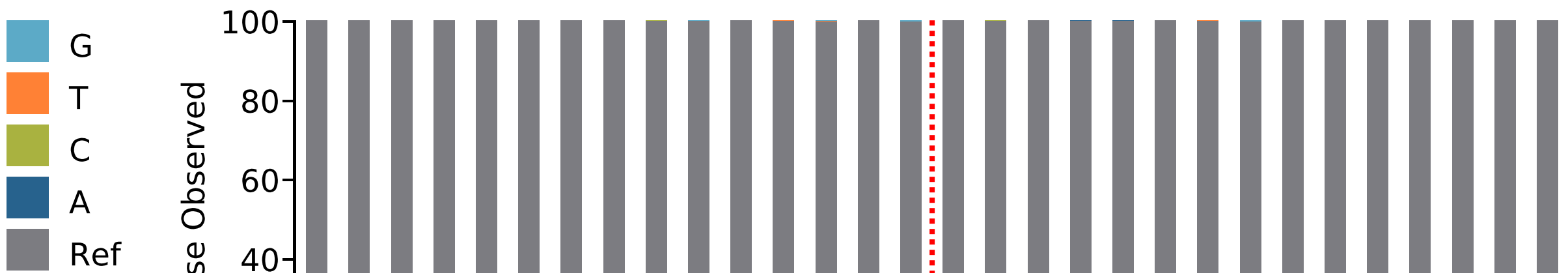

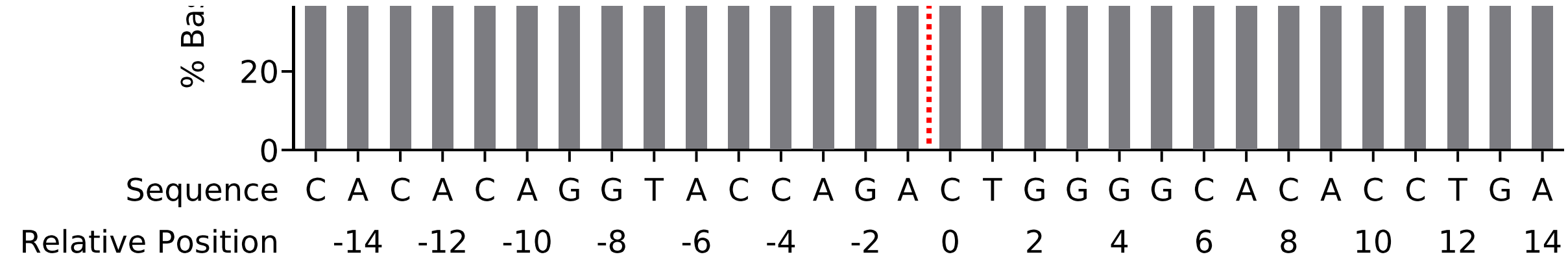

HBV_Cas9_EditR__TBCD

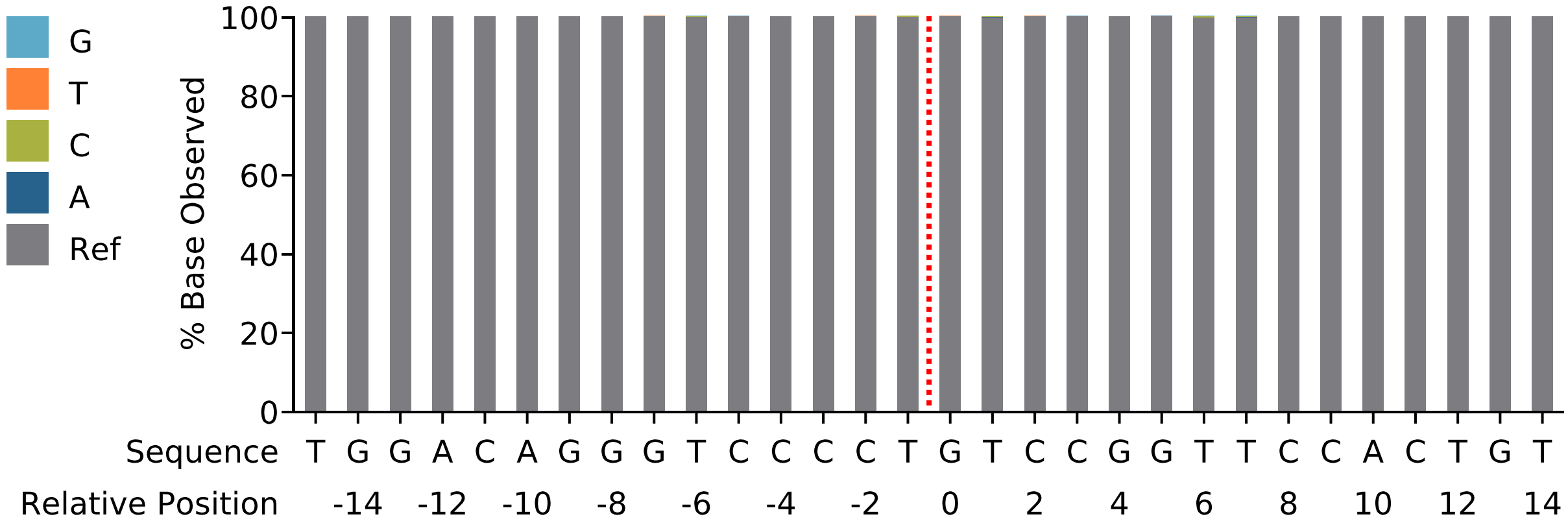

HBV_Cas9_EditR__none24

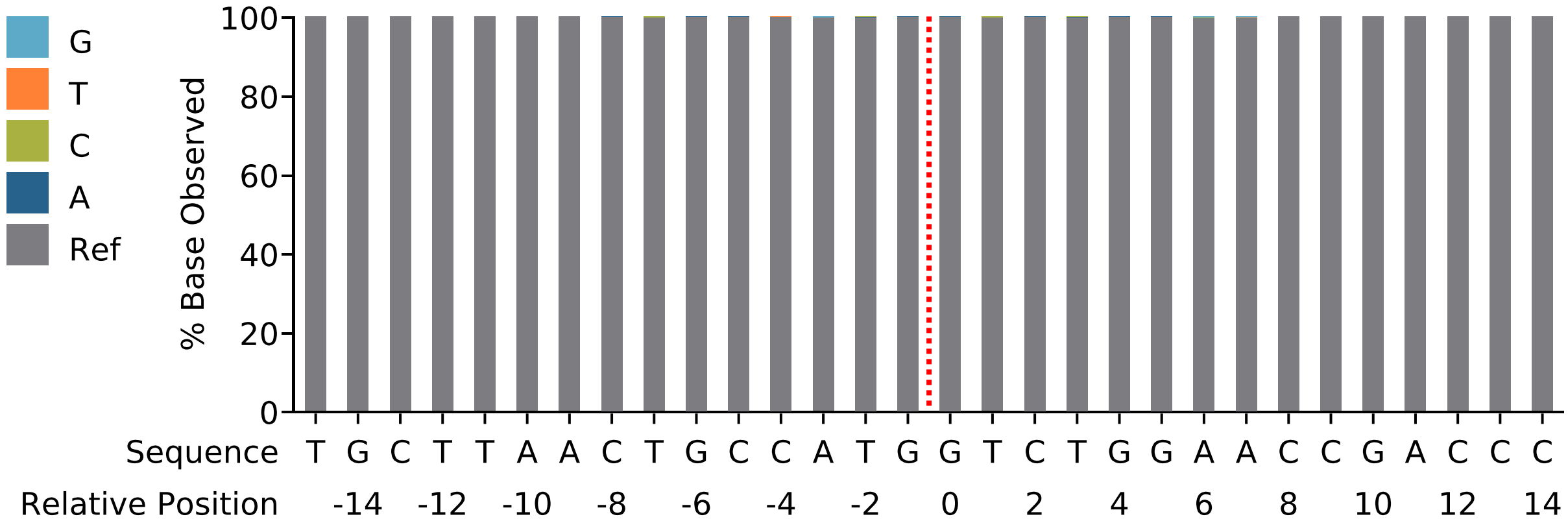

HBV_Cas9_gRNA9__DTNA

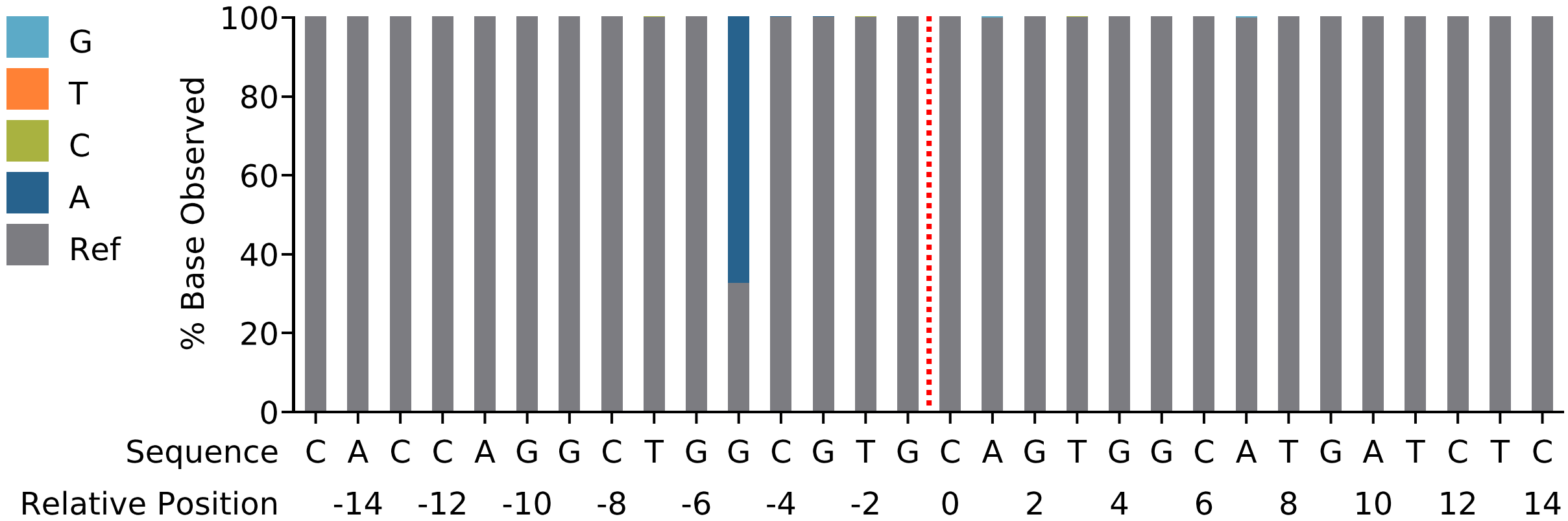

HBV_Cas9_gRNA9__LOC101929268

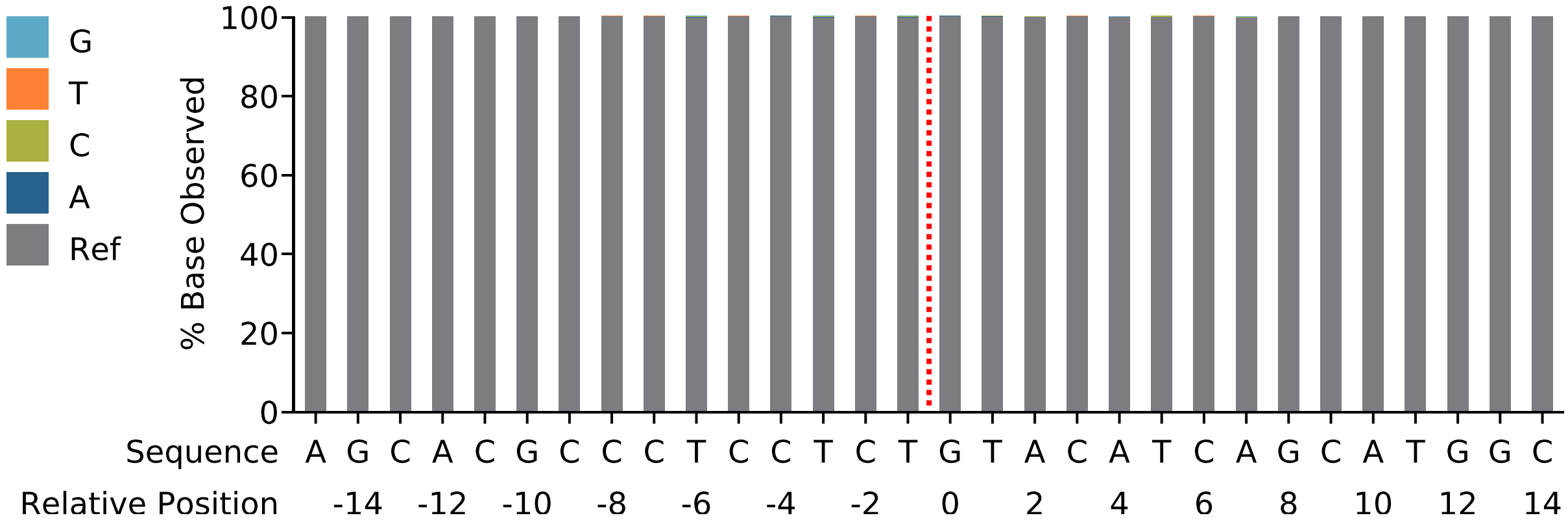

HBV_Cas9_gRNA9__MIR4663

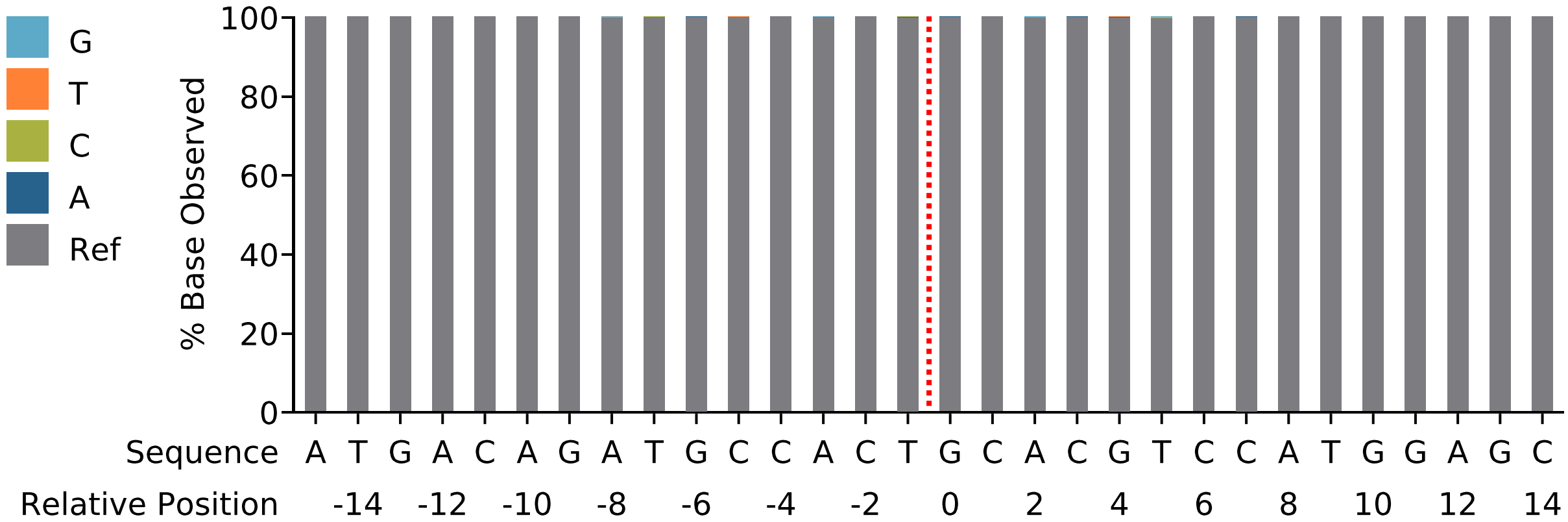

HBV_Cas9_gRNA9__PARP11-AS1

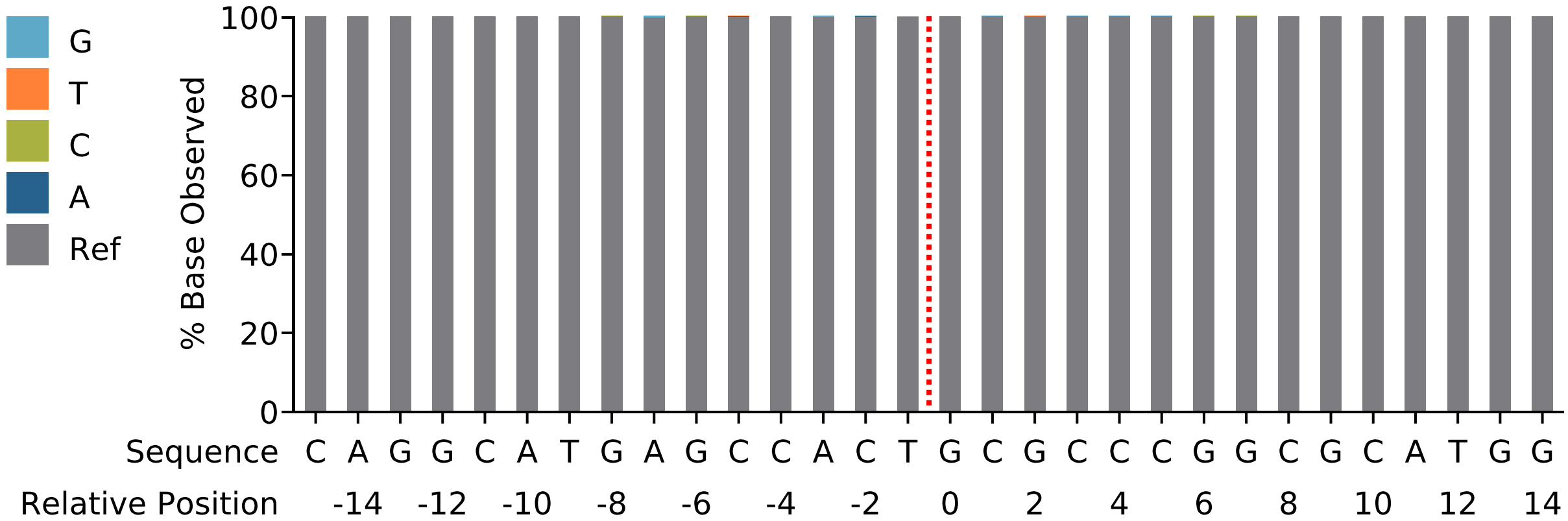

HBV_Cas9_gRNA9__PDE1C

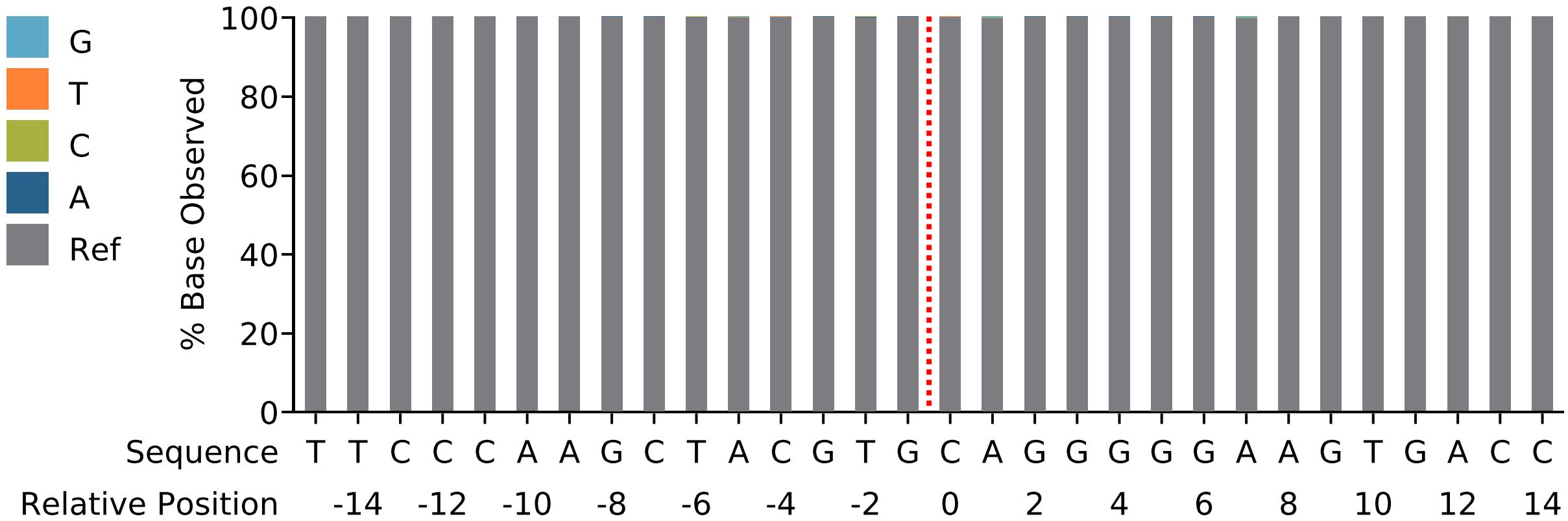

HBV_Cas9_gRNA9__PRRC2B

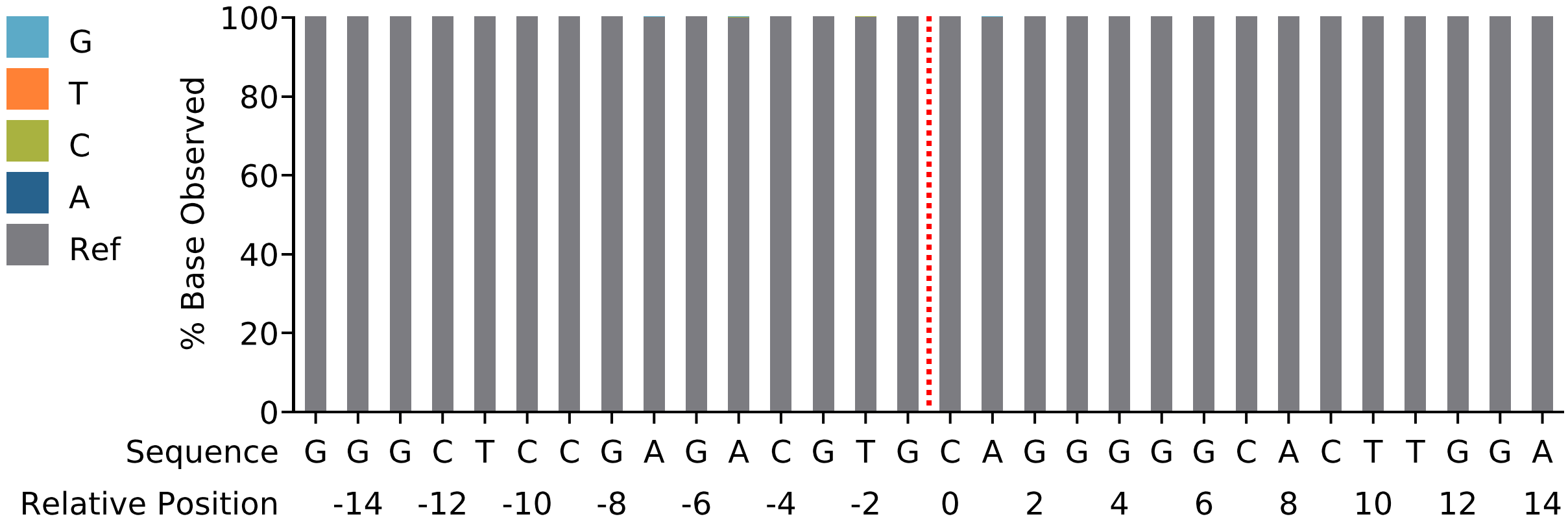

HBV_Cas9_gRNA9__SHISAL1

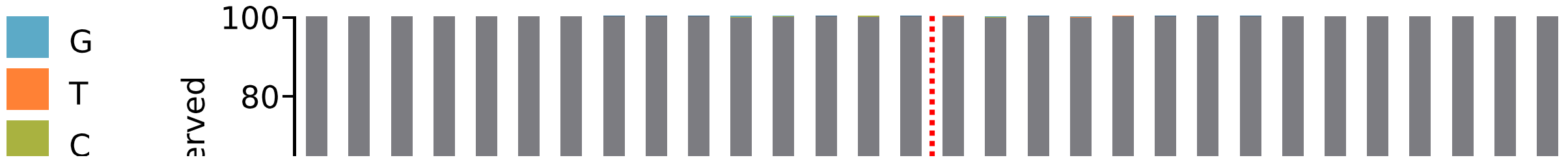

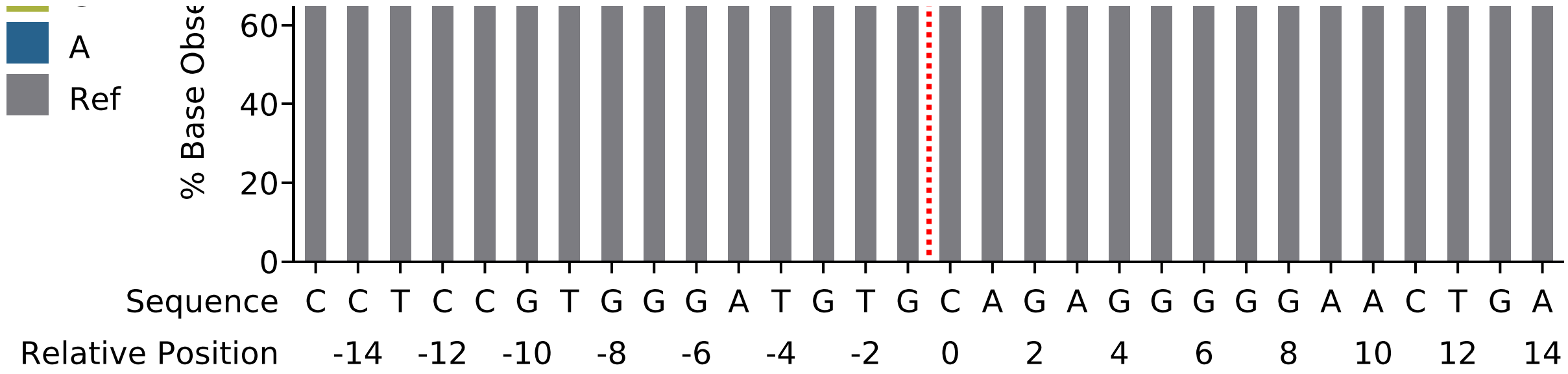

HBV_Cas9_gRNA9__none11

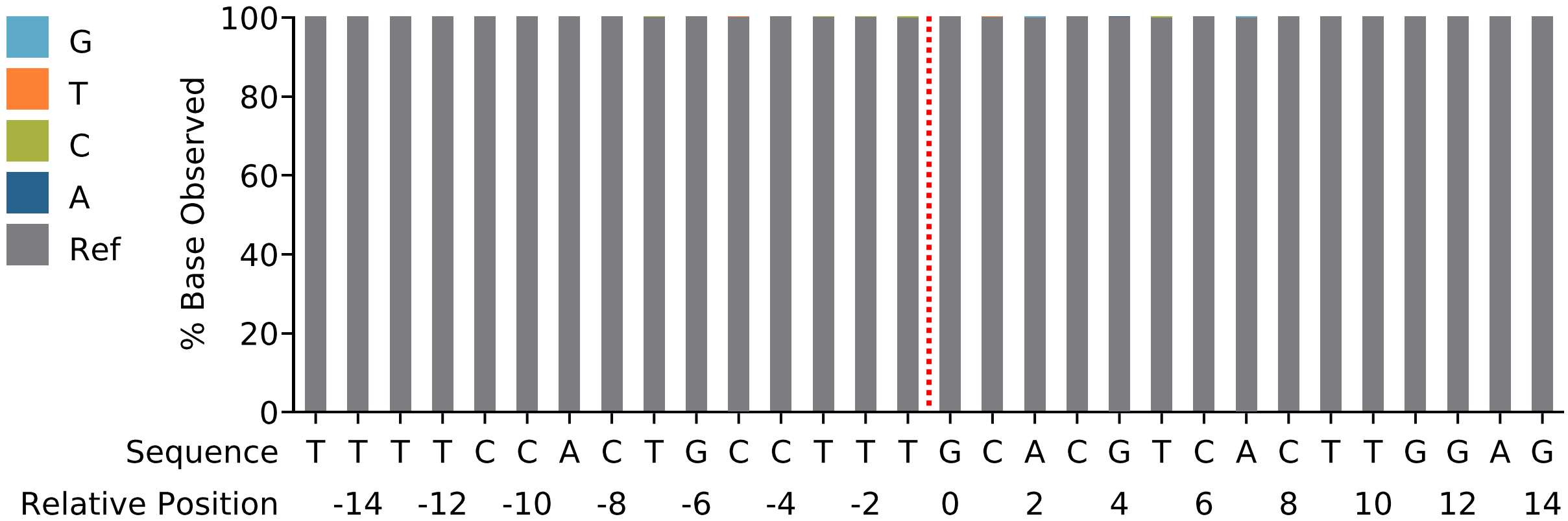

HBV_Cas9_gRNA9__none12

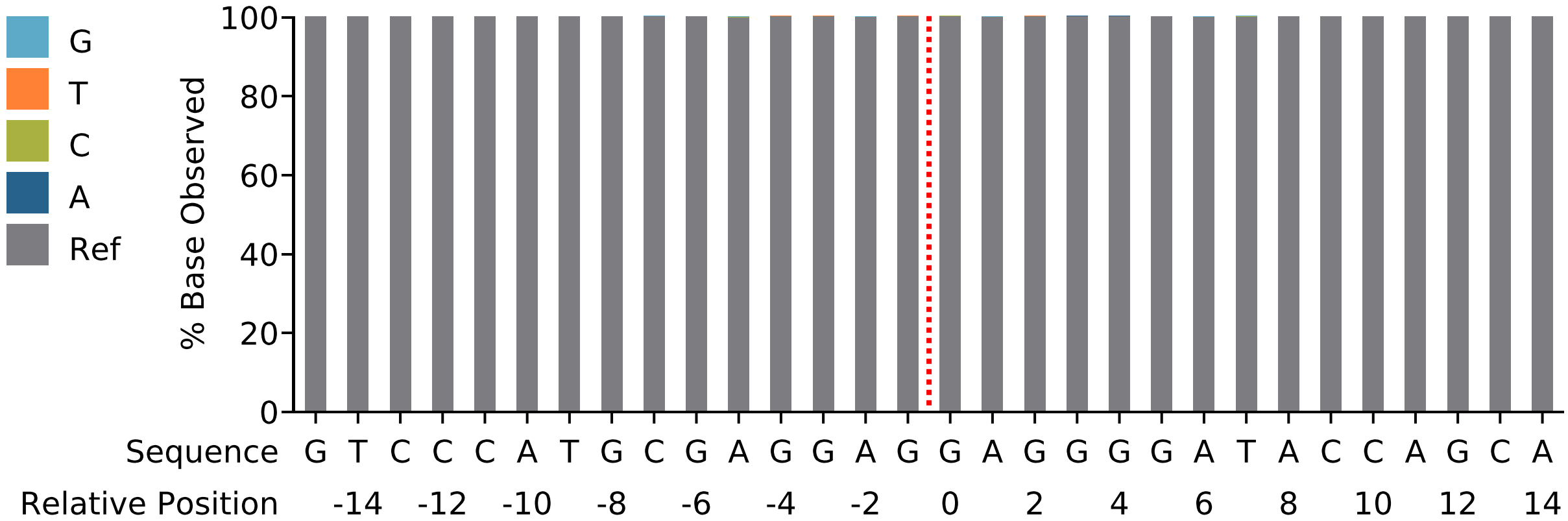

HBV_Cas9_gRNA9__none13

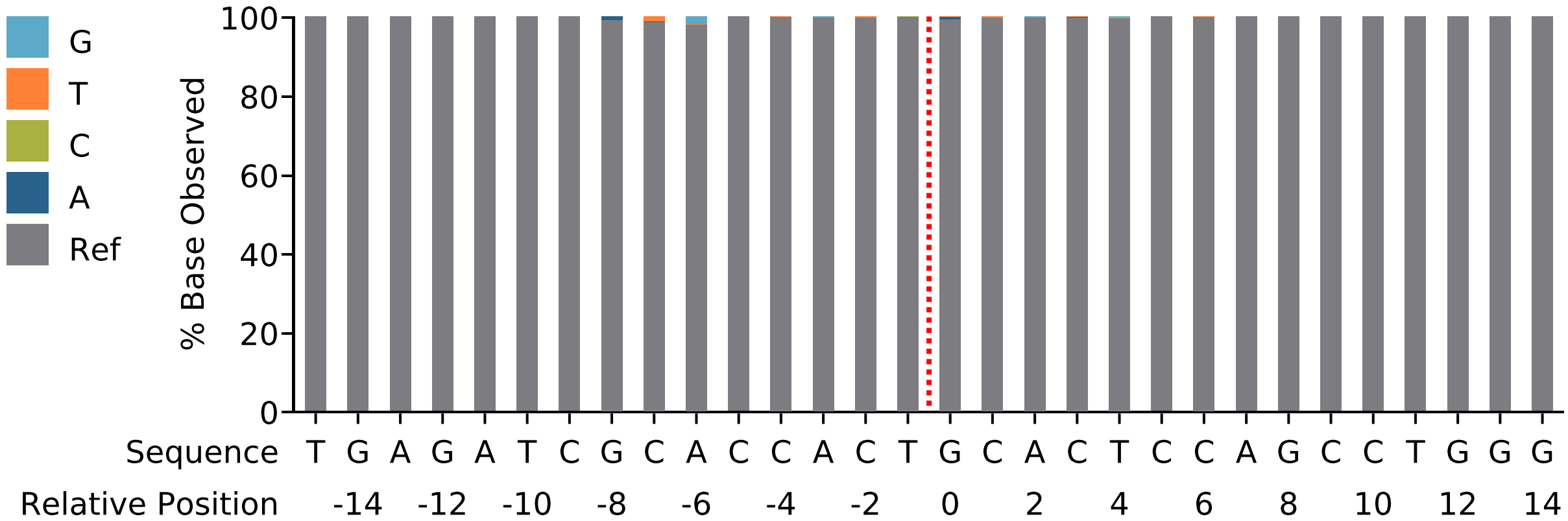

HBV_Cas9_gRNA9__none14

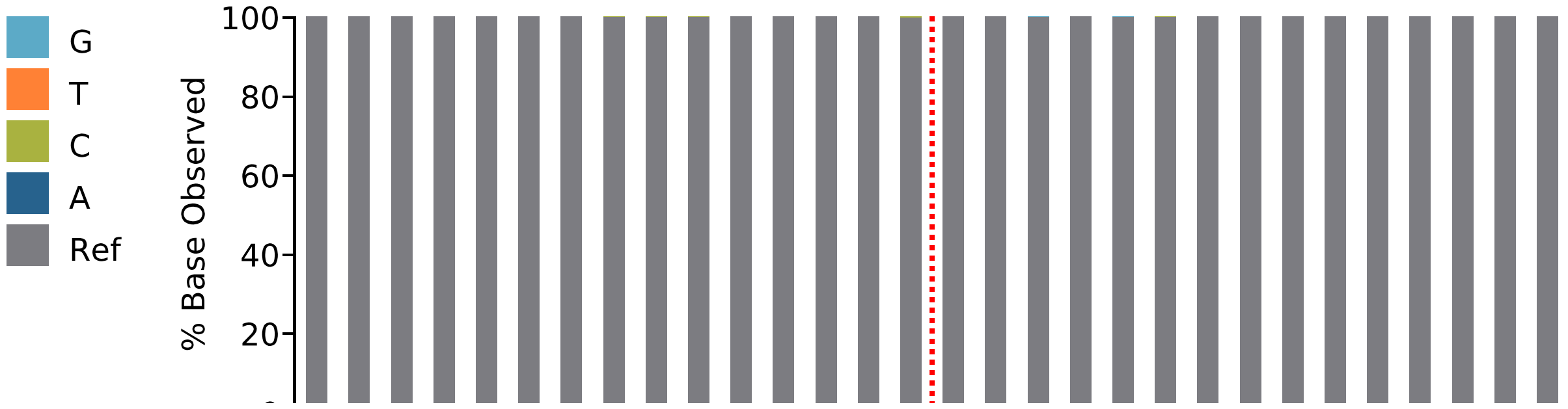

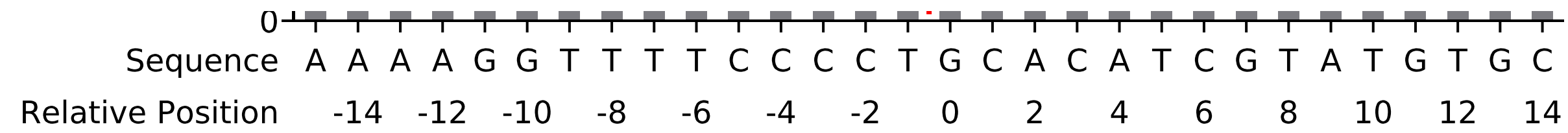

HBV_Cas9_gRNA9__none15

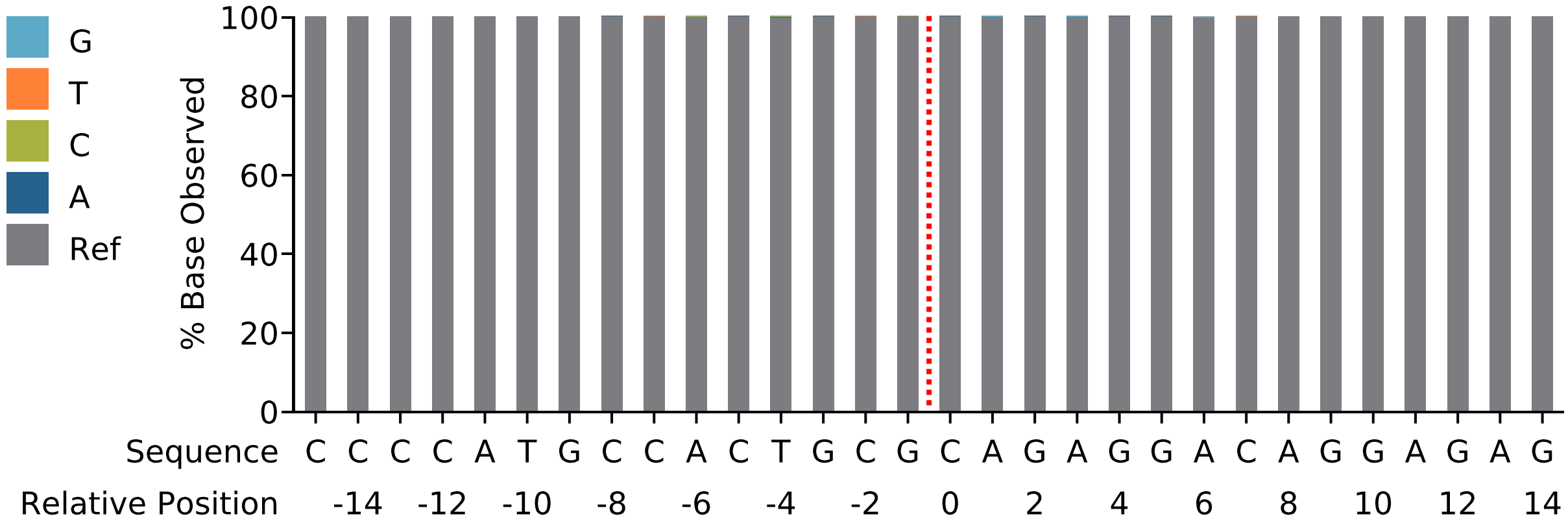

HBV_Cas9_gRNA9__none16

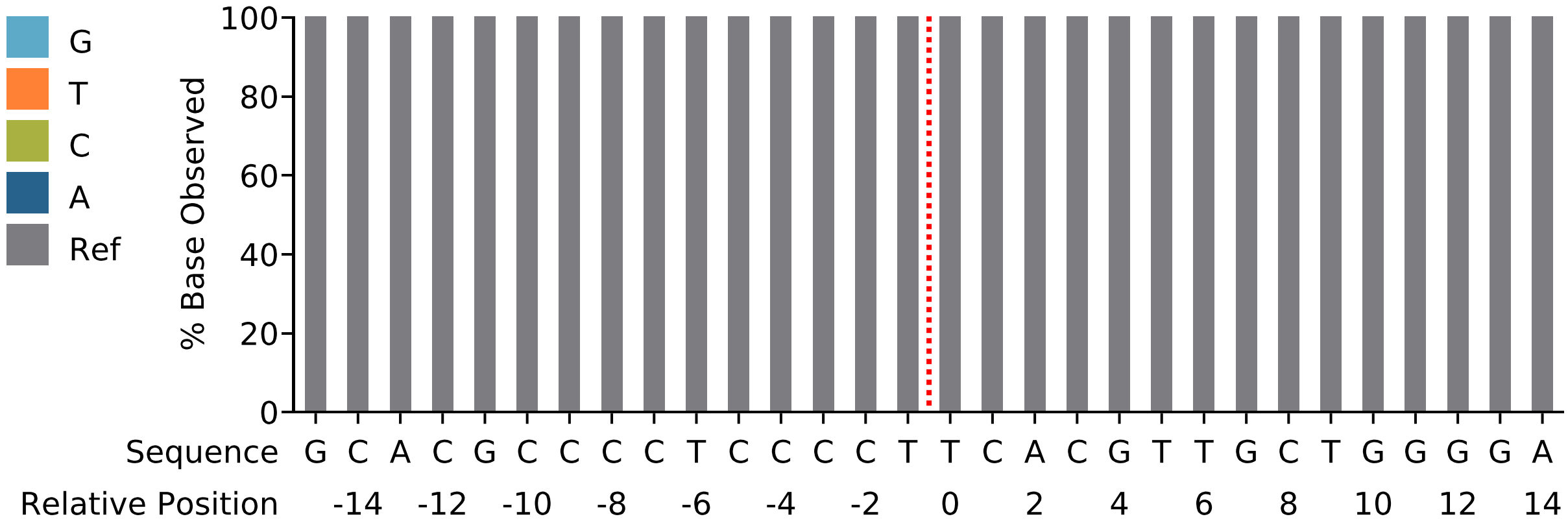

HBV_Cas9_gRNA9__none17

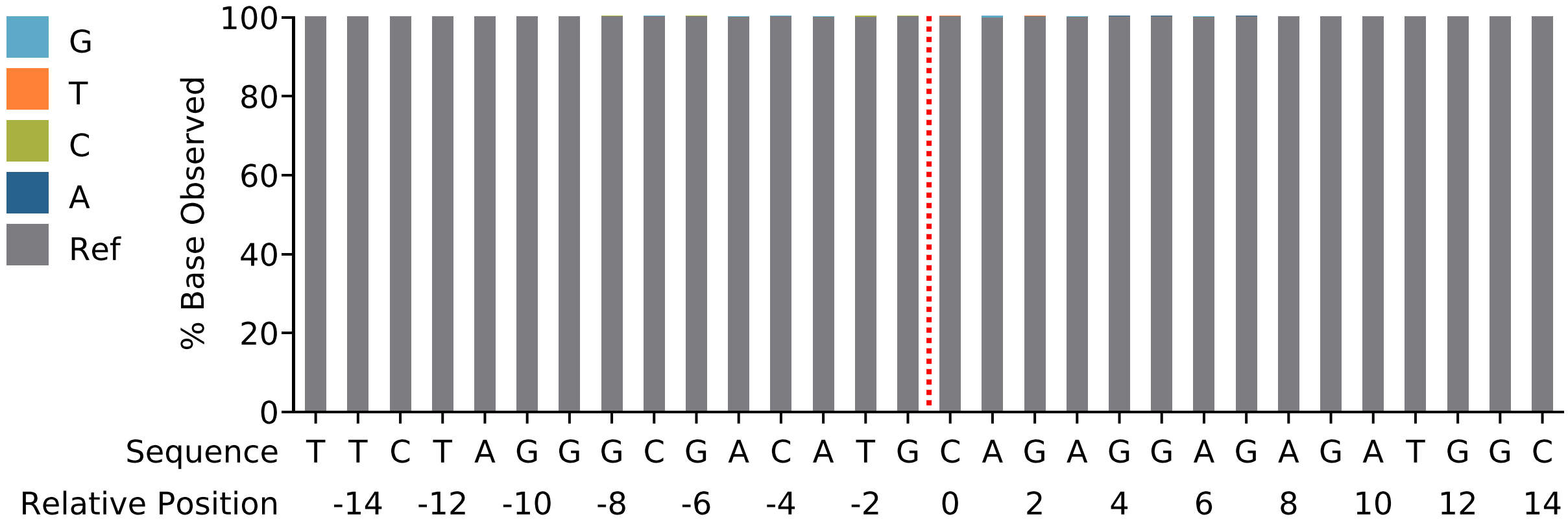

HBV_Cas9_gRNA9__none18

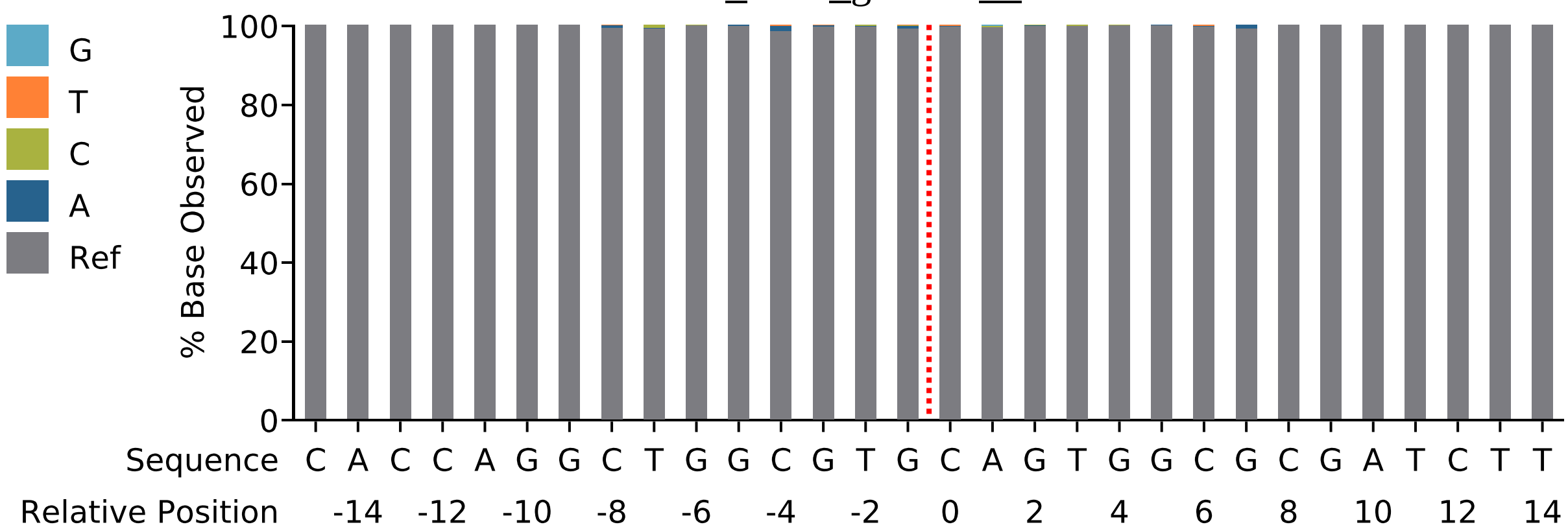

HBV_Cas9_gRNA9__none19

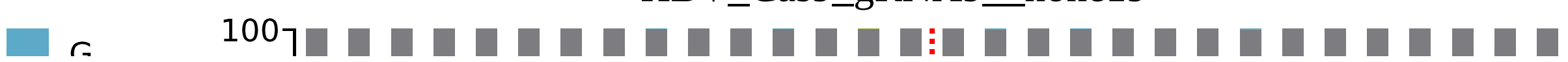

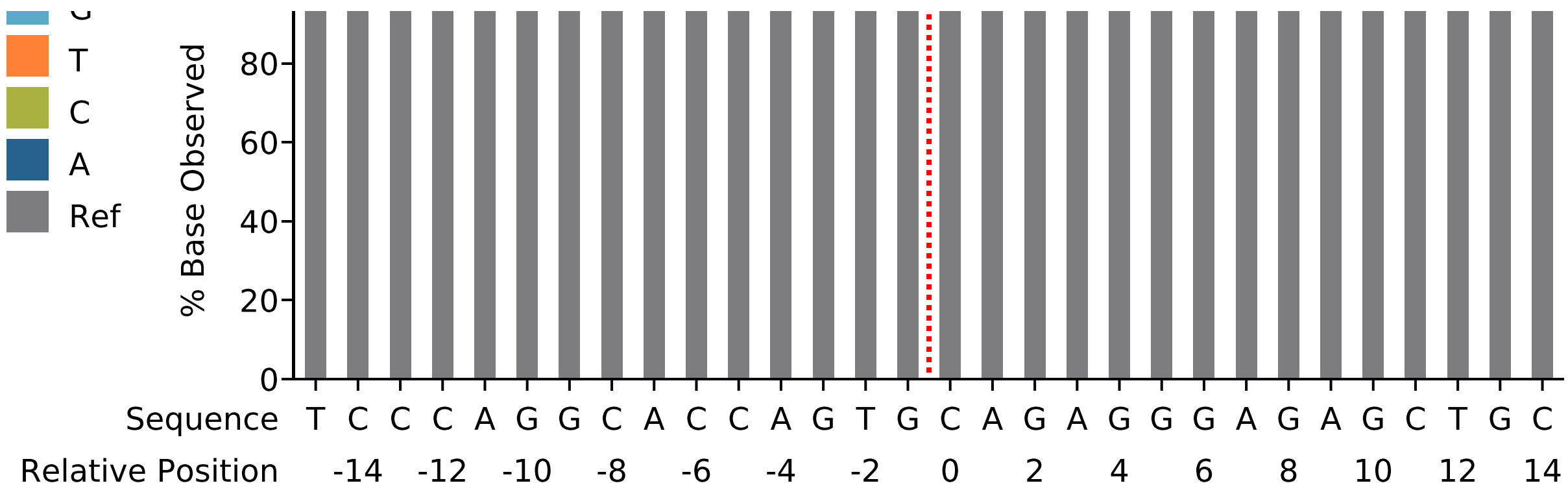

HBV_Cas9_gRNA9__none20

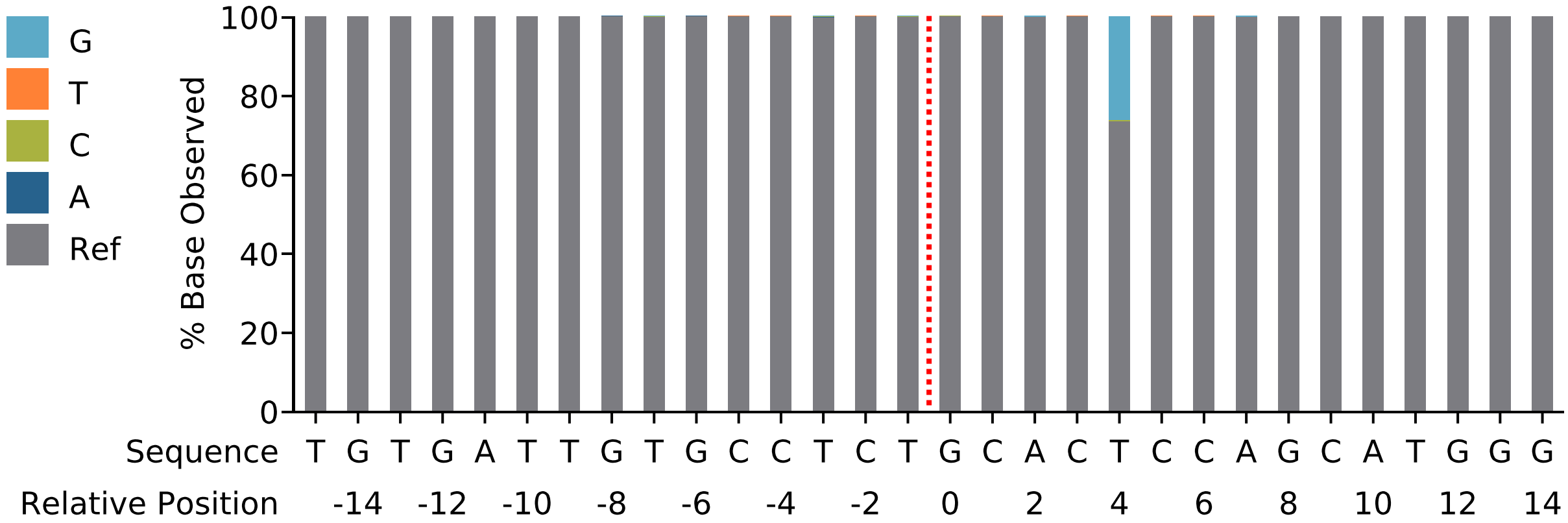

HBV_Cas9_gRNA9__none21

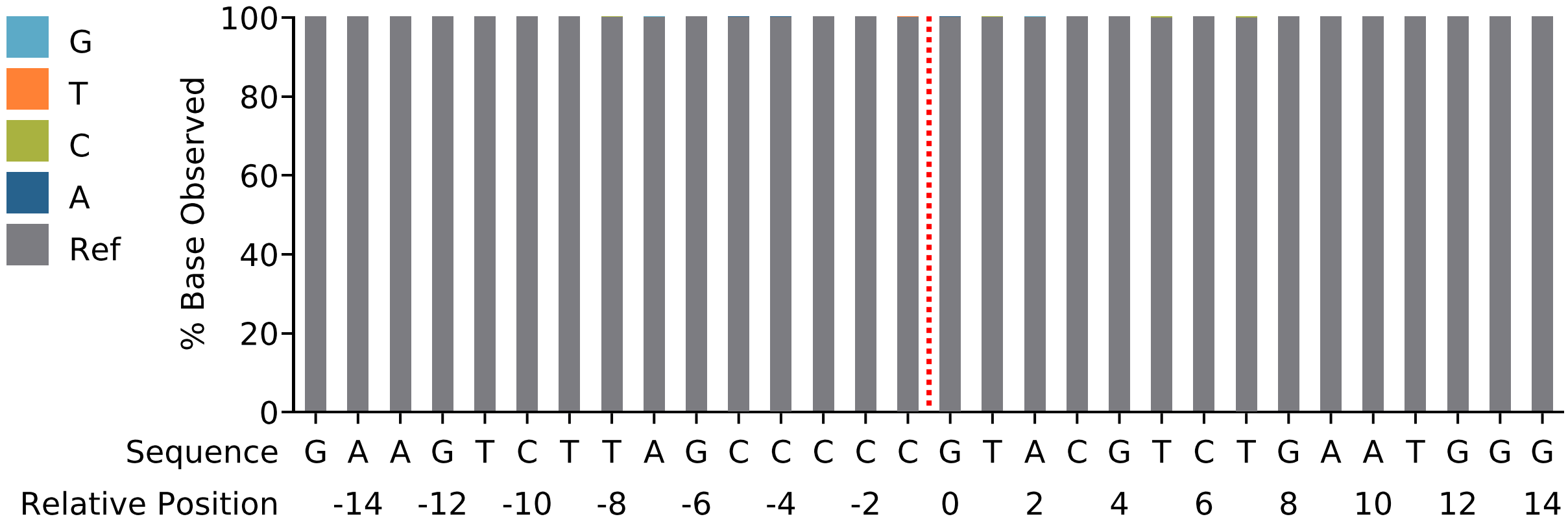

HBV_Cas9_gRNA9__none22

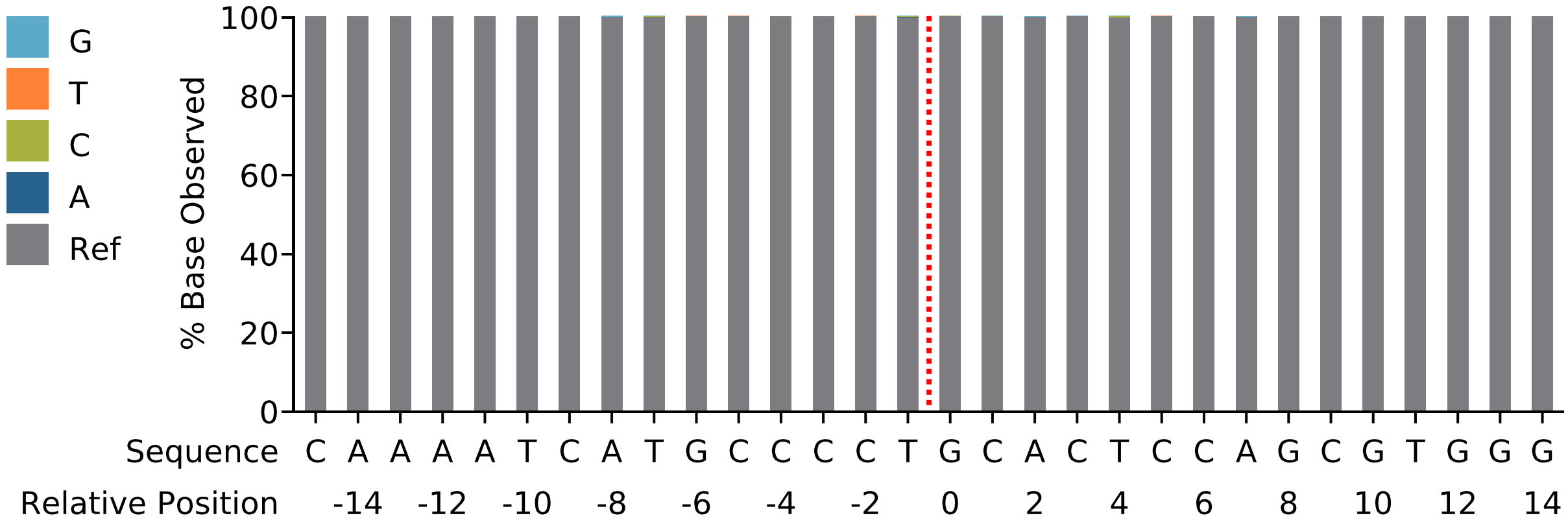

Supplement: Supplementary file 3 [file DataSheet3.pdf]
