# Supplementary material for: Combined RNA- and DNA-targeting CRISPR ribonucleoproteins enhance suppression of HBV transcripts
Source: Front Genome Ed. 2026 Sep 23;8:1824895. doi: 10.3389/fgeed.2026.1824895 (PMC13642003; doi:10.3389/fgeed.2026.1824895)

Analysis mode: CRISPAltRations 1_2_1
Start date: 2026/08/21 15:27
Targets: HBV_Cas12_gRNA4__CD300E, HBV_Cas12_gRNA4__CHKB-DT, HBV_Cas12_gRNA4__GNA12, HBV_Cas12_gRNA4__LINC00989, HBV_Cas12_gRNA4__LOC107986930, HBV_Cas12_gRNA4__MRGBP, HBV_Cas12_gRNA4__PCNX2, HBV_Cas12_gRNA4__SHROOM2, HBV_Cas12_gRNA4__SYNJ2BP, HBV_Cas12_gRNA4__TANK, HBV_Cas12_gRNA4__TCF12, HBV_Cas12_gRNA4__TEKT5, HBV_Cas12_gRNA4__VTI1A, HBV_Cas12_gRNA4__ZBTB44-DT, HBV_Cas12_gRNA4__ZNF292, HBV_Cas12_gRNA4__none52, HBV_Cas12_gRNA4__none54, HBV_Cas12_gRNA4__none55, HBV_Cas12_gRNA4__none56, HBV_Cas12a_Control__CNTN451, HBV_Cas12a_Control__POGLUT3, HBV_Cas12a_Control__ROR1, HBV_Cas12a_Control__SHISA6, HBV_Cas12a_Control__TMC4, HBV_Cas12a_Control__none44, HBV_Cas12a_Control__none46, HBV_Cas12a_Control__none47, HBV_Cas12a_Control__none48, HBV_Cas12a_Control__none49

Metrics

	Group ID	Treated	Total Reads	Percent Edited	Percent Unedited	Percent NHEJ	Percent Perfect HDR	Percent Imperfect HDR	Percent HDR	Percent Other	Percent In Frame	Percent Frameshift	Percent Indels	Percent Insertions	Percent Templated Insertion	Percent GC-Insertion	Percent Deletions
**HBV_Cas12_gRNA4__CD300E**	HBV_Fig4	FALSE	312	98.4	1.6	0.0	0.0	0.0	0.0	98.4	100.0	0.0	0.0	0.0	0.0	0.0	0.0
**HBV_Cas12_gRNA4__CHKB-DT**	HBV_Fig4	FALSE	4707	3.25	96.75	0.08	0.0	0.0	0.0	3.17	99.92	0.08	0.08	0.0	0.0	0.0	0.08
**HBV_Cas12_gRNA4__GNA12**	HBV_Fig4	FALSE	25294	3.01	96.99	0.11	0.0	0.0	0.0	2.9	99.89	0.11	0.11	0.01	100.0	0.0	0.11
**HBV_Cas12_gRNA4__LINC00989**	HBV_Fig4	FALSE	51895	3.18	96.82	0.08	0.0	0.0	0.0	3.1	99.92	0.08	0.08	0.0	0.0	0.0	0.08
**HBV_Cas12_gRNA4__LOC107986930**	HBV_Fig4	FALSE	26613	4.09	95.91	0.13	0.0	0.0	0.0	3.96	99.87	0.13	0.13	0.0	0.0	0.0	0.13
**HBV_Cas12_gRNA4__MRGBP**	HBV_Fig4	FALSE	11635	43.72	56.28	0.16	0.0	0.0	0.0	43.56	99.93	0.07	0.16	0.0	0.0	0.0	0.16
**HBV_Cas12_gRNA4__PCNX2**	HBV_Fig4	FALSE	135668	3.32	96.68	0.07	0.0	0.0	0.0	3.25	99.94	0.06	0.07	0.0	0.0	0.0	0.07
**HBV_Cas12_gRNA4__SHROOM2**	HBV_Fig4	FALSE	28044	3.11	96.89	0.37	0.0	0.0	0.0	2.74	99.63	0.37	0.37	0.06	100.0	0.0	0.3
**HBV_Cas12_gRNA4__SYNJ2BP**	HBV_Fig4	FALSE	38513	3.54	96.46	0.09	0.0	0.0	0.0	3.45	99.91	0.09	0.09	0.0	0.0	0.0	0.09
**HBV_Cas12_gRNA4__TANK**	HBV_Fig4	FALSE	62210	3.34	96.66	0.16	0.0	0.0	0.0	3.18	99.84	0.16	0.16	0.0	0.0	0.0	0.16
**HBV_Cas12_gRNA4__TCF12**	HBV_Fig4	FALSE	15145	99.97	0.03	99.85	0.0	0.0	0.0	0.12	99.97	0.03	99.85	99.85	0.0	0.27	0.01
**HBV_Cas12_gRNA4__TEKT5**	HBV_Fig4	FALSE	1742448	4.28	95.72	0.08	0.0	0.0	0.0	4.2	99.93	0.07	0.08	0.0	0.0	0.0	0.08
**HBV_Cas12_gRNA4__VTI1A**	HBV_Fig4	FALSE	20797	4.02	95.98	0.02	0.0	0.0	0.0	4.0	99.98	0.02	0.02	0.0	0.0	0.0	0.02
**HBV_Cas12_gRNA4__ZBTB44-DT**	HBV_Fig4	FALSE	2747	3.13	96.87	0.07	0.0	0.0	0.0	3.06	99.93	0.07	0.07	0.0	0.0	0.0	0.07
**HBV_Cas12_gRNA4__ZNF292**	HBV_Fig4	FALSE	34090	4.4	95.6	0.09	0.0	0.0	0.0	4.31	99.91	0.09	0.09	0.0	0.0	0.0	0.09
**HBV_Cas12_gRNA4__none52**	HBV_Fig4	FALSE	56104	3.55	96.45	0.01	0.0	0.0	0.0	3.54	99.99	0.01	0.01	0.0	0.0	0.0	0.01
**HBV_Cas12_gRNA4__none54**	HBV_Fig4	FALSE	3980	3.47	96.53	0.08	0.0	0.0	0.0	3.39	99.92	0.08	0.08	0.0	0.0	0.0	0.08
**HBV_Cas12_gRNA4__none55**	HBV_Fig4	FALSE	34556	99.9	0.1	0.15	0.0	0.0	0.0	99.75	99.86	0.14	0.15	0.0	100.0	0.0	0.14
**HBV_Cas12_gRNA4__none56**	HBV_Fig4	FALSE	48390	3.52	96.48	0.1	0.0	0.0	0.0	3.42	99.9	0.1	0.1	0.0	0.0	0.0	0.1
**HBV_Cas12a_Control__CNTN451**	HBV_Fig4	FALSE	40223	3.89	96.11	0.04	0.0	0.0	0.0	3.85	99.96	0.04	0.04	0.0	0.0	0.0	0.04
**HBV_Cas12a_Control__POGLUT3**	HBV_Fig4	FALSE	117861	37.12	62.88	0.28	0.0	0.0	0.0	36.84	99.72	0.28	0.28	0.01	100.0	0.0	0.27
**HBV_Cas12a_Control__ROR1**	HBV_Fig4	FALSE	6360	8.9	91.1	3.18	0.0	0.0	0.0	5.72	97.41	2.59	3.18	0.82	0.0	0.0	2.36
**HBV_Cas12a_Control__SHISA6**	HBV_Fig4	FALSE	52461	3.05	96.95	0.2	0.0	0.0	0.0	2.85	99.8	0.2	0.2	0.01	25.0	50.0	0.19
**HBV_Cas12a_Control__TMC4**	HBV_Fig4	FALSE	6446	100.0	0.0	100.0	0.0	0.0	0.0	0.0	97.27	2.73	100.0	100.0	0.0	0.0	0.05
**HBV_Cas12a_Control__none44**	HBV_Fig4	FALSE	327	93.88	6.12	85.02	0.0	0.0	0.0	8.86	38.84	61.16	85.02	22.02	0.0	22.22	63.0
**HBV_Cas12a_Control__none46**	HBV_Fig4	FALSE	1366608	54.89	45.11	0.08	0.0	0.0	0.0	54.81	99.92	0.08	0.08	0.01	94.29	0.71	0.07
**HBV_Cas12a_Control__none47**	HBV_Fig4	FALSE	18536	10.63	89.37	0.23	0.0	0.0	0.0	10.4	99.94	0.06	0.23	0.0	0.0	0.0	0.23
**HBV_Cas12a_Control__none48**	HBV_Fig4	FALSE	563602	4.14	95.86	0.1	0.0	0.0	0.0	4.04	99.91	0.09	0.1	0.0	0.0	0.0	0.09
**HBV_Cas12a_Control__none49**	HBV_Fig4	FALSE	241594	100.0	0.0	97.66	0.0	0.0	0.0	2.34	19.45	80.55	97.66	46.66	0.0	21.63	51.0

Percentages

% Edited *% Repair Pathways* *% Frameshift*

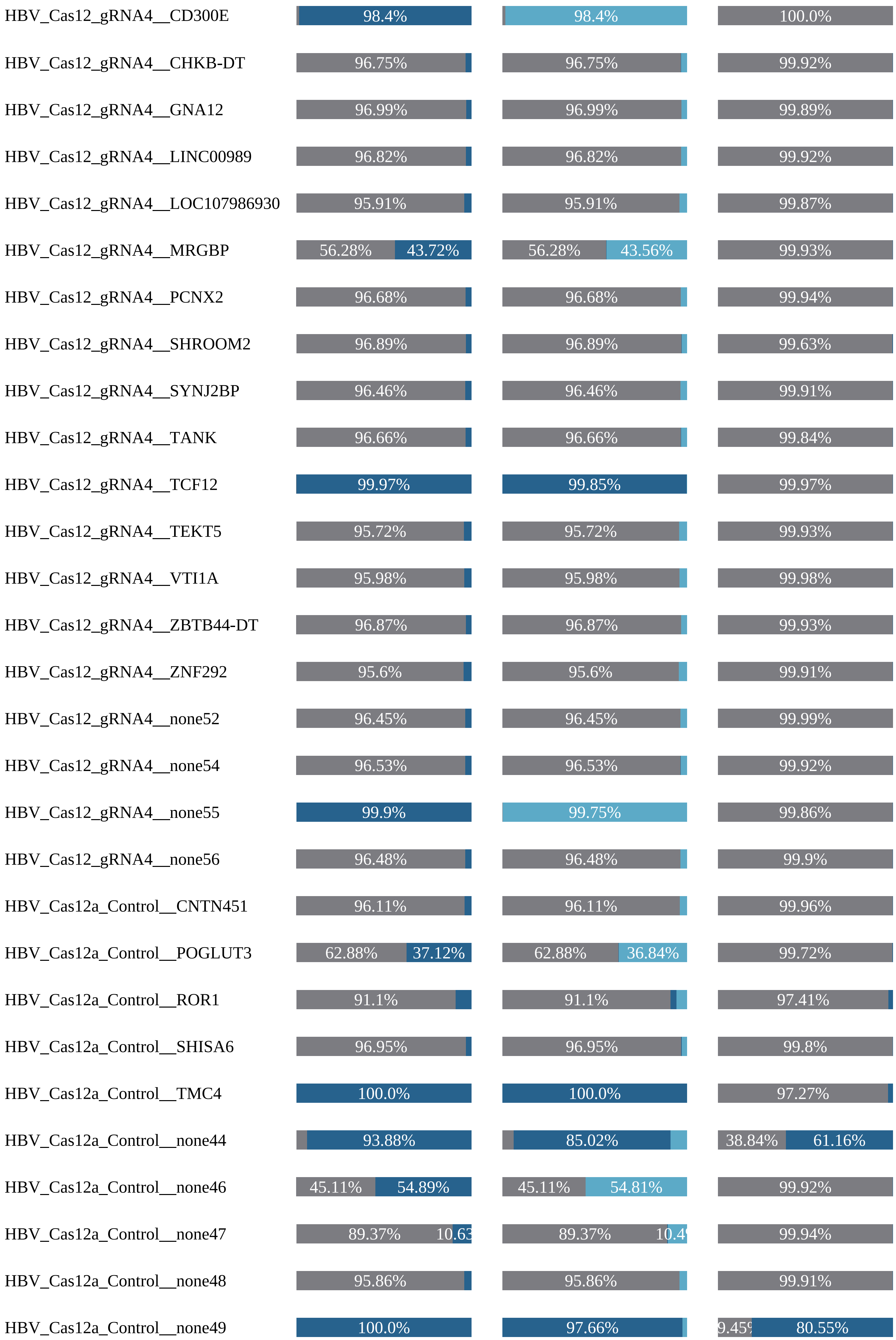
HBV_Cas12_gRNA4__CD300E 98.4% 98.4% 100.0%
HBV_Cas12_gRNA4__CHKB-DT 96.75% 96.75% 99.92%
HBV_Cas12_gRNA4__GNA12 96.99% 96.99% 99.89%
HBV_Cas12_gRNA4__LINC00989 96.82% 96.82% 99.92%
HBV_Cas12_gRNA4__LOC107986930 95.91% 95.91% 99.87%
HBV_Cas12_gRNA4__MRGBP 56.28% 43.72% 56.28% 43.56% 99.93%
HBV_Cas12_gRNA4__PCNX2 96.68% 96.68% 99.94%
HBV_Cas12_gRNA4__SHROOM2 96.89% 96.89% 99.63%
HBV_Cas12_gRNA4__SYNJ2BP 96.46% 96.46% 99.91%
HBV_Cas12_gRNA4__TANK 96.66% 96.66% 99.84%
HBV_Cas12_gRNA4__TCF12 99.97% 99.85% 99.97%
HBV_Cas12_gRNA4__TEKT5 95.72% 95.72% 99.93%
HBV_Cas12_gRNA4__VTI1A 95.98% 95.98% 99.98%
HBV_Cas12_gRNA4__ZBTB44-DT 96.87% 96.87% 99.93%
HBV_Cas12_gRNA4__ZNF292 95.6% 95.6% 99.91%
HBV_Cas12_gRNA4__none52 96.45% 96.45% 99.99%
HBV_Cas12_gRNA4__none54 96.53% 96.53% 99.92%
HBV_Cas12_gRNA4__none55 99.9% 99.75% 99.86%
HBV_Cas12_gRNA4__none56 96.48% 96.48% 99.9%
HBV_Cas12a_Control__CNTN451 96.11% 96.11% 99.96%
HBV_Cas12a_Control__POGLUT3 62.88% 37.12% 62.88% 36.84% 99.72%
HBV_Cas12a_Control__ROR1 91.1% 91.1% 97.41%
HBV_Cas12a_Control__SHISA6 96.95% 96.95% 99.8%
HBV_Cas12a_Control__TMC4 100.0% 100.0% 97.27%
HBV_Cas12a_Control__none44 93.88% 85.02% 38.84% 61.16%
HBV_Cas12a_Control__none46 45.11% 54.89% 45.11% 54.81% 99.92%
HBV_Cas12a_Control__none47 89.37% 10.63 89.37% 10.4 99.94%
HBV_Cas12a_Control__none48 95.86% 95.86% 99.91%
HBV_Cas12a_Control__none49 100.0% 97.66% 9.45 80.55%

Unedited Edited

Unedited NHEJ PerfectHDR ImperfectHDR Other

Inframe Frameshift

Insertions

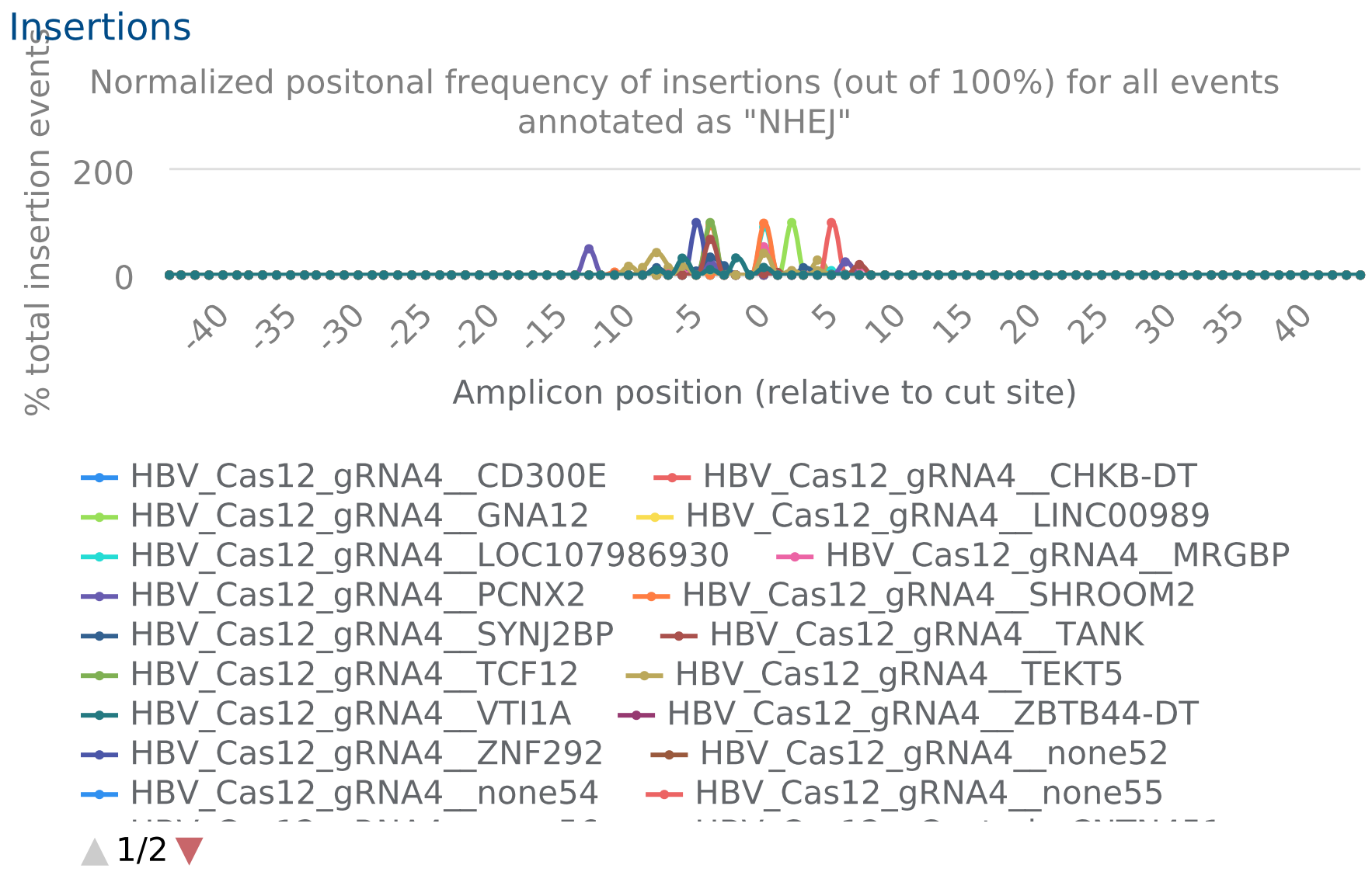

Deletions

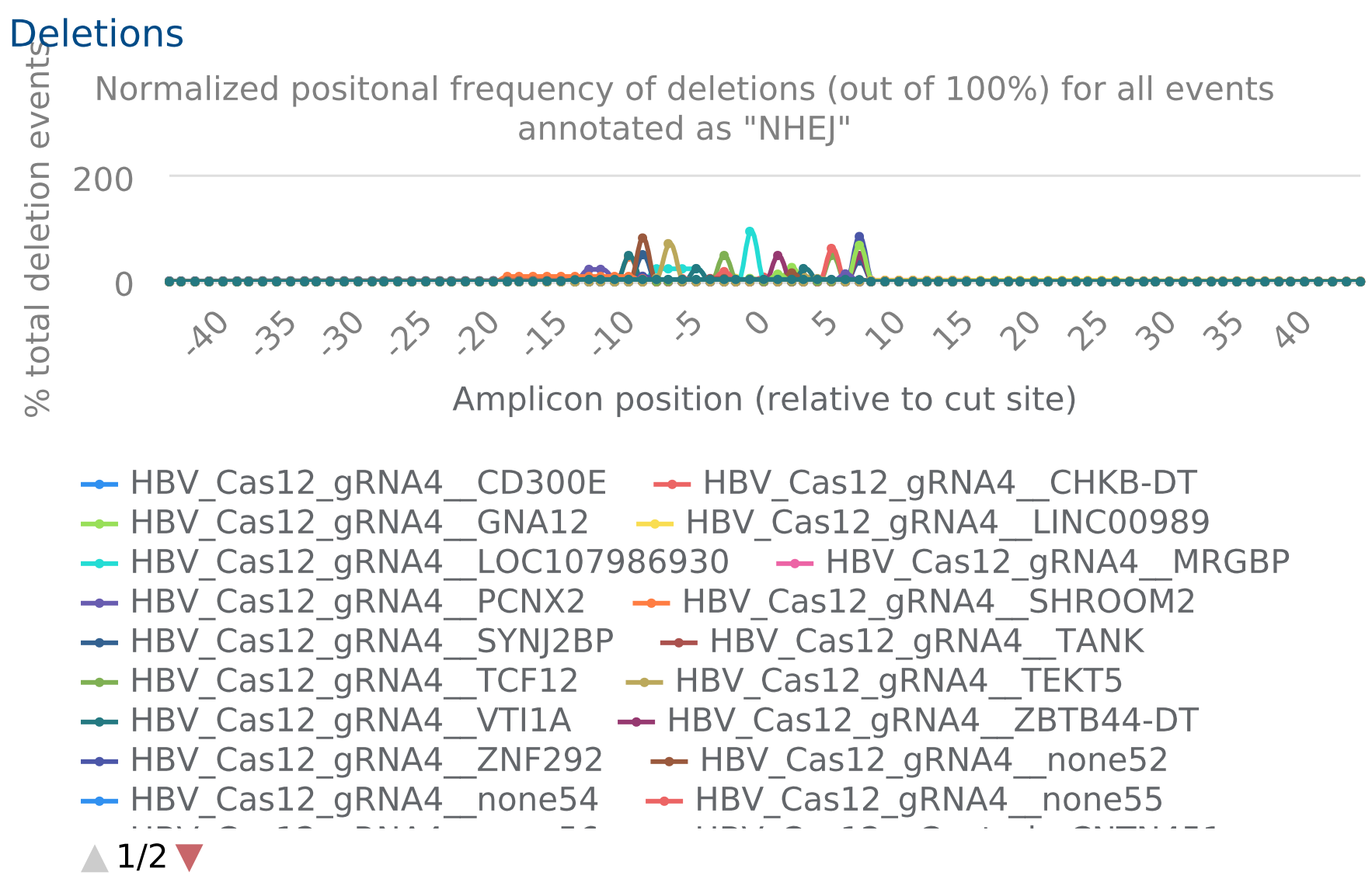

Indel Frequencies

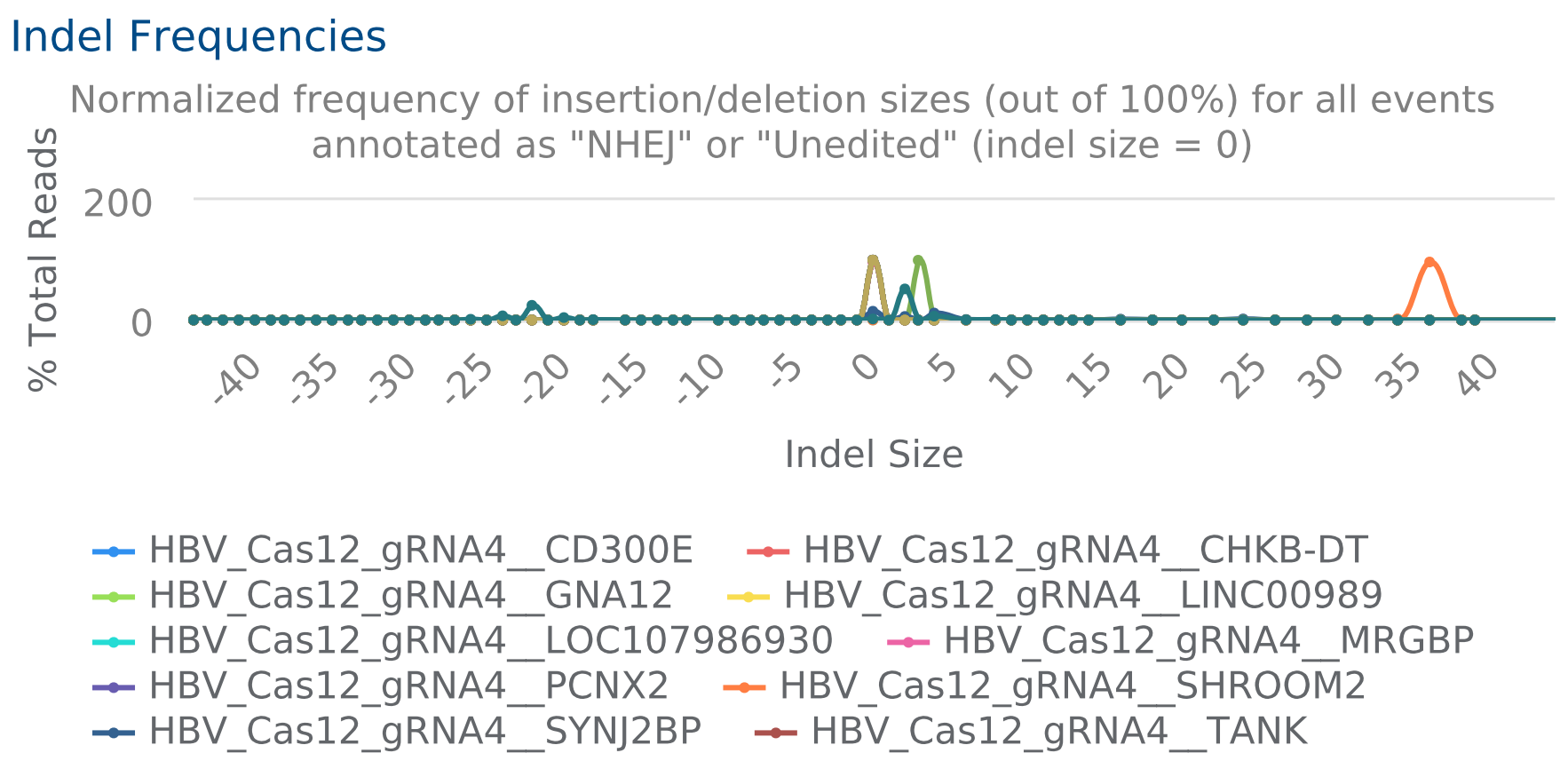

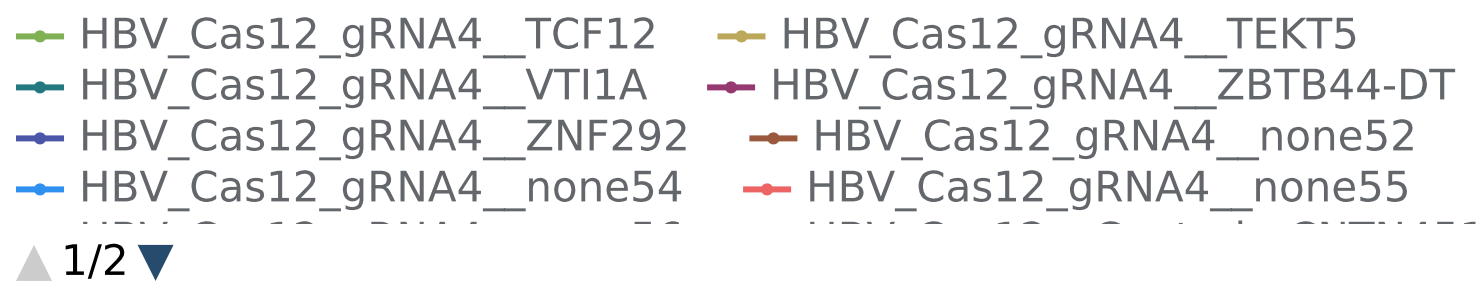

SNP Graph

HBV_Cas12_gRNA4__CD300E

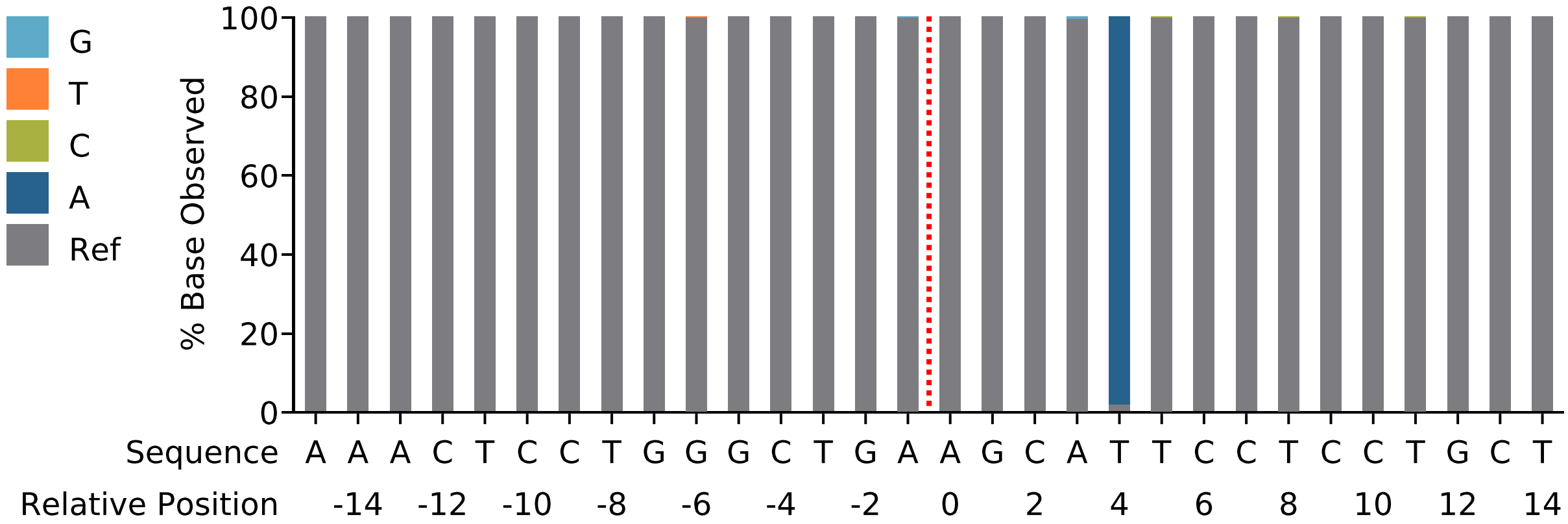

HBV_Cas12_gRNA4__CHKB-DT

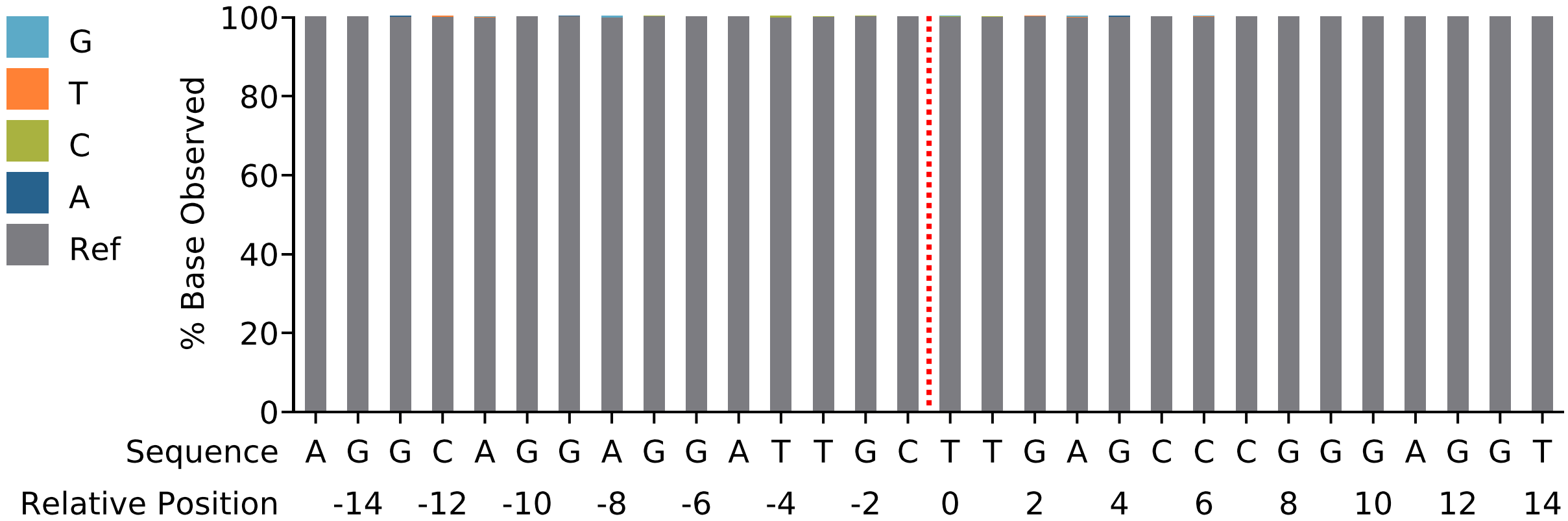

HBV_Cas12_gRNA4__GNA12

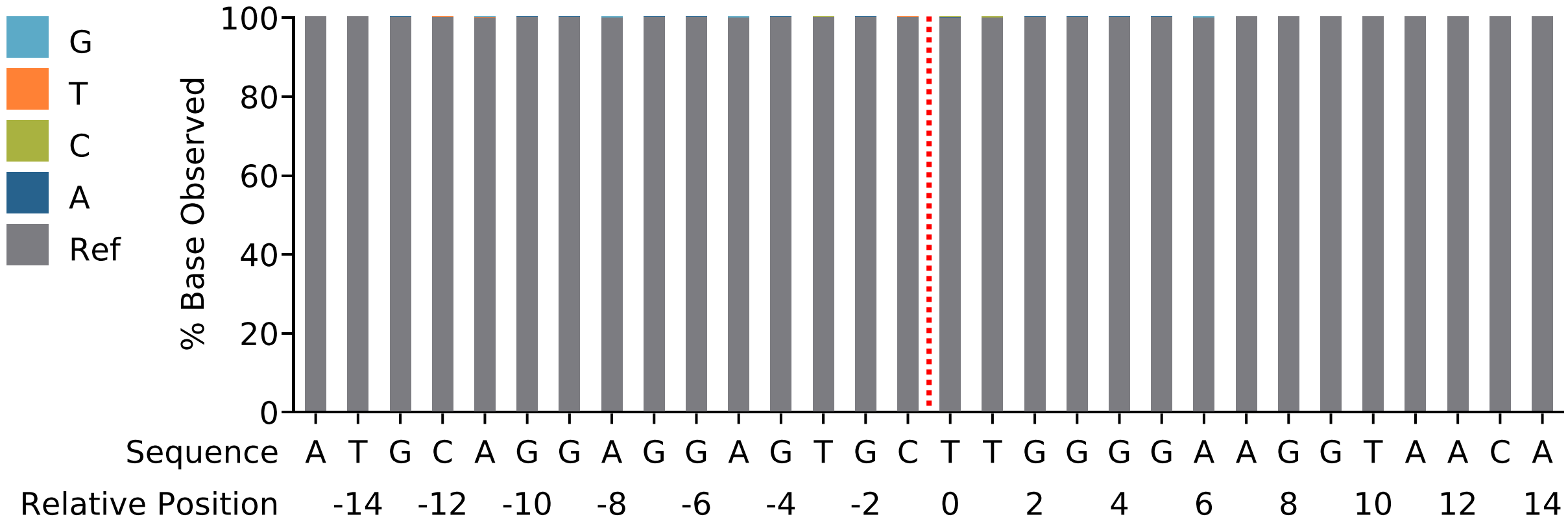

HBV_Cas12_gRNA4__LINC00989

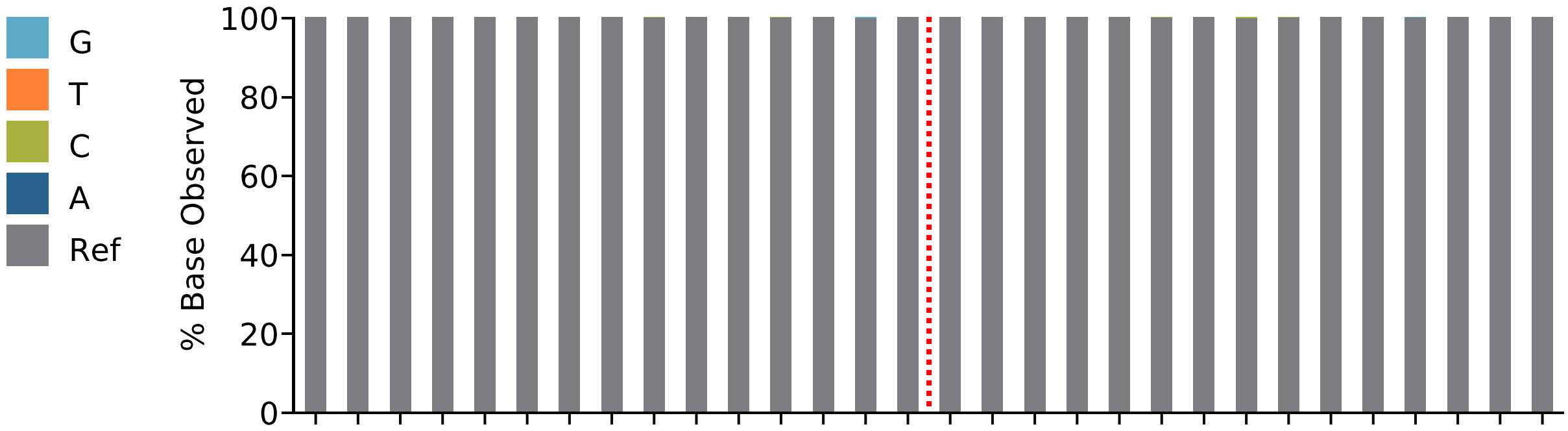

Sequence T C A A G T T C T G G T G A G G G C C C T C T T C C A G G T
Relative Position -14 -12 -10 -8 -6 -4 -2 0 2 4 6 8 10 12 14

HBV_Cas12_gRNA4__LOC107986930

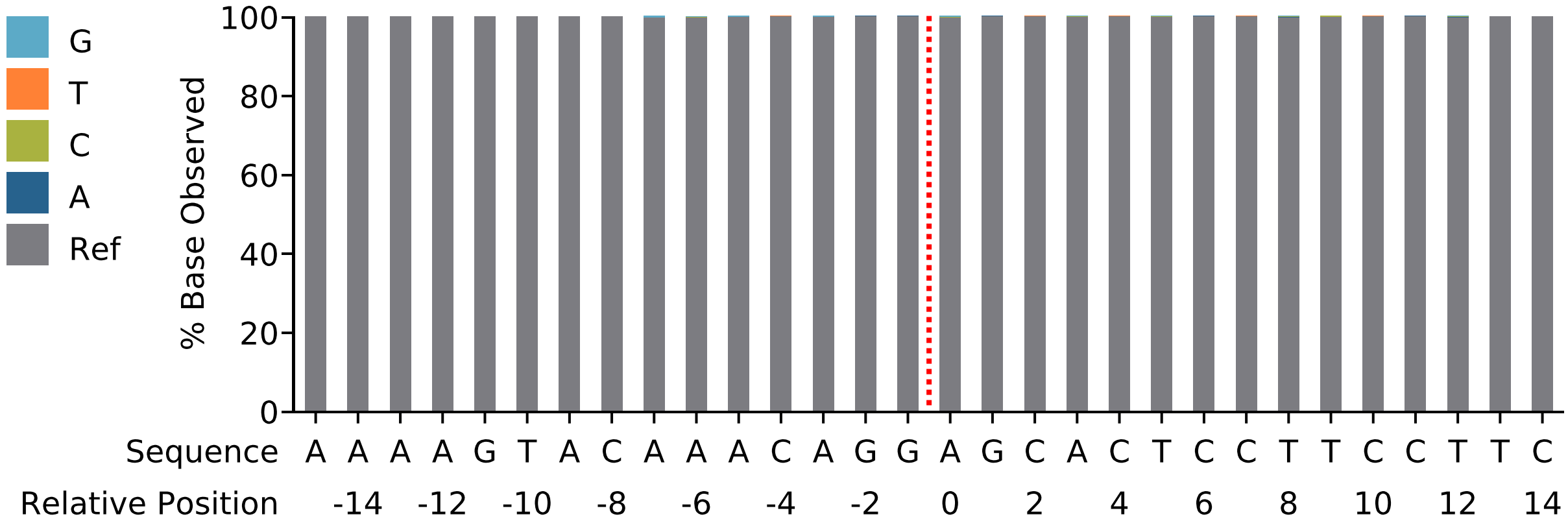

HBV_Cas12_gRNA4__MRGBP

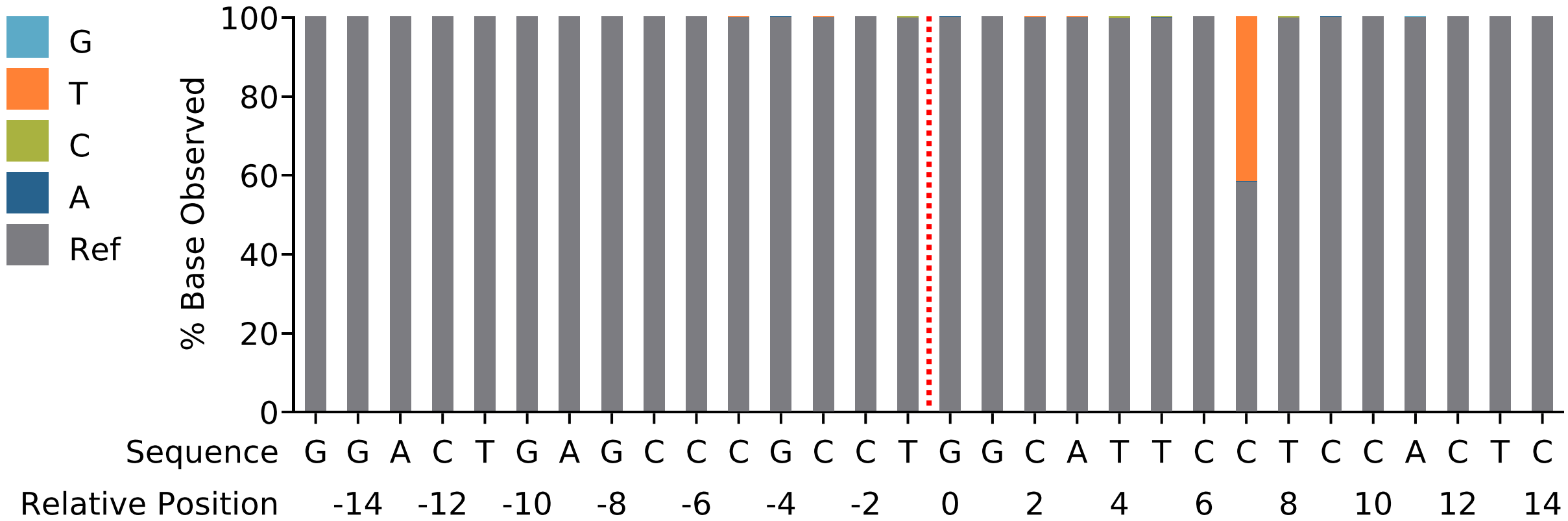

HBV_Cas12_gRNA4__PCNX2

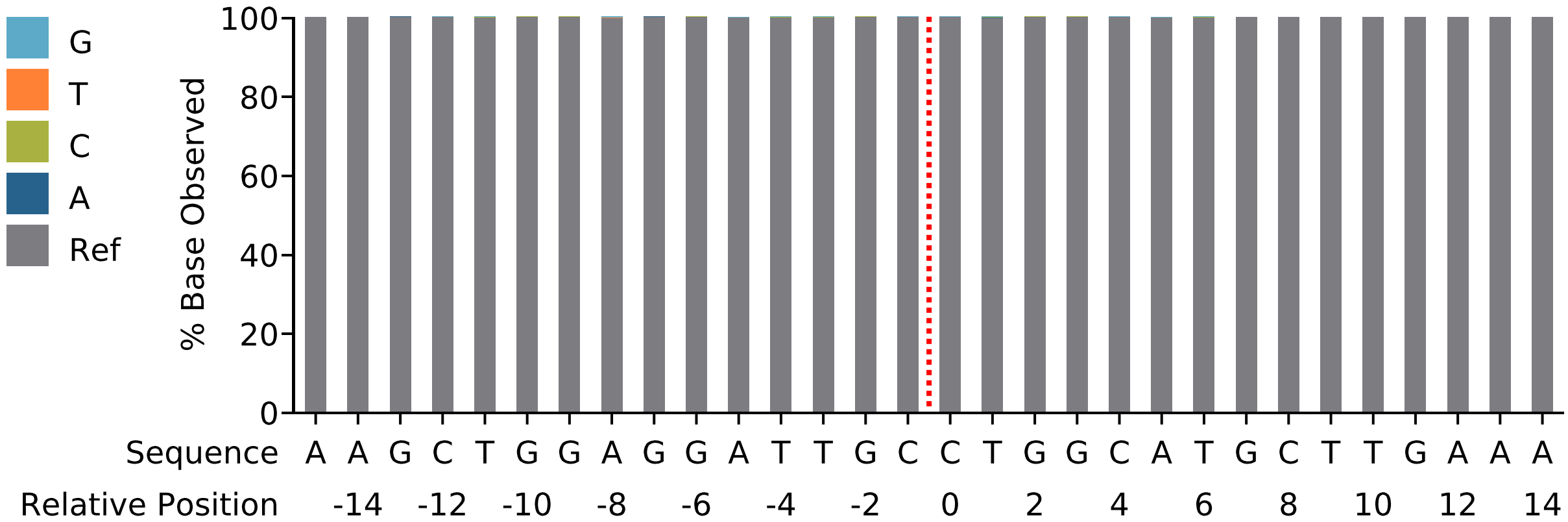

HBV_Cas12_gRNA4__SHROOM2

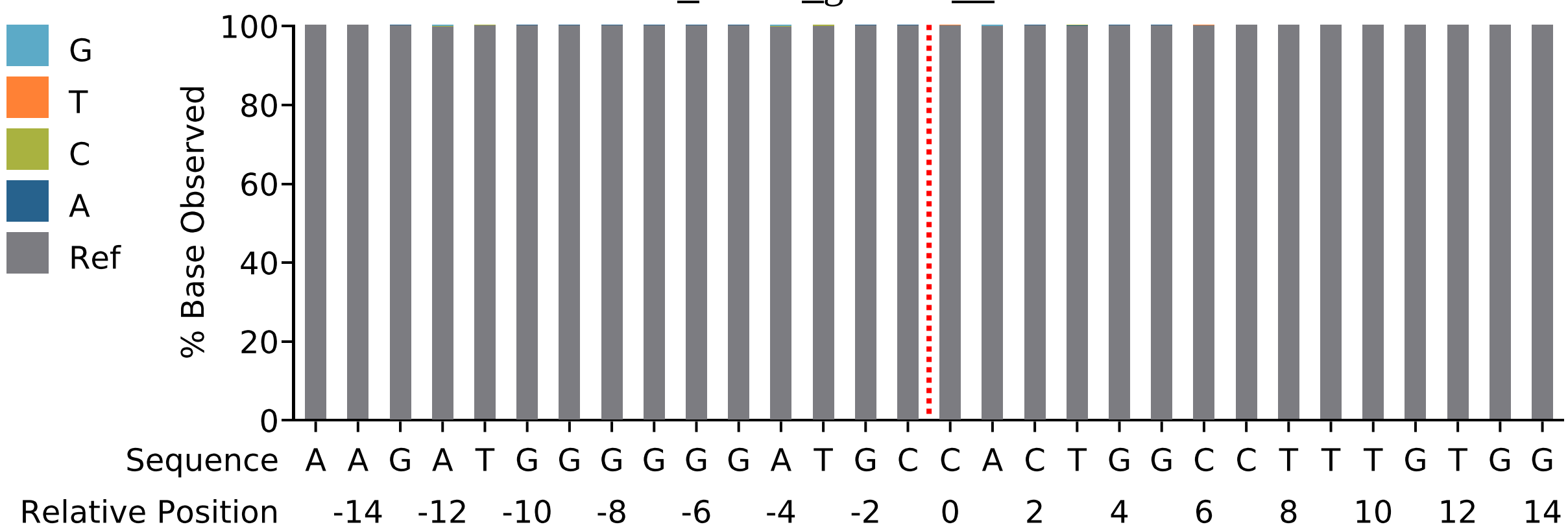

HBV_Cas12_gRNA4__SYNJ2BP

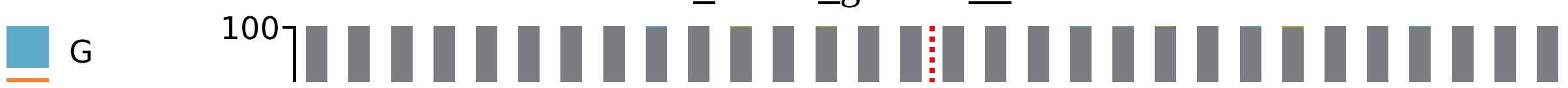

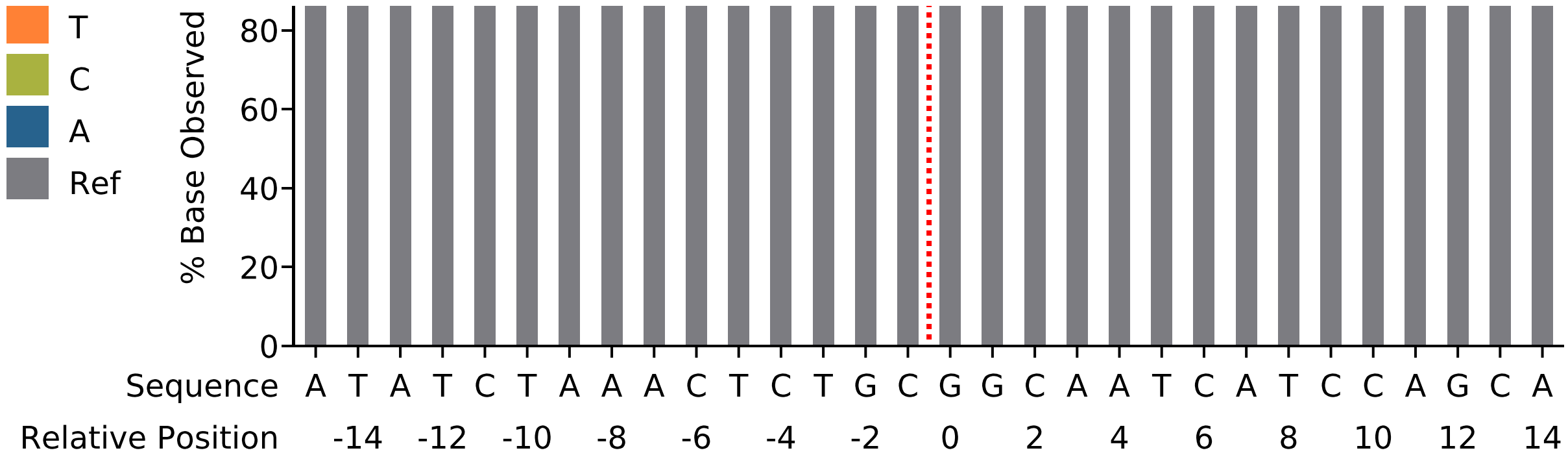

HBV_Cas12_gRNA4__TANK

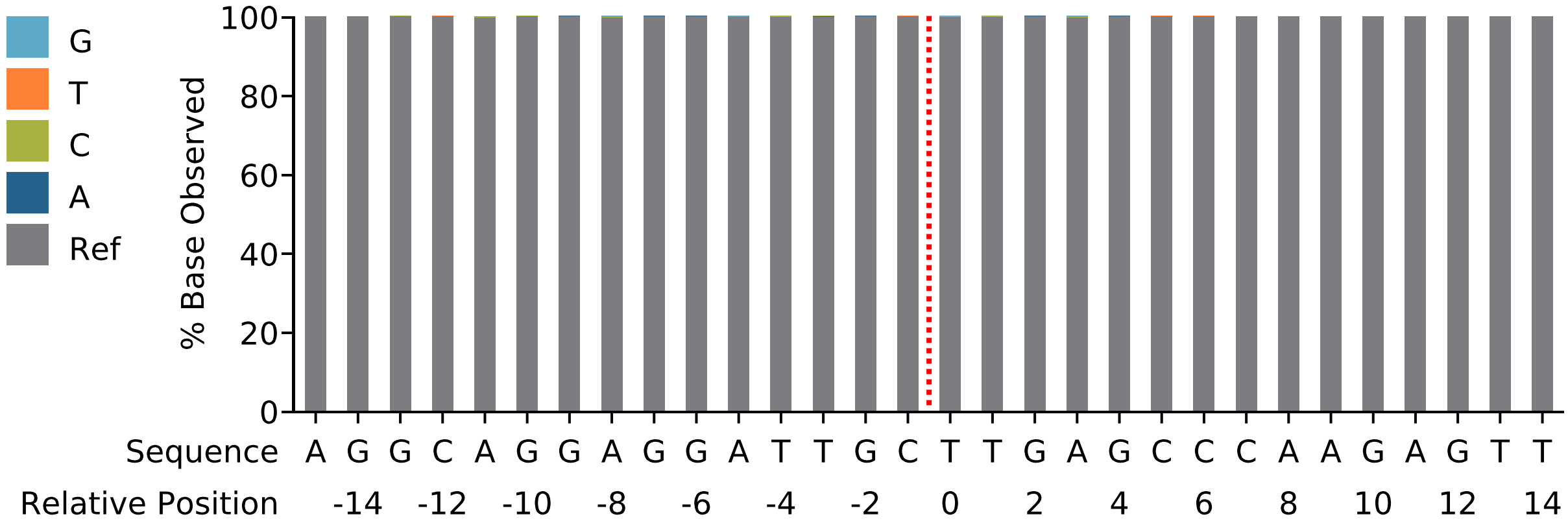

HBV_Cas12_gRNA4__TCF12

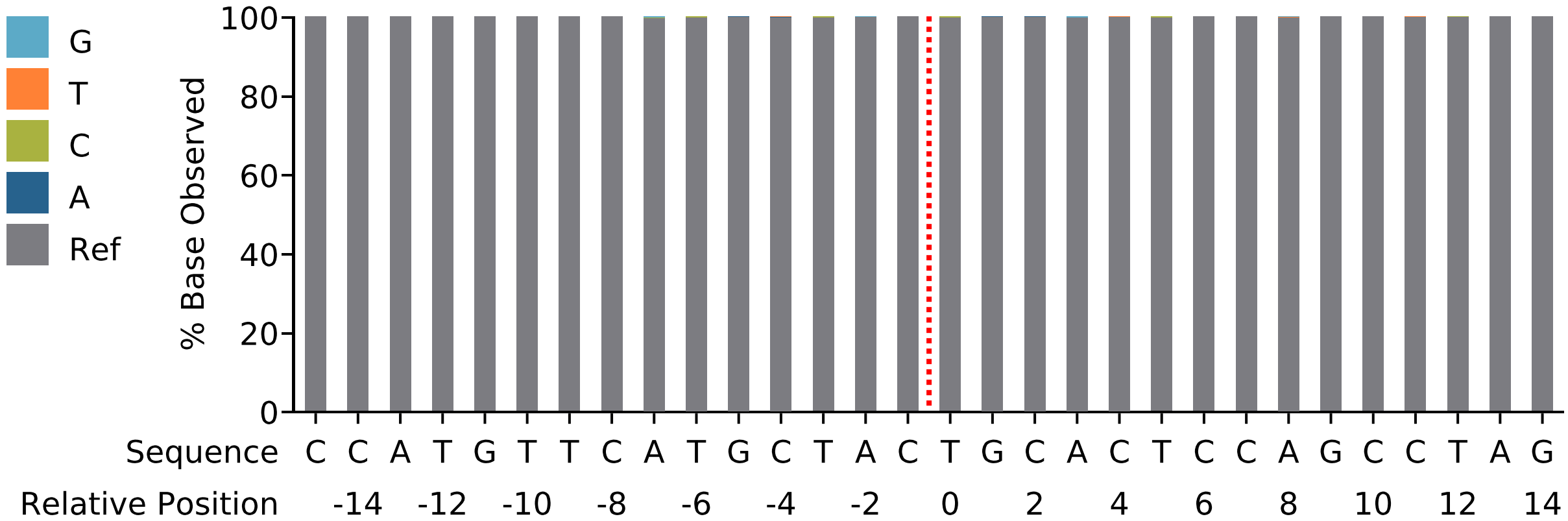

HBV_Cas12_gRNA4__TEKT5

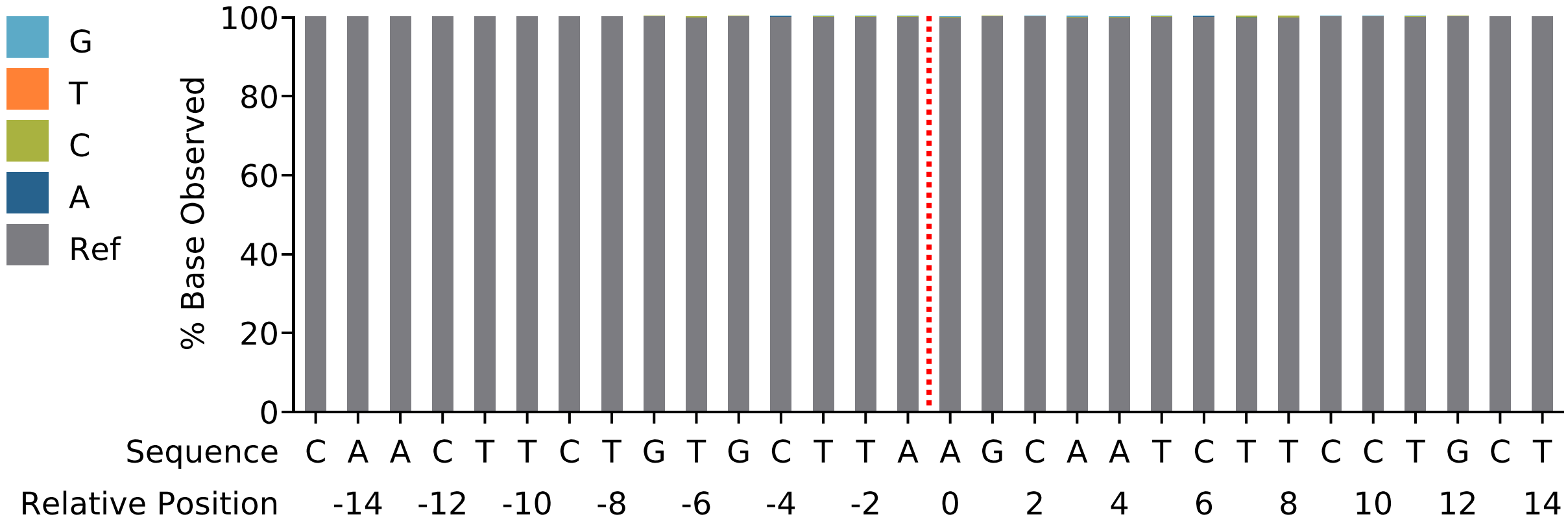

HBV_Cas12_gRNA4__VTI1A

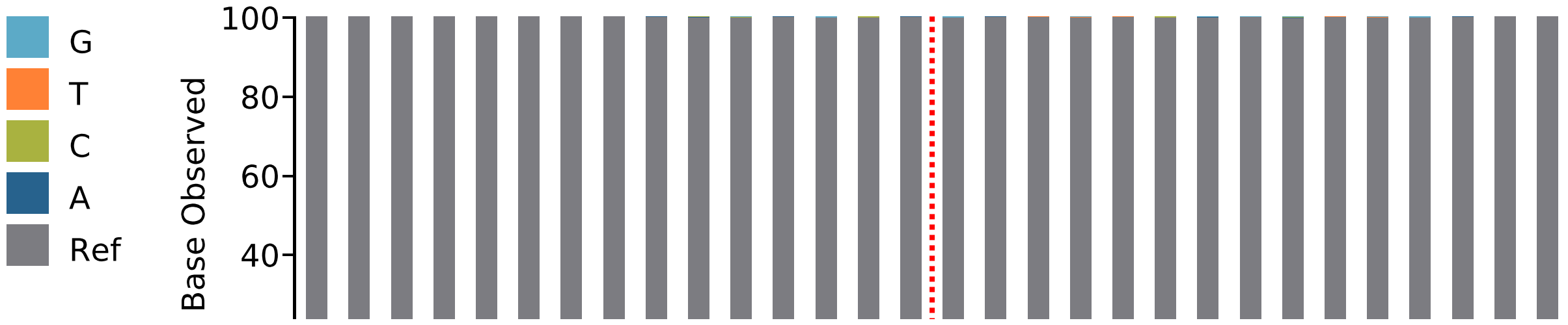

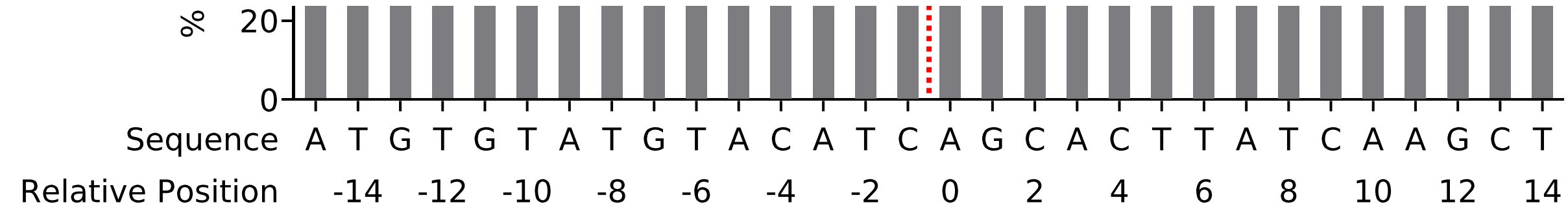

HBV_Cas12_gRNA4__ZBTB44-DT

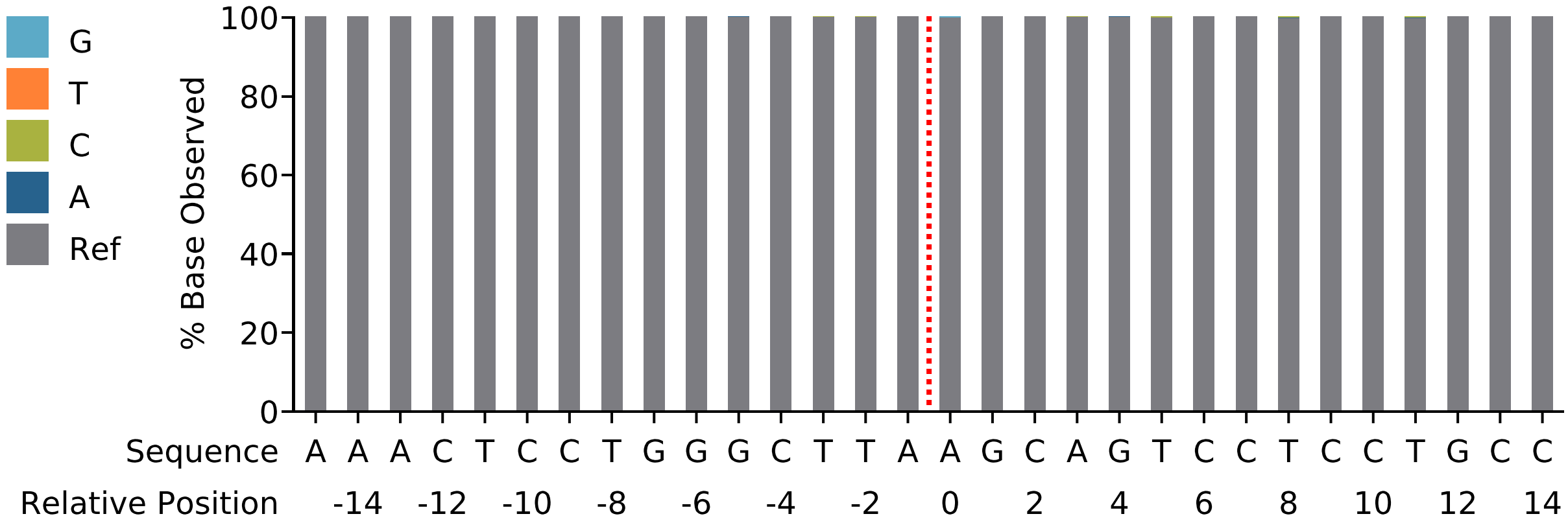

HBV_Cas12_gRNA4__ZNF292

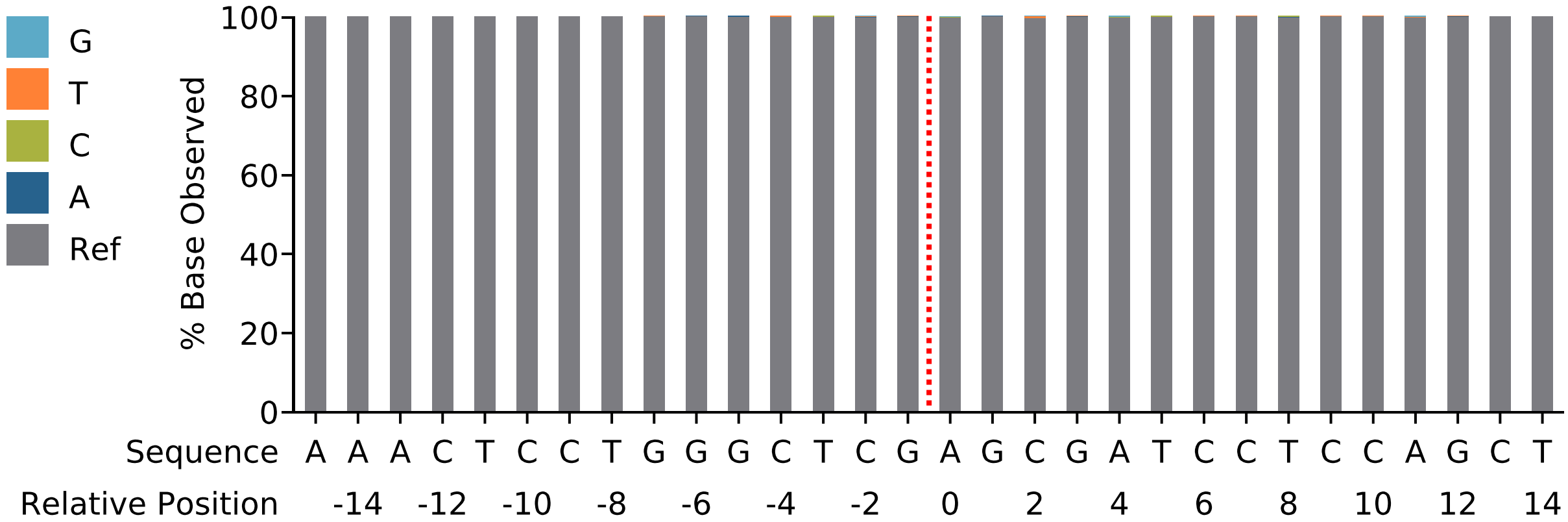

HBV_Cas12_gRNA4__none52

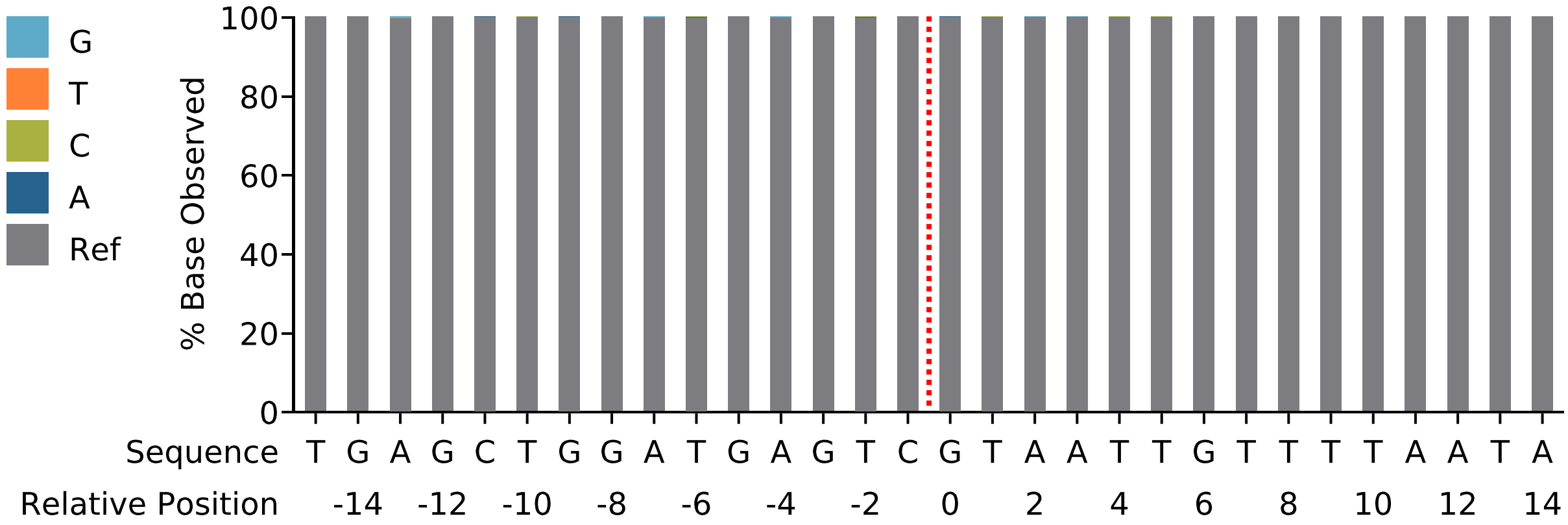

HBV_Cas12_gRNA4__none54

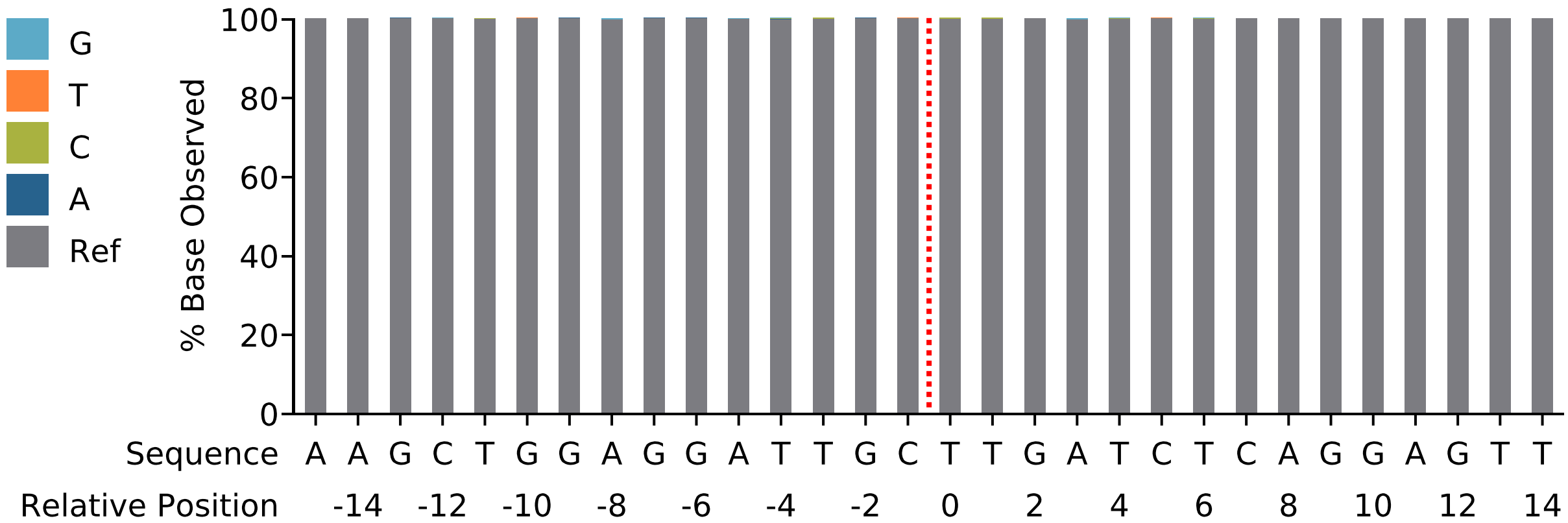

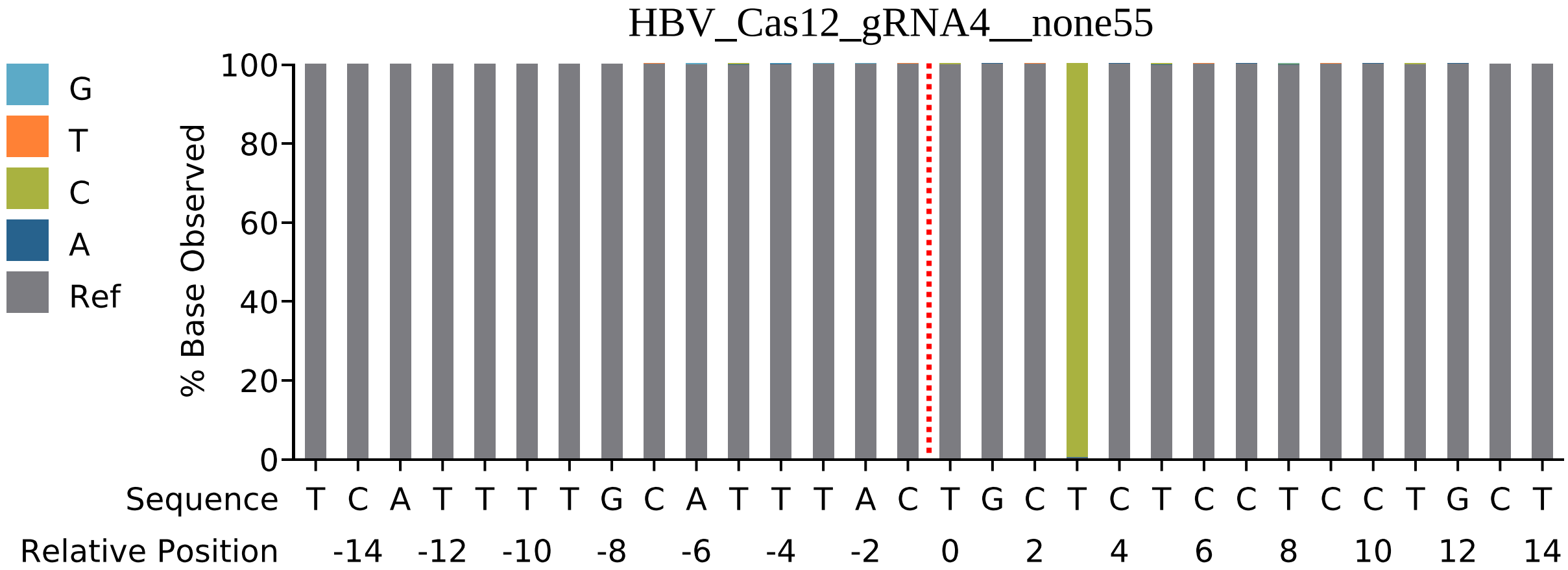
HBV_Cas12_gRNA4__none55
G
T
C
A
Ref
% Base Observed
0
20
40
60
80
100
Sequence T C A T T T T G C A T T T A C T G C T C T C C T C C T G C T
Relative Position -14 -12 -10 -8 -6 -4 -2 0 2 4 6 8 10 12 14

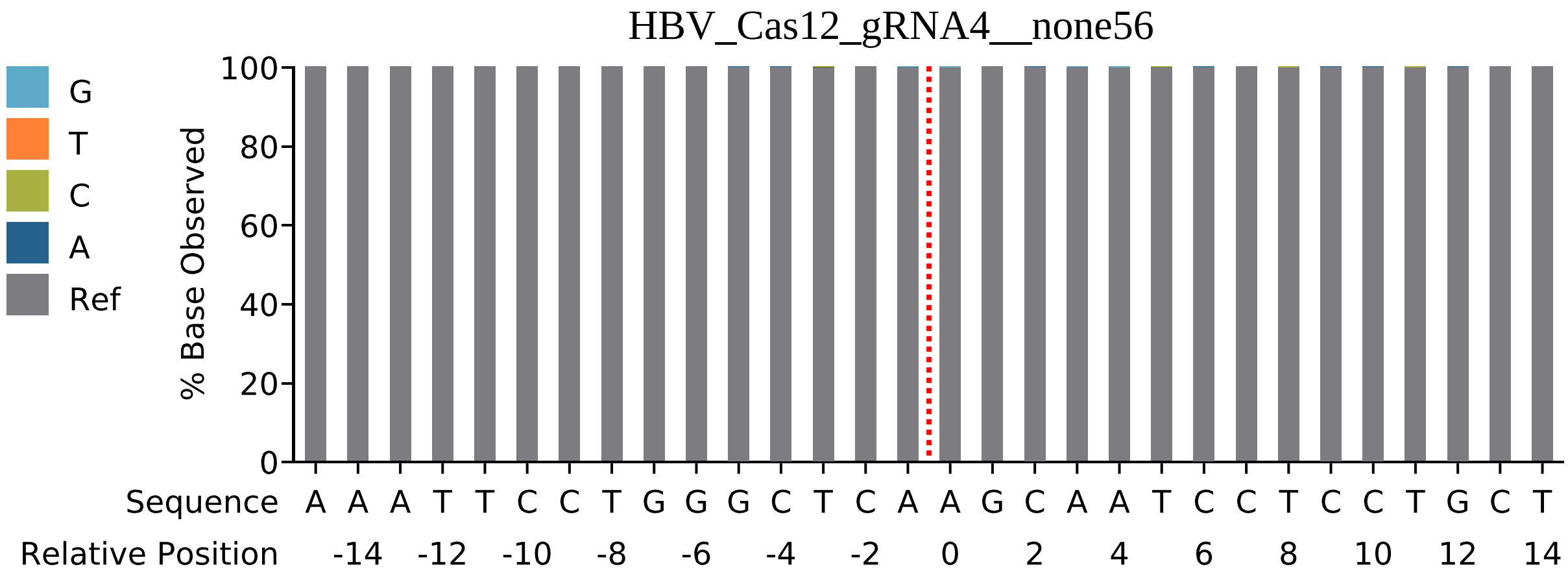
HBV_Cas12_gRNA4__none56
G
T
C
A
Ref
% Base Observed
0
20
40
60
80
100
Sequence A A A T T C C T G G G C T C A A G C A A T C C T C C T G C T
Relative Position -14 -12 -10 -8 -6 -4 -2 0 2 4 6 8 10 12 14

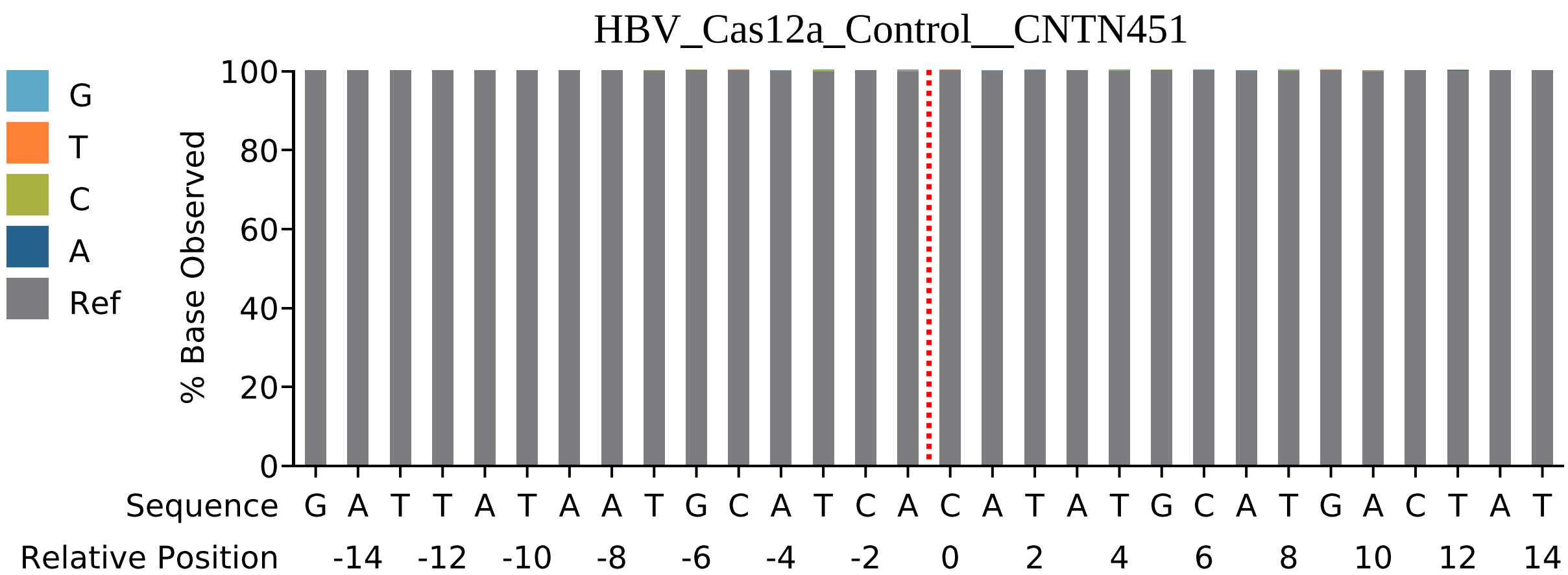
HBV_Cas12a_Control__CNTN451
G
T
C
A
Ref
% Base Observed
0
20
40
60
80
100
Sequence G A T T A T A A T G C A T C A C A T A T G C A T G A C T A T
Relative Position -14 -12 -10 -8 -6 -4 -2 0 2 4 6 8 10 12 14

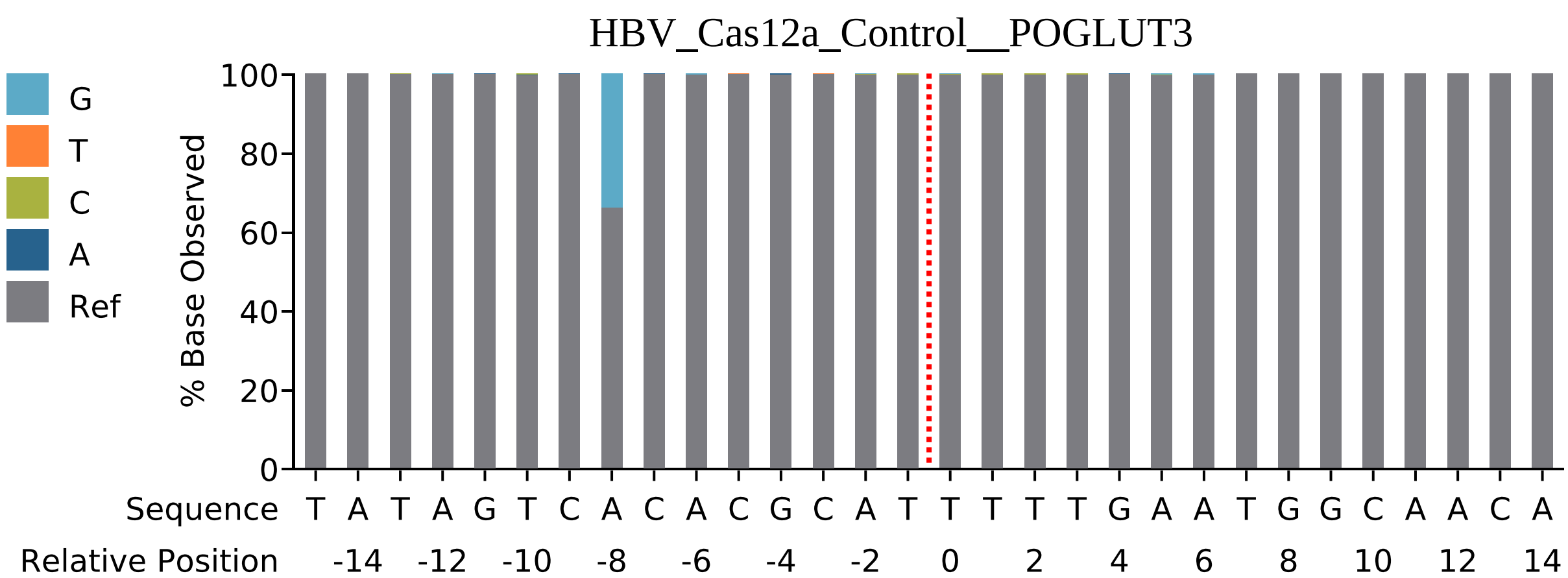
HBV_Cas12a_Control__POGLUT3
G
T
C
A
Ref
% Base Observed
0
20
40
60
80
100
Sequence T A T A G T C A C A C G C A T T T T T G A A T G G C A A C A
Relative Position -14 -12 -10 -8 -6 -4 -2 0 2 4 6 8 10 12 14

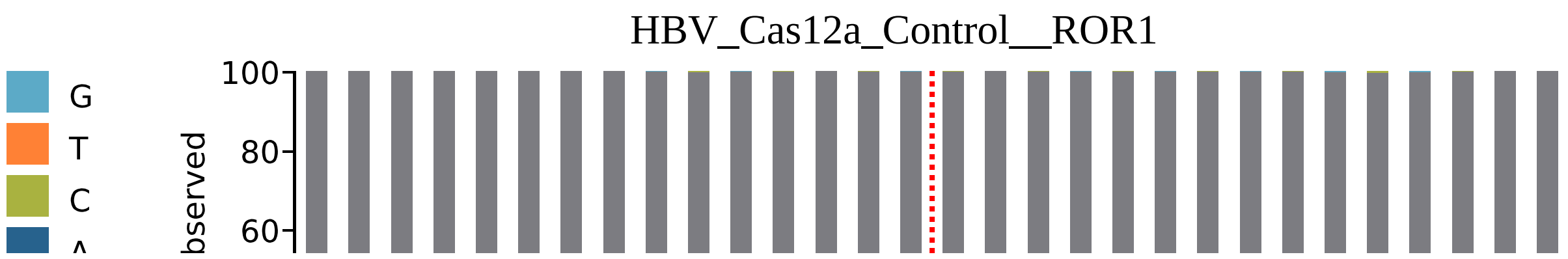
HBV_Cas12a_Control__ROR1
G
T
C
60
80
100

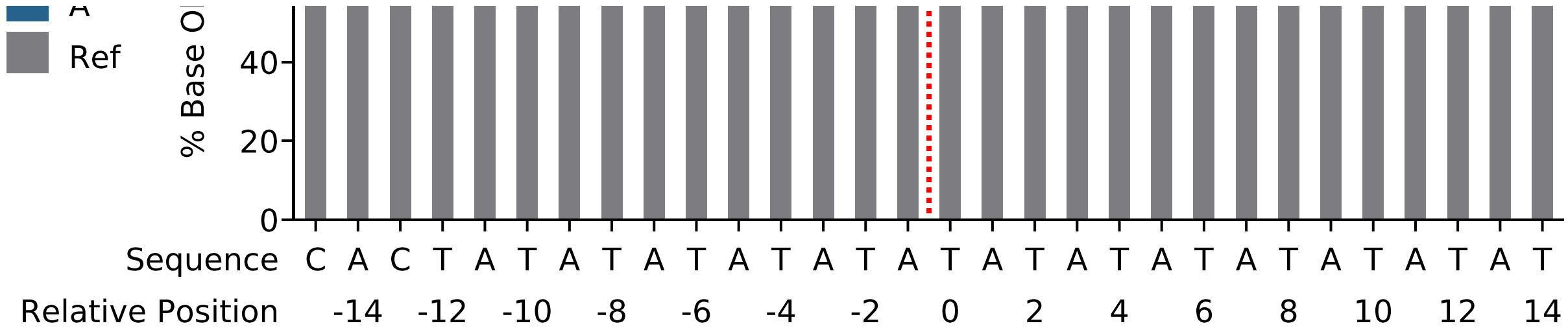

HBV_Cas12a_Control__SHISA6

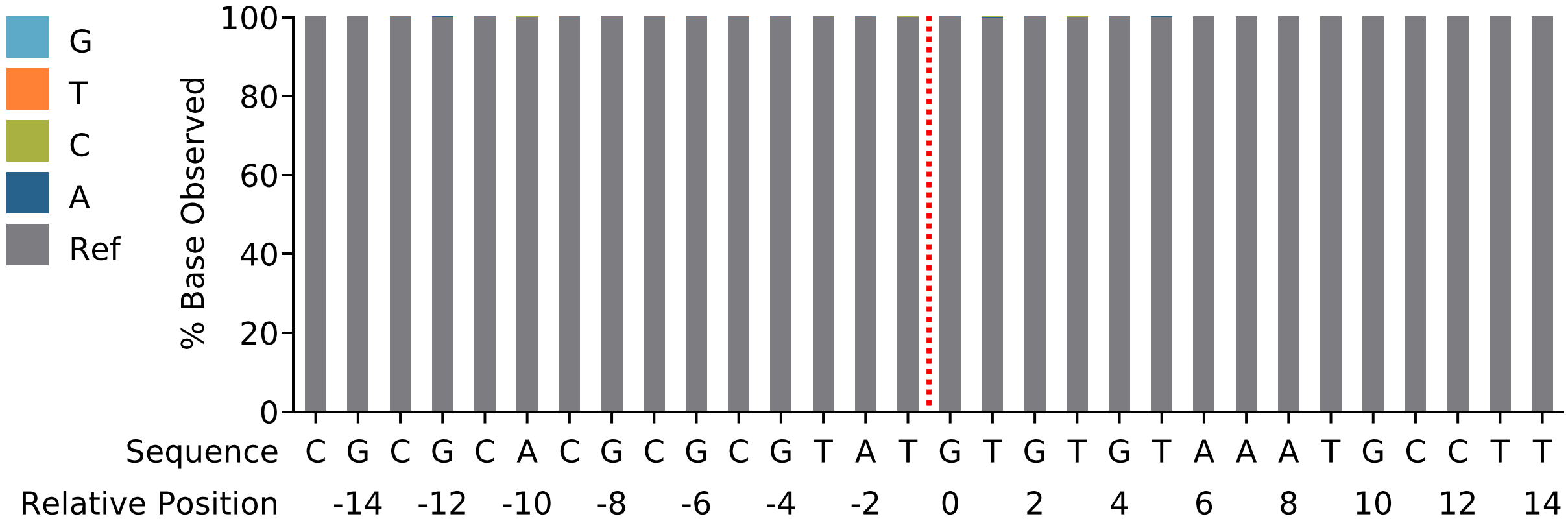

HBV_Cas12a_Control__TMC4

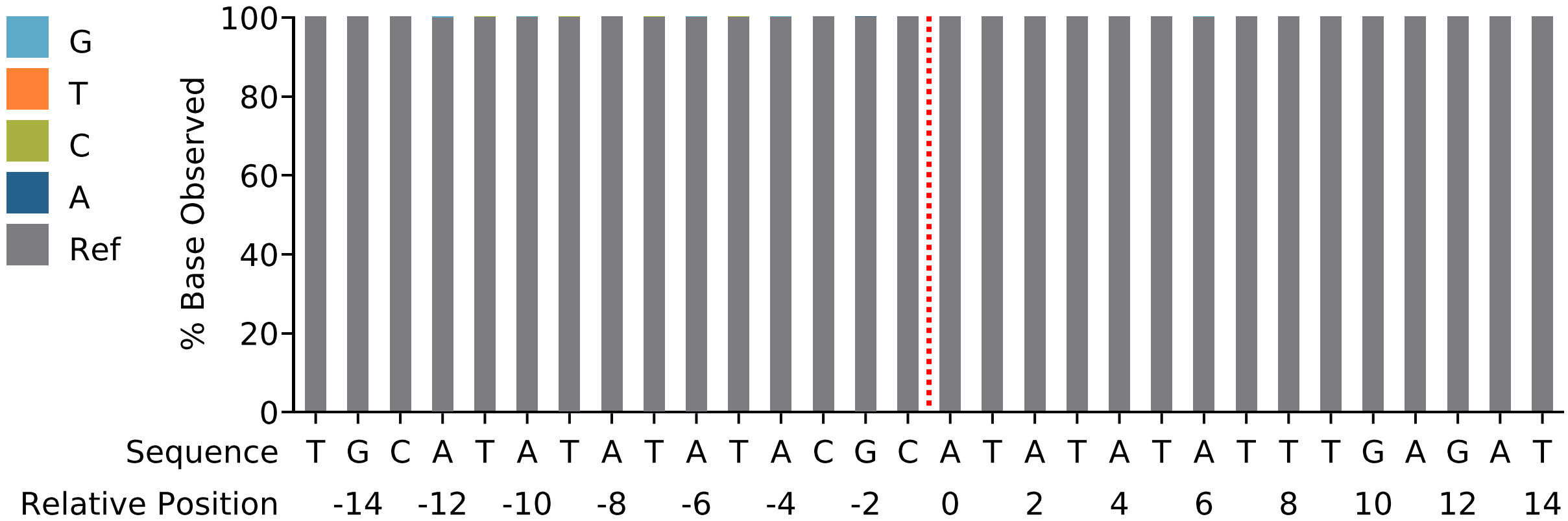

HBV_Cas12a_Control__none44

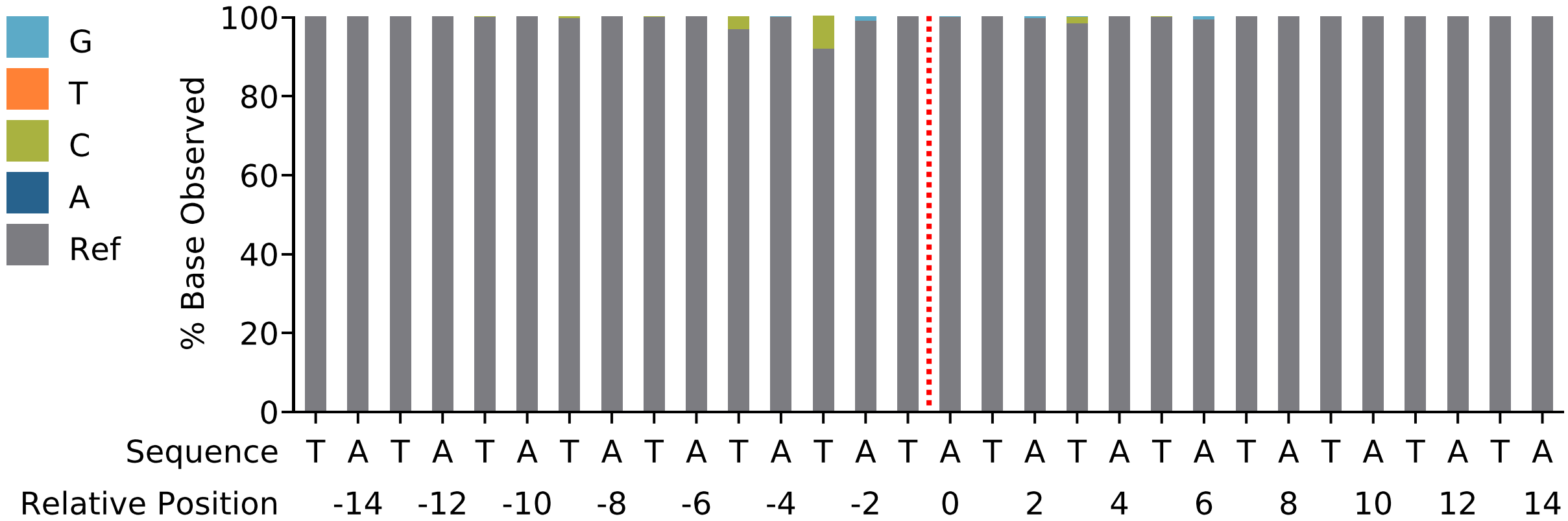

HBV_Cas12a_Control__none46

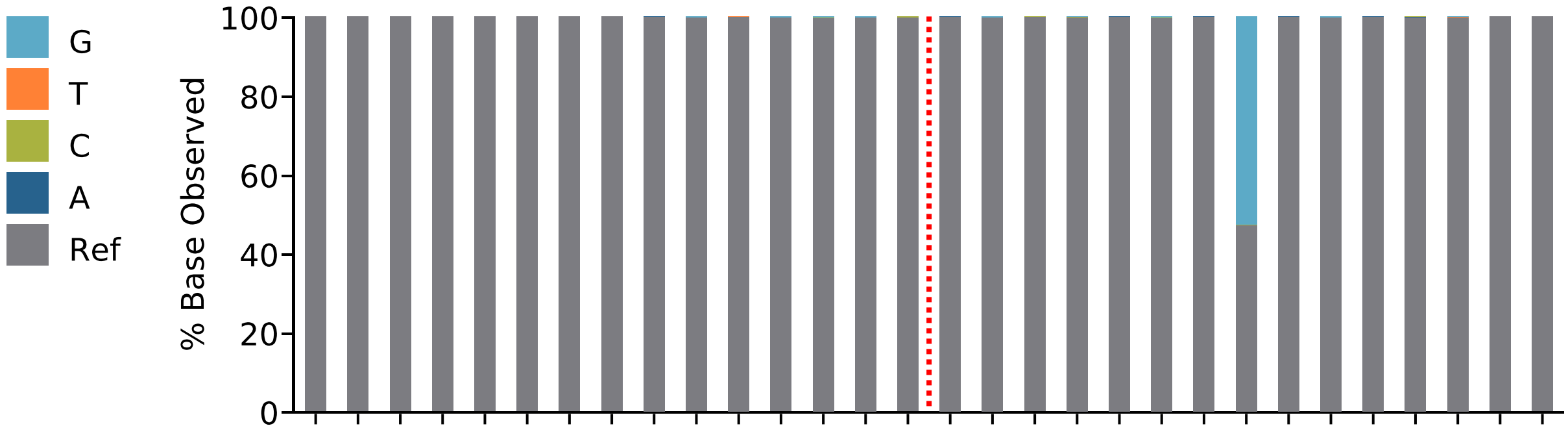

Sequence C C T A T A C A C A C A A A T C A T A C A C A C A C T A T A
Relative Position -14 -12 -10 -8 -6 -4 -2 0 2 4 6 8 10 12 14

HBV_Cas12a_Control__none47

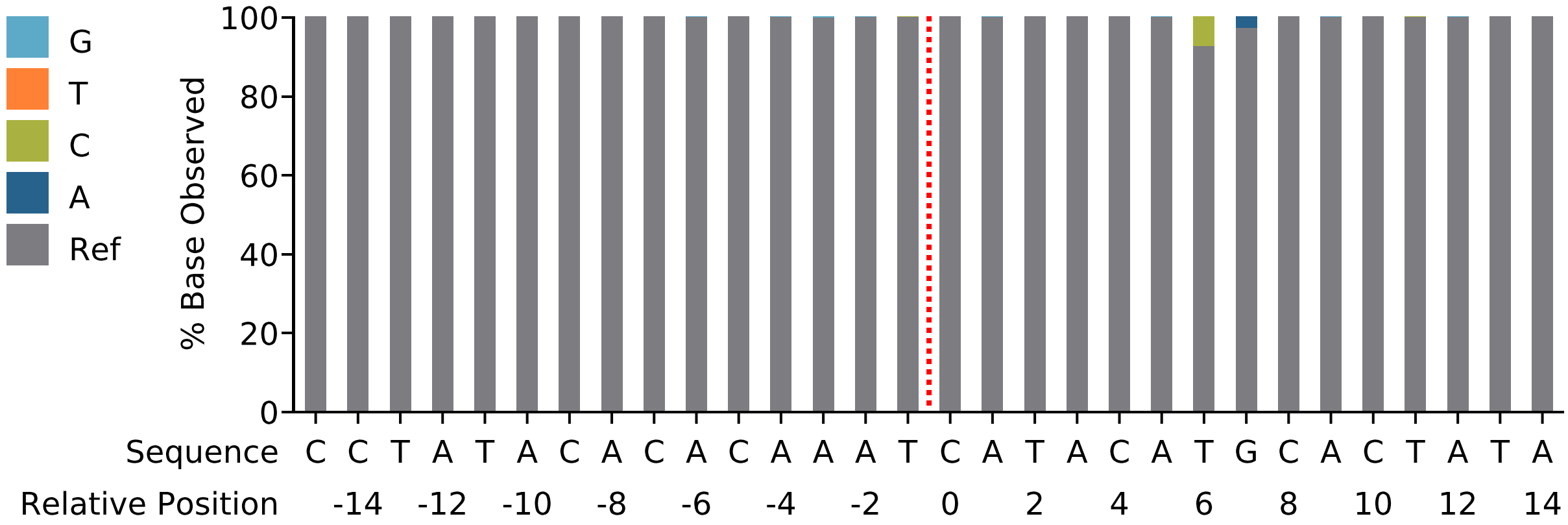

HBV_Cas12a_Control__none48

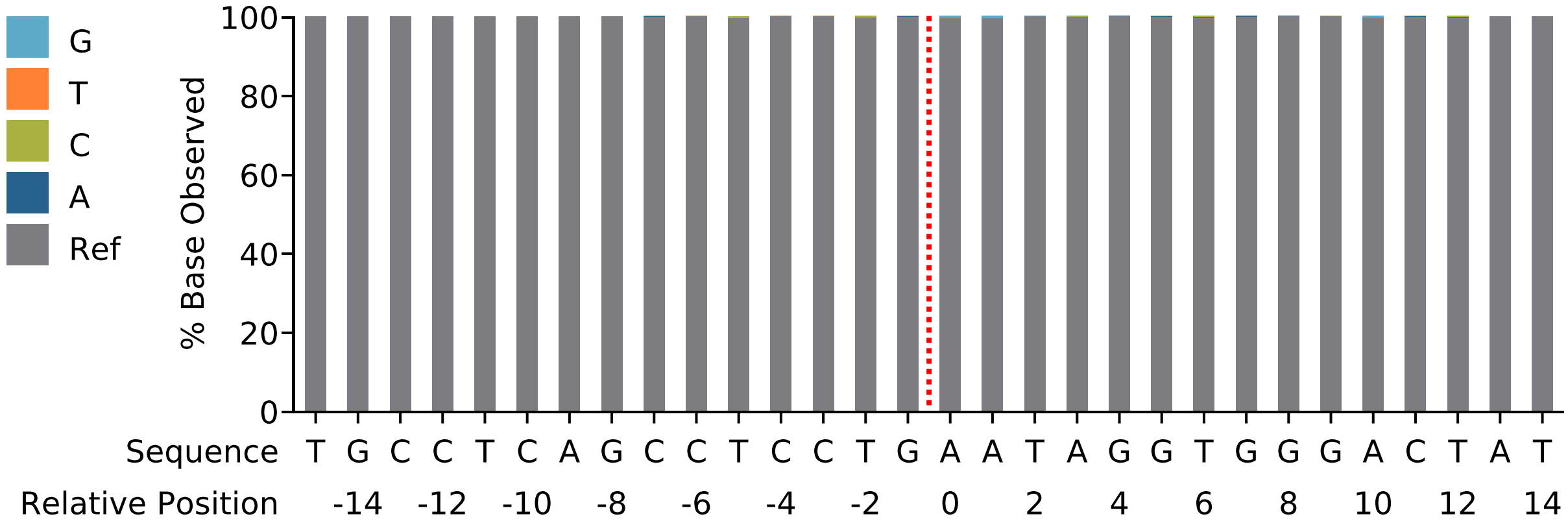

HBV_Cas12a_Control__none49

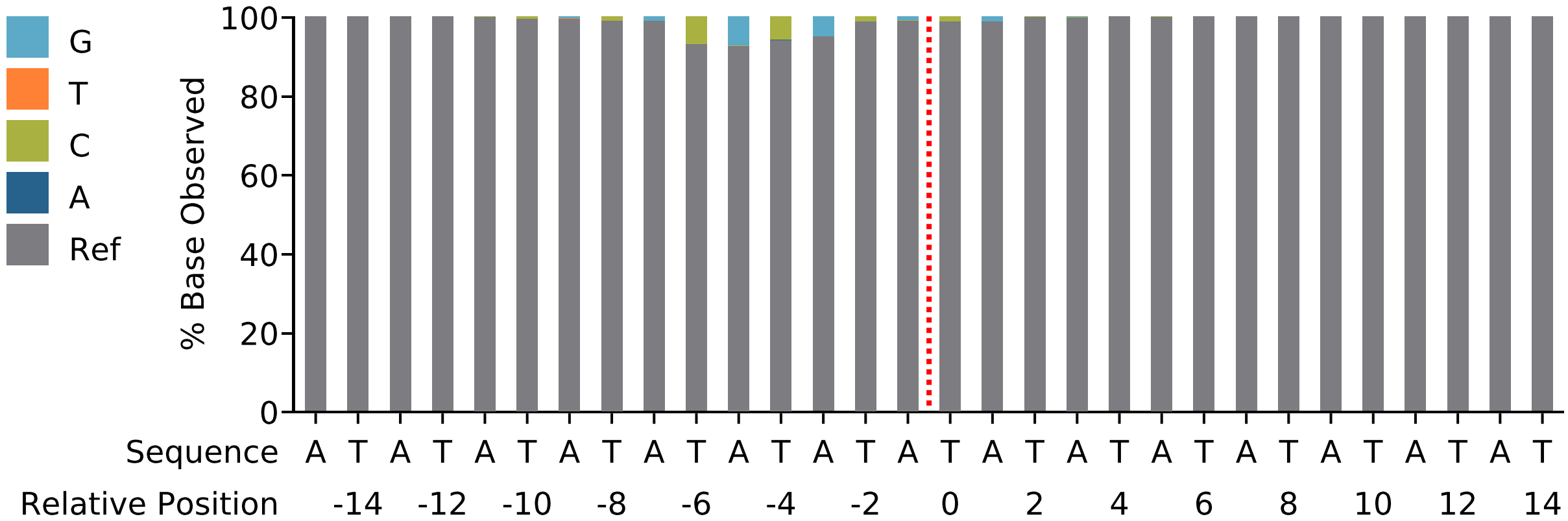

Supplement: Supplementary file 4 [file DataSheet4.pdf]
