# Supplementary material for: Combined RNA- and DNA-targeting CRISPR ribonucleoproteins enhance suppression of HBV transcripts
Source: Front Genome Ed. 2026 Sep 23;8:1824895. doi: 10.3389/fgeed.2026.1824895 (PMC13642003; doi:10.3389/fgeed.2026.1824895)

Analysis mode: CRISPAltRations 1_2_1
Start date: 2026/08/21 15:25
Targets: HBV_Cas9_EditR__ACTR3B, HBV_Cas9_EditR__CCDC60, HBV_Cas9_EditR__CLYBL, HBV_Cas9_EditR__FLYWCH1, HBV_Cas9_EditR__GALNT16, HBV_Cas9_EditR__JAM2, HBV_Cas9_EditR__LHPP, HBV_Cas9_EditR__LINC00301, HBV_Cas9_EditR__LOC105372647, HBV_Cas9_EditR__PRSS55, HBV_Cas9_EditR__RPS19BP1, HBV_Cas9_EditR__SUGP1, HBV_Cas9_EditR__TBCD, HBV_Cas9_EditR__none24, HBV_Cas9_gRNA9__DTNA, HBV_Cas9_gRNA9__LOC101929268, HBV_Cas9_gRNA9__MIR4663, HBV_Cas9_gRNA9__PARP11-AS1, HBV_Cas9_gRNA9__PDE1C, HBV_Cas9_gRNA9__PRRC2B, HBV_Cas9_gRNA9__SHISAL1, HBV_Cas9_gRNA9__none11, HBV_Cas9_gRNA9__none12, HBV_Cas9_gRNA9__none13, HBV_Cas9_gRNA9__none14, HBV_Cas9_gRNA9__none15, HBV_Cas9_gRNA9__none16, HBV_Cas9_gRNA9__none17, HBV_Cas9_gRNA9__none18, HBV_Cas9_gRNA9__none19, HBV_Cas9_gRNA9__none20, HBV_Cas9_gRNA9__none21, HBV_Cas9_gRNA9__none22

Metrics

	Group ID	Treated	Total Reads	Percent Edited	Percent Unedited	Percent NHEJ	Percent Perfect HDR	Percent Imperfect HDR	Percent HDR	Percent Other	Percent In Frame	Percent Frameshift	Percent Indels	Percent Insertions	Percent Templated Insertion	Percent GC-Insertion	Percent Deletions
**HBV_Cas9_EditR__ACTR3B**	HBV_Fig3	TRUE	7913	2.44	97.56	0.14	0.0	0.0	0.0	2.3	99.86	0.14	0.14	0.0	0.0	0.0	0.14
**HBV_Cas9_EditR__CCDC60**	HBV_Fig3	TRUE	4091	2.3	97.7	0.05	0.0	0.0	0.0	2.25	99.95	0.05	0.05	0.0	0.0	0.0	0.05
**HBV_Cas9_EditR__CLYBL**	HBV_Fig3	TRUE	24589	2.24	97.76	0.01	0.0	0.0	0.0	2.23	99.99	0.01	0.01	0.0	0.0	0.0	0.01
**HBV_Cas9_EditR__FLYWCH1**	HBV_Fig3	TRUE	17751	2.34	97.66	0.07	0.0	0.0	0.0	2.27	99.93	0.07	0.07	0.01	100.0	0.0	0.06
**HBV_Cas9_EditR__GALNT16**	HBV_Fig3	TRUE	98251	2.42	97.58	0.21	0.0	0.0	0.0	2.21	99.79	0.21	0.21	0.03	96.55	0.0	0.18
**HBV_Cas9_EditR__JAM2**	HBV_Fig3	TRUE	71677	2.71	97.29	0.04	0.0	0.0	0.0	2.67	99.96	0.04	0.04	0.01	100.0	0.0	0.03
**HBV_Cas9_EditR__LHPP**	HBV_Fig3	TRUE	16711	1.73	98.27	0.03	0.0	0.0	0.0	1.7	99.97	0.03	0.03	0.0	0.0	0.0	0.03
**HBV_Cas9_EditR__LINC00301**	HBV_Fig3	TRUE	49140	99.98	0.02	0.31	0.0	0.0	0.0	99.67	99.94	0.06	0.31	0.01	50.0	0.0	0.3
**HBV_Cas9_EditR__LOC105372647**	HBV_Fig3	TRUE	1188625	88.53	11.47	0.71	0.0	0.0	0.0	87.82	99.32	0.68	0.71	0.4	2.92	0.0	0.33
**HBV_Cas9_EditR__PRSS55**	HBV_Fig3	TRUE	82182	99.89	0.11	0.08	0.0	0.0	0.0	99.81	99.92	0.08	0.08	0.0	100.0	0.0	0.08
**HBV_Cas9_EditR__RPS19BP1**	HBV_Fig3	TRUE	16205	2.26	97.74	0.06	0.0	0.0	0.0	2.2	99.94	0.06	0.06	0.0	0.0	0.0	0.06
**HBV_Cas9_EditR__SUGP1**	HBV_Fig3	TRUE	17422	2.2	97.8	0.12	0.0	0.0	0.0	2.08	99.88	0.12	0.12	0.0	0.0	0.0	0.12
**HBV_Cas9_EditR__TBCD**	HBV_Fig3	TRUE	9857	2.39	97.61	0.07	0.0	0.0	0.0	2.32	99.93	0.07	0.07	0.01	100.0	0.0	0.06
**HBV_Cas9_EditR__none24**	HBV_Fig3	TRUE	38592	2.87	97.13	0.04	0.0	0.0	0.0	2.83	99.96	0.04	0.04	0.0	0.0	0.0	0.04
**HBV_Cas9_gRNA9__DTNA**	HBV_Fig3	TRUE	29173	68.38	31.62	0.03	0.0	0.0	0.0	68.35	99.97	0.03	0.03	0.01	0.0	0.0	0.02
**HBV_Cas9_gRNA9__LOC101929268**	HBV_Fig3	TRUE	64815	3.15	96.85	0.03	0.0	0.0	0.0	3.12	99.97	0.03	0.03	0.0	100.0	0.0	0.03
**HBV_Cas9_gRNA9__MIR4663**	HBV_Fig3	TRUE	73406	3.11	96.89	0.14	0.0	0.0	0.0	2.97	99.86	0.14	0.14	0.13	0.0	0.0	0.14
**HBV_Cas9_gRNA9__PARP11-AS1**	HBV_Fig3	TRUE	80483	2.63	97.37	0.02	0.0	0.0	0.0	2.61	99.98	0.02	0.02	0.0	0.0	0.0	0.02
**HBV_Cas9_gRNA9__PDE1C**	HBV_Fig3	TRUE	52652	2.29	97.71	0.13	0.0	0.0	0.0	2.16	99.87	0.13	0.13	0.0	100.0	0.0	0.13
**HBV_Cas9_gRNA9__PRRC2B**	HBV_Fig3	TRUE	46375	2.51	97.49	0.26	0.0	0.0	0.0	2.25	99.74	0.26	0.26	0.05	100.0	0.0	0.21
**HBV_Cas9_gRNA9__SHISAL1**	HBV_Fig3	TRUE	60585	2.76	97.24	0.12	0.0	0.0	0.0	2.64	99.88	0.12	0.12	0.02	85.71	0.0	0.1
**HBV_Cas9_gRNA9__none11**	HBV_Fig3	TRUE	3860	2.93	97.07	0.0	0.0	0.0	0.0	2.93	100.0	0.0	0.0	0.0	0.0	0.0	0.0
**HBV_Cas9_gRNA9__none12**	HBV_Fig3	TRUE	19866	2.49	97.51	0.08	0.0	0.0	0.0	2.41	99.96	0.04	0.08	0.02	100.0	0.0	0.06
**HBV_Cas9_gRNA9__none13**	HBV_Fig3	TRUE	1705	55.78	44.22	53.43	0.0	0.0	0.0	2.35	46.63	53.37	53.43	0.0	0.0	0.0	53.43
**HBV_Cas9_gRNA9__none14**	HBV_Fig3	TRUE	67784	3.18	96.82	0.19	0.0	0.0	0.0	2.99	99.81	0.19	0.19	0.04	100.0	0.0	0.15
**HBV_Cas9_gRNA9__none15**	HBV_Fig3	TRUE	22295	2.44	97.56	0.0	0.0	0.0	0.0	2.44	100.0	0.0	0.0	0.0	0.0	0.0	0.0
**HBV_Cas9_gRNA9__none16**	HBV_Fig3	TRUE	52	7.69	92.31	0.0	0.0	0.0	0.0	7.69	100.0	0.0	0.0	0.0	0.0	0.0	0.0
**HBV_Cas9_gRNA9__none17**	HBV_Fig3	TRUE	82137	2.74	97.26	0.01	0.0	0.0	0.0	2.73	99.99	0.01	0.01	0.0	100.0	0.0	0.01
**HBV_Cas9_gRNA9__none18**	HBV_Fig3	TRUE	164216	5.55	94.45	0.89	0.0	0.0	0.0	4.66	99.11	0.89	0.89	0.01	0.0	0.0	0.88
**HBV_Cas9_gRNA9__none19**	HBV_Fig3	TRUE	49552	2.7	97.3	0.01	0.0	0.0	0.0	2.69	99.99	0.01	0.01	0.0	0.0	0.0	0.01
**HBV_Cas9_gRNA9__none20**	HBV_Fig3	TRUE	58775	28.6	71.4	0.02	0.0	0.0	0.0	28.58	99.99	0.01	0.02	0.01	0.0	0.0	0.01

**HBV_Cas9_gRNA9__none21**	HBV_Fig3	TRUE	48009	2.73	97.27	0.32	0.0	0.0	0.0	2.41	99.68	0.32	0.32	0.05	73.08	0.0	0.27	
**HBV_Cas9_gRNA9__none22**	HBV_Fig3	TRUE	49173	2.62	97.38	0.04	0.0	0.0	0.0	2.58	99.96	0.04	0.04	0.01	0.0	0.0	0.03	

Percentages

	% Edited	*% Repair Pathways*	*% Frameshift*
HBV_Cas9_EditR__ACTR3B	97.56%	97.56%	99.86%
HBV_Cas9_EditR__CCDC60	97.7%	97.7%	99.95%
HBV_Cas9_EditR__CLYBL	97.76%	97.76%	99.99%
HBV_Cas9_EditR__FLYWCH1	97.66%	97.66%	99.93%
HBV_Cas9_EditR__GALNT16	97.58%	97.58%	99.79%
HBV_Cas9_EditR__JAM2	97.29%	97.29%	99.96%
HBV_Cas9_EditR__LHPP	98.27%	98.27%	99.97%
HBV_Cas9_EditR__LINC00301	99.98%	99.67%	99.94%
HBV_Cas9_EditR__LOC105372647	.47 88.53%	.47 87.82%	99.32%
HBV_Cas9_EditR__PRSS55	99.89%	99.81%	99.92%
HBV_Cas9_EditR__RPS19BP1	97.74%	97.74%	99.94%
HBV_Cas9_EditR__SUGP1	97.8%	97.8%	99.88%
HBV_Cas9_EditR__TBCD	97.61%	97.61%	99.93%
HBV_Cas9_EditR__none24	97.13%	97.13%	99.96%
HBV_Cas9_gRNA9__DTNA	31.62% 68.38%	31.62% 68.35%	99.97%
HBV_Cas9_gRNA9__LOC101929268	96.85%	96.85%	99.97%
HBV_Cas9_gRNA9__MIR4663	96.89%	96.89%	99.86%
HBV_Cas9_gRNA9__PARP11-AS1	97.37%	97.37%	99.98%
HBV_Cas9_gRNA9__PDE1C	97.71%	97.71%	99.87%
HBV_Cas9_gRNA9__PRRC2B	97.49%	97.49%	99.74%
HBV_Cas9_gRNA9__SHISAL1	97.24%	97.24%	99.88%
HBV_Cas9_gRNA9__none11	97.07%	97.07%	100.0%
HBV_Cas9_gRNA9__none12	97.51%	97.51%	99.96%
HBV_Cas9_gRNA9__none13	44.22% 55.78%	44.22% 53.43%	46.63% 53.37%
HBV_Cas9_gRNA9__none14	96.82%	96.82%	99.81%
HBV_Cas9_gRNA9__none15	97.56%	97.56%	100.0%

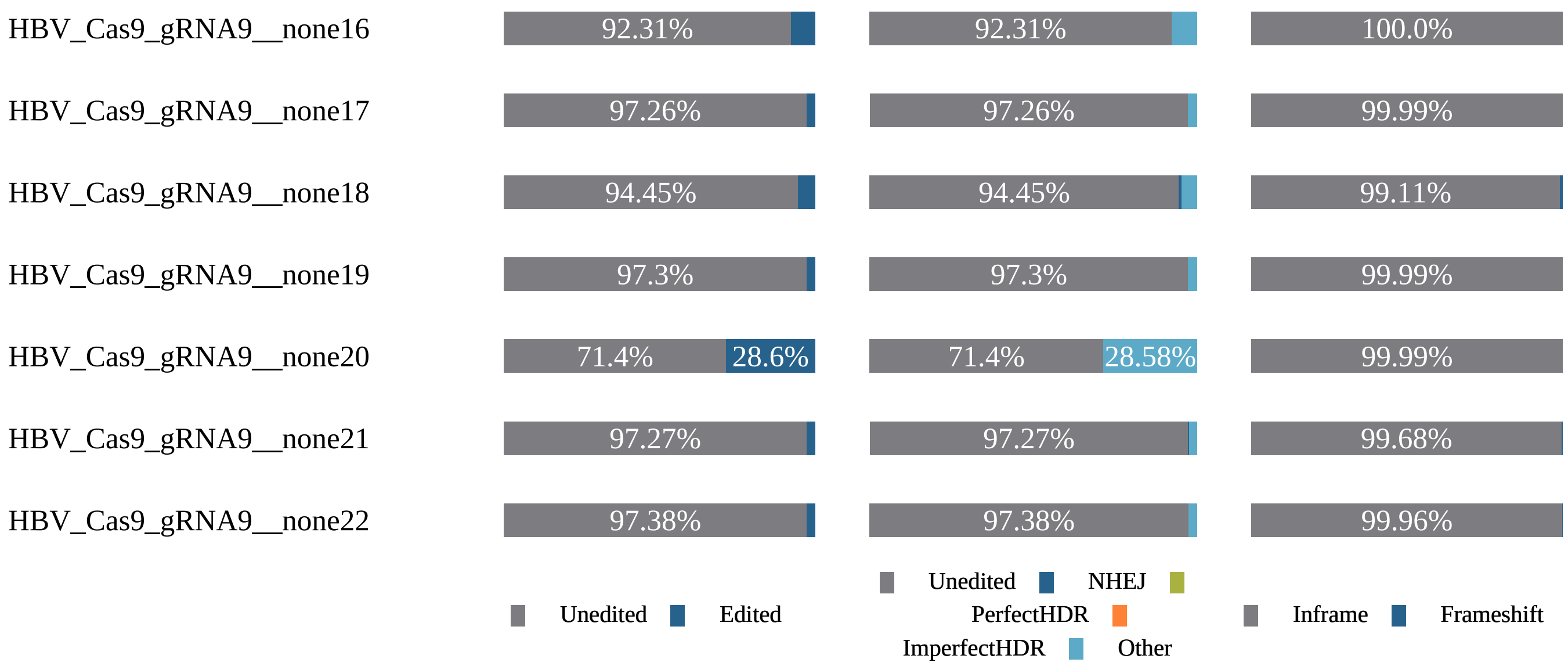

Insertions

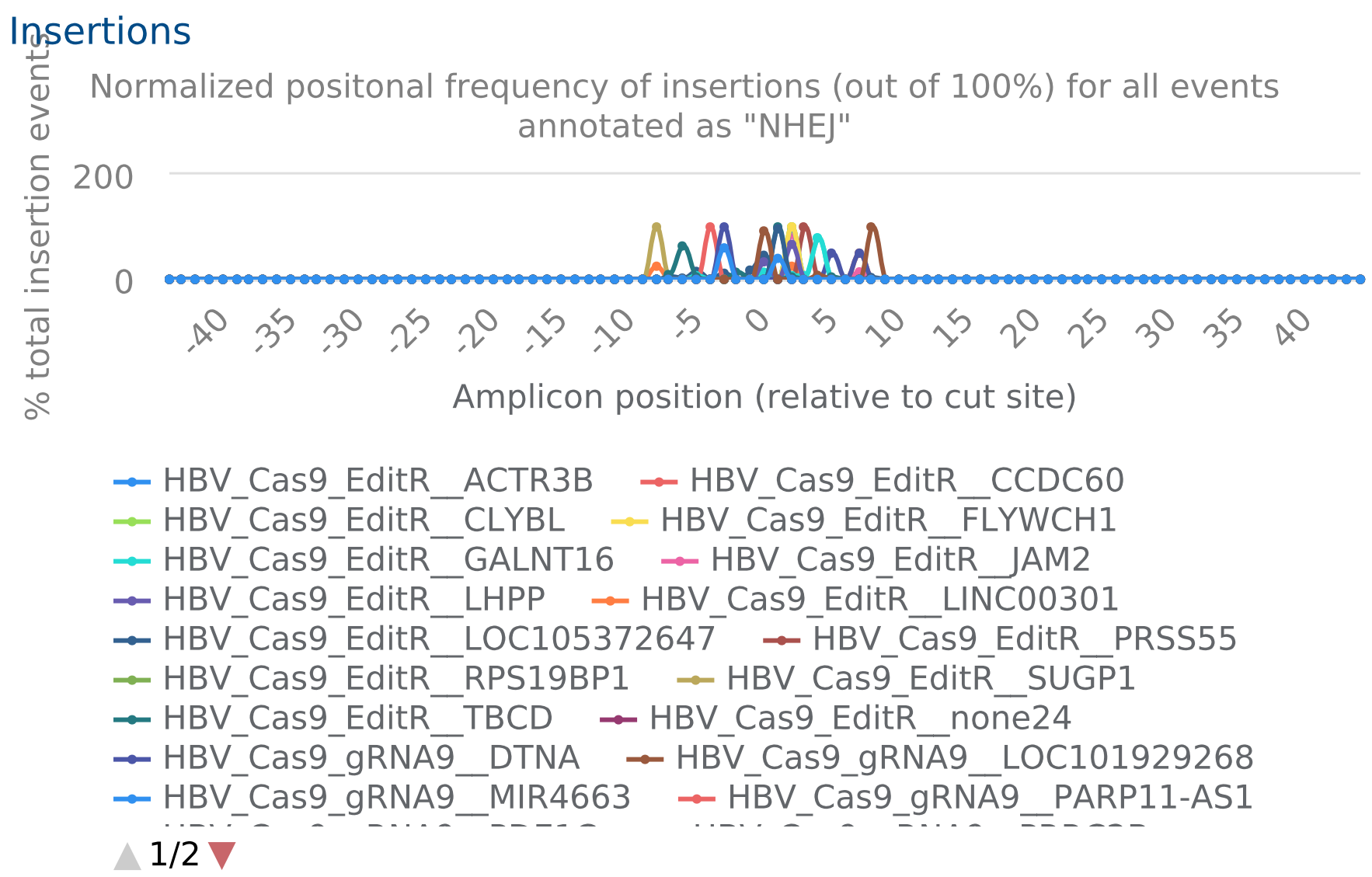

Deletions

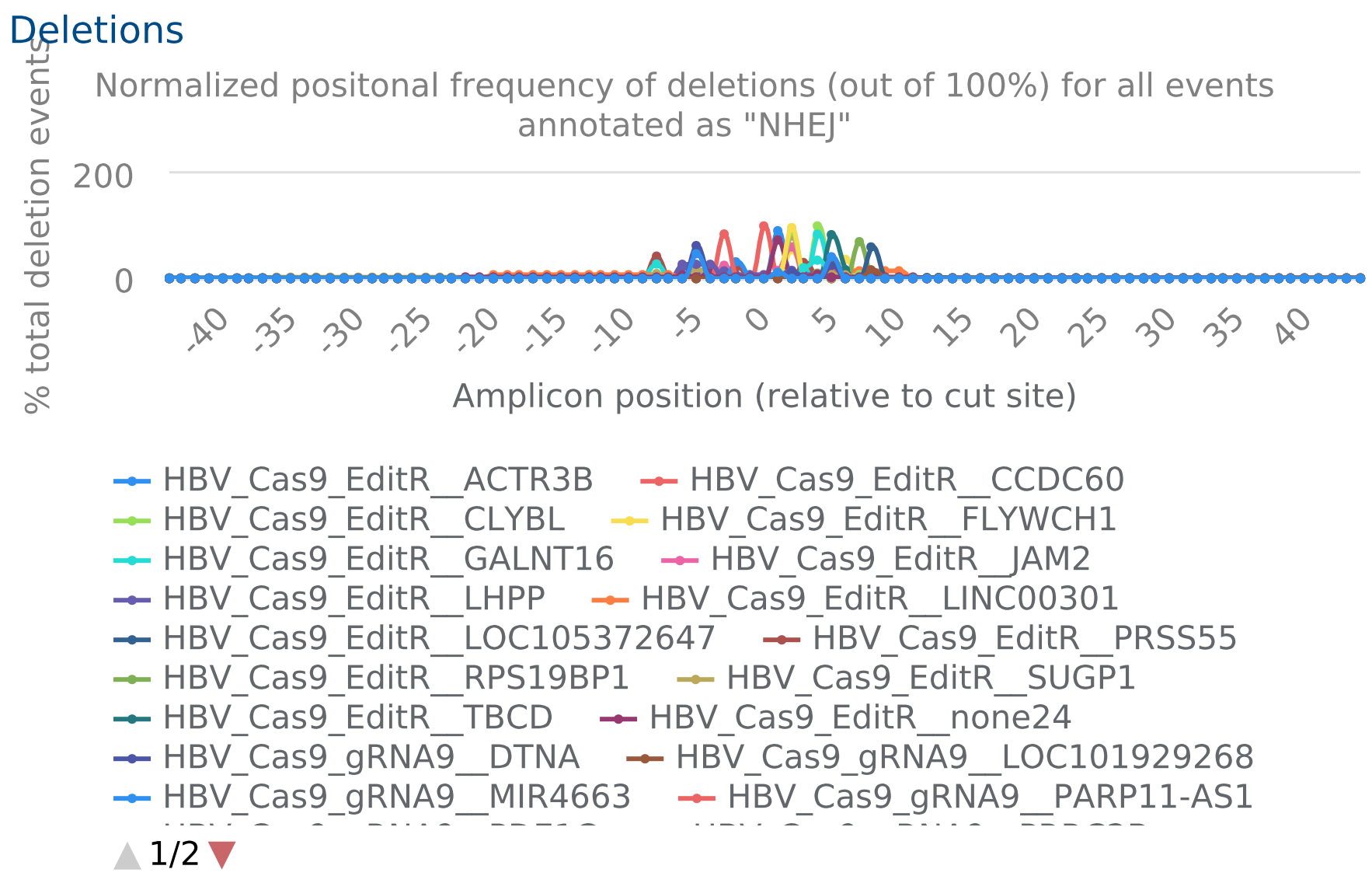

Indel Frequencies

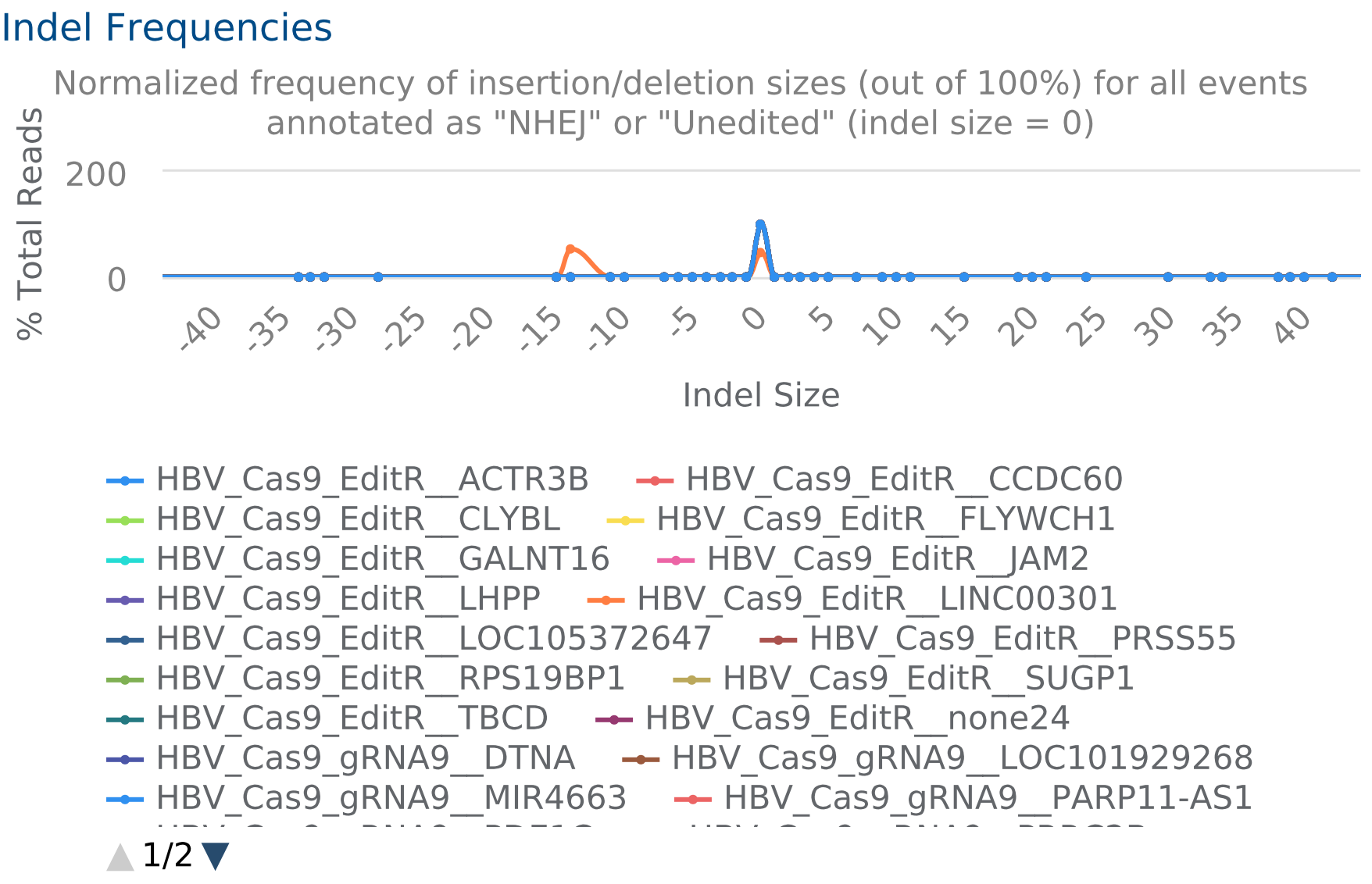

SNP Graph

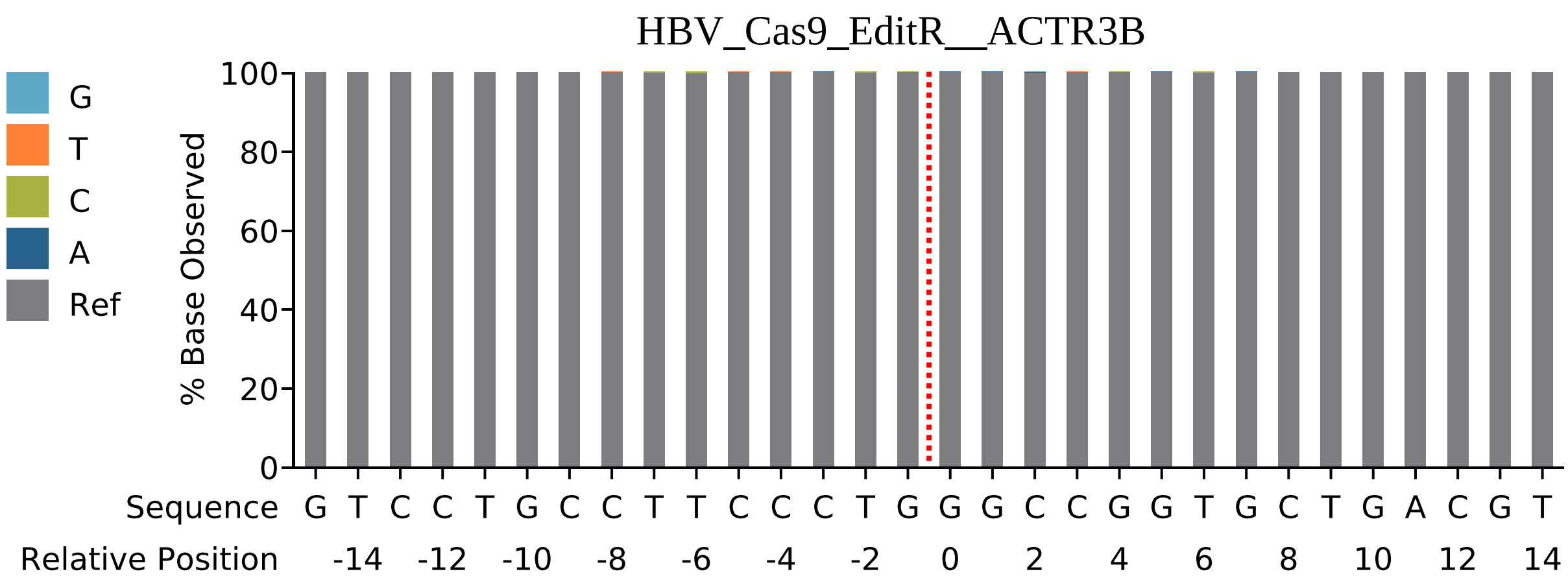

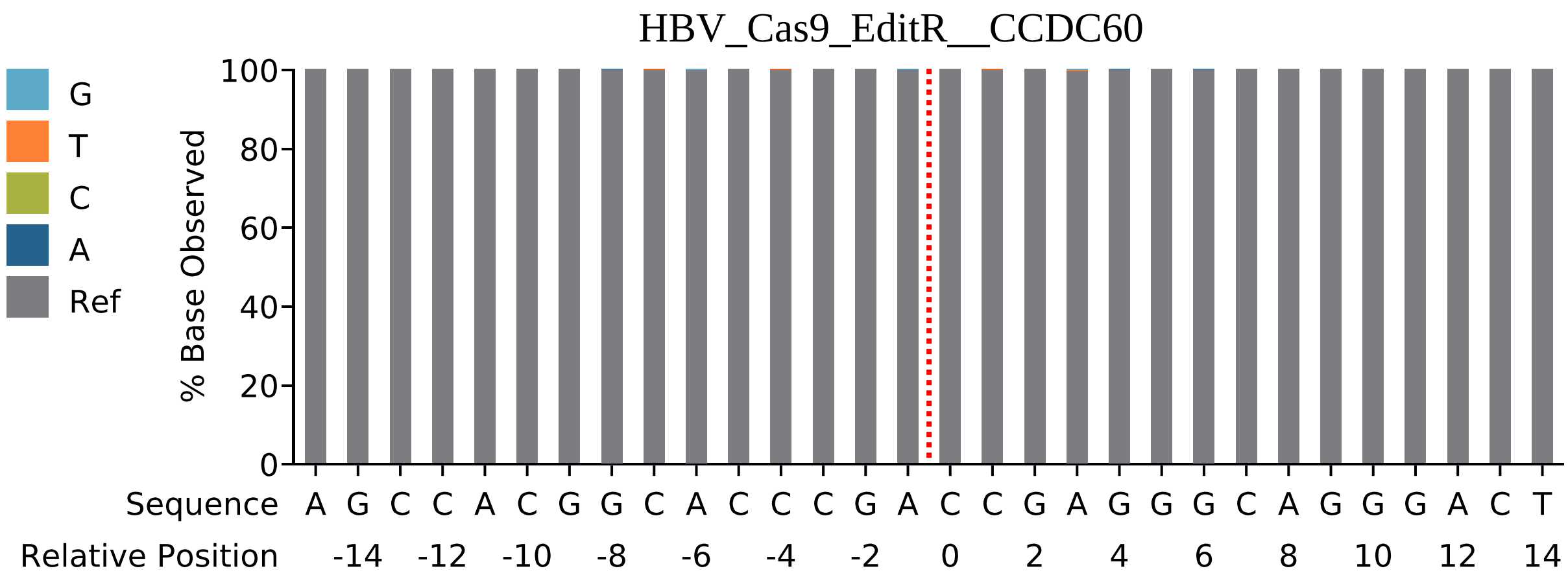

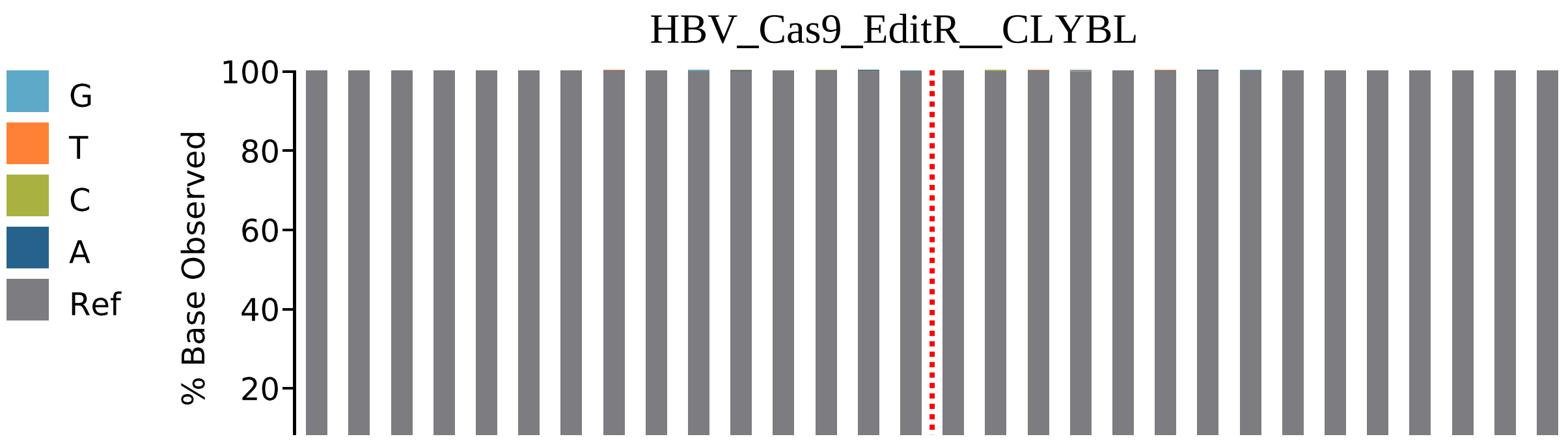

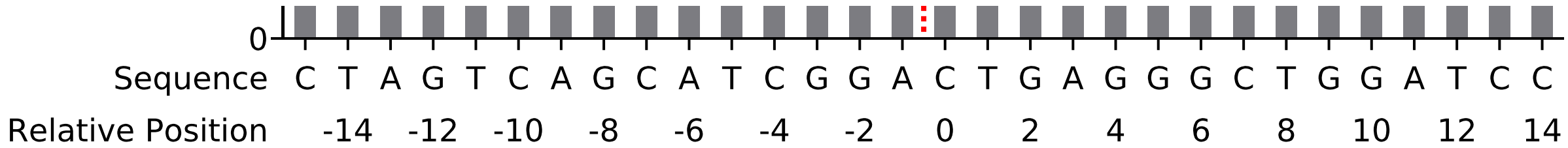

HBV_Cas9_EditR__FLYWCH1

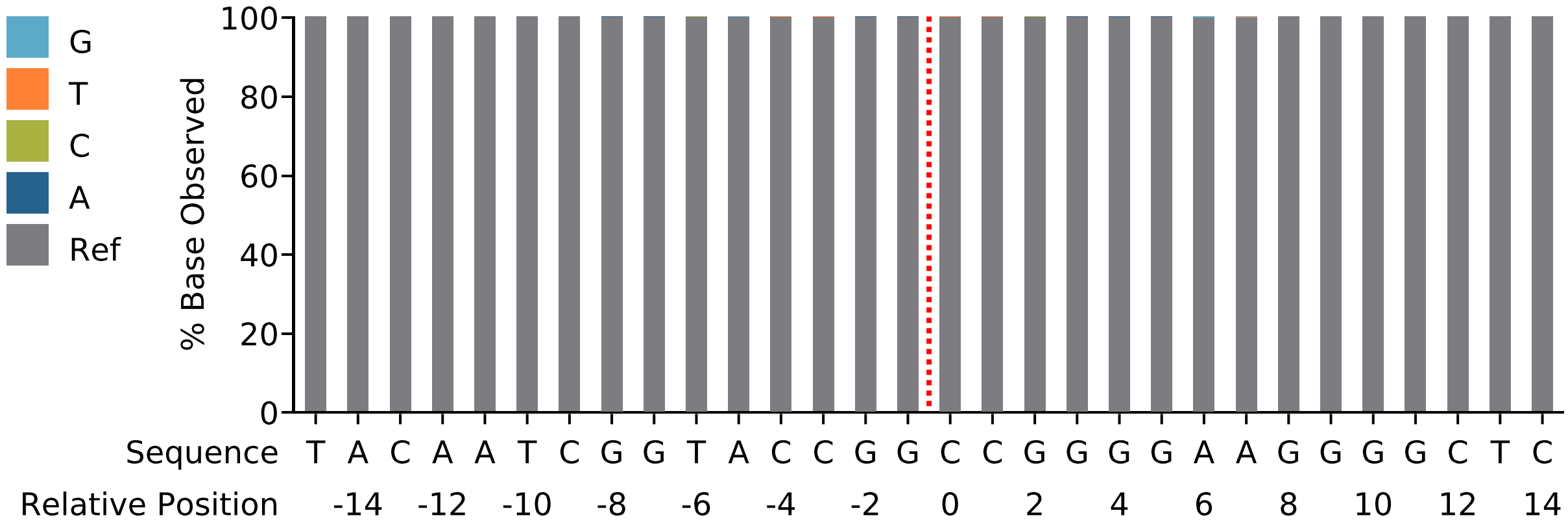

HBV_Cas9_EditR__GALNT16

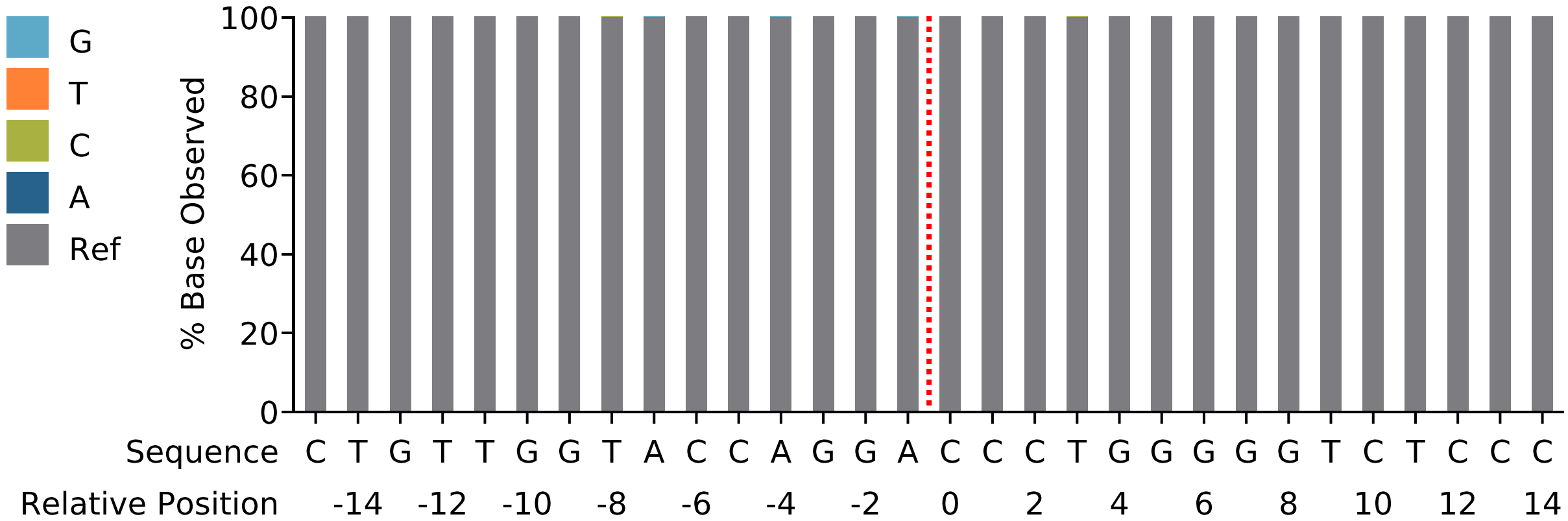

HBV_Cas9_EditR__JAM2

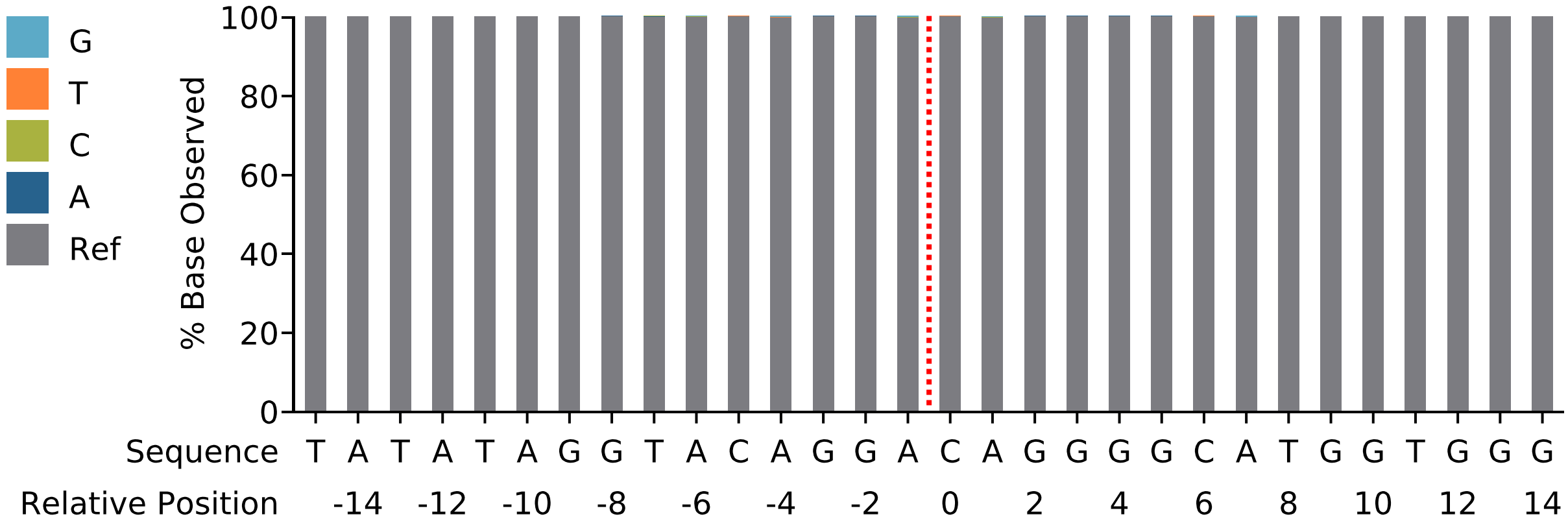

HBV_Cas9_EditR__LHPP

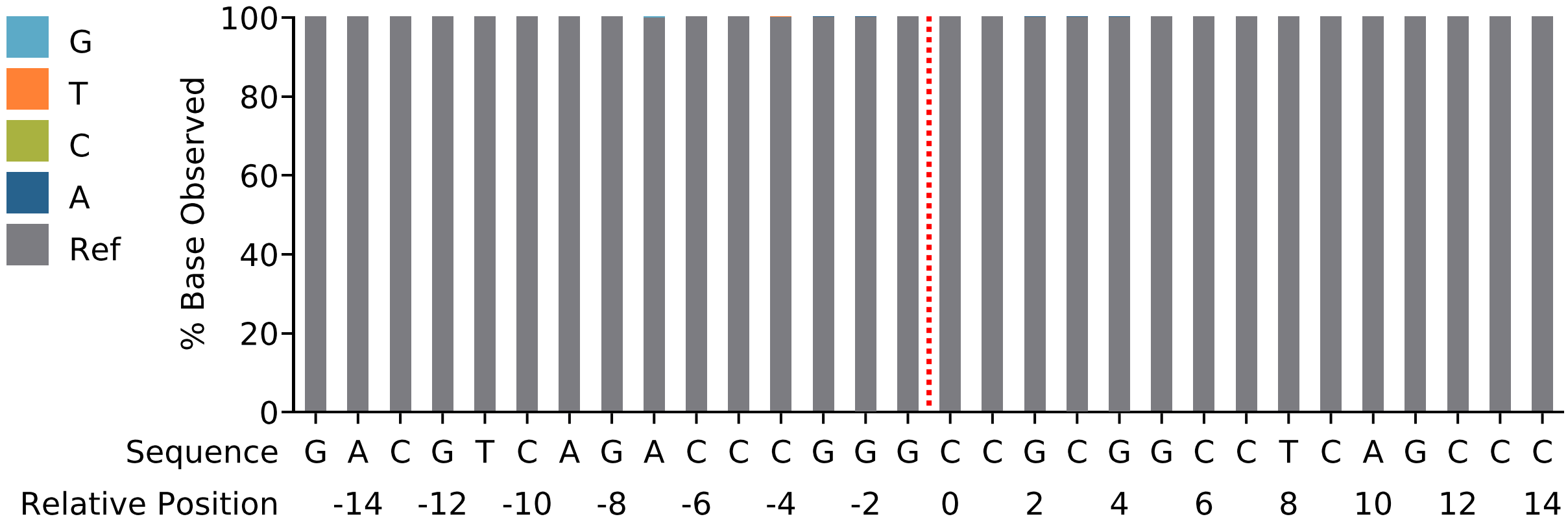

HBV_Cas9_EditR__LINC00301

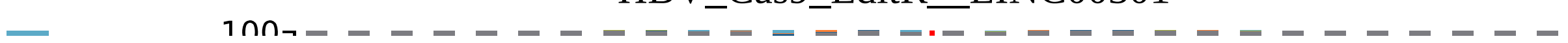

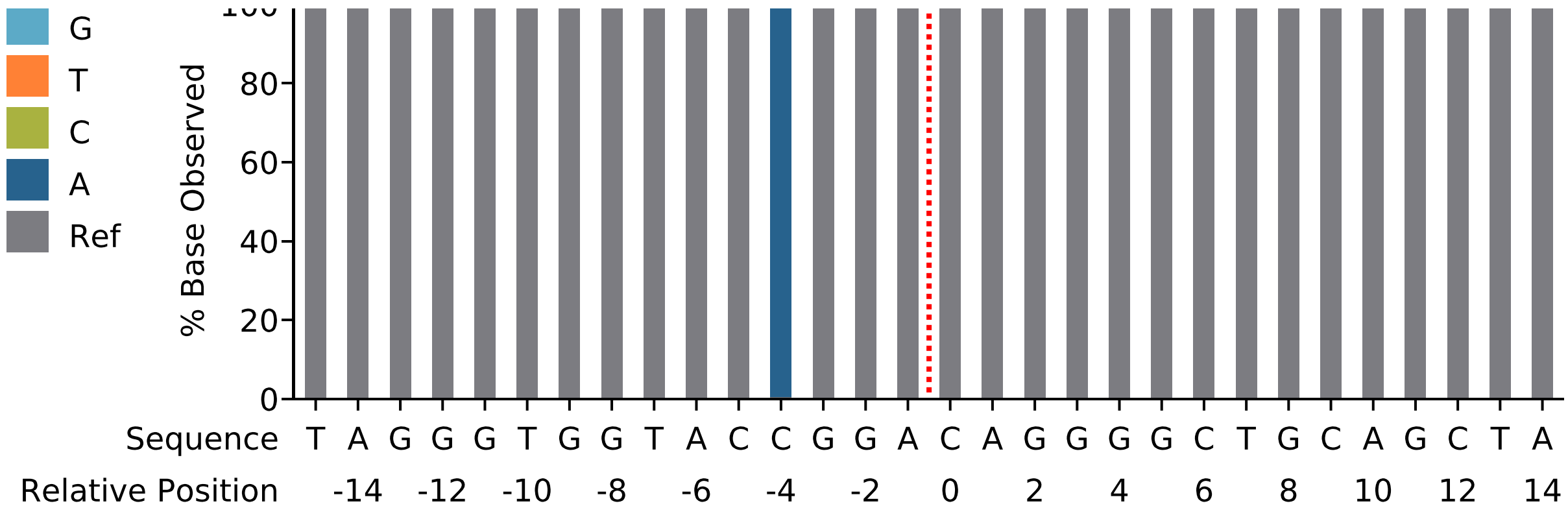

HBV_Cas9_EditR__LOC105372647

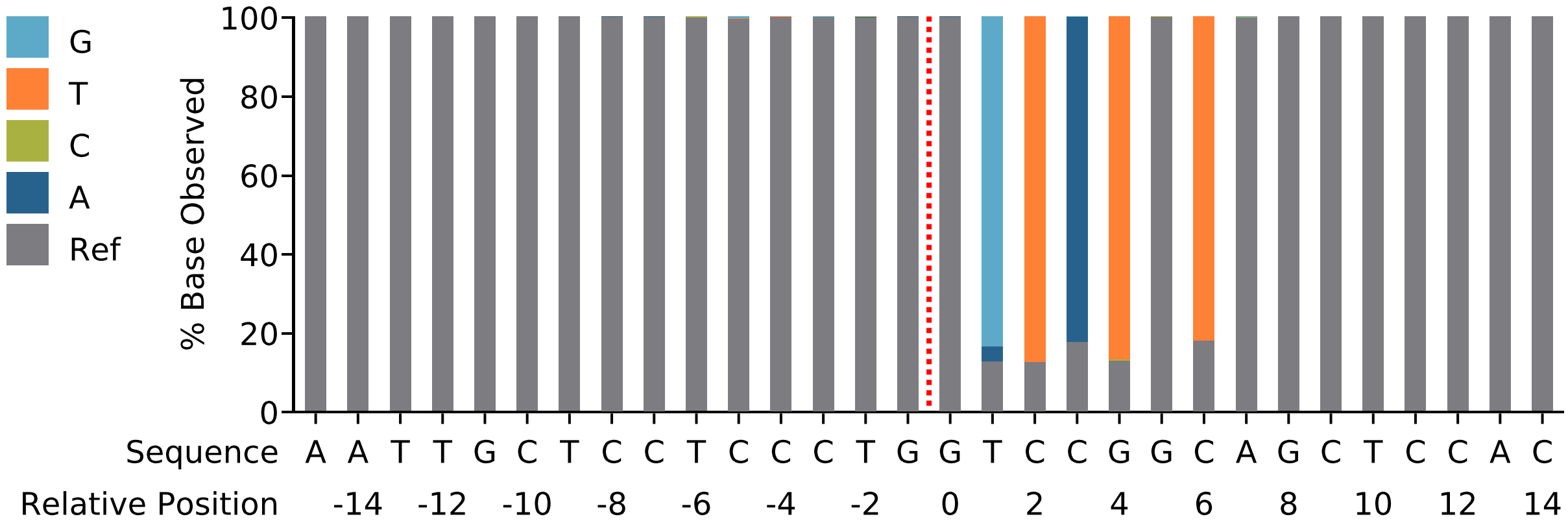

HBV_Cas9_EditR__PRSS55

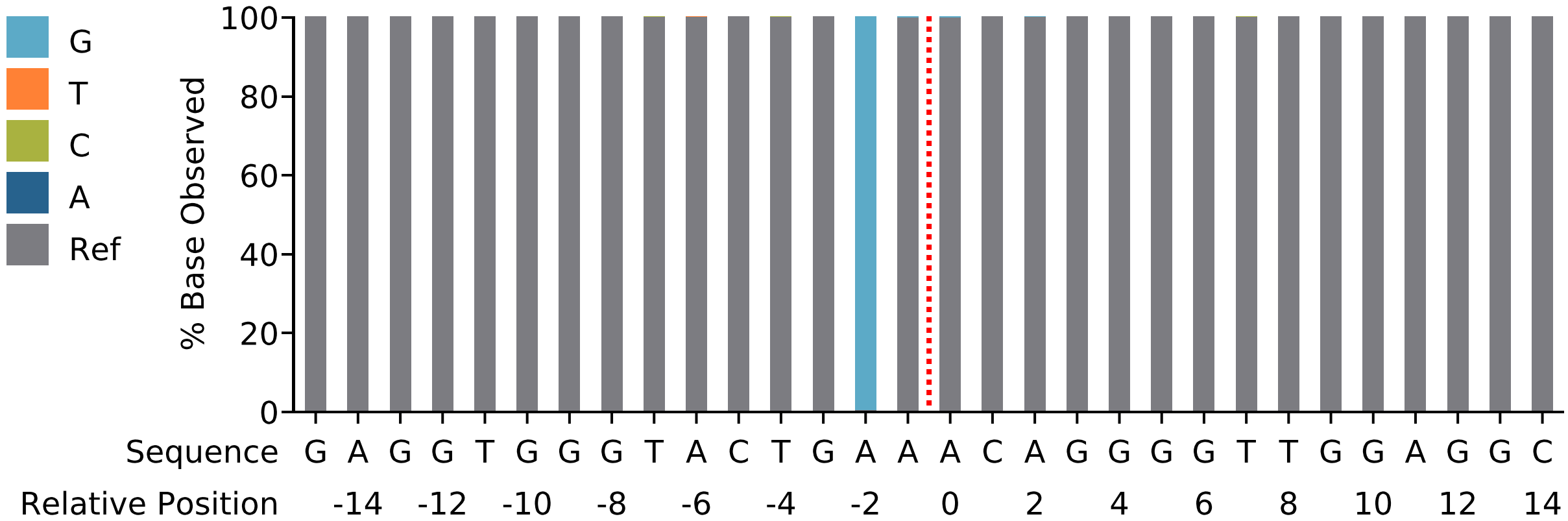

HBV_Cas9_EditR__RPS19BP1

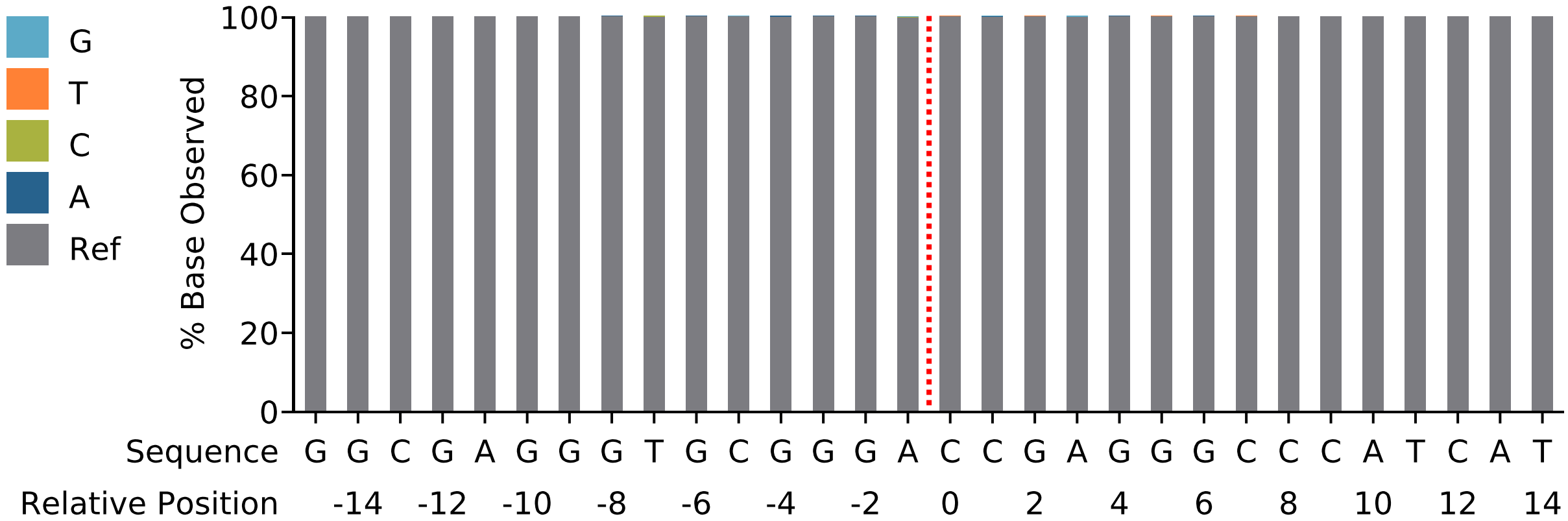

HBV_Cas9_EditR__SUGP1

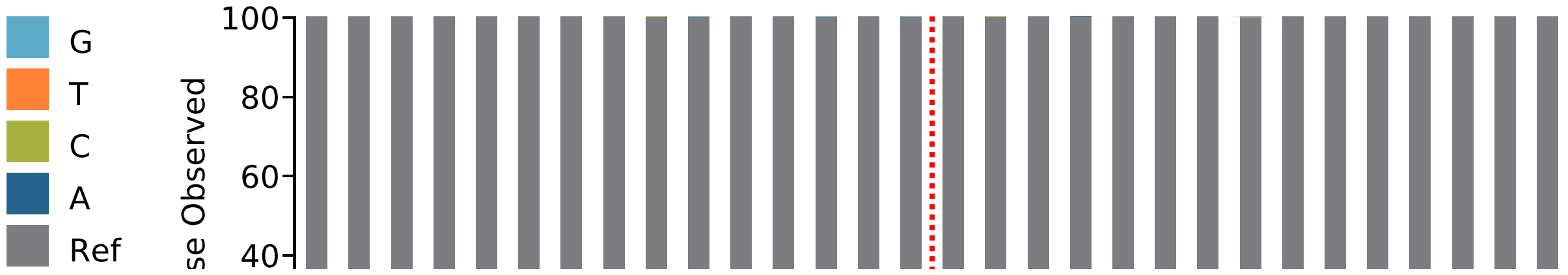

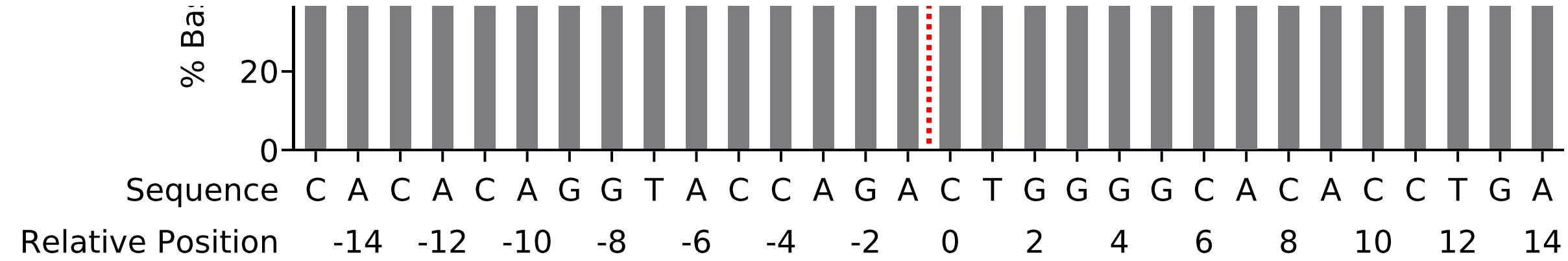

HBV_Cas9_EditR__TBCD

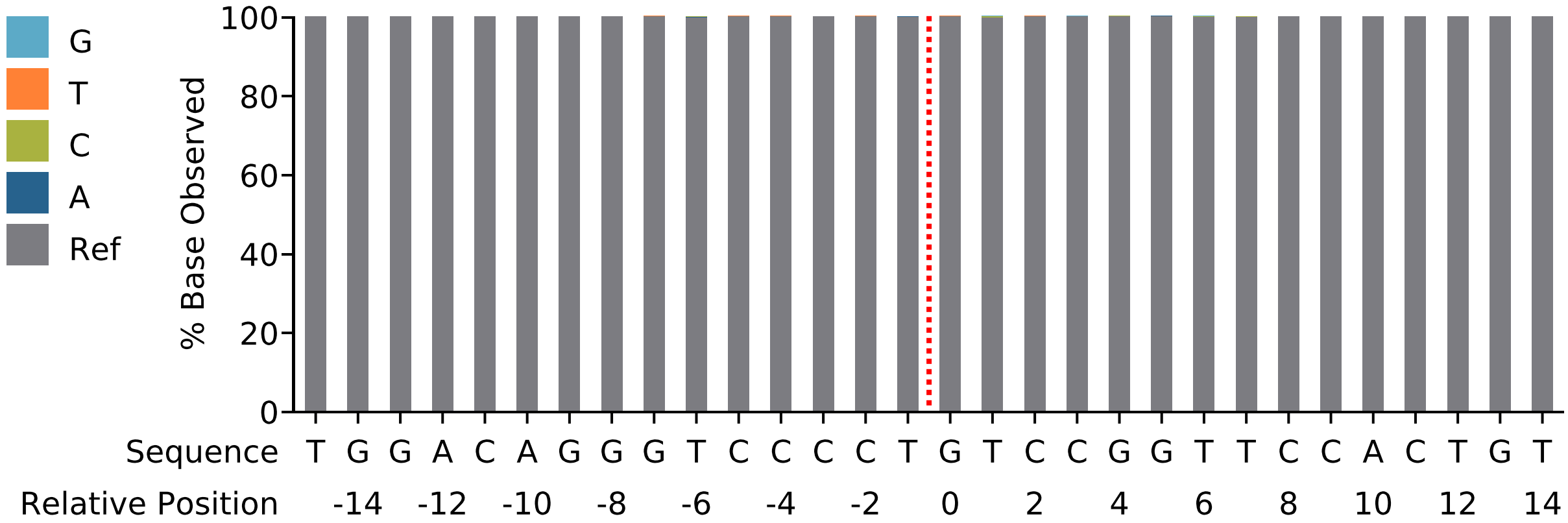

HBV_Cas9_EditR__none24

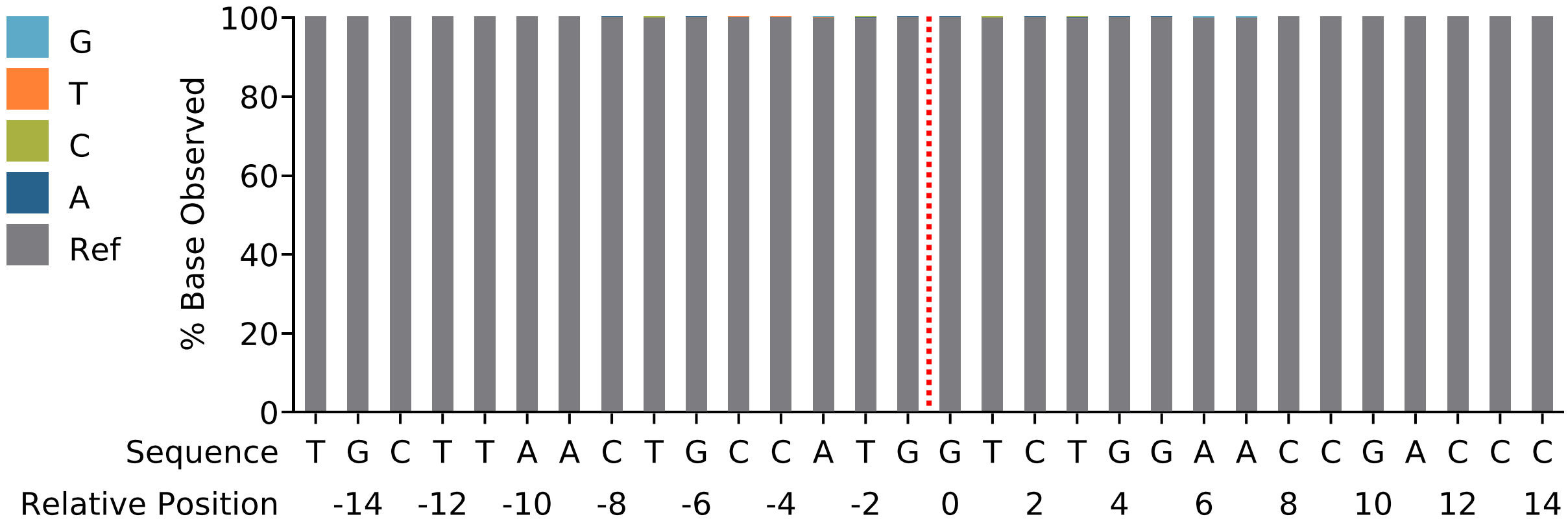

HBV_Cas9_gRNA9__DTNA

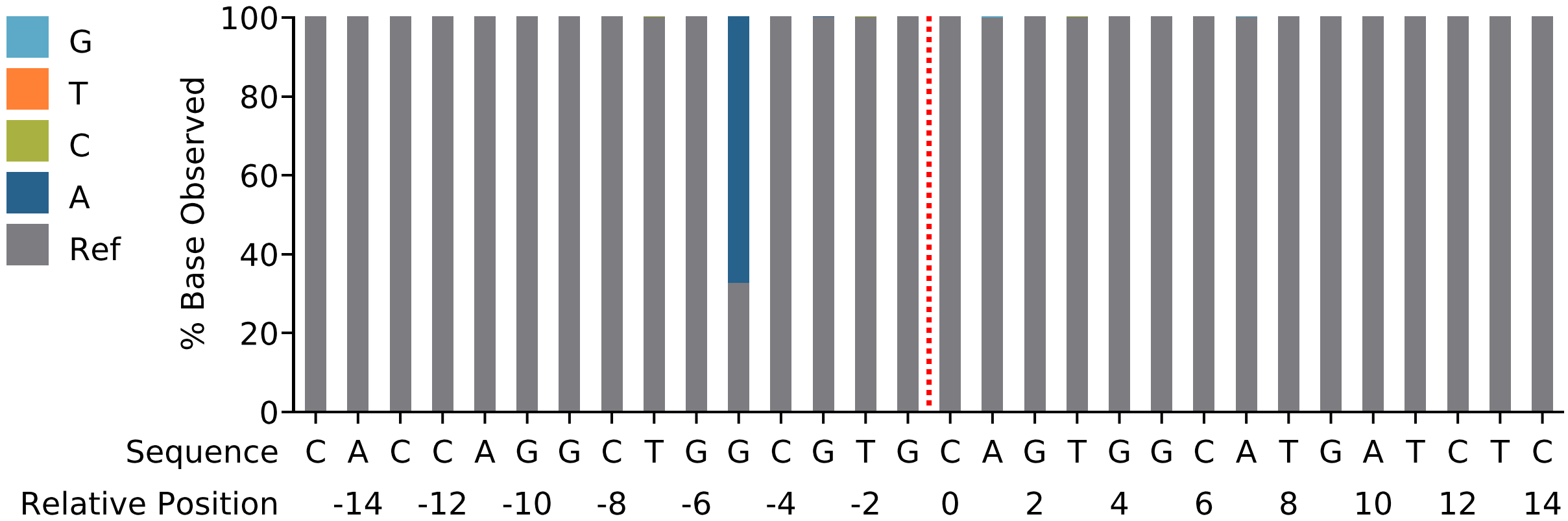

HBV_Cas9_gRNA9__LOC101929268

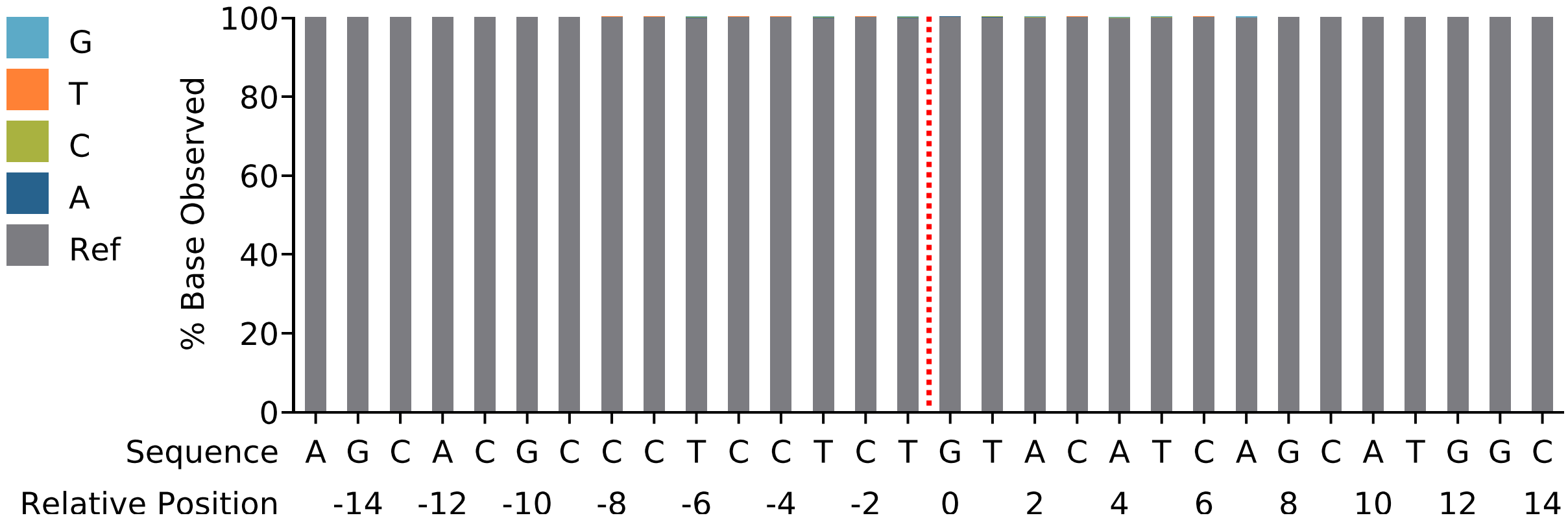

HBV_Cas9_gRNA9__MIR4663

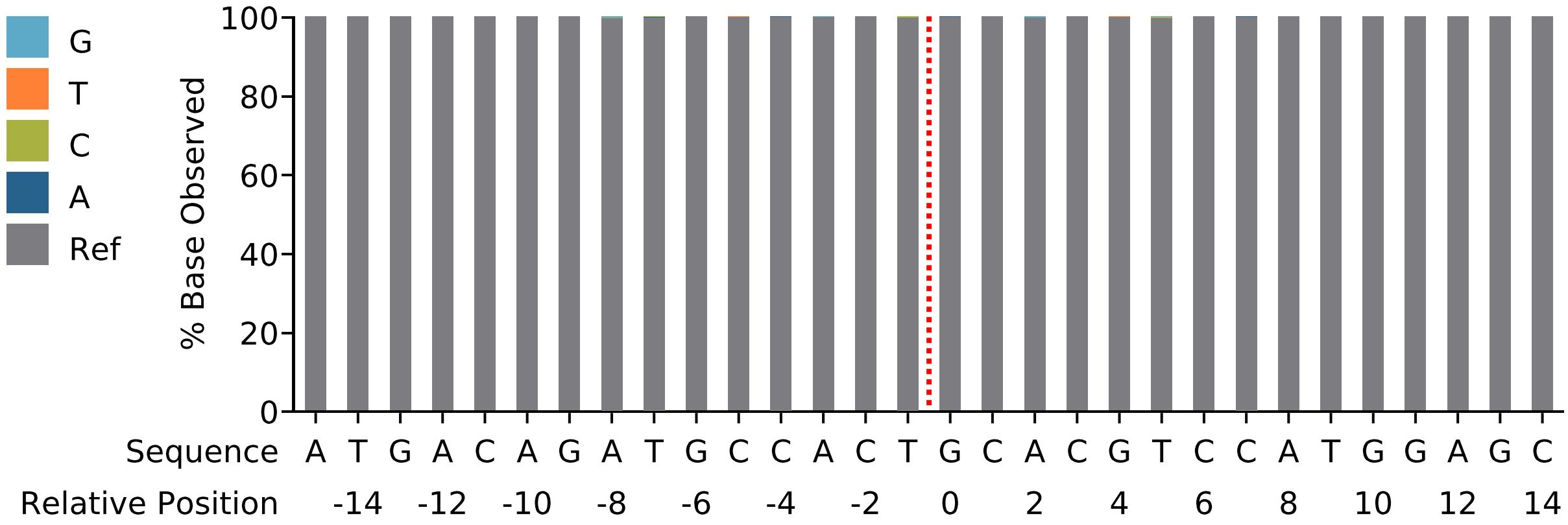

HBV_Cas9_gRNA9__PARP11-AS1

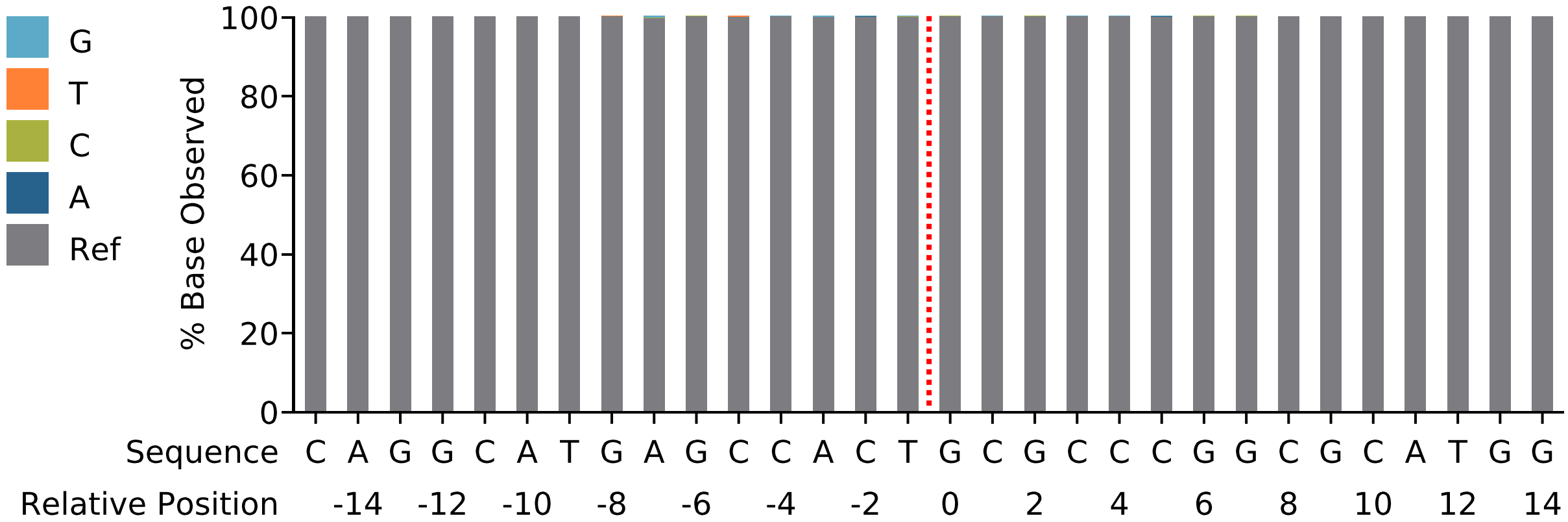

HBV_Cas9_gRNA9__PDE1C

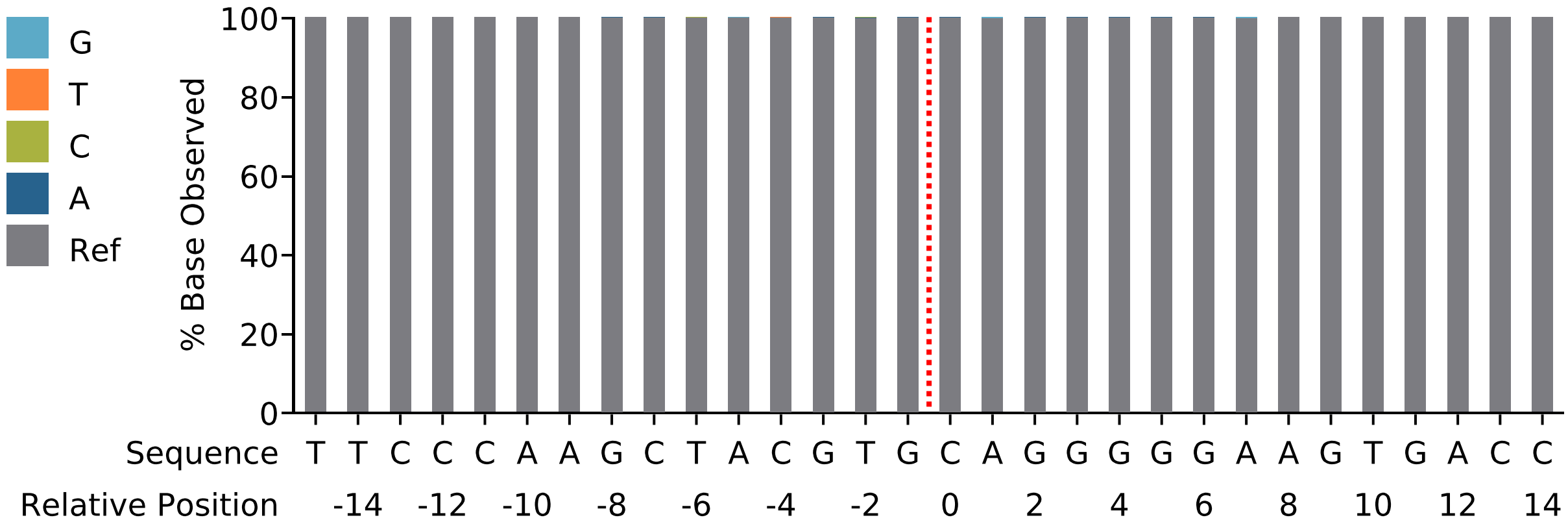

HBV_Cas9_gRNA9__PRRC2B

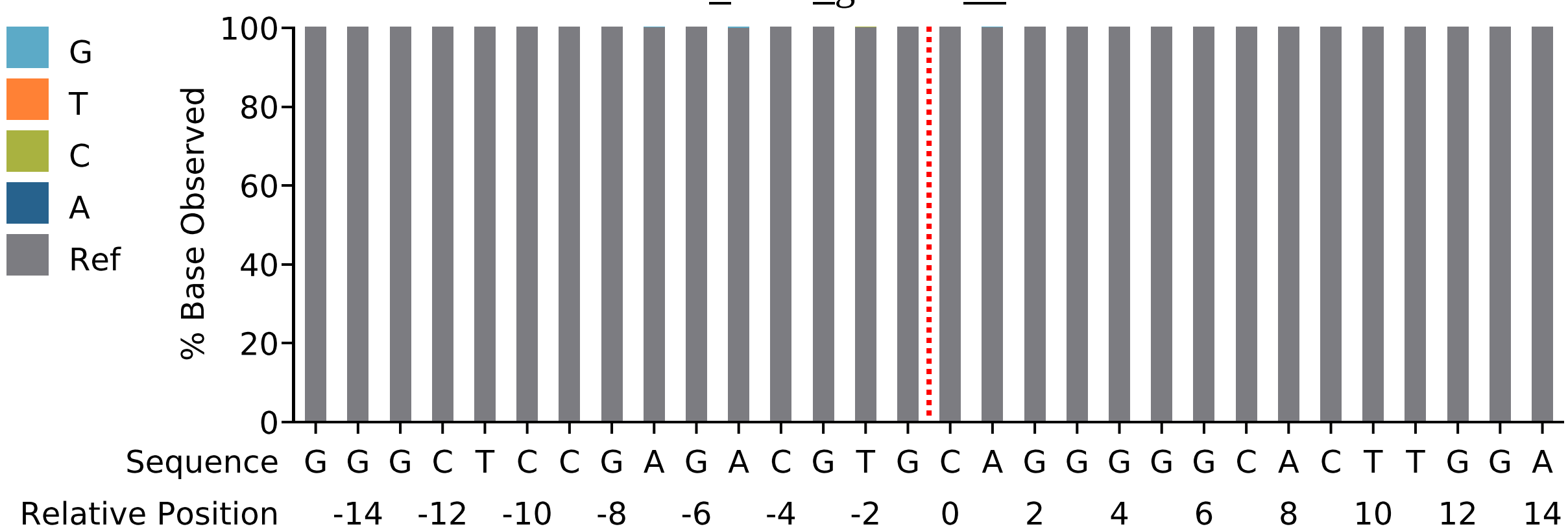

HBV_Cas9_gRNA9__SHISAL1

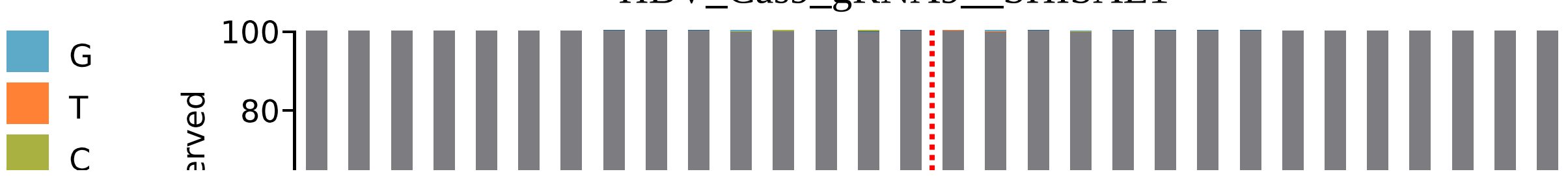

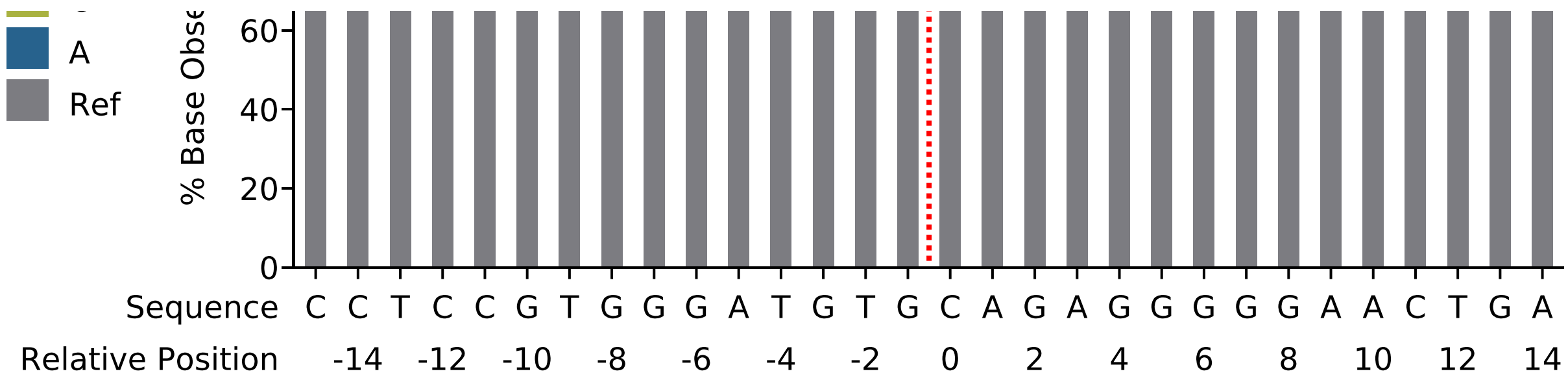

HBV_Cas9_gRNA9__none11

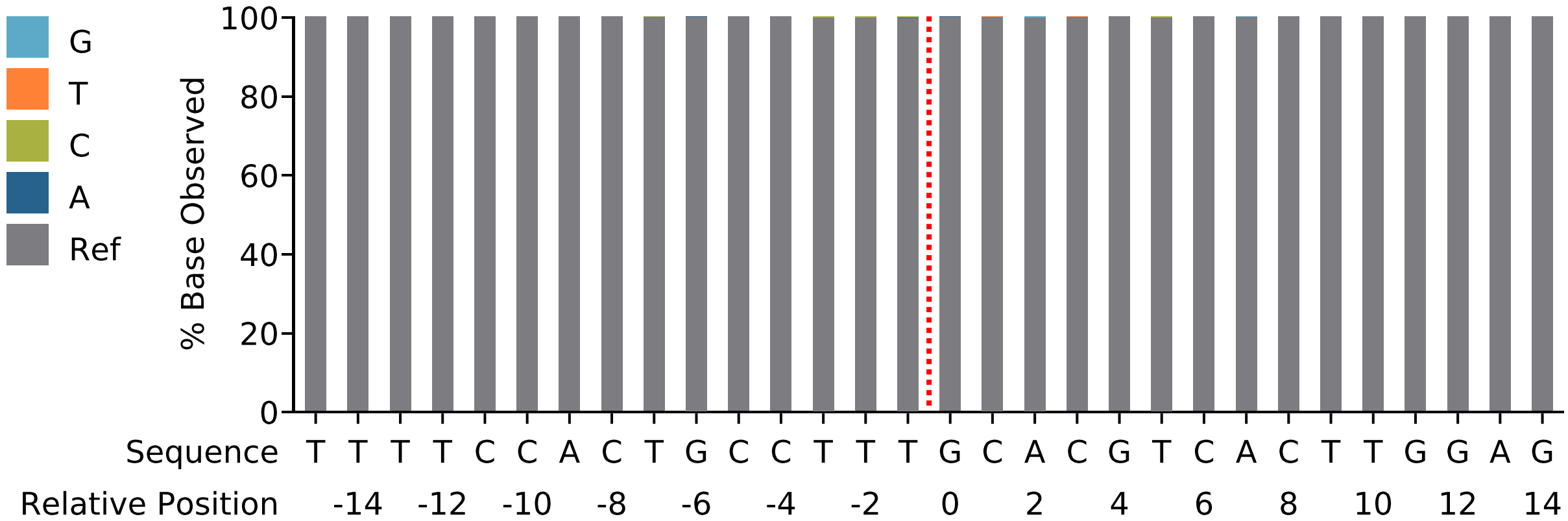

HBV_Cas9_gRNA9__none12

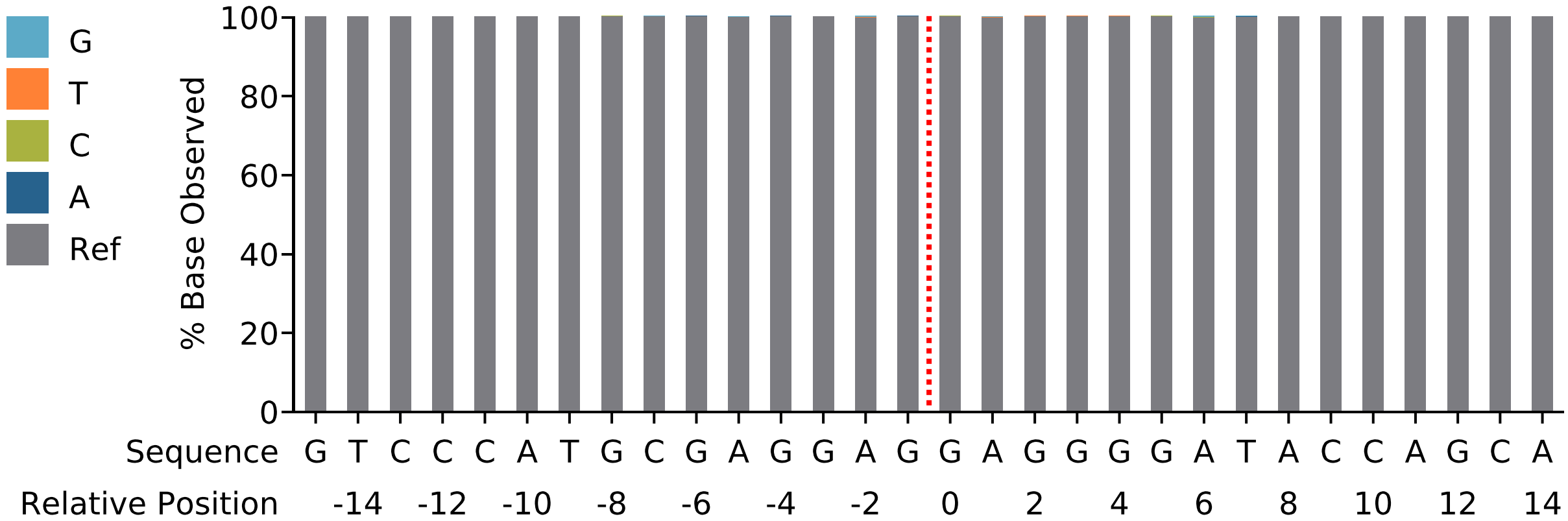

HBV_Cas9_gRNA9__none13

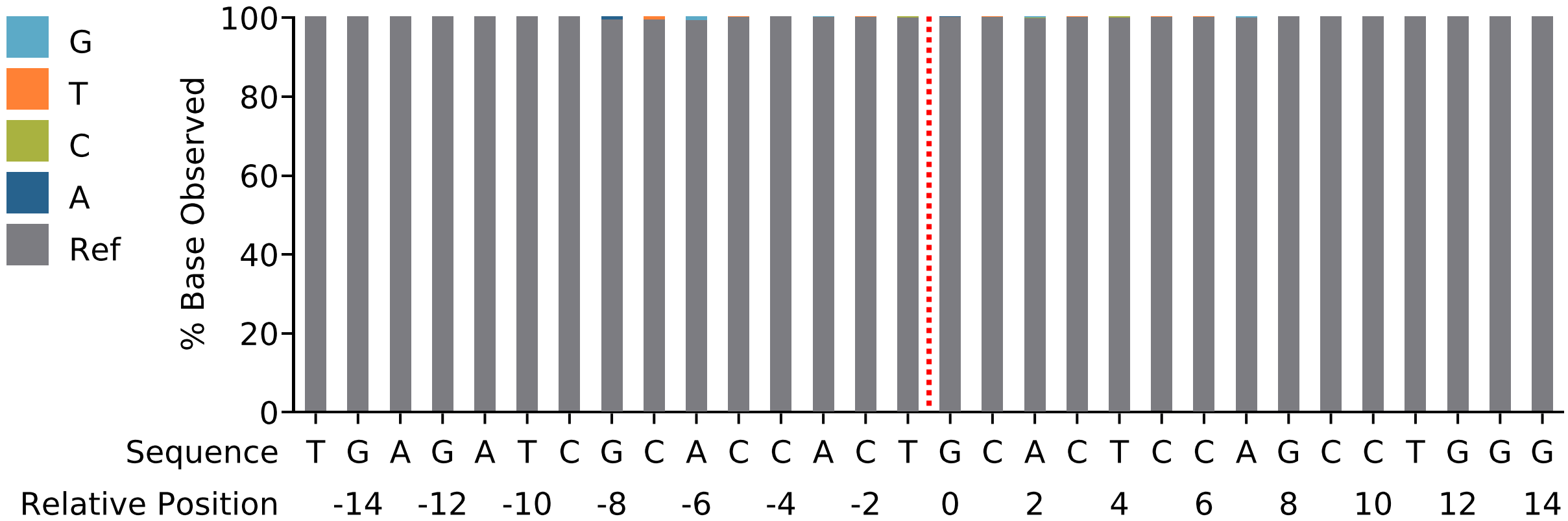

HBV_Cas9_gRNA9__none14

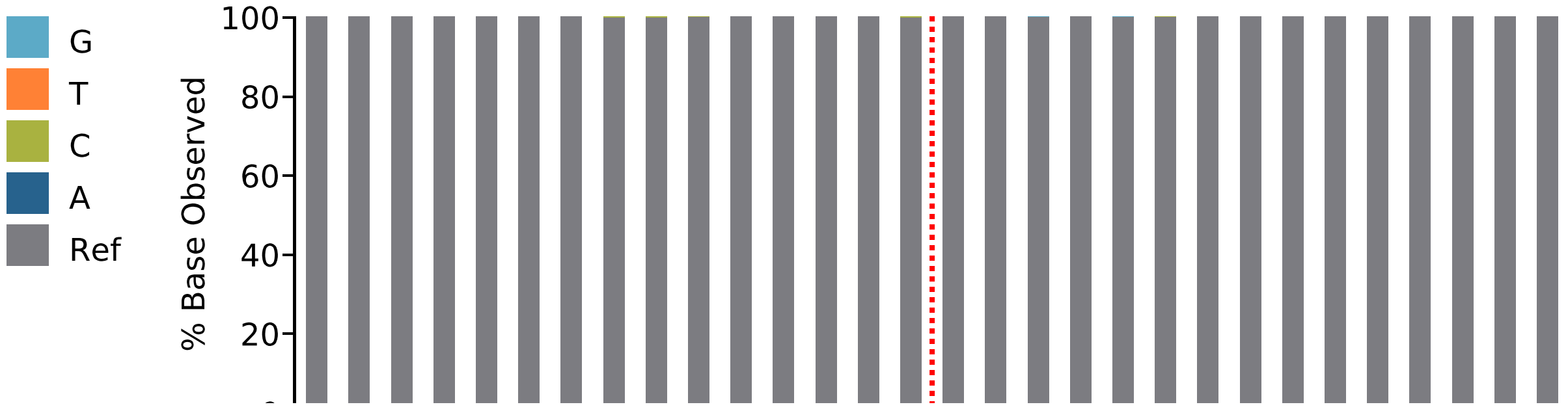

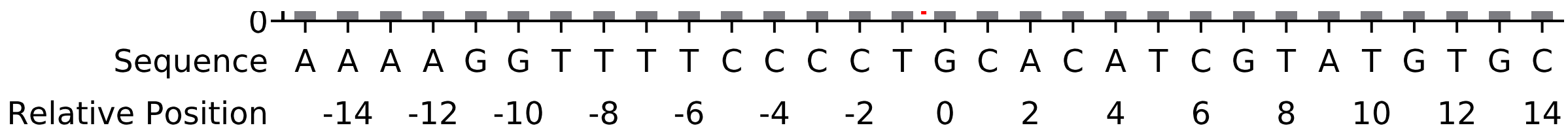

HBV_Cas9_gRNA9__none15

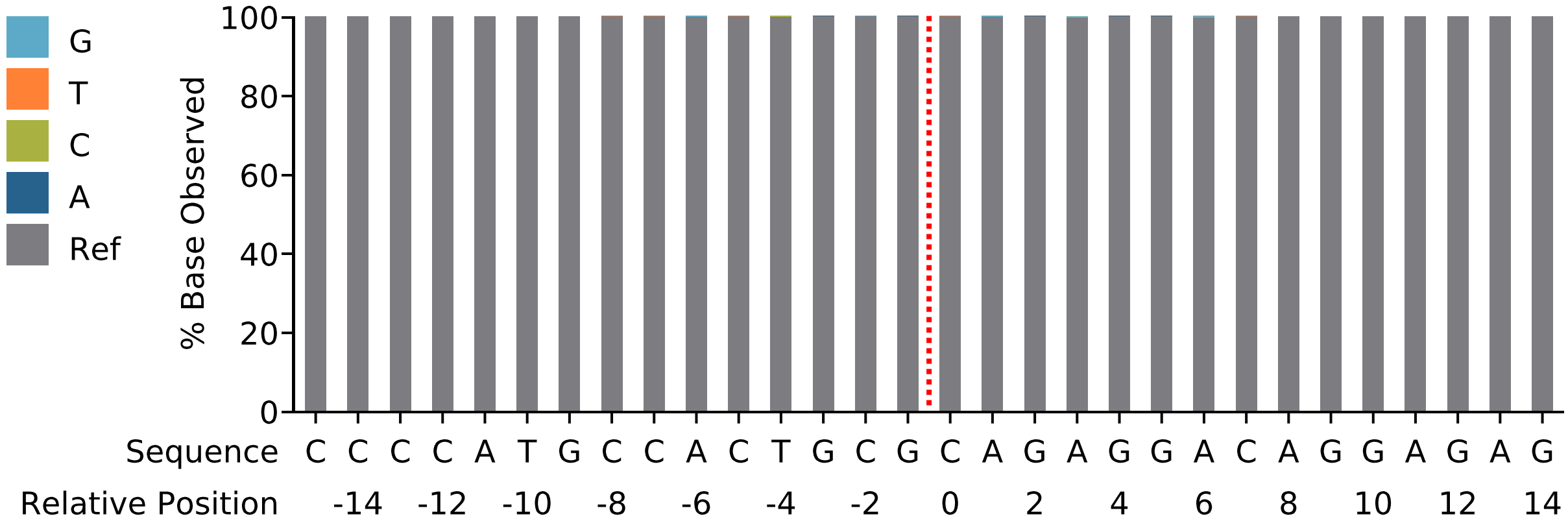

HBV_Cas9_gRNA9__none16

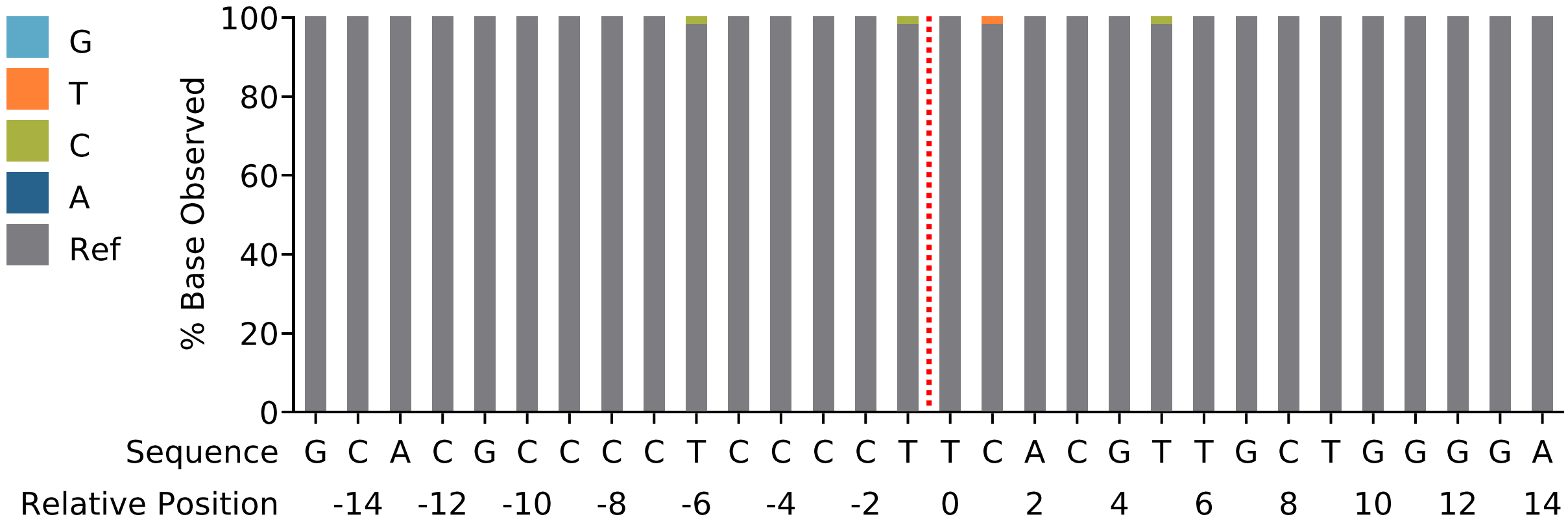

HBV_Cas9_gRNA9__none17

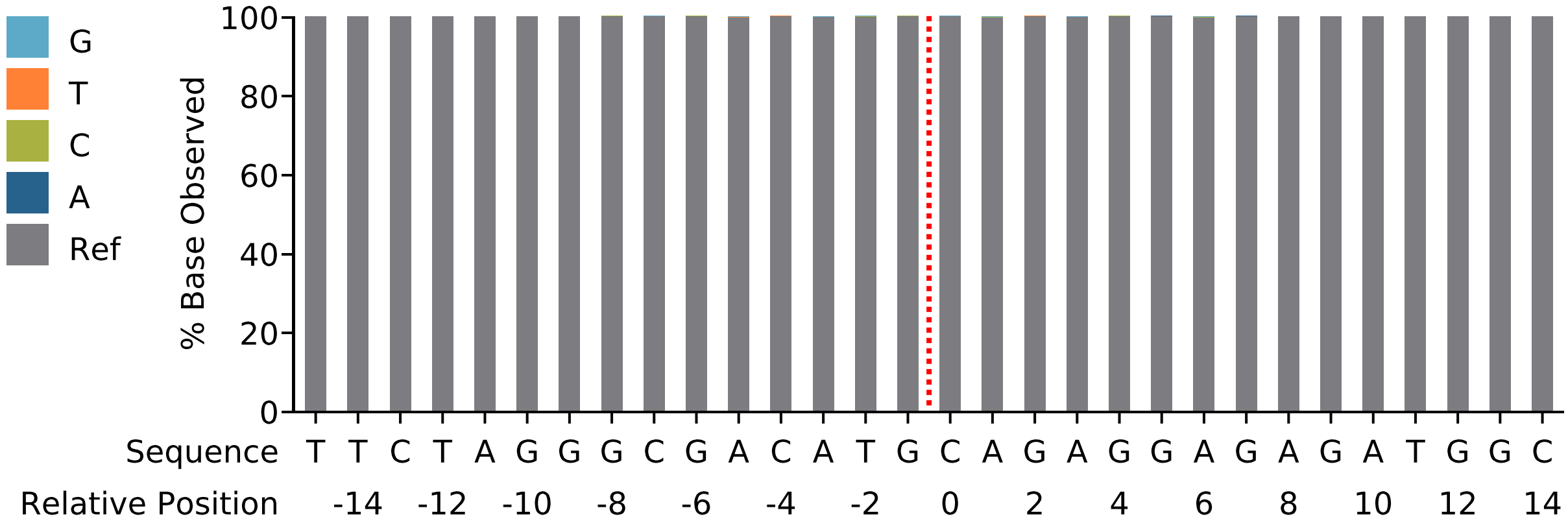

HBV_Cas9_gRNA9__none18

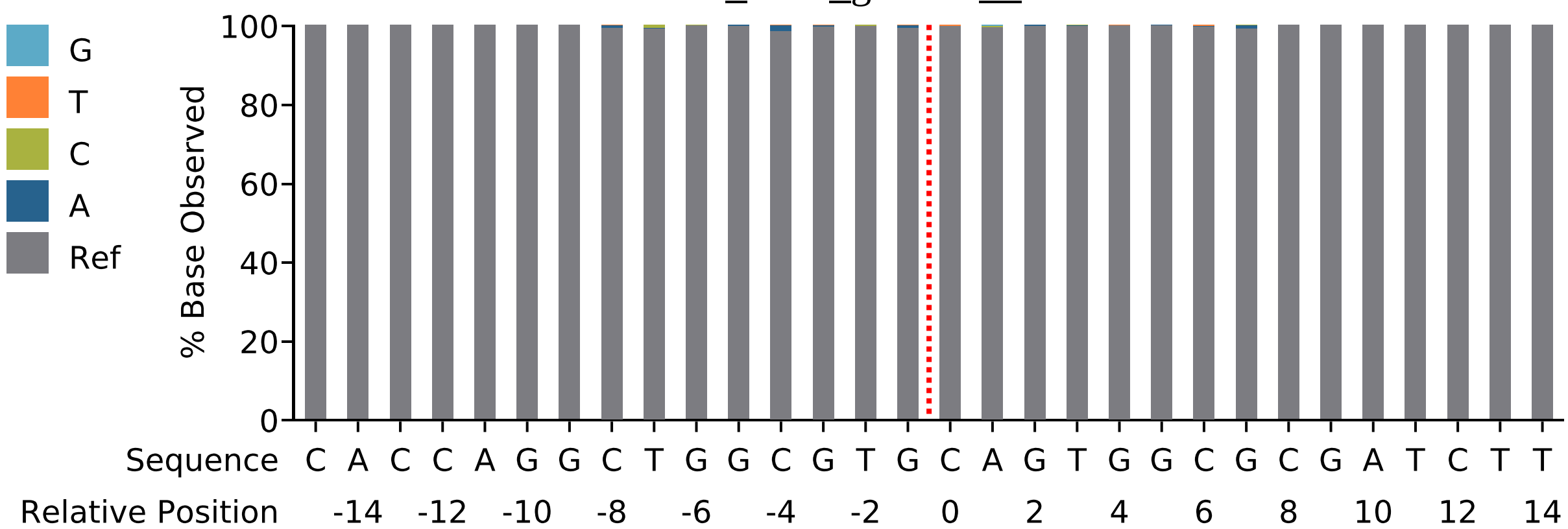

HBV_Cas9_gRNA9__none19

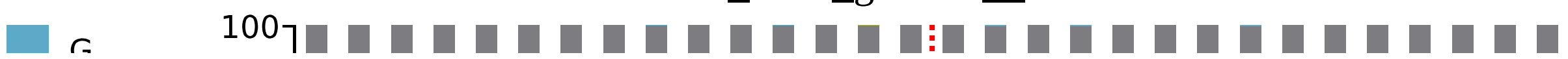

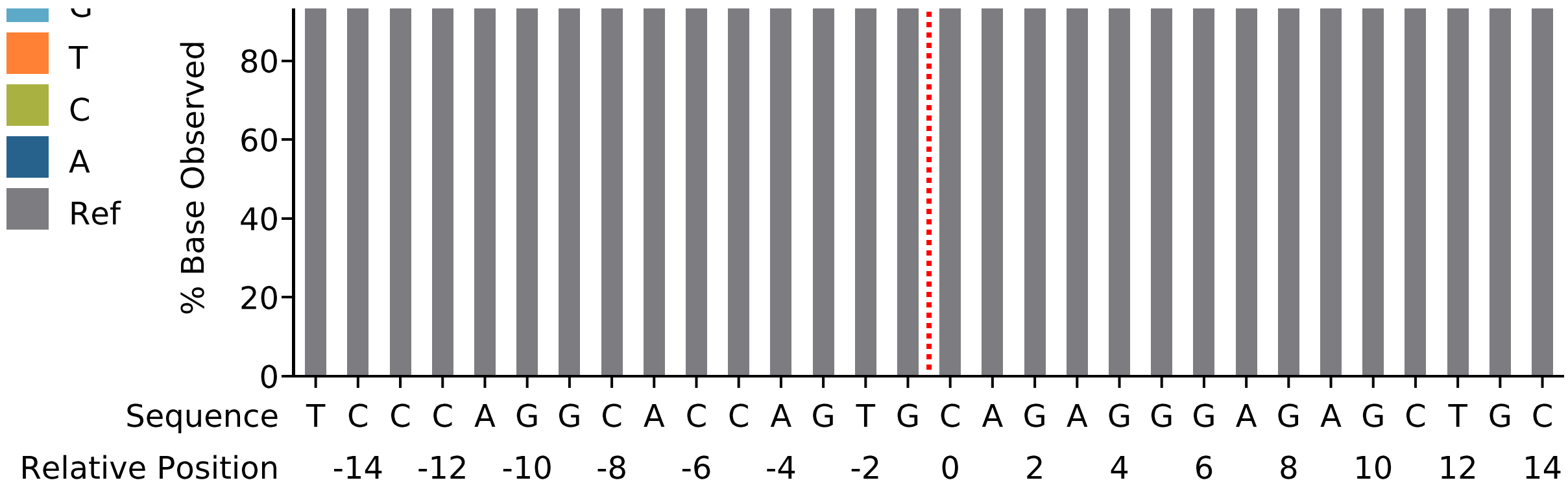

HBV_Cas9_gRNA9__none20

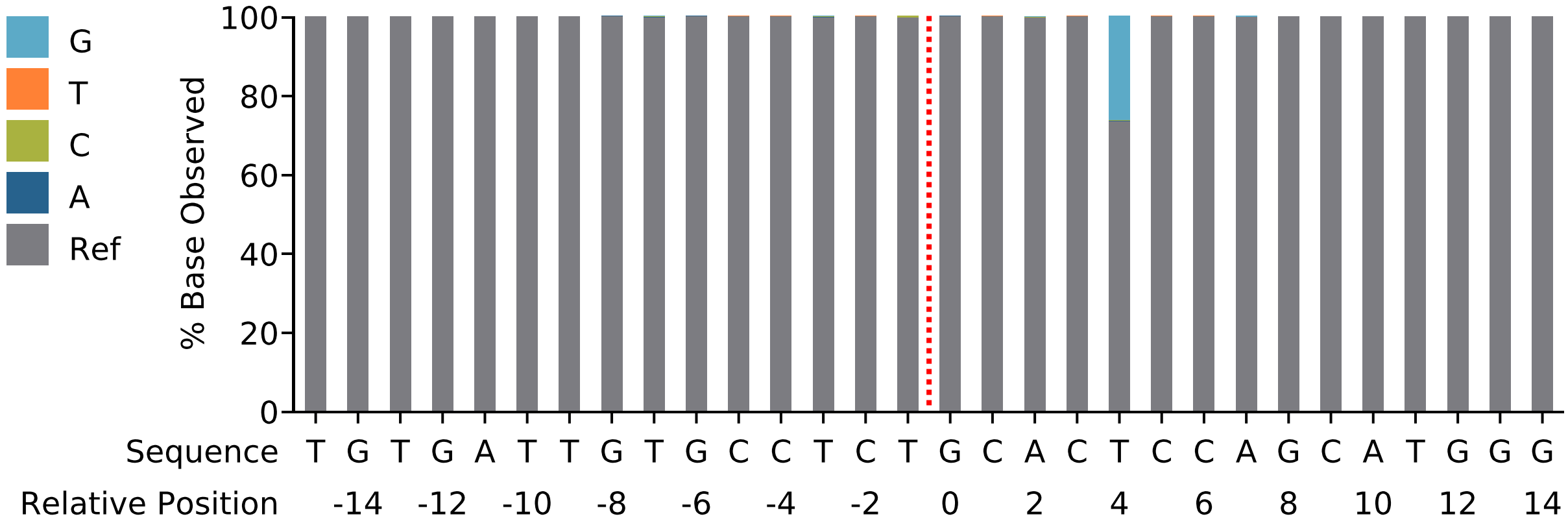

HBV_Cas9_gRNA9__none21

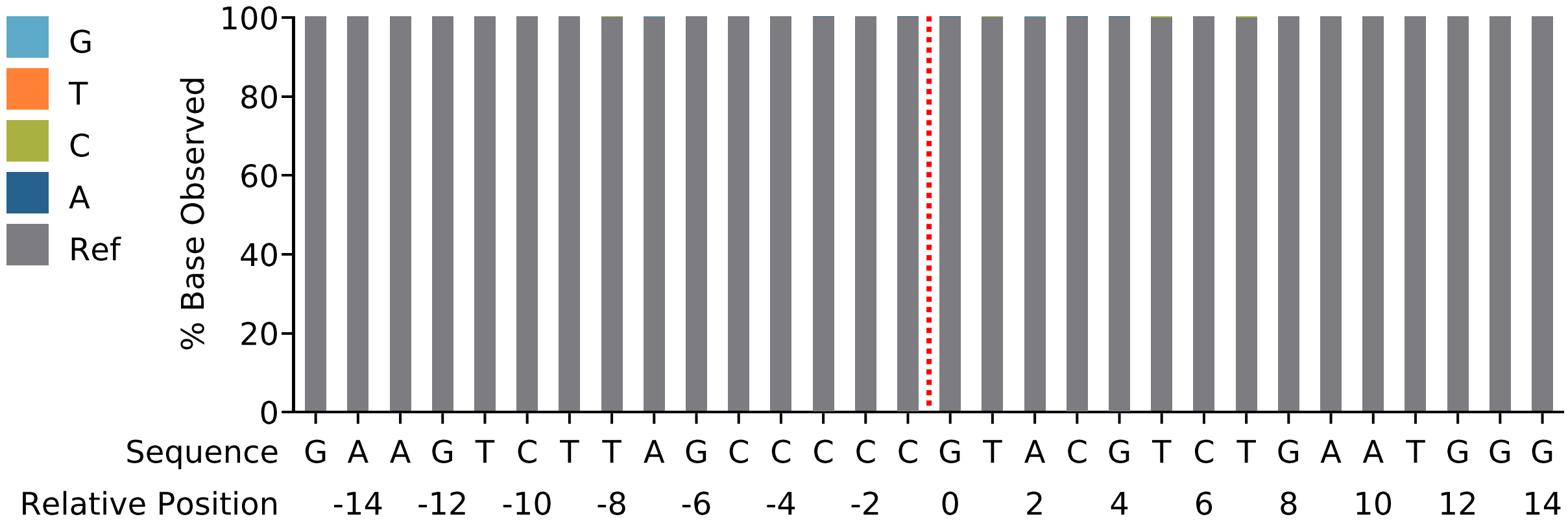

HBV_Cas9_gRNA9__none22

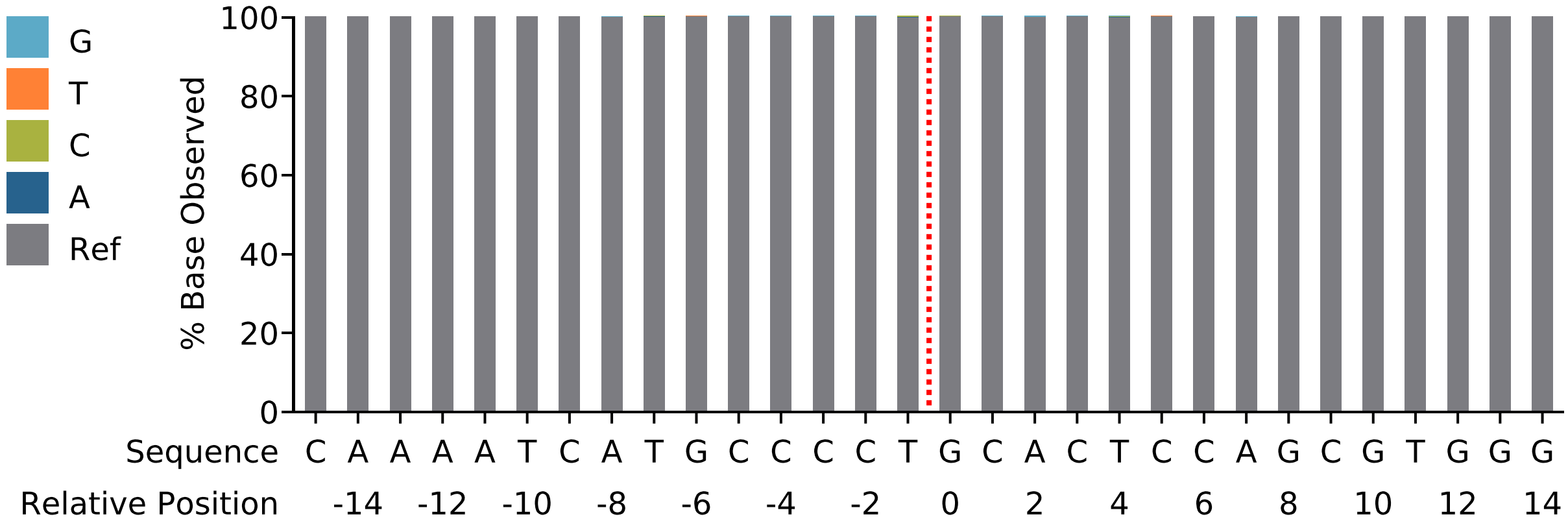

Supplement: Supplementary file 5 [file DataSheet5.pdf]
