# Supplementary material for: Combined RNA- and DNA-targeting CRISPR ribonucleoproteins enhance suppression of HBV transcripts
Source: Front Genome Ed. 2026 Sep 23;8:1824895. doi: 10.3389/fgeed.2026.1824895 (PMC13642003; doi:10.3389/fgeed.2026.1824895)

Analysis mode: CRISPAltRations 1_2_1
Start date: 2026/08/21 15:27
Targets: HBV_Cas12_gRNA4__CD300E, HBV_Cas12_gRNA4__CHKB-DT, HBV_Cas12_gRNA4__GNA12, HBV_Cas12_gRNA4__LINC00989, HBV_Cas12_gRNA4__LOC107986930, HBV_Cas12_gRNA4__MRGBP, HBV_Cas12_gRNA4__PCNX2, HBV_Cas12_gRNA4__SHROOM2, HBV_Cas12_gRNA4__SYNJ2BP, HBV_Cas12_gRNA4__TANK, HBV_Cas12_gRNA4__TCF12, HBV_Cas12_gRNA4__TEKT5, HBV_Cas12_gRNA4__VTI1A, HBV_Cas12_gRNA4__ZBTB44-DT, HBV_Cas12_gRNA4__ZNF292, HBV_Cas12_gRNA4__none52, HBV_Cas12_gRNA4__none54, HBV_Cas12_gRNA4__none55, HBV_Cas12_gRNA4__none56, HBV_Cas12a_Control__CNTN451, HBV_Cas12a_Control__POGLUT3, HBV_Cas12a_Control__ROR1, HBV_Cas12a_Control__SHISA6, HBV_Cas12a_Control__TMC4, HBV_Cas12a_Control__none44, HBV_Cas12a_Control__none46, HBV_Cas12a_Control__none47, HBV_Cas12a_Control__none48, HBV_Cas12a_Control__none49

Metrics

	Group ID	Treated	Total Reads	Percent Edited	Percent Unedited	Percent NHEJ	Percent Perfect HDR	Percent Imperfect HDR	Percent HDR	Percent Other	Percent In Frame	Percent Frameshift	Percent Indels	Percent Insertions	Percent Templated Insertion	Percent GC-Insertion	Percent Deletions
**HBV_Cas12_gRNA4__CD300E**	HBV_Fig4	FALSE	494	99.19	0.81	0.0	0.0	0.0	0.0	99.19	100.0	0.0	0.0	0.0	0.0	0.0	0.0
**HBV_Cas12_gRNA4__CHKB-DT**	HBV_Fig4	FALSE	8047	2.73	97.27	0.0	0.0	0.0	0.0	2.73	100.0	0.0	0.0	0.0	0.0	0.0	0.0
**HBV_Cas12_gRNA4__GNA12**	HBV_Fig4	FALSE	38853	3.3	96.7	0.1	0.0	0.0	0.0	3.2	99.9	0.1	0.1	0.01	100.0	0.0	0.09
**HBV_Cas12_gRNA4__LINC00989**	HBV_Fig4	FALSE	66223	3.29	96.71	0.08	0.0	0.0	0.0	3.21	99.92	0.08	0.08	0.0	0.0	0.0	0.08
**HBV_Cas12_gRNA4__LOC107986930**	HBV_Fig4	FALSE	39061	4.16	95.84	0.14	0.0	0.0	0.0	4.02	99.86	0.14	0.14	0.0	0.0	0.0	0.14
**HBV_Cas12_gRNA4__MRGBP**	HBV_Fig4	FALSE	19041	44.88	55.12	0.1	0.0	0.0	0.0	44.78	99.94	0.06	0.1	0.0	0.0	0.0	0.1
**HBV_Cas12_gRNA4__PCNX2**	HBV_Fig4	FALSE	176363	3.53	96.47	0.06	0.0	0.0	0.0	3.47	99.94	0.06	0.06	0.0	0.0	0.0	0.06
**HBV_Cas12_gRNA4__SHROOM2**	HBV_Fig4	FALSE	39027	3.67	96.33	0.38	0.0	0.0	0.0	3.29	99.62	0.38	0.38	0.07	88.89	0.0	0.31
**HBV_Cas12_gRNA4__SYNJ2BP**	HBV_Fig4	FALSE	52216	3.58	96.42	0.09	0.0	0.0	0.0	3.49	99.91	0.09	0.09	0.0	100.0	0.0	0.09
**HBV_Cas12_gRNA4__TANK**	HBV_Fig4	FALSE	86334	3.4	96.6	0.14	0.0	0.0	0.0	3.26	99.86	0.14	0.14	0.0	0.0	0.0	0.14
**HBV_Cas12_gRNA4__TCF12**	HBV_Fig4	FALSE	22474	99.99	0.01	99.87	0.0	0.0	0.0	0.12	99.96	0.04	99.87	99.87	0.0	0.23	0.01
**HBV_Cas12_gRNA4__TEKT5**	HBV_Fig4	FALSE	1968313	4.11	95.89	0.08	0.0	0.0	0.0	4.03	99.93	0.07	0.08	0.0	0.0	7.69	0.08
**HBV_Cas12_gRNA4__VTI1A**	HBV_Fig4	FALSE	28095	3.73	96.27	0.02	0.0	0.0	0.0	3.71	99.98	0.02	0.02	0.0	0.0	0.0	0.02
**HBV_Cas12_gRNA4__ZBTB44-DT**	HBV_Fig4	FALSE	4169	2.85	97.15	0.07	0.0	0.0	0.0	2.78	99.93	0.07	0.07	0.0	0.0	0.0	0.07
**HBV_Cas12_gRNA4__ZNF292**	HBV_Fig4	FALSE	51579	4.1	95.9	0.07	0.0	0.0	0.0	4.03	99.93	0.07	0.07	0.0	0.0	0.0	0.07
**HBV_Cas12_gRNA4__none52**	HBV_Fig4	FALSE	72949	3.74	96.26	0.02	0.0	0.0	0.0	3.72	99.98	0.02	0.02	0.0	0.0	0.0	0.02
**HBV_Cas12_gRNA4__none54**	HBV_Fig4	FALSE	5762	3.84	96.16	0.02	0.0	0.0	0.0	3.82	99.98	0.02	0.02	0.0	0.0	0.0	0.02
**HBV_Cas12_gRNA4__none55**	HBV_Fig4	FALSE	51346	99.91	0.09	0.1	0.0	0.0	0.0	99.81	99.9	0.1	0.1	0.0	0.0	0.0	0.1
**HBV_Cas12_gRNA4__none56**	HBV_Fig4	FALSE	65473	3.61	96.39	0.07	0.0	0.0	0.0	3.54	99.93	0.07	0.07	0.0	0.0	0.0	0.07
**HBV_Cas12a_Control__CNTN451**	HBV_Fig4	FALSE	58351	3.89	96.11	0.04	0.0	0.0	0.0	3.85	99.96	0.04	0.04	0.0	0.0	0.0	0.03
**HBV_Cas12a_Control__POGLUT3**	HBV_Fig4	FALSE	156919	36.72	63.28	0.27	0.0	0.0	0.0	36.45	99.73	0.27	0.27	0.01	100.0	0.0	0.26
**HBV_Cas12a_Control__ROR1**	HBV_Fig4	FALSE	8812	8.84	91.16	3.12	0.0	0.0	0.0	5.72	97.27	2.73	3.12	0.89	0.0	0.0	2.24
**HBV_Cas12a_Control__SHISA6**	HBV_Fig4	FALSE	74323	3.16	96.84	0.21	0.0	0.0	0.0	2.95	99.79	0.21	0.21	0.01	33.33	16.67	0.2
**HBV_Cas12a_Control__TMC4**	HBV_Fig4	FALSE	8510	100.0	0.0	100.0	0.0	0.0	0.0	0.0	96.92	3.08	100.0	99.96	0.0	0.0	0.06
**HBV_Cas12a_Control__none44**	HBV_Fig4	FALSE	514	94.16	5.84	85.02	0.0	0.0	0.0	9.14	36.19	63.81	85.02	20.43	0.0	28.57	64.59
**HBV_Cas12a_Control__none46**	HBV_Fig4	FALSE	1608076	54.58	45.42	0.07	0.0	0.0	0.0	54.51	99.93	0.07	0.07	0.01	90.14	1.41	0.07
**HBV_Cas12a_Control__none47**	HBV_Fig4	FALSE	25398	10.22	89.78	0.16	0.0	0.0	0.0	10.06	99.96	0.04	0.16	0.01	0.0	0.0	0.15
**HBV_Cas12a_Control__none48**	HBV_Fig4	FALSE	573675	3.95	96.05	0.09	0.0	0.0	0.0	3.86	99.91	0.09	0.09	0.0	0.0	0.0	0.09
**HBV_Cas12a_Control__none49**	HBV_Fig4	FALSE	249200	100.0	0.0	97.78	0.0	0.0	0.0	2.22	18.92	81.08	97.78	46.97	0.0	20.21	50.81

Percentages

% Edited *% Repair Pathways* *% Frameshift*

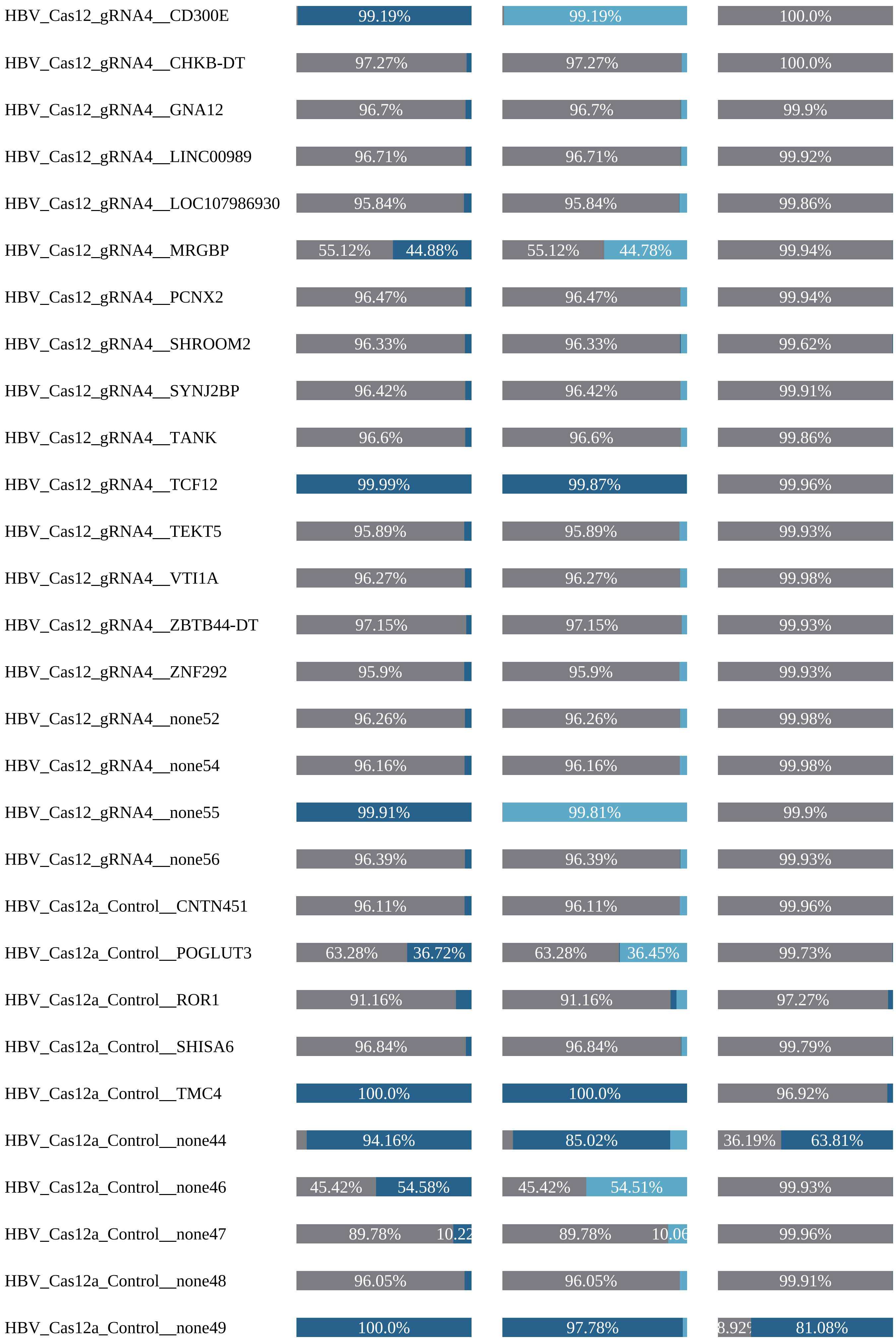

HBV_Cas12_gRNA4__CD300E 99.19% 99.19% 100.0%
HBV_Cas12_gRNA4__CHKB-DT 97.27% 97.27% 100.0%
HBV_Cas12_gRNA4__GNA12 96.7% 96.7% 99.9%
HBV_Cas12_gRNA4__LINC00989 96.71% 96.71% 99.92%
HBV_Cas12_gRNA4__LOC107986930 95.84% 95.84% 99.86%
HBV_Cas12_gRNA4__MRGBP 55.12% 44.88% 55.12% 44.78% 99.94%
HBV_Cas12_gRNA4__PCNX2 96.47% 96.47% 99.94%
HBV_Cas12_gRNA4__SHROOM2 96.33% 96.33% 99.62%
HBV_Cas12_gRNA4__SYNJ2BP 96.42% 96.42% 99.91%
HBV_Cas12_gRNA4__TANK 96.6% 96.6% 99.86%
HBV_Cas12_gRNA4__TCF12 99.99% 99.87% 99.96%
HBV_Cas12_gRNA4__TEKT5 95.89% 95.89% 99.93%
HBV_Cas12_gRNA4__VTI1A 96.27% 96.27% 99.98%
HBV_Cas12_gRNA4__ZBTB44-DT 97.15% 97.15% 99.93%
HBV_Cas12_gRNA4__ZNF292 95.9% 95.9% 99.93%
HBV_Cas12_gRNA4__none52 96.26% 96.26% 99.98%
HBV_Cas12_gRNA4__none54 96.16% 96.16% 99.98%
HBV_Cas12_gRNA4__none55 99.91% 99.81% 99.9%
HBV_Cas12_gRNA4__none56 96.39% 96.39% 99.93%
HBV_Cas12a_Control__CNTN451 96.11% 96.11% 99.96%
HBV_Cas12a_Control__POGLUT3 63.28% 36.72% 63.28% 36.45% 99.73%
HBV_Cas12a_Control__ROR1 91.16% 91.16% 97.27%
HBV_Cas12a_Control__SHISA6 96.84% 96.84% 99.79%
HBV_Cas12a_Control__TMC4 100.0% 100.0% 96.92%
HBV_Cas12a_Control__none44 94.16% 85.02% 36.19% 63.81%
HBV_Cas12a_Control__none46 45.42% 54.58% 45.42% 54.51% 99.93%
HBV_Cas12a_Control__none47 89.78% 10.22 89.78% 10.06 99.96%
HBV_Cas12a_Control__none48 96.05% 96.05% 99.91%
HBV_Cas12a_Control__none49 100.0% 97.78% 8.92 81.08%

Unedited Edited

Unedited NHEJ PerfectHDR ImperfectHDR Other

Inframe Frameshift

Insertions

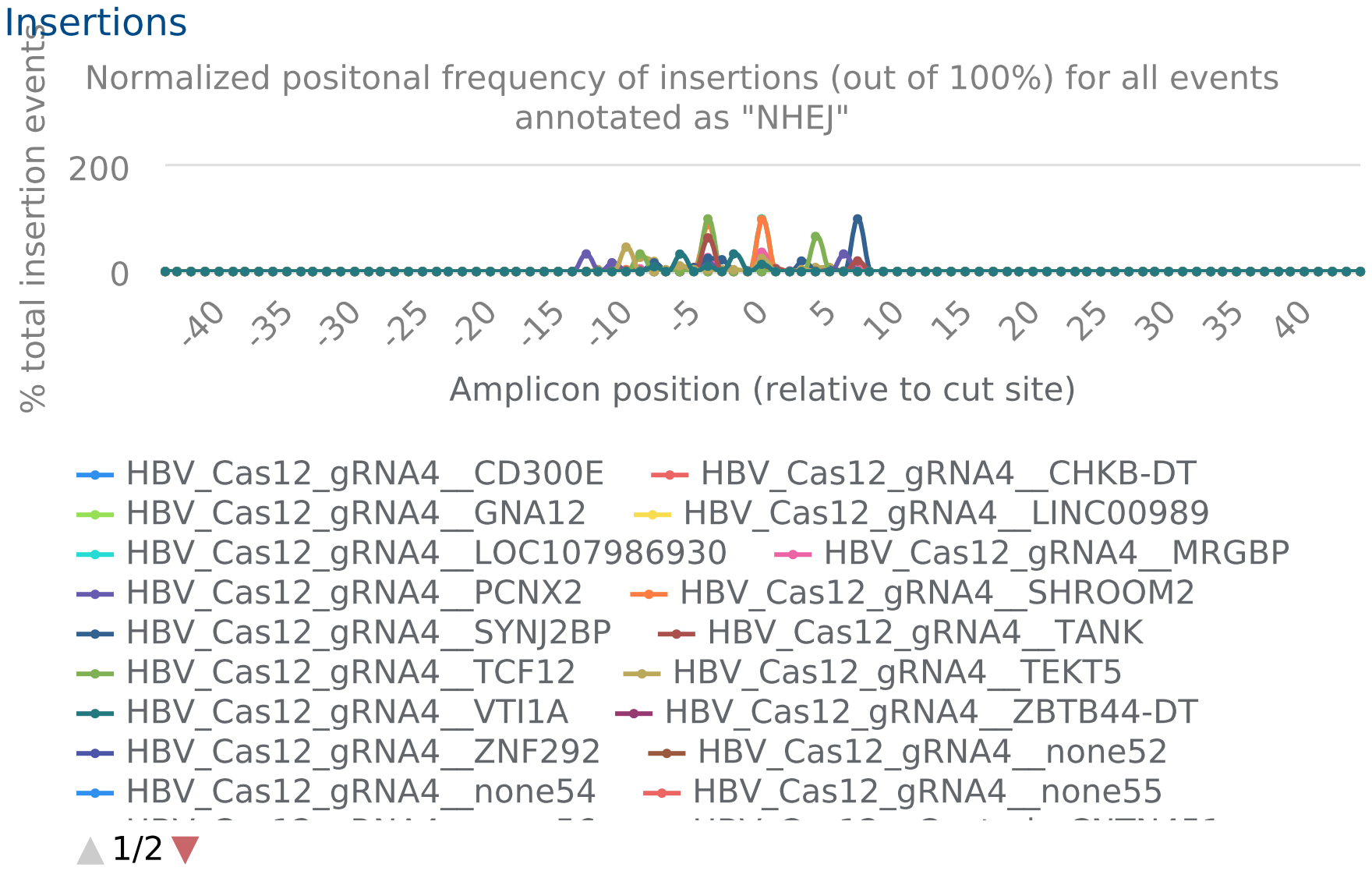

Deletions

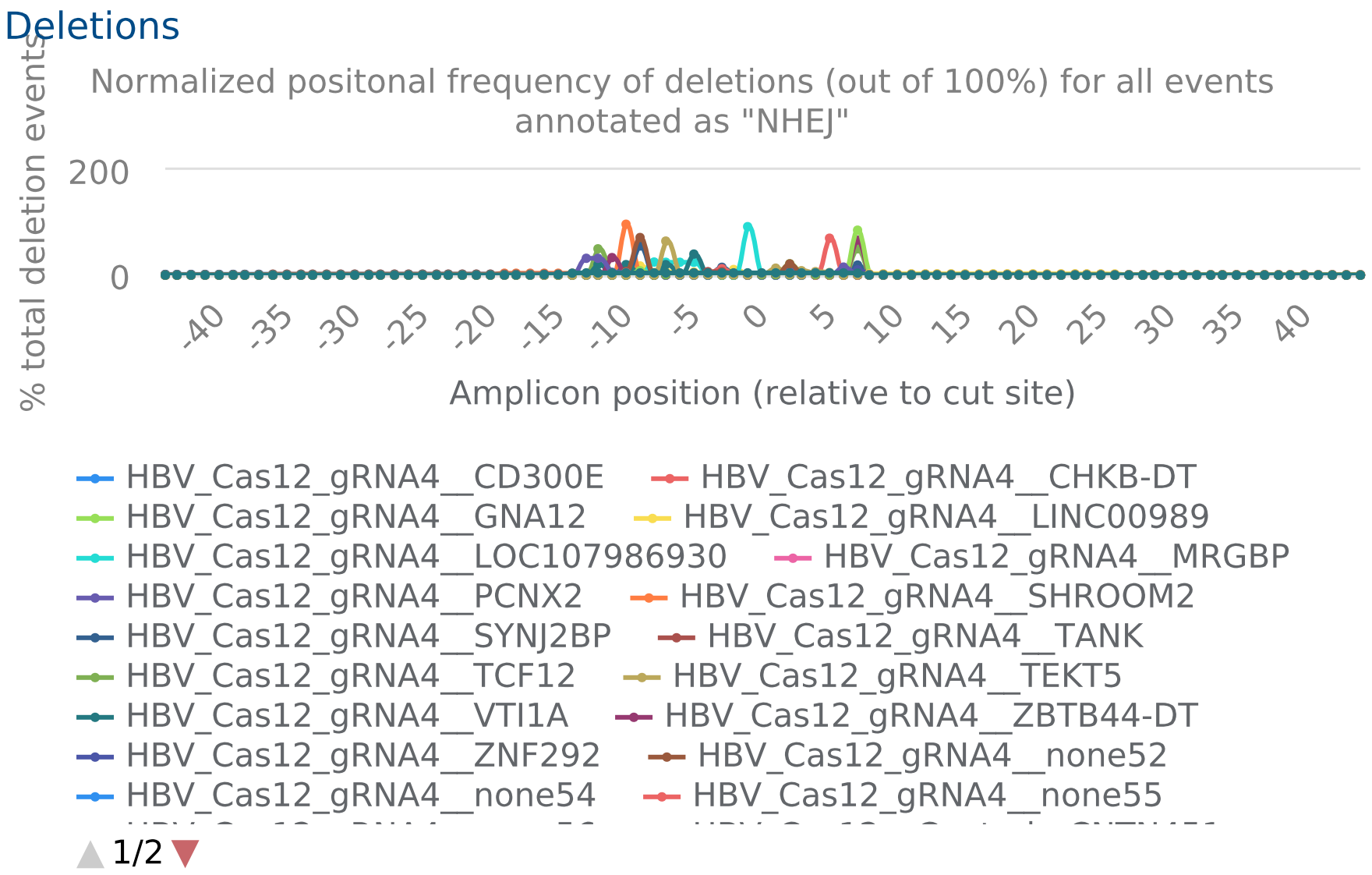

Indel Frequencies

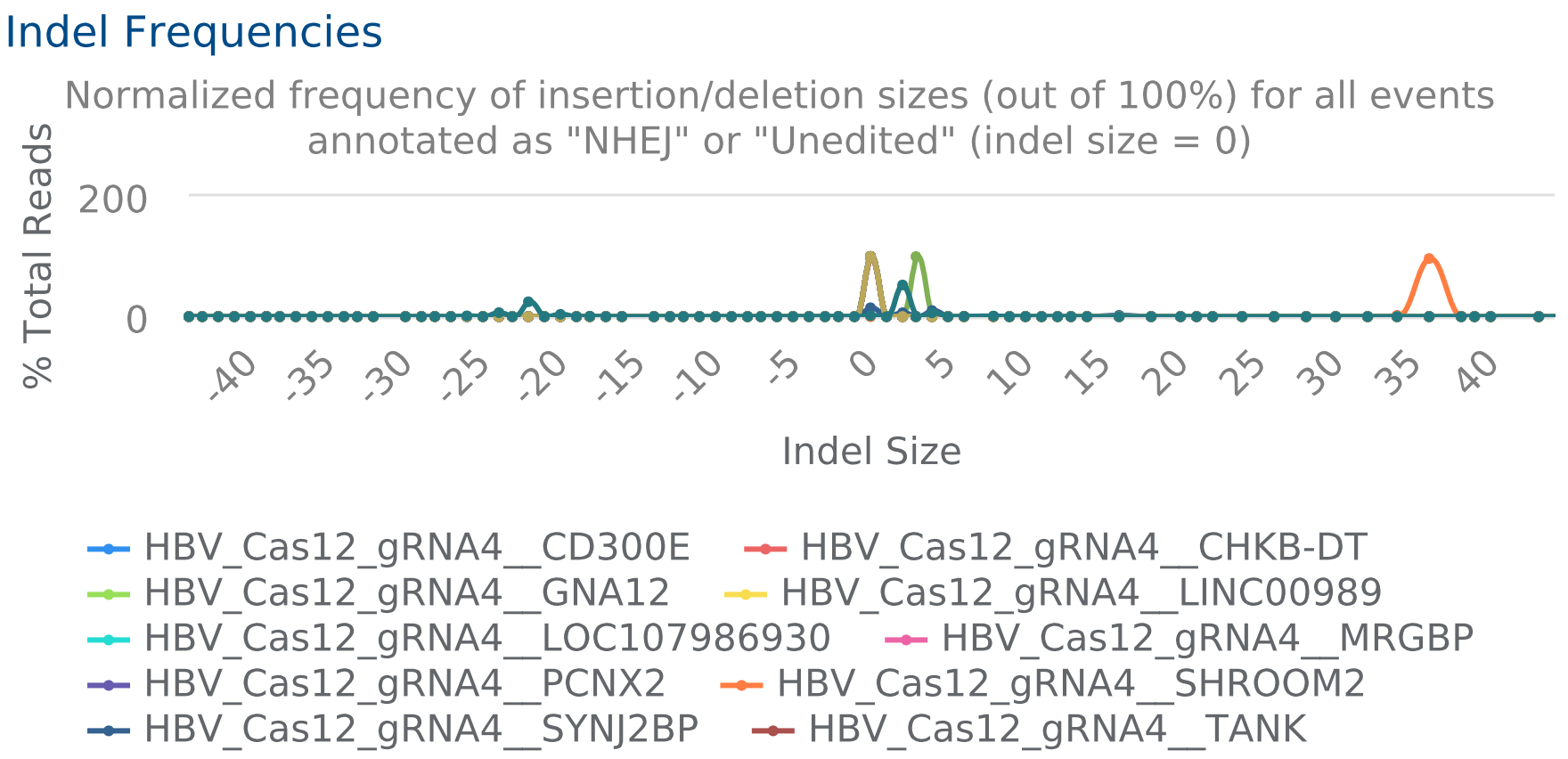

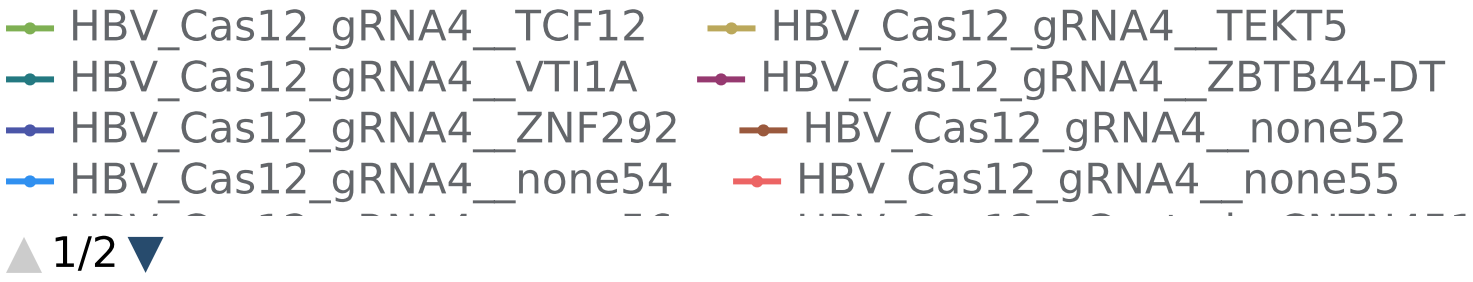

SNP Graph

HBV_Cas12_gRNA4__CD300E

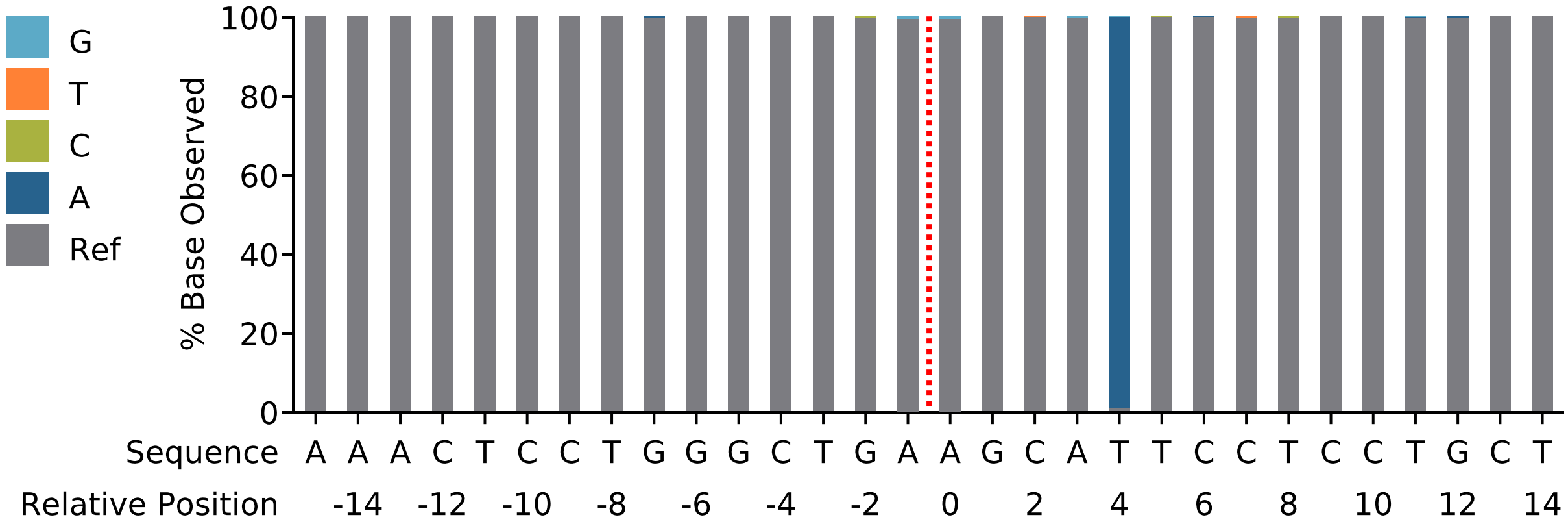

HBV_Cas12_gRNA4__CHKB-DT

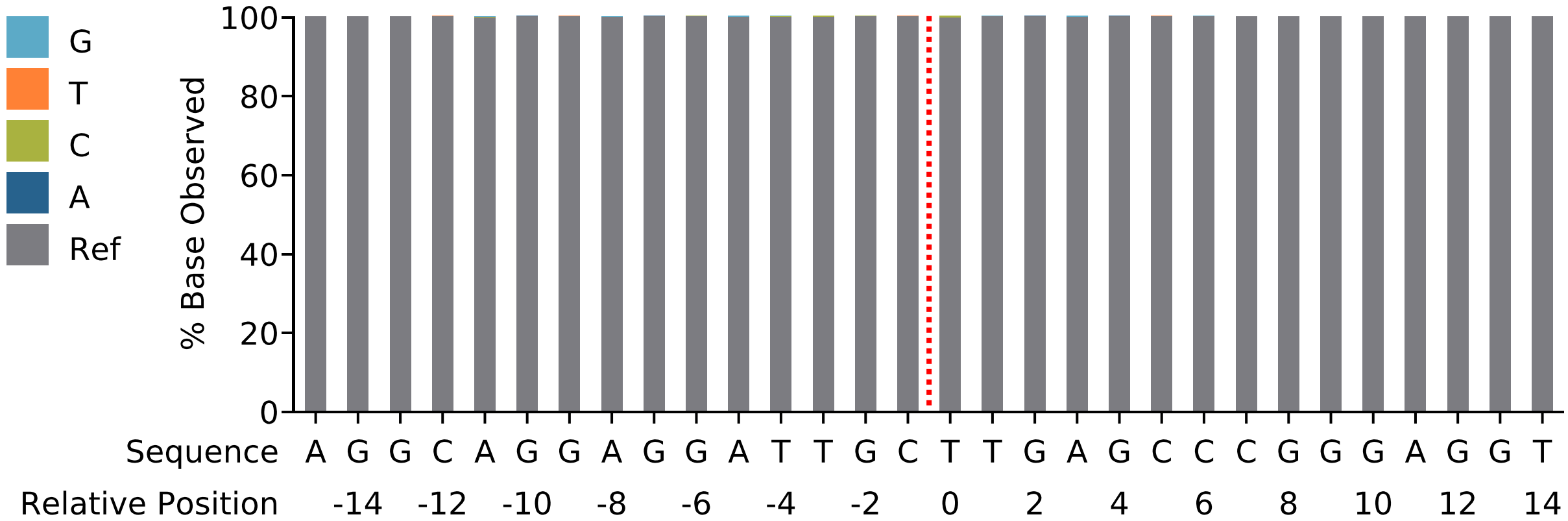

HBV_Cas12_gRNA4__GNA12

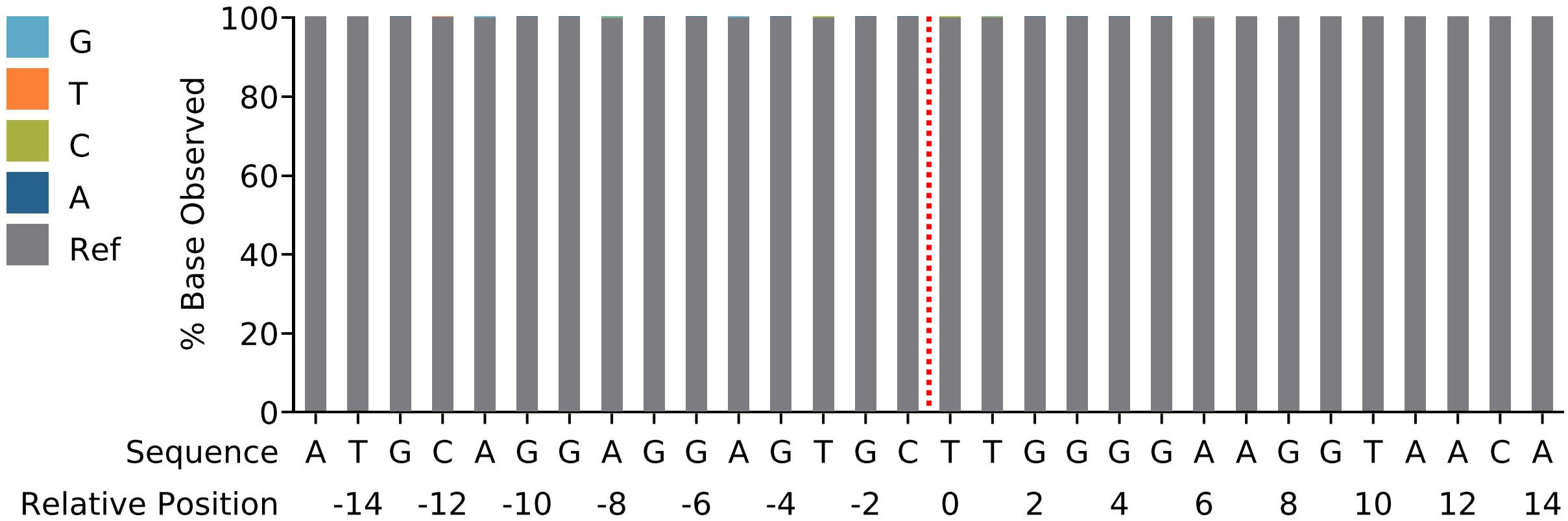

HBV_Cas12_gRNA4__LINC00989

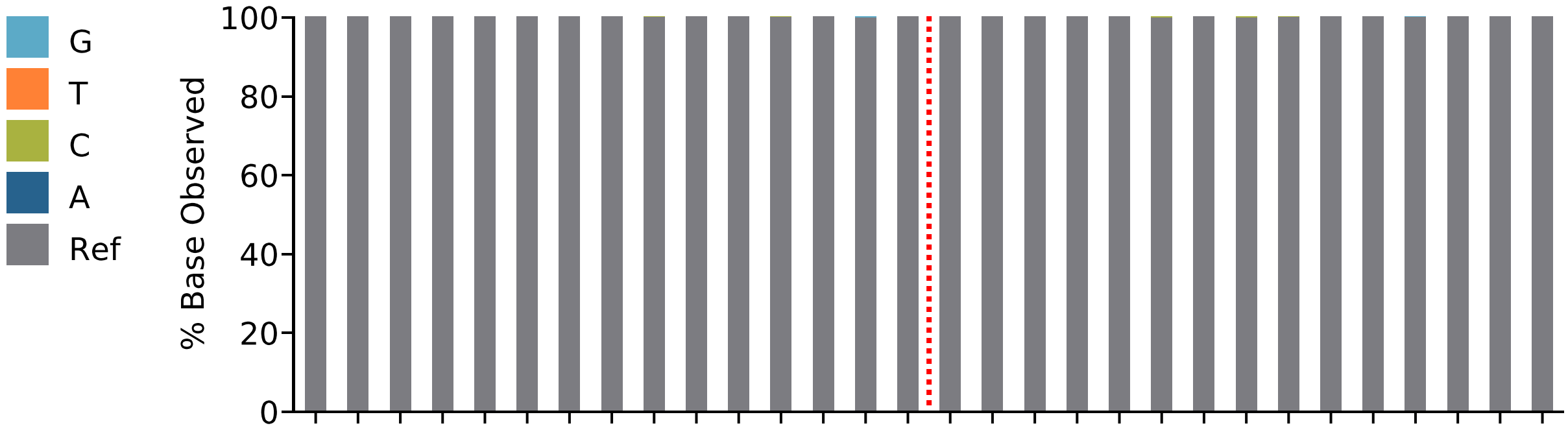

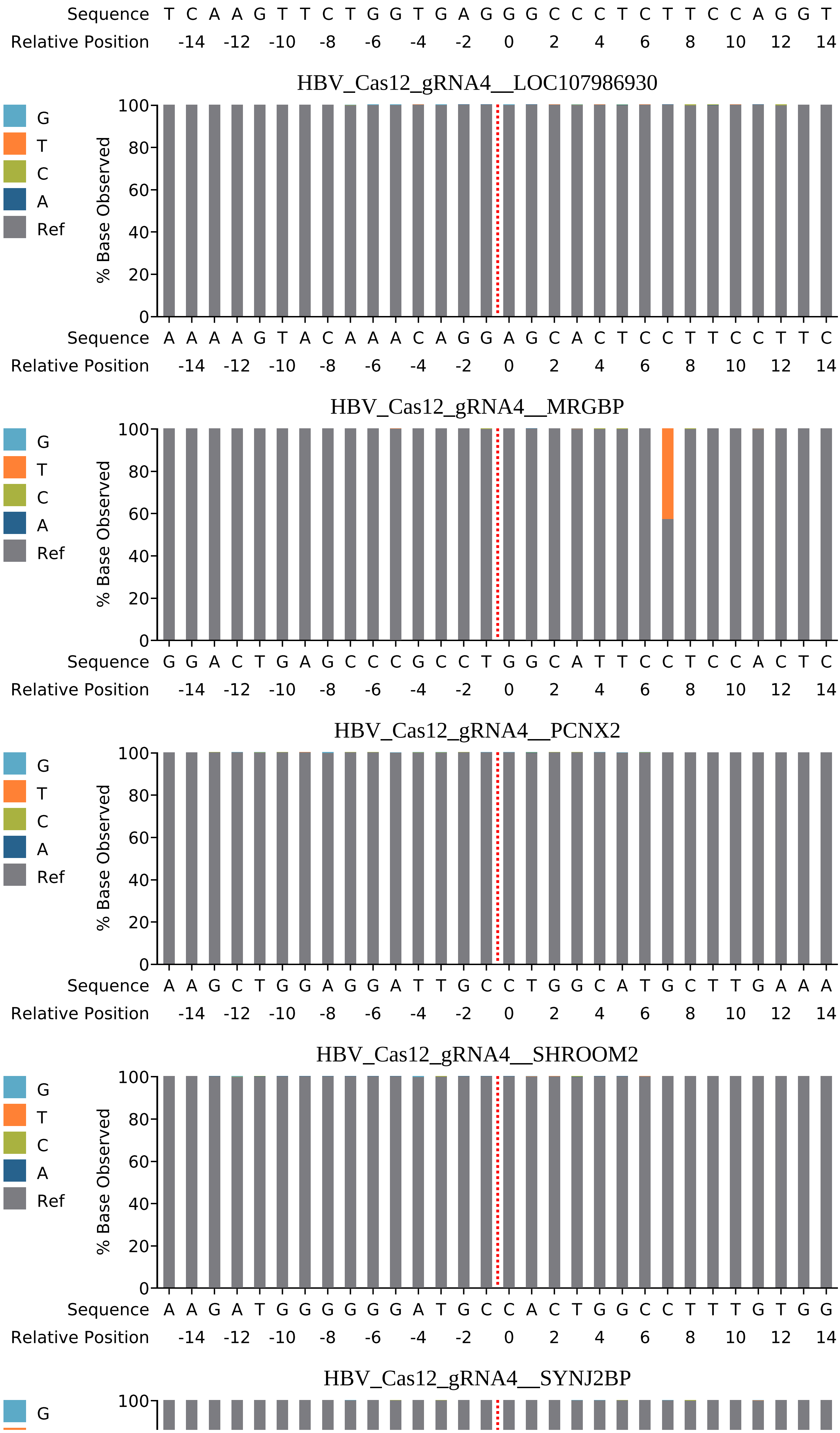
Sequence T C A A G T T C T G G T G A G G G C C C T C T T C C A G G T
Relative Position -14 -12 -10 -8 -6 -4 -2 0 2 4 6 8 10 12 14
HBV_Cas12_gRNA4__LOC107986930
G
T
C
A
Ref
% Base Observed
0 20 40 60 80 100
Sequence A A A A G T A C A A A C A G G A G C A C T C C T T C C T T C
Relative Position -14 -12 -10 -8 -6 -4 -2 0 2 4 6 8 10 12 14
HBV_Cas12_gRNA4__MRGBP
G
T
C
A
Ref
% Base Observed
0 20 40 60 80 100
Sequence G G A C T G A G C C C G C C T G G C A T T C C T C C A C T C
Relative Position -14 -12 -10 -8 -6 -4 -2 0 2 4 6 8 10 12 14
HBV_Cas12_gRNA4__PCNX2
G
T
C
A
Ref
% Base Observed
0 20 40 60 80 100
Sequence A A G C T G G A G G A T T G C C T G G C A T G C T T G A A A
Relative Position -14 -12 -10 -8 -6 -4 -2 0 2 4 6 8 10 12 14
HBV_Cas12_gRNA4__SHROOM2
G
T
C
A
Ref
% Base Observed
0 20 40 60 80 100
Sequence A A G A T G G G G G G A T G C C A C T G G C C T T T G T G G
Relative Position -14 -12 -10 -8 -6 -4 -2 0 2 4 6 8 10 12 14
HBV_Cas12_gRNA4__SYNJ2BP
G
100

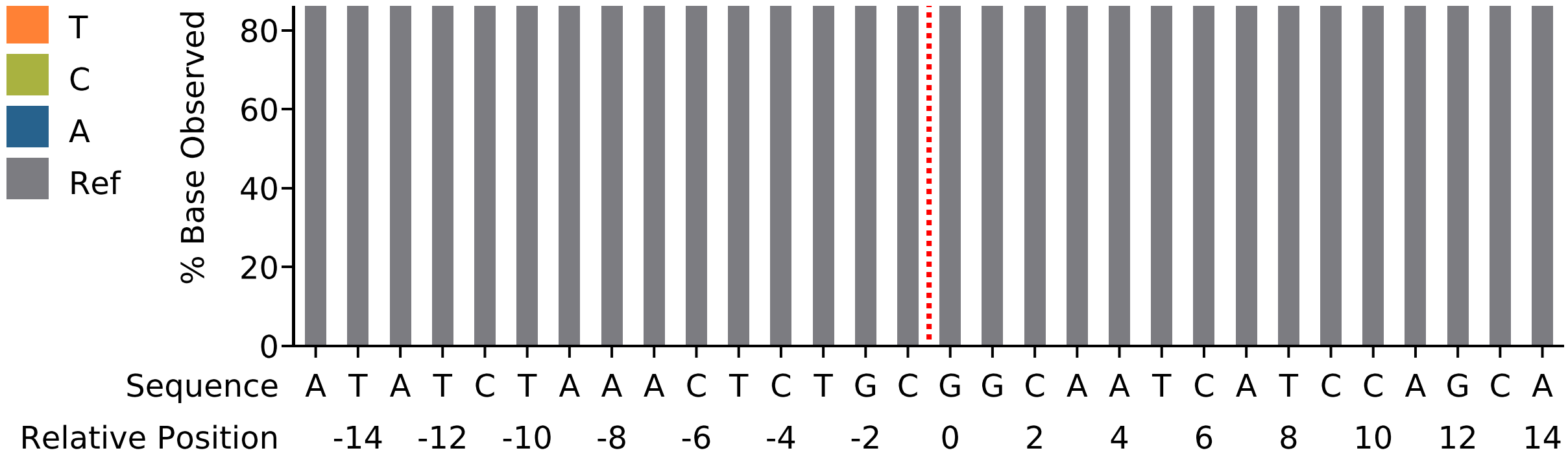

HBV_Cas12_gRNA4__TANK

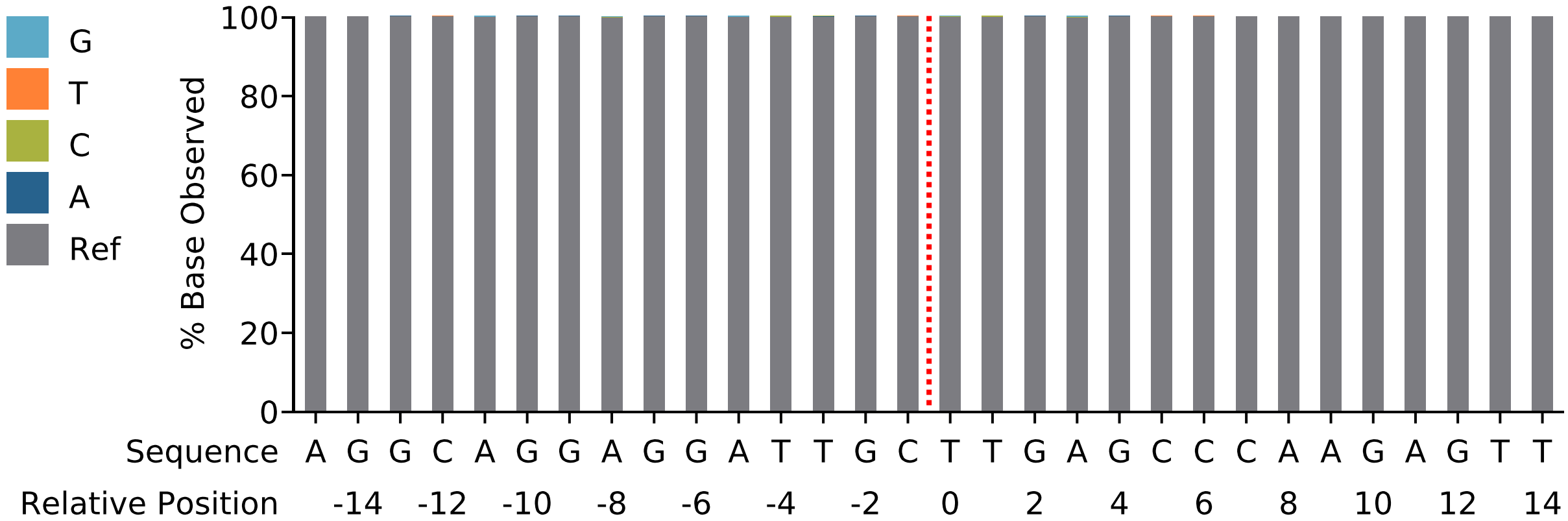

HBV_Cas12_gRNA4__TCF12

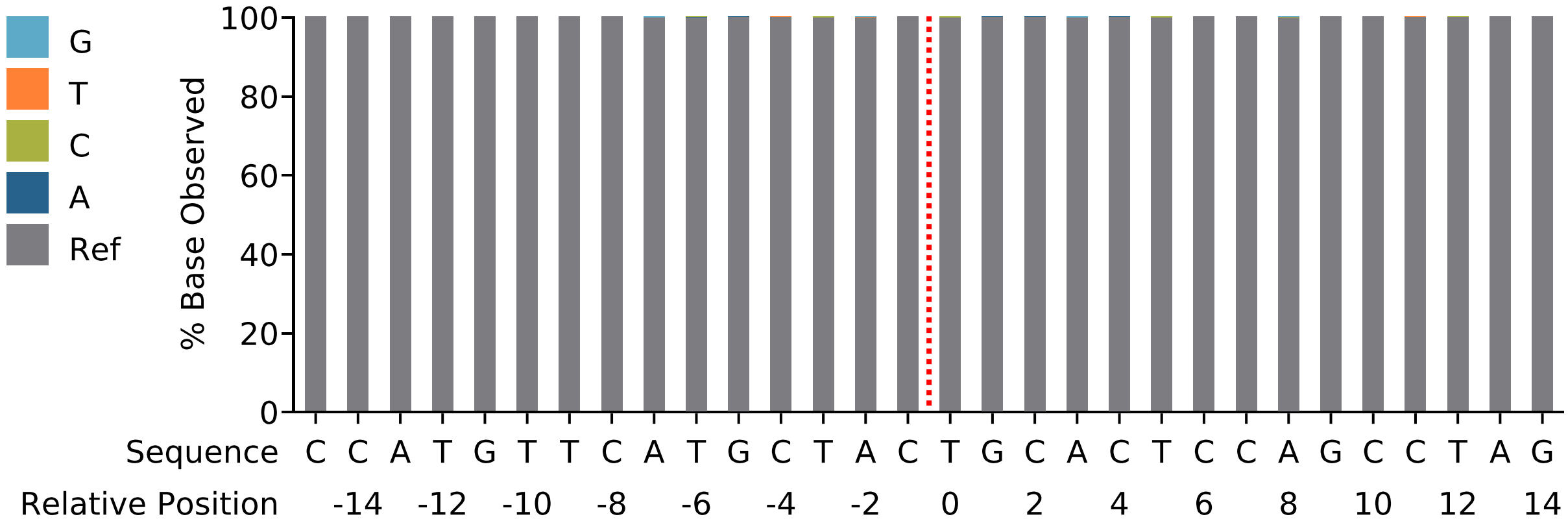

HBV_Cas12_gRNA4__TEKT5

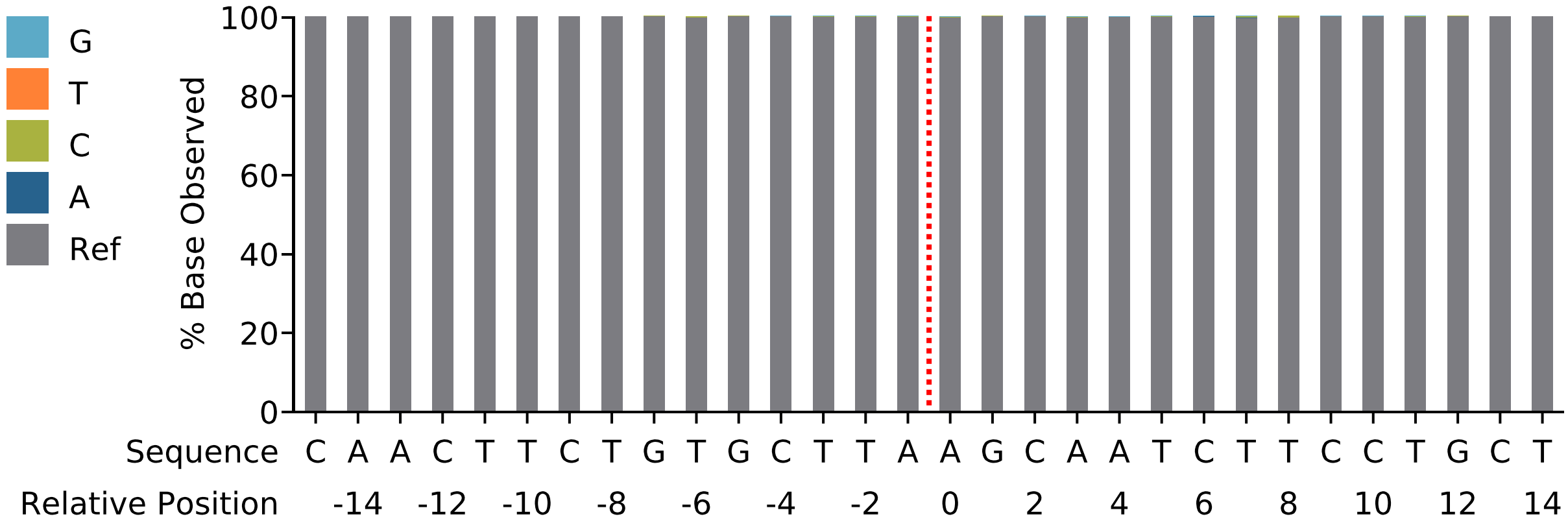

HBV_Cas12_gRNA4__VTI1A

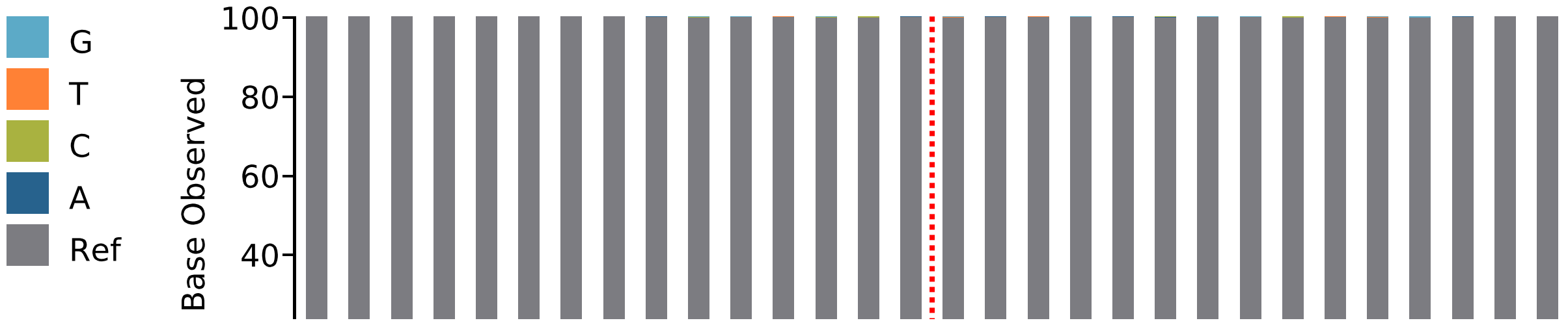

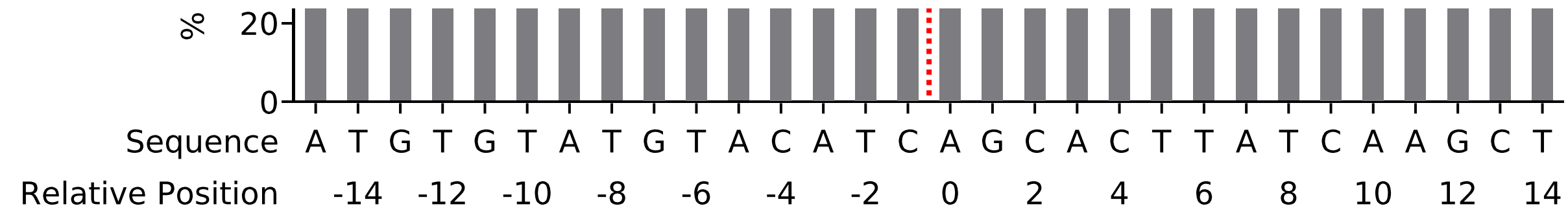

HBV_Cas12_gRNA4__ZBTB44-DT

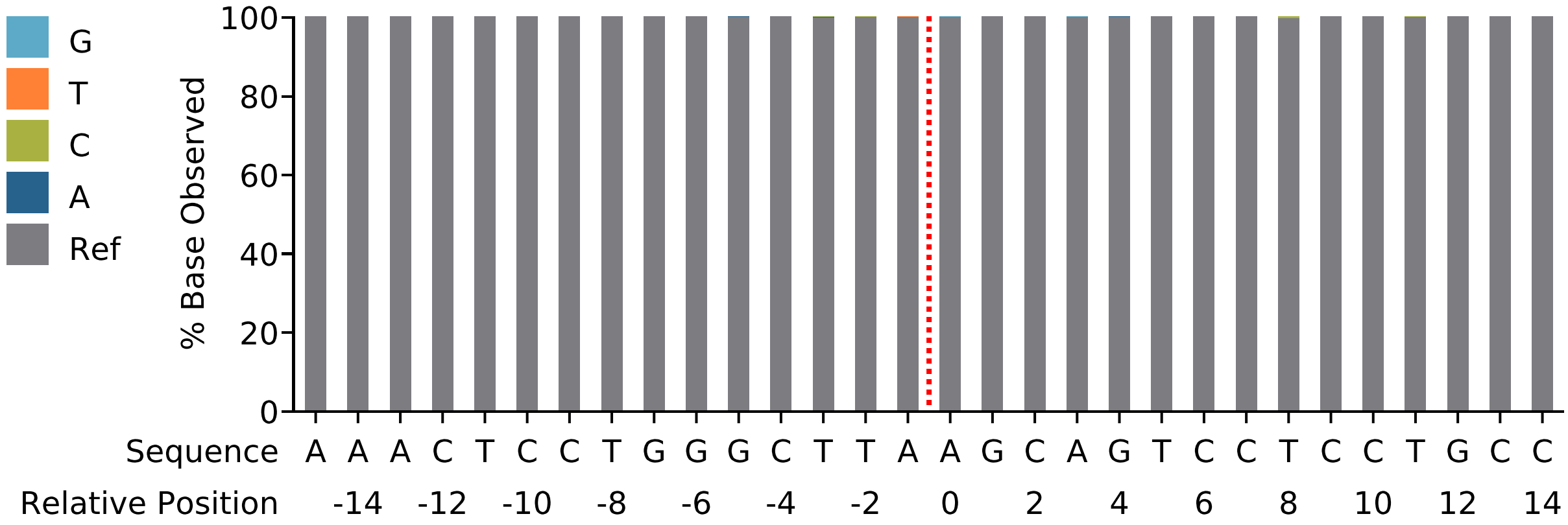

HBV_Cas12_gRNA4__ZNF292

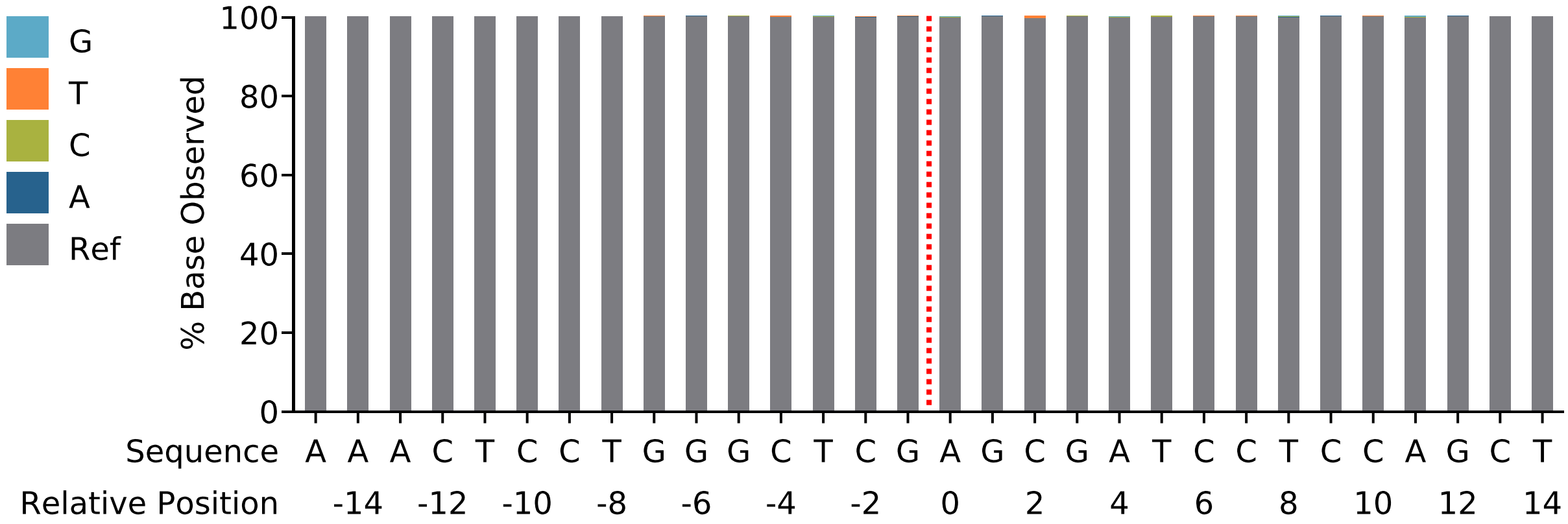

HBV_Cas12_gRNA4__none52

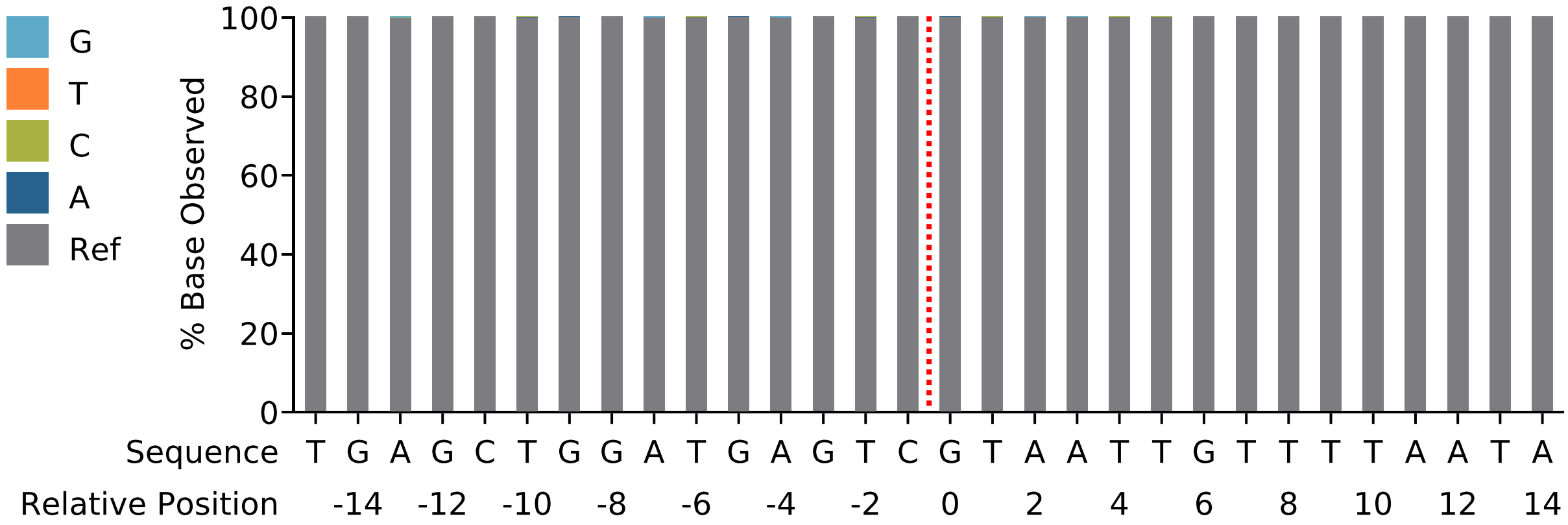

HBV_Cas12_gRNA4__none54

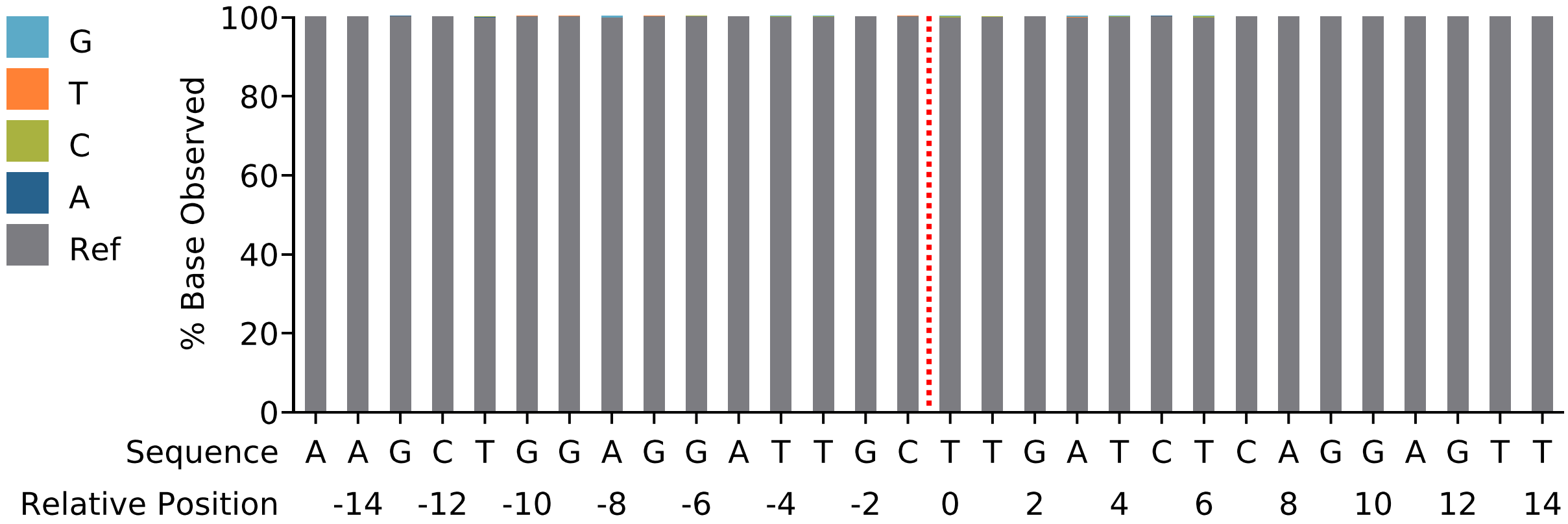

HBV_Cas12_gRNA4__none55

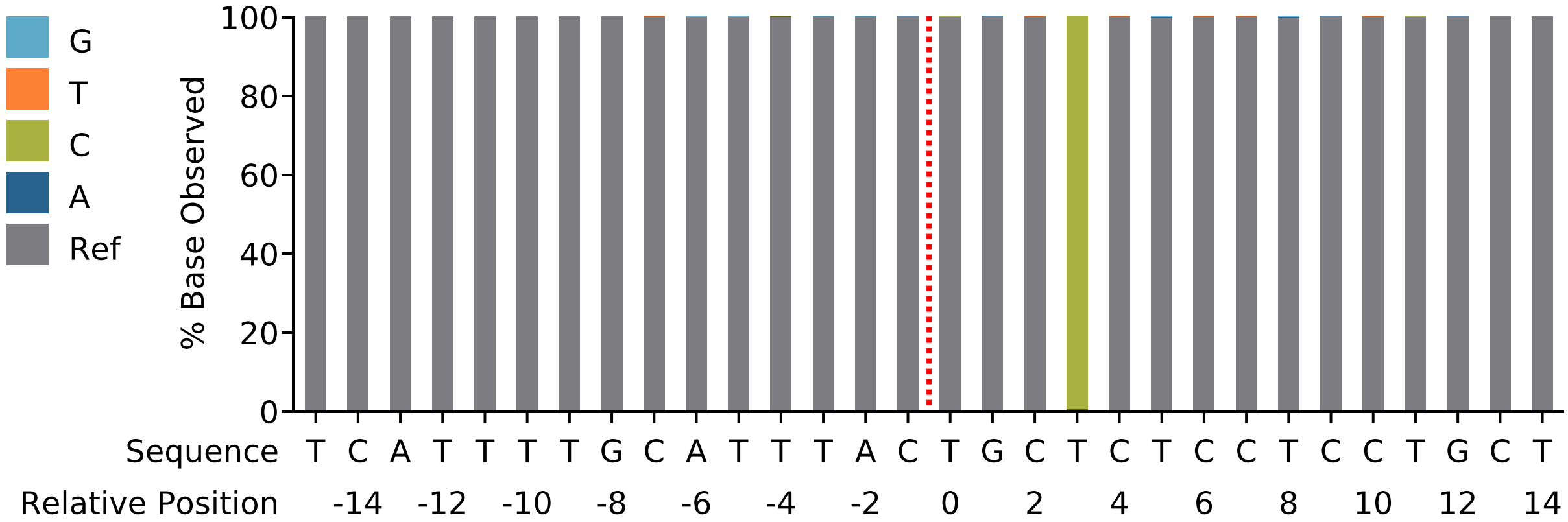

HBV_Cas12_gRNA4__none56

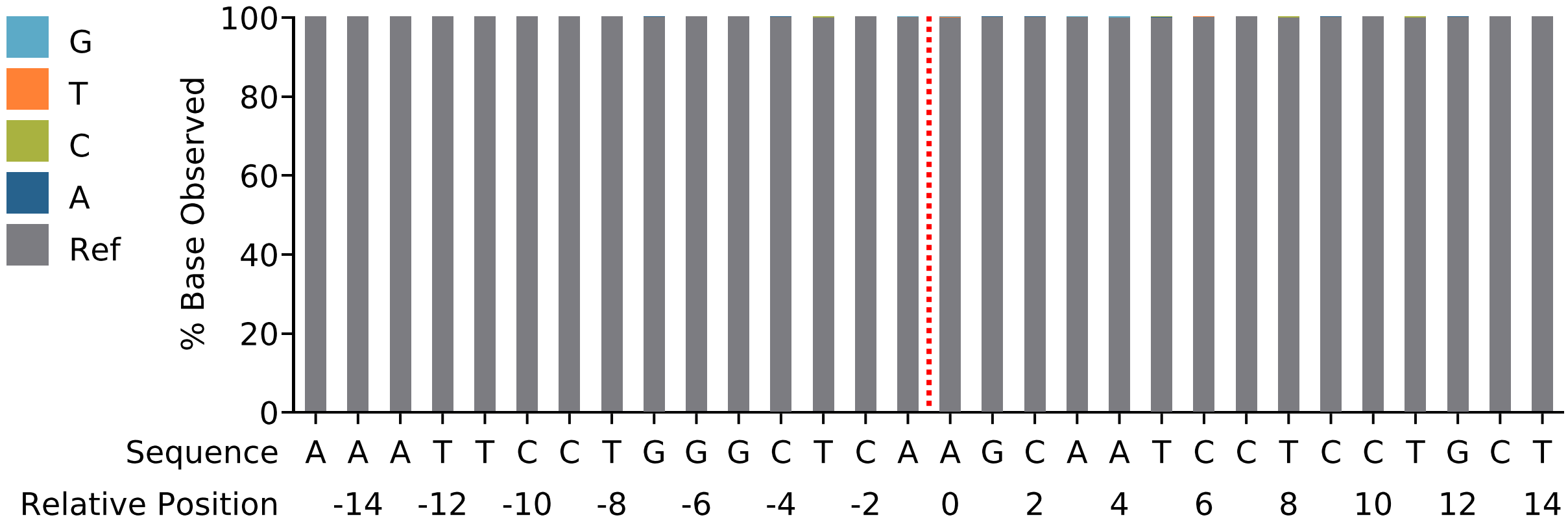

HBV_Cas12a_Control__CNTN451

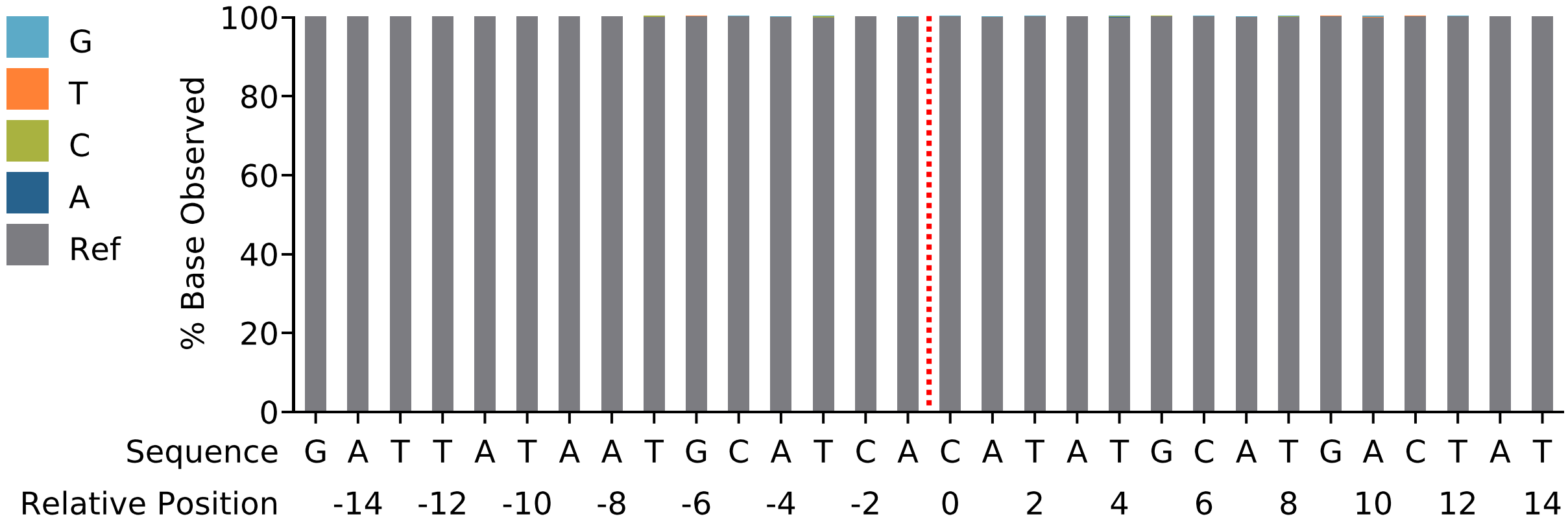

HBV_Cas12a_Control__POGLUT3

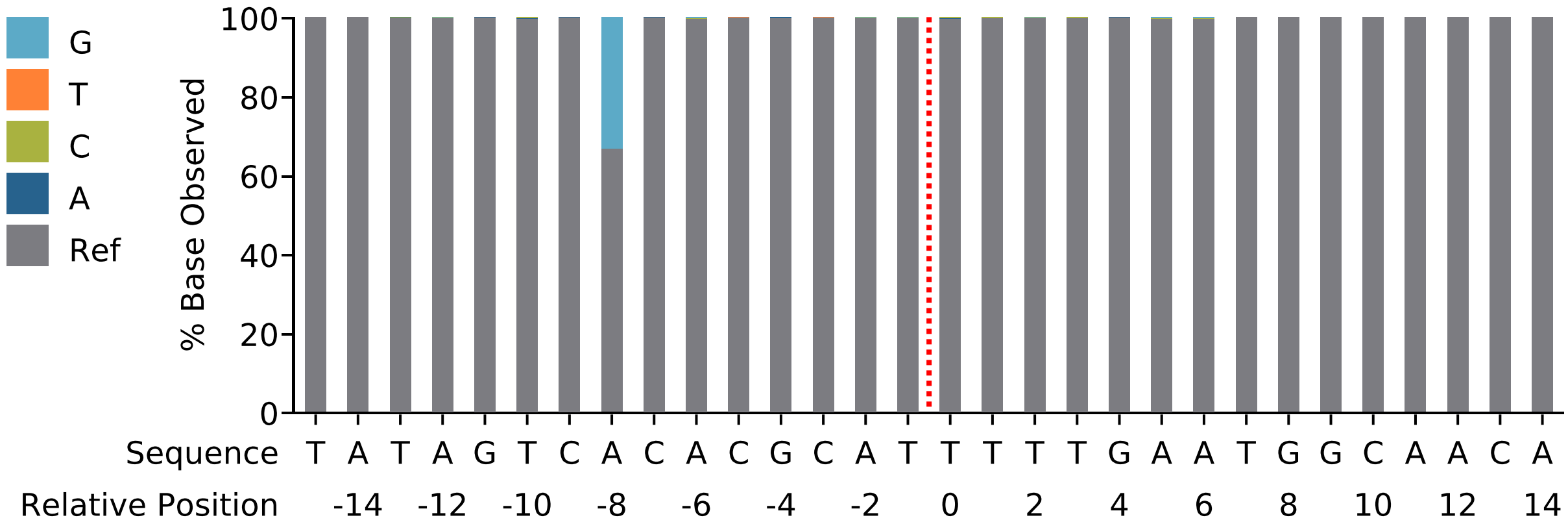

HBV_Cas12a_Control__ROR1

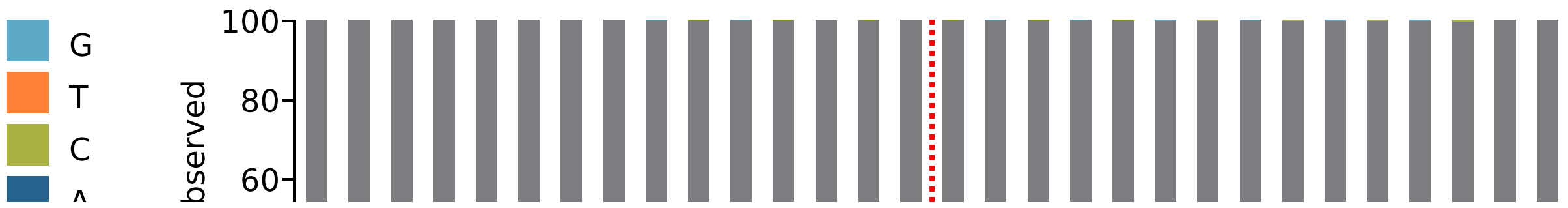

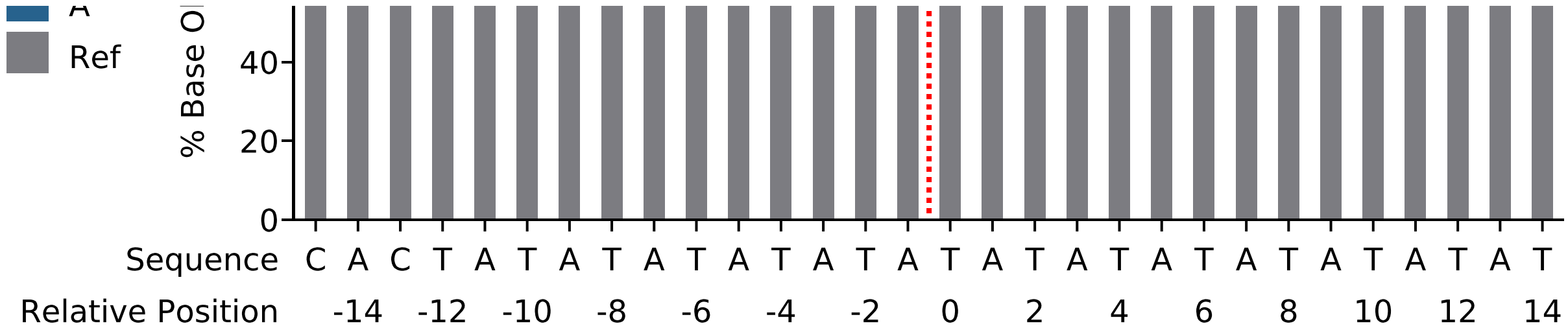

HBV_Cas12a_Control__SHISA6

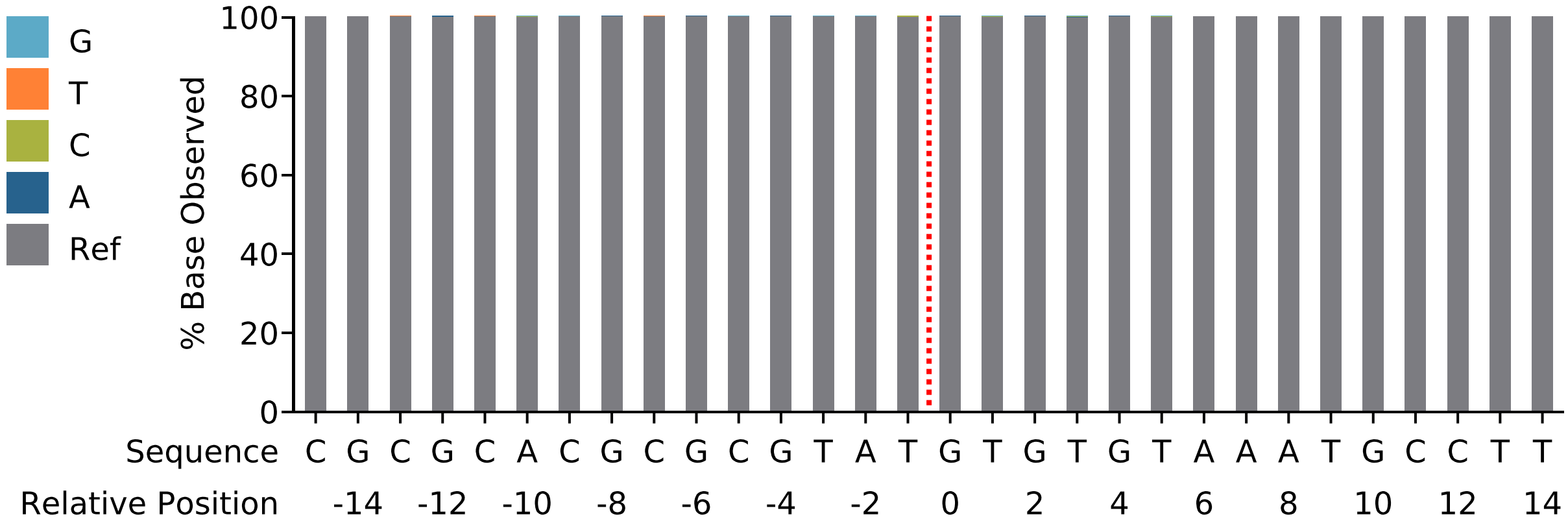

HBV_Cas12a_Control__TMC4

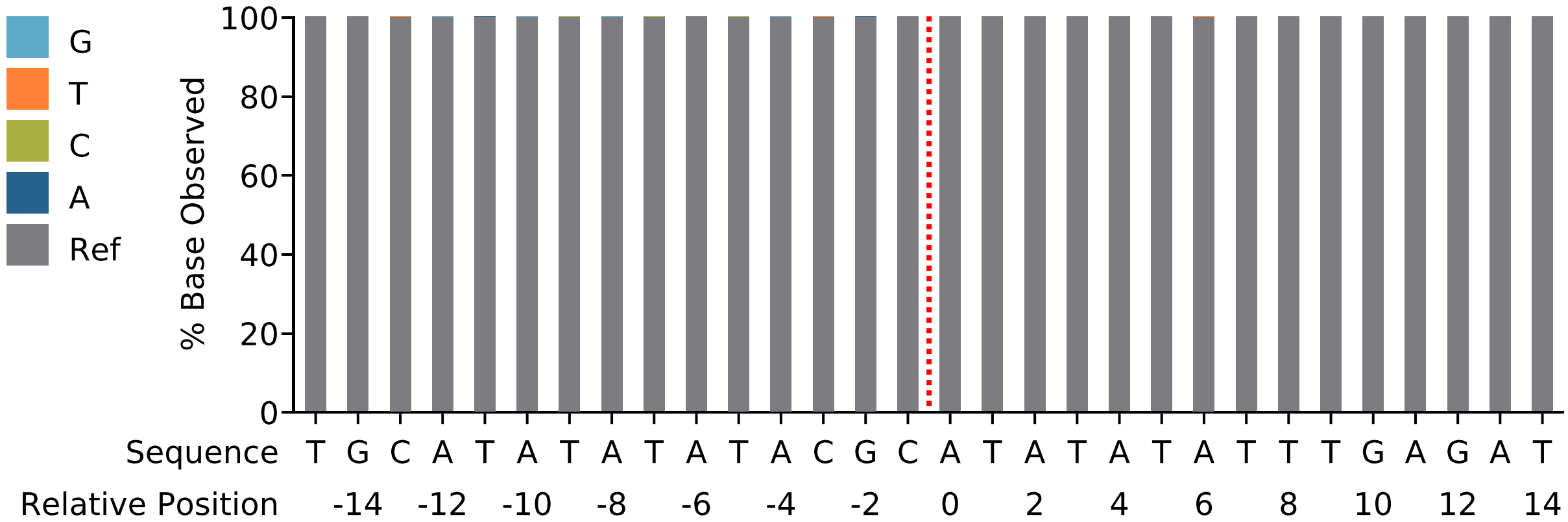

HBV_Cas12a_Control__none44

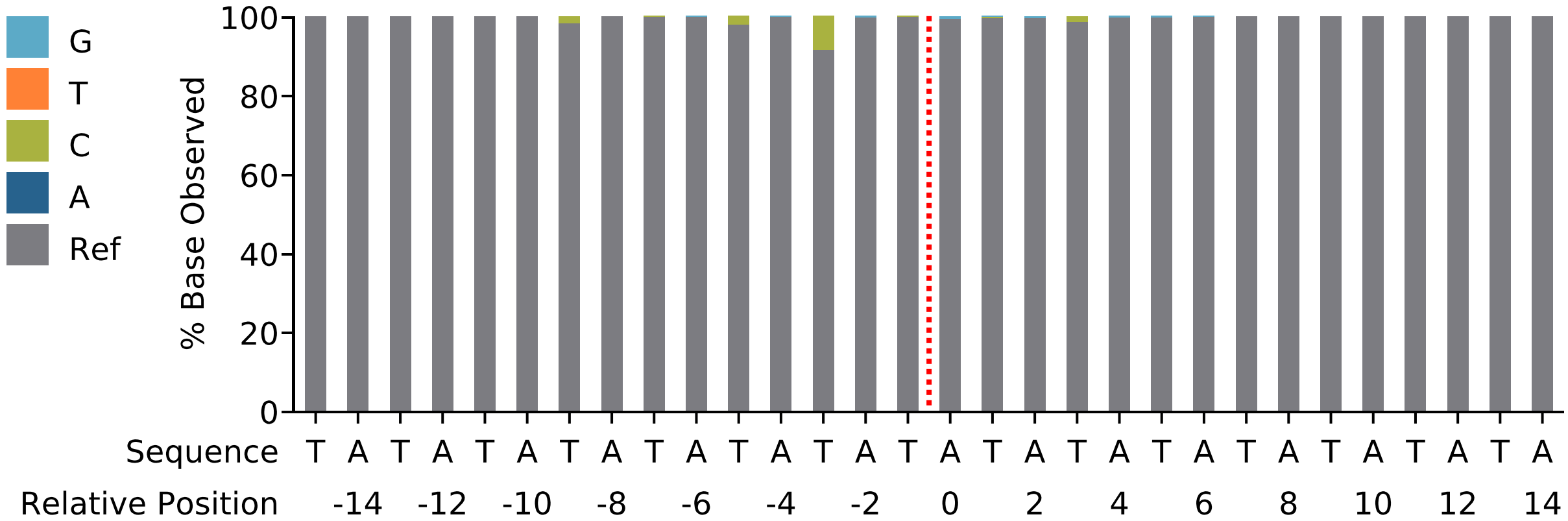

HBV_Cas12a_Control__none46

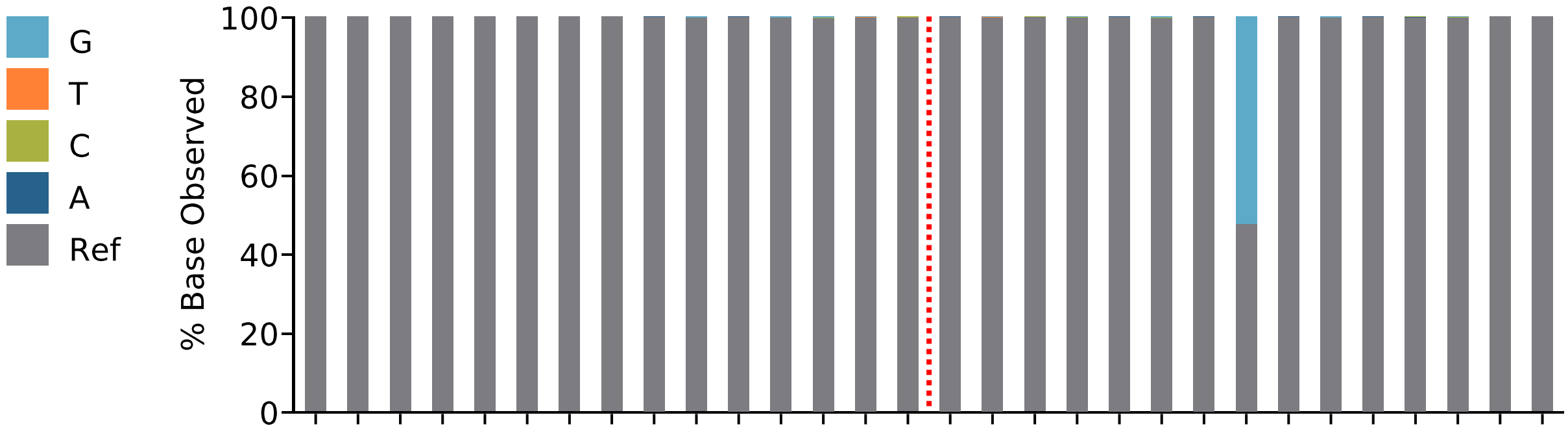

Sequence C C T A T A C A C A C A A A T C A T A C A C A C A C T A T A
Relative Position -14 -12 -10 -8 -6 -4 -2 0 2 4 6 8 10 12 14

HBV_Cas12a_Control__none47

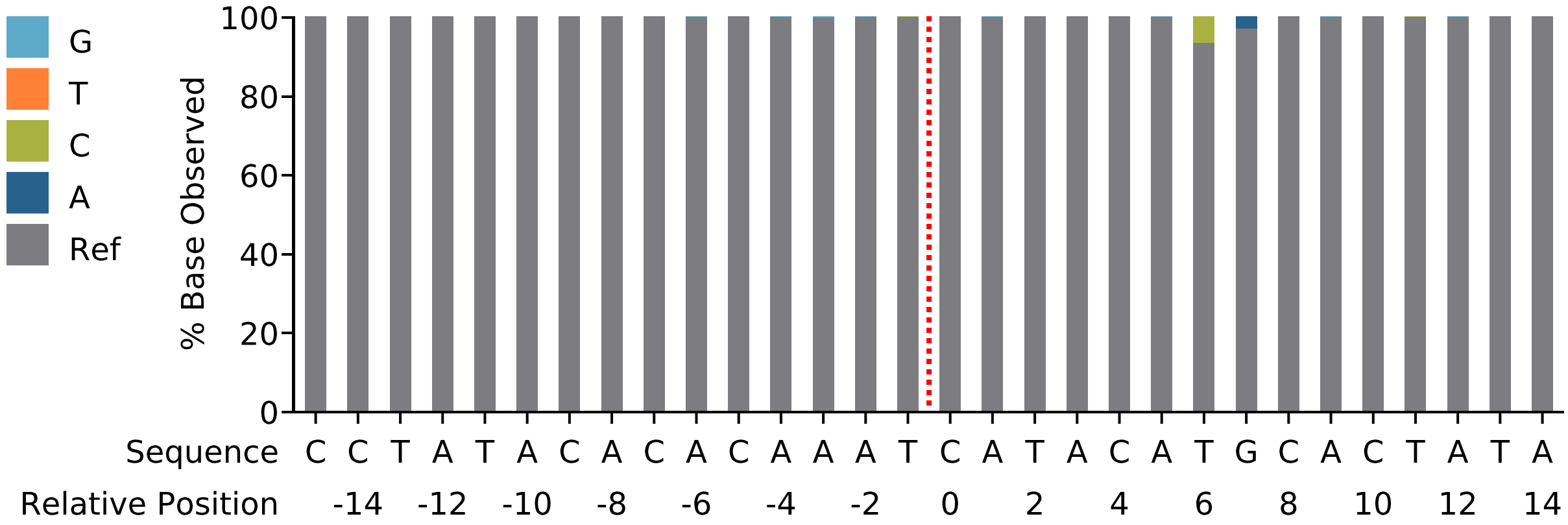

HBV_Cas12a_Control__none48

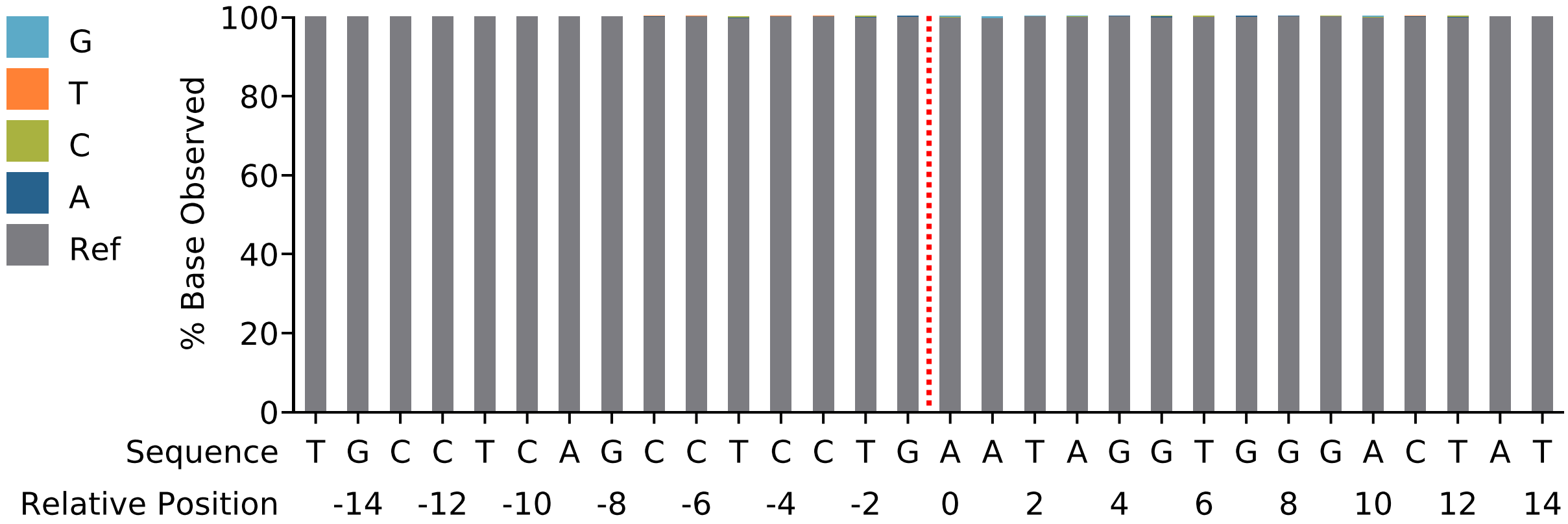

HBV_Cas12a_Control__none49

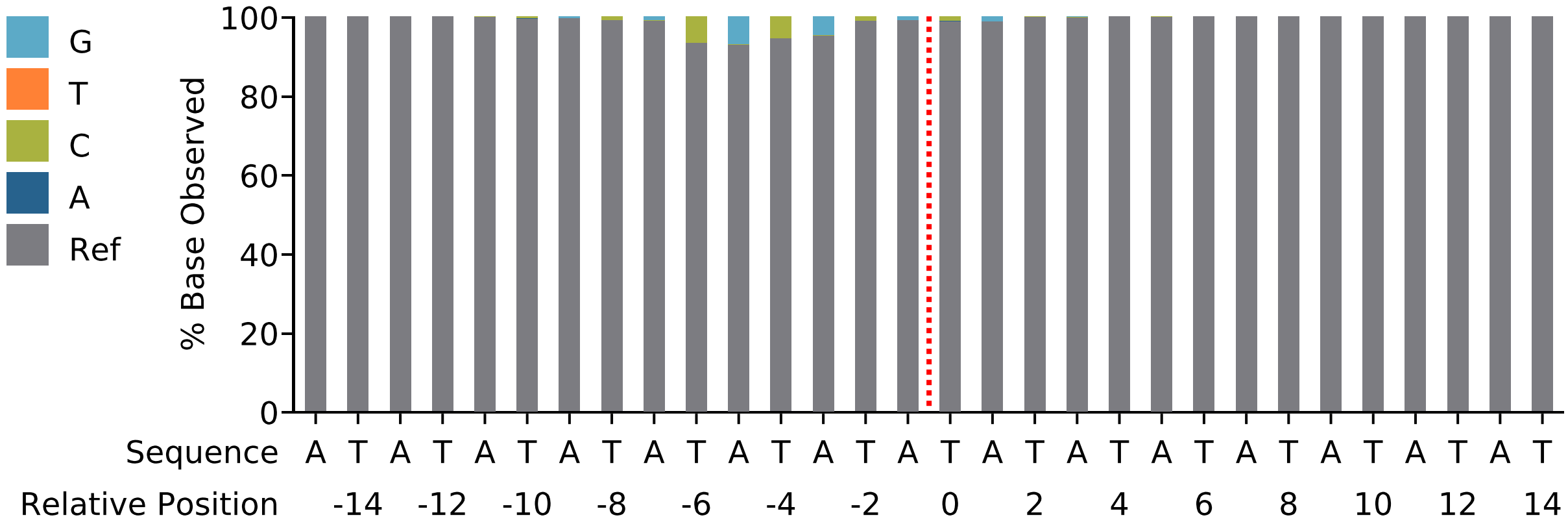

Supplement: Supplementary file 6 [file DataSheet6.pdf]
