# Supplementary material for: Combined RNA- and DNA-targeting CRISPR ribonucleoproteins enhance suppression of HBV transcripts
Source: Front Genome Ed. 2026 Sep 23;8:1824895. doi: 10.3389/fgeed.2026.1824895 (PMC13642003; doi:10.3389/fgeed.2026.1824895)

Analysis mode: CRISPAltRations 1_2_1
Start date: 2026/08/21 15:25
Targets: HBV_Cas9_EditR__ACTR3B, HBV_Cas9_EditR__CCDC60, HBV_Cas9_EditR__CLYBL, HBV_Cas9_EditR__FLYWCH1, HBV_Cas9_EditR__GALNT16, HBV_Cas9_EditR__JAM2, HBV_Cas9_EditR__LHPP, HBV_Cas9_EditR__LINC00301, HBV_Cas9_EditR__LOC105372647, HBV_Cas9_EditR__PRSS55, HBV_Cas9_EditR__RPS19BP1, HBV_Cas9_EditR__SUGP1, HBV_Cas9_EditR__TBCD, HBV_Cas9_EditR__none24, HBV_Cas9_gRNA9__DTNA, HBV_Cas9_gRNA9__LOC101929268, HBV_Cas9_gRNA9__MIR4663, HBV_Cas9_gRNA9__PARP11-AS1, HBV_Cas9_gRNA9__PDE1C, HBV_Cas9_gRNA9__PRRC2B, HBV_Cas9_gRNA9__SHISAL1, HBV_Cas9_gRNA9__none11, HBV_Cas9_gRNA9__none12, HBV_Cas9_gRNA9__none13, HBV_Cas9_gRNA9__none14, HBV_Cas9_gRNA9__none15, HBV_Cas9_gRNA9__none16, HBV_Cas9_gRNA9__none17, HBV_Cas9_gRNA9__none18, HBV_Cas9_gRNA9__none19, HBV_Cas9_gRNA9__none20, HBV_Cas9_gRNA9__none21, HBV_Cas9_gRNA9__none22

Metrics

	Group ID	Treated	Total Reads	Percent Edited	Percent Unedited	Percent NHEJ	Percent Perfect HDR	Percent Imperfect HDR	Percent HDR	Percent Other	Percent In Frame	Percent Frameshift	Percent Indels	Percent Insertions	Percent Templated Insertion	Percent GC-Insertion	Percent Deletions
**HBV_Cas9_EditR__ACTR3B**	HBV_Fig3	TRUE	28775	2.19	97.81	0.09	0.0	0.0	0.0	2.1	99.91	0.09	0.09	0.0	0.0	0.0	0.09
**HBV_Cas9_EditR__CCDC60**	HBV_Fig3	TRUE	9608	1.86	98.14	0.02	0.0	0.0	0.0	1.84	99.98	0.02	0.02	0.01	0.0	0.0	0.01
**HBV_Cas9_EditR__CLYBL**	HBV_Fig3	TRUE	56364	2.11	97.89	0.01	0.0	0.0	0.0	2.1	99.99	0.01	0.01	0.0	0.0	0.0	0.01
**HBV_Cas9_EditR__FLYWCH1**	HBV_Fig3	TRUE	41177	2.19	97.81	0.08	0.0	0.0	0.0	2.11	99.92	0.08	0.08	0.0	0.0	0.0	0.08
**HBV_Cas9_EditR__GALNT16**	HBV_Fig3	TRUE	241694	2.36	97.64	0.2	0.0	0.0	0.0	2.16	99.8	0.2	0.2	0.02	100.0	0.0	0.18
**HBV_Cas9_EditR__JAM2**	HBV_Fig3	TRUE	164841	2.57	97.43	0.06	0.0	0.0	0.0	2.51	99.94	0.06	0.06	0.02	100.0	0.0	0.04
**HBV_Cas9_EditR__LHPP**	HBV_Fig3	TRUE	48528	1.63	98.37	0.01	0.0	0.0	0.0	1.62	99.99	0.01	0.01	0.0	0.0	0.0	0.01
**HBV_Cas9_EditR__LINC00301**	HBV_Fig3	TRUE	115928	99.99	0.01	0.39	0.0	0.0	0.0	99.6	99.96	0.04	0.39	0.0	50.0	0.0	0.39
**HBV_Cas9_EditR__LOC105372647**	HBV_Fig3	TRUE	2864061	88.09	11.91	0.89	0.0	0.0	0.0	87.2	99.15	0.85	0.89	0.59	3.21	0.0	0.33
**HBV_Cas9_EditR__PRSS55**	HBV_Fig3	TRUE	251226	99.91	0.09	0.07	0.0	0.0	0.0	99.84	99.93	0.07	0.07	0.0	90.91	0.0	0.06
**HBV_Cas9_EditR__RPS19BP1**	HBV_Fig3	TRUE	50475	2.16	97.84	0.09	0.0	0.0	0.0	2.07	99.91	0.09	0.09	0.0	100.0	0.0	0.08
**HBV_Cas9_EditR__SUGP1**	HBV_Fig3	TRUE	43836	2.37	97.63	0.15	0.0	0.0	0.0	2.22	99.85	0.15	0.15	0.0	100.0	0.0	0.15
**HBV_Cas9_EditR__TBCD**	HBV_Fig3	TRUE	40730	2.52	97.48	0.05	0.0	0.0	0.0	2.47	99.95	0.05	0.05	0.01	100.0	0.0	0.04
**HBV_Cas9_EditR__none24**	HBV_Fig3	TRUE	82876	3.0	97.0	0.03	0.0	0.0	0.0	2.97	99.97	0.03	0.03	0.0	0.0	0.0	0.03
**HBV_Cas9_gRNA9__DTNA**	HBV_Fig3	TRUE	68336	67.14	32.86	0.01	0.0	0.0	0.0	67.13	99.99	0.01	0.01	0.0	0.0	0.0	0.01
**HBV_Cas9_gRNA9__LOC101929268**	HBV_Fig3	TRUE	137608	2.84	97.16	0.02	0.0	0.0	0.0	2.82	99.98	0.02	0.02	0.0	0.0	0.0	0.02
**HBV_Cas9_gRNA9__MIR4663**	HBV_Fig3	TRUE	184855	2.84	97.16	0.12	0.0	0.0	0.0	2.72	99.88	0.12	0.12	0.1	0.0	0.0	0.11
**HBV_Cas9_gRNA9__PARP11-AS1**	HBV_Fig3	TRUE	207134	2.29	97.71	0.01	0.0	0.0	0.0	2.28	99.99	0.01	0.01	0.0	0.0	0.0	0.01
**HBV_Cas9_gRNA9__PDE1C**	HBV_Fig3	TRUE	122486	2.3	97.7	0.12	0.0	0.0	0.0	2.18	99.88	0.12	0.12	0.02	100.0	0.0	0.11
**HBV_Cas9_gRNA9__PRRC2B**	HBV_Fig3	TRUE	117535	2.49	97.51	0.19	0.0	0.0	0.0	2.3	99.81	0.19	0.19	0.06	98.48	0.0	0.14
**HBV_Cas9_gRNA9__SHISAL1**	HBV_Fig3	TRUE	166663	2.65	97.35	0.14	0.0	0.0	0.0	2.51	99.86	0.14	0.14	0.03	86.21	0.0	0.1
**HBV_Cas9_gRNA9__none11**	HBV_Fig3	TRUE	13978	2.65	97.35	0.01	0.0	0.0	0.0	2.64	99.99	0.01	0.01	0.0	0.0	0.0	0.01
**HBV_Cas9_gRNA9__none12**	HBV_Fig3	TRUE	52092	2.34	97.66	0.1	0.0	0.0	0.0	2.24	99.96	0.04	0.1	0.0	0.0	0.0	0.1
**HBV_Cas9_gRNA9__none13**	HBV_Fig3	TRUE	4454	53.84	46.16	51.59	0.0	0.0	0.0	2.25	48.43	51.57	51.59	0.02	0.0	0.0	51.59
**HBV_Cas9_gRNA9__none14**	HBV_Fig3	TRUE	165105	3.01	96.99	0.16	0.0	0.0	0.0	2.85	99.84	0.16	0.16	0.04	100.0	0.0	0.13
**HBV_Cas9_gRNA9__none15**	HBV_Fig3	TRUE	47072	2.31	97.69	0.0	0.0	0.0	0.0	2.31	100.0	0.0	0.0	0.0	0.0	0.0	0.0
**HBV_Cas9_gRNA9__none16**	HBV_Fig3	TRUE	141	0.71	99.29	0.0	0.0	0.0	0.0	0.71	100.0	0.0	0.0	0.0	0.0	0.0	0.0
**HBV_Cas9_gRNA9__none17**	HBV_Fig3	TRUE	182312	2.62	97.38	0.01	0.0	0.0	0.0	2.61	99.99	0.01	0.01	0.0	100.0	0.0	0.01
**HBV_Cas9_gRNA9__none18**	HBV_Fig3	TRUE	99017	5.32	94.68	0.95	0.0	0.0	0.0	4.37	99.06	0.94	0.95	0.06	0.0	0.0	0.89
**HBV_Cas9_gRNA9__none19**	HBV_Fig3	TRUE	104060	2.58	97.42	0.03	0.0	0.0	0.0	2.55	99.97	0.03	0.03	0.0	0.0	0.0	0.03
**HBV_Cas9_gRNA9__none20**	HBV_Fig3	TRUE	142529	27.53	72.47	0.02	0.0	0.0	0.0	27.51	99.99	0.01	0.02	0.01	0.0	0.0	0.01

**HBV_Cas9_gRNA9__none21**	HBV_Fig3	TRUE	121156	2.54	97.46	0.29	0.0	0.0	0.0	2.25	99.71	0.29	0.29	0.06	85.51	0.0	0.23	
**HBV_Cas9_gRNA9__none22**	HBV_Fig3	TRUE	140766	2.57	97.43	0.06	0.0	0.0	0.0	2.51	99.96	0.04	0.06	0.03	0.0	0.0	0.03	

Percentages

	% Edited	*% Repair Pathways*	*% Frameshift*
HBV_Cas9_EditR__ACTR3B	97.81%	97.81%	99.91%
HBV_Cas9_EditR__CCDC60	98.14%	98.14%	99.98%
HBV_Cas9_EditR__CLYBL	97.89%	97.89%	99.99%
HBV_Cas9_EditR__FLYWCH1	97.81%	97.81%	99.92%
HBV_Cas9_EditR__GALNT16	97.64%	97.64%	99.8%
HBV_Cas9_EditR__JAM2	97.43%	97.43%	99.94%
HBV_Cas9_EditR__LHPP	98.37%	98.37%	99.99%
HBV_Cas9_EditR__LINC00301	99.99%	99.6%	99.96%
HBV_Cas9_EditR__LOC105372647	.91 88.09%	.91 87.2%	99.15%
HBV_Cas9_EditR__PRSS55	99.91%	99.84%	99.93%
HBV_Cas9_EditR__RPS19BP1	97.84%	97.84%	99.91%
HBV_Cas9_EditR__SUGP1	97.63%	97.63%	99.85%
HBV_Cas9_EditR__TBCD	97.48%	97.48%	99.95%
HBV_Cas9_EditR__none24	97.0%	97.0%	99.97%
HBV_Cas9_gRNA9__DTNA	32.86% 67.14%	32.86% 67.13%	99.99%
HBV_Cas9_gRNA9__LOC101929268	97.16%	97.16%	99.98%
HBV_Cas9_gRNA9__MIR4663	97.16%	97.16%	99.88%
HBV_Cas9_gRNA9__PARP11-AS1	97.71%	97.71%	99.99%
HBV_Cas9_gRNA9__PDE1C	97.7%	97.7%	99.88%
HBV_Cas9_gRNA9__PRRC2B	97.51%	97.51%	99.81%
HBV_Cas9_gRNA9__SHISAL1	97.35%	97.35%	99.86%
HBV_Cas9_gRNA9__none11	97.35%	97.35%	99.99%
HBV_Cas9_gRNA9__none12	97.66%	97.66%	99.96%
HBV_Cas9_gRNA9__none13	46.16% 53.84%	46.16% 51.59%	48.43% 51.57%
HBV_Cas9_gRNA9__none14	96.99%	96.99%	99.84%
HBV_Cas9_gRNA9__none15	97.69%	97.69%	100.0%

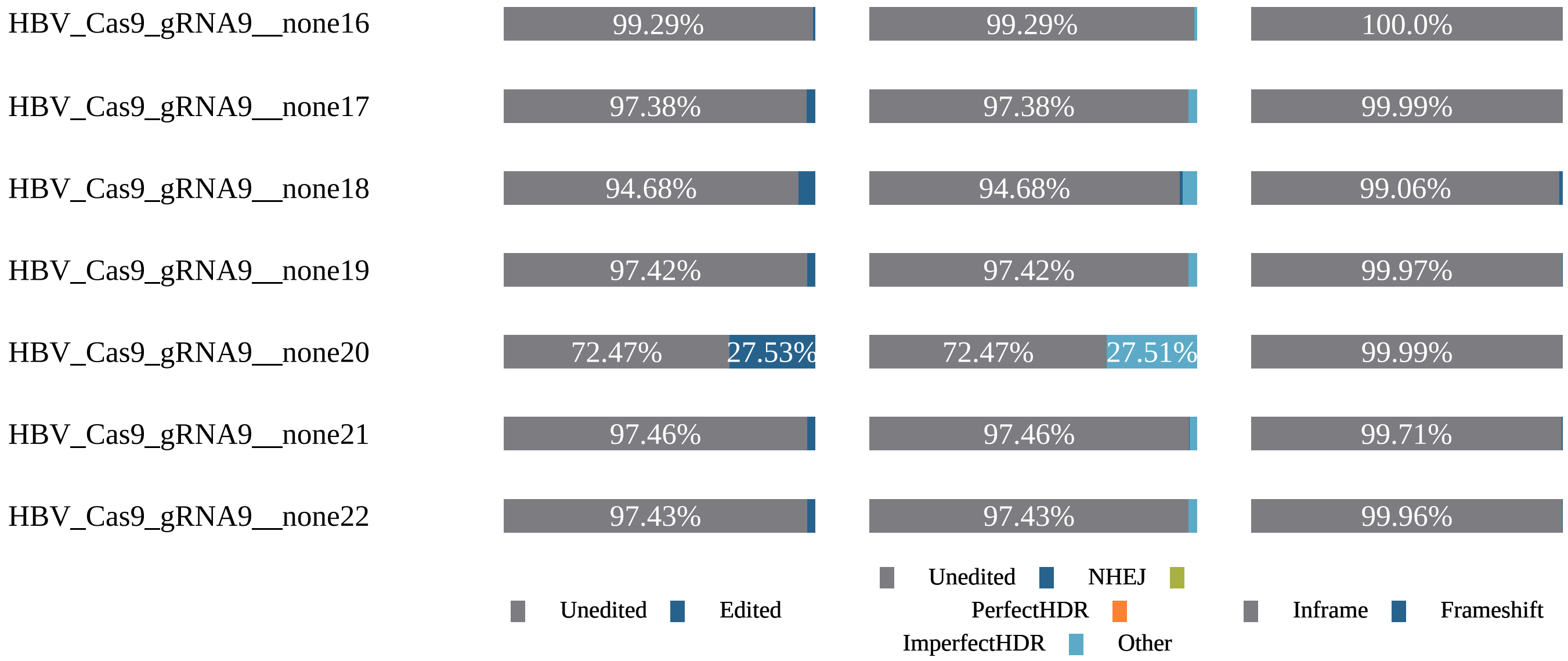

Insertions

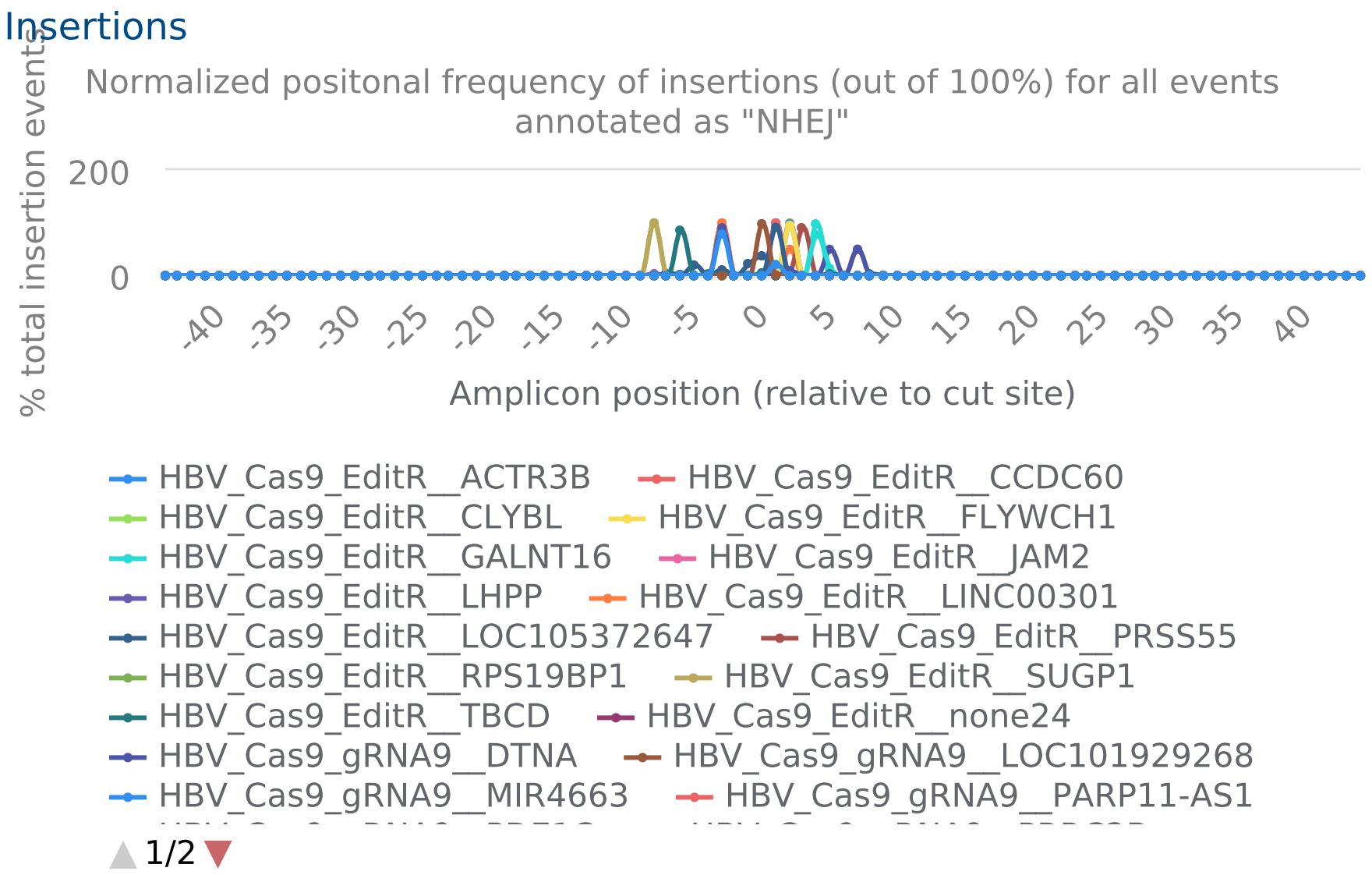

Deletions

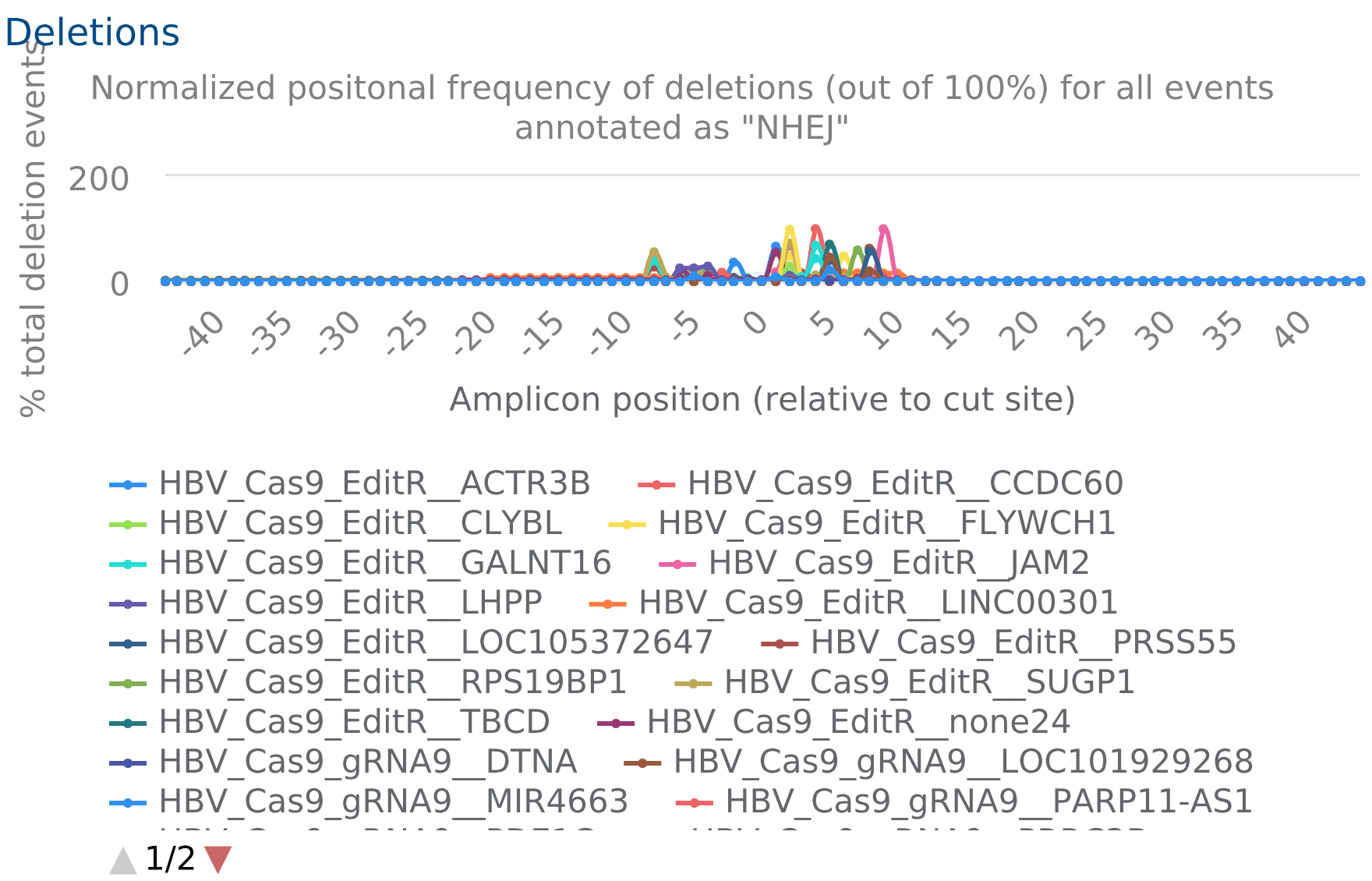

Indel Frequencies

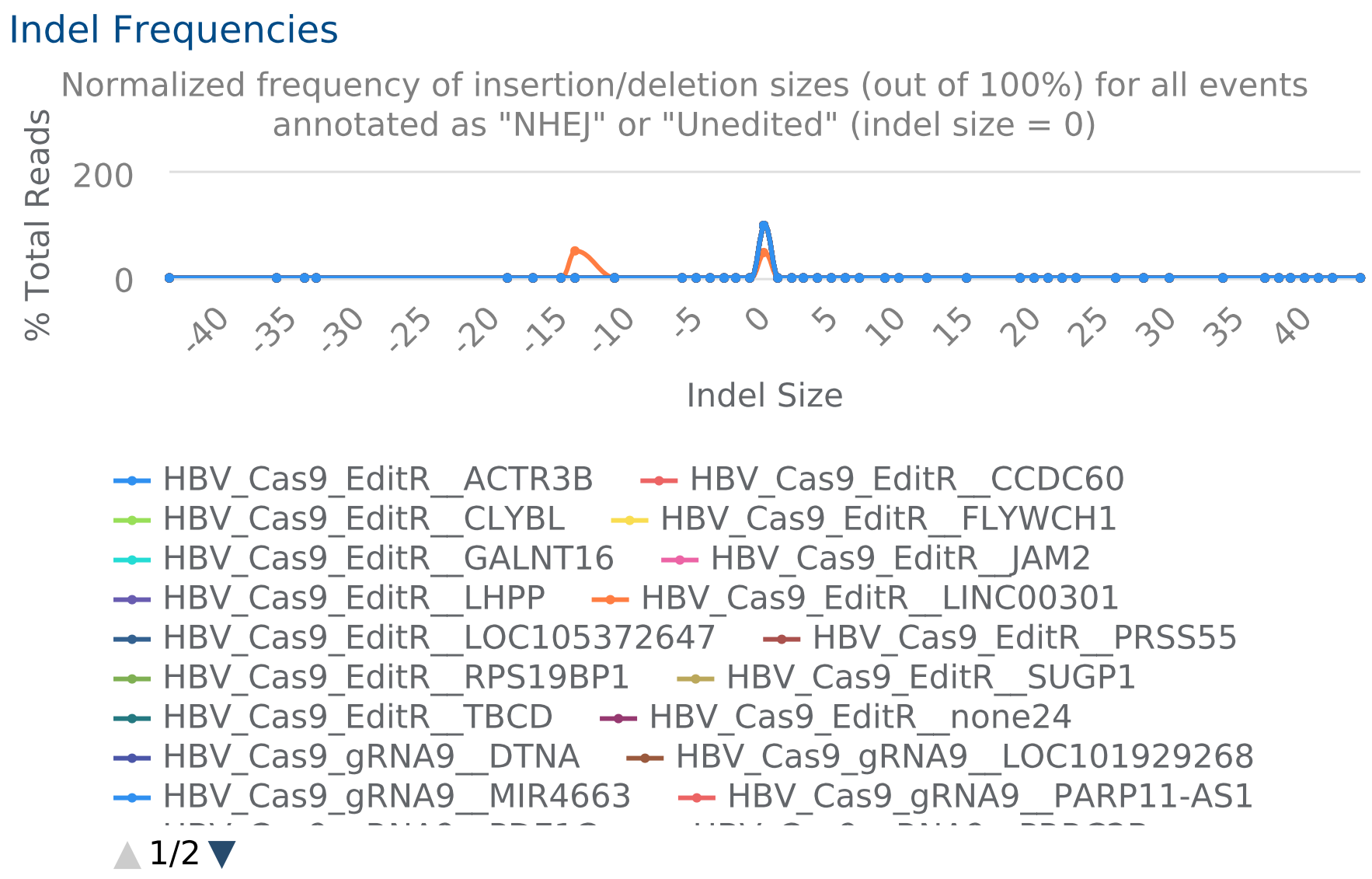

SNP Graph

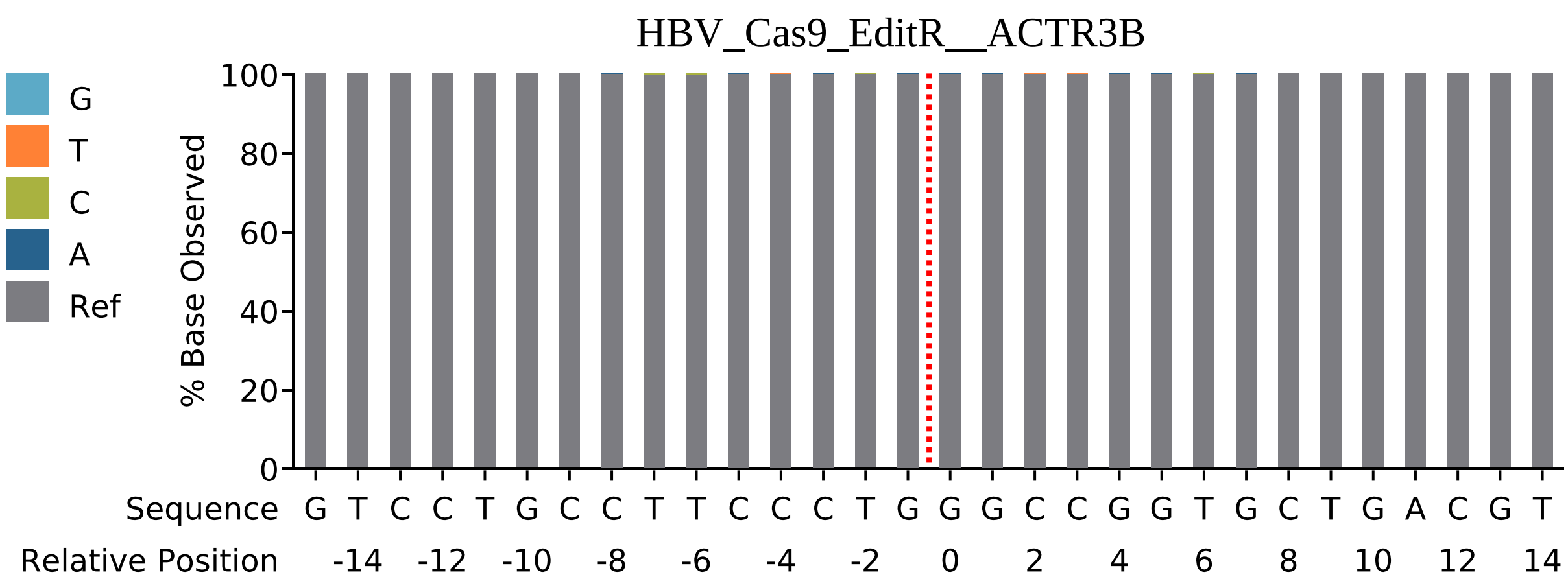

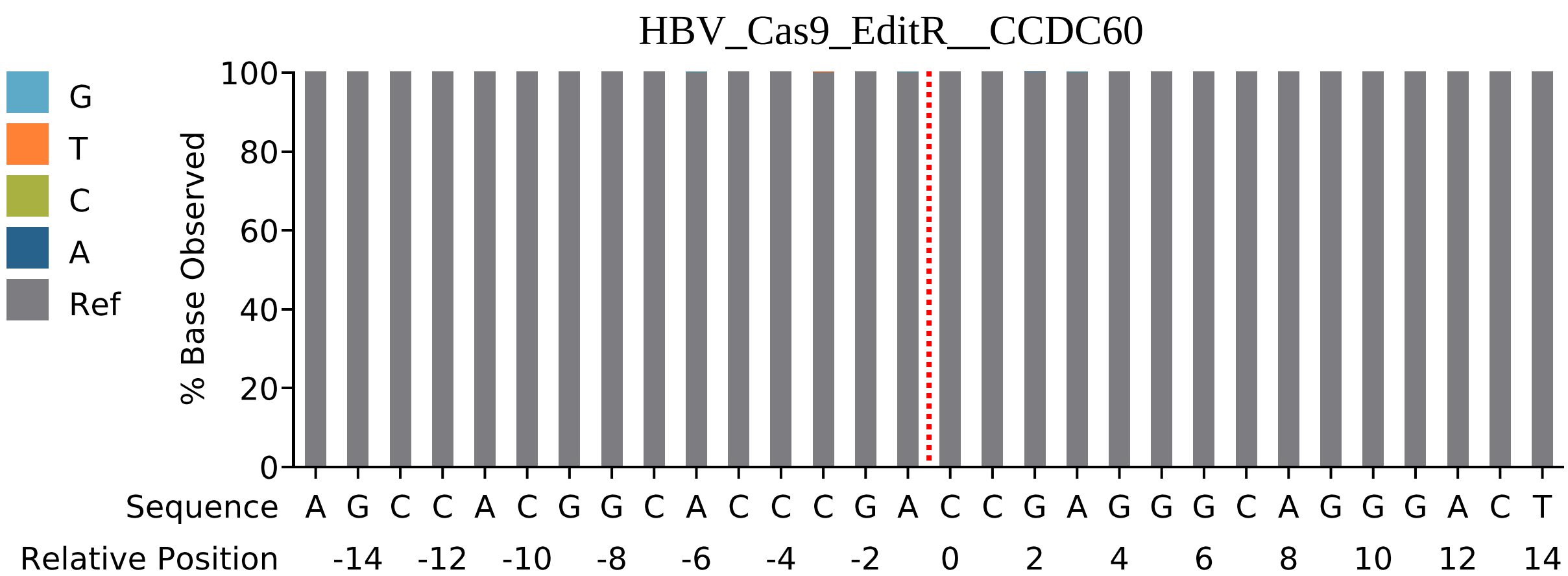

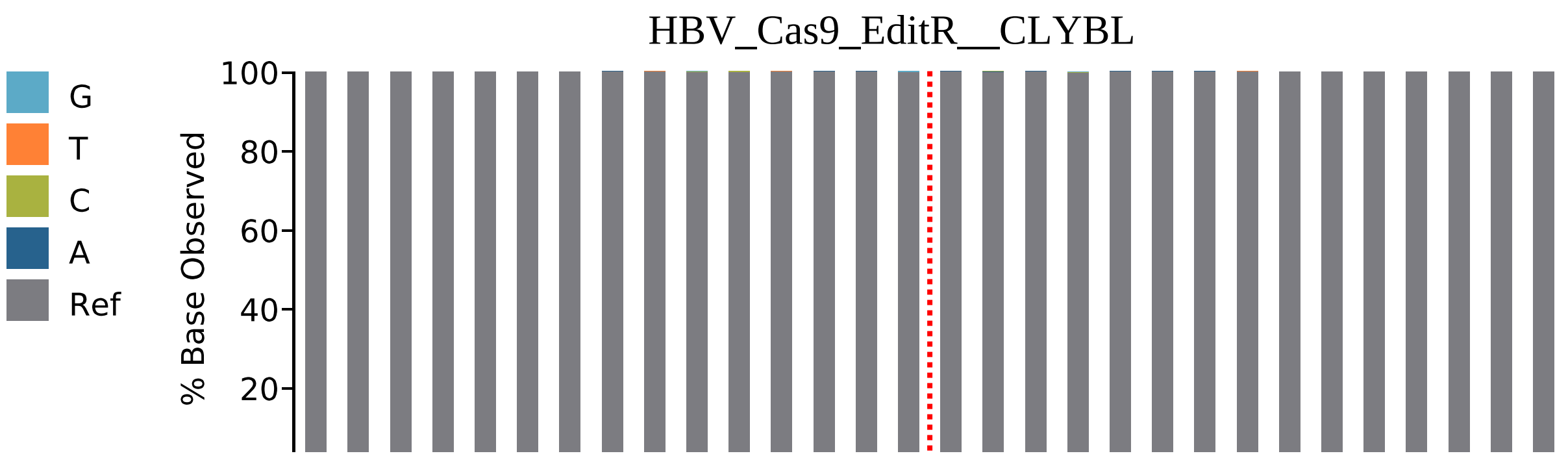

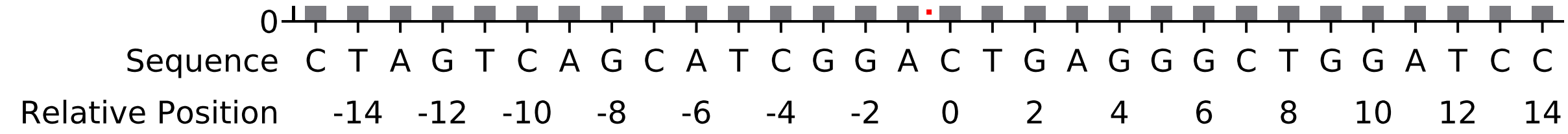

HBV_Cas9_EditR__FLYWCH1

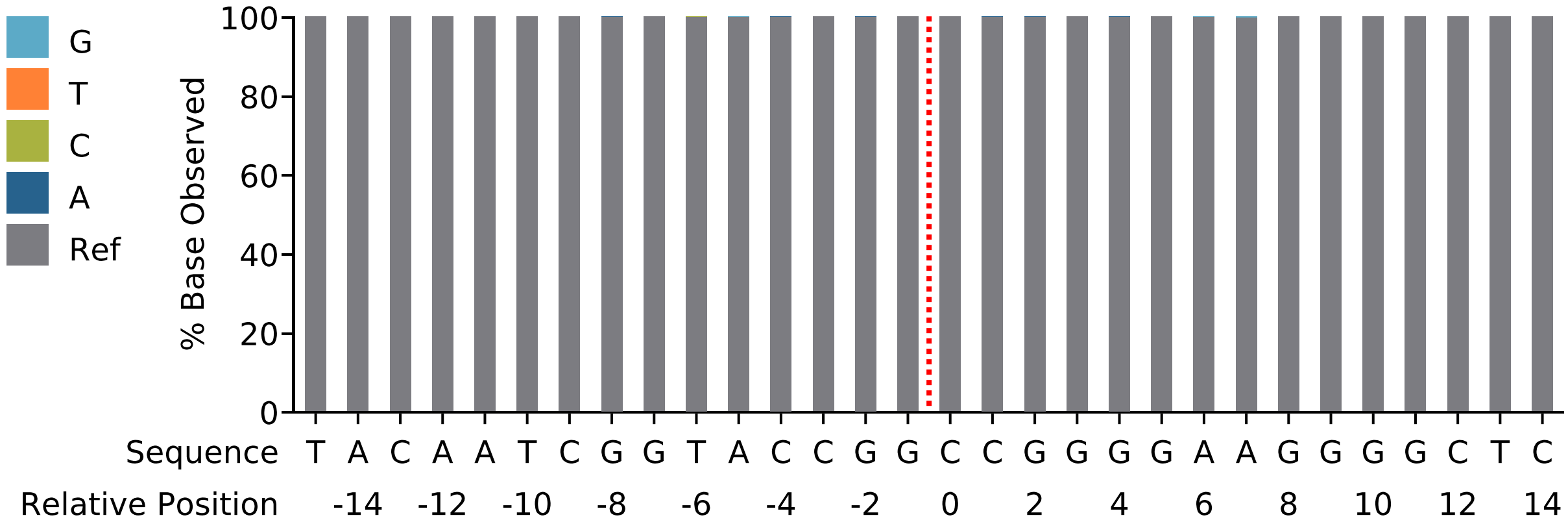

HBV_Cas9_EditR__GALNT16

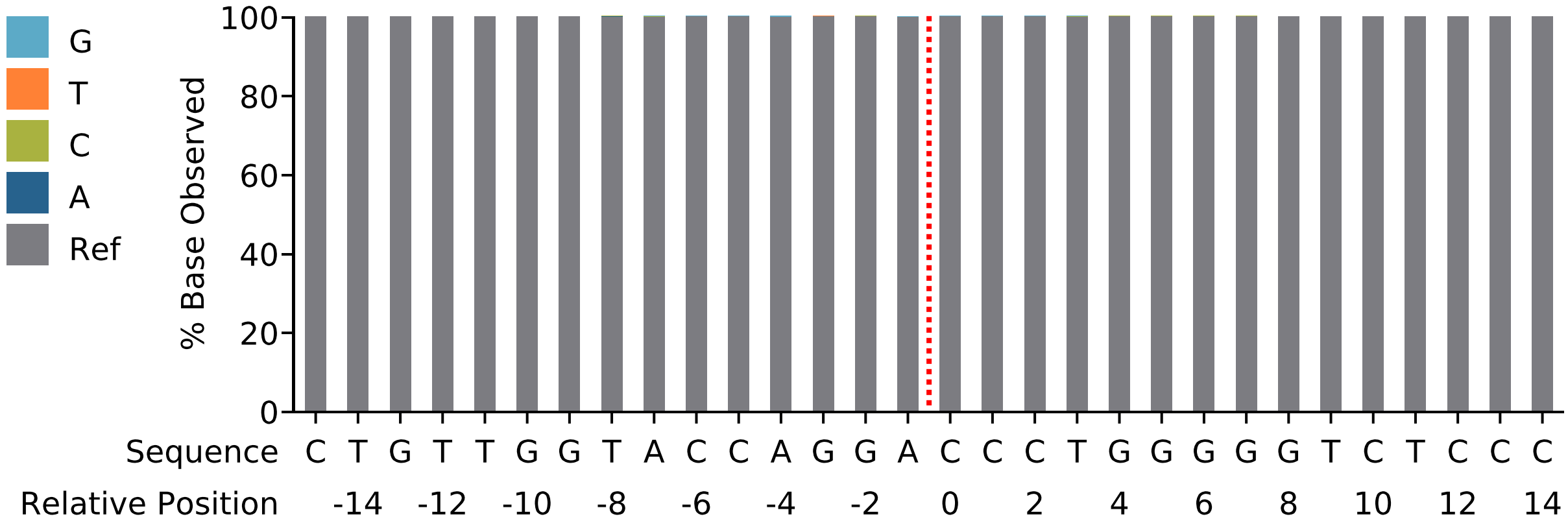

HBV_Cas9_EditR__JAM2

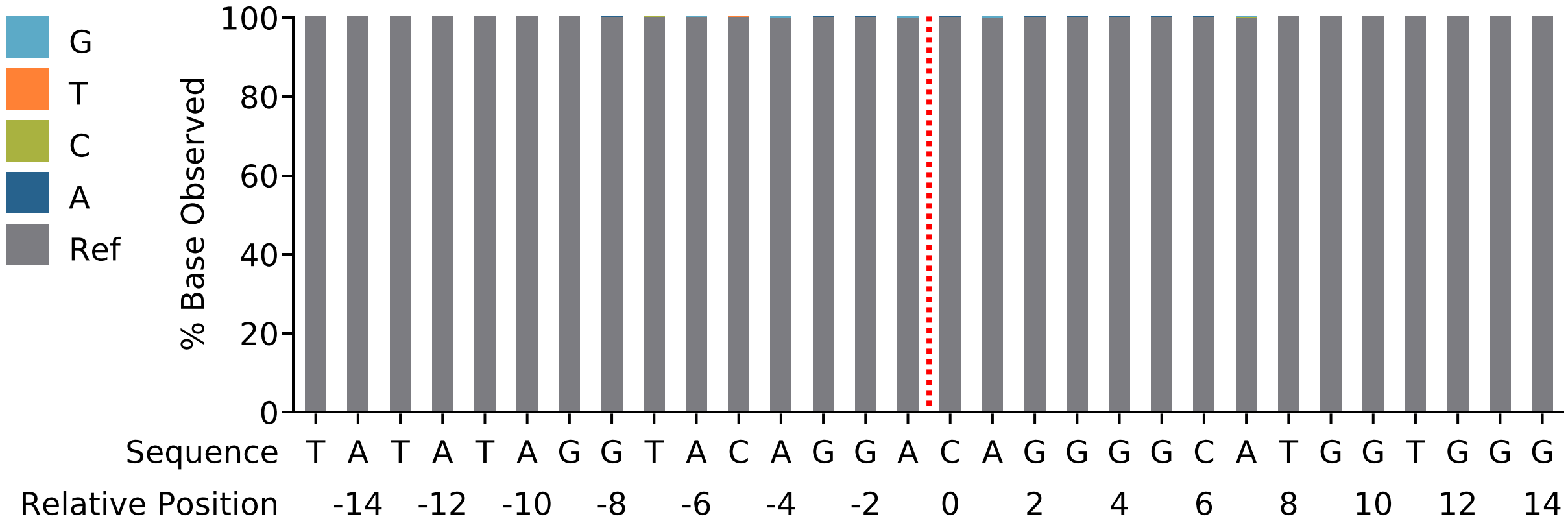

HBV_Cas9_EditR__LHPP

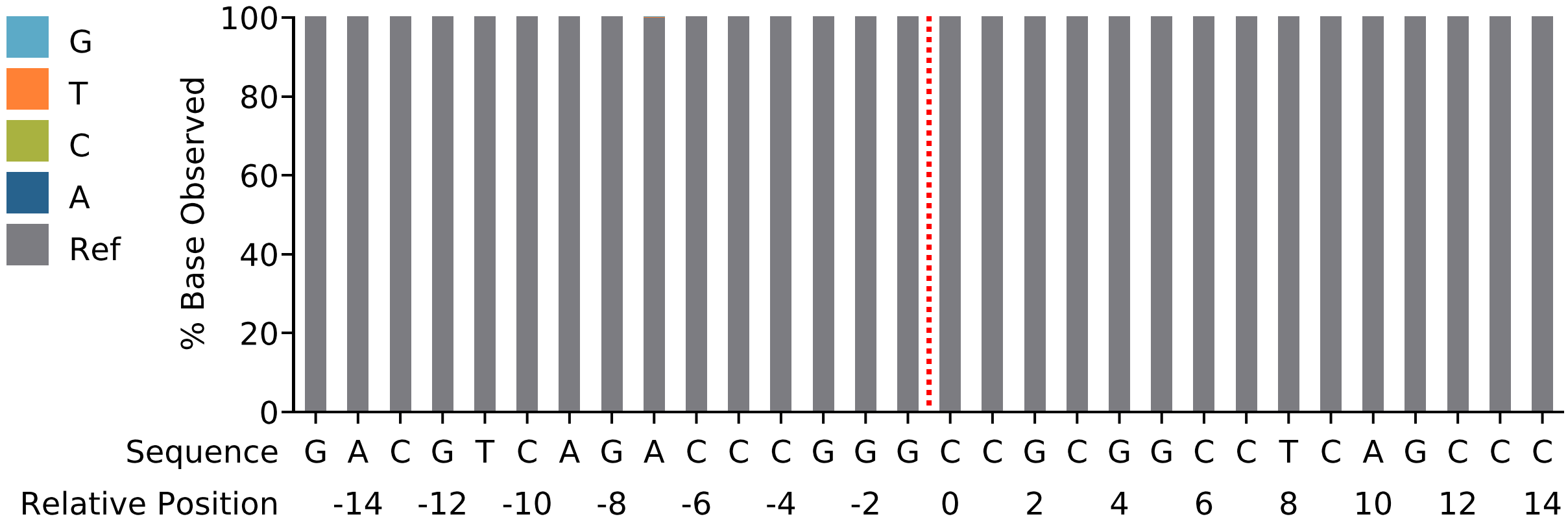

HBV_Cas9_EditR__LINC00301

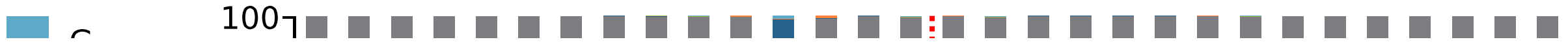

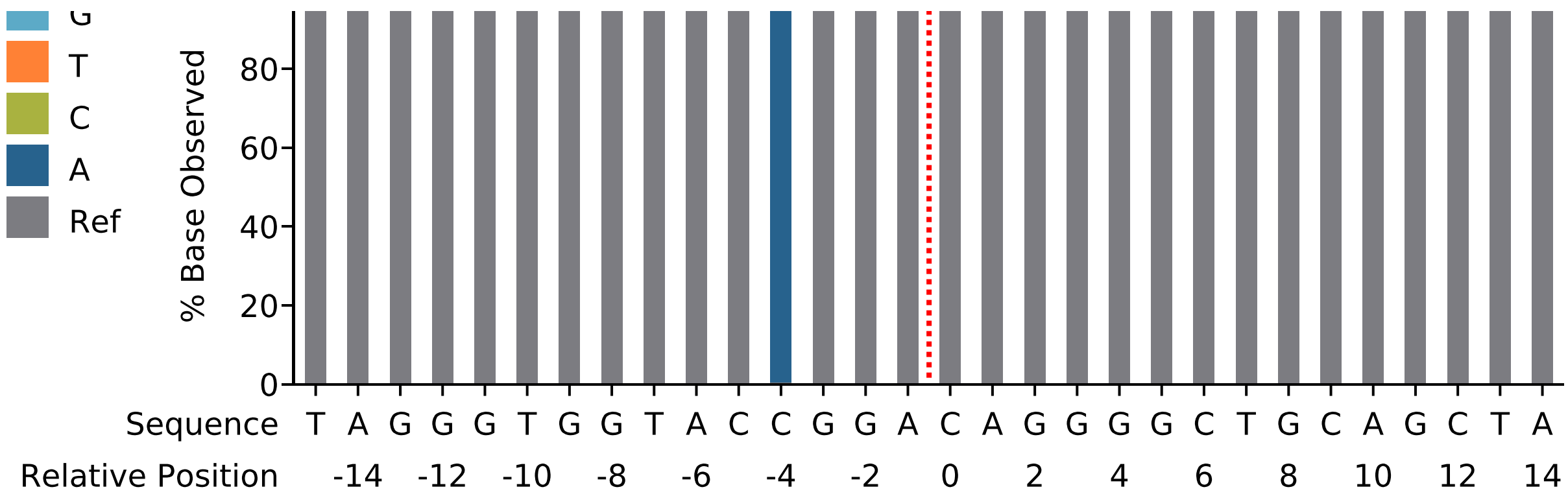

HBV_Cas9_EditR__LOC105372647

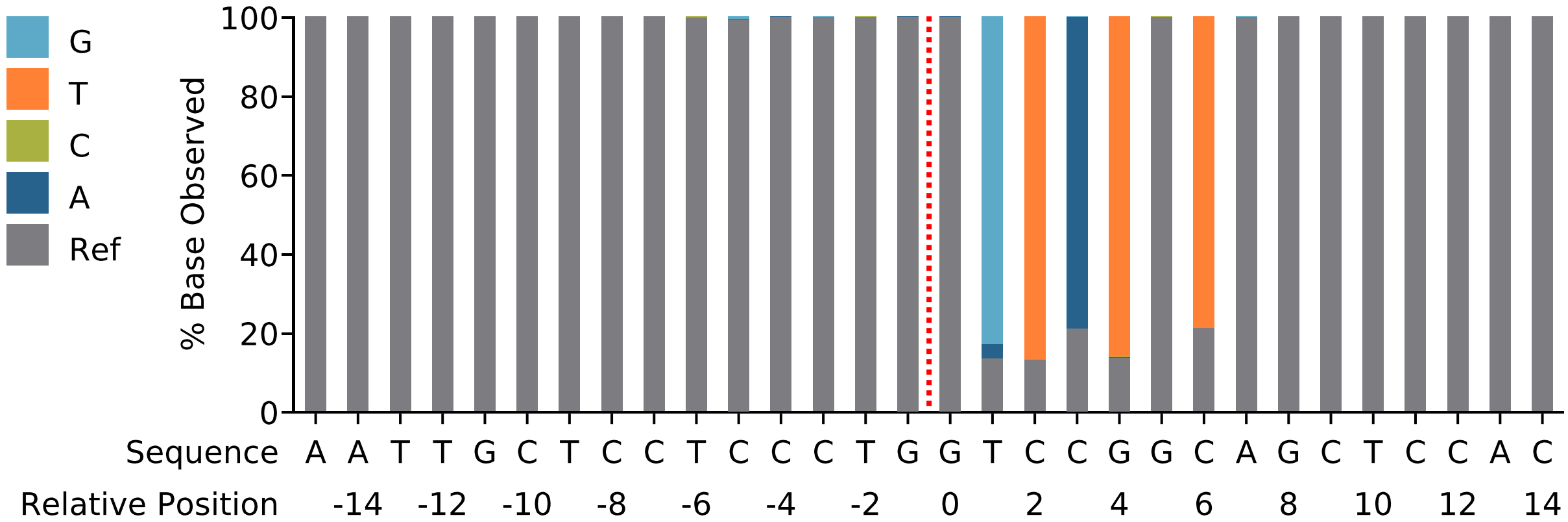

HBV_Cas9_EditR__PRSS55

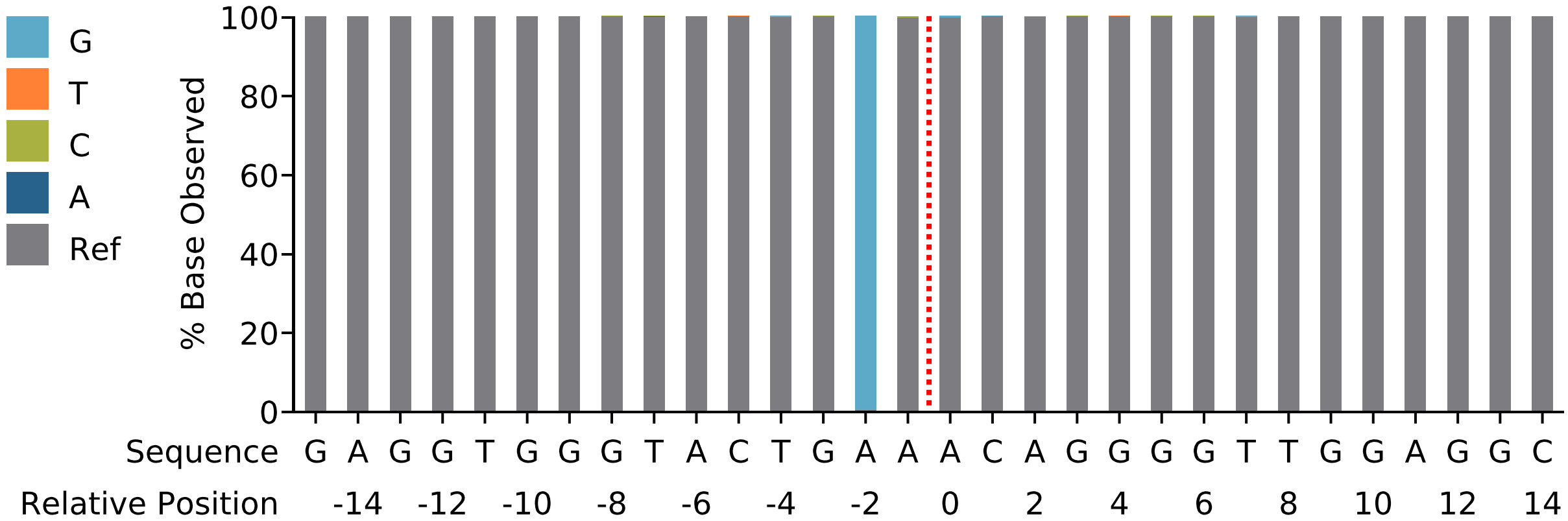

HBV_Cas9_EditR__RPS19BP1

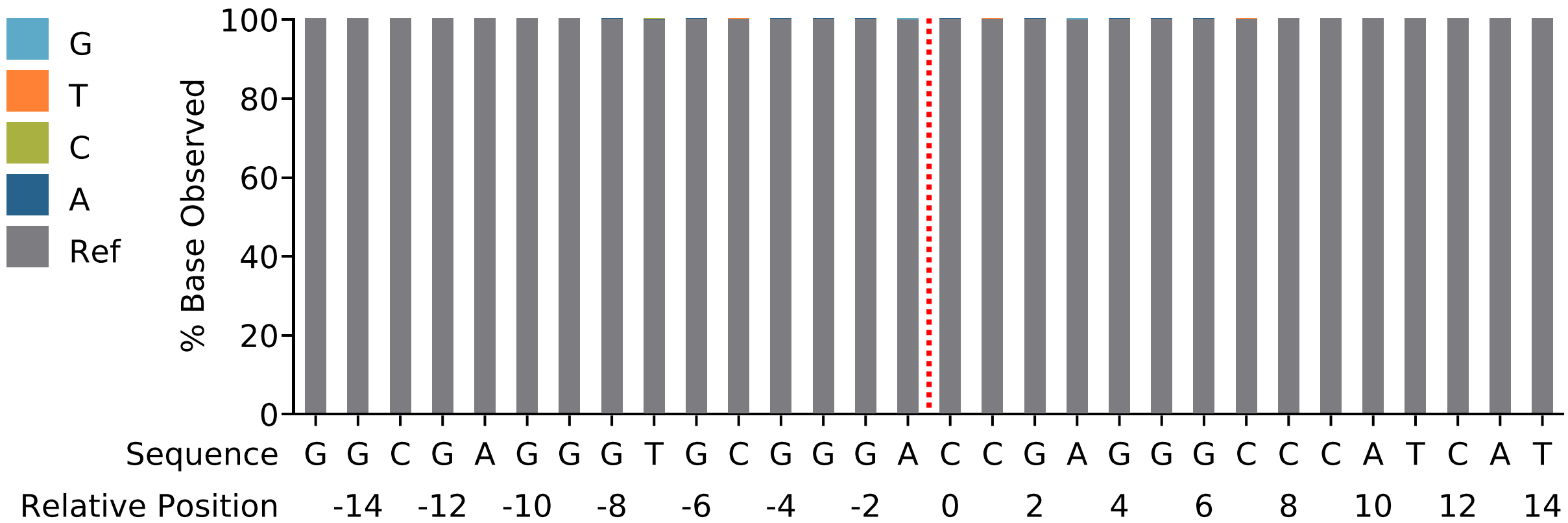

HBV_Cas9_EditR__SUGP1

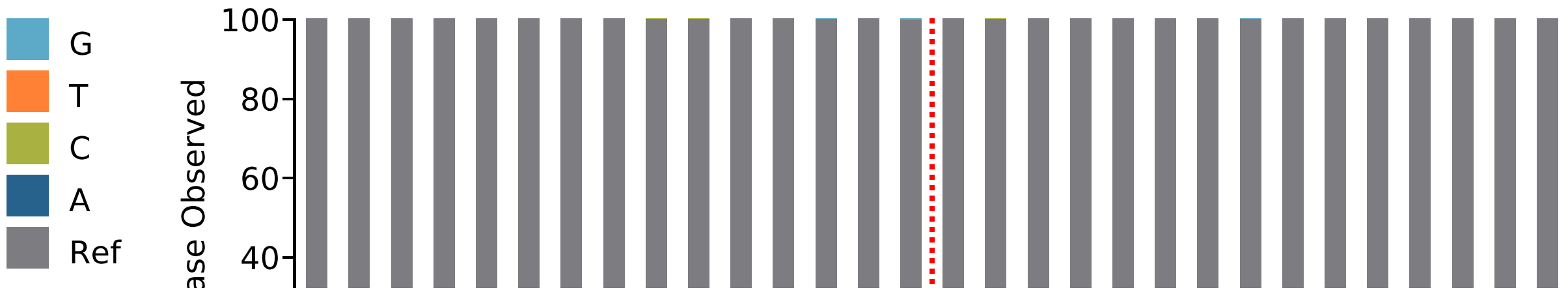

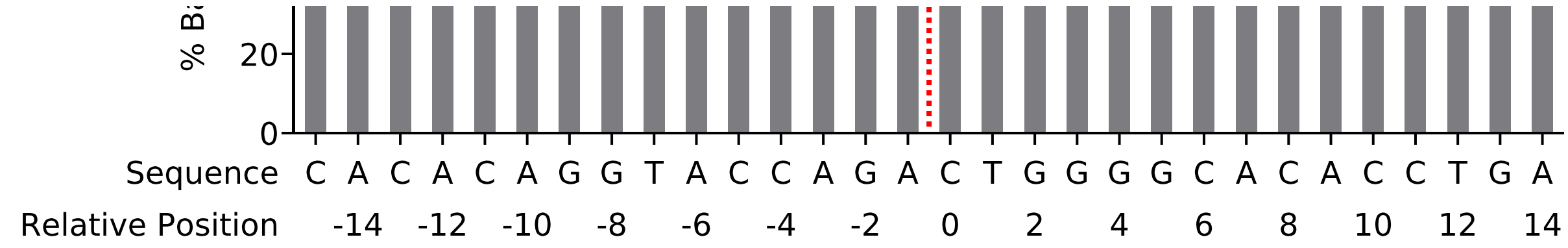

HBV_Cas9_EditR__TBCD

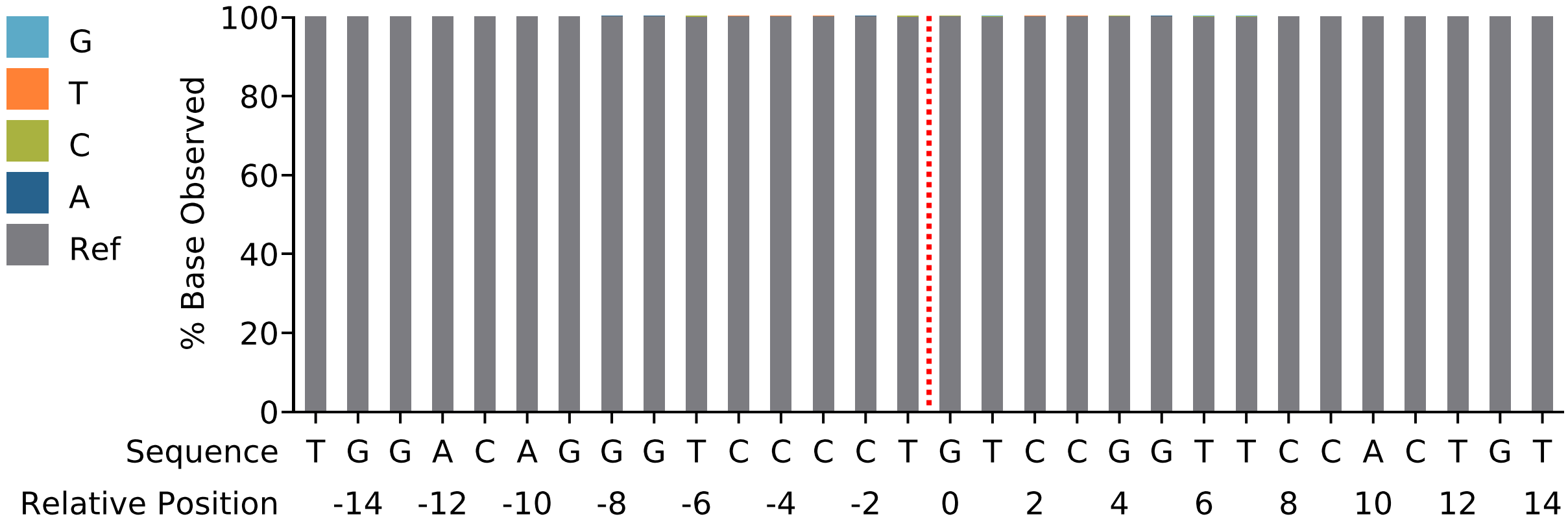

HBV_Cas9_EditR__none24

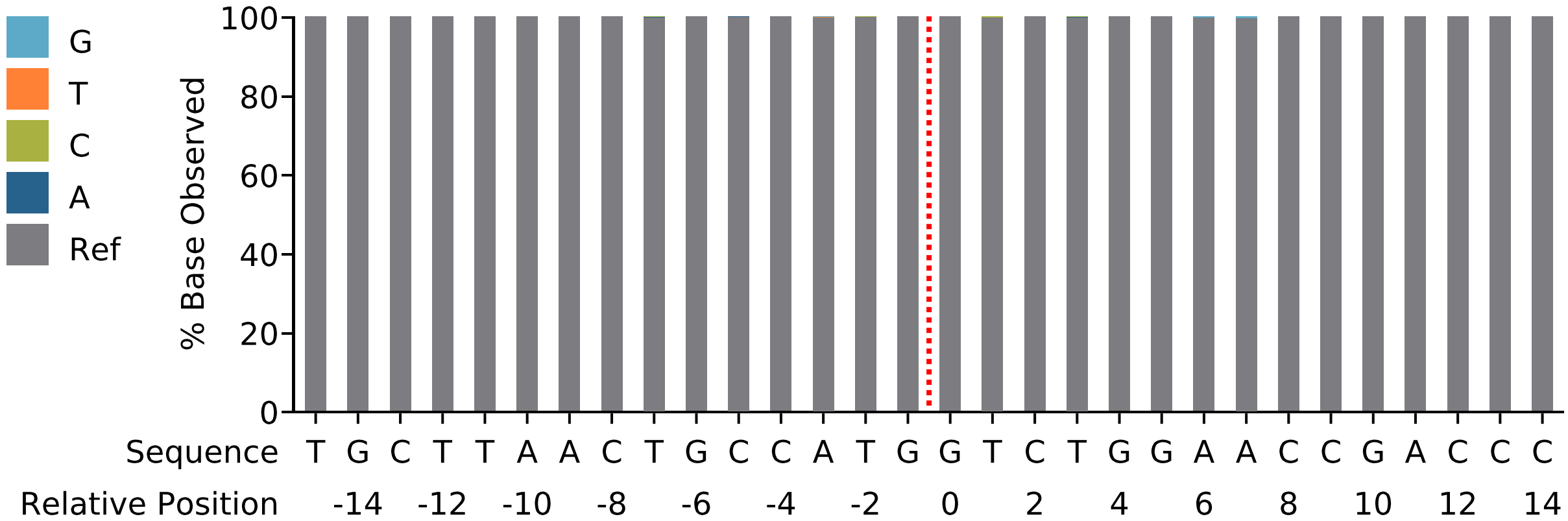

HBV_Cas9_gRNA9__DTNA

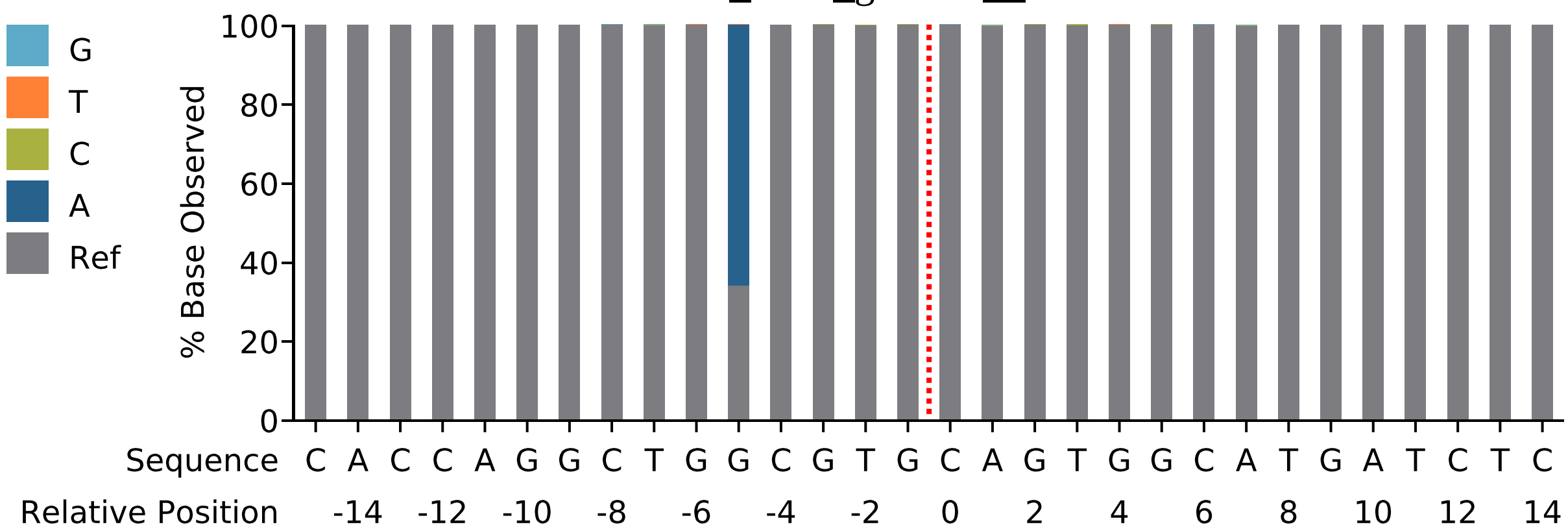

HBV_Cas9_gRNA9__LOC101929268

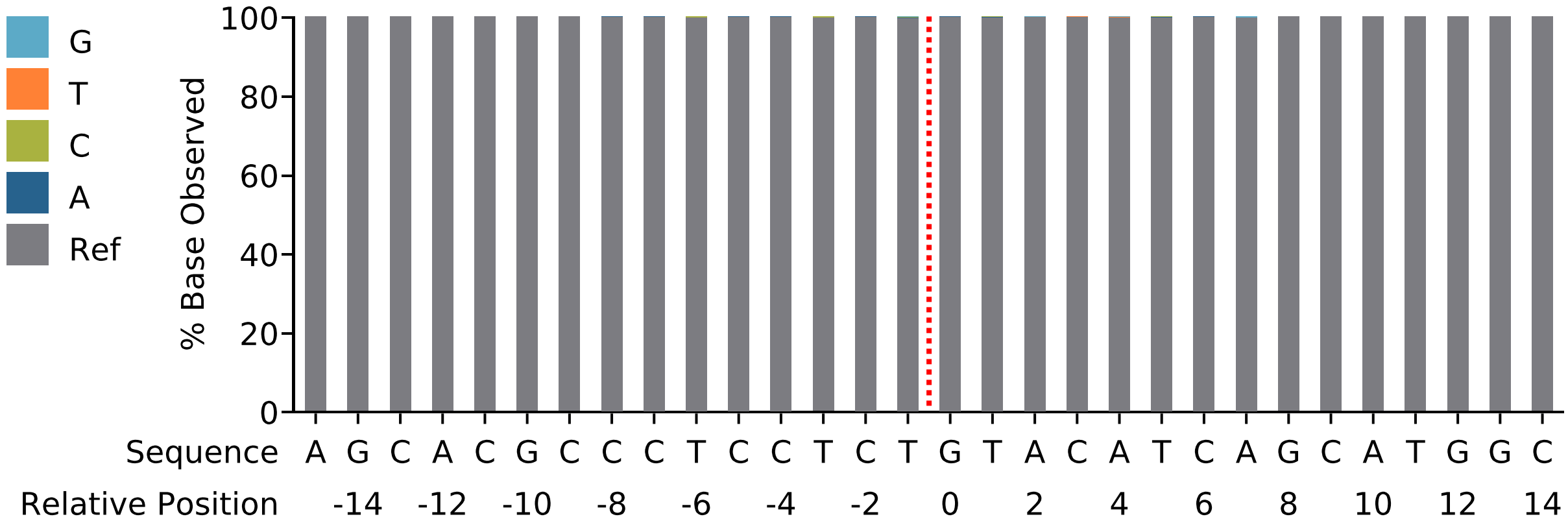

HBV_Cas9_gRNA9__MIR4663

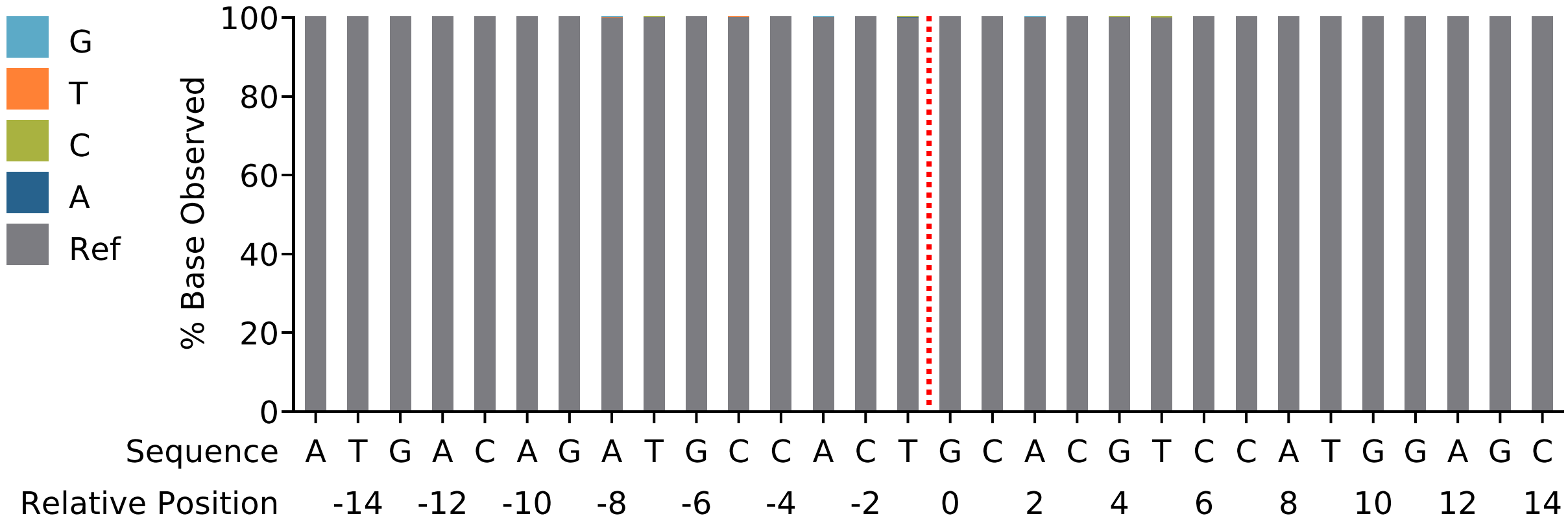

HBV_Cas9_gRNA9__PARP11-AS1

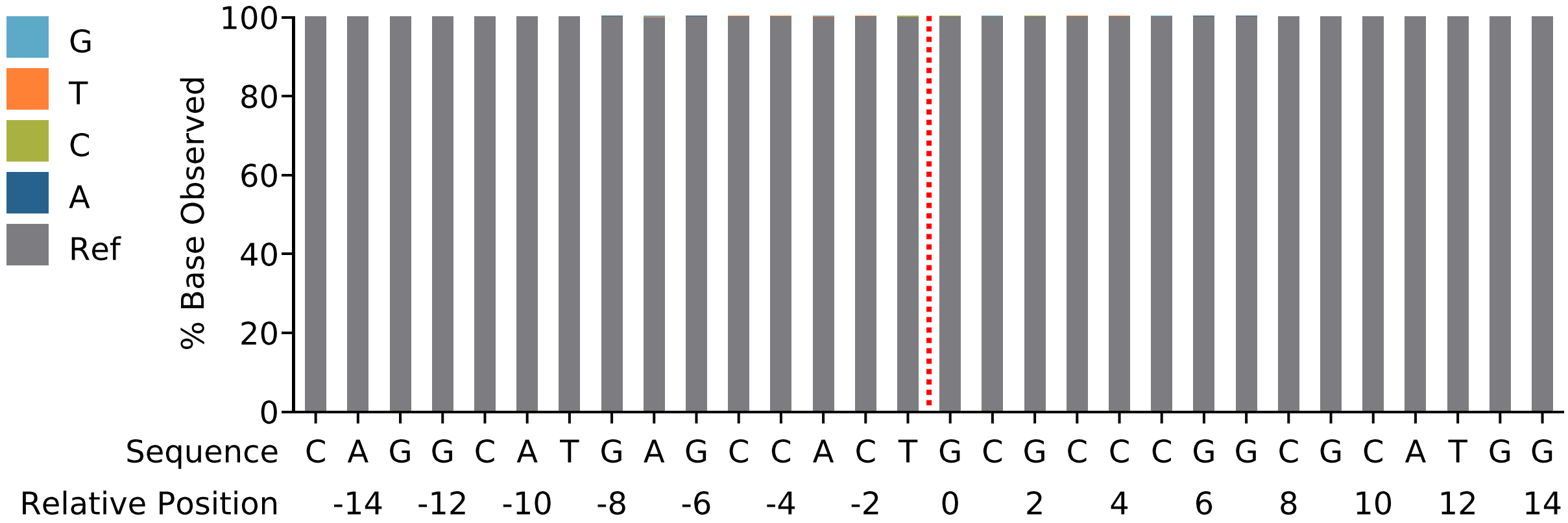

HBV_Cas9_gRNA9__PDE1C

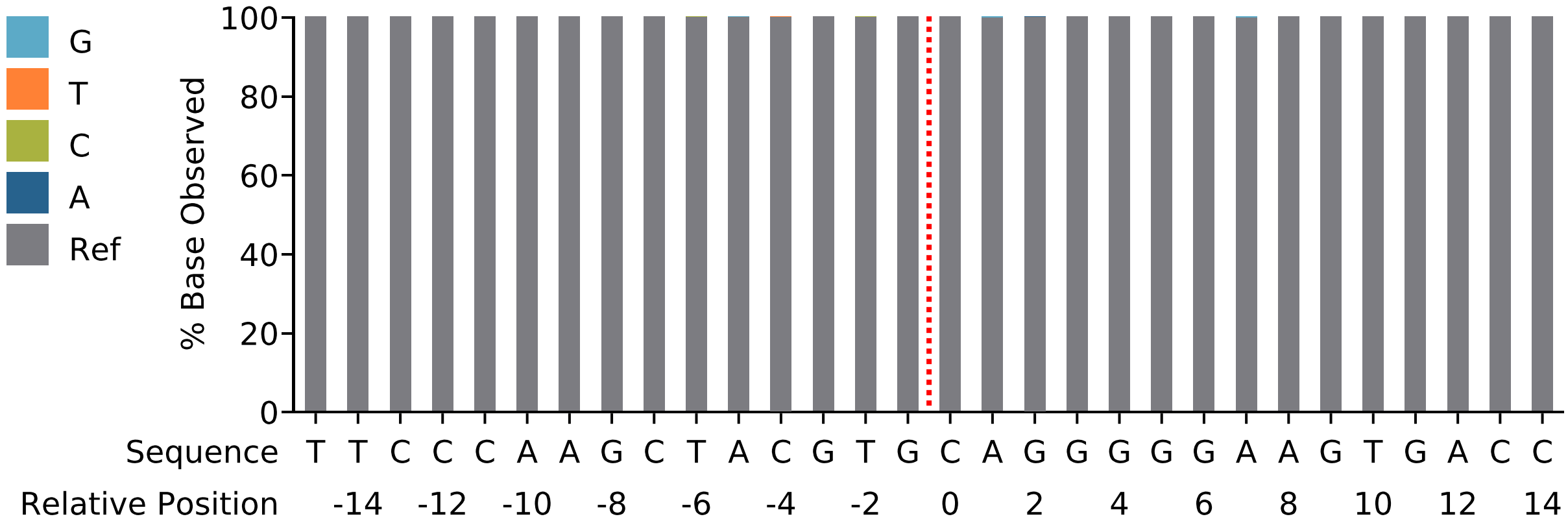

HBV_Cas9_gRNA9__PRRC2B

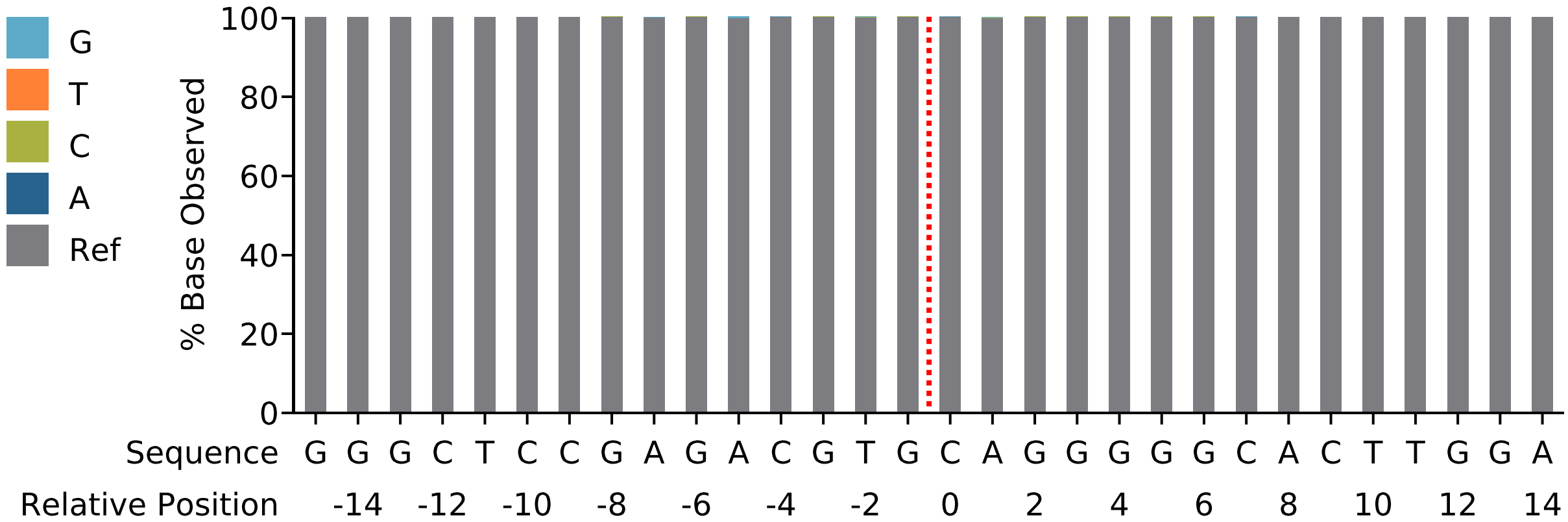

HBV_Cas9_gRNA9__SHISAL1

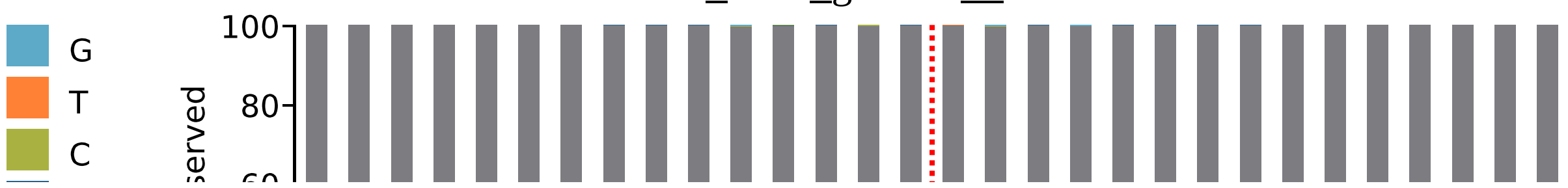

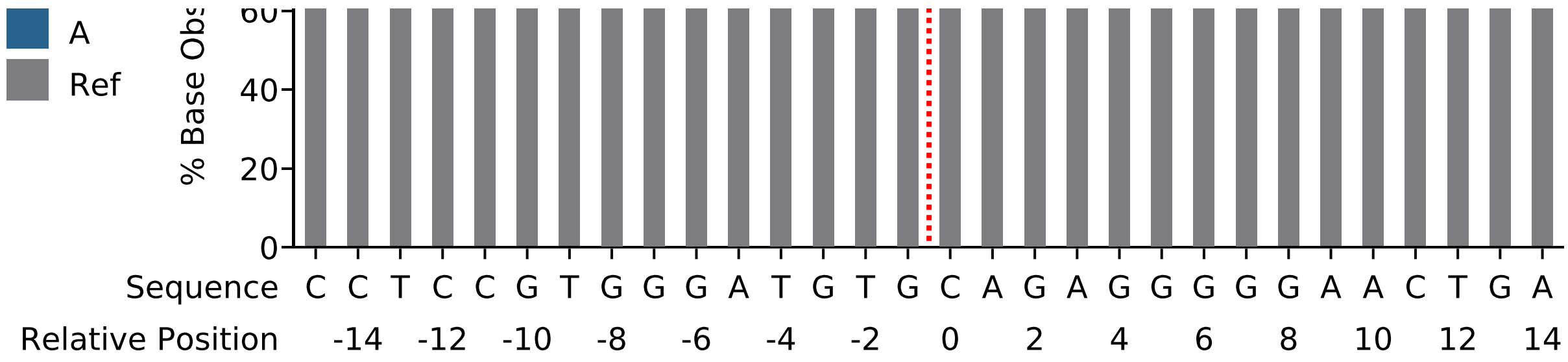

HBV_Cas9_gRNA9__none11

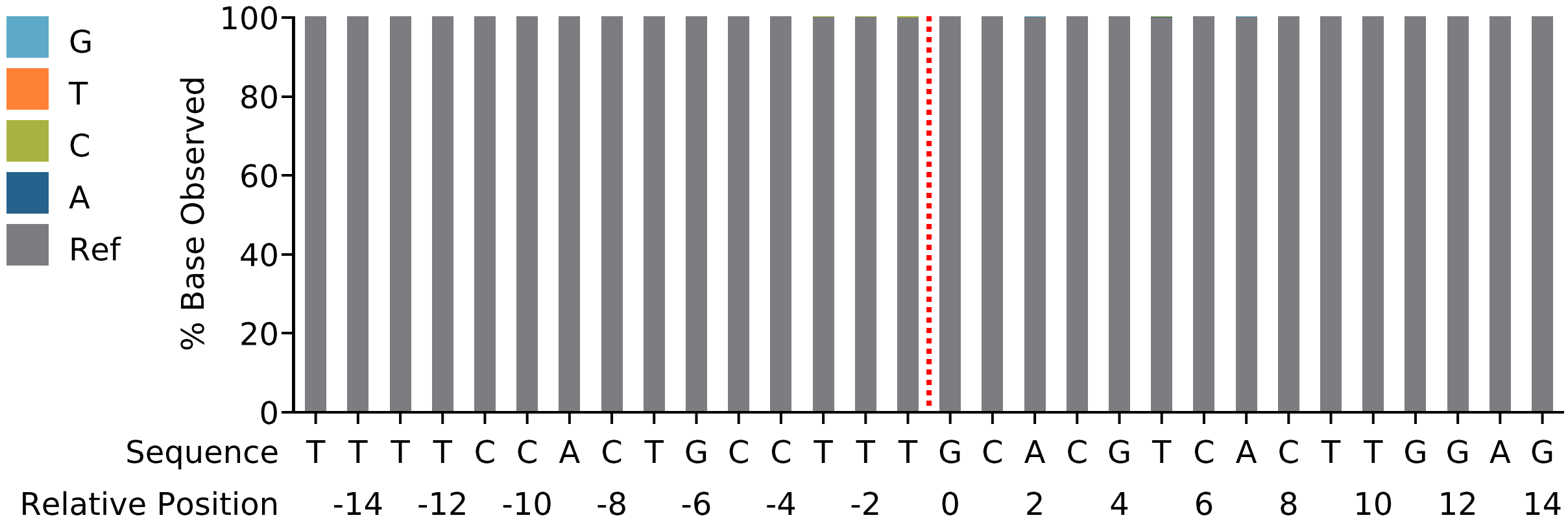

HBV_Cas9_gRNA9__none12

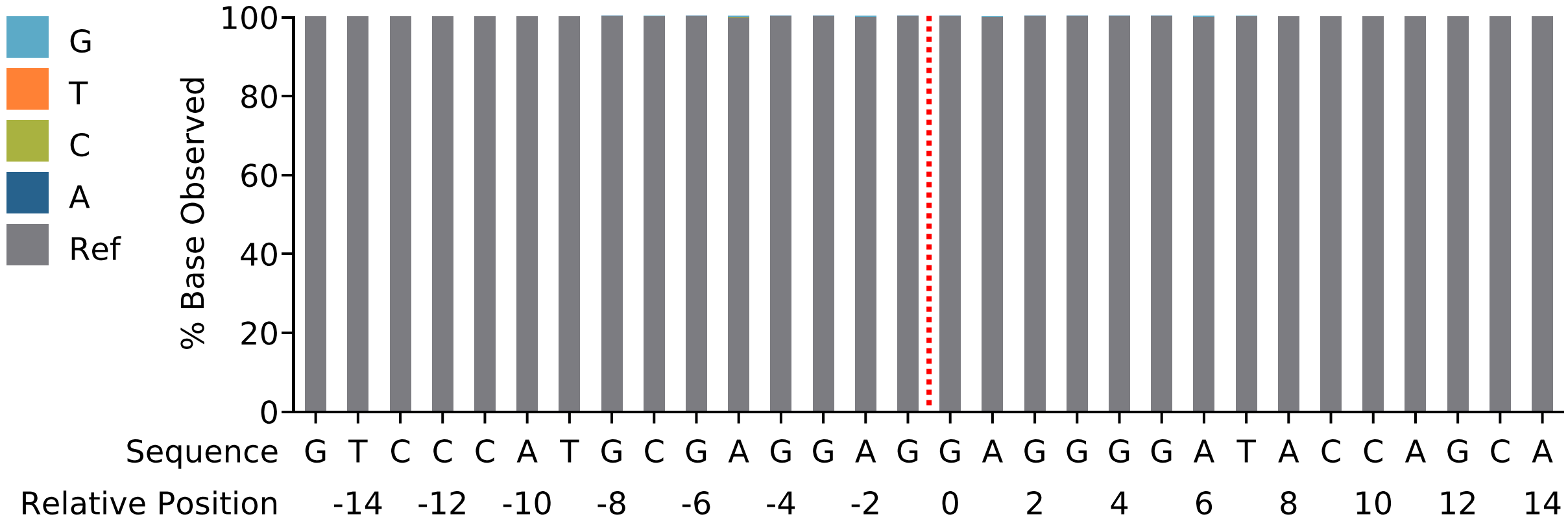

HBV_Cas9_gRNA9__none13

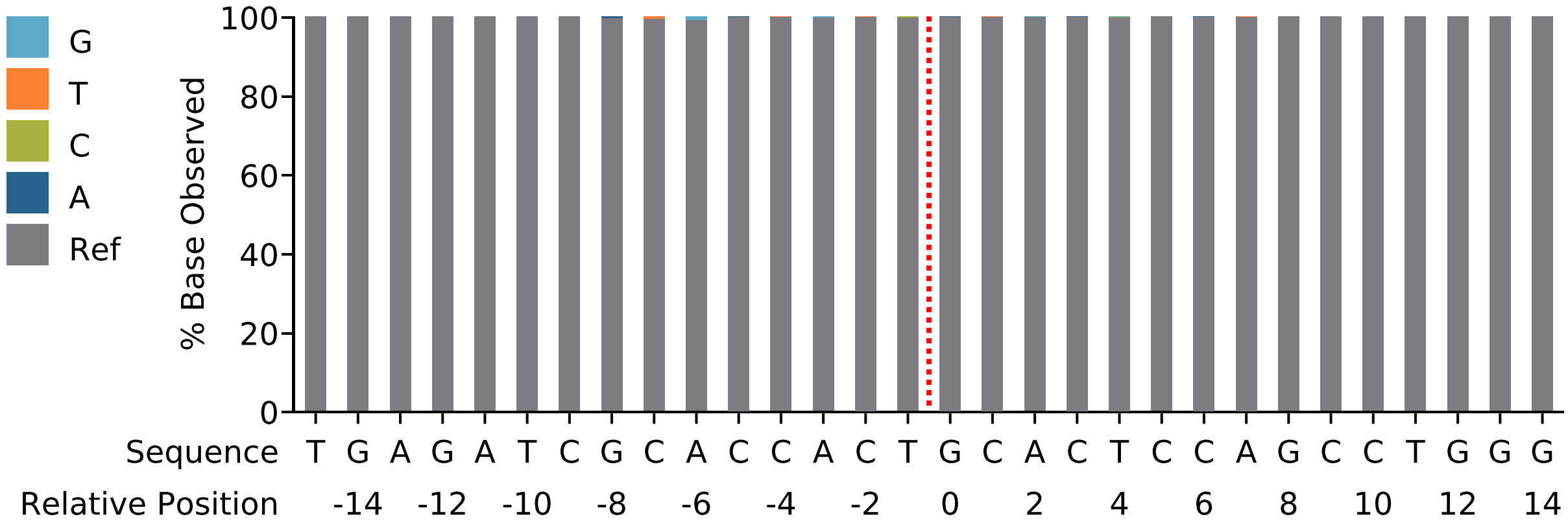

HBV_Cas9_gRNA9__none14

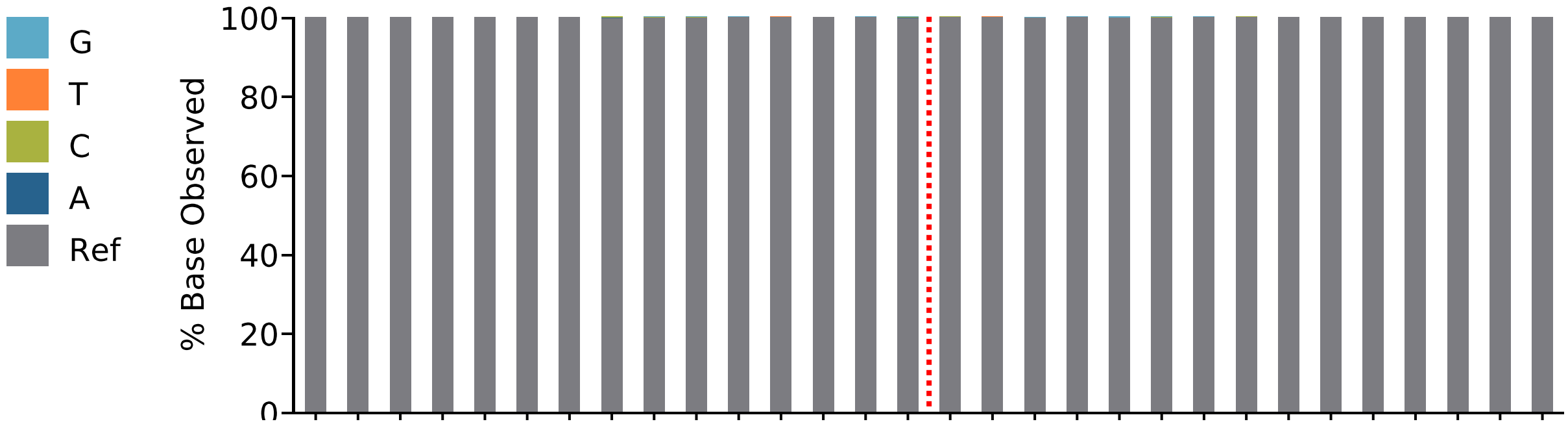

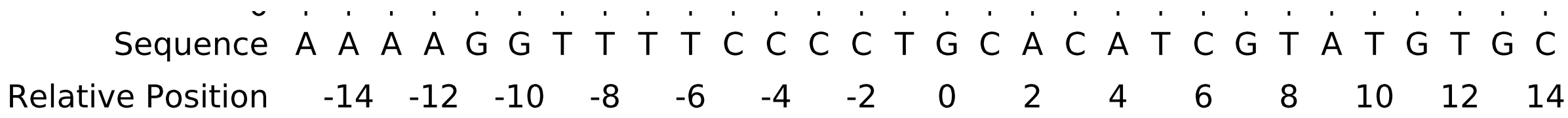

HBV_Cas9_gRNA9__none15

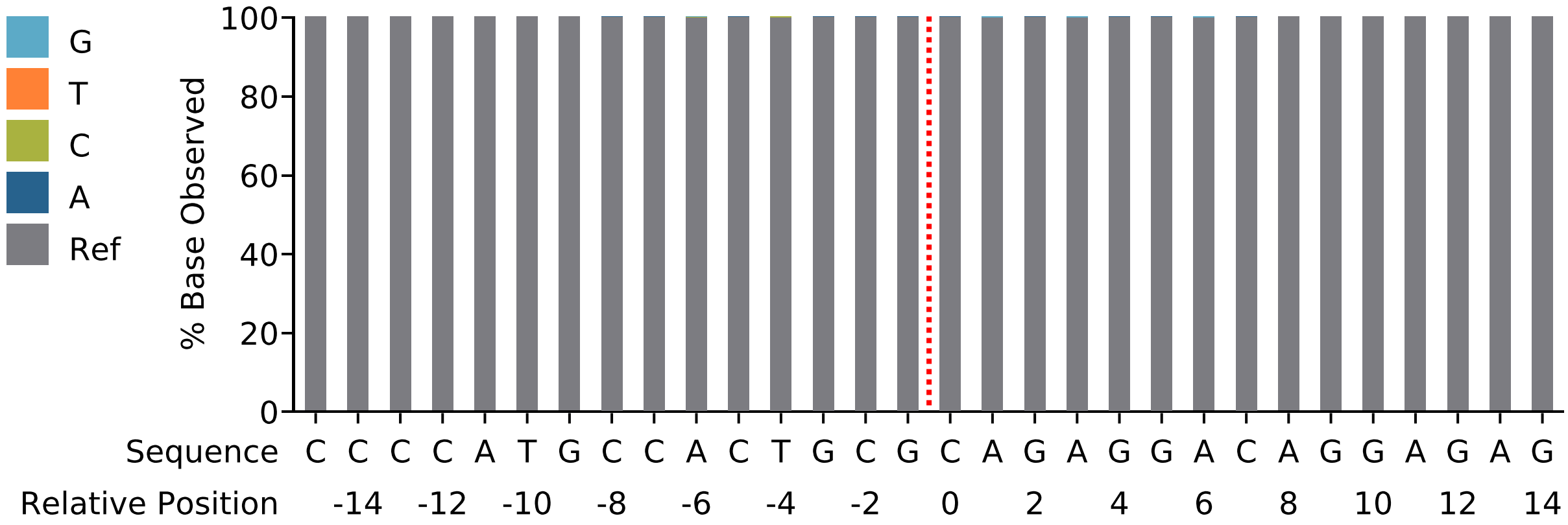

HBV_Cas9_gRNA9__none16

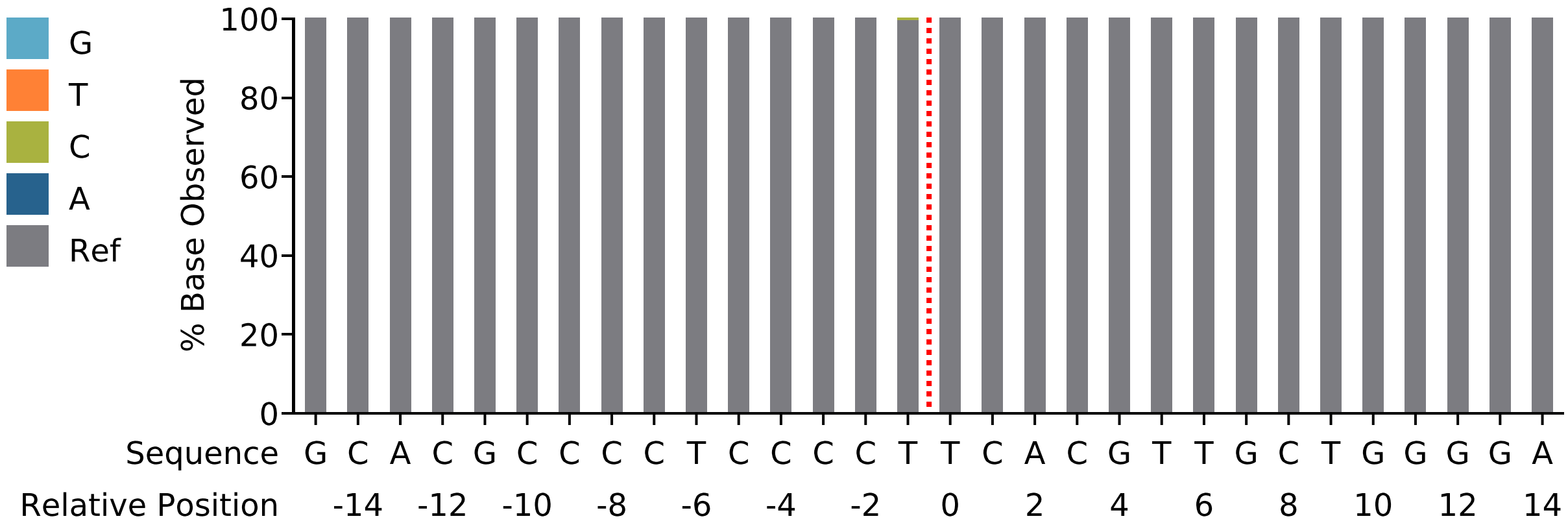

HBV_Cas9_gRNA9__none17

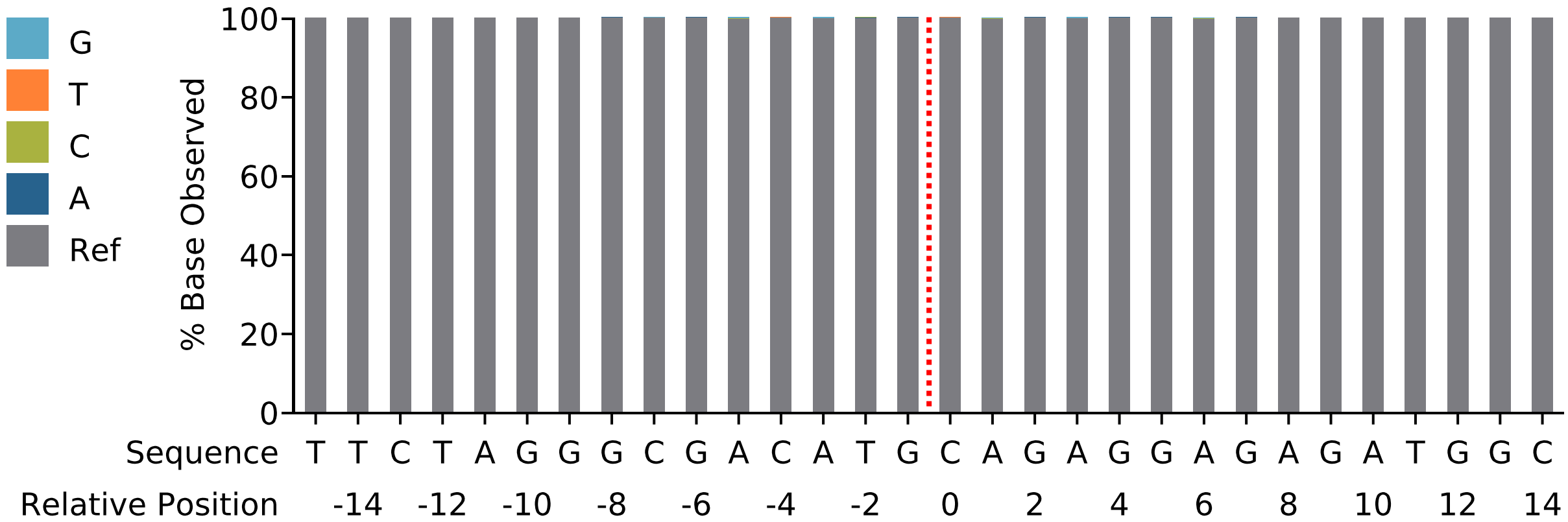

HBV_Cas9_gRNA9__none18

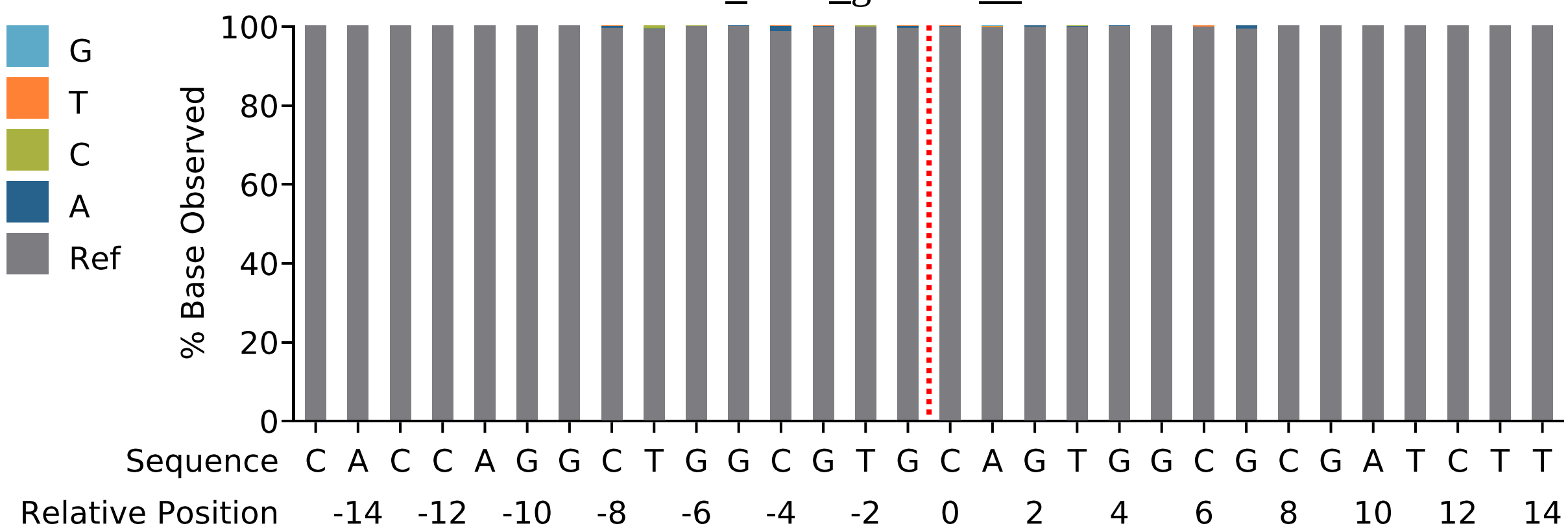

HBV_Cas9_gRNA9__none19

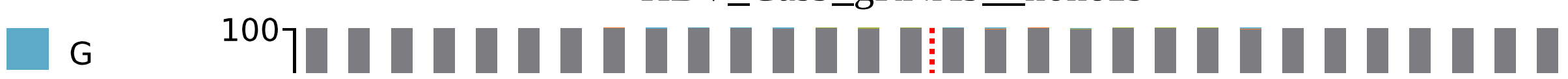

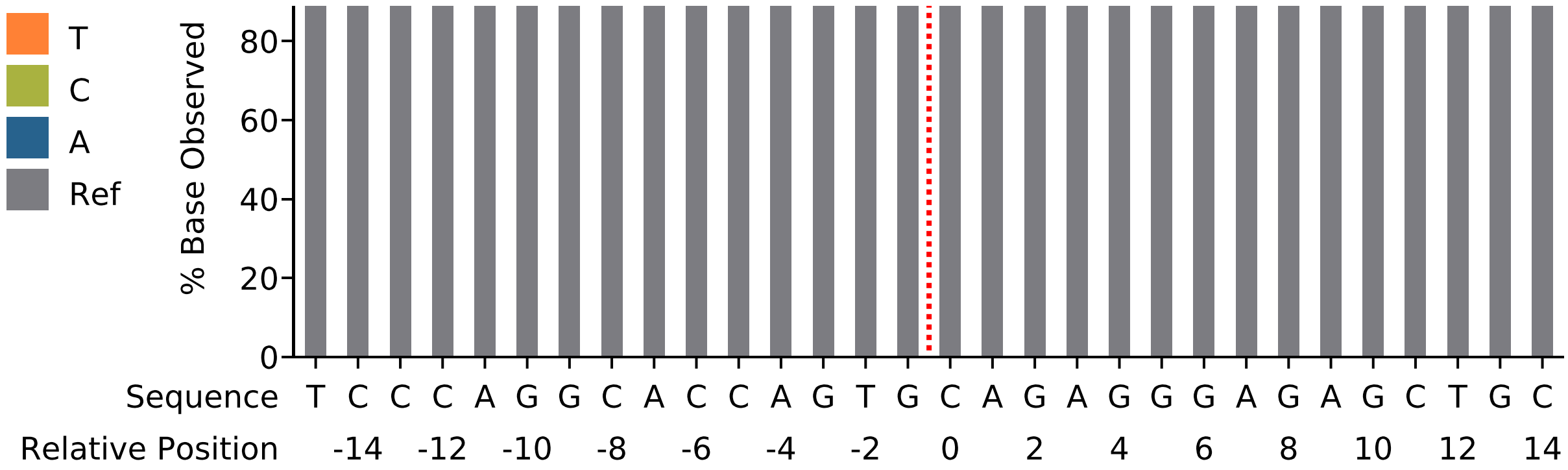

HBV_Cas9_gRNA9__none20

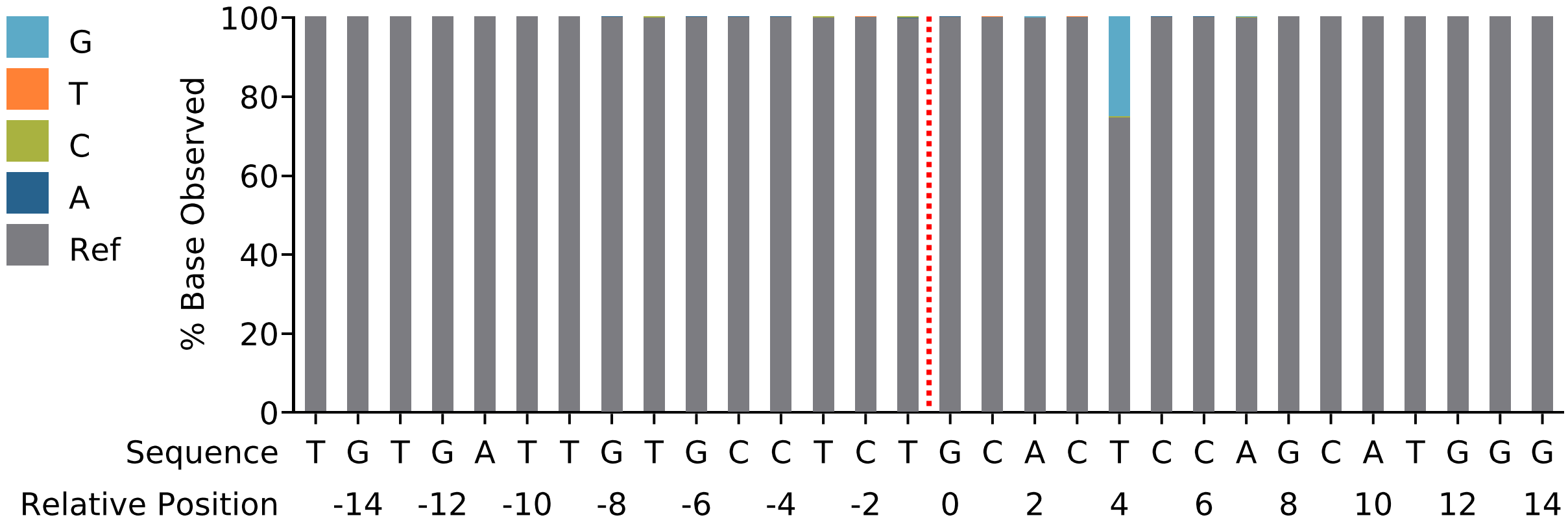

HBV_Cas9_gRNA9__none21

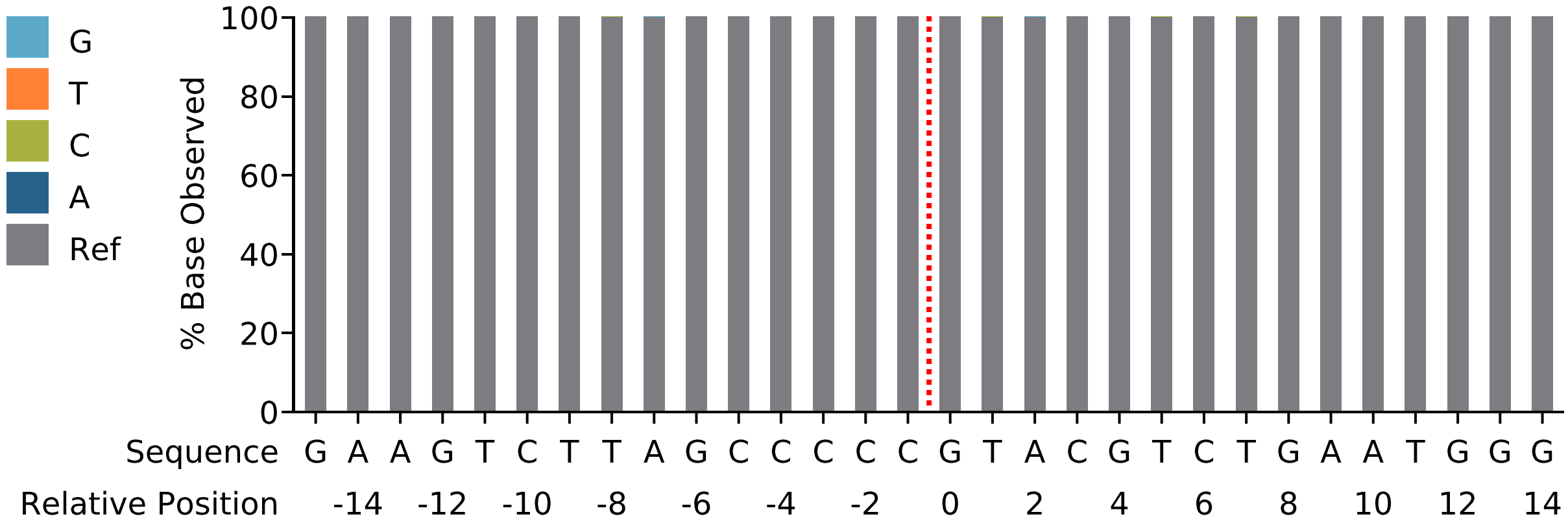

HBV_Cas9_gRNA9__none22

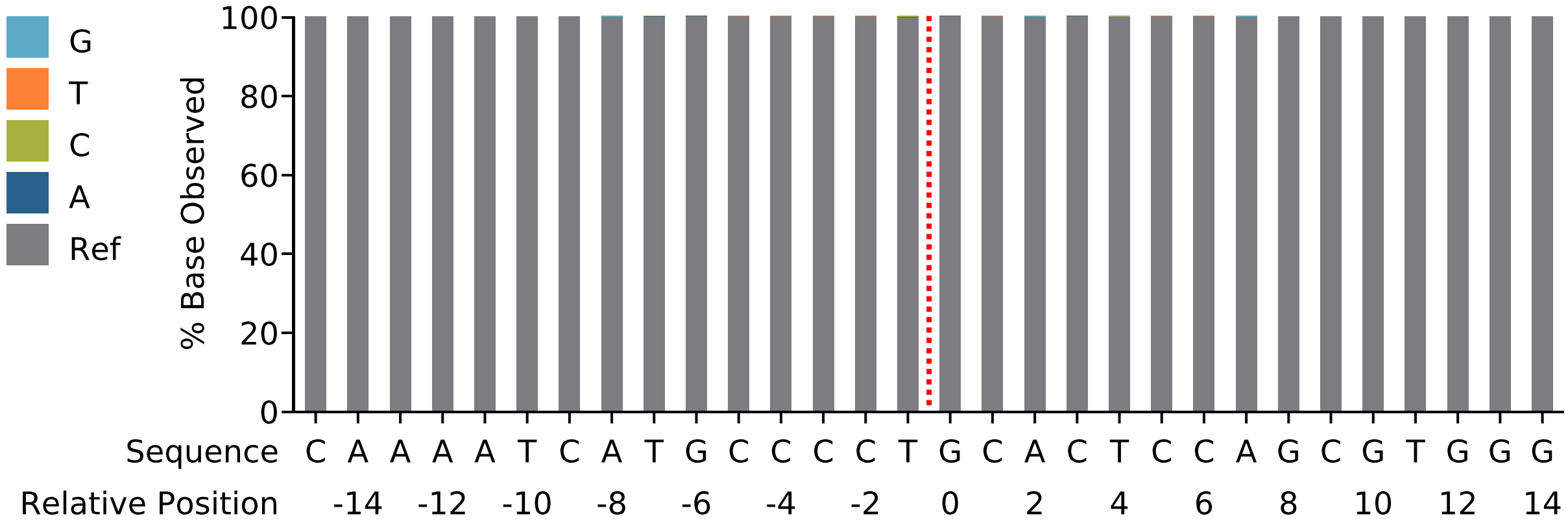

Supplement: Supplementary file 7 [file DataSheet7.pdf]
