# Supplementary material for: Combined RNA- and DNA-targeting CRISPR ribonucleoproteins enhance suppression of HBV transcripts
Source: Front Genome Ed. 2026 Sep 23;8:1824895. doi: 10.3389/fgeed.2026.1824895 (PMC13642003; doi:10.3389/fgeed.2026.1824895)

Analysis mode: CRISPAltRations 1_2_1
Start date: 2026/08/21 15:27
Targets: HBV_Cas12_gRNA4__CD300E, HBV_Cas12_gRNA4__CHKB-DT, HBV_Cas12_gRNA4__GNA12, HBV_Cas12_gRNA4__LINC00989, HBV_Cas12_gRNA4__LOC107986930, HBV_Cas12_gRNA4__MRGBP, HBV_Cas12_gRNA4__PCNX2, HBV_Cas12_gRNA4__SHROOM2, HBV_Cas12_gRNA4__SYNJ2BP, HBV_Cas12_gRNA4__TANK, HBV_Cas12_gRNA4__TCF12, HBV_Cas12_gRNA4__TEKT5, HBV_Cas12_gRNA4__VTI1A, HBV_Cas12_gRNA4__ZBTB44-DT, HBV_Cas12_gRNA4__ZNF292, HBV_Cas12_gRNA4__none52, HBV_Cas12_gRNA4__none54, HBV_Cas12_gRNA4__none55, HBV_Cas12_gRNA4__none56, HBV_Cas12a_Control__CNTN451, HBV_Cas12a_Control__POGLUT3, HBV_Cas12a_Control__ROR1, HBV_Cas12a_Control__SHISA6, HBV_Cas12a_Control__TMC4, HBV_Cas12a_Control__none44, HBV_Cas12a_Control__none46, HBV_Cas12a_Control__none47, HBV_Cas12a_Control__none48, HBV_Cas12a_Control__none49

Metrics

	Group ID	Treated	Total Reads	Percent Edited	Percent Unedited	Percent NHEJ	Percent Perfect HDR	Percent Imperfect HDR	Percent HDR	Percent Other	Percent In Frame	Percent Frameshift	Percent Indels	Percent Insertions	Percent Templated Insertion	Percent GC-Insertion	Percent Deletions
**HBV_Cas12_gRNA4__CD300E**	HBV_Fig4	FALSE	1352	98.67	1.33	0.22	0.0	0.0	0.0	98.45	99.78	0.22	0.22	0.0	0.0	0.0	0.22
**HBV_Cas12_gRNA4__CHKB-DT**	HBV_Fig4	FALSE	17468	3.21	96.79	0.03	0.0	0.0	0.0	3.18	99.97	0.03	0.03	0.0	0.0	0.0	0.03
**HBV_Cas12_gRNA4__GNA12**	HBV_Fig4	FALSE	86631	3.12	96.88	0.09	0.0	0.0	0.0	3.03	99.91	0.09	0.09	0.0	100.0	0.0	0.09
**HBV_Cas12_gRNA4__LINC00989**	HBV_Fig4	FALSE	168646	3.26	96.74	0.07	0.0	0.0	0.0	3.19	99.93	0.07	0.07	0.0	0.0	0.0	0.07
**HBV_Cas12_gRNA4__LOC107986930**	HBV_Fig4	FALSE	92654	4.15	95.85	0.12	0.0	0.0	0.0	4.03	99.88	0.12	0.12	0.0	0.0	0.0	0.12
**HBV_Cas12_gRNA4__MRGBP**	HBV_Fig4	FALSE	42002	43.94	56.06	0.16	0.0	0.0	0.0	43.78	99.93	0.07	0.16	0.0	0.0	0.0	0.16
**HBV_Cas12_gRNA4__PCNX2**	HBV_Fig4	FALSE	444048	3.42	96.58	0.07	0.0	0.0	0.0	3.35	99.93	0.07	0.07	0.0	0.0	0.0	0.07
**HBV_Cas12_gRNA4__SHROOM2**	HBV_Fig4	FALSE	101505	3.25	96.75	0.41	0.0	0.0	0.0	2.84	99.59	0.41	0.41	0.07	95.52	0.0	0.35
**HBV_Cas12_gRNA4__SYNJ2BP**	HBV_Fig4	FALSE	131882	3.28	96.72	0.07	0.0	0.0	0.0	3.21	99.93	0.07	0.07	0.0	0.0	0.0	0.07
**HBV_Cas12_gRNA4__TANK**	HBV_Fig4	FALSE	216523	3.4	96.6	0.17	0.0	0.0	0.0	3.23	99.83	0.17	0.17	0.0	0.0	0.0	0.16
**HBV_Cas12_gRNA4__TCF12**	HBV_Fig4	FALSE	56337	99.97	0.03	99.85	0.0	0.0	0.0	0.12	99.98	0.02	99.85	99.85	0.0	0.22	0.01
**HBV_Cas12_gRNA4__TEKT5**	HBV_Fig4	FALSE	999644	4.25	95.75	0.08	0.0	0.0	0.0	4.17	99.93	0.07	0.08	0.0	0.0	0.0	0.08
**HBV_Cas12_gRNA4__VTI1A**	HBV_Fig4	FALSE	75164	3.61	96.39	0.02	0.0	0.0	0.0	3.59	99.98	0.02	0.02	0.0	0.0	0.0	0.02
**HBV_Cas12_gRNA4__ZBTB44-DT**	HBV_Fig4	FALSE	9633	3.05	96.95	0.13	0.0	0.0	0.0	2.92	99.87	0.13	0.13	0.0	0.0	0.0	0.13
**HBV_Cas12_gRNA4__ZNF292**	HBV_Fig4	FALSE	125938	3.23	96.77	0.09	0.0	0.0	0.0	3.14	99.91	0.09	0.09	0.0	0.0	0.0	0.09
**HBV_Cas12_gRNA4__none52**	HBV_Fig4	FALSE	196095	3.62	96.38	0.02	0.0	0.0	0.0	3.6	99.98	0.02	0.02	0.0	0.0	0.0	0.02
**HBV_Cas12_gRNA4__none54**	HBV_Fig4	FALSE	12592	3.6	96.4	0.04	0.0	0.0	0.0	3.56	99.96	0.04	0.04	0.01	0.0	0.0	0.04
**HBV_Cas12_gRNA4__none55**	HBV_Fig4	FALSE	117302	99.89	0.11	0.12	0.0	0.0	0.0	99.77	99.88	0.12	0.12	0.0	100.0	0.0	0.12
**HBV_Cas12_gRNA4__none56**	HBV_Fig4	FALSE	157571	3.62	96.38	0.08	0.0	0.0	0.0	3.54	99.92	0.08	0.08	0.0	0.0	0.0	0.08
**HBV_Cas12a_Control__CNTN451**	HBV_Fig4	FALSE	135237	3.75	96.25	0.06	0.0	0.0	0.0	3.69	99.94	0.06	0.06	0.0	0.0	0.0	0.05
**HBV_Cas12a_Control__POGLUT3**	HBV_Fig4	FALSE	423256	36.65	63.35	0.26	0.0	0.0	0.0	36.39	99.74	0.26	0.26	0.01	97.87	0.0	0.25
**HBV_Cas12a_Control__ROR1**	HBV_Fig4	FALSE	38994	6.04	93.96	1.87	0.0	0.0	0.0	4.17	98.42	1.58	1.87	0.76	0.0	0.34	1.13
**HBV_Cas12a_Control__SHISA6**	HBV_Fig4	FALSE	177133	2.98	97.02	0.24	0.0	0.0	0.0	2.74	99.77	0.23	0.24	0.01	22.22	0.0	0.23
**HBV_Cas12a_Control__TMC4**	HBV_Fig4	FALSE	27790	99.99	0.01	99.99	0.0	0.0	0.0	0.0	97.18	2.82	99.99	99.98	0.0	0.0	0.02
**HBV_Cas12a_Control__none44**	HBV_Fig4	FALSE	1074	94.6	5.4	82.22	0.0	0.0	0.0	12.38	38.18	61.82	82.22	19.74	0.0	32.08	62.48
**HBV_Cas12a_Control__none46**	HBV_Fig4	FALSE	871104	53.6	46.4	0.08	0.0	0.0	0.0	53.52	99.92	0.08	0.08	0.01	90.8	2.3	0.07
**HBV_Cas12a_Control__none47**	HBV_Fig4	FALSE	75448	9.22	90.78	0.08	0.0	0.0	0.0	9.14	99.97	0.03	0.08	0.0	100.0	0.0	0.08
**HBV_Cas12a_Control__none48**	HBV_Fig4	FALSE	329977	4.01	95.99	0.08	0.0	0.0	0.0	3.93	99.92	0.08	0.08	0.0	0.0	0.0	0.08
**HBV_Cas12a_Control__none49**	HBV_Fig4	FALSE	140932	100.0	0.0	97.76	0.0	0.0	0.0	2.24	19.32	80.68	97.76	46.03	0.0	21.42	51.73

Percentages

	% Edited	*% Repair Pathways*	*% Frameshift*
HBV_Cas12_gRNA4__CD300E	98.67%	98.45%	99.78%
HBV_Cas12_gRNA4__CHKB-DT	96.79%	96.79%	99.97%
HBV_Cas12_gRNA4__GNA12	96.88%	96.88%	99.91%
HBV_Cas12_gRNA4__LINC00989	96.74%	96.74%	99.93%
HBV_Cas12_gRNA4__LOC107986930	95.85%	95.85%	99.88%
HBV_Cas12_gRNA4__MRGBP	56.06% 43.94%	56.06% 43.78%	99.93%
HBV_Cas12_gRNA4__PCNX2	96.58%	96.58%	99.93%
HBV_Cas12_gRNA4__SHROOM2	96.75%	96.75%	99.59%
HBV_Cas12_gRNA4__SYNJ2BP	96.72%	96.72%	99.93%
HBV_Cas12_gRNA4__TANK	96.6%	96.6%	99.83%
HBV_Cas12_gRNA4__TCF12	99.97%	99.85%	99.98%
HBV_Cas12_gRNA4__TEKT5	95.75%	95.75%	99.93%
HBV_Cas12_gRNA4__VTI1A	96.39%	96.39%	99.98%
HBV_Cas12_gRNA4__ZBTB44-DT	96.95%	96.95%	99.87%
HBV_Cas12_gRNA4__ZNF292	96.77%	96.77%	99.91%
HBV_Cas12_gRNA4__none52	96.38%	96.38%	99.98%
HBV_Cas12_gRNA4__none54	96.4%	96.4%	99.96%
HBV_Cas12_gRNA4__none55	99.89%	99.77%	99.88%
HBV_Cas12_gRNA4__none56	96.38%	96.38%	99.92%
HBV_Cas12a_Control__CNTN451	96.25%	96.25%	99.94%
HBV_Cas12a_Control__POGLUT3	63.35% 36.65%	63.35% 36.39%	99.74%
HBV_Cas12a_Control__ROR1	93.96%	93.96%	98.42%
HBV_Cas12a_Control__SHISA6	97.02%	97.02%	99.77%
HBV_Cas12a_Control__TMC4	99.99%	99.99%	97.18%
HBV_Cas12a_Control__none44	94.6%	82.22% 12.38	38.18% 61.82%
HBV_Cas12a_Control__none46	46.4% 53.6%	46.4% 53.52%	99.92%
HBV_Cas12a_Control__none47	90.78%	90.78%	99.97%
HBV_Cas12a_Control__none48	95.99%	95.99%	99.92%

HBV_Cas12a_Control__none49

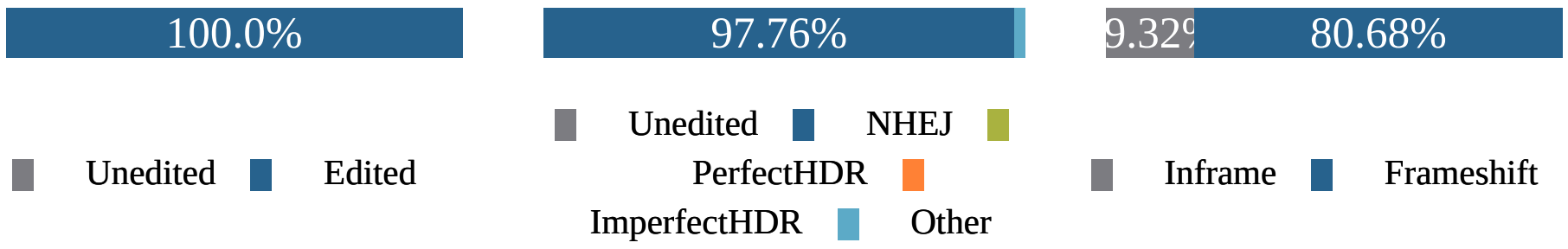

Insertions

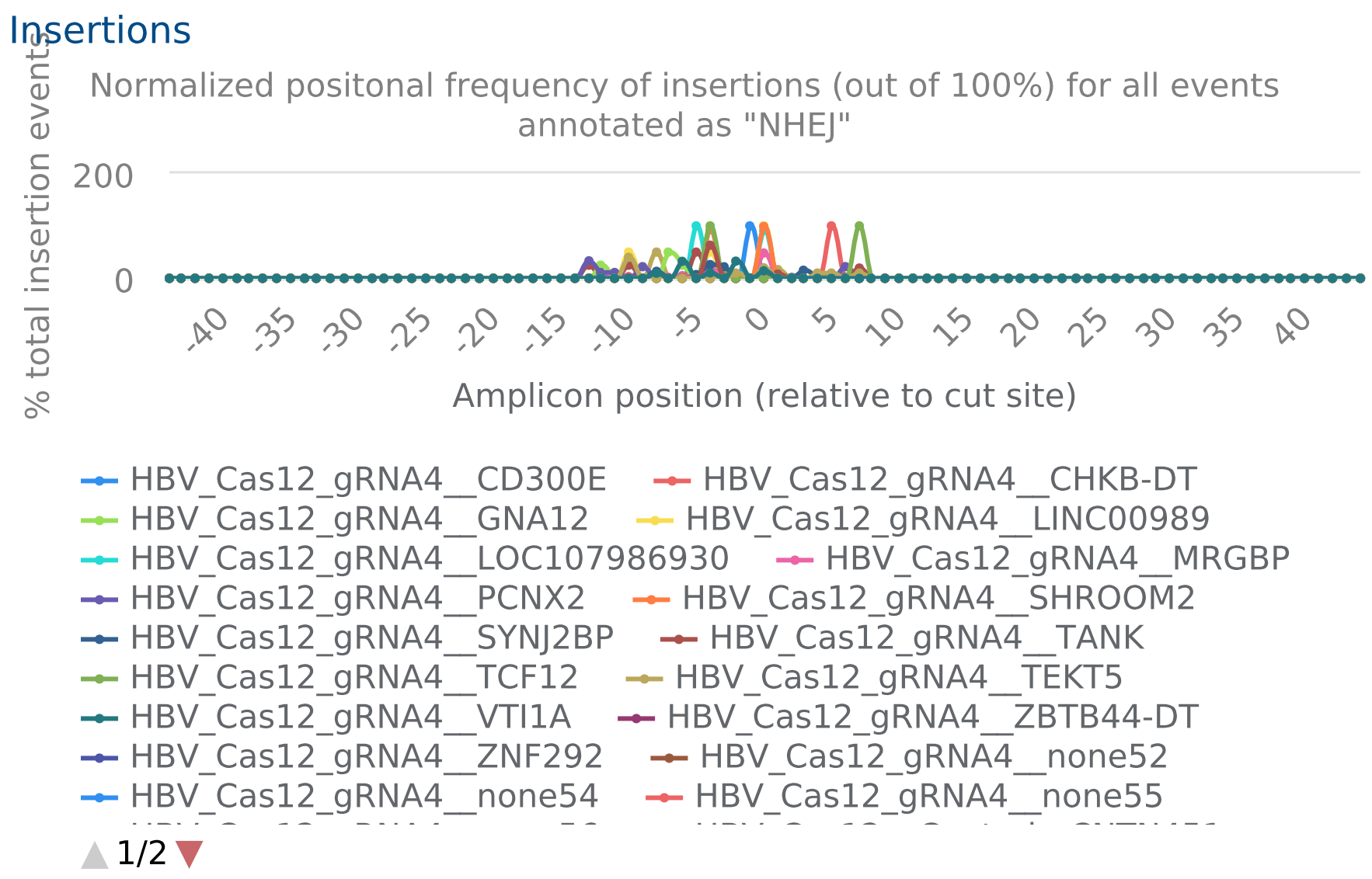

Deletions

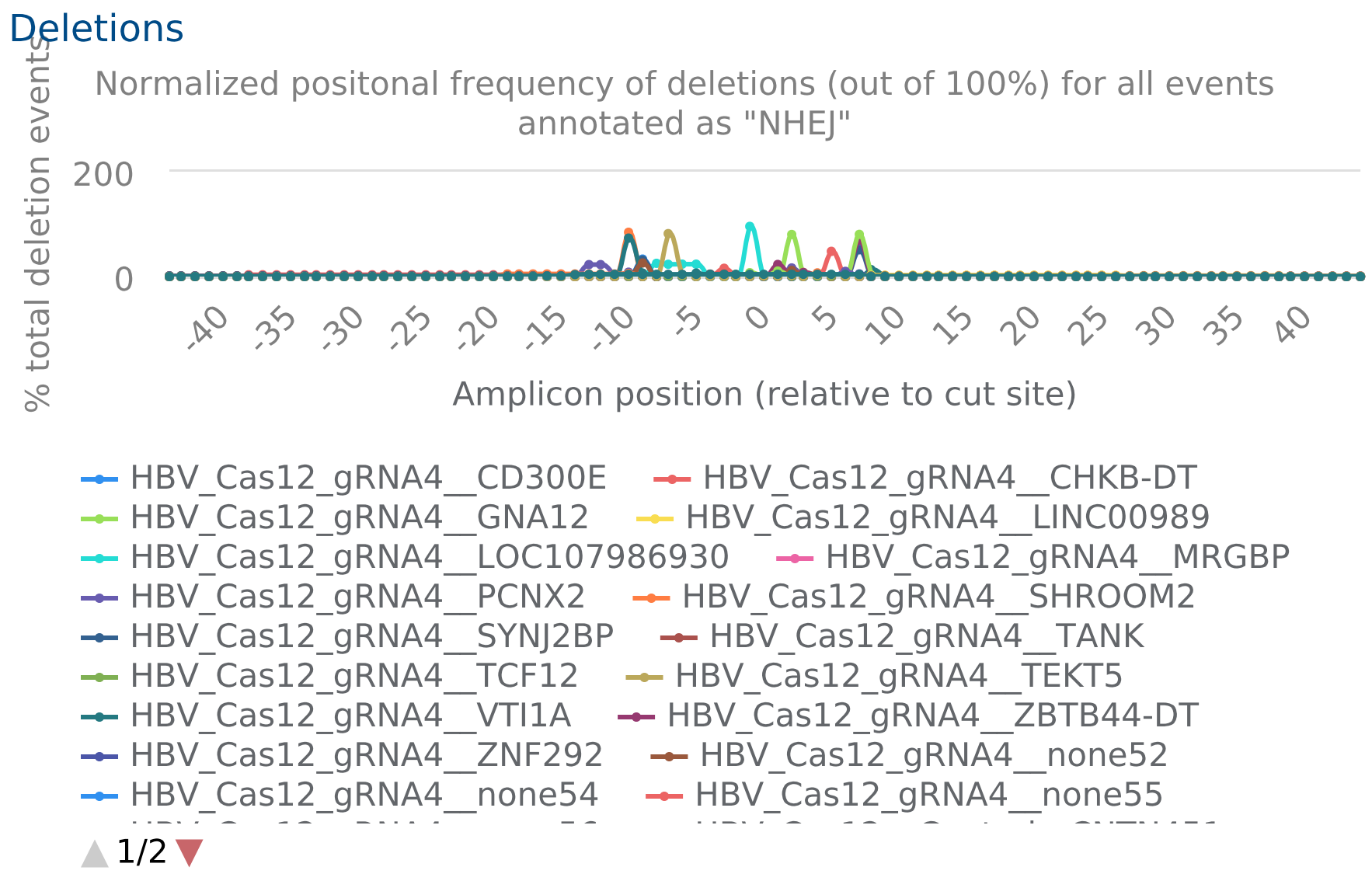

Indel Frequencies

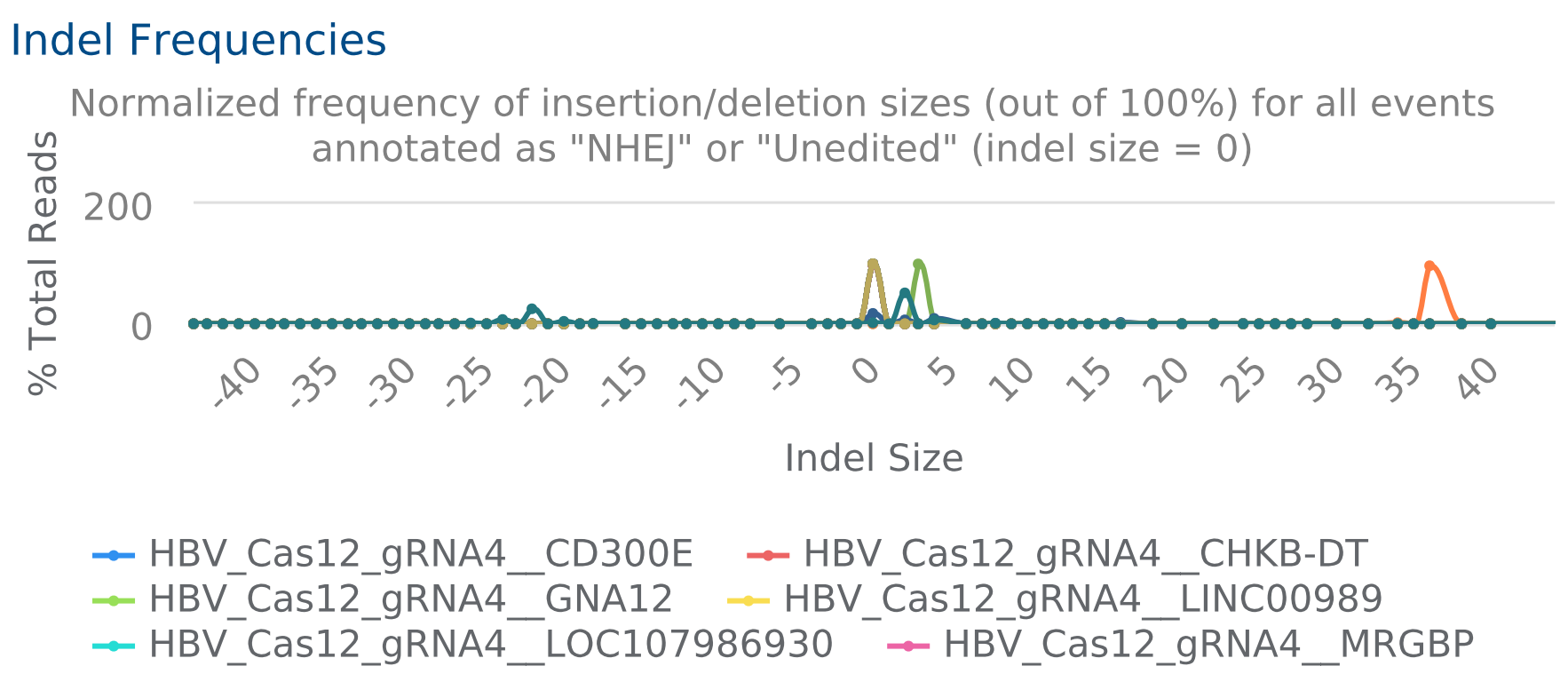

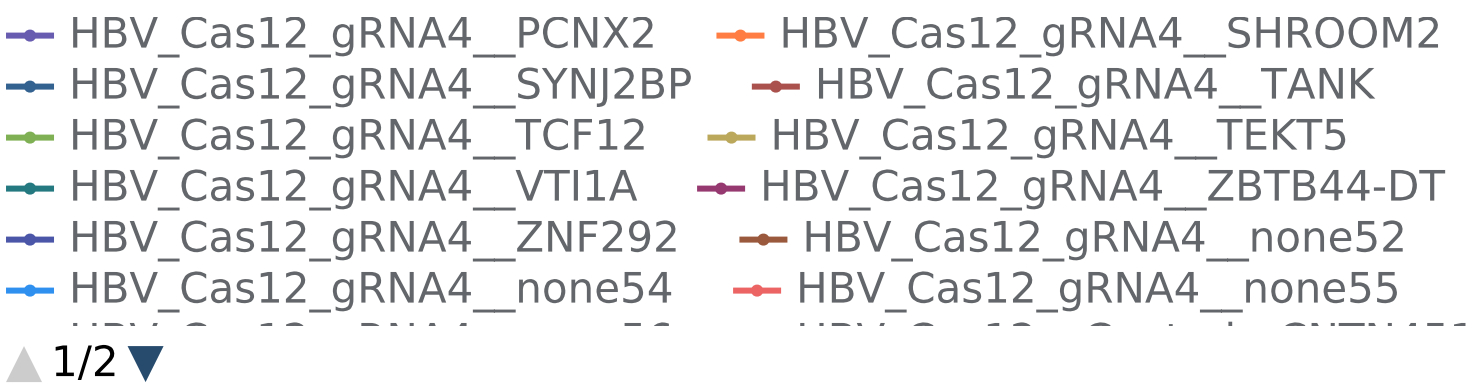
HBV_Cas12_gRNA4__PCNX2
HBV_Cas12_gRNA4__SHROOM2
HBV_Cas12_gRNA4__SYNJ2BP
HBV_Cas12_gRNA4__TANK
HBV_Cas12_gRNA4__TCF12
HBV_Cas12_gRNA4__TEKT5
HBV_Cas12_gRNA4__VTI1A
HBV_Cas12_gRNA4__ZBTB44-DT
HBV_Cas12_gRNA4__ZNF292
HBV_Cas12_gRNA4__none52
HBV_Cas12_gRNA4__none54
HBV_Cas12_gRNA4__none55
1/2

SNP Graph

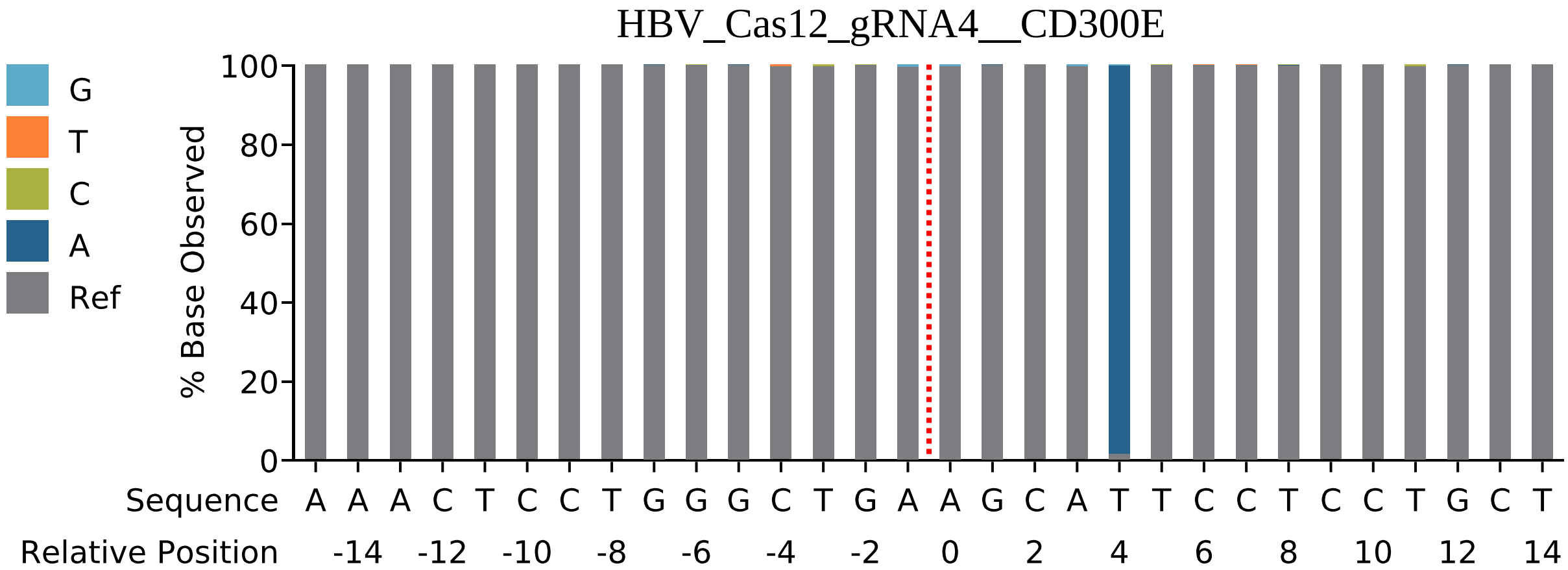
HBV_Cas12_gRNA4__CD300E
G
T
C
A
Ref
% Base Observed
100
80
60
40
20
0
Sequence A A A C T C C T G G G C T G A A G C A T T C C T C C T G C T
Relative Position -14 -12 -10 -8 -6 -4 -2 0 2 4 6 8 10 12 14

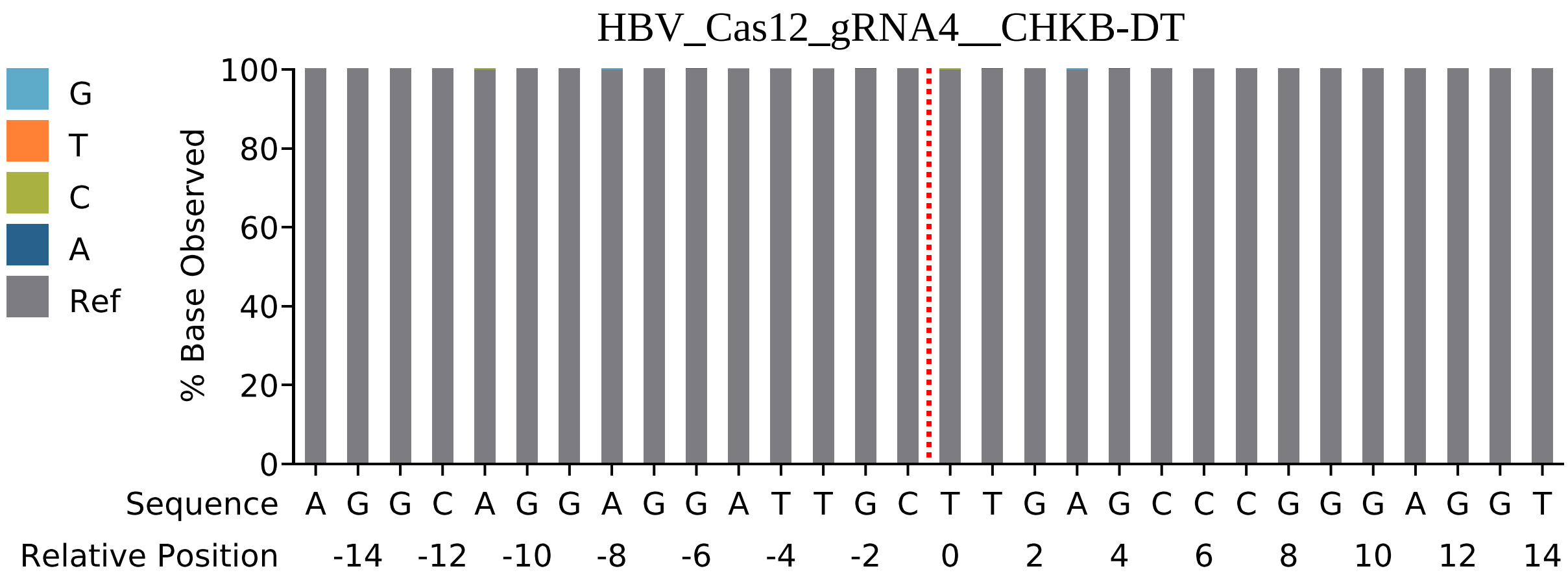
HBV_Cas12_gRNA4__CHKB-DT
G
T
C
A
Ref
% Base Observed
100
80
60
40
20
0
Sequence A G G C A G G A G G A T T G C T T G A G C C C G G G A G G T
Relative Position -14 -12 -10 -8 -6 -4 -2 0 2 4 6 8 10 12 14

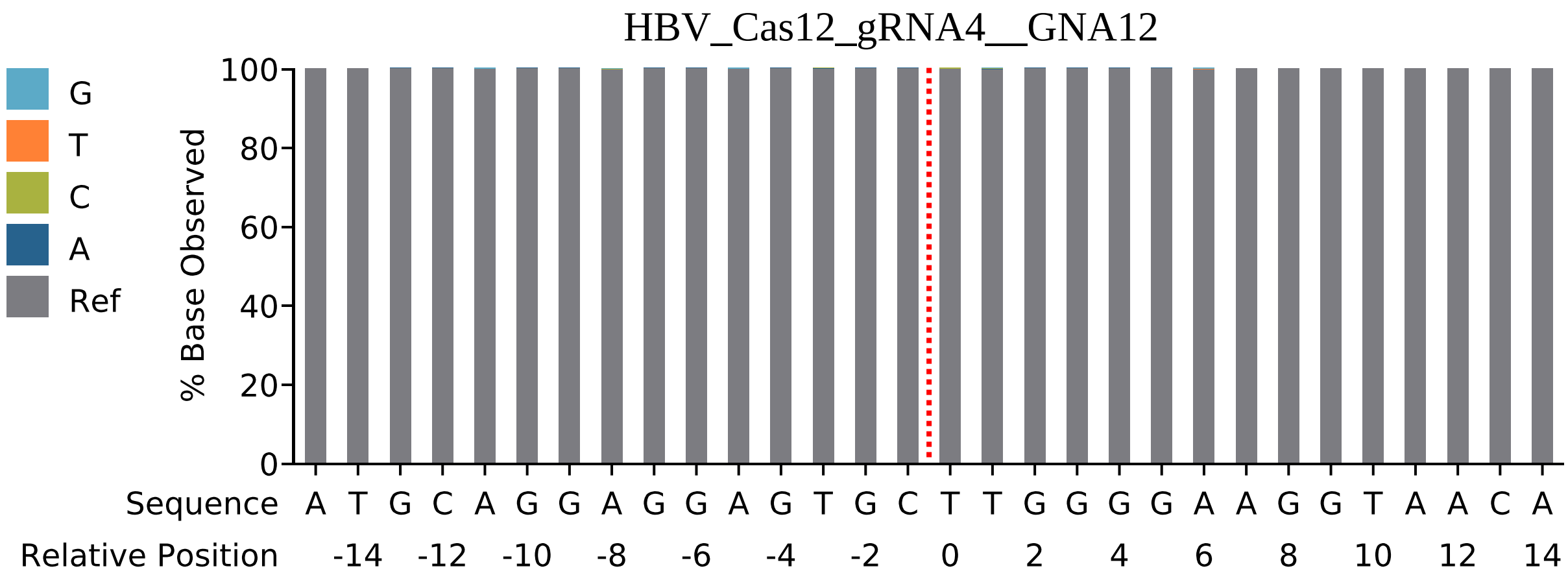
HBV_Cas12_gRNA4__GNA12
G
T
C
A
Ref
% Base Observed
100
80
60
40
20
0
Sequence A T G C A G G A G G A G T G C T T G G G G A A G G T A A C A
Relative Position -14 -12 -10 -8 -6 -4 -2 0 2 4 6 8 10 12 14

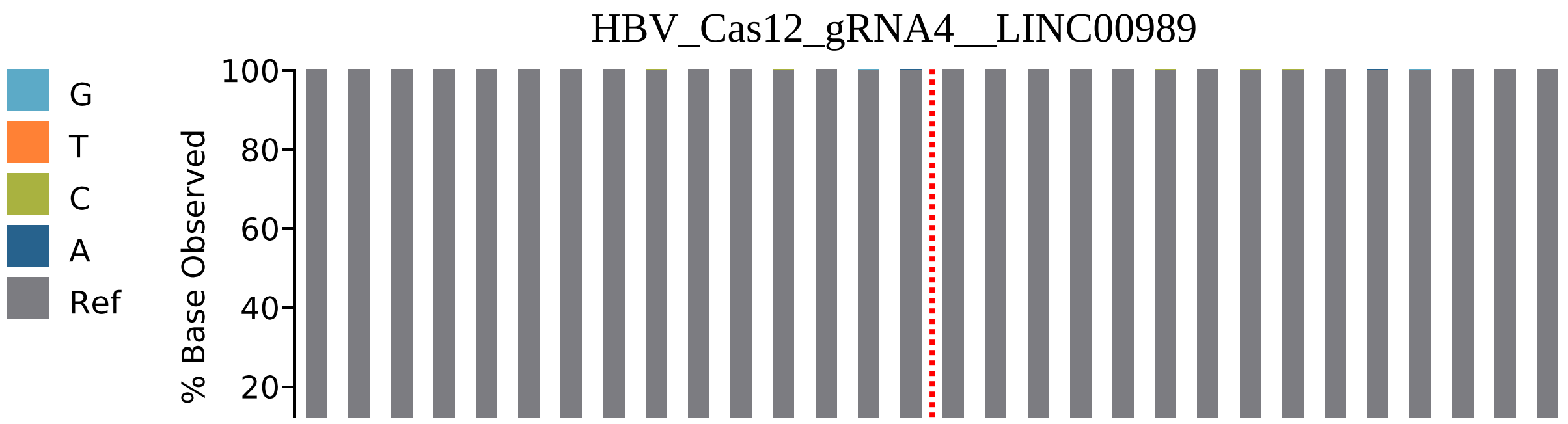
HBV_Cas12_gRNA4__LINC00989
G
T
C
A
Ref
% Base Observed
100
80
60
40
20

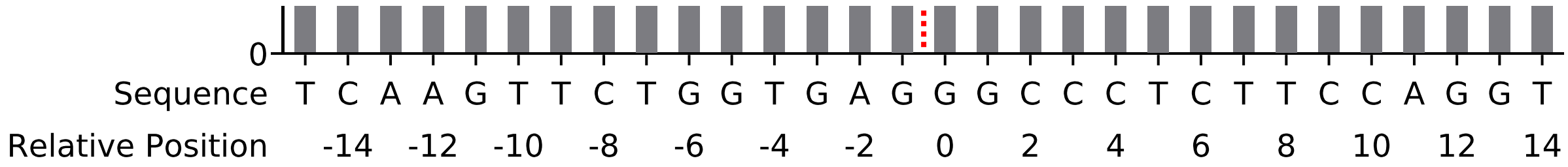

HBV_Cas12_gRNA4__LOC107986930

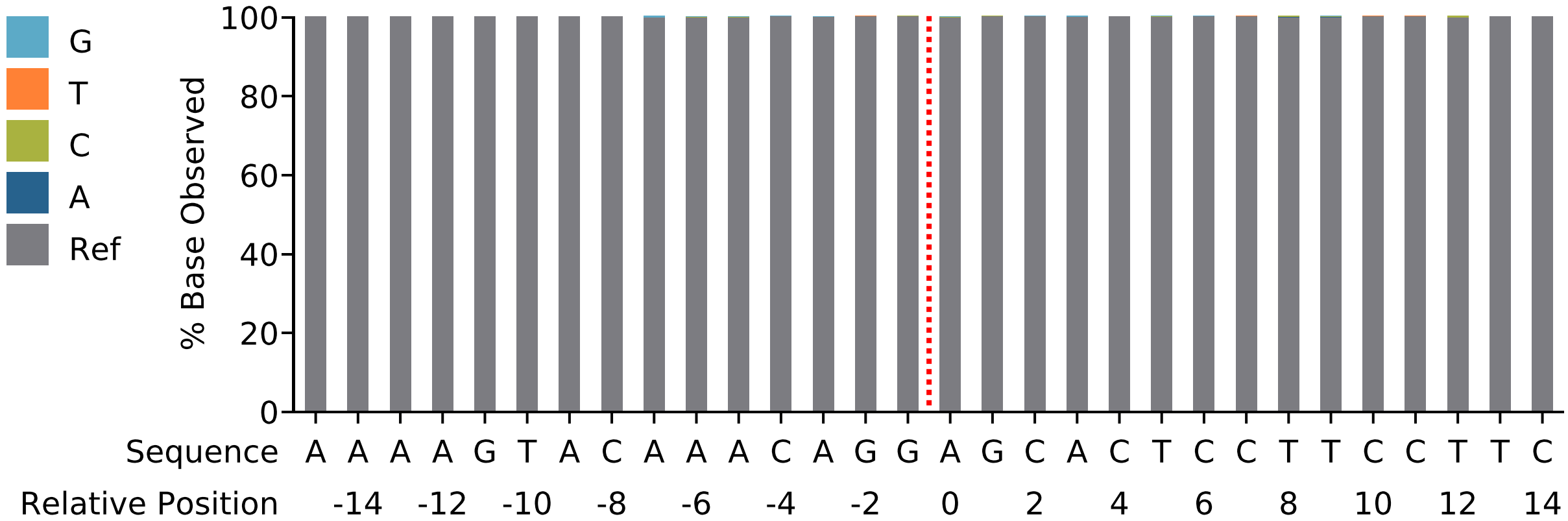

HBV_Cas12_gRNA4__MRGBP

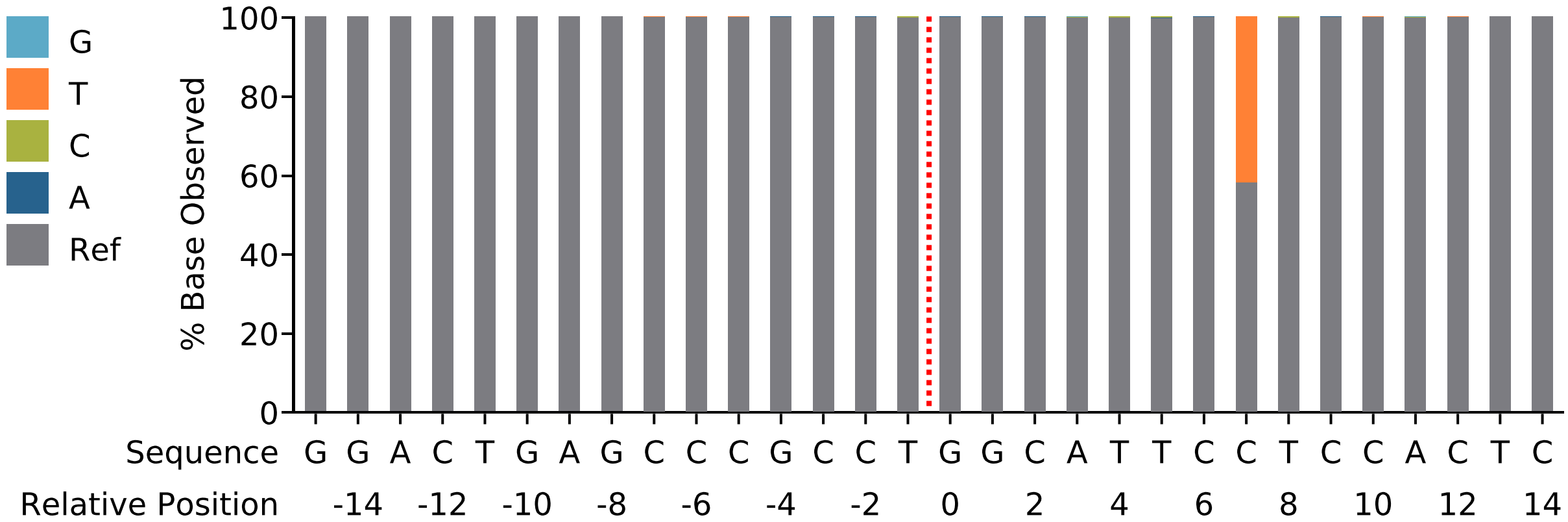

HBV_Cas12_gRNA4__PCNX2

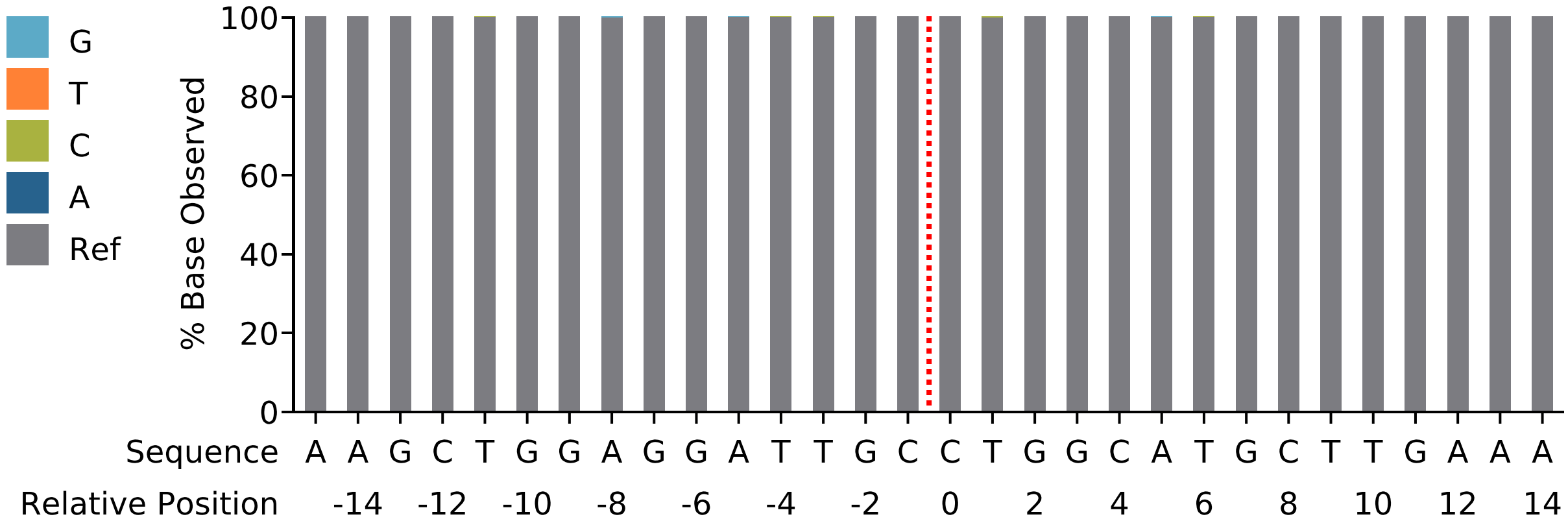

HBV_Cas12_gRNA4__SHROOM2

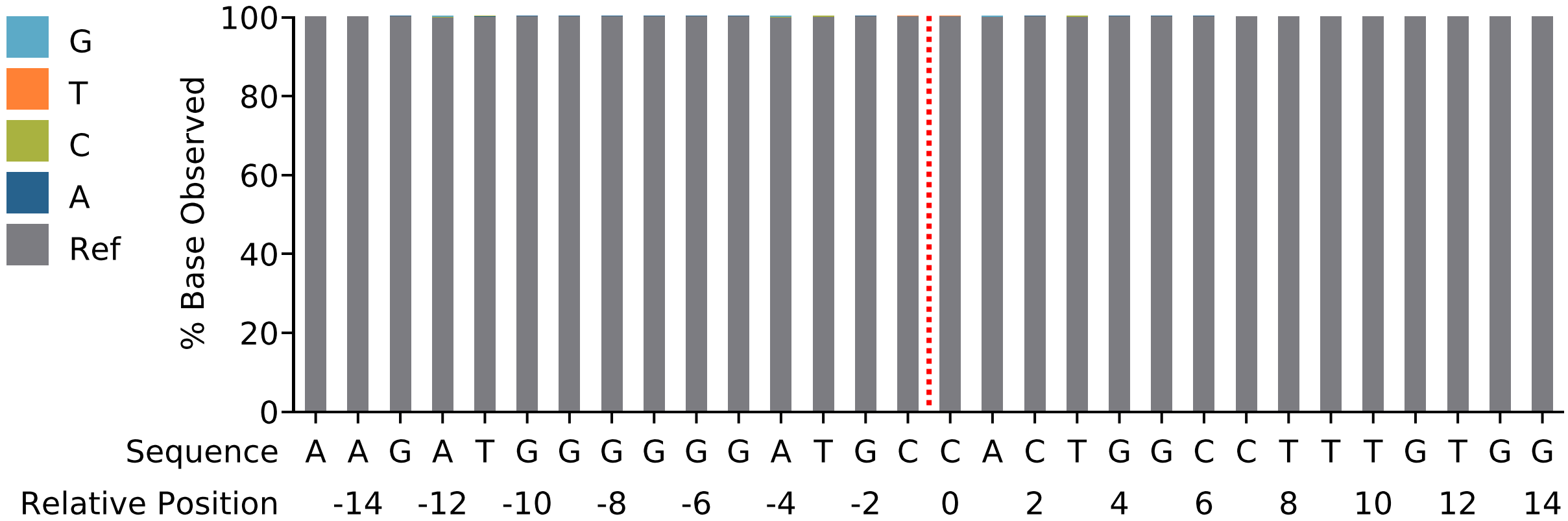

HBV_Cas12_gRNA4__SYNJ2BP

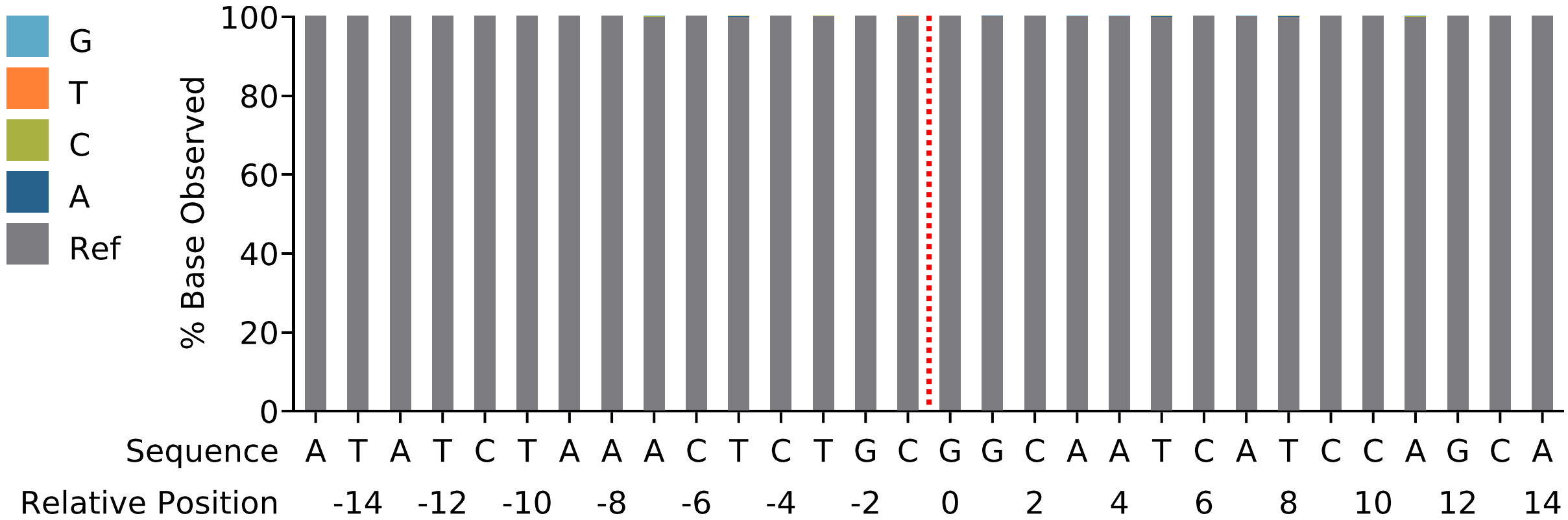

HBV_Cas12_gRNA4__TANK

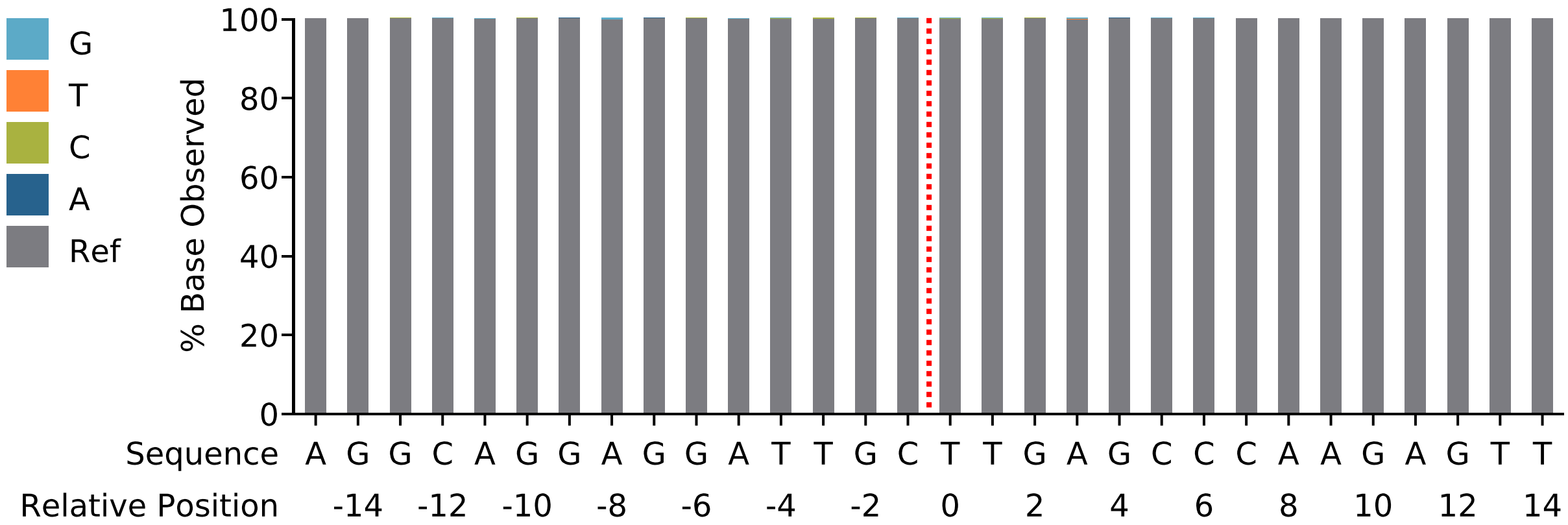

HBV_Cas12_gRNA4__TCF12

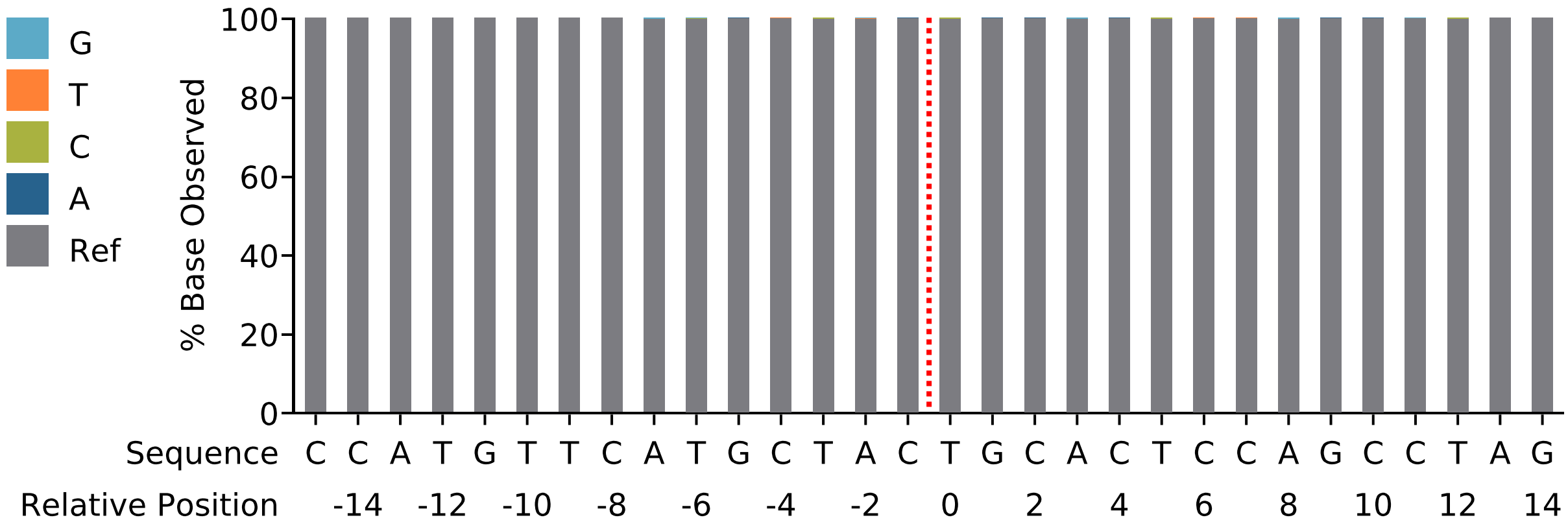

HBV_Cas12_gRNA4__TEKT5

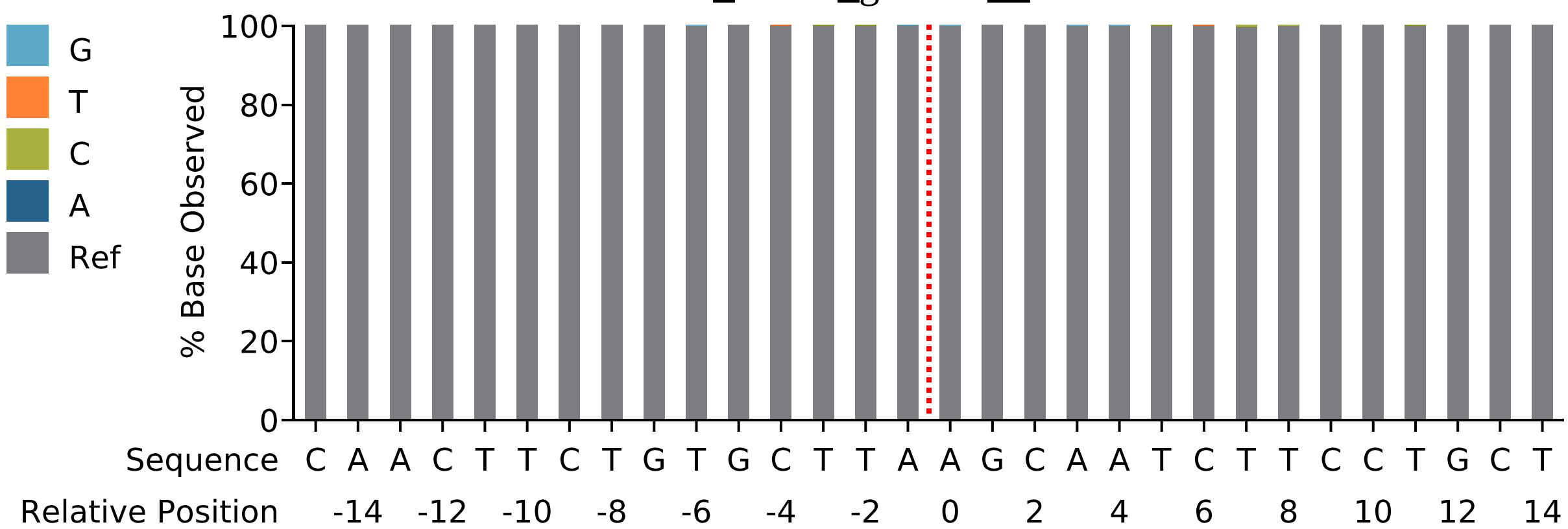

HBV_Cas12_gRNA4__VTI1A

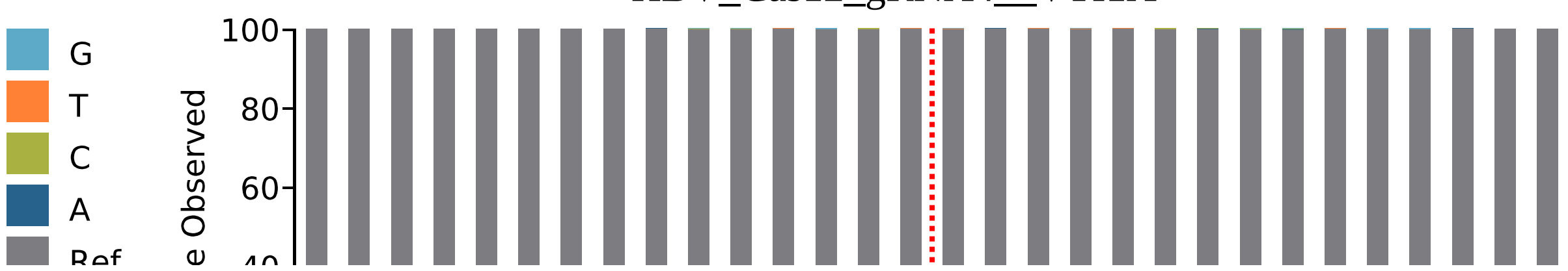

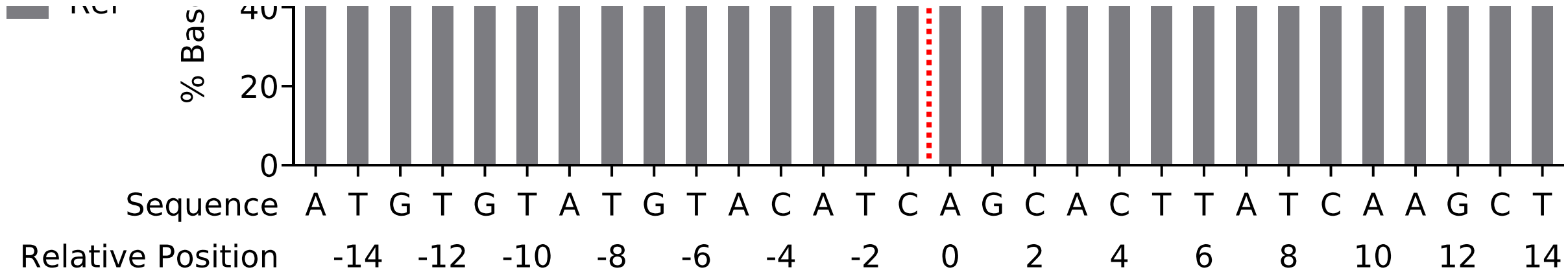

HBV_Cas12_gRNA4__ZBTB44-DT

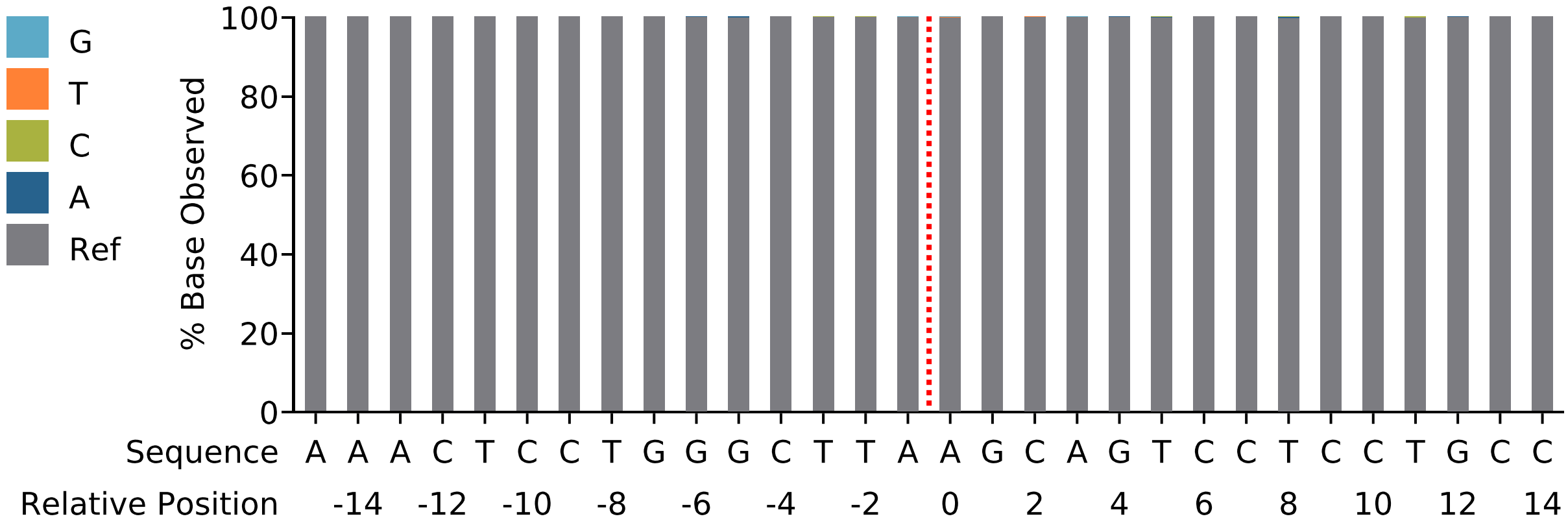

HBV_Cas12_gRNA4__ZNF292

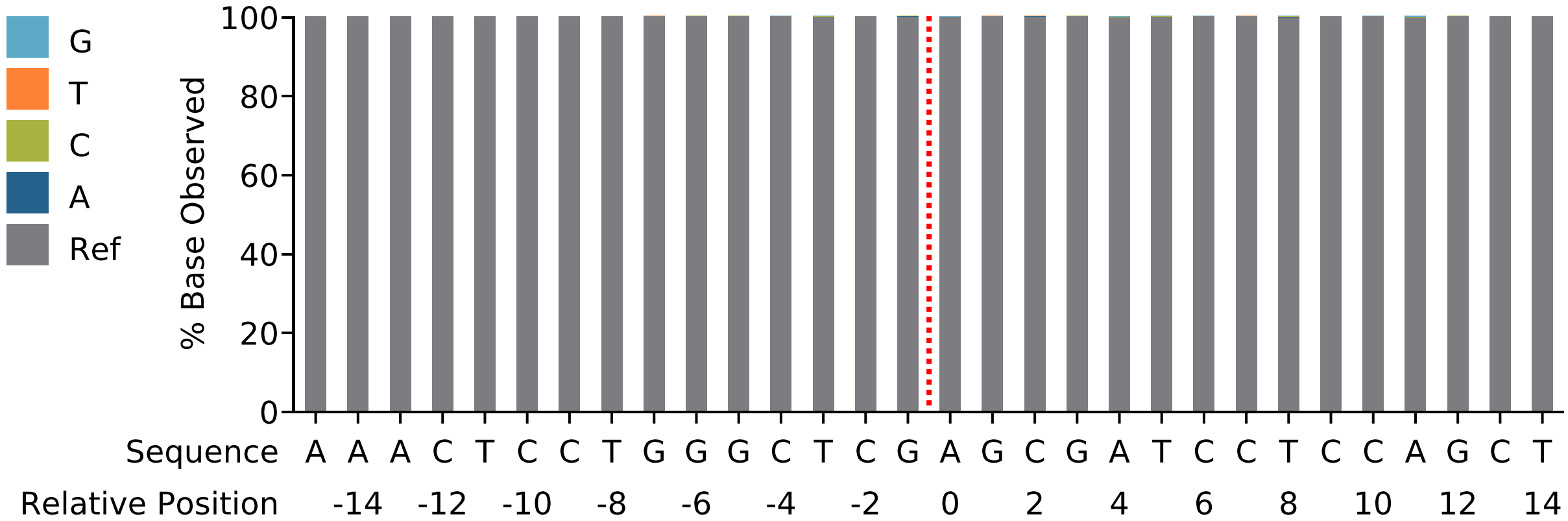

HBV_Cas12_gRNA4__none52

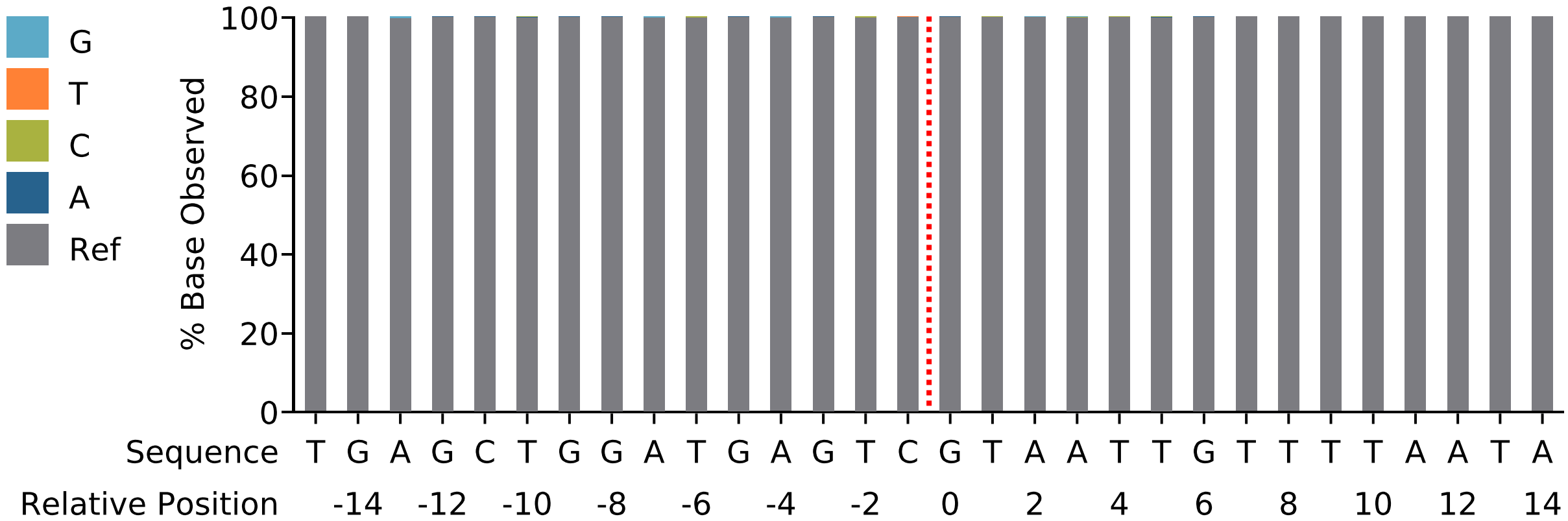

HBV_Cas12_gRNA4__none54

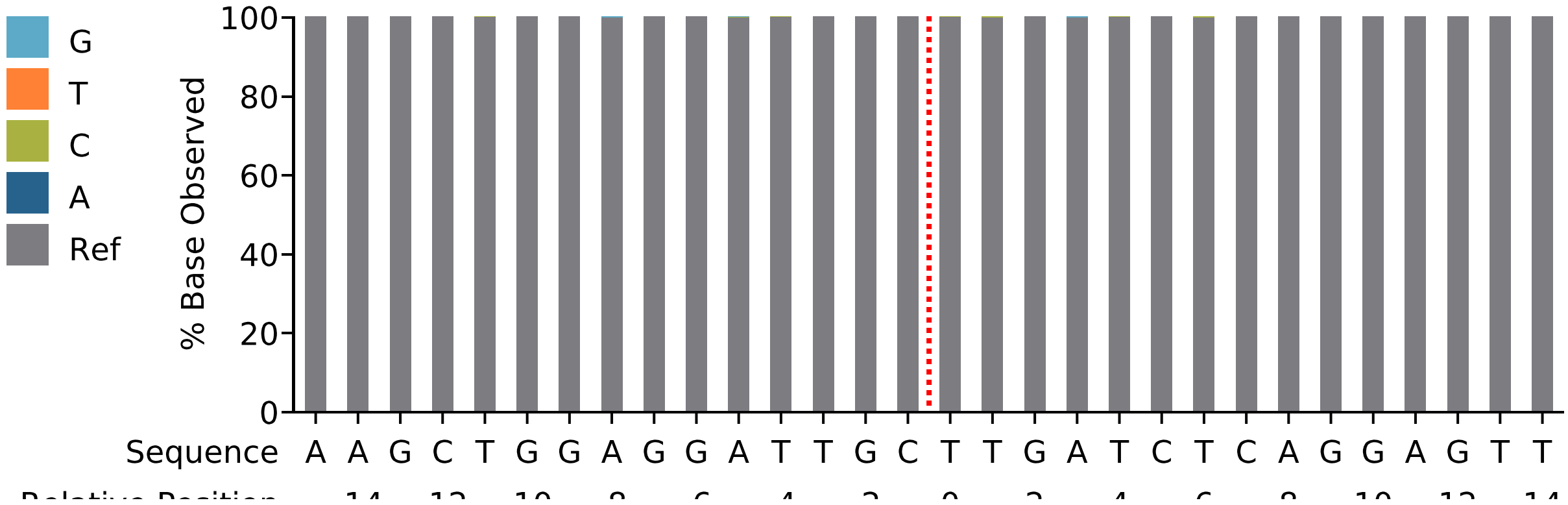

HBV_Cas12_gRNA4__none55

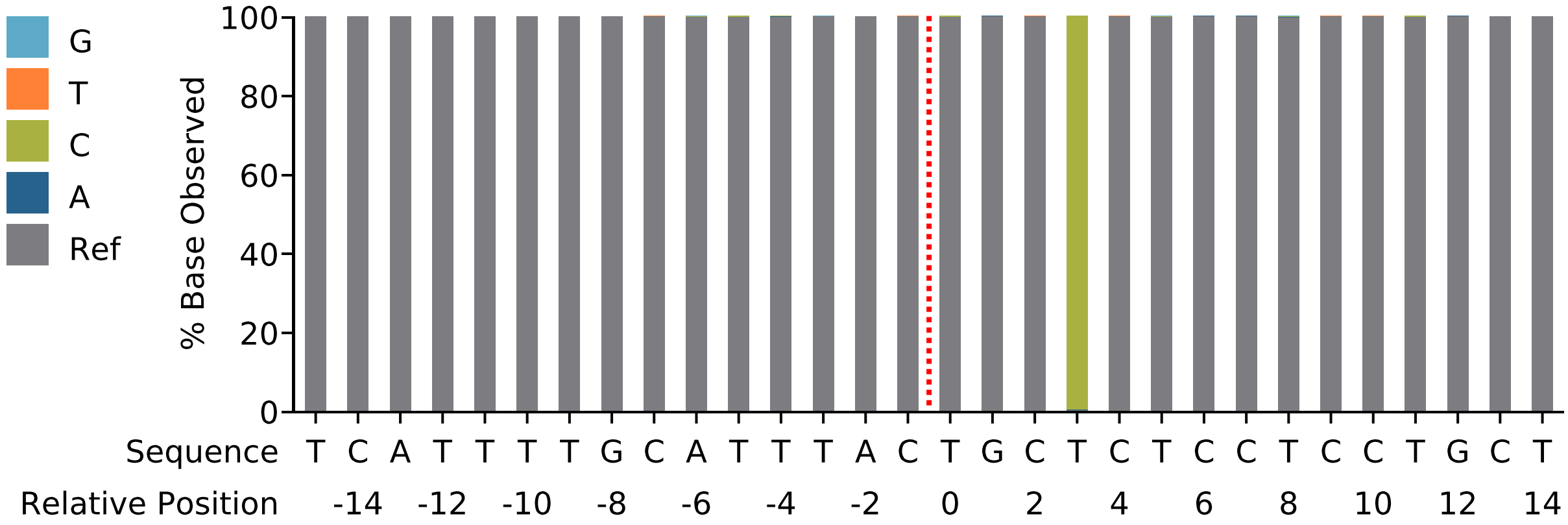

HBV_Cas12_gRNA4__none56

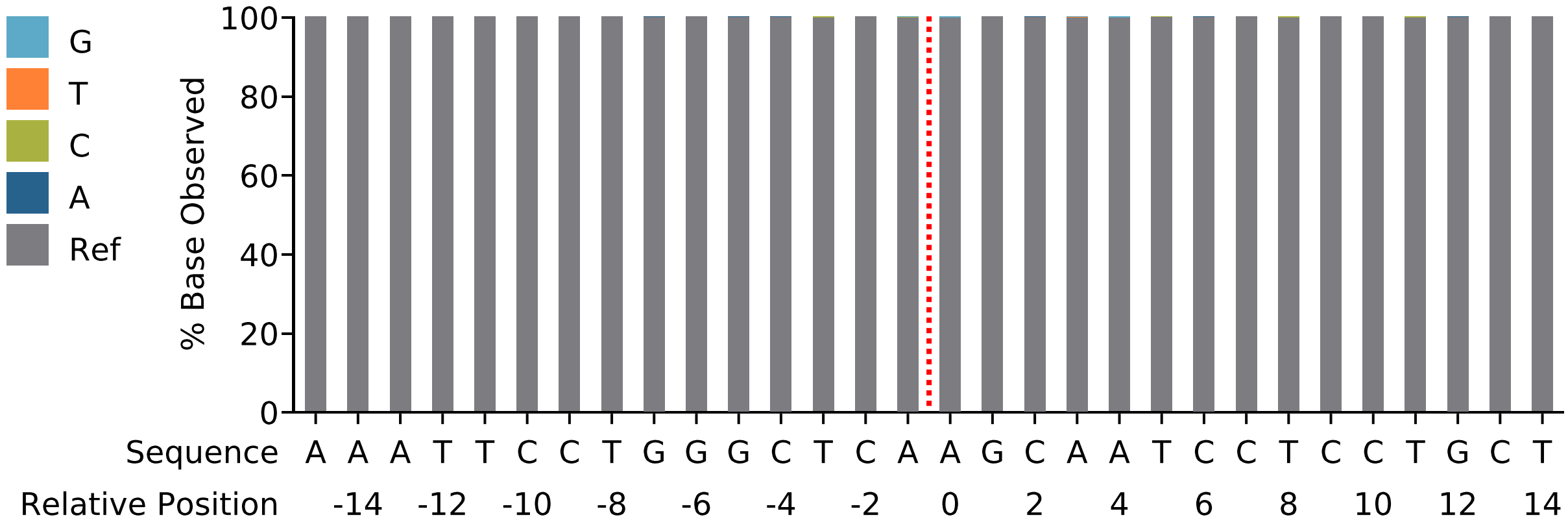

HBV_Cas12a_Control__CNTN451

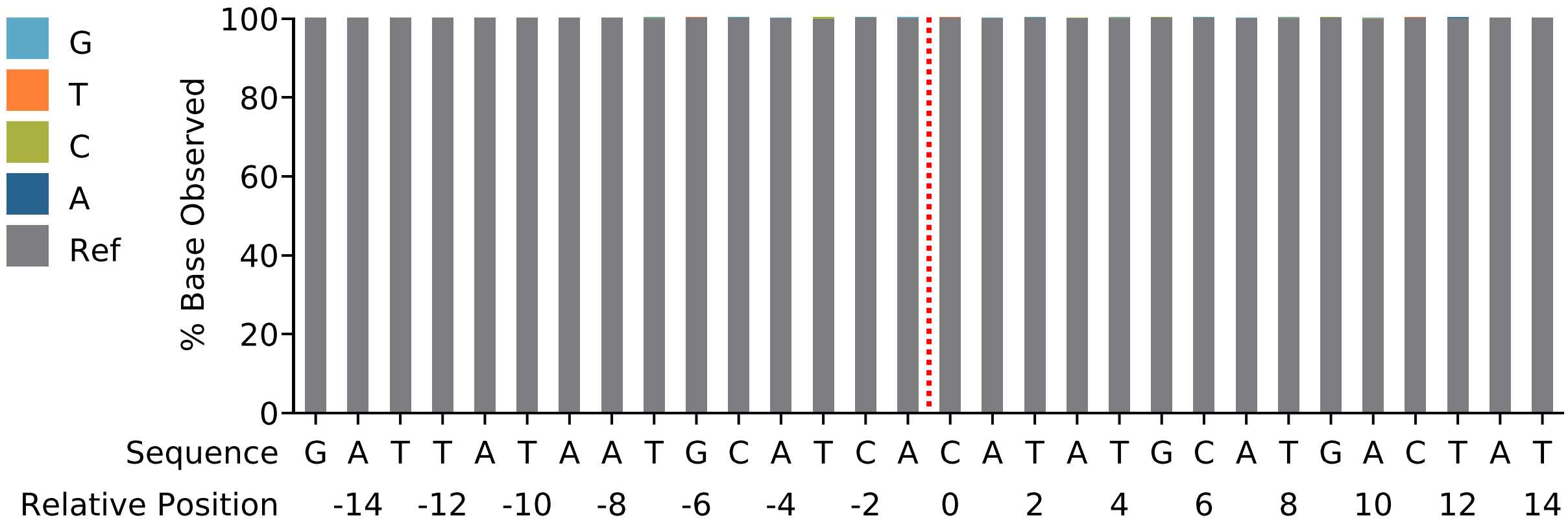

HBV_Cas12a_Control__POGLUT3

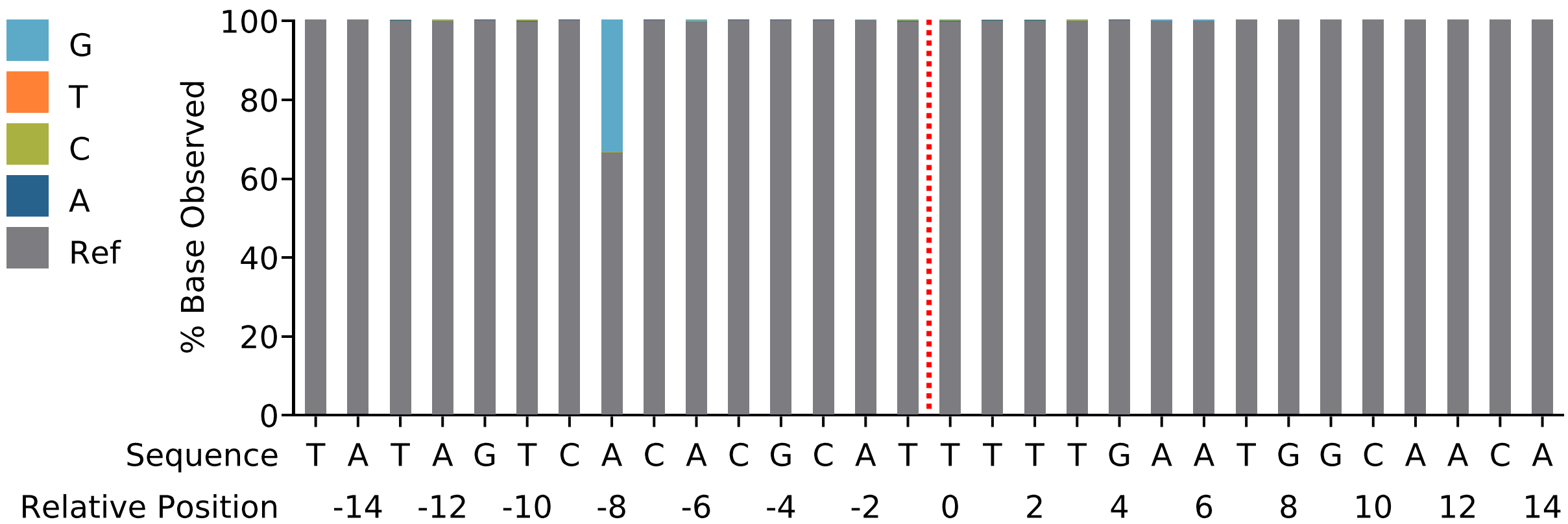

HBV_Cas12a_Control__ROR1

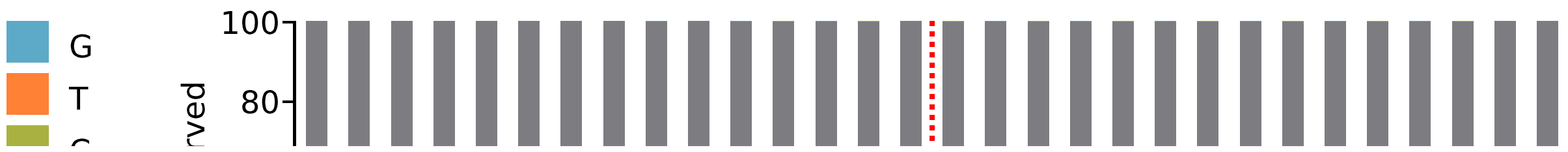

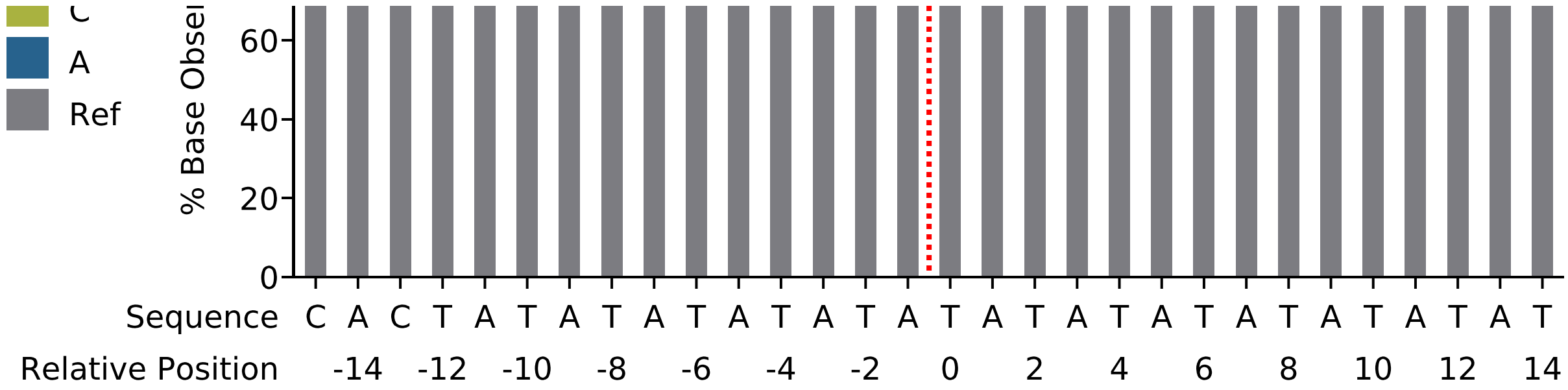

HBV_Cas12a_Control__SHISA6

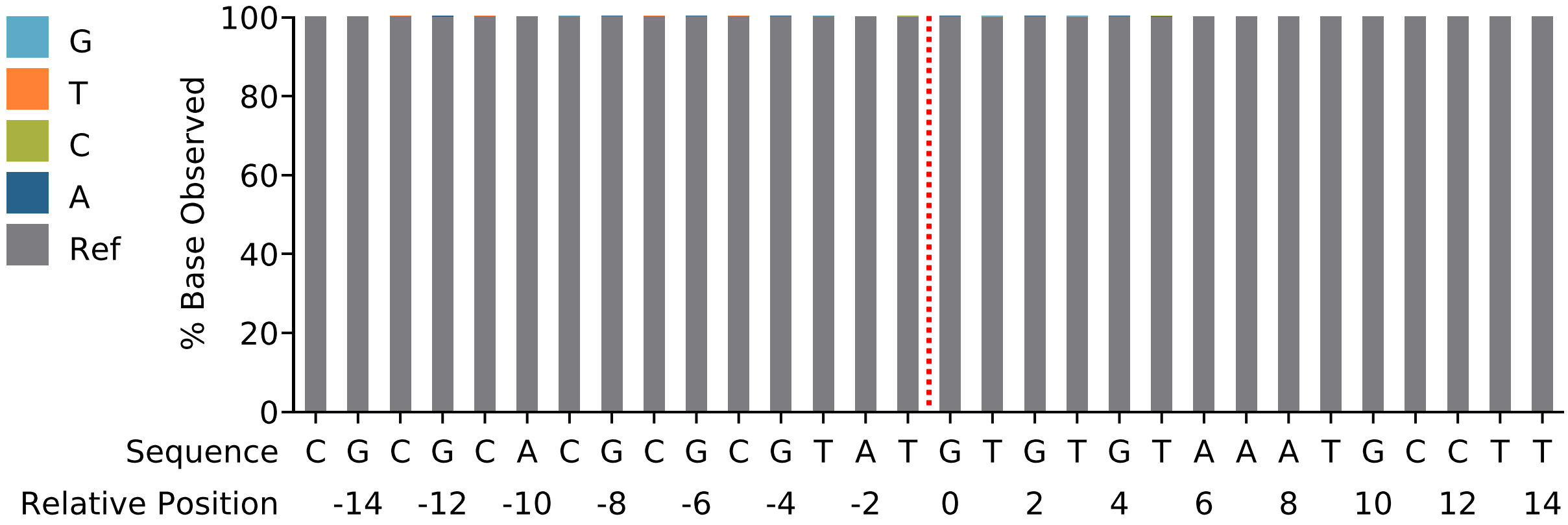

HBV_Cas12a_Control__TMC4

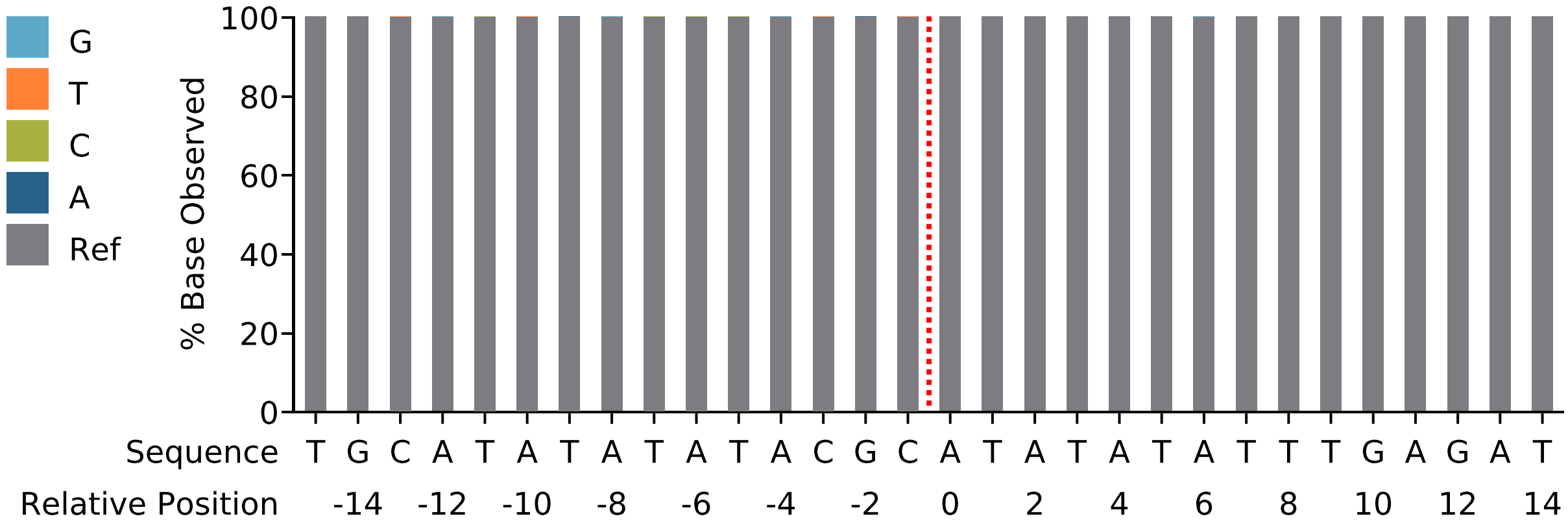

HBV_Cas12a_Control__none44

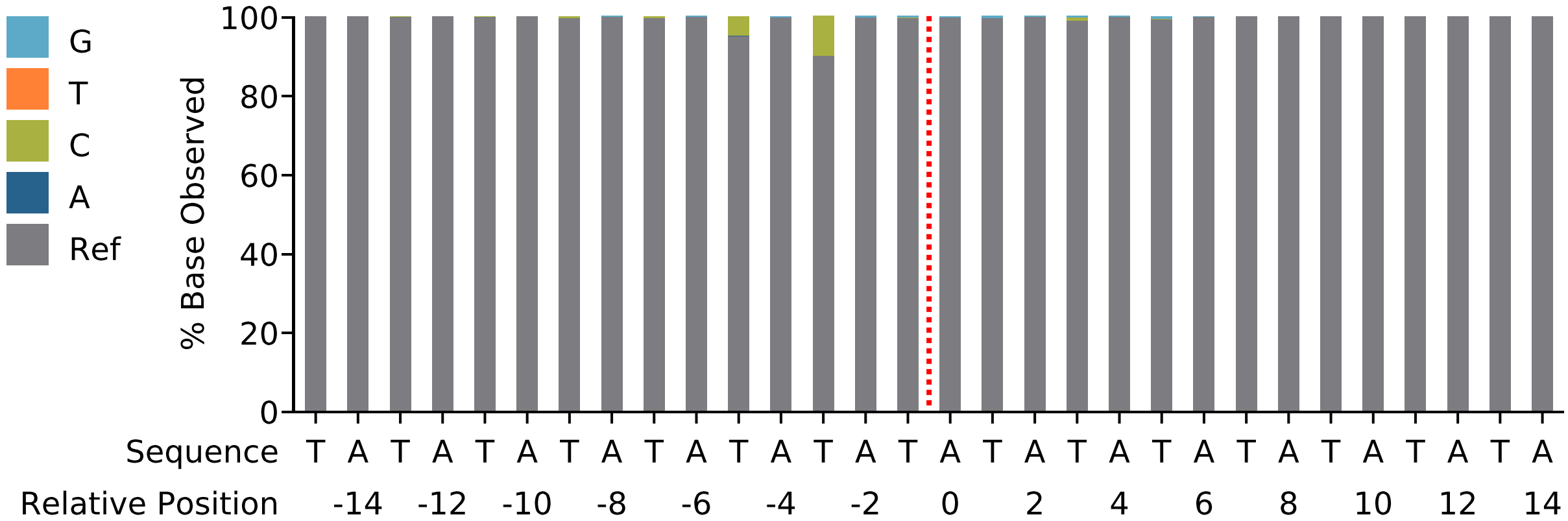

HBV_Cas12a_Control__none46

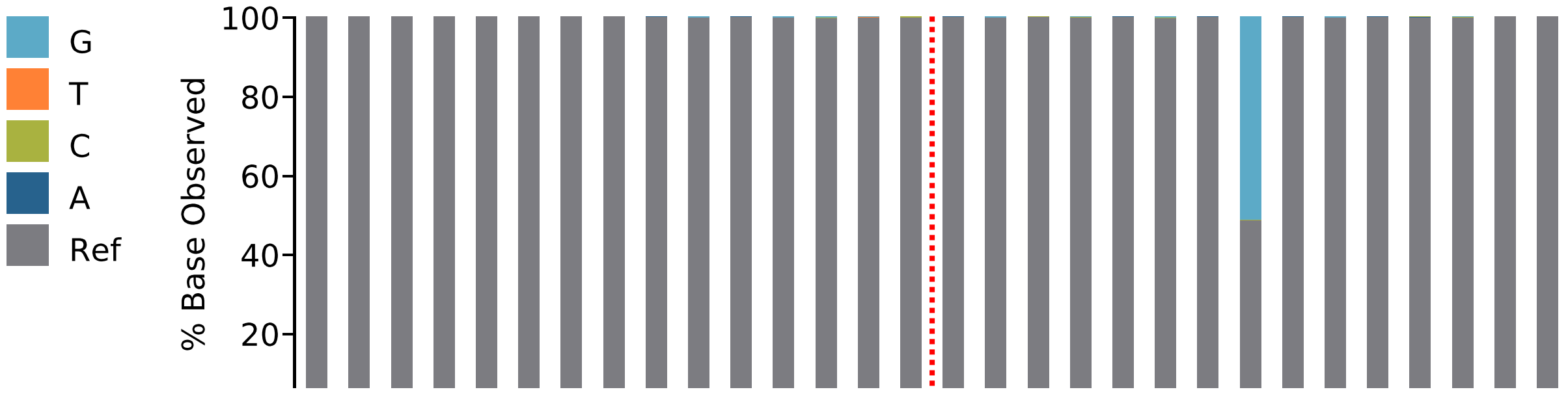

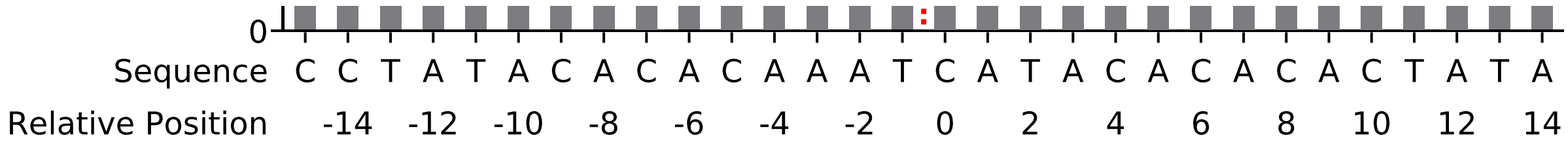

HBV_Cas12a_Control__none47

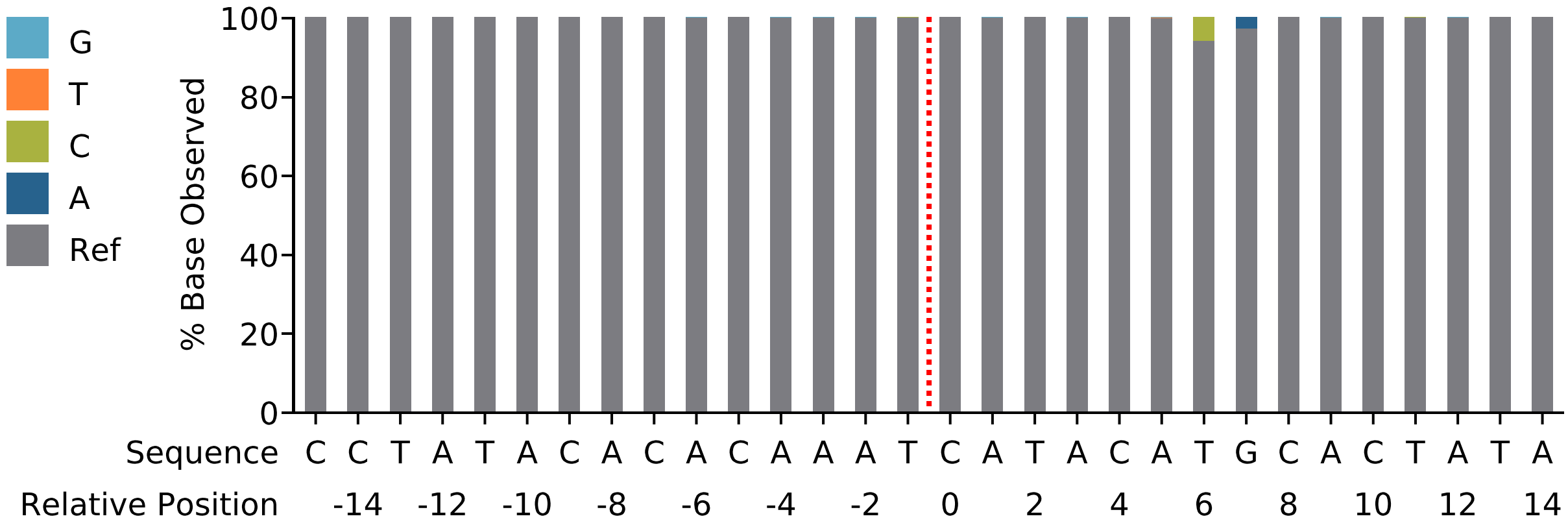

HBV_Cas12a_Control__none48

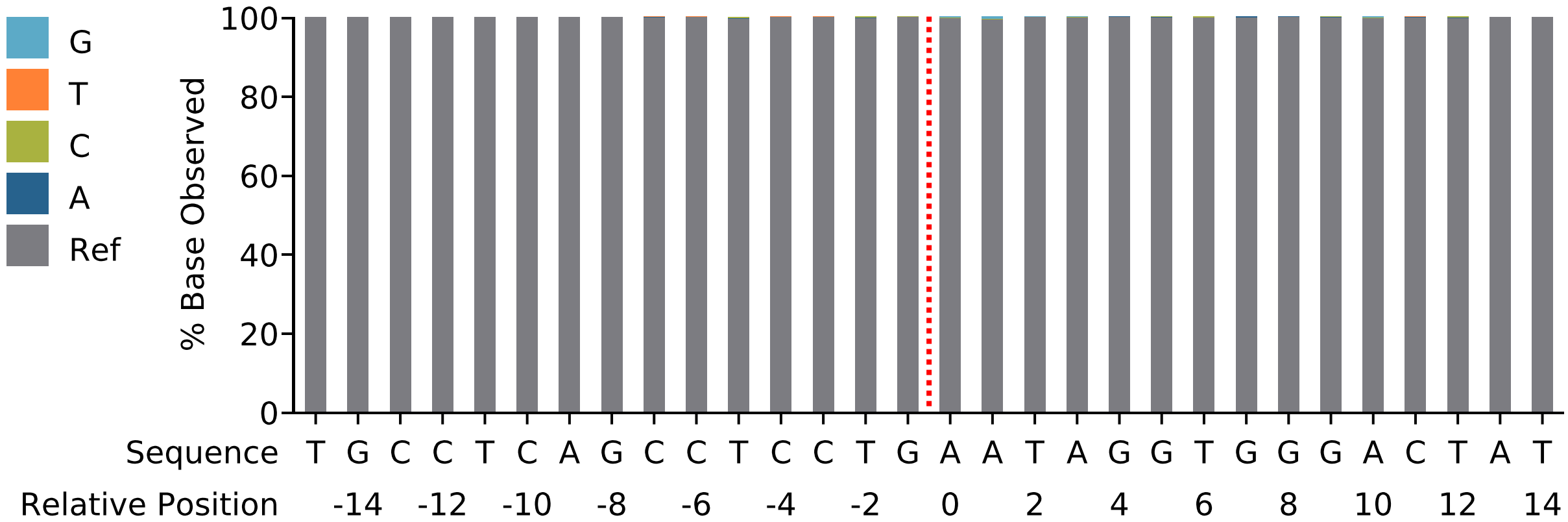

HBV_Cas12a_Control__none49

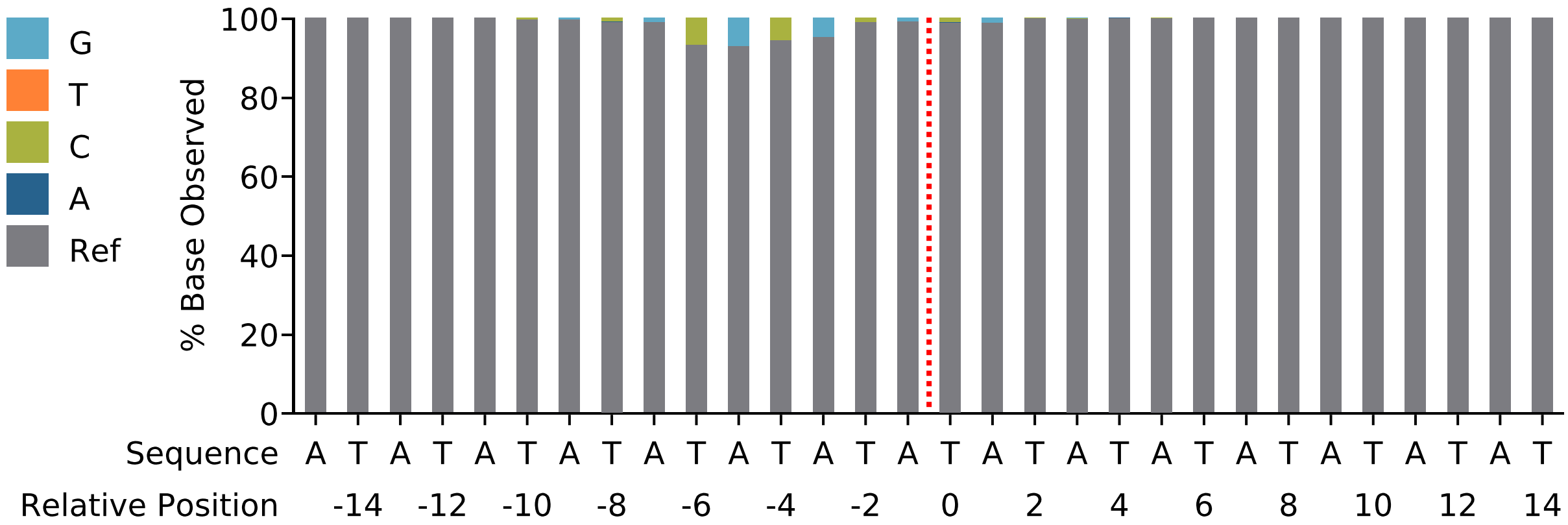

Supplement: Supplementary file 8 [file DataSheet8.pdf]
