# Supplementary material for: Combined RNA- and DNA-targeting CRISPR ribonucleoproteins enhance suppression of HBV transcripts
Source: Front Genome Ed. 2026 Sep 23;8:1824895. doi: 10.3389/fgeed.2026.1824895 (PMC13642003; doi:10.3389/fgeed.2026.1824895)
Supplement: Supplementary file 9 [file Table2.docx]

**Targeted rhAmpSeq Sequencing for In Silico–Predicted CRISPR Off-Target Analysis**

The sequences of the Cas9 control gRNA, Cas9 HBV gRNA9, Cas12a control gRNA, and Cas12a HBV gRNA4 were submitted to Integrated DNA Technologies (IDT, Coralville, IA, USA) for custom rhAmpSeq™ panel design. In collaboration with Admera Health, LLC (South Plainfield, NJ, USA), candidate off-target sites were identified by in silico analysis and reviewed based on genomic coordinates, reference genome assembly, target sequence, and strand orientation (listed in the Supplemental Excel file). For each gRNA, 20 candidate target regions were initially selected, prioritizing sites with high variant influence scores and representation across chromosomes and genomic features, including exons, introns, and intergenic regions. Target regions were provided as Browser Extensible Data (BED)-format files for primer design using the IDT rhAmpSeq Design Tool. Multiple primer-pair assays were generated for each target and evaluated for assay quality and compatibility with multiplex pooling. Assays compatible with the primary or secondary multiplex pools were retained, whereas assays incompatible with both pools or lacking a suitable design were excluded. Following quality control and multiplex compatibility assessment, 14, 19, 10, and 19 target sites (indicated by yellow highlighting in the Supplemental Excel file) were retained for the Cas9 control gRNA, Cas9 HBV gRNA9, Cas12a control gRNA, and Cas12a HBV gRNA4, respectively.

Genomic DNA was extracted from samples representing the gc/C9+gc/C12a, g9/C9+gc/C12a, gc/C9+g4/C12a, and g9/C9+g4/C12a combined treatment groups using QIAamp DNA Kit (QIAGEN, Hilden, Germany, Cat# 51304) according to the manufacturer's instructions. Fifty nanograms of genomic DNA per sample were used for targeted amplification with the custom rhAmpSeq primer panel. Amplicon libraries were generated using rhAmpSeq library reagents, followed by a second PCR to incorporate sample-specific sequencing indexes. Indexed libraries were purified, quantified, and pooled at equimolar concentrations based on quality-control measurements. Libraries were sequenced using an Illumina NovaSeq X Plus platform (San Diego, CA, USA) with 150-bp paired-end reads, generating approximately 3.3 million read pairs (6 Gb) per sample. Sequencing data were used to assess sequence variation at the selected predicted off-target sites in comparison with hg38, the human reference genome assembly GRCh38.p14 (NCBI accession GCA_000001405.29).
